# Socioeconomic disparities in cancer incidence and mortality in England and the impact of age-at-diagnosis on cancer mortality

**DOI:** 10.1371/journal.pone.0253854

**Published:** 2021-07-14

**Authors:** Ayşe Arık, Erengul Dodd, Andrew Cairns, George Streftaris

**Affiliations:** 1 Mathematical and Computer Sciences, Heriot-Watt University, Edinburgh, United Kingdom; 2 Maxwell Institute for Mathematical Sciences, Edinburgh, United Kingdom; 3 Mathematical Sciences, University of Southampton, Southampton, United Kingdom; University of Oxford, UNITED KINGDOM

## Abstract

**Background:**

We identify socioeconomic disparities by region in cancer morbidity and mortality in England for all-cancer and type-specific cancers, and use incidence data to quantify the impact of cancer diagnosis delays on cancer deaths between 2001–2016.

**Methods and findings:**

We obtain population cancer morbidity and mortality rates at various age, year, gender, deprivation, and region levels based on a Bayesian approach. A significant increase in type-specific cancer deaths, which can also vary among regions, is shown as a result of delay in cancer diagnoses. Our analysis suggests increase of 7.75% (7.42% to 8.25%) in female lung cancer mortality in London, as an impact of 12-month delay in cancer diagnosis, and a 3.39% (3.29% to 3.48%) increase in male lung cancer mortality across all regions. The same delay can cause a 23.56% (23.09% to 24.30%) increase in male bowel cancer mortality. Furthermore, for all-cancer mortality, the highest increase in deprivation gap happened in the East Midlands, from 199 (186 to 212) in 2001, to 239 (224 to 252) in 2016 for males, and from 114 (107 to 121) to 163 (155 to 171) for females. Also, for female lung cancer, the deprivation gap has widened with the highest change in the North West, e.g. for incidence from 180 (172 to 188) to 272 (261 to 282), whereas it has narrowed for prostate cancer incidence with the biggest reduction in the South West from 165 (139 to 190) in 2001 to 95 (72 to 117) in 2016.

**Conclusions:**

The analysis reveals considerable disparities in all-cancer and some type-specific cancers with respect to socioeconomic status. Furthermore, a significant increase in cancer deaths is shown as a result of delays in cancer diagnoses which can be linked to concerns about the effect of delay in cancer screening and diagnosis during the COVID-19 pandemic. Public health interventions at regional and deprivation level can contribute to prevention of cancer deaths.

## Introduction

Cancer is one of the major causes of morbidity and mortality in England and Wales. The Office for National Statistics (ONS) reported that cancer has remained the main cause of death since 2011 by accounting for 28.1% of all deaths in 2017 [1, 2]. Socioeconomic inequalities in cancer morbidity, mortality or survival have been well documented for type-specific cancers in different countries [3–18]. On the other hand, changes in type-specific cancer mortality and incidence rates by region and socioeconomic deprivation are less well-characterised in England [19–26]. Regional differences in all-cancer and the most common cancer types have been recently highlighted as a significant issue for cancer morbidity in England between 1981 and 2016 [19].

In this paper we focus on socioeconomic disparities for cancer morbidity and mortality in nine regions in England for the period 2001–2016. By bringing together morbidity and mortality data, we also quantify the impact of cancer diagnosis delays on cancer deaths using a model that links cancer morbidity and mortality. Differences in cancer mortality and morbidity have been addressed in previously published work [19, 20, 23, 24]. Yet cancer morbidity and mortality have not been linked in the literature using a modelling approach. Our research provides important findings related to socioeconomic inequalities in cancer morbidity and mortality in England, based on comprehensive modelling of relevant rates. Specifically, we investigate socioeconomic disparities by region in all-cancer, lung, bowel, prostate and breast cancer morbidity and mortality in England. We examine trends and differences between the most and least affected groups of different deprivation levels in each region of England, specifically the North East, the North West, Yorkshire and the Humber, the East Midlands, the West Midlands, the East, London, the South East and the South West, allowing to identify groups at greatest risk of cancer incidence and mortality. Importantly, we also associate cancer morbidity and mortality through average age-at-diagnosis and quantify the impact of cancer diagnosis delays on cancer mortality.

We use national statistics data on cancer registrations and deaths by age group from 2001 to 2016, to estimate region- and deprivation-specific trends and variations in different types of cancer for both genders in England. Specifically, we investigate incidence and mortality for the four most common types of cancer, i.e. malignant neoplasm of lung and neighbouring sites (trachea and bronchus), colorectal (or bowel) cancer, prostate cancer and breast cancer, along with all-cancer rates.

## Materials and methods

### Data

In this population-based modelling study, we use data for cancer registration numbers and deaths for the 9 regions of England (according to Nomenclature of Territorial Units for Statistics) from 2001 to 2016, provided by the ONS [27]. The data are split by individual cancer site code based on the International Statistical Classification of Diseases and Related Health Problems (ICD-10), five-year age-bands, year, gender, the Index of Multiple Deprivation (IMD), and region. Age-, deprivation- and region-specific population counts are also obtained by the ONS. This represents a richer and more informative data set compared to that used by the authors in previous work [19], as the inclusion of the IMD factor adds a classification layer and higher resolution to the data.

The IMD is an official relative measure of deprivation designed for small areas of England, based on seven domains of deprivation with different weights, i.e. income deprivation (22.5%), employment deprivation (22.5%), education, skills and training deprivation (13.5%), health deprivation and disability (13.5%), crime (9.3%), barriers to housing and services (9.3%), and living environment deprivation (9.3%). It divides the population in 10 equal deciles, where Decile/Level 1 represents the most deprived group and Decile/Level 10 shows the least deprived group.

Age-at-diagnosis in cancer registrations ranges from zero to 95+, with groups 0, 1–4, 5–9, …, 95+, and age at death starts from 20–24 and goes to 85+. Population counts start from age 20–24 and go through to age 85+. As the last age group for cancer registrations is 95+ but 85+ for cancer deaths and population counts, we grouped the last age in cancer registrations and deaths from age 85 onwards. For modelling purposes, each five-year age-band is represented by the relevant midpoint of the group and the last age group is represented by age 90.

### Statistical modelling

We examine trends and disparities in cancer registrations and deaths by employing a hierarchical Bayesian setting. Type-specific cancer registrations and deaths are modelled separately, since incidence and mortality levels and trends vary by type of cancer and type of observation. Whilst we follow the common tendency in the literature and assume a Poisson distribution for cancer counts, we allow for overdispersion through a hierarchical Poisson-lognormal Bayesian model in order to take into account the heterogeneous structure of England population [19]. The general structure of the model is given as

$$\begin{array}{cc} C_{a,t,d,g,r} & ∼\text{Poisson}(\theta_{a,t,d,g,r}E_{a,t,d,g,r})\\ \theta_{a,t,d,g,r} & ∼\text{Lognormal}(\mu_{a,t,d,g,r},\sigma^{2})\\ \mu_{a,t,d,g,r} & =\beta X. \end{array}$$

Here, *C*_*a*,*t*,*d*,*g*,*r*_ shows either the number of cancer-specific registrations or deaths at age *a* in year *t* for gender *g* in region *r* and decile of deprivation *d*, whenever applicable. *E*_*a*,*t*,*d*,*g*,*r*_ and *θ*_*a*,*t*,*d*,*g*,*r*_ represent the corresponding population estimates, and incidence or mortality rates, respectively. The location parameter of the lognormal distribution, *μ*_*a*,*t*,*d*,*g*,*r*_, is formulated to take into account how incidence or mortality rates depend on the main covariates considered here, namely age, year, gender, deprivation and region, which are generally denoted as ***X*** with associated model parameters denoted by ***β***. We also construct potential two-way interaction terms between these main covariates, with relevant terms encompassed in vectors ***X*** and ***β***.

Moreover, the following non-informative prior distributions are assumed for model parameters, to reflect relative prior ignorance on their values:

$$\begin{array}{cc} \beta_{\text{j}} & ∼\text{Normal}(0,10^{4})\\ \sigma^{2} & ∼\text{Inverse-Gamma}(1,0.001), \end{array}$$

for *j* = 1, 2, …, *p* where *p* is the number of coefficients in each model. We fit the models using Markov chain Monte Carlo (MCMC) methodology, implemented in the Bayesian analysis software WinBUGS [28, 29].

For modelling purposes, age is considered as a categorical variable with different levels depending on cancer type, except for prostate cancer mortality where it is included as a numerical variable in the best fitted model (Table 1). Whilst all-cancer and breast cancer incidence are modelled from age 22 onwards, other cancer types are modelled from age 47 onwards following relevant literature [30–38]. For all-cancer mortality, the two youngest age groups, i.e. ages 20–24 and 25–29, are merged in one group due to the small numbers of cases, whereas we start modelling breast cancer mortality from age group 35–39 onwards (Table 1).

**Table 1:** Structure and levels of age covariate in the best fitted models for different types of cancer.

Type of cancer	Age
All-cancer morbidity	{22, 27, …, 82, 90}, categorical, 14 levels
Lung cancer morbidity	{47, 52, …, 82, 90}, categorical, 9 levels
Bowel cancer morbidity	{47, 52, …, 82, 90}, categorical, 9 levels
Prostate cancer morbidity	{47, 52, …, 82, 90}, categorical, 9 levels
Breast cancer morbidity	{22, 27, …, 82, 90}, categorical, 14 levels
All-cancer mortality	{25, 32, …, 82, 90}, categorical, 13 levels
Lung cancer mortality	{47, 52, …, 82, 90}, categorical, 9 levels
Bowel cancer mortality	{47, 52, …, 82, 90}, categorical, 9 levels
Prostate cancer mortality	{47, 52, …, 82, 90}, numerical
Breast cancer mortality	{37, 42, …, 82, 90}, categorical, 11 levels

Furthermore, year is used as a numerical variable, after being standardised to have a zero mean and unit variance. Region and deprivation enter the models as categorical variables with 9 and 10 levels, respectively. We also impose a sum-to-zero constraint in the estimation of categorical variables, to facilitate their interpretation.

We carry out a systematic variable selection procedure in R-INLA software to determine the best fitted model for incidence and mortality rates [39]. Two different criteria are used: Deviance Information Criterion (DIC) and Bayes factor [40, 41]. We start the variable selection with the simplest model, i.e. the null model, and add new variables provided that the new model suggests a better fit to the data. Specifically, the variables which imply a lower DIC or a Bayes factor greater than 3 are included in the best fitted models [40, 41].

We check the fit of the models using Pearson residuals, *r*_*a*,*t*,*d*,*g*,*r*_, across age *a* and year *t* for a given region *r*, deprivation decile *d*, and gender *g*. These are given as

$$\begin{array}{c} r_{a,t,d,g,r}=\frac{C_{a,t,d,g,r}-E\left(C_{a,t,d,g,r}\right)}{\sqrt{\text{Var}(C_{a,t,d,g,r})}}, \end{array}$$

where $E\left(C_{a,t,d,g,r}\right)={\hat{\theta }}_{a,t,d,g,r}E_{a,t,d,g,r}$ and Var(*C*_*a*,*t*,*d*,*g*,*r*_) = *E*(*C*_*a*,*t*,*d*,*g*,*r*_) × (1 + *E*(*C*_*a*,*t*,*d*,*g*,*r*_)exp(*σ*^2^ − 1)) [42]. The model fit appeared to be adequate in all cases (see S1 File for relevant plots).

We use age-standardised incidence and mortality rates to evaluate deprivation variation in cancer morbidity and mortality rates by region. Age-standardised rates were calculated by region and decile of deprivation for each gender based on the European Standard Population (ESP) 2013 structure, developed by the statistical office of the European Union, Eurostat [43].

#### Analysis based on age-standardisation

We quantified the deprivation gap over years in each region, whenever deprivation is a significant covariate in the relevant model, using absolute differences, denoted as AD_*t*,*r*_. This is based on the difference between the rates in the highest- and lowest-incidence or mortality deprivation decile in each region, separately for different types of cancer and gender, i.e.

$$\begin{array}{c} {\text{AD}}_{t,r}={\hat{\theta }}_{t,r}^{\text{max}}-{\hat{\theta }}_{t,r}^{\text{min}}, \end{array}$$

where ${\hat{\theta }}_{t,r}^{\text{max}}$ is the highest fitted rate in year *t* and region *r* across all deprivation deciles, and ${\hat{\theta }}_{t,r}^{\text{min}}$ is the relevant lowest fitted rate in the same year and region. Additionally, we considered a measure of overall-temporal-change, denoted as AC_*d*,*r*_, from 2001 to 2016 for each deprivation decile in each region, separately for different types of cancer and gender, as follows:

$$\begin{array}{c} {\text{AC}}_{d,r}={\hat{\theta }}_{2016,d,r}-{\hat{\theta }}_{2001,d,r}, \end{array}$$

where ${\hat{\theta }}_{2016,d,r}$ and ${\hat{\theta }}_{2001,d,r}$ are the corresponding age-standardised fitted rates in 2016 and 2001, for deprivation decile *d* in region *r*.

#### Average age-at-diagnosis

Age-at-diagnosis is considered an important risk factor for cancer mortality [44–47]. Hence, we estimated average age-at-diagnosis, denoted by *AAD*_*t*,*d*,*g*,*r*_ for deprivation decile *d*, region *r*, year *t* and gender *g*, using type-specific fitted incidence rates as follows:

$$\begin{array}{c} {\text{AAD}}_{t,d,g,r}=\frac{\sum_{a}a{\hat{\theta }}_{a,t,d,g,r}E_{a}^{\text{std}}}{\sum_{a}{\hat{\theta }}_{a,t,d,g,r}E_{a}^{\text{std}}}, \end{array}$$

where $E_{a}^{\text{std}}$ shows population numbers at age *a* according to the European Standard Population 2013, and ${\hat{\theta }}_{a,t,d,g,r}$ are the relevant fitted incidence rates. Average age-at-diagnosis is then weighted over years, to provide a cross-sectional measure that can be linked to mortality throughout the period 2001–2016. This is obtained as

$$\begin{array}{c} {\text{AAD}}_{d,g,r}=\frac{\sum_{t}{\text{AAD}}_{t,d,g,r}E_{t,d,g,r}}{\sum_{t}E_{t,d,g,r}}, \end{array}$$

where *E*_*t*,*d*,*g*,*r*_ are the population numbers (as provided by the ONS) aggregated over age groups. Whenever deprivation is not a significant variable in the relevant mortality model, *AAD*_*d*,*g*,*r*_ is averaged over deprivation deciles.

Average age-at-diagnosis is used as a numerical variable in our mortality models, with the aim to quantify its impact on mortality rates. Note that different standardisations are considered to facilitate convergence of the MCMC estimation algorithms (see S1 File for details). Also, average age-at-diagnosis is derived based on fitted incidence rates, where the best fitted models are mostly used. The only exceptions are bowel cancer and breast cancer where two additional two-way interaction terms (between age-deprivation, and age-region) are included in the models, in order to quantify relevant average age-at-diagnosis variables in a way that allows to distinguish between deprivation deciles and regions.

#### Best fitted models

Tables 2 and 3 show variables included in the best fitted models for different types of cancer morbidity and mortality, respectively. Note that interaction terms may include different powers of year, as well. All main covariates (i.e. age, year, gender, deprivation and region) are involved in the best fitted model for each type of cancer incidence. On the other hand, the deprivation variable has not improved the overall fit of models for prostate and breast cancer mortality rates. Therefore, for these cancer types the deprivation variable is not included in the final model and we have less complicated structures involving age, year, region and interaction terms.

**Table 2:** Variables included in the best fitted models for different types of cancer morbidity; main variables are denoted as age (a), year (t), gender (g), region (r), deprivation (d), with corresponding interactions shown as, e.g., a:t.

Cancer morbidity	Highest year power	r	d	a:t	a:g	a:d	a:r	t:g	t:d	t:r	g:d	g:r	d:r
Male all-cancer	3	yes	yes	yes	no	yes	no	no	no	yes	no	no	no
Female all-cancer	3	yes	yes	yes	no	yes	yes	no	no	yes	no	no	no
Lung cancer	1	yes	yes	yes	yes	yes	yes	yes	yes	yes	no	yes	yes
Bowel cancer	2	yes	yes	yes	yes	no	no	yes	no	no	yes	yes	no
Prostate cancer	3	yes	yes	yes	no	no	yes	no	yes	yes	no	no	yes
Breast cancer	1	yes	yes	yes	no	no	no	no	no	no	no	no	no

**Table 3:** Variables included in the best fitted models for different types of cancer mortality; main variables are denoted as age (a), year (t), gender (g), region (r), deprivation (d), average age-at-diagnosis (AAD), with corresponding interactions shown as, e.g., a:t.

Cancer mortality	Highest year power	r	d	a:t	a:g	a:d	a:r	t:g	t:d	t:r	g:d	g:r	d:r	AAD	AAD:region
Male all-cancer	2	yes	yes	yes	no	yes	no	no	yes	yes	no	no	no	yes	yes
Female all-cancer	1	yes	yes	yes	no	yes	no	no	yes	yes	no	no	no	yes	no
Male lung cancer	1	yes	yes	yes	no	yes	no	no	yes	no	no	no	no	yes	no
Female lung cancer	2	yes	yes	yes	no	yes	yes	no	yes	yes	no	no	no	yes	yes
Male bowel cancer	1	yes	yes	yes	no	no	no	no	no	no	no	no	no	yes	no
Female bowel cancer	1	no	yes	yes	no	no	no	no	no	no	no	no	no	yes	no
Prostate cancer	2	no	no	yes	no	no	no	no	no	no	no	no	no	yes	no
Breast cancer	1	yes	no	yes	no	no	no	no	no	no	no	no	no	yes	no

## Results

### All-cancer

**Fig 1:**
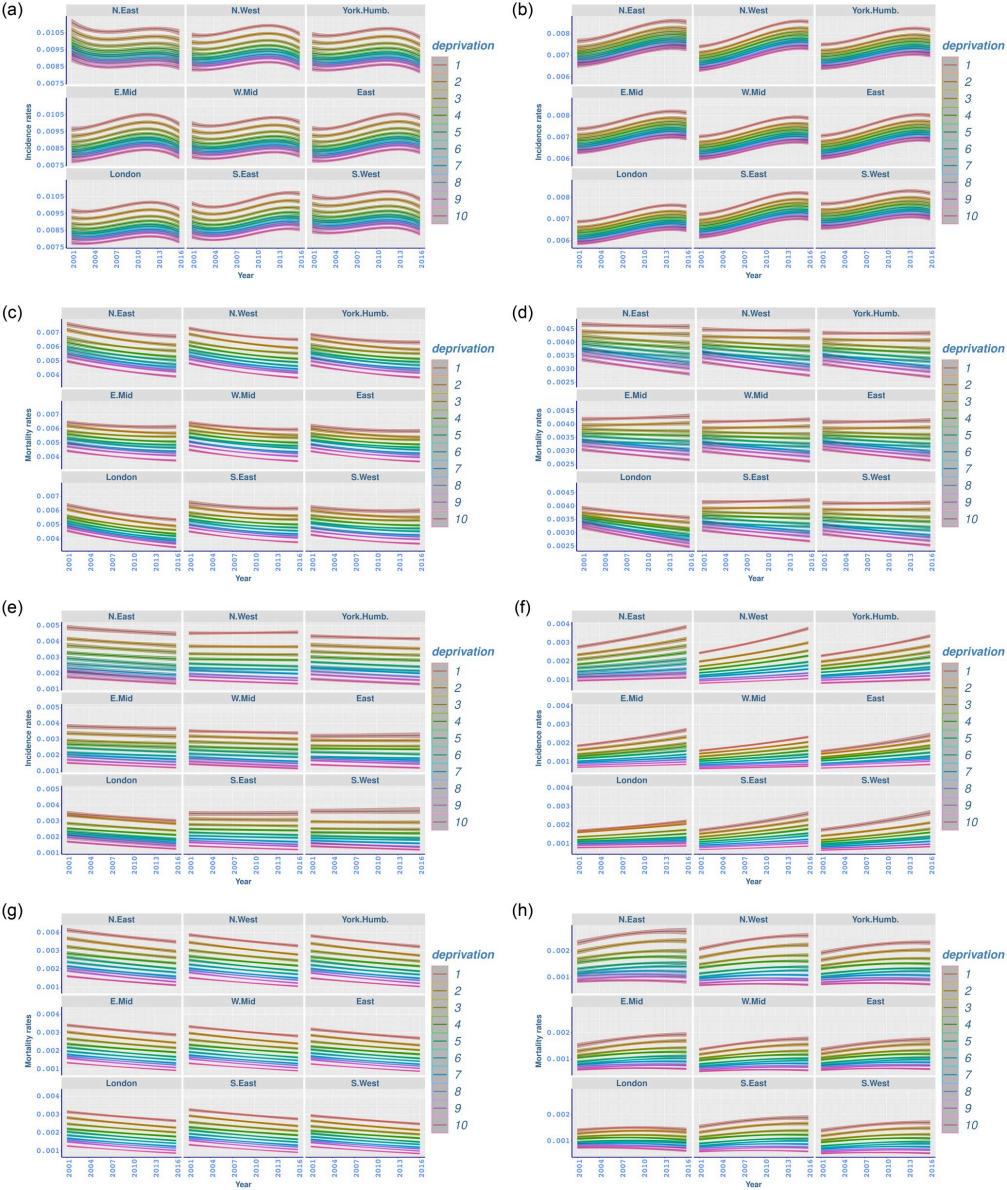
Age-standardised fitted incidence and mortality rates (with 95% credible intervals) for all-cancer, and lung, trachea and bronchus cancer, between 2001 and 2016 for different deprivation deciles and regions in England. (a) Male all-cancer incidence; (b) Female all-cancer incidence; (c) Male all-cancer mortality; (d) Female all-cancer mortality; (e) Male lung cancer incidence; (f) Female lung cancer incidence; (g) Male lung cancer mortality; (h) Female lung cancer mortality.

Fig 1a shows that age-standardised incidence rates for males vary considerably across deprivation deciles and in different regions, with flattened or dropping rates in the most recent years, whilst there is a steeper increasing trend for female counterparts with similar flattened rates in recent years (Fig 1b). For both genders, and in all regions, there are distinct and significant differences between incidence rates in high and low deprivation groups, with the highest incidence rates having been realised in the most deprived population groups (decile 1), and the lowest rates in the least deprived groups (decile 10) (Fig 1a and 1b).

The overall temporal change for different deprivation groups, as presented in Table 4, shows that all-cancer incidence rates for males have significantly increased in certain regions, e.g. the East and the South East, whereas a decrease is observed in some other regions, e.g. the North East. The highest increase has occurred in the most deprived decile in the East (64.52, 49.02 to 80.14, per 100,000 people), whereas a significant decrease is shown in the North East since 2001, with the highest change in the most deprived group (−58.65, −80.37 to −35.95). For females, all-cancer incidence rates have significantly increased in all regions, with the highest change shown for the most deprived decile in the North West (115.66, 104.36 to 126.98) (Table 4). Importantly, although the overall-temporal-changes in all-cancer incidence are mostly significant for both genders, these changes when compared between the most and least deprived deciles for a given region, are not considerably different from each other.

**Table 4:** Overall-temporal-changes (AC_*d*,*r*_, per 100,000 people) from 2001 to 2016, in age-standardised fitted incidence rates for deprivation levels 1 and 10 and all regions in England for both genders; 95% credible intervals in brackets.

Type of cancer	Deprivation decile	N.East	N.West	York.Humb.	E.Mid	W.Mid	East	London	S.East	S.West
**Men**										
**All-cancer**	most (1)	−58.65 (−80.37,−35.95)	7.64 (−7.18,22.3)	−15.86 (−33.52,0.14)	31.41 (15.47,47.9)	9.84 (−5.93,25.48)	64.52 (49.02,80.14)	11.98 (−3.63,27.09)	59.49 (44.81,74.39)	−13.45 (−29.2,1.88)
	least (10)	−49.84 (−67.2,−31.51)	3.49 (−8.44,15.34)	−15.36 (−29.36,−2.45)	22.72 (10.01,35.92)	5.35 (−7.4,17.92)	49.25 (37.15,61.61)	7.15 (−5.38,19.44)	45.12 (33.54,56.78)	−13.46 (−26.2,−1.18)
**Lung cancer**	most (1)	−42.46 (−59.07,−26.11)	4.64 (−8.18,19.75)	−17.18 (−29.71,−2.21)	−15.73 (−28.14,−0.67)	−13.64 (−25.24,−1.33)	4.26 (−7.08,16.24)	−47.51 (−59.3,−34.97)	−0.04 (−12.29,12.12)	2.04 (−11.68,18.26)
	least (10)	−41.51 (−51.14,−33.48)	−24.16 (−29.96,−18.69)	−31.87 (−37.99,−24.2)	−29.52 (−37.59,−22.9)	−27.9 (−32.81,−22.43)	−20.99 (−26.05,−15.61)	−46.96 (−52.67,−40.65)	−23.09 (−28.03,−18.53)	−22.01 (−27.91,−15.36)
**Bowel cancer**	most (1)	−10.81 (−14.51,−7.05)	−10.12 (−13.61,−6.6)	−9.8 (−13.16,−6.37)	−9.64 (−12.94,−6.29)	−9.84 (−13.25,−6.4)	−9.31 (−12.52,−6.06)	−8.49 (−11.4,−5.54)	−9.29 (−12.53,−6.04)	−9.7 (−13.06,−6.29)
	least (10)	−9.63 (−12.93,−6.24)	−9.01 (−12.07,−5.85)	−8.73 (−11.69,−5.68)	−8.58 (−11.5,−5.58)	−8.77 (−11.76,−5.67)	−8.29 (−11.12,−5.37)	−7.56 (−10.11,−4.92)	−8.28 (−11.13,−5.37)	−8.64 (−11.6,−5.59)
**Prostate cancer**	most (1)	−11.73 (−28.76,5.61)	14.97 (0.87,29.92)	17.95 (4.72,31.7)	53.68 (39.04,70.15)	18.79 (−0.72,38.77)	69.78 (54.17,87.43)	84.54 (65.35,105.28)	79.81 (62.38,98.4)	6.31 (−9.75,22.37)
	least (10)	−82.52 (−106.66,−59.27)	−46.02 (−62.32,−31.48)	−41.88 (−59.41,−25.91)	8.07 (−9.42,25.44)	−50.08 (−66.83,−33.09)	15.06 (−2.72,31.39)	18.37 (5.23,33.63)	26.36 (11.72,39.23)	−65.63 (−81.29,−49.47)
**Women**										
**All-cancer**	most (1)	88.96 (71.85,105.73)	115.66 (104.36,126.98)	67.78 (55.59,79.7)	75.43 (62.18,88.87)	85.9 (73.98,97.45)	94.88 (83.08,106.17)	71.41 (60.7,82.49)	96.6 (86.41,107.14)	48.79 (36.38,60.88)
	least (10)	77.13 (62.6,91.28)	99.86 (90.46,109.42)	59.13 (48.69,69.42)	65.58 (54.2,77.09)	74.45 (64.27,84.43)	81.95 (71.93,91.56)	61.82 (52.67,71.3)	83.53 (74.63,92.65)	42.96 (32.38,53.29)
**Lung cancer**	most (1)	108.15 (95.52,119.25)	131.61 (122.56,141.56)	106.14 (97.15,116.34)	85.33 (77.1,94.52)	73.79 (66.98,79.64)	84.48 (76.29,94.79)	52.71 (45.96,60.84)	89.27 (80.32,97.52)	91.58 (81.86,103.53)
	least (10)	16.03 (10.2,21.45)	23.87 (19.78,27.37)	18.89 (14.53,23.67)	15.79 (11.17,19.3)	14.24 (11.3,16.93)	19.37 (15.78,23.4)	8.24 (5.07,11.39)	18.91 (16.07,21.87)	18.62 (15.49,22.39)
**Bowel cancer**	most (1)	1.5 (−0.82,3.89)	1.47 (−0.82,3.84)	1.42 (−0.78,3.71)	1.46 (−0.81,3.79)	1.46 (−0.79,3.81)	1.53 (−0.85,4.01)	1.38 (−0.76,3.59)	1.53 (−0.84,3.96)	1.59 (−0.88,4.14)
	least (10)	1.44 (−0.78,3.73)	1.41 (−0.78,3.66)	1.36 (−0.75,3.54)	1.4 (−0.77,3.63)	1.4 (−0.77,3.63)	1.47 (−0.81,3.81)	1.33 (−0.73,3.45)	1.46 (−0.81,3.8)	1.52 (−0.82,3.94)
**Breast cancer**	most (1)	18.52 (16.84,20.16)	18.88 (17.19,20.54)	18.42 (16.78,20.08)	19.08 (17.37,20.81)	19.06 (17.41,20.76)	18.88 (17.19,20.56)	18.17 (16.57,19.79)	19.24 (17.54,20.98)	19.95 (18.15,21.77)
	least (10)	22.03 (20.06,24)	22.46 (20.46,24.46)	21.9 (19.94,23.86)	22.69 (20.66,24.73)	22.67 (20.67,24.67)	22.45 (20.47,24.49)	21.61 (19.7,23.54)	22.88 (20.86,24.95)	23.73 (21.62,25.84)

Table 5 shows that for all-cancer incidence, the difference between the highest- and lowest-incidence rates among deprivation deciles is significantly high in all regions, with the highest gap in the North East (e.g. 218 (209 to 226) for males in 2001). The table also shows that there is a general increase in absolute differences between 2001 and 2016. The gap in male all-cancer incidence has not significantly changed, with an exception in the East where there is a significant increase (from 189 (182 to 196) in 2001 to 205 (197 to 212) in 2016). However, considerable increases occurred for female all-cancer incidence from 2001 to 2016 in more regions, specifically in the North East, the North West, the West Midlands, the East, and the South East, where the highest increase is shown in the North West (from 109 (104 to 115) to 125 (119 to 131)).

**Table 5:** Absolute deprivation differences (AD_*t*,*r*_), per 100,000 people, in age-standardised fitted incidence rates in 2001 and 2016 for all regions in England and both genders; 95% credible intervals in brackets.

Type of cancer	Year	N.East	N.West	York.Humb.	E.Mid	W.Mid	East	London	S.East	S.West
**Men**										
**All-cancer**	2001	218 (209,226)	203 (195,210)	203 (196,211)	189 (181,195)	195 (188,202)	189 (182,196)	189 (182,196)	197 (190,204)	205 (198,213)
	2016	209 (202,217)	207 (200,214)	203 (196,210)	197 (190,204)	200 (192,206)	205 (197,212)	194 (187,201)	212 (204,219)	205 (198,213)
**Lung cancer**	2001	310 (297,323)	293 (282,303)	269 (256,282)	230 (215,244)	207 (198,217)	178 (163,193)	177 (163,191)	203 (189,217)	218 (205,233)
	2016	309 (297,323)	322 (312,332)	284 (273,295)	244 (232,255)	222 (212,234)	204 (190,221)	177 (167,187)	226 (210,241)	243 (226,263)
**Bowel cancer**	2001	27 (23,31)	25 (22,29)	25 (21,28)	24 (21,28)	25 (21,28)	23 (20,27)	21 (18,25)	23 (20,27)	24 (21,28)
	2016	26 (22,30)	24 (21,28)	23 (20,27)	23 (20,27)	24 (20,27)	22 (19,26)	20 (17,23)	22 (19,26)	23 (20,27)
**Prostate cancer**	2001	134 (111,162)	127 (112,143)	159 (141,176)	107 (92,125)	136 (118,156)	111 (93,129)	40 (28,53)	112 (97,130)	165 (139,190)
	2016	69 (54,88)	67 (56,82)	101 (86,117)	71 (57,88)	71 (57,86)	68 (51,87)	53 (39,72)	65 (48,83)	95 (72,117)
**Women**										
**All-cancer**	2001	113 (108,118)	109 (104,115)	110 (105,115)	109 (104,114)	104 (99,109)	105 (100,110)	102 (97,107)	107 (101,112)	113 (108,118)
	2016	125 (119,131)	125 (119,131)	119 (113,124)	119 (113,124)	115 (110,120)	118 (112,123)	111 (106,117)	120 (114,125)	119 (113,124)
**Lung cancer**	2001	180 (172,188)	162 (156,167)	146 (139,153)	115 (108,121)	96 (92,101)	90 (82,97)	89 (83,95)	103 (96,110)	108 (100,115)
	2016	272 (261,282)	270 (261,279)	233 (225,243)	185 (175,193)	156 (149,163)	155 (144,168)	133 (127,141)	173 (161,185)	181 (167,194)
**Bowel cancer**	2001	6 (4,8)	6 (4,8)	6 (4,8)	6 (4,8)	6 (4,8)	6 (4,9)	6 (4,8)	6 (4,9)	6 (4,9)
	2016	6 (4,9)	6 (4,8)	6 (4,8)	6 (4,8)	6 (4,8)	6 (4,9)	6 (4,8)	6 (4,9)	6 (4,9)
**Breast cancer**	2001	34 (32,37)	35 (32,38)	34 (31,37)	36 (33,38)	35 (33,38)	35 (32,38)	34 (31,36)	36 (33,39)	37 (34,40)
	2016	38 (35,41)	39 (36,42)	38 (35,41)	39 (36,42)	39 (36,42)	39 (36,42)	37 (34,40)	39 (36,42)	41 (38,44)

All-cancer mortality has declined since 2001, with the lowest rates and steeper improvements, for both men and women, in more affluent groups (Fig 1c and 1d). Table 6 highlights that males have experienced high all-cancer mortality improvements in all deprivation deciles and regions. However, the mortality improvement in female all-cancer mortality is less manifested and not significant for the most deprived decile of the population in most regions. The highest mortality improvement is shown in the least deprived group in London, particularly −117.14 (−126.98 to −106.32) for males, and −72.83 (−82.02 to −62.98) for females (Table 6). We can also note a marginally significant increase in female all-cancer mortality for the most deprived population groups in the East Midlands and the West Midlands. Moreover, Table 6 indicates that, for females, the overall-temporal-changes are distinctively different between the most and least deprived deciles in all regions. Yet this is not always the case for males.

**Table 6:** Overall-temporal-changes (AC_*d*,*r*_, per 100,000 people) from 2001 to 2016, in age-standardised fitted mortality rates for deprivation levels 1 and 10 and all regions in England for both genders; 95% credible intervals in brackets.

Type of cancer	Deprivation decile	N.East	N.West	York.Humb.	E.Mid	W.Mid	East	London	S.East	S.West
**Men**										
**All-cancer**	most (1)	−87.11 (−105.79,−64.64)	−78.82 (−94.4,−61.85)	−54.73 (−71.98,−38.96)	−29.16 (−46.16,−11.08)	−46.43 (−64.81,−30.34)	−35.7 (−52.32,−20.39)	−101.66 (−118.91,−85.44)	−40.88 (−59.05,−27.67)	−24.14 (−40.71,−7.25)
	least (10)	−107.82 (−122.65,−93.98)	−101.66 (−111.76,−92.08)	−87.38 (−98.37,−76.64)	−68.91 (−81.13,−57.6)	−80.88 (−91.79,−68.46)	−73.18 (−82.81,−64.55)	−117.14 (−126.98,−106.32)	−77.14 (−86.77,−66.96)	−64.14 (−75.06,−52.45)
**Lung cancer**	most (1)	−64.51 (−77.41,−50.41)	−60.48 (−72.27,−47.29)	−59.57 (−71.34,−46.75)	−53.22 (−63.53,−41.67)	−52.04 (−62.22,−40.59)	−49.73 (−59.63,−38.82)	−49.12 (−58.86,−38.39)	−50.96 (−60.98,−39.78)	−45.95 (−54.94,−35.82)
	least (10)	−49.35 (−56.59,−42.91)	−46.84 (−53.54,−40.76)	−46.13 (−52.82,−40.05)	−41.46 (−47.28,−36.03)	−40.49 (−46.29,−35.18)	−39.34 (−44.95,−34.33)	−39.22 (−44.7,−34.19)	−41.31 (−47.44,−35.87)	−38.26 (−43.67,−33.18)
**Bowel cancer**	most (1)	−12.27 (−13.33,−11.2)	−11.77 (−12.73,−10.8)	−11.4 (−12.43,−10.42)	−11.37 (−12.36,−10.41)	−11.64 (−12.6,−10.68)	−10.92 (−11.84,−10)	−9.39 (−10.27,−8.54)	−10.8 (−11.72,−9.86)	−10.74 (−11.67,−9.81)
	least (10)	−10.19 (−11.08,−9.28)	−9.78 (−10.58,−8.96)	−9.46 (−10.31,−8.64)	−9.39 (−10.21,−8.55)	−9.69 (−10.47,−8.86)	−9.04 (−9.8,−8.29)	−7.73 (−8.46,−6.99)	−8.92 (−9.67,−8.12)	−8.88 (−9.67,−8.07)
**Prostate cancer**		−20.15 (−23.16,−17.13)	−20.2 (−23.22,−17.17)	−19.98 (−22.96,−17)	−20.49 (−23.57,−17.42)	−19.83 (−22.79,−16.87)	−20.05 (−23.05,−17.05)	−19.19 (−22.09,−16.3)	−19.7 (−22.65,−16.74)	−20.39 (−23.44,−17.31)
**Women**										
**All-cancer**	most (1)	−8.06 (−23.25,5.94)	−4.47 (−16.62,7.32)	−0.91 (−13.9,11.42)	11.06 (−2.05,24.82)	9.19 (−3.03,21.18)	5.83 (−6.68,19.22)	−36.94 (−48.58,−24.9)	7.59 (−4.47,20.88)	2.04 (−10.16,14.89)
	least (10)	−54.35 (−64.43,−42.56)	−50.82 (−58.87,−40.77)	−47.43 (−57.39,−37.72)	−37.97 (−46.04,−28.01)	−38.29 (−47.6,−28.45)	−41.25 (−49.82,−32.64)	−72.83 (−82.02,−62.98)	−40.73 (−48.73,−31.59)	−42.26 (−50.64,−33.78)
**Lung cancer**	most (1)	43.57 (27.04,58.82)	51.14 (41.15,62.07)	39.59 (28.44,49.68)	41.53 (30.96,54.14)	40.44 (31.39,49.91)	38.69 (29.31,48.31)	2.3 (−5.66,11.46)	34.59 (25.21,44.42)	30.96 (19.76,40.96)
	least (10)	−1.78 (−8.5,4.66)	1.73 (−2.27,7.02)	−0.54 (−5.33,5.35)	2.84 (−1.62,7.12)	3.6 (−0.84,8.4)	3.49 (−0.96,8.13)	−11.93 (−16.3,−6.36)	0.73 (−2.88,4.79)	0.67 (−3.84,5.08)
**Bowel cancer**	most (1)	−5.2 (−5.97,−4.49)	−5.17 (−5.92,−4.45)	−5.2 (−5.96,−4.47)	−5.18 (−5.94,−4.47)	−5.18 (−5.93,−4.46)	−5.25 (−6.02,−4.53)	−5.28 (−6.06,−4.55)	−5.24 (−6.02,−4.52)	−5.25 (−6.01,−4.52)
	least (10)	−4.54 (−5.21,−3.89)	−4.51 (−5.18,−3.86)	−4.53 (−5.2,−3.88)	−4.52 (−5.19,−3.87)	−4.52 (−5.19,−3.86)	−4.58 (−5.26,−3.92)	−4.6 (−5.29,−3.94)	−4.57 (−5.25,−3.91)	−4.57 (−5.25,−3.92)
**Breast cancer**		−19.4 (−20.68,−18.07)	−19.86 (−21.14,−18.57)	−19.44 (−20.71,−18.17)	−20.67 (−22.04,−19.28)	−20.54 (−21.87,−19.21)	−20.8 (−22.14,−19.45)	−20.02 (−21.3,−18.68)	−20.58 (−21.92,−19.23)	−19.92 (−21.24,−18.6)

Similarly to incidence, Table 7 demonstrates that all-cancer mortality differences between the highest and lowest rates among deprivation deciles are significantly high in all regions, with the highest gap again in the North East (e.g. 262 (249 to 275) for males in 2001).

**Table 7:** Absolute deprivation differences (AD_*t*,*r*_), per 100,000 people, in age-standardised fitted mortality rates in 2001 and 2016 for all regions in England and both genders; 95% credible intervals in brackets.

Type of cancer	Year	N.East	N.West	York.Humb.	E.Mid	W.Mid	East	London	S.East	S.West	
**Men**											
**All-cancer**	2001	262 (249,275)	247 (236,259)	215 (202,228)	199 (186,212)	189 (179,201)	178 (163,192)	180 (168,193)	202 (187,216)	194 (181,206)	
	2016	283 (269,296)	270 (259,281)	248 (235,261)	239 (224,252)	224 (212,234)	216 (203,229)	196 (184,205)	238 (227,252)	234 (219,247)	
**Lung cancer**	2001	253 (243,263)	235 (226,244)	232 (223,240)	206 (198,214)	202 (194,209)	191 (183,199)	187 (179,195)	192 (184,200)	170 (162,177)	
	2016	238 (230,246)	222 (214,230)	218 (211,227)	194 (187,202)	190 (183,197)	180 (174,188)	177 (171,184)	183 (176,190)	162 (156,169)	
**Bowel cancer**	2001	13 (12,14)	13 (11,14)	12 (11,13)	12 (11,13)	12 (11,14)	12 (11,13)	11 (10,12)	12 (11,13)	12 (11,13)	
	2016	11 (10,12)	11 (10,12)	10 (9,11)	10 (9,11)	10 (9,11)	10 (9,11)	9 (8,10)	10 (9,11)	10 (9,11)	
**Prostate cancer**	2001	−	−	−	−	−	−	−	−	−	14 (11,17)
	2016	−	−	−	−	−	−	−	−	−	13 (10,16)
**Women**											
**All-cancer**	2001	131 (123,139)	121 (114,129)	116 (109,124)	114 (107,121)	109 (103,116)	102 (95,110)	73 (66,80)	105 (98,112)	115 (108,122)	
	2016	177 (170,185)	168 (161,176)	163 (156,170)	163 (155,171)	157 (150,164)	149 (142,157)	108 (102,115)	154 (146,161)	159 (153,167)	
**Lung cancer**	2001	146 (137,156)	138 (130,145)	119 (111,127)	92 (83,100)	83 (77,88)	77 (72,85)	66 (60,73)	94 (88,100)	86 (80,93)	
	2016	192 (180,205)	187 (178,196)	159 (149,167)	130 (123,139)	119 (113,127)	112 (105,121)	80 (74,88)	127 (119,137)	117 (109,124)	
**Bowel cancer**	2001	5 (5,6)	5 (5,6)	5 (5,6)	5 (5,6)	5 (5,6)	5 (5,6)	5 (5,6)	5 (5,6)	5 (5,6)	
	2016	5 (4,5)	5 (4,5)	5 (4,5)	5 (4,5)	5 (4,5)	5 (4,5)	5 (4,5)	5 (4,5)	5 (4,5)	
**Breast cancer**	2001	−	−	−	−	−	−	−	−	−	6 (5,8)
	2016	−	−	−	−	−	−	−	−	−	5 (4,6)

As also shown in Table 7, there is an increasing trend in deprivation gap between 2001 and 2016 for all-cancer mortality in both men and women, with the increase in absolute differences from 2001 to 2016 being significant in almost all regions. The highest change in absolute differences occurred in the East Midlands for both genders, from 199 (186 to 212) to 239 (224 to 252) for males, and from 114 (107 to 121) to 163 (155 to 171) for females. Note that the East Midlands is followed by the South West and the South East with similar changes in absolute differences for males and females, respectively.

Relative differences between deprivation deciles for all-cancer mortality have significantly increased from 2001 to 2016 with the highest change in London for both genders. This is important as there were no considerable changes, in relative terms, for the corresponding morbidity, and suggesting all-cancer morbidity and mortality are increasing at different rates (see S1 File for details). This was not the case in type-specific cancers apart from lung cancer where relative differences for both incidence and mortality in men and women have significantly increased.

### Lung cancer

Socioeconomic and regional disparities in lung cancer differed for men and women. Although men and women in more deprived deciles have higher lung cancer incidence, we observe reversed trends for men and women (Table 4). Incidence rates of lung cancer for females point out a rapidly increasing trend, especially for more deprived groups in each region, with a widening gap over years, particularly in the north of England (Fig 1e and 1f and Table 5). This is consistent with similar findings in the literature [20, 48].

Table 4 shows that lung cancer incidence for men declined in all regions between 2001 and 2016, with significantly higher decreases in the least deprived group in each region. The highest reduction in lung cancer incidence occurred in London, with comparable rates between socioeconomic groups. For females, lung cancer incidence has significantly increased in all regions. However, the increase is distinctively higher for the most deprived decile of the population, with the North East showing an increase of 108.15(95.52 to 119.25) for the most deprived group, while the increase was 16.03(10.2 to 21.45) in the least deprived group.

Absolute differences between the highest and lowest lung cancer incidence rates among deprivation groups are significantly high for both men and women in all regions, with the widest gap in the North East and the North West (Table 5). The deprivation gap for men has increased significantly in the North West from 293 (282 to 303) in 2001, to 322 (312 to 332) in 2016, yet the differences remain similar in other regions. In contrast, the deprivation gap in female lung cancer incidence has considerably risen in each region, with the highest change in the North West, from 162 (156 to 167) in 2001 to 270 (261 to 279) in 2016, followed by the North East (from 180 (172 to 188) to 272 (261 to 282)), and Yorkshire and the Humber (from 146 (139 to 153) to 233 (225 to 243)) (Table 5).

Lung cancer mortality for men has improved, whilst it has mostly deteriorated for women, with bigger disparities in more deprived levels (Fig 1g and 1h). This effect is more prominent in northern regions of England. Once again, the analysis suggests distinct differences among deprivation deciles in addition to a clear widening gap between the highest and lowest mortality rates for females, where the highest rates occurred in the most deprived deciles in each region. This is consistent with similar findings in the literature [16].

The temporal changes reported in Table 6 reveal mortality improvement in male lung cancer in all regions, with the highest improvements in the north of England. Although females suffer an increase in lung-cancer mortality rates, a significant improvement has been observed for the most affluent group (decile 10) in London (−11.93, −16.3 to −6.36). The highest increase in female lung cancer mortality happened in the most deprived decile of the North West with 51.14 (41.15 to 62.07), followed again by the most deprived group in the North East (43.57, 27.04 to 58.82) and the East Midlands (41.53, 30.96 to 54.14). Overall-temporal-changes are not significantly different between the most and least deprived deciles for male lung cancer mortality. On the other hand, the changes in female lung cancer mortality are not comparable between the most and least deprived groups, with the most deprived decile of the population experiencing considerably higher increases in mortality (Table 6).

Table 7 shows significant lung cancer mortality differences between deprivation groups, both for men and women in all regions. For men, although these differences have moderately decreased between 2001 and 2016, the changes are not significant. On the contrary, the deprivation gap for female lung cancer mortality has considerably increased, with the highest change in the North West (from 138 (130 to 145) in 2001 to 187 (178 to 196) in 2016). This is followed by the North East (from 146 (137 to 156) to 192 (180 to 205)), and Yorkshire and the Humber (from 119 (111 to 127) to 159 (149 to 167)) (Table 7).

### Bowel cancer

**Fig 2:**
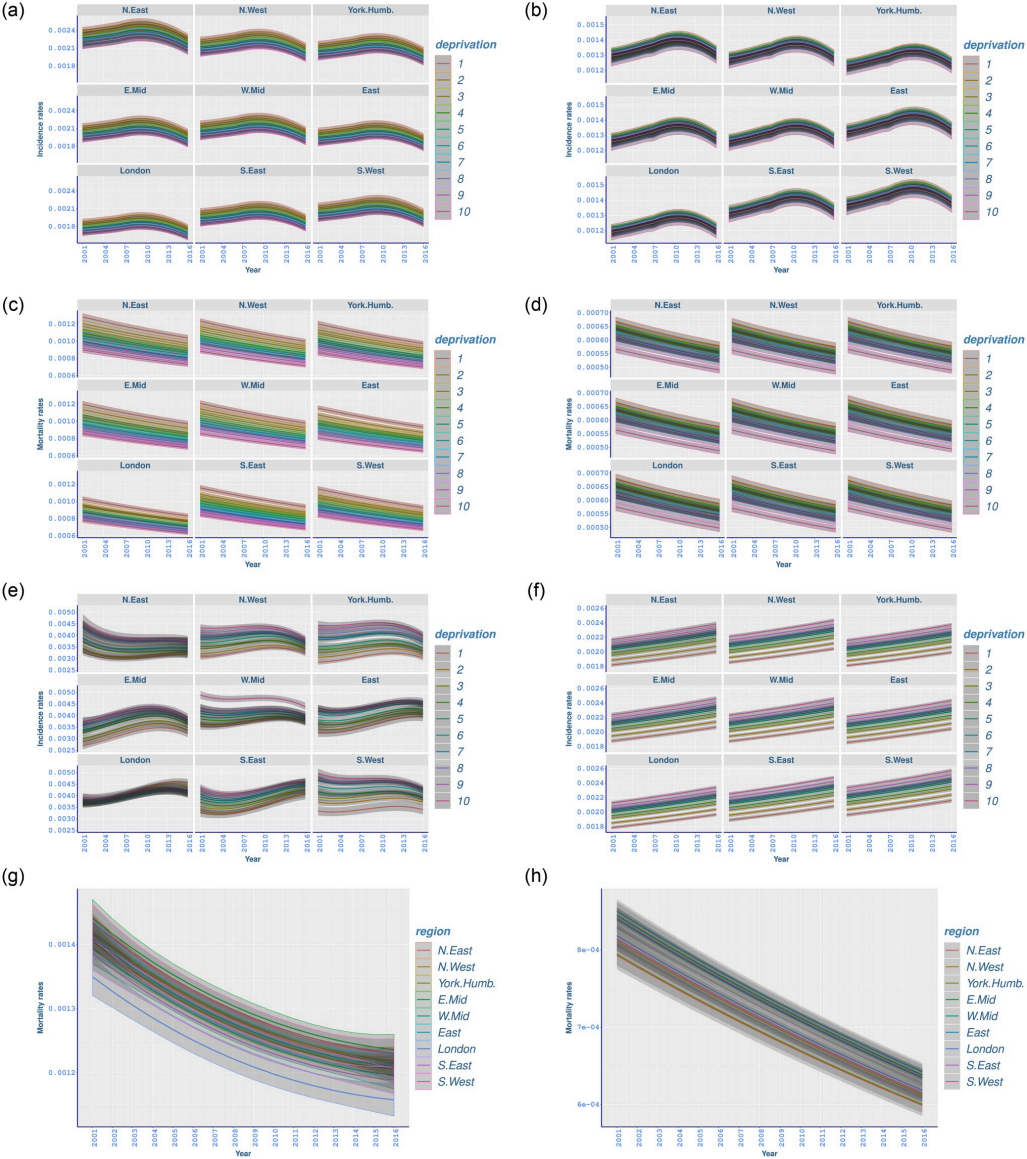
Age-standardised fitted incidence and mortality rates (with 95% credible intervals) for bowel, prostate, and breast cancer, between 2001 and 2016 for different deprivation deciles and regions in England. (a) Male bowel cancer incidence; (b) Female bowel cancer incidence; (c) Male bowel cancer mortality; (d) Female bowel cancer mortality; (e) Prostate cancer incidence; (f) Breast cancer incidence; (g) Prostate cancer mortality; (h) Breast cancer mortality.

Age-standardised fitted incidence rates of bowel cancer point towards lower regional and socioeconomic differences compared to all-cancer and lung cancer incidence, with higher rates for more deprived groups. An increasing trend up to around 2010 is followed by a decreasing trend for both males and females (Fig 2a and 2b). The decreasing trend in more recent years may be related to the uptake of English Bowel Cancer Screening, which has dropped from 54% (2006–2009) to 49% (2010–2015) [49].

The overall-temporal-changes from 2001 to 2016, as presented in Table 4, show significant reductions for males and marginal increases for females. Particularly, the biggest reduction in male bowel cancer incidence occurred for the most deprived decile in the North East (−10.81, −14.51to−7.05), whilst the biggest increase for females was shown in the same decile in the South West (1.59, −0.88to4.14) (Table 4). The marginal increase in female incidence can potentially be linked to the uptake of the English Bowel Screening programme, where the uptake for women is noted to be higher between 2010 and 2015 (47% for men vs 56% for women) [49].

Findings reported in Table 5 demonstrate that absolute differences for bowel cancer rates between the highest and lowest rates among deprivation groups for males are significant and show a marginal decreasing over time. For females, these differences are smaller and mostly remained unchanged between 2001 and 2016.

Similar to all-cancer and lung cancer mortality rates, bowel cancer mortality has declined in all deprivation deciles for both genders since 2001, although people in more deprived deciles experienced higher mortality rates (Fig 2c and 2d).

Table 6 points out that men in the most deprived group in the North East benefited from the highest mortality improvement (−12.27, −13.33 to −11.2), followed by comparable changes in other regions. Yet, the comparison of overall-temporal-changes between the most and least deprived deciles in each region shows significant differences; e.g. in East, with −10.92 (−11.84 to −10.00) for decile 1, and −9.04 (−9.8 to −8.29) for decile 10 (Table 6). Likewise, women in the most deprived deciles experienced higher mortality improvement compared to those in more affluent groups.

Low socioeconomic variation in bowel cancer mortality has been manifested by the absolute differences findings in Table 7, for both men and women. Also, the deprivation gap between the most and least deprived deciles has been marginally narrowing for males between 2001 and 2016, while remaining unchanged for females (Table 7, Fig 2a and 2b).

### Prostate cancer

The overall trend in prostate cancer incidence shows a distinct regional variation and a narrowing deprivation gap between 2001 and 2016 (Fig 2e). Unlike all-cancer, lung, and bowel cancer incidence, men in less deprived deciles of the population have experienced prostate cancer in higher rates. This is consistent with similar findings in the literature [20].

However, different than in other regions, the highest prostate cancer incidence in London occurred in more deprived group, with a widening gap since 2001 (Tables 4 and 5). Moreover, prostate cancer incidence has varied from north of England to south of England in a particular way: while it has mostly increased in the south, it generally decreased in the north.

Table 4 shows that the highest temporal increase occurred in the most deprived decile in London (84.54, 65.35 to 105.28) and the South East (79.81, 62.38 to 98.4), while there was a decrease in the least deprived decile in the North East (−82.52, −106.66 to −59.27). The South West stands as an exception where the incidence rates have mostly declined, with the highest variation in the most affluent group (−65.63, −81.29 to −49.47). Hereby, incidence rates for men living in the northern regions seem to show bigger changes in less deprived deciles, whereas the incidence for men living in the southern regions has shown higher changes in more deprived deciles.

As in other cancer types, there is a significant deprivation gap for prostate cancer incidence, as shown by the absolute deprivation differences in Table 5. However, this gap has declined between 2001 and 2016, with the biggest reduction in the South West, from 165 (139 to 190) to 95 (72 to 117), followed by comparable changes in the North East and the West Midlands. The only exception has occurred in London, where a marginal increase is observed (Table 5).

There is a decreasing trend in prostate cancer mortality rates, where the improvement in prostate cancer mortality has slowed down in most recent years (Fig 2g). In contrary to the other cancers we investigated, deprivation is not found as a significant factor to explain changes in prostate cancer mortality rates. The highest improvement in prostate cancer mortality occurred in the East Midlands (−20.49, −23.57 to −17.42), with comparable changes in other regions (Table 6). The gap between the highest and lowest mortality rates among regions of England has been declining with comparable changes over the years.

### Breast cancer

Fig 2f exhibits that breast cancer incidence has increased in all regions with higher rates in more affluent population deciles.

Significant increases in breast cancer incidence have occurred for all deprivation levels and regions (Table 4). Only marginally significant increases are noted in the deprivation gap in each region for breast cancer incidence (Table 5).

Fig 2h displays a decreasing trend in breast cancer mortality rates over time, with deprivation having no significant impact on this cancer type—as was the case with prostate cancer mortality. The highest decline between 2001 and 2016 occurred in the East Midlands (−20.67, −22.04 to −19.28), with similar changes in other regions (Table 6). The gap between the highest and lowest mortality rates among regions of England has marginally declined since 2001 (Table 7).

### Impact of age-at-diagnosis on cancer mortality

We quantify the impact of one-year, 6-month and 3-month delay in cancer diagnosis on cancer mortality rates. This is achieved by including age-at-diagnosis (as obtained in the section, namely Statistical Modelling), as an explanatory factor in the model for estimating mortality (see Table 3). The effect of the average age-at-diagnosis coefficient estimate is then considered, distinguished by region where appropriate (e.g. for female lung cancer mortality). Our analysis found that the impact of age-at-diagnosis on mortality did not vary by deprivation decile, as the interaction between age-at-diagnosis and deprivation is not found significant in the mortality models.

**Fig 3:**
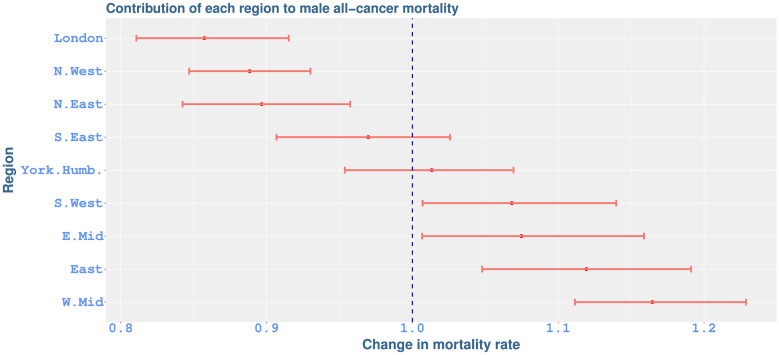
Contribution of each region to all-cancer mortality rates with respect to average age-at-diagnosis (dots), with 95% credible intervals (bars). The vertical line indicates the average regional impact.

Fig 3 shows the contribution of each region to all-cancer mortality with respect to average age-at-diagnosis. The values on the horizontal axis quantify the additional (or lower) impact of age-at-diagnosis on mortality in each region, as compared to the overall average across regions (indicated by value 1). For example, a 1.1 value in the figure points out a region where age-at-diagnosis has a 10% increased impact. The effects of the South East and Yorkshire and Humber are not significantly different from the average. London exhibits a significantly lower effect (−14%, −18.92 to −8.48), with comparable rates in the North West and the North East. The West Midlands has the highest additional effect (16.45%, 11.14 to 22.88), followed by the East, the East Midlands, and the South West. Note that there are no significant differences in female all-cancer mortality among regions of England with respect to average age-at-diagnosis.

**Fig 4:**
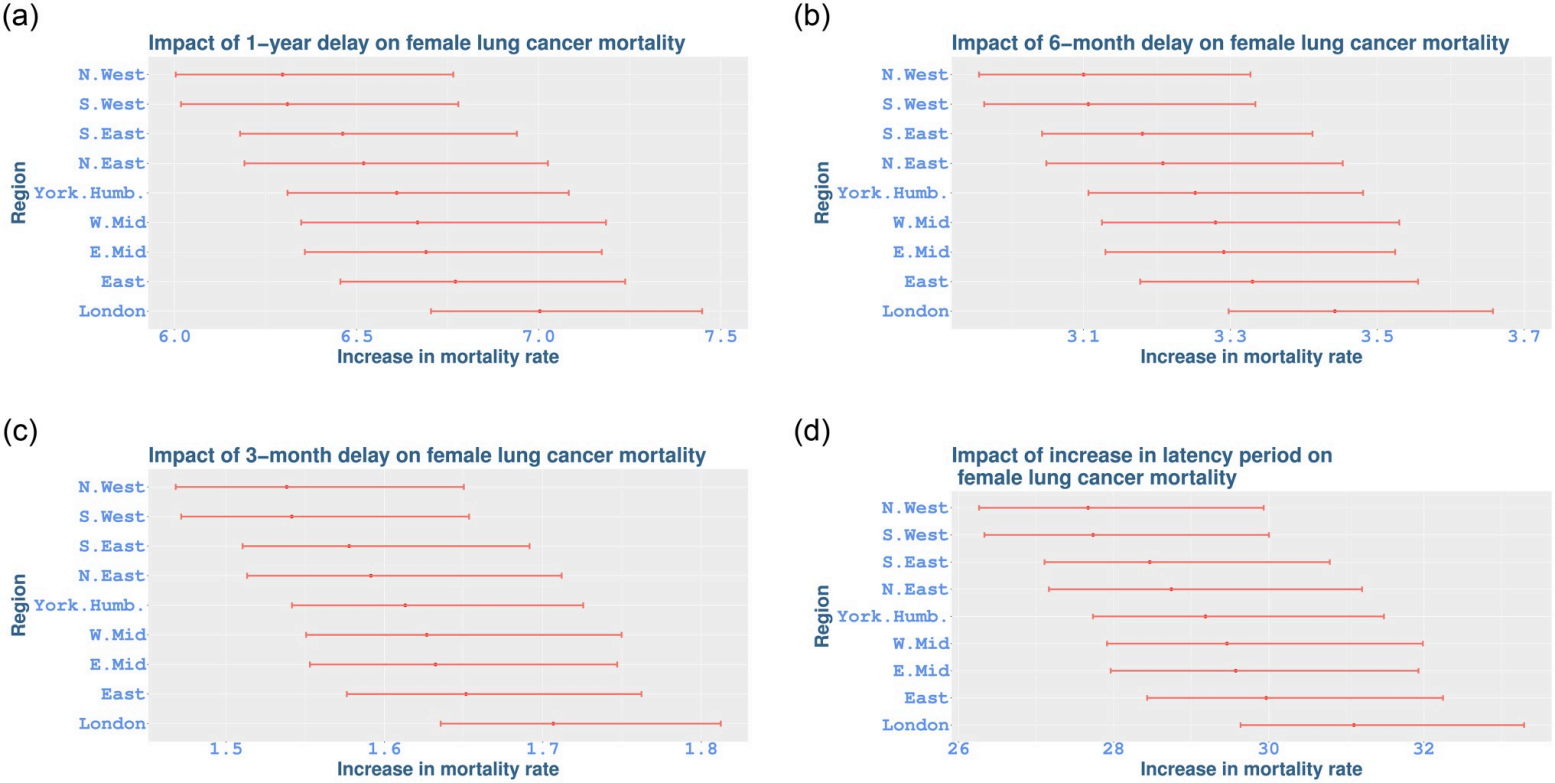
The impact of delay in average age-at-diagnosis for female mortality rates of trachea, bronchus and lung cancer in different regions of England. The numbers represent percentage increase in mortality rates (means shown with dots, 95% credible intervals with bars). (a) 1-year impact; (b) 6-month impact; (c) 3-month impact; (d) 4-year increase in latency period.

For males, the impact of one-year and 6-month delay in lung cancer diagnosis on lung cancer mortality rates would be an increase of 3.39% (3.29 to 3.48) and 1.68% (1.631.73) respectively. A 3-month delay is associated with a smaller (less than 1%) but significant increase in mortality. Our analysis suggests that the impact does not vary by region for males. Fig 4 shows the impact of such delays by region for females, and illustrates both regional variation and a higher increase in female lung cancer mortality in regions of England under similar scenarios. The figure indicates that a 3-month delay in lung cancer diagnosis would cause an approximately 2% increase in death rates, whilst a one-year delay would mean that lung cancer mortality could increase by around 7%. Also, the highest increase is shown in London whereas the lowest increase occurs in the North West with significant differences between them.

For bowel cancer mortality, our model shows that the impact of average age-at-diagnosis does not vary among regions. The impact of one-year, 6-month and 3-month delay in bowel cancer diagnosis on male bowel cancer mortality rates would be an increase of 23.56% (23.09 to 24.30), 11.16% (10.95 to 11.49), and 5.43% (5.33 to 5.59), respectively across all regions. Similar delays would cause a lower increase in bowel cancer mortality for women (less than 0.4%) and in prostate cancer mortality (less than 0.43%). The impact of the same delay in breast cancer diagnosis shows high variability and is not significant. Our analysis did not find a regional age-at-diagnosis effect for either prostate cancer or breast cancer.

We note here that in the aforementioned results, we use an increase in age-at-diagnosis as a proxy for delays in cancer diagnosis, due for example to delays in medical care. Under our modelling, alternative scenarios can be considered, relating to long-term changes linked to increased latency in cancer registrations, from initiation of the disease to medical diagnosis. In such cases, as shown in Fig 4d, our model suggests that an increase around a quarter of the latency period for lung cancer in women [50], over the entire period covered in our study, i.e. a 3-month increase per year, giving an aggregated increase of 4 years over 2001–2016, would result in an increase around 30% increase in lung cancer mortality rates across different regions of England.

## Discussion

The research in this paper builds on earlier work by the authors on regional variation in cancer morbidity, and goes beyond previously published research [19] by investigating socioeconomic disparities for cancer, by associating cancer incidence and mortality, and by specifying the potential impact of delays in age-at-diagnosis on cancer mortality rates. Specifically, based on comprehensive statistical modelling that also accounts for uncertainty, our analysis reveals socioeconomic differences by region in cancer morbidity and mortality, more specifically in all-cancer in addition to cancers linked to lifestyle factors, such as lung and bowel cancers. Mortality improvements in these cancer types are less manifested for females. The socioeconomic differences by region remain for prostate and breast cancer morbidity whereas only regional differences are identified for the relevant cancer mortality.

Furthermore, we quantify the impact of delay in cancer diagnosis on cancer mortality rates. Hereby, we observe significant increases in lung cancer and prostate cancer mortality rates as a result of increases in average age-at-diagnosis, whereas increases in breast cancer mortality are highly uncertain. Importantly, these findings can be associated with significant concerns about the effect of the COVID-19 pandemic outbreak on cancer patients that may have been faced with suspension of relevant screening or routine diagnostic investigations [51, 52].

The analysis also shows that in most cases there is a significant deprivation gap, with the most deprived groups of the population having generally higher cancer morbidity and mortality rates as compared to the least deprived. Moreover, there are also significant changes from 2001 to 2016 in the deprivation gap for all-cancer and female lung cancer morbidity and mortality, in addition to prostate cancer morbidity in regions of England. For other cancer types there are only marginally significant changes. Specifically, a significant increase has been identified in the deprivation gap for all-cancer, except from male all-cancer incidence where a significant increase for male morbidity only occurred in the East. The deprivation gap for female lung cancer has considerably widened between 2001 and 2016, with the highest change in the North West. A marginally significant decrease has occurred in the deprivation gap for male bowel cancer, whereas the gap for female bowel cancer remained unchanged. Additionally, the deprivation gap in prostate cancer incidence has declined in all regions, yet it has marginally widened in London. The deprivation gap for breast cancer incidence has only increased marginally. Prostate and breast cancer mortality indicate marginally important changes among regions of England.

### Strengths and limitations of this study

This study provides comprehensive population-based estimates of all- and type-specific cancer incidence and mortality by age, year, gender, region, and deprivation index in England for the period 2001 to 2016. We show that average age-at-diagnosis is an important variable to explain cancer mortality, and we also quantify the impact of delays in cancer diagnosis on cancer mortality. However, in this research we have not considered explicitly the impact of diagnosis delays in a certain type of cancer on mortality linked to other types of cancer or all-cancer mortality.

In this study we only focus on the most common type-specific cancers, while other cancer types (e.g. cervical cancer) that are also important in terms of public health and treatment interventions have not been explored. Also, the results could potentially be extended if additional, clinical data were available at diagnosis, such as biomarkers linked to treatment and survival.

## Conclusions

A number of cancer reforms, e.g. the NHS Cancer Plan [53] and the Cancer Reform Strategy [54], and initiatives, e.g. the National Awareness and Early Diagnosis Initiative [55], have been introduced after the Calman-Hine report in 1995 [56] in order to cope with inequalities in access to cancer services, to increase cancer awareness, to diagnose cancer earlier and so on. Yet again not all socioeconomic groups can benefit equally, and deprivation differences are indisputable especially among cancers linked to lifestyle factors [21].

Exploring differences among population groups can show the impact of previous reforms and initiatives on cancer mortality and incidence by also informing future policy makers about the success of earlier methods, e.g. in line with Marmot review into health inequalities in England [57, 58]. Comparison of cancer incidence and mortality across different socioeconomic groups can also reveal valuable insights by potentially addressing the need for region-based public health interventions—for example, smoking cessation, physical activity campaigns, cancer awareness and screening programmes [59, 60]—aiming to earlier detection and reduction of cancer morbidity. Hereby, widening gaps in all-cancer and female lung cancer, or regional variations in the impact of age-at-diagnosis on cancer mortality can potentially be addressed by targeting region-based public health interventions. In this way, for instance, women living in low-income groups in north of England can have wider access to better health services and high quality treatment.

Also, our research shows that London stands as an exception in some cases, e.g. in all-cancer mortality, lung cancer mortality and prostate cancer incidence, where the changes over years are distinctively different as compared to other regions. This might be related to various factors. First, London is the most ethnically diverse region across the English regions, including African (7%), Indian (6.6%), Caribbean (4.2%), any other white (12.6%), and White-British (44.9%) in 2011 [61]. Second, in addition to healthy migrant effect and differential selective migration within England, higher health investment in London is also included in possible explanations [22].

## Supporting information

S1 File This consists of all the supporting tables/figures for this paper. (PDF)
