# Supplementary material for: Socioeconomic disparities in cancer incidence and mortality in England and the impact of age-at-diagnosis on cancer mortality
Source: PLoS One. 2021 Jul 14;16(7):e0253854. doi: 10.1371/journal.pone.0253854 (PMC8279298; doi:10.1371/journal.pone.0253854)
Supplement: S1 File — (PDF) [file pone.0253854.s001.pdf]

Supporting Information

Socioeconomic disparities in cancer incidence and mortality in England and the impact of age-at-diagnosis on cancer mortality

Ayşe Arık, Erengul Dodd, Andrew Cairns, George Streftaris

Contents

1 **Best fitted models for cancer morbidity and mortality** **13**

2 **Relative differences** **15**

3 **Average age-at-diagnosis** **16**

List of Tables

1 **S1 Table. Estimated coefficients for the best fitting model for male all-cancer morbidity.** 18
2 **S2 Table. Estimated coefficients for the best fitting model for female all-cancer morbidity.** 20
3 **S3 Table. Estimated coefficients for the best fitting model for male all-cancer mortality.** 23
4 **S4 Table. Estimated coefficients for the best fitting model for female all-cancer mortality.** 25
5 **S5 Table. Absolute deprivation differences ($\mathbf{AD}_{t,r}$), per 100,000 people, in age-standardised fitted incidence rates of all-cancer from 2001 to 2016 for all regions in England for males; 95% credible intervals in brackets** . 41
6 **S6 Table. Relative deprivation differences ($\mathbf{RD}_{t,r}$) in age-standardised fitted incidence rates of all-cancer from 2001 to 2016 for all regions of England for males; 95% credible intervals in brackets** 42
7 **S7 Table. Absolute deprivation differences ($\mathbf{AD}_{t,r}$), per 100,000 people, in age-standardised fitted incidence rates of all-cancer from 2001 to 2016 for all regions in England for females; 95% credible intervals in brackets** 43
8 **S8 Table. Relative deprivation differences ($\mathbf{RD}_{t,r}$) in age-standardised fitted incidence rates of all-cancer from 2001 to 2016 for all regions in England for females; 95% credible intervals in brackets** 44
9 **S9 Table. Absolute deprivation differences ($\mathbf{AD}_{t,r}$), per 100,000 people, in age-standardised fitted mortality rates of all-cancer from 2001 to 2016 for all regions in England for males; 95% credible intervals in brackets** . 59
10 **S10 Table. Relative deprivation differences ($\mathbf{RD}_{t,r}$) in age-standardised fitted mortality rates of all-cancer from 2001 to 2016 in for all regions in England for males; 95% credible intervals in brackets** 60
11 **S11 Table. Absolute deprivation differences ($\mathbf{AD}_{t,r}$), per 100,000 people, in age-standardised fitted mortality rates of all-cancer from 2001 to 2016 for all regions in England for females; 95% credible intervals in brackets** 61
12 **S12 Table. Relative deprivation differences ($\mathbf{RD}_{t,r}$) in age-standardised fitted mortality rates of all-cancer from 2001 to 2016 for all regions in England for females; 95% credible intervals in brackets** 62
13 **S13 Table. Estimated coefficients for the best fitting model for lung cancer morbidity.** 63
14 **S14 Table. Estimated coefficients for the best fitting model for male lung cancer mortality.** 66
15 **S15 Table. Estimated coefficients for the best fitting model for female lung cancer mortality.** 68
16 **S16 Table. Absolute deprivation differences ($\mathbf{AD}_{t,r}$), per 100,000 people, in age-standardised fitted incidence rates of lung, trachea and bronchus cancer from 2001 to 2016 for all regions in England for males; 95% credible intervals in brackets** 84

17 **S17 Table. Relative deprivation differences ($\mathbf{RD}_{t,r}$) in age-standardised fitted incidence rates of lung, trachea and bronchus cancer from 2001 to 2016 for all regions in England for males; 95% credible intervals in brackets** 85
18 **S18 Table. Absolute deprivation differences ($\mathbf{AD}_{t,r}$), per 100,000 people, in age-standardised fitted incidence rates of lung, trachea and bronchus cancer from 2001 to 2016 for all regions in England for females; 95% credible intervals in brackets** 86
19 **S19 Table. Relative deprivation differences ($\mathbf{RD}_{t,r}$) in age-standardised fitted incidence rates of lung, trachea and bronchus cancer from 2001 to 2016 for all regions in England for females; 95% credible intervals in brackets** 87
20 **S20 Table. Absolute deprivation differences ($\mathbf{AD}_{t,r}$), per 100,000 people, in age-standardised fitted mortality rates of lung, trachea and bronchus cancer from 2001 to 2016 for all regions in England for males; 95% credible intervals in brackets** 102
21 **S21 Table. Relative deprivation differences ($\mathbf{RD}_{t,r}$) in age-standardised fitted mortality rates of lung, trachea and bronchus cancer from 2001 to 2016 for all regions in England for males; 95% credible intervals in brackets** 103
22 **S22 Table. Absolute deprivation differences ($\mathbf{AD}_{t,r}$), per 100,000 people, in age-standardised fitted mortality rates of lung, trachea and bronchus cancer from 2001 to 2016 for all regions in England for females; 95% credible intervals in brackets** 104
23 **S23 Table. Relative deprivation differences ($\mathbf{RD}_{t,r}$) in age-standardised fitted mortality rates of lung, trachea and bronchus cancer from 2001 to 2016 for all regions in England for females; 95% credible intervals in brackets** 105
24 **S24 Table. Estimated coefficients for the best fitting model for bowel cancer morbidity.** 106
25 **S25 Table. Estimated coefficients for the best fitting model for male bowel cancer mortality.** 107
26 **S26 Table. Estimated coefficients for the best fitting model for female bowel cancer mortality.** 107
27 **S27 Table. Absolute deprivation differences ($\mathbf{AD}_{t,r}$), per 100,000 people, in age-standardised fitted incidence rates of bowel cancer from 2001 to 2016 for all regions in England for males; 95% credible intervals in brackets** 122
28 **S28 Table. Relative deprivation differences ($\mathbf{RD}_{t,r}$) in age-standardised fitted incidence rates of bowel cancer from 2001 to 2016 for all regions in England for males; 95% credible intervals in brackets** 123
29 **S29 Table. Absolute deprivation differences ($\mathbf{AD}_{t,r}$), per 100,000 people, in age-standardised fitted incidence rates of bowel cancer from 2001 to 2016 for all regions in England for females; 95% credible intervals in brackets** 124
30 **S30 Table. Relative deprivation differences ($\mathbf{AD}_{t,r}$) in age-standardised fitted incidence rates of bowel cancer from 2001 to 2016 for all regions in England for females; 95% credible intervals in brackets** 125
31 **S31 Table. Absolute deprivation differences ($\mathbf{AD}_{t,r}$), per 100,000 people, in age-standardised fitted mortality rates of bowel cancer from 2001 to 2016 for all regions in England for males; 95% credible intervals in brackets** 140
32 **S32 Table. Relative deprivation differences ($\mathbf{RD}_{t,r}$) in age-standardised fitted mortality rates of bowel cancer from 2001 to 2016 for all regions in England for males; 95% credible intervals in brackets** 141

33 **S33 Table. Absolute deprivation differences ($AD_{t,r}$), per 100,000 people, in age-standardised fitted mortality rates of bowel cancer from 2001 to 2016 for all regions in England for females; 95% credible intervals in brackets** 142
34 **S34 Table. Relative deprivation differences ($RD_{t,r}$) in age-standardised fitted mortality rates of bowel cancer from 2001 to 2016 for all regions in England for females; 95% credible intervals in brackets** 143
35 **S35 Table. Estimated coefficients for the best fitting model for prostate cancer morbidity.** 144
36 **S36 Table. Estimated coefficients for the best fitting model for prostate cancer mortality.** 146
37 **S37 Table. Absolute deprivation differences ($AD_{t,r}$), per 100,000 people, in age-standardised fitted incidence rates of malignant neoplasm of prostate from 2001 to 2016 for all regions in England for males; 95% credible intervals in brackets** 154
38 **S38 Table. Relative deprivation differences ($RD_{t,r}$) in age-standardised fitted incidence rates of malignant neoplasm of prostate from 2001 to 2016 for all regions in England for males; 95% credible intervals in brackets** 155
39 **S39 Table. Estimated coefficients for the best fitting model for breast cancer morbidity.** 162
40 **S40 Table. Estimated coefficients for the best fitting model for breast cancer mortality.** 162
41 **S41 Table. Absolute deprivation differences ($AD_{t,r}$), per 100,000 people, in age-standardised fitted incidence rates of malignant neoplasm of breast from 2001 to 2016 for all regions in England for males; 95% credible intervals in brackets** 170
42 **S42 Table. Relative deprivation differences ($RD_{t,r}$) in age-standardised fitted incidence rates of malignant neoplasm of breast from 2001 to 2016 for all regions in England for males; 95% credible intervals in brackets** . 171
43 **S43 Table. Absolute regional differences (AD_t), per 100,000 people, and relative regional differences (RD_t) in age-standardised fitted mortality rates of prostate and breast cancers from 2001 to 2016; 95% credible intervals in brackets** 178

List of Figures

1 **S1 Fig. Heat map of Pearson residuals for all-cancer morbidity for males in North East, London, and South West, deprivation deciles 1, 5, and 10: orange/light blue cells indicate areas with good fit, while red/dark blue cells indicate areas with poor fit. Note that there is a small number of residuals greater than 4, and these are included in the last category.** . 27
2 **S2 Fig. Heat map of Pearson residuals for all-cancer morbidity for females in North East, London, and South West, deprivation deciles 1, 5, and 10: orange/light blue cells indicate areas with good fit, while red/dark blue cells indicate areas with poor fit. Note that there is a small number of residuals greater than 4, and these are included in the last category.** 28
3 **S3 Fig. All-cancer morbidity for males, age 62, deprivation deciles 1-4 for all regions in England between 2001 and 2016: observed rates (dots), fitted rates (lines), with 95% credible intervals for the fitted rates.** 29
4 **S4 Fig. All-cancer morbidity for males, age 62, deprivation deciles 5-8 for all regions in England between 2001 and 2016: observed rates (dots), fitted rates (lines), with 95% credible intervals for the fitted rates.** 30

5 **S5 Fig. All-cancer morbidity for males, age 62, deprivation deciles 9-10 for all regions in England between 2001 and 2016: observed rates (dots), fitted rates (lines), with 95% credible intervals for the fitted rates.** 31
6 **S6 Fig. All-cancer morbidity for males, age 72, deprivation deciles 1-4 for all regions in England between 2001 and 2016: observed rates (dots), fitted rates (lines), with 95% credible intervals for the fitted rates.** 32
7 **S7 Fig. All-cancer morbidity for males, age 72, deprivation deciles 5-8 for all regions in England between 2001 and 2016: observed rates (dots), fitted rates (lines), with 95% credible intervals for the fitted rates.** 33
8 **S8 Fig. All-cancer morbidity for males, age 72, deprivation deciles 9-10 for all regions in England between 2001 and 2016: observed rates (dots), fitted rates (lines), with 95% credible intervals for the fitted rates.** 34
9 **S9 Fig. All-cancer morbidity for females, age 62, deprivation deciles 1-4 for all regions in England between 2001 and 2016: observed rates (dots), fitted rates (lines), with 95% credible intervals for the fitted rates.** 35
10 **S10 Fig. All-cancer morbidity for females, age 62, deprivation deciles 5-8 for all regions in England between 2001 and 2016: observed rates (dots), fitted rates (lines), with 95% credible intervals for the fitted rates.** 36
11 **S11 Fig. All-cancer morbidity for females, age 62, deprivation deciles 9-10 for all regions in England between 2001 and 2016: observed rates (dots), fitted rates (lines), with 95% credible intervals for the fitted rates.** 37
12 **S12 Fig. All-cancer morbidity for females, age 72, deprivation deciles 1-4 for all regions in England between 2001 and 2016: observed rates (dots), fitted rates (lines), with 95% credible intervals for the fitted rates.** 38
13 **S13 Fig. All-cancer morbidity for females, age 72, deprivation deciles 5-8 for all regions in England between 2001 and 2016: observed rates (dots), fitted rates (lines), with 95% credible intervals for the fitted rates.** 39
14 **S14 Fig. All-cancer morbidity for females, age 72, deprivation deciles 9-10 for all regions in England between 2001 and 2016: observed rates (dots), fitted rates (lines), with 95% credible intervals for the fitted rates.** 40
15 **S15 Fig. Heat map of Pearson residuals for all-cancer mortality for males in North East, London, and South West, deprivation deciles 1, 5, and 10: orange/light blue cells indicate areas with good fit, while red/dark blue cells indicate areas with poor fit. Note that there is a small number of residuals greater than 4, and these are included in the last category.** . 45
16 **S16 Fig. Heat map of Pearson residuals for all-cancer mortality for females in North East, London, and South West, deprivation deciles 1, 5, and 10: orange/light blue cells indicate areas with good fit, while red/dark blue cells indicate areas with poor fit. Note that there is a small number of residuals greater than 4, and these are included in the last category.** 46
17 **S17 Fig. All-cancer mortality for males, age 62, deprivation deciles 1-4 for all regions in England between 2001 and 2016: observed rates (dots), fitted rates (lines), with 95% credible intervals for the fitted rates.** 47
18 **S18 Fig. All-cancer mortality for males, age 62, deprivation deciles 5-8 for all regions in England between 2001 and 2016: observed rates (dots), fitted rates (lines), with 95% credible intervals for the fitted rates.** 48
19 **S19 Fig. All-cancer mortality for males, age 62, deprivation deciles 9-10 for all regions in England between 2001 and 2016: observed rates (dots), fitted rates (lines), with 95% credible intervals for the fitted rates.** 49
20 **S20 Fig. All-cancer mortality for males, age 72, deprivation deciles 1-4 for all regions in England between 2001 and 2016: observed rates (dots), fitted rates (lines), with 95% credible intervals for the fitted rates.** 50

21 **S21 Fig. All-cancer mortality for males, age 72, deprivation deciles 5-8 for all regions in England between 2001 and 2016: observed rates (dots), fitted rates (lines), with 95% credible intervals for the fitted rates.** 51
22 **S22 Fig. All-cancer mortality for males, age 72, deprivation deciles 9-10 for all regions in England between 2001 and 2016: observed rates (dots), fitted rates (lines), with 95% credible intervals for the fitted rates.** 52
23 **S23 Fig. All-cancer mortality for females, age 62, deprivation deciles 1-4 for all regions in England between 2001 and 2016: observed rates (dots), fitted rates (lines), with 95% credible intervals for the fitted rates.** 53
24 **S24 Fig. All-cancer mortality for females, age 62, deprivation deciles 5-8 for all regions in England between 2001 and 2016: observed rates (dots), fitted rates (lines), with 95% credible intervals for the fitted rates.** 54
25 **S25 Fig. All-cancer mortality for females, age 62, deprivation deciles 9-10 for all regions in England between 2001 and 2016: observed rates (dots), fitted rates (lines), with 95% credible intervals for the fitted rates.** 55
26 **S26 Fig. All-cancer mortality for females, age 72, deprivation deciles 1-4 for all regions in England between 2001 and 2016: observed rates (dots), fitted rates (lines), with 95% credible intervals for the fitted rates.** 56
27 **S27 Fig. All-cancer mortality for females, age 72, deprivation deciles 5-8 for all regions in England between 2001 and 2016: observed rates (dots), fitted rates (lines), with 95% credible intervals for the fitted rates.** 57
28 **S28 Fig. All-cancer mortality for females, age 72, deprivation deciles 9-10 for all regions in England between 2001 and 2016: observed rates (dots), fitted rates (lines), with 95% credible intervals for the fitted rates.** 58
29 **S29 Fig. Heat map of Pearson residuals for trachea, bronchus and lung cancer morbidity for males in North East, London, and South West, deprivation deciles 1, 5, and 10: orange/light blue cells indicate areas with good fit, while red/dark blue cells indicate areas with poor fit. Note that there is a small number of residuals greater than 4, and these are included in the last category.** 70
30 **S30 Fig. Heat map of Pearson residuals for trachea, bronchus and lung cancer morbidity for females in North East, London, and South West, deprivation deciles 1, 5, and 10: orange/light blue cells indicate areas with good fit, while red/dark blue cells indicate areas with poor fit. Note that there is a small number of residuals greater than 4, and these are included in the last category.** 71
31 **S31 Fig. Trachea, bronchus and lung cancer morbidity for males, age 62, deprivation deciles 1-4 for all regions in England between 2001 and 2016: observed rates (dots), fitted rates (lines), with 95% credible intervals for the fitted rates.** 72
32 **S32 Fig. Trachea, bronchus and lung cancer morbidity for males, age 62, deprivation deciles 5-8 for all regions in England between 2001 and 2016: observed rates (dots), fitted rates (lines), with 95% credible intervals for the fitted rates.** 73
33 **S33 Fig. Trachea, bronchus and lung cancer morbidity for males, age 62, deprivation deciles 9-10 for all regions in England between 2001 and 2016: observed rates (dots), fitted rates (lines), with 95% credible intervals for the fitted rates.** 74
34 **S34 Fig. Trachea, bronchus and lung cancer morbidity for males, age 72, deprivation deciles 1-4 for all regions in England between 2001 and 2016: observed rates (dots), fitted rates (lines), with 95% credible intervals for the fitted rates.** 75

35 **S35 Fig. Trachea, bronchus and lung cancer morbidity for males, age 72, deprivation deciles 5-8 for all regions in England between 2001 and 2016: observed rates (dots), fitted rates (lines), with 95% credible intervals for the fitted rates.** 76

36 **S36 Fig. Trachea, bronchus and lung cancer morbidity for males, age 72, deprivation deciles 9-10 for all regions in England between 2001 and 2016: observed rates (dots), fitted rates (lines), with 95% credible intervals for the fitted rates.** 77

37 **S37 Fig. Trachea, bronchus and lung cancer morbidity for females, age 62, deprivation deciles 1-4 for all regions in England between 2001 and 2016: observed rates (dots), fitted rates (lines), with 95% credible intervals for the fitted rates.** 78

38 **S38 Fig. Trachea, bronchus and lung cancer morbidity for females, age 62, deprivation deciles 5-8 for all regions in England between 2001 and 2016: observed rates (dots), fitted rates (lines), with 95% credible intervals for the fitted rates.** 79

39 **S39 Fig. Trachea, bronchus and lung cancer morbidity for females, age 62, deprivation deciles 9-10 for all regions in England between 2001 and 2016: observed rates (dots), fitted rates (lines), with 95% credible intervals for the fitted rates.** 80

40 **S40 Fig. Trachea, bronchus and lung cancer morbidity for females, age 72, deprivation deciles 1-4 for all regions in England between 2001 and 2016: observed rates (dots), fitted rates (lines), with 95% credible intervals for the fitted rates.** 81

41 **S41 Fig. Trachea, bronchus and lung cancer morbidity for females, age 72, deprivation deciles 5-8 for all regions in England between 2001 and 2016: observed rates (dots), fitted rates (lines), with 95% credible intervals for the fitted rates.** 82

42 **S42 Fig. Trachea, bronchus and lung cancer morbidity for females, age 72, deprivation deciles 9-10 for all regions in England between 2001 and 2016: observed rates (dots), fitted rates (lines), with 95% credible intervals for the fitted rates.** 83

43 **S43 Fig. Heat map of Pearson residuals for trachea, bronchus and lung cancer mortality for males in North East, London, and South West, deprivation deciles 1, 5, and 10: orange/light blue cells indicate areas with good fit, while red/dark blue cells indicate areas with poor fit. Note that there is a small number of residuals greater than 4, and these are included in the last category.** 88

44 **S44 Fig. Heat map of Pearson residuals for trachea, bronchus and lung cancer mortality for females in North East, London, and South West, deprivation deciles 1, 5, and 10: orange/light blue cells indicate areas with good fit, while red/dark blue cells indicate areas with poor fit. Note that there is a small number of residuals greater than 4, and these are included in the last category.** 89

45 **S45 Fig. Trachea, bronchus and lung cancer mortality for males, age 62, deprivation deciles 1-4 for all regions in England between 2001 and 2016: observed rates (dots), fitted rates (lines), with 95% credible intervals for the fitted rates.** 90

46 **S46 Fig. Trachea, bronchus and lung cancer mortality for males, age 62, deprivation deciles 5-8 for all regions in England between 2001 and 2016: observed rates (dots), fitted rates (lines), with 95% credible intervals for the fitted rates.** 91

47 **S47 Fig. Trachea, bronchus and lung cancer mortality for males, age 62, deprivation deciles 9-10 for all regions in England between 2001 and 2016: observed rates (dots), fitted rates (lines), with 95% credible intervals for the fitted rates.** 92
48 **S48 Fig. Trachea, bronchus and lung cancer mortality for males, age 72, deprivation deciles 1-4 for all regions in England between 2001 and 2016: observed rates (dots), fitted rates (lines), with 95% credible intervals for the fitted rates.** 93
49 **S49 Fig. Trachea, bronchus and lung cancer mortality for males, age 72, deprivation deciles 5-8 for all regions in England between 2001 and 2016: observed rates (dots), fitted rates (lines), with 95% credible intervals for the fitted rates.** 94
50 **S50 Fig. Trachea, bronchus and lung cancer mortality for males, age 72, deprivation deciles 9-10 for all regions in England between 2001 and 2016: observed rates (dots), fitted rates (lines), with 95% credible intervals for the fitted rates.** 95
51 **S51 Fig. Trachea, bronchus and lung cancer mortality for females, age 62, deprivation deciles 1-4 for all regions in England between 2001 and 2016: observed rates (dots), fitted rates (lines), with 95% credible intervals for the fitted rates.** 96
52 **S52 Fig. Trachea, bronchus and lung cancer mortality for females, age 62, deprivation deciles 5-8 for all regions in England between 2001 and 2016: observed rates (dots), fitted rates (lines), with 95% credible intervals for the fitted rates.** 97
53 **S53 Fig. Trachea, bronchus and lung cancer mortality for females, age 62, deprivation deciles 9-10 for all regions in England between 2001 and 2016: observed rates (dots), fitted rates (lines), with 95% credible intervals for the fitted rates.** 98
54 **S54 Fig. Trachea, bronchus and lung cancer mortality for females, age 72, deprivation deciles 1-4 for all regions in England between 2001 and 2016: observed rates (dots), fitted rates (lines), with 95% credible intervals for the fitted rates.** 99
55 **S55 Fig. Trachea, bronchus and lung cancer mortality for females, age 72, deprivation deciles 5-8 for all regions in England between 2001 and 2016: observed rates (dots), fitted rates (lines), with 95% credible intervals for the fitted rates.** 100
56 **S56 Fig. Trachea, bronchus and lung cancer mortality for females, age 72, deprivation deciles 9-10 for all regions in England between 2001 and 2016: observed rates (dots), fitted rates (lines), with 95% credible intervals for the fitted rates.** 101
57 **S57 Fig. Heat map of Pearson residuals for bowel cancer morbidity for males in North East, London, and South West, deprivation deciles 1, 5, and 10: orange/light blue cells indicate areas with good fit, while red/dark blue cells indicate areas with poor fit. Note that there is a small number of residuals greater than 4, and these are included in the last category.** 108
58 **S58 Fig. Heat map of Pearson residuals for bowel cancer morbidity for females in North East, London, and South West, deprivation deciles 1, 5, and 10: orange/light blue cells indicate areas with good fit, while red/dark blue cells indicate areas with poor fit. Note that there is a small number of residuals greater than 4, and these are included in the last category.** 109

59 **S59 Fig. Bowel cancer morbidity for males, age 62, deprivation deciles 1-4 for all regions in England between 2001 and 2016: observed rates (dots), fitted rates (lines), with 95% credible intervals for the fitted rates.** 110
60 **S60 Fig. Bowel cancer morbidity for males, age 62, deprivation deciles 5-8 for all regions in England between 2001 and 2016: observed rates (dots), fitted rates (lines), with 95% credible intervals for the fitted rates.** 111
61 **S61 Fig. Bowel cancer morbidity for males, age 62, deprivation deciles 9-10 for all regions in England between 2001 and 2016: observed rates (dots), fitted rates (lines), with 95% credible intervals for the fitted rates.** 112
62 **S62 Fig. Bowel cancer morbidity for males, age 72, deprivation deciles 1-4 for all regions in England between 2001 and 2016: observed rates (dots), fitted rates (lines), with 95% credible intervals for the fitted rates.** 113
63 **S63 Fig. Bowel cancer morbidity for males, age 72, deprivation deciles 5-8 for all regions in England between 2001 and 2016: observed rates (dots), fitted rates (lines), with 95% credible intervals for the fitted rates.** 114
64 **S64 Fig. Bowel cancer morbidity for males, age 72, deprivation deciles 9-10 for all regions in England between 2001 and 2016: observed rates (dots), fitted rates (lines), with 95% credible intervals for the fitted rates.** 115
65 **S65 Fig. Bowel cancer morbidity for females, age 62, deprivation deciles 1-4 for all regions in England between 2001 and 2016: observed rates (dots), fitted rates (lines), with 95% credible intervals for the fitted rates.** 116
66 **S66 Fig. Bowel cancer morbidity for females, age 62, deprivation deciles 5-8 for all regions in England between 2001 and 2016: observed rates (dots), fitted rates (lines), with 95% credible intervals for the fitted rates.** 117
67 **S67 Fig. Bowel cancer morbidity for females, age 62, deprivation deciles 9-10 for all regions in England between 2001 and 2016: observed rates (dots), fitted rates (lines), with 95% credible intervals for the fitted rates.** 118
68 **S68 Fig. Bowel cancer morbidity for females, age 72, deprivation deciles 1-4 for all regions in England between 2001 and 2016: observed rates (dots), fitted rates (lines), with 95% credible intervals for the fitted rates.** 119
69 **S69 Fig. Bowel cancer morbidity for females, age 72, deprivation deciles 5-8 for all regions in England between 2001 and 2016: observed rates (dots), fitted rates (lines), with 95% credible intervals for the fitted rates.** 120
70 **S70 Fig. Bowel cancer morbidity for females, age 72, deprivation deciles 9-10 for all regions in England between 2001 and 2016: observed rates (dots), fitted rates (lines), with 95% credible intervals for the fitted rates.** 121
71 **S71 Fig. Heat map of Pearson residuals for bowel cancer mortality for males in North East, London, and South West, deprivation deciles 1, 5, and 10: orange/light blue cells indicate areas with good fit, while red/dark blue cells indicate areas with poor fit. Note that there is a small number of residuals greater than 4, and these are included in the last category.** 126
72 **S72 Fig. Heat map of Pearson residuals for bowel cancer mortality for females in North East, London, and South West, deprivation deciles 1, 5, and 10: orange/light blue cells indicate areas with good fit, while red/dark blue cells indicate areas with poor fit. Note that there is a small number of residuals greater than 4, and these are included in the last category.** 127
73 **S73 Fig. Bowel cancer mortality for males, age 62, deprivation deciles 1-4 for all regions in England between 2001 and 2016: observed rates (dots), fitted rates (lines), with 95% credible intervals for the fitted rates.** 128
74 **S74 Fig. Bowel cancer mortality for males, age 62, deprivation deciles 5-8 for all regions in England between 2001 and 2016: observed rates (dots), fitted rates (lines), with 95% credible intervals for the fitted rates.** 129

75 **S75 Fig. Bowel cancer mortality for males, age 62, deprivation deciles 9-10 for all regions in England between 2001 and 2016: observed rates (dots), fitted rates (lines), with 95% credible intervals for the fitted rates.** 130
76 **S76 Fig. Bowel cancer mortality for males, age 72, deprivation deciles 1-4 for all regions in England between 2001 and 2016: observed rates (dots), fitted rates (lines), with 95% credible intervals for the fitted rates.** 131
77 **S77 Fig. Bowel cancer mortality for males, age 72, deprivation deciles 5-8 for all regions in England between 2001 and 2016: observed rates (dots), fitted rates (lines), with 95% credible intervals for the fitted rates.** 132
78 **S78 Fig. Bowel cancer mortality for males, age 72, deprivation deciles 9-10 for all regions in England between 2001 and 2016: observed rates (dots), fitted rates (lines), with 95% credible intervals for the fitted rates.** 133
79 **S79 Fig. Bowel cancer mortality for females, age 62, deprivation deciles 1-4 for all regions in England between 2001 and 2016: observed rates (dots), fitted rates (lines), with 95% credible intervals for the fitted rates.** 134
80 **S80 Fig. Bowel cancer mortality for females, age 62, deprivation deciles 5-8 for all regions in England between 2001 and 2016: observed rates (dots), fitted rates (lines), with 95% credible intervals for the fitted rates.** 135
81 **S81 Fig. Bowel cancer mortality for females, age 62, deprivation deciles 9-10 for all regions in England between 2001 and 2016: observed rates (dots), fitted rates (lines), with 95% credible intervals for the fitted rates.** 136
82 **S82 Fig. Bowel cancer mortality for females, age 72, deprivation deciles 1-4 for all regions in England between 2001 and 2016: observed rates (dots), fitted rates (lines), with 95% credible intervals for the fitted rates.** 137
83 **S83 Fig. Bowel cancer mortality for females, age 72, deprivation deciles 5-8 for all regions in England between 2001 and 2016: observed rates (dots), fitted rates (lines), with 95% credible intervals for the fitted rates.** 138
84 **S84 Fig. Bowel cancer mortality for females, age 72, deprivation deciles 9-10 for all regions in England between 2001 and 2016: observed rates (dots), fitted rates (lines), with 95% credible intervals for the fitted rates.** 139
85 **S85 Fig. Heat map of Pearson residuals for prostate cancer morbidity in North East, London, and South West, deprivation deciles 1, 5, and 10: orange/light blue cells indicate areas with good fit, while red/dark blue cells indicate areas with poor fit. Note that there is a small number of residuals greater than 4, and these are included in the last category.** . . 147
86 **S86 Fig.** Prostate cancer morbidity, age 62, deprivation deciles 1-4 for all regions in England between 2001 and 2006: observed rates (dots), fitted rates (lines), with 95% credible intervals for the fitted rates. 148
87 **S87 Fig.** Prostate cancer morbidity, age 62, deprivation deciles 5-8 for all regions in England between 2001 and 2006: observed rates (dots), fitted rates (lines), with 95% credible intervals for the fitted rates. 149
88 **S88 Fig.** Prostate cancer morbidity, age 62, deprivation deciles 9-10 for all regions in England between 2001 and 2006: observed rates (dots), fitted rates (lines), with 95% credible intervals for the fitted rates. 150
89 **S89 Fig.** Prostate cancer morbidity, age 72, deprivation deciles 1-4 for all regions in England between 2001 and 2016: observed rates (dots), fitted rates (lines), with 95% credible intervals for the fitted rates. 151
90 **S90 Fig.** Prostate cancer morbidity, age 72, deprivation deciles 5-8 for all regions in England between 2001 and 2016: observed rates (dots), fitted rates (lines), with 95% credible intervals for the fitted rates. 152
91 **S91 Fig.** Prostate cancer morbidity, age 72, deprivation deciles 9-10 for all regions in England between 2001 and 2016: observed rates (dots), fitted rates (lines), with 95% credible intervals for the fitted rates. 153

92 **S92 Fig. Heat map of Pearson residuals for prostate cancer mortality in North East, London, and South West, deprivation deciles 1, 5, and 10: orange/light blue cells indicate areas with good fit, while red/dark blue cells indicate areas with poor fit. Note that there is a small number of residuals greater than 4, and these are included in the last category.** . . 156
93 **S93 Fig. Prostate cancer mortality, ages 47-57 for all regions in England between 2001 and 2006: observed rates (dots), fitted rates (lines), with 95% credible intervals for the fitted rates.** 157
94 **S94 Fig. Prostate cancer mortality, ages 62-67 for all regions in England between 2001 and 2016: observed rates (dots), fitted rates (lines), with 95% credible intervals for the fitted rates.** 158
95 **S95 Fig. Prostate cancer mortality, ages 72-77 for all regions in England between 2001 and 2016: observed rates (dots), fitted rates (lines), with 95% credible intervals for the fitted rates.** 159
96 **S96 Fig. Prostate cancer mortality, ages 82-90 for all regions in England between 2001 and 2016: observed rates (dots), fitted rates (lines), with 95% credible intervals for the fitted rates.** 160
97 **S97 Fig. Prostate cancer mortality for all regions in England in 2001 and 2016: observed rates (dots), fitted rates (lines), with 95% credible intervals for the fitted rates.** 161
98 **S98 Fig. Heat map of Pearson residuals for breast cancer morbidity in North East, London, and South West, deprivation deciles 1, 5, and 10: orange/light blue cells indicate areas with good fit, while red/dark blue cells indicate areas with poor fit. Note that there is a small number of residuals greater than 4, and these are included in the last category.** . . 163
99 **S99 Fig. Breast cancer morbidity, age 52, deprivation deciles 1-4 for all regions in England between 2001 and 2016: observed rates (dots), fitted rates (lines), with 95% credible intervals for the fitted rates.** 164
100 **S100 Fig. Breast cancer morbidity, age 52, deprivation deciles 5-8 for all regions in England between 2001 and 2016: observed rates (dots), fitted rates (lines), with 95% credible intervals for the fitted rates.** 165
101 **S101 Fig. Breast cancer morbidity, age 52, deprivation deciles 9-10 for all regions in England between 2001 and 2016: observed rates (dots), fitted rates (lines), with 95% credible intervals for the fitted rates.** 166
102 **S102 Fig. Breast cancer morbidity, age 72, deprivation deciles 1-4 for all regions in England between 2001 and 2016: observed rates (dots), fitted rates (lines), with 95% credible intervals for the fitted rates.** 167
103 **S103 Fig. Breast cancer morbidity, age 72, deprivation deciles 5-8 for all regions in England between 2001 and 2016: observed rates (dots), fitted rates (lines), with 95% credible intervals for the fitted rates.** 168
104 **S104 Fig. Breast cancer morbidity, age 72, deprivation deciles 9-10 for all regions in England between 2001 and 2016: observed rates (dots), fitted rates (lines), with 95% credible intervals for the fitted rates.** 169
105 **S105 Fig. Heat map of Pearson residuals for breast cancer mortality in North East, London, and South West, deprivation deciles 1, 5, and 10: orange/light blue cells indicate areas with good fit, while red/dark blue cells indicate areas with poor fit. Note that there is a small number of residuals greater than 4, and these are included in the last category.** . . 172
106 **S106 Fig. Breast cancer mortality, ages 37-42 for all regions in England between 2001 and 2006: observed rates (dots), fitted rates (lines), with 95% credible intervals for the fitted rates.** 173
107 **S107 Fig. Breast cancer mortality, ages 47-57 for all regions in England between 2001 and 2016: observed rates (dots), fitted rates (lines), with 95% credible intervals for the fitted rates.** 174

108 **S108 Fig. Breast cancer mortality, ages 62-72 for all regions in England between 2001 and 2016: observed rates (dots), fitted rates (lines), with 95% credible intervals for the fitted rates.** 175
109 **S109 Fig. Breast cancer mortality, ages 77-90 for all regions in England between 2001 and 2016: observed rates (dots), fitted rates (lines), with 95% credible intervals for the fitted rates.** 176
110 **S110 Fig. Breast cancer mortality for all regions in England in 2001 and 2016: observed rates (dots), fitted rates (lines), with 95% credible intervals for the fitted rates.** 177

1 Best fitted models for cancer morbidity and mortality

Model for all-cancer incidence rates The modelling for all-cancer incidence is done separately for each gender from age 22 onwards. The covariates are regressed on the location parameter of the lognormal distribution for males as follows:

$$\mu_{a,t,d,r} = \beta_0 + \beta_{1,a} + \beta_2 t + \beta_3 t^2 + \beta_4 t^3 + \beta_{5,r} + \beta_{6,d} + \beta_{7,a,d} + \beta_{8,a} t + \beta_{9,r} t + \beta_{10,a} t^2 + \beta_{11,r} t^2.$$

Here age is a categorical variable with levels $a = 1, \ldots, 14$ where a maps to $\{22, 27, \ldots, 90\}$, respectively, and $\beta_{1,a}$ denotes the corresponding coefficients; year, denoted by t, is a numerical variable with $t \in \{2001, 2002, \ldots, 2016\}$, including higher powers of year (up to 3); region is a categorical variable with levels $r = 1, \ldots, 9$ where r maps to {North East, North West, Yorkshire and the Humber, East Midlands, West Midlands, East, London, South East and South West}, respectively, and $\beta_{5,r}$ denotes the corresponding coefficients; deprivation is a categorical variable with levels $d = 1, 2, \ldots, 10$ where 1 is the most deprived level and 10 is the least deprived level, and $\beta_{6,d}$ shows the relevant coefficient estimates.

The structure of the location parameter for female all-cancer incidence is obtained as given below:

$$\mu_{a,t,d,r} = \beta_0 + \beta_{1,a} + \beta_2 t + \beta_3 t^2 + \beta_4 t^3 + \beta_{5,r} + \beta_{6,d} + \beta_{7,a,d} + \beta_{8,a} t + \beta_{9,r} t + \beta_{10,a,r} + \beta_{11,a} t^2.$$

Note that we have one different interaction term, i.e. the one between age and region, $\beta_{10,a,r}$, compared to male all-cancer incidence.

Model for trachea, bronchus and lung cancer incidence rates Malignant neoplasm of trachea, bronchus and lung is modelled from age 47 onwards by following the general literature. The structure of the location parameter of the lognormal distribution is shown explicitly as follows:

$$\begin{aligned}\mu_{a,t,d,g,r} = \beta_0 + \beta_{1,a} + \beta_2 t + \beta_{3,g} + \beta_{4,r} + \beta_{5,d} + \beta_{6,a,g} + \beta_{7,a,d} + \beta_{8,g} t \\ + \beta_{9,a} t + \beta_{10,g,r} + \beta_{11,d,r} + \beta_{12,a,r} + \beta_{13,d} t + \beta_{14,r} t.\end{aligned}$$

Here age is a categorical variable with levels $a = 1, \ldots, 9$ where a maps to $\{47, 52, \ldots, 90\}$, respectively; year is a numerical variable; gender is a categorical variable with levels $g = 1, 2$ where g maps to {Male, Female}, respectively, and $\beta_{3,g}$ shows the relevant coefficient estimates. Several interaction terms between age and other covariates, such as gender, $\beta_{6,a,g}$, or deprivation, $\beta_{7,a,d}$, are added into the model in addition to some other interaction terms, e.g. between gender and region, $\beta_{10,g,r}$, or age and region, $\beta_{12,a,r}$.

Model for bowel cancer incidence rates Malignant neoplasm of bowel is modelled from age 47 onwards. The location parameter of the lognormal distribution is shown as follows:

$$\begin{aligned}\mu_{a,t,d,r} = \beta_0 + \beta_{1,a} + \beta_{2,1} t_1 + \beta_{2,2} t_2 + \beta_{3,g} + \beta_{4,r} + \beta_{5,d} + \beta_{6,1} t_1^2 + \beta_{6,2} t_2^2 + \beta_{7,a,g} + \beta_{8,1,a} t_1 \\ + \beta_{8,2,a} t_2 + \beta_{9,g,r} + \beta_{10,g,d} + \beta_{11,1,g} t_1 + \beta_{11,2,g} t_2.\end{aligned}$$

Here age is a categorical variable with levels $a = 1, \ldots, 9$ where a maps to $\{47, 52, \ldots, 90\}$, respectively; year, denoted by t_1 and t_2, is a numerical variable with $t_1 \leq 2006$ and $t_2 > 2006$ for 2001-2016, and $\beta_{2,1}$ and $\beta_{2,2}$ denote the corresponding coefficients, respectively.

Model for prostate cancer incidence rates Malignant neoplasm of prostate is modelled from age 47 onwards, as well. The location parameter of the lognormal distribution is expressed as given below:

$$\mu_{a,t,d,r} = \beta_0 + \beta_{1,a} + \beta_2 t + \beta_3 t^2 + \beta_4 t^3 + \beta_{5,r} + \beta_{6,d} + \beta_{7,a} t + \beta_{8,a,r} \\ + \beta_{9,r} t + \beta_{10,r,d} + \beta_{11,d} t + \beta_{12,a} t^2 + \beta_{13,r} t^2.$$

Here age is a categorical variable with levels $a = 1, \ldots, 9$ where a maps to $\{47, 52, \ldots, 90\}$, respectively; year is a numerical variable, including different powers of year (up to 3). Different than other cancer incidence models, we observe less interaction terms in this model. Yet, the interactions have also appeared between higher powers of year and other covariates, e.g. age, $\beta_{7,a} t$ or $\beta_{12,a} t^2$, and region, $\beta_{9,r} t$ or $\beta_{13,r} t^2$.

Model for breast cancer incidence rates The modelling for malignant neoplasm of breast is carried out from age 22 onwards as this cancer is also an issue for younger age groups.[1–4] The location parameter of the lognormal distribution has less complicated structure as given below:

$$\mu_{a,t,d,r} = \beta_0 + \beta_{1,a} + \beta_2 t + \beta_{3,r} + \beta_{4,d} + \beta_{5,a} t.$$

Here age is a categorical variable with levels $a = 1, \ldots, 14$ where a maps to $\{22, 32, \ldots, 90\}$, respectively; year is a numerical variable. The only interaction appeared between age and year such that $\beta_{5,a} t$.

Model for all-cancer mortality rates All-cancer death numbers are modelled separately for each gender from age 22 onwards. The deaths for the youngest two ages (i.e. ages 20-24 and 25-29, represented by age 25) are grouped together whilst the rest of the data represents five-year age bands until the oldest age group. The location parameter in the best fitted model of male all-cancer mortality is obtained as

$$\mu_{a,t,d,r} = \beta_0 + \beta_{1,a} + \beta_2 t + \beta_3 \text{AAD} + \beta_{4,r} + \beta_{5,d} + \beta_6 t^2 + \beta_{7,a,d} + \beta_{8,a} t + \beta_{9,r} t + \beta_{10,d} t \\ + \beta_{11,r} \text{AAD} + \beta_{12,a} t^2.$$

Here age is a categorical variable with levels $a = 1, \ldots, 13$ where a maps to $\{25, 32, 37, \ldots, 90\}$, respectively; year is a numerical variable; average age-at-diagnosis, denoted by AAD, is a numerical variable. Furthermore, many interaction terms appear in the model such as the ones between age and other covariates, e.g. deprivation, $\beta_{7,a,d}$, or year, $\beta_{8,a} t$, besides the ones between region and other covariates, e.g. year, $\beta_{9,r} t$, or average age-at-diagnosis, $\beta_{11,r}\text{AAD}$.

The structure of the location parameter for female all-cancer mortality is realised as:

$$\mu_{a,t,d,r} = \beta_0 + \beta_{1,a} + \beta_2 t + \beta_3 \text{AAD} + \beta_{4,r} + \beta_{5,d} + \beta_{6,a,d} + \beta_{7,a} t + \beta_{8,d} t + \beta_{9,r} t.$$

Note that none of the higher powers of year is involved in this model. Also, average age-at-diagnosis, i.e. AAD, has only average impact on female all-cancer mortality in all regions.

Model for trachea, bronchus and lung cancer mortality rates Lung cancer deaths are grouped with the neighbouring sites, namely trachea and bronchus. Then, trachea, bronchus and lung cancer mortality is modelled from age 47 onwards. Gender-specific best fitted models are obtained in order to avoid correlation between average age-at-diagnosis and gender covariates.

The structure of the location parameter in male lung cancer mortality model is obtained as

$$\mu_{a,t,d,r} = \beta_0 + \beta_{1,a} + \beta_2 t + \beta_3 \text{AAD} + \beta_{4,r} + \beta_{5,d} + \beta_{6,a} t + \beta_{7,a,d} + \beta_{8,d} t.$$

Here age is a categorical variable with levels $a = 1, \ldots, 9$ where a maps to $\{47,\ 52,\ \ldots,\ 90\}$, respectively; year and average age-at-diagnosis are numerical variables.

The location parameter for female lung cancer mortality is achieved as follows:

$$\mu_{a,t,d,r} = \beta_0 + \beta_{1,a} + \beta_2 t + \beta_3 \text{AAD} + \beta_{4,r} + \beta_{5,d} + \beta_6 t^2 + \beta_{7,a} t + \beta_{8,a,d} + \beta_{9,r}\text{AAD} + \beta_{10,a,r} \\ + \beta_{11,r} t + \beta_{12,d} t + \beta_{13,a} t^2.$$

Female lung cancer mortality model has a more complicated structure due to the existence of more interaction terms, specifically the ones between average age-at-diagnosis and region, $\beta_{9,r}\text{AAD}$, age and region, $\beta_{10,a,r}$, and region and year, $\beta_{11,r} t$.

Model for bowel cancer mortality rates Bowel cancer deaths are modelled from age 47 onwards separately for each gender.

The structure of the location parameter in male bowel cancer mortality is obtained as

$$\mu_{a,t,d,r} = \beta_0 + \beta_{1,a} + \beta_2 t + \beta_3 \text{AAD} + \beta_{4,r} + \beta_{5,d} + \beta_{6,a} t,$$

whilst the location parameter in the female model is expressed as

$$\mu_{a,t,d,r} = \beta_0 + \beta_{1,a} + \beta_2 t + \beta_3 \text{AAD} + \beta_{4,d} + \beta_{5,a} t.$$

Here age is a categorical variable with levels $a = 1, \ldots, 9$ where a maps to $\{47,\ 52,\ \ldots,\ 90\}$, respectively; year and average age-at-diagnosis are numerical variables.

Model for prostate cancer mortality rates Prostate cancer deaths are modelled from age 47 onwards. Different than other cancer types, the best fitted model is achieved when both age and year are numerical variables. We have different powers of age (up to 3) and year (up to 2) in the model. Covariates regressed on the location parameter are explicitly shown as follows:

$$\mu_{a,t,r} = \beta_0 + \beta_1 a + \beta_2 t + \beta_3 \text{AAD} + \beta_4 a^2 + \beta_5 a^3 + \beta_6 t^2 + \beta_7 a t + \beta_8 a^2 t.$$

Note that interaction terms have only occurred between age and year, i.e. $\beta_7 a t$ and $\beta_8 a^2 t$. More importantly, deprivation is not found as a considerable covariate to explain distributional changes in prostate cancer mortality rates. Last but not least, AAD covariate has behaved as a substitute for region covariate as soon as it is considered in the variable selection.

Model for breast cancer mortality rates The modelling for breast cancer deaths is done from age 37 onwards.

$$\mu_{a,t,r} = \beta_0 + \beta_{1,a} + \beta_2 t + \beta_3 \text{AAD} + \beta_{4,r} + \beta_{5,a} t.$$

Here age is a categorical variable with levels $a = 1, \ldots, 11$ where a maps to $\{37,\ 42,\ \ldots,\ 90\}$, respectively. Although the best fitted model is obtained when age is categorical and year is numerical, as well, deprivation is not found statistically significant to explain the changes in breast cancer mortality rates.

2 Relative differences

We quantify the deprivation gap over years in each region using relative differences, shown by $\text{RD}_{t,r}$, along with absolute differences and overall-temporal-changes. The calculation is based on

the difference between the highest- and lowest-incidence/mortality decile in each region, separately for different types of cancer and gender, i.e.

$$\mathrm{RD}_{t,r} = \frac{\hat{\theta}_{t,r}^{\max} - \hat{\theta}_{t,r}^{\min}}{\hat{\theta}_{t,r}^{\min}},$$

where $\hat{\theta}_{t,r}^{\max}$ is the highest fitted rate in year t and region r across all deprivation deciles, and $\hat{\theta}_{t,r}^{\min}$ is the relevant lowest fitted rate in the same year and region.

3 Average age-at-diagnosis

Average age-at-diagnosis is considered as a numerical variable in mortality modelling. In a similar manner with other numerical variables, it is standardised to facilitate convergence. It is used after being standardised either to have a zero mean and unit variance (for all-cancer mortality, and lung cancer mortality), or by subtracting the relevant mean (for bowel cancer mortality, and breast cancer mortality).

Our analysis highlights the effect of (average) age-at-diagnosis on type-specific cancer mortality. Let us consider a real-term increase, denoted by c^*, in the original average age-at-diagnosis, i.e. AAD_1, in a given year such that

$$\mathrm{AAD}_2 = \mathrm{AAD}_1 + c^*,$$

which can be written after standardisation, e.g. mean-centring and unit-scaling, as

$$\begin{aligned}
\mathrm{AAD}_2^{\mathrm{std}} &= \frac{(\mathrm{AAD}_1 + c^*) - \overline{\mathrm{AAD}}_1}{\mathrm{sd}(\mathrm{AAD}_1)} \\
&= \frac{(\mathrm{AAD}_1 - \overline{\mathrm{AAD}}_1)}{\mathrm{sd}(\mathrm{AAD}_1)} + \frac{c^*}{\mathrm{sd}(\mathrm{AAD}_1)} \\
&= \mathrm{AAD}_1^{\mathrm{std}} + \frac{c^*}{\mathrm{sd}(\mathrm{AAD}_1)},
\end{aligned}$$

where $\overline{\mathrm{AAD}}_1$ and $\mathrm{sd}(\mathrm{AAD}_1)$ show the relevant mean and standard deviation for AAD_1. Let us also assume that average age-at-diagnosis has an interaction with region covariate in the model under inspection. The change in fitted mortality rates, shown by $\hat{\theta}_1$ and $\hat{\theta}_2$ (before and after the increase c^*, respectively), is derived by using mean of lognormal distribution as follows:

$$\frac{\hat{\theta}_2}{\hat{\theta}_1} = \exp\left(\hat{\boldsymbol{\beta}} \frac{c^*}{\mathrm{sd}(\mathrm{AAD}_1)}\right),$$

where $\hat{\boldsymbol{\beta}}$ presents all parameter estimates associated with average age-at-diagnosis. Moreover, the relevant fitted mortality rates can be explicitly written as

$$\begin{aligned}
\log \hat{\theta}_1 &= \beta_0 + \beta_1 \mathrm{AAD}_1^{\mathrm{std}} + \beta_{2,r} \mathrm{AAD}_1^{\mathrm{std}} + \dots \\
\log \hat{\theta}_2 &= \beta_0 + \beta_1 \mathrm{AAD}_2^{\mathrm{std}} + \beta_{2,r} \mathrm{AAD}_2^{\mathrm{std}} + \dots \\
&= \beta_0 + \beta_1 \left(\mathrm{AAD}_1^{\mathrm{std}} + \frac{c^*}{\mathrm{sd}(\mathrm{AAD}_1)}\right) + \beta_{2,r} \left(\mathrm{AAD}_1^{\mathrm{std}} + \frac{c^*}{\mathrm{sd}(\mathrm{AAD}_1)}\right) + \dots \\
&= (\beta_1 + \beta_{2,r}) \left(\frac{c^*}{\mathrm{sd}(\mathrm{AAD}_1)}\right) + \log \hat{\theta}_1 \\
&= \hat{\boldsymbol{\beta}} \left(\frac{c^*}{\mathrm{sd}(\mathrm{AAD}_1)}\right) + \log \hat{\theta}_1.
\end{aligned}$$

References

[1] ONS. Cancer Registration Statistics, England, 2013. Office for National Statistics; 2015.

[2] ONS. Cancer Registration Statistics, England, 2015. Office for National Statistics; 2017.

[3] ONS. Cancer Registration Statistics, England, 2016. Office for National Statistics; 2018.

[4] CRUK. Cancer Incidence by age; 2018. Available from: https://www.cancerresearchuk.org/health-professional/cancer-statistics/incidence/age#heading-Two.

S1 Table. Estimated coefficients for the best fitting model for male all-cancer morbidity.

Covariate	Parameter	Mean	SD	%2.5	%97.5
Intercept	β_0	-5.4330	0.0023	-5.4370	-5.4290
Age	β_{1,age_1}	-2.6190	0.0162	-2.6500	-2.5860
	β_{1,age_2}	-2.2120	0.0123	-2.2350	-2.1860
	β_{1,age_3}	-1.9860	0.0111	-2.0080	-1.9660
	β_{1,age_4}	-1.7180	0.0097	-1.7360	-1.6980
	β_{1,age_5}	-1.3270	0.0074	-1.3410	-1.3130
	β_{1,age_6}	-0.8144	0.0058	-0.8259	-0.8034
	β_{1,age_7}	-0.1887	0.0051	-0.1980	-0.1787
	β_{1,age_8}	0.4429	0.0039	0.4356	0.4511
	β_{1,age_9}	0.9648	0.0038	0.9575	0.9718
	$\beta_{1,\text{age}_{10}}$	1.3920	0.0034	1.3850	1.3990
	$\beta_{1,\text{age}_{11}}$	1.6760	0.0035	1.6690	1.6820
	$\beta_{1,\text{age}_{12}}$	1.8970	0.0037	1.8900	1.9040
	$\beta_{1,\text{age}_{13}}$	2.0370	0.0038	2.0290	2.0440
	$\beta_{1,\text{age}_{14}}$	2.4570	0.0040	2.4490	2.4640
Year	β_2	0.0573	0.0024	0.0524	0.0619
Year^2	β_3	-0.0083	0.0017	-0.0113	-0.0052
Year^3	β_4	-0.0130	0.0010	-0.0150	-0.0111
Region	$\beta_{5,\text{region}_1}$	0.0208	0.0043	0.0129	0.0295
	$\beta_{5,\text{region}_2}$	0.0361	0.0029	0.0304	0.0416
	$\beta_{5,\text{region}_3}$	0.0231	0.0032	0.0165	0.0291
	$\beta_{5,\text{region}_4}$	-0.0050	0.0035	-0.0117	0.0019
	$\beta_{5,\text{region}_5}$	-0.0265	0.0033	-0.0330	-0.0199
	$\beta_{5,\text{region}_6}$	-0.0185	0.0033	-0.0252	-0.0119
	$\beta_{5,\text{region}_7}$	-0.0398	0.0031	-0.0462	-0.0336
	$\beta_{5,\text{region}_8}$	-0.0176	0.0029	-0.0234	-0.0121
	$\beta_{5,\text{region}_9}$	0.0273	0.0031	0.0213	0.0331
Deprivation	$\beta_{6,\text{deprivation}_1}$	0.1162	0.0039	0.1082	0.1238
	$\beta_{6,\text{deprivation}_2}$	0.0675	0.0040	0.0595	0.0752
	$\beta_{6,\text{deprivation}_3}$	0.0169	0.0041	0.0086	0.0248
	$\beta_{6,\text{deprivation}_4}$	0.0048	0.0042	-0.0036	0.0133
	$\beta_{6,\text{deprivation}_5}$	-0.0100	0.0039	-0.0172	-0.0017
	$\beta_{6,\text{deprivation}_6}$	-0.0245	0.0046	-0.0338	-0.0157
	$\beta_{6,\text{deprivation}_7}$	-0.0286	0.0042	-0.0364	-0.0198
	$\beta_{6,\text{deprivation}_8}$	-0.0392	0.0043	-0.0481	-0.0311
	$\beta_{6,\text{deprivation}_9}$	-0.0394	0.0042	-0.0487	-0.0312
	$\beta_{6,\text{deprivation}_{10}}$	-0.0638	0.0040	-0.0720	-0.0556
Age:Deprivation	$\beta_{7,\text{age}_1,\text{deprivation}_1}$	-0.0989	0.0290	-0.1625	-0.0468
	$\beta_{7,\text{age}_2,\text{deprivation}_1}$	-0.1370	0.0243	-0.1837	-0.0918
	$\beta_{7,\text{age}_3,\text{deprivation}_1}$	-0.1047	0.0198	-0.1435	-0.0633
	$\beta_{7,\text{age}_4,\text{deprivation}_1}$	-0.0534	0.0186	-0.0899	-0.0177
	$\beta_{7,\text{age}_5,\text{deprivation}_1}$	0.0492	0.0156	0.0191	0.0805
	$\beta_{7,\text{age}_6,\text{deprivation}_1}$	0.0999	0.0122	0.0756	0.1229
	$\beta_{7,\text{age}_7,\text{deprivation}_1}$	0.0957	0.0104	0.0758	0.1170
	$\beta_{7,\text{age}_8,\text{deprivation}_1}$	0.1042	0.0082	0.0881	0.1209
	$\beta_{7,\text{age}_9,\text{deprivation}_1}$	0.0714	0.0074	0.0570	0.0864
	$\beta_{7,\text{age}_{10},\text{deprivation}_1}$	0.0351	0.0070	0.0211	0.0487
	$\beta_{7,\text{age}_{11},\text{deprivation}_1}$	0.0292	0.0067	0.0162	0.0425
	$\beta_{7,\text{age}_{12},\text{deprivation}_1}$	-0.0006	0.0069	-0.0143	0.0132
	$\beta_{7,\text{age}_{13},\text{deprivation}_1}$	-0.0252	0.0076	-0.0403	-0.0102
	$\beta_{7,\text{age}_{14},\text{deprivation}_1}$	-0.0649	0.0085	-0.0815	-0.0484
	$\beta_{7,\text{age}_1,\text{deprivation}_2}$	-0.0859	0.0252	-0.1346	-0.0341
	$\beta_{7,\text{age}_2,\text{deprivation}_2}$	-0.1152	0.0266	-0.1702	-0.0638
	$\beta_{7,\text{age}_3,\text{deprivation}_2}$	-0.1041	0.0196	-0.1455	-0.0668
	$\beta_{7,\text{age}_4,\text{deprivation}_2}$	-0.0492	0.0180	-0.0853	-0.0160
	$\beta_{7,\text{age}_5,\text{deprivation}_2}$	0.0149	0.0143	-0.0148	0.0415
Age:Deprivation	$\beta_{7,\text{age}_6,\text{deprivation}_2}$	0.0866	0.0122	0.0642	0.1116
	$\beta_{7,\text{age}_7,\text{deprivation}_2}$	0.0889	0.0097	0.0703	0.1074
	$\beta_{7,\text{age}_8,\text{deprivation}_2}$	0.0738	0.0086	0.0568	0.0909
	$\beta_{7,\text{age}_9,\text{deprivation}_2}$	0.0539	0.0074	0.0389	0.0678
	$\beta_{7,\text{age}_{10},\text{deprivation}_2}$	0.0285	0.0070	0.0146	0.0422
	$\beta_{7,\text{age}_{11},\text{deprivation}_2}$	0.0231	0.0068	0.0098	0.0362
	$\beta_{7,\text{age}_{12},\text{deprivation}_2}$	0.0176	0.0069	0.0040	0.0308
	$\beta_{7,\text{age}_{13},\text{deprivation}_2}$	-0.0036	0.0074	-0.0177	0.0112
	$\beta_{7,\text{age}_{14},\text{deprivation}_2}$	-0.0293	0.0081	-0.0446	-0.0133
	$\beta_{7,\text{age}_1,\text{deprivation}_3}$	-0.0954	0.0272	-0.1479	-0.0425
	$\beta_{7,\text{age}_2,\text{deprivation}_3}$	-0.1095	0.0234	-0.1557	-0.0661
	$\beta_{7,\text{age}_3,\text{deprivation}_3}$	-0.0758	0.0212	-0.1212	-0.0366
	$\beta_{7,\text{age}_4,\text{deprivation}_3}$	-0.0422	0.0182	-0.0789	-0.0080
	$\beta_{7,\text{age}_5,\text{deprivation}_3}$	-0.0138	0.0149	-0.0418	0.0153
	$\beta_{7,\text{age}_6,\text{deprivation}_3}$	0.0436	0.0128	0.0184	0.0678
	$\beta_{7,\text{age}_7,\text{deprivation}_3}$	0.0718	0.0099	0.0527	0.0910
	$\beta_{7,\text{age}_8,\text{deprivation}_3}$	0.0438	0.0087	0.0267	0.0604
	$\beta_{7,\text{age}_9,\text{deprivation}_3}$	0.0364	0.0076	0.0214	0.0512
	$\beta_{7,\text{age}_{10},\text{deprivation}_3}$	0.0353	0.0069	0.0220	0.0495
	$\beta_{7,\text{age}_{11},\text{deprivation}_3}$	0.0394	0.0067	0.0266	0.0525
	$\beta_{7,\text{age}_{12},\text{deprivation}_3}$	0.0368	0.0072	0.0229	0.0510
	$\beta_{7,\text{age}_{13},\text{deprivation}_3}$	0.0244	0.0077	0.0092	0.0402
	$\beta_{7,\text{age}_{14},\text{deprivation}_3}$	0.0053	0.0082	-0.0115	0.0213
	$\beta_{7,\text{age}_1,\text{deprivation}_4}$	-0.0712	0.0321	-0.1399	-0.0125
	$\beta_{7,\text{age}_2,\text{deprivation}_4}$	-0.0196	0.0201	-0.0576	0.0189
	$\beta_{7,\text{age}_3,\text{deprivation}_4}$	-0.0386	0.0225	-0.0816	0.0072
	$\beta_{7,\text{age}_4,\text{deprivation}_4}$	-0.0258	0.0192	-0.0623	0.0104
	$\beta_{7,\text{age}_5,\text{deprivation}_4}$	-0.0028	0.0143	-0.0311	0.0244
	$\beta_{7,\text{age}_6,\text{deprivation}_4}$	0.0412	0.0126	0.0175	0.0661
	$\beta_{7,\text{age}_7,\text{deprivation}_4}$	0.0290	0.0101	0.0103	0.0487
	$\beta_{7,\text{age}_8,\text{deprivation}_4}$	0.0205	0.0084	0.0040	0.0374
	$\beta_{7,\text{age}_9,\text{deprivation}_4}$	0.0206	0.0073	0.0058	0.0350
	$\beta_{7,\text{age}_{10},\text{deprivation}_4}$	0.0116	0.0068	-0.0017	0.0244
	$\beta_{7,\text{age}_{11},\text{deprivation}_4}$	0.0135	0.0069	0.0000	0.0270
	$\beta_{7,\text{age}_{12},\text{deprivation}_4}$	0.0193	0.0068	0.0061	0.0335
	$\beta_{7,\text{age}_{13},\text{deprivation}_4}$	0.0077	0.0076	-0.0063	0.0226
	$\beta_{7,\text{age}_{14},\text{deprivation}_4}$	-0.0053	0.0081	-0.0214	0.0108
	$\beta_{7,\text{age}_1,\text{deprivation}_5}$	0.0109	0.0269	-0.0373	0.0674
	$\beta_{7,\text{age}_2,\text{deprivation}_5}$	0.0046	0.0217	-0.0432	0.0424
	$\beta_{7,\text{age}_3,\text{deprivation}_5}$	-0.0202	0.0214	-0.0669	0.0201
	$\beta_{7,\text{age}_4,\text{deprivation}_5}$	0.0010	0.0191	-0.0356	0.0403
	$\beta_{7,\text{age}_5,\text{deprivation}_5}$	-0.0099	0.0146	-0.0412	0.0178
	$\beta_{7,\text{age}_6,\text{deprivation}_5}$	0.0000	0.0117	-0.0238	0.0239
	$\beta_{7,\text{age}_7,\text{deprivation}_5}$	-0.0051	0.0103	-0.0253	0.0147
	$\beta_{7,\text{age}_8,\text{deprivation}_5}$	-0.0107	0.0081	-0.0268	0.0047
	$\beta_{7,\text{age}_9,\text{deprivation}_5}$	0.0023	0.0073	-0.0127	0.0164
	$\beta_{7,\text{age}_{10},\text{deprivation}_5}$	0.0017	0.0067	-0.0117	0.0147
	$\beta_{7,\text{age}_{11},\text{deprivation}_5}$	0.0037	0.0065	-0.0097	0.0158
	$\beta_{7,\text{age}_{12},\text{deprivation}_5}$	0.0017	0.0065	-0.0112	0.0141
	$\beta_{7,\text{age}_{13},\text{deprivation}_5}$	0.0114	0.0072	-0.0027	0.0262
	$\beta_{7,\text{age}_{14},\text{deprivation}_5}$	0.0088	0.0077	-0.0062	0.0239
	$\beta_{7,\text{age}_1,\text{deprivation}_6}$	0.0232	0.0308	-0.0365	0.0865
	$\beta_{7,\text{age}_2,\text{deprivation}_6}$	-0.0336	0.0245	-0.0827	0.0131
	$\beta_{7,\text{age}_3,\text{deprivation}_6}$	0.0012	0.0235	-0.0456	0.0439
	$\beta_{7,\text{age}_4,\text{deprivation}_6}$	0.0256	0.0188	-0.0092	0.0637
	$\beta_{7,\text{age}_5,\text{deprivation}_6}$	0.0081	0.0164	-0.0246	0.0379

Covariate	Parameter	Mean	SD	%2.5	%97.5	Covariate	Parameter	Mean	SD	%2.5	%97.5
Age:Deprivation	$\beta_{7,\text{age}_6,\text{deprivation}_6}$	-0.0228	0.0137	-0.0485	0.0029		$\beta_{7,\text{age}_6,\text{deprivation}_{10}}$	-0.0829	0.0131	-0.1078	-0.0571
	$\beta_{7,\text{age}_7,\text{deprivation}_6}$	-0.0173	0.0110	-0.0385	0.0042		$\beta_{7,\text{age}_7,\text{deprivation}_{10}}$	-0.0619	0.0103	-0.0810	-0.0401
	$\beta_{7,\text{age}_8,\text{deprivation}_6}$	-0.0167	0.0083	-0.0326	-0.0001		$\beta_{7,\text{age}_8,\text{deprivation}_{10}}$	-0.0582	0.0083	-0.0755	-0.0421
	$\beta_{7,\text{age}_9,\text{deprivation}_6}$	-0.0133	0.0074	-0.0273	0.0020		$\beta_{7,\text{age}_9,\text{deprivation}_{10}}$	-0.0515	0.0072	-0.0658	-0.0372
	$\beta_{7,\text{age}_{10},\text{deprivation}_6}$	-0.0049	0.0073	-0.0186	0.0104		$\beta_{7,\text{age}_{10},\text{deprivation}_{10}}$	-0.0345	0.0069	-0.0484	-0.0212
	$\beta_{7,\text{age}_{11},\text{deprivation}_6}$	0.0021	0.0071	-0.0118	0.0165		$\beta_{7,\text{age}_{11},\text{deprivation}_{10}}$	-0.0301	0.0070	-0.0443	-0.0169
	$\beta_{7,\text{age}_{12},\text{deprivation}_6}$	0.0081	0.0071	-0.0051	0.0219		$\beta_{7,\text{age}_{12},\text{deprivation}_{10}}$	-0.0421	0.0069	-0.0553	-0.0285
	$\beta_{7,\text{age}_{13},\text{deprivation}_6}$	0.0162	0.0073	0.0017	0.0312		$\beta_{7,\text{age}_{13},\text{deprivation}_{10}}$	-0.0262	0.0073	-0.0408	-0.0120
	$\beta_{7,\text{age}_{14},\text{deprivation}_6}$	0.0239	0.0083	0.0077	0.0404		$\beta_{7,\text{age}_{14},\text{deprivation}_{10}}$	0.0158	0.0074	0.0004	0.0301
	$\beta_{7,\text{age}_1,\text{deprivation}_7}$	0.0679	0.0317	0.0029	0.1312	Age:Year	β_{8,age_1}	-0.0288	0.0100	-0.0471	-0.0083
	$\beta_{7,\text{age}_2,\text{deprivation}_7}$	0.0747	0.0263	0.0223	0.1197		β_{8,age_2}	0.0336	0.0095	0.0160	0.0527
	$\beta_{7,\text{age}_3,\text{deprivation}_7}$	0.0365	0.0237	-0.0067	0.0873		β_{8,age_3}	0.0472	0.0073	0.0336	0.0622
	$\beta_{7,\text{age}_4,\text{deprivation}_7}$	0.0178	0.0190	-0.0202	0.0544		β_{8,age_4}	0.0355	0.0065	0.0226	0.0478
	$\beta_{7,\text{age}_5,\text{deprivation}_7}$	0.0058	0.0156	-0.0242	0.0371		β_{8,age_5}	0.0214	0.0054	0.0105	0.0316
	$\beta_{7,\text{age}_6,\text{deprivation}_7}$	-0.0529	0.0138	-0.0789	-0.0252		β_{8,age_6}	0.0211	0.0045	0.0125	0.0300
	$\beta_{7,\text{age}_7,\text{deprivation}_7}$	-0.0476	0.0102	-0.0684	-0.0279		β_{8,age_7}	0.0079	0.0036	0.0008	0.0150
	$\beta_{7,\text{age}_8,\text{deprivation}_7}$	-0.0437	0.0086	-0.0602	-0.0262		β_{8,age_8}	0.0050	0.0029	-0.0008	0.0109
	$\beta_{7,\text{age}_9,\text{deprivation}_7}$	-0.0337	0.0076	-0.0493	-0.0193		β_{8,age_9}	0.0006	0.0026	-0.0047	0.0056
	$\beta_{7,\text{age}_{10},\text{deprivation}_7}$	-0.0196	0.0066	-0.0331	-0.0068		$\beta_{8,\text{age}_{10}}$	-0.0093	0.0024	-0.0139	-0.0047
	$\beta_{7,\text{age}_{11},\text{deprivation}_7}$	-0.0257	0.0067	-0.0387	-0.0131		$\beta_{8,\text{age}_{11}}$	-0.0157	0.0024	-0.0202	-0.0108
	$\beta_{7,\text{age}_{12},\text{deprivation}_7}$	-0.0062	0.0068	-0.0193	0.0065		$\beta_{8,\text{age}_{12}}$	-0.0352	0.0023	-0.0396	-0.0305
	$\beta_{7,\text{age}_{13},\text{deprivation}_7}$	0.0050	0.0074	-0.0090	0.0196		$\beta_{8,\text{age}_{13}}$	-0.0431	0.0024	-0.0478	-0.0383
	$\beta_{7,\text{age}_{14},\text{deprivation}_7}$	0.0218	0.0078	0.0059	0.0372		$\beta_{8,\text{age}_{14}}$	-0.0402	0.0029	-0.0456	-0.0345
	$\beta_{7,\text{age}_1,\text{deprivation}_8}$	0.0793	0.0268	0.0303	0.1339	Region:Year	$\beta_{9,\text{region}_1}$	-0.0201	0.0029	-0.0255	-0.0143
	$\beta_{7,\text{age}_2,\text{deprivation}_8}$	0.0976	0.0279	0.0429	0.1487		$\beta_{9,\text{region}_2}$	-0.0012	0.0020	-0.0052	0.0025
	$\beta_{7,\text{age}_3,\text{deprivation}_8}$	0.0694	0.0192	0.0320	0.1085		$\beta_{9,\text{region}_3}$	-0.0082	0.0022	-0.0127	-0.0039
	$\beta_{7,\text{age}_4,\text{deprivation}_8}$	0.0263	0.0198	-0.0171	0.0625		$\beta_{9,\text{region}_4}$	0.0064	0.0023	0.0018	0.0109
	$\beta_{7,\text{age}_5,\text{deprivation}_8}$	-0.0335	0.0149	-0.0620	-0.0061		$\beta_{9,\text{region}_5}$	-0.0004	0.0022	-0.0046	0.0037
	$\beta_{7,\text{age}_6,\text{deprivation}_8}$	-0.0456	0.0123	-0.0686	-0.0214		$\beta_{9,\text{region}_6}$	0.0164	0.0021	0.0124	0.0206
	$\beta_{7,\text{age}_7,\text{deprivation}_8}$	-0.0719	0.0102	-0.0912	-0.0529		$\beta_{9,\text{region}_7}$	0.0003	0.0022	-0.0038	0.0048
	$\beta_{7,\text{age}_8,\text{deprivation}_8}$	-0.0593	0.0080	-0.0745	-0.0426		$\beta_{9,\text{region}_8}$	0.0142	0.0019	0.0103	0.0180
	$\beta_{7,\text{age}_9,\text{deprivation}_8}$	-0.0367	0.0071	-0.0507	-0.0229		$\beta_{9,\text{region}_9}$	-0.0074	0.0021	-0.0114	-0.0032
	$\beta_{7,\text{age}_{10},\text{deprivation}_8}$	-0.0182	0.0068	-0.0315	-0.0051	Age : Year^2	β_{10,age_1}	0.0071	0.0114	-0.0154	0.0283
	$\beta_{7,\text{age}_{11},\text{deprivation}_8}$	-0.0235	0.0065	-0.0363	-0.0106		β_{10,age_2}	-0.0289	0.0087	-0.0445	-0.0123
	$\beta_{7,\text{age}_{12},\text{deprivation}_8}$	-0.0084	0.0070	-0.0216	0.0051		β_{10,age_3}	0.0106	0.0079	-0.0044	0.0266
	$\beta_{7,\text{age}_{13},\text{deprivation}_8}$	0.0083	0.0071	-0.0052	0.0220		β_{10,age_4}	-0.0033	0.0068	-0.0170	0.0084
	$\beta_{7,\text{age}_{14},\text{deprivation}_8}$	0.0161	0.0074	0.0014	0.0311		β_{10,age_5}	0.0030	0.0058	-0.0081	0.0143
	$\beta_{7,\text{age}_1,\text{deprivation}_9}$	0.0992	0.0307	0.0338	0.1541		β_{10,age_6}	0.0180	0.0044	0.0095	0.0265
	$\beta_{7,\text{age}_2,\text{deprivation}_9}$	0.0935	0.0242	0.0466	0.1478		β_{10,age_7}	0.0025	0.0037	-0.0052	0.0095
	$\beta_{7,\text{age}_3,\text{deprivation}_9}$	0.1275	0.0238	0.0810	0.1719		β_{10,age_8}	-0.0075	0.0030	-0.0135	-0.0018
	$\beta_{7,\text{age}_4,\text{deprivation}_9}$	0.0283	0.0200	-0.0119	0.0671		β_{10,age_9}	-0.0085	0.0028	-0.0139	-0.0030
	$\beta_{7,\text{age}_5,\text{deprivation}_9}$	0.0063	0.0171	-0.0276	0.0373		$\beta_{10,\text{age}_{10}}$	-0.0119	0.0025	-0.0169	-0.0068
	$\beta_{7,\text{age}_6,\text{deprivation}_9}$	-0.0671	0.0142	-0.0925	-0.0344		$\beta_{10,\text{age}_{11}}$	-0.0008	0.0026	-0.0057	0.0043
	$\beta_{7,\text{age}_7,\text{deprivation}_9}$	-0.0817	0.0105	-0.1027	-0.0600		$\beta_{10,\text{age}_{12}}$	0.0116	0.0026	0.0063	0.0168
	$\beta_{7,\text{age}_8,\text{deprivation}_9}$	-0.0537	0.0082	-0.0700	-0.0380		$\beta_{10,\text{age}_{13}}$	-0.0063	0.0029	-0.0120	-0.0006
	$\beta_{7,\text{age}_9,\text{deprivation}_9}$	-0.0495	0.0076	-0.0640	-0.0351		$\beta_{10,\text{age}_{14}}$	0.0145	0.0031	0.0088	0.0209
	$\beta_{7,\text{age}_{10},\text{deprivation}_9}$	-0.0349	0.0068	-0.0486	-0.0217	Region : Year^2	$\beta_{11,\text{region}_1}$	0.0147	0.0032	0.0080	0.0208
	$\beta_{7,\text{age}_{11},\text{deprivation}_9}$	-0.0316	0.0065	-0.0441	-0.0184		$\beta_{11,\text{region}_2}$	-0.0062	0.0023	-0.0104	-0.0015
	$\beta_{7,\text{age}_{12},\text{deprivation}_9}$	-0.0262	0.0068	-0.0396	-0.0127		$\beta_{11,\text{region}_3}$	-0.0045	0.0023	-0.0090	0.0000
	$\beta_{7,\text{age}_{13},\text{deprivation}_9}$	-0.0180	0.0073	-0.0322	-0.0031		$\beta_{11,\text{region}_4}$	-0.0137	0.0027	-0.0189	-0.0083
	$\beta_{7,\text{age}_{14},\text{deprivation}_9}$	0.0077	0.0078	-0.0081	0.0227		$\beta_{11,\text{region}_5}$	0.0029	0.0025	-0.0020	0.0080
	$\beta_{7,\text{age}_1,\text{deprivation}_{10}}$	0.0710	0.0242	0.0205	0.1179		$\beta_{11,\text{region}_6}$	-0.0008	0.0024	-0.0055	0.0043
	$\beta_{7,\text{age}_2,\text{deprivation}_{10}}$	0.1444	0.0263	0.0898	0.1946		$\beta_{11,\text{region}_7}$	-0.0035	0.0023	-0.0081	0.0010
	$\beta_{7,\text{age}_3,\text{deprivation}_{10}}$	0.1087	0.0278	0.0545	0.1635		$\beta_{11,\text{region}_8}$	0.0132	0.0022	0.0091	0.0179
	$\beta_{7,\text{age}_4,\text{deprivation}_{10}}$	0.0716	0.0207	0.0332	0.1136		$\beta_{11,\text{region}_9}$	-0.0020	0.0024	-0.0064	0.0029
	$\beta_{7,\text{age}_5,\text{deprivation}_{10}}$	-0.0241	0.0157	-0.0558	0.0076		σ^2	0.0011	0.0001	0.0009	0.0013

S2 Table. Estimated coefficients for the best fitting model for female all-cancer morbidity.

Covariate	Parameter	Mean	SD	%2.5	%97.5
Intercept	β_0	-5.3520	0.0019	-5.3560	-5.3490
Age	β_{1,age_1}	-2.7030	0.0141	-2.7340	-2.6770
	β_{1,age_2}	-1.9440	0.0117	-1.9640	-1.9190
	β_{1,age_3}	-1.5290	0.0084	-1.5440	-1.5120
	β_{1,age_4}	-1.1110	0.0072	-1.1250	-1.0970
	β_{1,age_5}	-0.6428	0.0061	-0.6544	-0.6302
	β_{1,age_6}	-0.2125	0.0051	-0.2222	-0.2018
	β_{1,age_7}	0.1657	0.0047	0.1564	0.1747
	β_{1,age_8}	0.4203	0.0039	0.4127	0.4283
	β_{1,age_9}	0.7384	0.0038	0.7309	0.7458
	$\beta_{1,\text{age}_{10}}$	0.9852	0.0036	0.9781	0.9925
	$\beta_{1,\text{age}_{11}}$	1.1070	0.0036	1.1000	1.1130
	$\beta_{1,\text{age}_{12}}$	1.3080	0.0036	1.3010	1.3160
	$\beta_{1,\text{age}_{13}}$	1.4570	0.0039	1.4490	1.4650
	$\beta_{1,\text{age}_{14}}$	1.9610	0.0036	1.9540	1.9680
Year	β_2	0.0732	0.0021	0.0690	0.0771
Year^2	β_3	-0.0068	0.0013	-0.0093	-0.0041
Year^3	β_4	-0.0085	0.0010	-0.0104	-0.0066
Region	$\beta_{5,\text{region}_1}$	0.0595	0.0049	0.0497	0.0688
	$\beta_{5,\text{region}_2}$	0.0389	0.0030	0.0327	0.0445
	$\beta_{5,\text{region}_3}$	0.0236	0.0038	0.0160	0.0305
	$\beta_{5,\text{region}_4}$	0.0131	0.0040	0.0051	0.0211
	$\beta_{5,\text{region}_5}$	-0.0353	0.0038	-0.0427	-0.0274
	$\beta_{5,\text{region}_6}$	-0.0360	0.0034	-0.0428	-0.0294
	$\beta_{5,\text{region}_7}$	-0.0983	0.0030	-0.1043	-0.0923
	$\beta_{5,\text{region}_8}$	-0.0109	0.0031	-0.0169	-0.0048
	$\beta_{5,\text{region}_9}$	0.0454	0.0037	0.0383	0.0521
Deprivation	$\beta_{6,\text{deprivation}_1}$	0.0558	0.0036	0.0485	0.0626
	$\beta_{6,\text{deprivation}_2}$	0.0314	0.0033	0.0250	0.0381
	$\beta_{6,\text{deprivation}_3}$	0.0165	0.0034	0.0099	0.0234
	$\beta_{6,\text{deprivation}_4}$	0.0068	0.0036	-0.0005	0.0137
	$\beta_{6,\text{deprivation}_5}$	-0.0052	0.0037	-0.0125	0.0015
	$\beta_{6,\text{deprivation}_6}$	-0.0146	0.0040	-0.0230	-0.0079
	$\beta_{6,\text{deprivation}_7}$	-0.0096	0.0042	-0.0178	-0.0013
	$\beta_{6,\text{deprivation}_8}$	-0.0225	0.0041	-0.0306	-0.0143
	$\beta_{6,\text{deprivation}_9}$	-0.0239	0.0041	-0.0314	-0.0158
	$\beta_{6,\text{deprivation}_{10}}$	-0.0348	0.0039	-0.0424	-0.0275
Age:Deprivation	$\beta_{7,\text{age}_1,\text{deprivation}_1}$	-0.0112	0.0287	-0.0719	0.0443
	$\beta_{7,\text{age}_2,\text{deprivation}_1}$	-0.0931	0.0188	-0.1313	-0.0576
	$\beta_{7,\text{age}_3,\text{deprivation}_1}$	-0.1126	0.0170	-0.1476	-0.0806
	$\beta_{7,\text{age}_4,\text{deprivation}_1}$	-0.0728	0.0140	-0.1003	-0.0444
	$\beta_{7,\text{age}_5,\text{deprivation}_1}$	-0.0678	0.0115	-0.0900	-0.0445
	$\beta_{7,\text{age}_6,\text{deprivation}_1}$	-0.0406	0.0098	-0.0595	-0.0212
	$\beta_{7,\text{age}_7,\text{deprivation}_1}$	-0.0041	0.0090	-0.0216	0.0139
	$\beta_{7,\text{age}_8,\text{deprivation}_1}$	0.0579	0.0084	0.0413	0.0742
	$\beta_{7,\text{age}_9,\text{deprivation}_1}$	0.0760	0.0078	0.0604	0.0918
	$\beta_{7,\text{age}_{10},\text{deprivation}_1}$	0.0851	0.0075	0.0704	0.0995
	$\beta_{7,\text{age}_{11},\text{deprivation}_1}$	0.0932	0.0074	0.0785	0.1076
	$\beta_{7,\text{age}_{12},\text{deprivation}_1}$	0.0630	0.0073	0.0480	0.0767
	$\beta_{7,\text{age}_{13},\text{deprivation}_1}$	0.0284	0.0076	0.0131	0.0439
	$\beta_{7,\text{age}_{14},\text{deprivation}_1}$	-0.0013	0.0075	-0.0161	0.0136
	$\beta_{7,\text{age}_1,\text{deprivation}_2}$	-0.0122	0.0265	-0.0658	0.0364
	$\beta_{7,\text{age}_2,\text{deprivation}_2}$	-0.0702	0.0187	-0.1084	-0.0340
	$\beta_{7,\text{age}_3,\text{deprivation}_2}$	-0.0548	0.0160	-0.0859	-0.0250
	$\beta_{7,\text{age}_4,\text{deprivation}_2}$	-0.0658	0.0121	-0.0888	-0.0417
	$\beta_{7,\text{age}_5,\text{deprivation}_2}$	-0.0280	0.0108	-0.0488	-0.0085
	$\beta_{7,\text{age}_6,\text{deprivation}_2}$	-0.0373	0.0095	-0.0577	-0.0188
Age:Deprivation	$\beta_{7,\text{age}_7,\text{deprivation}_2}$	0.0016	0.0086	-0.0155	0.0181
	$\beta_{7,\text{age}_8,\text{deprivation}_2}$	0.0354	0.0083	0.0193	0.0524
	$\beta_{7,\text{age}_9,\text{deprivation}_2}$	0.0498	0.0071	0.0361	0.0635
	$\beta_{7,\text{age}_{10},\text{deprivation}_2}$	0.0609	0.0072	0.0468	0.0749
	$\beta_{7,\text{age}_{11},\text{deprivation}_2}$	0.0630	0.0069	0.0487	0.0762
	$\beta_{7,\text{age}_{12},\text{deprivation}_2}$	0.0461	0.0067	0.0327	0.0589
	$\beta_{7,\text{age}_{13},\text{deprivation}_2}$	0.0240	0.0072	0.0107	0.0378
	$\beta_{7,\text{age}_{14},\text{deprivation}_2}$	-0.0123	0.0071	-0.0261	0.0015
	$\beta_{7,\text{age}_1,\text{deprivation}_3}$	-0.0347	0.0275	-0.0844	0.0269
	$\beta_{7,\text{age}_2,\text{deprivation}_3}$	-0.0526	0.0199	-0.0914	-0.0090
	$\beta_{7,\text{age}_3,\text{deprivation}_3}$	-0.0498	0.0146	-0.0787	-0.0212
	$\beta_{7,\text{age}_4,\text{deprivation}_3}$	-0.0336	0.0130	-0.0586	-0.0082
	$\beta_{7,\text{age}_5,\text{deprivation}_3}$	-0.0073	0.0111	-0.0303	0.0138
	$\beta_{7,\text{age}_6,\text{deprivation}_3}$	-0.0088	0.0091	-0.0267	0.0092
	$\beta_{7,\text{age}_7,\text{deprivation}_3}$	-0.0011	0.0087	-0.0183	0.0159
	$\beta_{7,\text{age}_8,\text{deprivation}_3}$	0.0236	0.0078	0.0083	0.0385
	$\beta_{7,\text{age}_9,\text{deprivation}_3}$	0.0373	0.0075	0.0231	0.0521
	$\beta_{7,\text{age}_{10},\text{deprivation}_3}$	0.0326	0.0070	0.0192	0.0466
	$\beta_{7,\text{age}_{11},\text{deprivation}_3}$	0.0469	0.0069	0.0335	0.0602
	$\beta_{7,\text{age}_{12},\text{deprivation}_3}$	0.0316	0.0070	0.0169	0.0452
	$\beta_{7,\text{age}_{13},\text{deprivation}_3}$	0.0158	0.0069	0.0022	0.0293
	$\beta_{7,\text{age}_{14},\text{deprivation}_3}$	0.0000	0.0071	-0.0142	0.0136
	$\beta_{7,\text{age}_1,\text{deprivation}_4}$	-0.0139	0.0275	-0.0665	0.0383
	$\beta_{7,\text{age}_2,\text{deprivation}_4}$	-0.0357	0.0186	-0.0717	0.0006
	$\beta_{7,\text{age}_3,\text{deprivation}_4}$	-0.0060	0.0161	-0.0385	0.0237
	$\beta_{7,\text{age}_4,\text{deprivation}_4}$	-0.0119	0.0132	-0.0356	0.0140
	$\beta_{7,\text{age}_5,\text{deprivation}_4}$	-0.0122	0.0111	-0.0343	0.0099
	$\beta_{7,\text{age}_6,\text{deprivation}_4}$	-0.0397	0.0088	-0.0566	-0.0221
	$\beta_{7,\text{age}_7,\text{deprivation}_4}$	0.0048	0.0087	-0.0123	0.0224
	$\beta_{7,\text{age}_8,\text{deprivation}_4}$	0.0141	0.0082	-0.0015	0.0307
	$\beta_{7,\text{age}_9,\text{deprivation}_4}$	0.0137	0.0075	-0.0011	0.0285
	$\beta_{7,\text{age}_{10},\text{deprivation}_4}$	0.0140	0.0068	0.0003	0.0274
	$\beta_{7,\text{age}_{11},\text{deprivation}_4}$	0.0232	0.0071	0.0092	0.0369
	$\beta_{7,\text{age}_{12},\text{deprivation}_4}$	0.0211	0.0069	0.0075	0.0350
	$\beta_{7,\text{age}_{13},\text{deprivation}_4}$	0.0186	0.0068	0.0051	0.0318
	$\beta_{7,\text{age}_{14},\text{deprivation}_4}$	0.0100	0.0068	-0.0035	0.0236
	$\beta_{7,\text{age}_1,\text{deprivation}_5}$	-0.0074	0.0294	-0.0655	0.0439
	$\beta_{7,\text{age}_2,\text{deprivation}_5}$	-0.0390	0.0227	-0.0811	0.0053
	$\beta_{7,\text{age}_3,\text{deprivation}_5}$	-0.0085	0.0157	-0.0405	0.0208
	$\beta_{7,\text{age}_4,\text{deprivation}_5}$	0.0003	0.0125	-0.0254	0.0245
	$\beta_{7,\text{age}_5,\text{deprivation}_5}$	0.0197	0.0100	-0.0010	0.0390
	$\beta_{7,\text{age}_6,\text{deprivation}_5}$	0.0006	0.0098	-0.0193	0.0196
	$\beta_{7,\text{age}_7,\text{deprivation}_5}$	0.0135	0.0082	-0.0028	0.0293
	$\beta_{7,\text{age}_8,\text{deprivation}_5}$	-0.0018	0.0080	-0.0175	0.0145
	$\beta_{7,\text{age}_9,\text{deprivation}_5}$	0.0031	0.0076	-0.0114	0.0177
	$\beta_{7,\text{age}_{10},\text{deprivation}_5}$	0.0194	0.0069	0.0057	0.0327
	$\beta_{7,\text{age}_{11},\text{deprivation}_5}$	-0.0054	0.0072	-0.0201	0.0093
	$\beta_{7,\text{age}_{12},\text{deprivation}_5}$	-0.0053	0.0071	-0.0193	0.0090
	$\beta_{7,\text{age}_{13},\text{deprivation}_5}$	0.0041	0.0069	-0.0094	0.0174
	$\beta_{7,\text{age}_{14},\text{deprivation}_5}$	0.0065	0.0068	-0.0068	0.0193
	$\beta_{7,\text{age}_1,\text{deprivation}_6}$	-0.0592	0.0341	-0.1281	0.0019
	$\beta_{7,\text{age}_2,\text{deprivation}_6}$	0.0177	0.0208	-0.0224	0.0607
	$\beta_{7,\text{age}_3,\text{deprivation}_6}$	0.0091	0.0172	-0.0232	0.0455
	$\beta_{7,\text{age}_4,\text{deprivation}_6}$	0.0187	0.0139	-0.0103	0.0465
	$\beta_{7,\text{age}_5,\text{deprivation}_6}$	0.0216	0.0114	-0.0007	0.0447
	$\beta_{7,\text{age}_6,\text{deprivation}_6}$	0.0140	0.0099	-0.0050	0.0340
	$\beta_{7,\text{age}_7,\text{deprivation}_6}$	-0.0047	0.0086	-0.0210	0.0133

Covariate	Parameter	Mean	SD	%2.5	%97.5	Covariate	Parameter	Mean	SD	%2.5	%97.5
Age:Deprivation	$\beta_{7,\text{age}_8,\text{deprivation}_6}$	-0.0045	0.0080	-0.0190	0.0125		$\beta_{7,\text{age}_9,\text{deprivation}_{10}}$	-0.0499	0.0078	-0.0651	-0.0341
	$\beta_{7,\text{age}_9,\text{deprivation}_6}$	-0.0129	0.0077	-0.0274	0.0022		$\beta_{7,\text{age}_{10},\text{deprivation}_{10}}$	-0.0697	0.0072	-0.0845	-0.0558
	$\beta_{7,\text{age}_{10},\text{deprivation}_6}$	-0.0112	0.0073	-0.0249	0.0038		$\beta_{7,\text{age}_{11},\text{deprivation}_{10}}$	-0.0639	0.0078	-0.0793	-0.0490
	$\beta_{7,\text{age}_{11},\text{deprivation}_6}$	-0.0164	0.0073	-0.0309	-0.0020		$\beta_{7,\text{age}_{12},\text{deprivation}_{10}}$	-0.0594	0.0072	-0.0733	-0.0456
	$\beta_{7,\text{age}_{12},\text{deprivation}_6}$	0.0093	0.0070	-0.0045	0.0233		$\beta_{7,\text{age}_{13},\text{deprivation}_{10}}$	-0.0353	0.0077	-0.0511	-0.0204
	$\beta_{7,\text{age}_{13},\text{deprivation}_6}$	0.0039	0.0072	-0.0098	0.0180		$\beta_{7,\text{age}_{14},\text{deprivation}_{10}}$	-0.0083	0.0074	-0.0236	0.0061
	$\beta_{7,\text{age}_{14},\text{deprivation}_6}$	0.0147	0.0069	0.0012	0.0284	Age:Year	β_{8,age_1}	0.0454	0.0106	0.0249	0.0668
	$\beta_{7,\text{age}_1,\text{deprivation}_7}$	0.0525	0.0333	-0.0098	0.1146		β_{8,age_2}	0.1000	0.0076	0.0852	0.1157
	$\beta_{7,\text{age}_2,\text{deprivation}_7}$	0.0520	0.0221	0.0084	0.0958		β_{8,age_3}	0.0325	0.0055	0.0215	0.0431
	$\beta_{7,\text{age}_3,\text{deprivation}_7}$	0.0190	0.0178	-0.0156	0.0521		β_{8,age_4}	0.0069	0.0046	-0.0019	0.0161
	$\beta_{7,\text{age}_4,\text{deprivation}_7}$	0.0145	0.0143	-0.0131	0.0417		β_{8,age_5}	-0.0023	0.0039	-0.0099	0.0049
	$\beta_{7,\text{age}_5,\text{deprivation}_7}$	0.0149	0.0117	-0.0075	0.0378		β_{8,age_6}	0.0082	0.0033	0.0019	0.0144
	$\beta_{7,\text{age}_6,\text{deprivation}_7}$	0.0189	0.0093	0.0009	0.0370		β_{8,age_7}	-0.0398	0.0029	-0.0455	-0.0343
	$\beta_{7,\text{age}_7,\text{deprivation}_7}$	-0.0039	0.0084	-0.0217	0.0123		β_{8,age_8}	-0.0523	0.0027	-0.0576	-0.0472
	$\beta_{7,\text{age}_8,\text{deprivation}_7}$	-0.0238	0.0079	-0.0390	-0.0085		β_{8,age_9}	-0.0330	0.0025	-0.0379	-0.0280
	$\beta_{7,\text{age}_9,\text{deprivation}_7}$	-0.0343	0.0079	-0.0497	-0.0192		$\beta_{8,\text{age}_{10}}$	-0.0001	0.0024	-0.0049	0.0046
	$\beta_{7,\text{age}_{10},\text{deprivation}_7}$	-0.0245	0.0077	-0.0394	-0.0089		$\beta_{8,\text{age}_{11}}$	-0.0122	0.0025	-0.0170	-0.0072
	$\beta_{7,\text{age}_{11},\text{deprivation}_7}$	-0.0375	0.0074	-0.0519	-0.0237		$\beta_{8,\text{age}_{12}}$	-0.0210	0.0023	-0.0256	-0.0165
	$\beta_{7,\text{age}_{12},\text{deprivation}_7}$	-0.0231	0.0073	-0.0375	-0.0091		$\beta_{8,\text{age}_{13}}$	-0.0127	0.0024	-0.0175	-0.0079
	$\beta_{7,\text{age}_{13},\text{deprivation}_7}$	-0.0108	0.0072	-0.0251	0.0033		$\beta_{8,\text{age}_{14}}$	-0.0196	0.0024	-0.0243	-0.0149
	$\beta_{7,\text{age}_{14},\text{deprivation}_7}$	-0.0139	0.0073	-0.0285	0.0003	Region:Year	$\beta_{9,\text{region}_1}$	0.0006	0.0029	-0.0050	0.0060
	$\beta_{7,\text{age}_1,\text{deprivation}_8}$	0.0772	0.0341	0.0176	0.1493		$\beta_{9,\text{region}_2}$	0.0116	0.0020	0.0077	0.0155
	$\beta_{7,\text{age}_2,\text{deprivation}_8}$	0.0508	0.0227	0.0062	0.0994		$\beta_{9,\text{region}_3}$	-0.0065	0.0022	-0.0106	-0.0023
	$\beta_{7,\text{age}_3,\text{deprivation}_8}$	0.0407	0.0187	0.0053	0.0770		$\beta_{9,\text{region}_4}$	-0.0031	0.0024	-0.0077	0.0016
	$\beta_{7,\text{age}_4,\text{deprivation}_8}$	0.0154	0.0150	-0.0132	0.0448		$\beta_{9,\text{region}_5}$	0.0025	0.0022	-0.0017	0.0067
	$\beta_{7,\text{age}_5,\text{deprivation}_8}$	0.0048	0.0114	-0.0171	0.0263		$\beta_{9,\text{region}_6}$	0.0059	0.0021	0.0017	0.0101
	$\beta_{7,\text{age}_6,\text{deprivation}_8}$	0.0301	0.0101	0.0104	0.0494		$\beta_{9,\text{region}_7}$	-0.0024	0.0021	-0.0067	0.0019
	$\beta_{7,\text{age}_7,\text{deprivation}_8}$	0.0009	0.0087	-0.0159	0.0170		$\beta_{9,\text{region}_8}$	0.0057	0.0019	0.0018	0.0094
	$\beta_{7,\text{age}_8,\text{deprivation}_8}$	-0.0429	0.0081	-0.0592	-0.0272		$\beta_{9,\text{region}_9}$	-0.0142	0.0021	-0.0182	-0.0100
	$\beta_{7,\text{age}_9,\text{deprivation}_8}$	-0.0410	0.0073	-0.0555	-0.0268	Age:Region	$\beta_{10,\text{age}_1,\text{region}_1}$	0.0620	0.0368	-0.0092	0.1355
	$\beta_{7,\text{age}_{10},\text{deprivation}_8}$	-0.0500	0.0073	-0.0642	-0.0353		$\beta_{10,\text{age}_1,\text{region}_2}$	0.0397	0.0256	-0.0144	0.0876
	$\beta_{7,\text{age}_{11},\text{deprivation}_8}$	-0.0370	0.0071	-0.0508	-0.0227		$\beta_{10,\text{age}_1,\text{region}_3}$	-0.0549	0.0292	-0.1146	-0.0020
	$\beta_{7,\text{age}_{12},\text{deprivation}_8}$	-0.0409	0.0073	-0.0557	-0.0267		$\beta_{10,\text{age}_1,\text{region}_4}$	-0.0172	0.0322	-0.0809	0.0437
	$\beta_{7,\text{age}_{13},\text{deprivation}_8}$	-0.0188	0.0073	-0.0334	-0.0042		$\beta_{10,\text{age}_1,\text{region}_5}$	-0.0739	0.0267	-0.1273	-0.0230
	$\beta_{7,\text{age}_{14},\text{deprivation}_8}$	0.0106	0.0075	-0.0050	0.0246		$\beta_{10,\text{age}_1,\text{region}_6}$	0.0108	0.0315	-0.0559	0.0717
	$\beta_{7,\text{age}_1,\text{deprivation}_9}$	0.0420	0.0366	-0.0283	0.1127		$\beta_{10,\text{age}_1,\text{region}_7}$	0.0326	0.0242	-0.0127	0.0848
	$\beta_{7,\text{age}_2,\text{deprivation}_9}$	0.0526	0.0273	0.0052	0.1220		$\beta_{10,\text{age}_1,\text{region}_8}$	-0.0064	0.0247	-0.0560	0.0392
	$\beta_{7,\text{age}_3,\text{deprivation}_9}$	0.0708	0.0184	0.0367	0.1086		$\beta_{10,\text{age}_1,\text{region}_9}$	0.0073	0.0313	-0.0568	0.0651
	$\beta_{7,\text{age}_4,\text{deprivation}_9}$	0.0622	0.0139	0.0370	0.0912		$\beta_{10,\text{age}_2,\text{region}_1}$	0.0581	0.0241	0.0081	0.1007
	$\beta_{7,\text{age}_5,\text{deprivation}_9}$	0.0009	0.0110	-0.0211	0.0224		$\beta_{10,\text{age}_2,\text{region}_2}$	0.0406	0.0158	0.0104	0.0730
	$\beta_{7,\text{age}_6,\text{deprivation}_9}$	0.0516	0.0095	0.0322	0.0701		$\beta_{10,\text{age}_2,\text{region}_3}$	0.0725	0.0199	0.0304	0.1112
	$\beta_{7,\text{age}_7,\text{deprivation}_9}$	-0.0072	0.0083	-0.0230	0.0097		$\beta_{10,\text{age}_2,\text{region}_4}$	0.0517	0.0233	0.0055	0.0985
	$\beta_{7,\text{age}_8,\text{deprivation}_9}$	-0.0301	0.0082	-0.0455	-0.0129		$\beta_{10,\text{age}_2,\text{region}_5}$	0.0097	0.0187	-0.0263	0.0505
	$\beta_{7,\text{age}_9,\text{deprivation}_9}$	-0.0417	0.0074	-0.0557	-0.0272		$\beta_{10,\text{age}_2,\text{region}_6}$	-0.0834	0.0210	-0.1264	-0.0442
	$\beta_{7,\text{age}_{10},\text{deprivation}_9}$	-0.0567	0.0075	-0.0715	-0.0420		$\beta_{10,\text{age}_2,\text{region}_7}$	-0.1612	0.0179	-0.1935	-0.1197
	$\beta_{7,\text{age}_{11},\text{deprivation}_9}$	-0.0661	0.0074	-0.0803	-0.0510		$\beta_{10,\text{age}_2,\text{region}_8}$	-0.0366	0.0180	-0.0724	0.0003
	$\beta_{7,\text{age}_{12},\text{deprivation}_9}$	-0.0423	0.0075	-0.0566	-0.0272		$\beta_{10,\text{age}_2,\text{region}_9}$	0.0487	0.0222	0.0078	0.0957
	$\beta_{7,\text{age}_{13},\text{deprivation}_9}$	-0.0298	0.0077	-0.0449	-0.0146		$\beta_{10,\text{age}_3,\text{region}_1}$	0.0011	0.0217	-0.0414	0.0437
	$\beta_{7,\text{age}_{14},\text{deprivation}_9}$	-0.0059	0.0072	-0.0198	0.0083		$\beta_{10,\text{age}_3,\text{region}_2}$	0.0104	0.0146	-0.0179	0.0392
	$\beta_{7,\text{age}_1,\text{deprivation}_{10}}$	-0.0332	0.0330	-0.1072	0.0254		$\beta_{10,\text{age}_3,\text{region}_3}$	0.0575	0.0167	0.0276	0.0921
	$\beta_{7,\text{age}_2,\text{deprivation}_{10}}$	0.1176	0.0225	0.0694	0.1583		$\beta_{10,\text{age}_3,\text{region}_4}$	0.0276	0.0176	-0.0058	0.0610
	$\beta_{7,\text{age}_3,\text{deprivation}_{10}}$	0.0920	0.0187	0.0555	0.1311		$\beta_{10,\text{age}_3,\text{region}_5}$	0.0254	0.0172	-0.0109	0.0571
	$\beta_{7,\text{age}_4,\text{deprivation}_{10}}$	0.0730	0.0144	0.0452	0.1014		$\beta_{10,\text{age}_3,\text{region}_6}$	-0.0225	0.0163	-0.0539	0.0109
	$\beta_{7,\text{age}_5,\text{deprivation}_{10}}$	0.0534	0.0110	0.0332	0.0753		$\beta_{10,\text{age}_3,\text{region}_7}$	-0.1175	0.0142	-0.1443	-0.0887
	$\beta_{7,\text{age}_6,\text{deprivation}_{10}}$	0.0113	0.0097	-0.0082	0.0291		$\beta_{10,\text{age}_3,\text{region}_8}$	-0.0288	0.0146	-0.0570	-0.0022
	$\beta_{7,\text{age}_7,\text{deprivation}_{10}}$	0.0003	0.0086	-0.0167	0.0176		$\beta_{10,\text{age}_3,\text{region}_9}$	0.0469	0.0161	0.0142	0.0782
	$\beta_{7,\text{age}_8,\text{deprivation}_{10}}$	-0.0279	0.0082	-0.0433	-0.0113		$\beta_{10,\text{age}_4,\text{region}_1}$	0.0473	0.0183	0.0122	0.0837

Covariate	Parameter	Mean	SD	%2.5	%97.5	Covariate	Parameter	Mean	SD	%2.5	%97.5
Age:Region	$\beta_{10,\text{age}_4,\text{region}_2}$	0.0001	0.0125	-0.0231	0.0245	Age:Region	$\beta_{10,\text{age}_{10},\text{region}_5}$	0.0048	0.0067	-0.0088	0.0175
	$\beta_{10,\text{age}_4,\text{region}_3}$	0.0349	0.0135	0.0096	0.0624		$\beta_{10,\text{age}_{10},\text{region}_6}$	0.0262	0.0068	0.0135	0.0402
	$\beta_{10,\text{age}_4,\text{region}_4}$	0.0128	0.0154	-0.0175	0.0422		$\beta_{10,\text{age}_{10},\text{region}_7}$	0.0224	0.0067	0.0095	0.0355
	$\beta_{10,\text{age}_4,\text{region}_5}$	-0.0214	0.0143	-0.0512	0.0068		$\beta_{10,\text{age}_{10},\text{region}_8}$	0.0091	0.0061	-0.0029	0.0210
	$\beta_{10,\text{age}_4,\text{region}_6}$	-0.0315	0.0129	-0.0539	-0.0043		$\beta_{10,\text{age}_{10},\text{region}_9}$	-0.0380	0.0065	-0.0501	-0.0256
	$\beta_{10,\text{age}_4,\text{region}_7}$	-0.0635	0.0117	-0.0864	-0.0400		$\beta_{10,\text{age}_{11},\text{region}_1}$	0.0004	0.0086	-0.0162	0.0168
	$\beta_{10,\text{age}_4,\text{region}_8}$	-0.0095	0.0111	-0.0304	0.0126		$\beta_{10,\text{age}_{11},\text{region}_2}$	0.0062	0.0060	-0.0057	0.0181
	$\beta_{10,\text{age}_4,\text{region}_9}$	0.0307	0.0136	0.0038	0.0582		$\beta_{10,\text{age}_{11},\text{region}_3}$	-0.0035	0.0073	-0.0181	0.0111
	$\beta_{10,\text{age}_5,\text{region}_1}$	-0.0134	0.0151	-0.0445	0.0153		$\beta_{10,\text{age}_{11},\text{region}_4}$	-0.0101	0.0073	-0.0247	0.0042
	$\beta_{10,\text{age}_5,\text{region}_2}$	-0.0101	0.0096	-0.0284	0.0106		$\beta_{10,\text{age}_{11},\text{region}_5}$	-0.0141	0.0073	-0.0286	0.0001
	$\beta_{10,\text{age}_5,\text{region}_3}$	-0.0051	0.0116	-0.0285	0.0173		$\beta_{10,\text{age}_{11},\text{region}_6}$	0.0064	0.0067	-0.0063	0.0195
	$\beta_{10,\text{age}_5,\text{region}_4}$	-0.0032	0.0110	-0.0245	0.0175		$\beta_{10,\text{age}_{11},\text{region}_7}$	0.0332	0.0069	0.0202	0.0466
	$\beta_{10,\text{age}_5,\text{region}_5}$	0.0212	0.0112	-0.0017	0.0417		$\beta_{10,\text{age}_{11},\text{region}_8}$	0.0081	0.0059	-0.0035	0.0197
	$\beta_{10,\text{age}_5,\text{region}_6}$	0.0090	0.0105	-0.0108	0.0305		$\beta_{10,\text{age}_{11},\text{region}_9}$	-0.0265	0.0069	-0.0402	-0.0127
	$\beta_{10,\text{age}_5,\text{region}_7}$	0.0068	0.0092	-0.0119	0.0243		$\beta_{10,\text{age}_{12},\text{region}_1}$	0.0180	0.0089	0.0009	0.0354
	$\beta_{10,\text{age}_5,\text{region}_8}$	-0.0013	0.0093	-0.0189	0.0171		$\beta_{10,\text{age}_{12},\text{region}_2}$	-0.0064	0.0062	-0.0178	0.0064
	$\beta_{10,\text{age}_5,\text{region}_9}$	-0.0039	0.0112	-0.0252	0.0178		$\beta_{10,\text{age}_{12},\text{region}_3}$	-0.0027	0.0069	-0.0165	0.0107
	$\beta_{10,\text{age}_6,\text{region}_1}$	-0.0464	0.0119	-0.0700	-0.0242		$\beta_{10,\text{age}_{12},\text{region}_4}$	-0.0080	0.0074	-0.0230	0.0065
	$\beta_{10,\text{age}_6,\text{region}_2}$	-0.0243	0.0080	-0.0398	-0.0086		$\beta_{10,\text{age}_{12},\text{region}_5}$	-0.0109	0.0070	-0.0249	0.0027
	$\beta_{10,\text{age}_6,\text{region}_3}$	-0.0106	0.0095	-0.0294	0.0075		$\beta_{10,\text{age}_{12},\text{region}_6}$	0.0170	0.0068	0.0038	0.0306
	$\beta_{10,\text{age}_6,\text{region}_4}$	0.0033	0.0102	-0.0165	0.0245		$\beta_{10,\text{age}_{12},\text{region}_7}$	0.0268	0.0066	0.0138	0.0391
	$\beta_{10,\text{age}_6,\text{region}_5}$	0.0296	0.0092	0.0125	0.0479		$\beta_{10,\text{age}_{12},\text{region}_8}$	-0.0059	0.0060	-0.0180	0.0059
	$\beta_{10,\text{age}_6,\text{region}_6}$	0.0057	0.0094	-0.0133	0.0241		$\beta_{10,\text{age}_{12},\text{region}_9}$	-0.0279	0.0066	-0.0407	-0.0153
	$\beta_{10,\text{age}_6,\text{region}_7}$	0.0181	0.0083	0.0018	0.0336		$\beta_{10,\text{age}_{13},\text{region}_1}$	0.0183	0.0092	0.0008	0.0362
	$\beta_{10,\text{age}_6,\text{region}_8}$	0.0089	0.0077	-0.0070	0.0241		$\beta_{10,\text{age}_{13},\text{region}_2}$	-0.0127	0.0063	-0.0244	-0.0002
	$\beta_{10,\text{age}_6,\text{region}_9}$	0.0157	0.0099	-0.0035	0.0354		$\beta_{10,\text{age}_{13},\text{region}_3}$	-0.0054	0.0072	-0.0192	0.0089
	$\beta_{10,\text{age}_7,\text{region}_1}$	-0.0382	0.0105	-0.0587	-0.0168		$\beta_{10,\text{age}_{13},\text{region}_4}$	-0.0164	0.0074	-0.0308	-0.0012
	$\beta_{10,\text{age}_7,\text{region}_2}$	-0.0134	0.0074	-0.0274	0.0011		$\beta_{10,\text{age}_{13},\text{region}_5}$	-0.0087	0.0071	-0.0225	0.0049
	$\beta_{10,\text{age}_7,\text{region}_3}$	-0.0222	0.0085	-0.0383	-0.0058		$\beta_{10,\text{age}_{13},\text{region}_6}$	0.0093	0.0068	-0.0041	0.0224
	$\beta_{10,\text{age}_7,\text{region}_4}$	-0.0018	0.0087	-0.0189	0.0152		$\beta_{10,\text{age}_{13},\text{region}_7}$	0.0523	0.0066	0.0394	0.0659
	$\beta_{10,\text{age}_7,\text{region}_5}$	0.0187	0.0079	0.0037	0.0341		$\beta_{10,\text{age}_{13},\text{region}_8}$	-0.0015	0.0060	-0.0128	0.0109
	$\beta_{10,\text{age}_7,\text{region}_6}$	0.0253	0.0075	0.0111	0.0397		$\beta_{10,\text{age}_{13},\text{region}_9}$	-0.0353	0.0070	-0.0491	-0.0218
	$\beta_{10,\text{age}_7,\text{region}_7}$	0.0091	0.0075	-0.0058	0.0235		$\beta_{10,\text{age}_{14},\text{region}_1}$	-0.0362	0.0092	-0.0543	-0.0182
	$\beta_{10,\text{age}_7,\text{region}_8}$	0.0219	0.0071	0.0076	0.0353		$\beta_{10,\text{age}_{14},\text{region}_2}$	-0.0261	0.0061	-0.0377	-0.0143
	$\beta_{10,\text{age}_7,\text{region}_9}$	0.0006	0.0082	-0.0155	0.0162		$\beta_{10,\text{age}_{14},\text{region}_3}$	-0.0024	0.0070	-0.0158	0.0111
	$\beta_{10,\text{age}_8,\text{region}_1}$	-0.0296	0.0104	-0.0499	-0.0095		$\beta_{10,\text{age}_{14},\text{region}_4}$	-0.0347	0.0076	-0.0496	-0.0194
	$\beta_{10,\text{age}_8,\text{region}_2}$	-0.0088	0.0068	-0.0221	0.0044		$\beta_{10,\text{age}_{14},\text{region}_5}$	-0.0180	0.0070	-0.0320	-0.0045
	$\beta_{10,\text{age}_8,\text{region}_3}$	-0.0126	0.0078	-0.0278	0.0026		$\beta_{10,\text{age}_{14},\text{region}_6}$	-0.0028	0.0066	-0.0150	0.0106
	$\beta_{10,\text{age}_8,\text{region}_4}$	-0.0050	0.0086	-0.0214	0.0119		$\beta_{10,\text{age}_{14},\text{region}_7}$	0.0993	0.0069	0.0862	0.1132
	$\beta_{10,\text{age}_8,\text{region}_5}$	0.0251	0.0078	0.0097	0.0394		$\beta_{10,\text{age}_{14},\text{region}_8}$	0.0204	0.0059	0.0090	0.0322
	$\beta_{10,\text{age}_8,\text{region}_6}$	0.0066	0.0076	-0.0073	0.0215		$\beta_{10,\text{age}_{14},\text{region}_9}$	0.0006	0.0066	-0.0127	0.0137
	$\beta_{10,\text{age}_8,\text{region}_7}$	0.0152	0.0071	0.0012	0.0293	Age : Year^2	β_{11,age_1}	0.0093	0.0106	-0.0113	0.0292
	$\beta_{10,\text{age}_8,\text{region}_8}$	0.0114	0.0067	-0.0017	0.0248		β_{11,age_2}	-0.0114	0.0091	-0.0299	0.0047
	$\beta_{10,\text{age}_8,\text{region}_9}$	-0.0024	0.0076	-0.0170	0.0123		β_{11,age_3}	-0.0018	0.0062	-0.0140	0.0107
	$\beta_{10,\text{age}_9,\text{region}_1}$	-0.0415	0.0092	-0.0591	-0.0236		β_{11,age_4}	0.0053	0.0052	-0.0046	0.0152
	$\beta_{10,\text{age}_9,\text{region}_2}$	0.0072	0.0064	-0.0048	0.0199		β_{11,age_5}	-0.0056	0.0047	-0.0149	0.0039
	$\beta_{10,\text{age}_9,\text{region}_3}$	-0.0221	0.0070	-0.0349	-0.0081		β_{11,age_6}	0.0090	0.0036	0.0017	0.0158
	$\beta_{10,\text{age}_9,\text{region}_4}$	-0.0002	0.0076	-0.0150	0.0154		β_{11,age_7}	0.0084	0.0032	0.0021	0.0146
	$\beta_{10,\text{age}_9,\text{region}_5}$	0.0126	0.0073	-0.0021	0.0275		β_{11,age_8}	0.0085	0.0028	0.0030	0.0143
	$\beta_{10,\text{age}_9,\text{region}_6}$	0.0239	0.0070	0.0102	0.0385		β_{11,age_9}	-0.0144	0.0028	-0.0196	-0.0088
	$\beta_{10,\text{age}_9,\text{region}_7}$	0.0264	0.0070	0.0134	0.0402		$\beta_{11,\text{age}_{10}}$	-0.0283	0.0027	-0.0335	-0.0230
	$\beta_{10,\text{age}_9,\text{region}_8}$	0.0101	0.0062	-0.0020	0.0224		$\beta_{11,\text{age}_{11}}$	0.0091	0.0026	0.0036	0.0142
	$\beta_{10,\text{age}_9,\text{region}_9}$	-0.0164	0.0069	-0.0295	-0.0028		$\beta_{11,\text{age}_{12}}$	0.0046	0.0025	-0.0005	0.0097
	$\beta_{10,\text{age}_{10},\text{region}_1}$	0.0002	0.0090	-0.0176	0.0185		$\beta_{11,\text{age}_{13}}$	-0.0076	0.0029	-0.0135	-0.0021
	$\beta_{10,\text{age}_{10},\text{region}_2}$	-0.0024	0.0064	-0.0152	0.0100		$\beta_{11,\text{age}_{14}}$	0.0149	0.0026	0.0097	0.0201
	$\beta_{10,\text{age}_{10},\text{region}_3}$	-0.0234	0.0071	-0.0376	-0.0092		σ^2	0.0011	0.0001	0.0009	0.0013
	$\beta_{10,\text{age}_{10},\text{region}_4}$	0.0013	0.0075	-0.0130	0.0166						

S3 Table. Estimated coefficients for the best fitting model for male all-cancer mortality.

Covariate	Parameter	Mean	SD	%2.5	%97.5	Covariate	Parameter	Mean	SD	%2.5	%97.5
Intercept	β_0	-6.2740	0.0024	-6.2780	-6.2690		$\beta_{7,\mathrm{age}_7,\mathrm{deprivation}_2}$	0.1423	0.0106	0.1192	0.1607
Age	β_{1,age_1}	-3.4840	0.0168	-3.5160	-3.4520		$\beta_{7,\mathrm{age}_8,\mathrm{deprivation}_2}$	0.1062	0.0085	0.0911	0.1234
	β_{1,age_2}	-2.9230	0.0110	-2.9450	-2.9050		$\beta_{7,\mathrm{age}_9,\mathrm{deprivation}_2}$	0.0875	0.0087	0.0684	0.1035
	β_{1,age_3}	-2.3930	0.0142	-2.4280	-2.3740		$\beta_{7,\mathrm{age}_{10},\mathrm{deprivation}_2}$	0.0494	0.0079	0.0332	0.0644
	β_{1,age_4}	-1.8040	0.0110	-1.8260	-1.7850		$\beta_{7,\mathrm{age}_{11},\mathrm{deprivation}_2}$	0.0138	0.0079	-0.0001	0.0301
	β_{1,age_5}	-1.0960	0.0113	-1.1170	-1.0810		$\beta_{7,\mathrm{age}_{12},\mathrm{deprivation}_2}$	-0.0759	0.0074	-0.0906	-0.0609
	β_{1,age_6}	-0.3831	0.0084	-0.4006	-0.3642		$\beta_{7,\mathrm{age}_{13},\mathrm{deprivation}_2}$	-0.1440	0.0083	-0.1590	-0.1292
	β_{1,age_7}	0.2998	0.0063	0.2882	0.3125		$\beta_{7,\mathrm{age}_1,\mathrm{deprivation}_3}$	-0.1606	0.0489	-0.2553	-0.0533
	β_{1,age_8}	0.8570	0.0046	0.8477	0.8651		$\beta_{7,\mathrm{age}_2,\mathrm{deprivation}_3}$	-0.0830	0.0416	-0.1411	-0.0108
	β_{1,age_9}	1.3440	0.0048	1.3330	1.3520		$\beta_{7,\mathrm{age}_3,\mathrm{deprivation}_3}$	0.0321	0.0491	-0.0507	0.0949
	$\beta_{1,\mathrm{age}_{10}}$	1.7630	0.0044	1.7540	1.7720		$\beta_{7,\mathrm{age}_4,\mathrm{deprivation}_3}$	-0.0199	0.0157	-0.0552	0.0138
	$\beta_{1,\mathrm{age}_{11}}$	2.1470	0.0038	2.1400	2.1540		$\beta_{7,\mathrm{age}_5,\mathrm{deprivation}_3}$	0.0519	0.0169	0.0152	0.0777
	$\beta_{1,\mathrm{age}_{12}}$	2.5020	0.0033	2.4960	2.5080		$\beta_{7,\mathrm{age}_6,\mathrm{deprivation}_3}$	0.0897	0.0177	0.0598	0.1238
	$\beta_{1,\mathrm{age}_{13}}$	3.1690	0.0039	3.1600	3.1750		$\beta_{7,\mathrm{age}_7,\mathrm{deprivation}_3}$	0.0738	0.0098	0.0526	0.0905
Year	β_2	-0.0698	0.0024	-0.0750	-0.0658		$\beta_{7,\mathrm{age}_8,\mathrm{deprivation}_3}$	0.0605	0.0102	0.0403	0.0817
AAD	β_3	0.2637	0.0046	0.2561	0.2731		$\beta_{7,\mathrm{age}_9,\mathrm{deprivation}_3}$	0.0420	0.0107	0.0204	0.0628
Region	$\beta_{4,\mathrm{region}_1}$	-0.0689	0.0047	-0.0780	-0.0598		$\beta_{7,\mathrm{age}_{10},\mathrm{deprivation}_3}$	0.0380	0.0078	0.0230	0.0532
	$\beta_{4,\mathrm{region}_2}$	0.1115	0.0030	0.1057	0.1172		$\beta_{7,\mathrm{age}_{11},\mathrm{deprivation}_3}$	-0.0009	0.0097	-0.0220	0.0162
	$\beta_{4,\mathrm{region}_3}$	-0.1120	0.0040	-0.1204	-0.1035		$\beta_{7,\mathrm{age}_{12},\mathrm{deprivation}_3}$	-0.0399	0.0106	-0.0624	-0.0204
	$\beta_{4,\mathrm{region}_4}$	-0.0180	0.0033	-0.0239	-0.0115		$\beta_{7,\mathrm{age}_{13},\mathrm{deprivation}_3}$	-0.0837	0.0088	-0.1021	-0.0674
	$\beta_{4,\mathrm{region}_5}$	0.2940	0.0069	0.2817	0.3086		$\beta_{7,\mathrm{age}_1,\mathrm{deprivation}_4}$	-0.0350	0.0636	-0.1420	0.0656
	$\beta_{4,\mathrm{region}_6}$	-0.0235	0.0026	-0.0284	-0.0182		$\beta_{7,\mathrm{age}_2,\mathrm{deprivation}_4}$	-0.1351	0.0451	-0.2288	-0.0419
	$\beta_{4,\mathrm{region}_7}$	-0.3492	0.0056	-0.3627	-0.3392		$\beta_{7,\mathrm{age}_3,\mathrm{deprivation}_4}$	-0.0120	0.0282	-0.0639	0.0373
	$\beta_{4,\mathrm{region}_8}$	0.1051	0.0029	0.0993	0.1109		$\beta_{7,\mathrm{age}_4,\mathrm{deprivation}_4}$	0.0095	0.0219	-0.0316	0.0599
	$\beta_{4,\mathrm{region}_9}$	0.0609	0.0036	0.0545	0.0689		$\beta_{7,\mathrm{age}_5,\mathrm{deprivation}_4}$	0.0450	0.0254	-0.0044	0.0858
Deprivation	$\beta_{5,\mathrm{deprivation}_1}$	0.7792	0.0092	0.7610	0.7997		$\beta_{7,\mathrm{age}_6,\mathrm{deprivation}_4}$	0.0632	0.0131	0.0357	0.0896
	$\beta_{5,\mathrm{deprivation}_2}$	0.4615	0.0072	0.4494	0.4778		$\beta_{7,\mathrm{age}_7,\mathrm{deprivation}_4}$	0.0384	0.0121	0.0193	0.0658
	$\beta_{5,\mathrm{deprivation}_3}$	0.1457	0.0065	0.1350	0.1597		$\beta_{7,\mathrm{age}_8,\mathrm{deprivation}_4}$	0.0492	0.0132	0.0272	0.0762
	$\beta_{5,\mathrm{deprivation}_4}$	0.0279	0.0080	0.0134	0.0422		$\beta_{7,\mathrm{age}_9,\mathrm{deprivation}_4}$	0.0261	0.0120	0.0043	0.0512
	$\beta_{5,\mathrm{deprivation}_5}$	-0.0606	0.0056	-0.0706	-0.0494		$\beta_{7,\mathrm{age}_{10},\mathrm{deprivation}_4}$	0.0092	0.0115	-0.0130	0.0307
	$\beta_{5,\mathrm{deprivation}_6}$	-0.2827	0.0071	-0.2938	-0.2690		$\beta_{7,\mathrm{age}_{11},\mathrm{deprivation}_4}$	0.0042	0.0110	-0.0191	0.0229
	$\beta_{5,\mathrm{deprivation}_7}$	-0.2139	0.0055	-0.2244	-0.2048		$\beta_{7,\mathrm{age}_{12},\mathrm{deprivation}_4}$	-0.0146	0.0105	-0.0324	0.0062
	$\beta_{5,\mathrm{deprivation}_8}$	-0.3447	0.0075	-0.3601	-0.3322		$\beta_{7,\mathrm{age}_{13},\mathrm{deprivation}_4}$	-0.0482	0.0105	-0.0678	-0.0284
	$\beta_{5,\mathrm{deprivation}_9}$	-0.2207	0.0054	-0.2300	-0.2105		$\beta_{7,\mathrm{age}_1,\mathrm{deprivation}_5}$	-0.0203	0.0374	-0.0995	0.0394
	$\beta_{5,\mathrm{deprivation}_{10}}$	-0.2918	0.0059	-0.3047	-0.2802		$\beta_{7,\mathrm{age}_2,\mathrm{deprivation}_5}$	-0.0680	0.0601	-0.1690	0.0171
Year^2	β_6	0.0084	0.0027	0.0033	0.0136		$\beta_{7,\mathrm{age}_3,\mathrm{deprivation}_5}$	-0.0377	0.0261	-0.0787	0.0151
Age:Deprivation	$\beta_{7,\mathrm{age}_1,\mathrm{deprivation}_1}$	-0.1408	0.0390	-0.2267	-0.0660		$\beta_{7,\mathrm{age}_4,\mathrm{deprivation}_5}$	0.0220	0.0322	-0.0411	0.0730
	$\beta_{7,\mathrm{age}_2,\mathrm{deprivation}_1}$	0.0040	0.0348	-0.0516	0.0828		$\beta_{7,\mathrm{age}_5,\mathrm{deprivation}_5}$	0.0338	0.0161	0.0011	0.0653
	$\beta_{7,\mathrm{age}_3,\mathrm{deprivation}_1}$	-0.0300	0.0262	-0.0916	0.0184		$\beta_{7,\mathrm{age}_6,\mathrm{deprivation}_5}$	0.0116	0.0124	-0.0107	0.0358
	$\beta_{7,\mathrm{age}_4,\mathrm{deprivation}_1}$	0.1178	0.0257	0.0668	0.1780		$\beta_{7,\mathrm{age}_7,\mathrm{deprivation}_5}$	0.0105	0.0100	-0.0098	0.0299
	$\beta_{7,\mathrm{age}_5,\mathrm{deprivation}_1}$	0.1149	0.0191	0.0830	0.1541		$\beta_{7,\mathrm{age}_8,\mathrm{deprivation}_5}$	0.0137	0.0104	-0.0078	0.0330
	$\beta_{7,\mathrm{age}_6,\mathrm{deprivation}_1}$	0.0953	0.0144	0.0666	0.1239		$\beta_{7,\mathrm{age}_9,\mathrm{deprivation}_5}$	0.0166	0.0099	-0.0034	0.0342
	$\beta_{7,\mathrm{age}_7,\mathrm{deprivation}_1}$	0.1350	0.0116	0.1120	0.1582		$\beta_{7,\mathrm{age}_{10},\mathrm{deprivation}_5}$	0.0074	0.0088	-0.0092	0.0246
	$\beta_{7,\mathrm{age}_8,\mathrm{deprivation}_1}$	0.1117	0.0098	0.0932	0.1315		$\beta_{7,\mathrm{age}_{11},\mathrm{deprivation}_5}$	-0.0011	0.0088	-0.0172	0.0157
	$\beta_{7,\mathrm{age}_9,\mathrm{deprivation}_1}$	0.0697	0.0090	0.0527	0.0870		$\beta_{7,\mathrm{age}_{12},\mathrm{deprivation}_5}$	0.0089	0.0085	-0.0058	0.0260
	$\beta_{7,\mathrm{age}_{10},\mathrm{deprivation}_1}$	0.0050	0.0083	-0.0111	0.0209		$\beta_{7,\mathrm{age}_{13},\mathrm{deprivation}_5}$	0.0027	0.0082	-0.0129	0.0179
	$\beta_{7,\mathrm{age}_{11},\mathrm{deprivation}_1}$	-0.0654	0.0083	-0.0823	-0.0496		$\beta_{7,\mathrm{age}_1,\mathrm{deprivation}_6}$	0.0831	0.0583	0.0033	0.2053
	$\beta_{7,\mathrm{age}_{12},\mathrm{deprivation}_1}$	-0.1569	0.0090	-0.1743	-0.1387		$\beta_{7,\mathrm{age}_2,\mathrm{deprivation}_6}$	-0.1508	0.0528	-0.2121	-0.0414
	$\beta_{7,\mathrm{age}_{13},\mathrm{deprivation}_1}$	-0.2603	0.0091	-0.2783	-0.2423		$\beta_{7,\mathrm{age}_3,\mathrm{deprivation}_6}$	-0.0929	0.0449	-0.1968	-0.0401
	$\beta_{7,\mathrm{age}_1,\mathrm{deprivation}_2}$	-0.2106	0.0317	-0.2763	-0.1399		$\beta_{7,\mathrm{age}_4,\mathrm{deprivation}_6}$	0.0731	0.0355	0.0235	0.1366
	$\beta_{7,\mathrm{age}_2,\mathrm{deprivation}_2}$	-0.1752	0.0298	-0.2408	-0.1216		$\beta_{7,\mathrm{age}_5,\mathrm{deprivation}_6}$	-0.0338	0.0170	-0.0619	-0.0014
	$\beta_{7,\mathrm{age}_3,\mathrm{deprivation}_2}$	-0.0759	0.0295	-0.1244	-0.0281		$\beta_{7,\mathrm{age}_6,\mathrm{deprivation}_6}$	-0.0046	0.0131	-0.0311	0.0204
	$\beta_{7,\mathrm{age}_4,\mathrm{deprivation}_2}$	0.0298	0.0270	-0.0080	0.0929		$\beta_{7,\mathrm{age}_7,\mathrm{deprivation}_6}$	-0.0049	0.0100	-0.0252	0.0150
	$\beta_{7,\mathrm{age}_5,\mathrm{deprivation}_2}$	0.1197	0.0197	0.0756	0.1541		$\beta_{7,\mathrm{age}_8,\mathrm{deprivation}_6}$	-0.0147	0.0106	-0.0384	0.0038
	$\beta_{7,\mathrm{age}_6,\mathrm{deprivation}_2}$	0.1328	0.0143	0.1024	0.1576		$\beta_{7,\mathrm{age}_9,\mathrm{deprivation}_6}$	0.0112	0.0090	-0.0100	0.0285

Covariate	Parameter	Mean	SD	%2.5	%97.5	Covariate	Parameter	Mean	SD	%2.5	%97.5
Age:Deprivation	$\beta_{7,\text{age}_{10},\text{deprivation}_6}$	0.0014	0.0088	-0.0151	0.0162	Age:Year	β_{8,age_1}	-0.0134	0.0209	-0.0532	0.0222
	$\beta_{7,\text{age}_{11},\text{deprivation}_6}$	0.0233	0.0090	0.0050	0.0396		β_{8,age_2}	0.0158	0.0120	-0.0030	0.0399
	$\beta_{7,\text{age}_{12},\text{deprivation}_6}$	0.0471	0.0082	0.0310	0.0619		β_{8,age_3}	0.0324	0.0133	0.0026	0.0537
	$\beta_{7,\text{age}_{13},\text{deprivation}_6}$	0.0626	0.0080	0.0461	0.0786		β_{8,age_4}	-0.0103	0.0065	-0.0229	0.0014
	$\beta_{7,\text{age}_1,\text{deprivation}_7}$	0.0685	0.0434	-0.0208	0.1424		β_{8,age_5}	-0.0355	0.0080	-0.0484	-0.0148
	$\beta_{7,\text{age}_2,\text{deprivation}_7}$	0.0782	0.0277	0.0186	0.1317		β_{8,age_6}	-0.0447	0.0051	-0.0546	-0.0335
	$\beta_{7,\text{age}_3,\text{deprivation}_7}$	0.1041	0.0269	0.0624	0.1563		β_{8,age_7}	-0.0293	0.0043	-0.0373	-0.0199
	$\beta_{7,\text{age}_4,\text{deprivation}_7}$	-0.0212	0.0234	-0.0700	0.0282		β_{8,age_8}	-0.0129	0.0043	-0.0216	-0.0045
	$\beta_{7,\text{age}_5,\text{deprivation}_7}$	-0.0608	0.0215	-0.0999	-0.0161		β_{8,age_9}	-0.0060	0.0033	-0.0122	0.0006
	$\beta_{7,\text{age}_6,\text{deprivation}_7}$	-0.0830	0.0151	-0.1149	-0.0563		$\beta_{8,\text{age}_{10}}$	-0.0014	0.0033	-0.0074	0.0053
	$\beta_{7,\text{age}_7,\text{deprivation}_7}$	-0.0542	0.0141	-0.0816	-0.0215		$\beta_{8,\text{age}_{11}}$	-0.0045	0.0034	-0.0107	0.0023
	$\beta_{7,\text{age}_8,\text{deprivation}_7}$	-0.0588	0.0089	-0.0740	-0.0422		$\beta_{8,\text{age}_{12}}$	0.0307	0.0031	0.0250	0.0370
	$\beta_{7,\text{age}_9,\text{deprivation}_7}$	-0.0454	0.0098	-0.0643	-0.0266		$\beta_{8,\text{age}_{13}}$	0.0790	0.0030	0.0735	0.0848
	$\beta_{7,\text{age}_{10},\text{deprivation}_7}$	-0.0431	0.0094	-0.0601	-0.0238	Region:Year	$\beta_{9,\text{region}_1}$	-0.0107	0.0037	-0.0172	-0.0030
	$\beta_{7,\text{age}_{11},\text{deprivation}_7}$	0.0013	0.0083	-0.0145	0.0181		$\beta_{9,\text{region}_2}$	-0.0087	0.0024	-0.0139	-0.0040
	$\beta_{7,\text{age}_{12},\text{deprivation}_7}$	0.0363	0.0078	0.0224	0.0534		$\beta_{9,\text{region}_3}$	0.0008	0.0028	-0.0047	0.0062
	$\beta_{7,\text{age}_{13},\text{deprivation}_7}$	0.0781	0.0077	0.0632	0.0929		$\beta_{9,\text{region}_4}$	0.0126	0.0030	0.0067	0.0182
	$\beta_{7,\text{age}_1,\text{deprivation}_8}$	0.1268	0.0412	0.0565	0.2206		$\beta_{9,\text{region}_5}$	0.0038	0.0028	-0.0013	0.0094
	$\beta_{7,\text{age}_2,\text{deprivation}_8}$	0.2546	0.0249	0.2139	0.3055		$\beta_{9,\text{region}_6}$	0.0084	0.0027	0.0025	0.0132
	$\beta_{7,\text{age}_3,\text{deprivation}_8}$	-0.0343	0.0454	-0.0988	0.0467		$\beta_{9,\text{region}_7}$	-0.0270	0.0029	-0.0323	-0.0213
	$\beta_{7,\text{age}_4,\text{deprivation}_8}$	-0.0772	0.0370	-0.1637	-0.0267		$\beta_{9,\text{region}_8}$	0.0065	0.0025	0.0020	0.0120
	$\beta_{7,\text{age}_5,\text{deprivation}_8}$	-0.0530	0.0157	-0.0910	-0.0257		$\beta_{9,\text{region}_9}$	0.0144	0.0028	0.0095	0.0203
	$\beta_{7,\text{age}_6,\text{deprivation}_8}$	-0.1231	0.0160	-0.1557	-0.0900	Deprivation:Year	$\beta_{10,\text{deprivation}_1}$	0.0254	0.0029	0.0195	0.0312
	$\beta_{7,\text{age}_7,\text{deprivation}_8}$	-0.1055	0.0124	-0.1273	-0.0790		$\beta_{10,\text{deprivation}_2}$	0.0113	0.0025	0.0063	0.0158
	$\beta_{7,\text{age}_8,\text{deprivation}_8}$	-0.0738	0.0107	-0.0947	-0.0510		$\beta_{10,\text{deprivation}_3}$	0.0152	0.0034	0.0088	0.0224
	$\beta_{7,\text{age}_9,\text{deprivation}_8}$	-0.0562	0.0098	-0.0745	-0.0363		$\beta_{10,\text{deprivation}_4}$	0.0051	0.0029	-0.0000	0.0109
	$\beta_{7,\text{age}_{10},\text{deprivation}_8}$	-0.0239	0.0096	-0.0418	-0.0052		$\beta_{10,\text{deprivation}_5}$	0.0045	0.0026	-0.0004	0.0100
	$\beta_{7,\text{age}_{11},\text{deprivation}_8}$	0.0100	0.0085	-0.0093	0.0247		$\beta_{10,\text{deprivation}_6}$	-0.0043	0.0026	-0.0092	0.0009
	$\beta_{7,\text{age}_{12},\text{deprivation}_8}$	0.0518	0.0082	0.0359	0.0669		$\beta_{10,\text{deprivation}_7}$	-0.0075	0.0025	-0.0123	-0.0025
	$\beta_{7,\text{age}_{13},\text{deprivation}_8}$	0.1039	0.0072	0.0883	0.1162		$\beta_{10,\text{deprivation}_8}$	-0.0147	0.0028	-0.0199	-0.0093
	$\beta_{7,\text{age}_1,\text{deprivation}_9}$	0.1596	0.0666	0.0798	0.2850		$\beta_{10,\text{deprivation}_9}$	-0.0129	0.0030	-0.0181	-0.0062
	$\beta_{7,\text{age}_2,\text{deprivation}_9}$	0.2222	0.0264	0.1720	0.2805		$\beta_{10,\text{deprivation}_{10}}$	-0.0220	0.0033	-0.0289	-0.0156
	$\beta_{7,\text{age}_3,\text{deprivation}_9}$	0.0260	0.0196	-0.0121	0.0594	Region:AAD	$\beta_{11,\text{region}_1}$	-0.0136	0.0040	-0.0213	-0.0054
	$\beta_{7,\text{age}_4,\text{deprivation}_9}$	-0.0647	0.0308	-0.1266	-0.0162		$\beta_{11,\text{region}_2}$	-0.0147	0.0030	-0.0207	-0.0090
	$\beta_{7,\text{age}_5,\text{deprivation}_9}$	-0.0686	0.0199	-0.1082	-0.0385		$\beta_{11,\text{region}_3}$	0.0016	0.0036	-0.0059	0.0084
	$\beta_{7,\text{age}_6,\text{deprivation}_9}$	-0.1020	0.0178	-0.1354	-0.0659		$\beta_{11,\text{region}_4}$	0.0089	0.0043	0.0008	0.0184
	$\beta_{7,\text{age}_7,\text{deprivation}_9}$	-0.1178	0.0135	-0.1460	-0.0941		$\beta_{11,\text{region}_5}$	0.0189	0.0032	0.0131	0.0256
	$\beta_{7,\text{age}_8,\text{deprivation}_9}$	-0.0957	0.0099	-0.1142	-0.0757		$\beta_{11,\text{region}_6}$	0.0139	0.0041	0.0058	0.0218
	$\beta_{7,\text{age}_9,\text{deprivation}_9}$	-0.0821	0.0092	-0.1009	-0.0663		$\beta_{11,\text{region}_7}$	-0.0192	0.0036	-0.0261	-0.0110
	$\beta_{7,\text{age}_{10},\text{deprivation}_9}$	-0.0326	0.0079	-0.0488	-0.0175		$\beta_{11,\text{region}_8}$	-0.0039	0.0039	-0.0122	0.0032
	$\beta_{7,\text{age}_{11},\text{deprivation}_9}$	-0.0033	0.0085	-0.0216	0.0116		$\beta_{11,\text{region}_9}$	0.0081	0.0042	0.0009	0.0163
	$\beta_{7,\text{age}_{12},\text{deprivation}_9}$	0.0503	0.0079	0.0352	0.0661	Region : Year^2	β_{12,age_1}	-0.0096	0.0118	-0.0341	0.0094
	$\beta_{7,\text{age}_{13},\text{deprivation}_9}$	0.1087	0.0074	0.0937	0.1223		β_{12,age_2}	0.0094	0.0081	-0.0043	0.0238
	$\beta_{7,\text{age}_1,\text{deprivation}_{10}}$	0.1293	0.0598	0.0215	0.2295		β_{12,age_3}	-0.0050	0.0089	-0.0269	0.0113
	$\beta_{7,\text{age}_2,\text{deprivation}_{10}}$	0.0530	0.0249	0.0066	0.0907		β_{12,age_4}	0.0162	0.0069	0.0033	0.0315
	$\beta_{7,\text{age}_3,\text{deprivation}_{10}}$	0.1206	0.0290	0.0683	0.1814		β_{12,age_5}	0.0227	0.0082	0.0088	0.0395
	$\beta_{7,\text{age}_4,\text{deprivation}_{10}}$	-0.0693	0.0297	-0.1169	-0.0155		β_{12,age_6}	0.0097	0.0064	-0.0068	0.0189
	$\beta_{7,\text{age}_5,\text{deprivation}_{10}}$	-0.1491	0.0256	-0.1884	-0.1000		β_{12,age_7}	-0.0133	0.0044	-0.0223	-0.0053
	$\beta_{7,\text{age}_6,\text{deprivation}_{10}}$	-0.0799	0.0175	-0.1110	-0.0486		β_{12,age_8}	-0.0023	0.0044	-0.0104	0.0063
	$\beta_{7,\text{age}_7,\text{deprivation}_{10}}$	-0.1176	0.0142	-0.1465	-0.0935		β_{12,age_9}	-0.0143	0.0037	-0.0215	-0.0067
	$\beta_{7,\text{age}_8,\text{deprivation}_{10}}$	-0.0982	0.0114	-0.1184	-0.0756		$\beta_{12,\text{age}_{10}}$	-0.0060	0.0032	-0.0119	0.0008
	$\beta_{7,\text{age}_9,\text{deprivation}_{10}}$	-0.0694	0.0114	-0.0889	-0.0483		$\beta_{12,\text{age}_{11}}$	-0.0018	0.0033	-0.0079	0.0054
	$\beta_{7,\text{age}_{10},\text{deprivation}_{10}}$	-0.0107	0.0100	-0.0312	0.0080		$\beta_{12,\text{age}_{12}}$	-0.0208	0.0037	-0.0285	-0.0140
	$\beta_{7,\text{age}_{11},\text{deprivation}_{10}}$	0.0181	0.0099	-0.0012	0.0374		$\beta_{12,\text{age}_{13}}$	0.0152	0.0040	0.0081	0.0226
	$\beta_{7,\text{age}_{12},\text{deprivation}_{10}}$	0.0929	0.0097	0.0732	0.1134		σ^2	0.0004	0.0001	0.0003	0.0007
	$\beta_{7,\text{age}_{13},\text{deprivation}_{10}}$	0.1803	0.0091	0.1595	0.1985						

S4 Table. Estimated coefficients for the best fitting model for female all-cancer mortality.

Covariate	Parameter	Mean	SD	%2.5	%97.5	Covariate	Parameter	Mean	SD	%2.5	%97.5
Intercept	β_0	-6.3700	0.0019	-6.3740	-6.3670		$\beta_{6,\text{age}_1,\text{deprivation}_2}$	-0.0831	0.0366	-0.1563	-0.0135
Age	β_{1,age_1}	-3.4660	0.0143	-3.4980	-3.4420		$\beta_{6,\text{age}_2,\text{deprivation}_2}$	-0.1153	0.0461	-0.1961	-0.0303
	β_{1,age_2}	-2.5500	0.0155	-2.5760	-2.5240		$\beta_{6,\text{age}_3,\text{deprivation}_2}$	-0.0233	0.0293	-0.0905	0.0360
	β_{1,age_3}	-1.9420	0.0128	-1.9650	-1.9150		$\beta_{6,\text{age}_4,\text{deprivation}_2}$	0.0180	0.0211	-0.0239	0.0569
	β_{1,age_4}	-1.3290	0.0074	-1.3460	-1.3160		$\beta_{6,\text{age}_5,\text{deprivation}_2}$	0.0378	0.0157	0.0086	0.0675
	β_{1,age_5}	-0.7461	0.0068	-0.7596	-0.7328		$\beta_{6,\text{age}_6,\text{deprivation}_2}$	0.0525	0.0146	0.0206	0.0792
	β_{1,age_6}	-0.1910	0.0051	-0.2017	-0.1811		$\beta_{6,\text{age}_7,\text{deprivation}_2}$	0.0668	0.0127	0.0420	0.0903
	β_{1,age_7}	0.2933	0.0046	0.2840	0.3030		$\beta_{6,\text{age}_8,\text{deprivation}_2}$	0.0588	0.0117	0.0379	0.0832
	β_{1,age_8}	0.7219	0.0038	0.7148	0.7296		$\beta_{6,\text{age}_9,\text{deprivation}_2}$	0.0940	0.0109	0.0740	0.1147
	β_{1,age_9}	1.1010	0.0034	1.0940	1.1080		$\beta_{6,\text{age}_{10},\text{deprivation}_2}$	0.0458	0.0099	0.0286	0.0664
	$\beta_{1,\text{age}_{10}}$	1.4590	0.0034	1.4530	1.4670		$\beta_{6,\text{age}_{11},\text{deprivation}_2}$	0.0111	0.0101	-0.0085	0.0304
	$\beta_{1,\text{age}_{11}}$	1.7830	0.0031	1.7770	1.7890		$\beta_{6,\text{age}_{12},\text{deprivation}_2}$	-0.0361	0.0099	-0.0550	-0.0165
	$\beta_{1,\text{age}_{12}}$	2.0580	0.0031	2.0520	2.0640		$\beta_{6,\text{age}_{13},\text{deprivation}_2}$	-0.1270	0.0097	-0.1467	-0.1081
	$\beta_{1,\text{age}_{13}}$	2.8090	0.0030	2.8030	2.8140		$\beta_{6,\text{age}_1,\text{deprivation}_3}$	-0.0495	0.0421	-0.1241	0.0369
Year	β_2	-0.0529	0.0023	-0.0576	-0.0486		$\beta_{6,\text{age}_2,\text{deprivation}_3}$	0.0019	0.0356	-0.0503	0.0840
AAD	β_3	0.2120	0.0069	0.2011	0.2252		$\beta_{6,\text{age}_3,\text{deprivation}_3}$	-0.0312	0.0413	-0.0944	0.0526
Region	$\beta_{4,\text{region}_1}$	-0.0498	0.0064	-0.0618	-0.0383		$\beta_{6,\text{age}_4,\text{deprivation}_3}$	0.0015	0.0236	-0.0407	0.0502
	$\beta_{4,\text{region}_2}$	0.0906	0.0034	0.0846	0.0977		$\beta_{6,\text{age}_5,\text{deprivation}_3}$	0.0294	0.0188	-0.0037	0.0674
	$\beta_{4,\text{region}_3}$	-0.0856	0.0043	-0.0949	-0.0780		$\beta_{6,\text{age}_6,\text{deprivation}_3}$	0.0277	0.0168	-0.0018	0.0593
	$\beta_{4,\text{region}_4}$	0.0170	0.0037	0.0098	0.0242		$\beta_{6,\text{age}_7,\text{deprivation}_3}$	0.0317	0.0134	0.0030	0.0534
	$\beta_{4,\text{region}_5}$	0.1990	0.0073	0.1859	0.2128		$\beta_{6,\text{age}_8,\text{deprivation}_3}$	0.0516	0.0129	0.0281	0.0772
	$\beta_{4,\text{region}_6}$	-0.0669	0.0036	-0.0742	-0.0599		$\beta_{6,\text{age}_9,\text{deprivation}_3}$	0.0258	0.0116	0.0016	0.0472
	$\beta_{4,\text{region}_7}$	-0.4221	0.0117	-0.4437	-0.4040		$\beta_{6,\text{age}_{10},\text{deprivation}_3}$	0.0215	0.0095	0.0030	0.0397
	$\beta_{4,\text{region}_8}$	0.1000	0.0043	0.0924	0.1091		$\beta_{6,\text{age}_{11},\text{deprivation}_3}$	-0.0021	0.0089	-0.0198	0.0154
	$\beta_{4,\text{region}_9}$	0.2178	0.0088	0.2039	0.2349		$\beta_{6,\text{age}_{12},\text{deprivation}_3}$	-0.0241	0.0099	-0.0452	-0.0061
Deprivation	$\beta_{5,\text{deprivation}_1}$	0.3480	0.0076	0.3347	0.3636		$\beta_{6,\text{age}_{13},\text{deprivation}_3}$	-0.0842	0.0099	-0.1061	-0.0664
	$\beta_{5,\text{deprivation}_2}$	0.1944	0.0060	0.1835	0.2052		$\beta_{6,\text{age}_1,\text{deprivation}_4}$	-0.0049	0.0402	-0.0827	0.0608
	$\beta_{5,\text{deprivation}_3}$	-0.0173	0.0065	-0.0290	-0.0018		$\beta_{6,\text{age}_2,\text{deprivation}_4}$	-0.0517	0.0413	-0.1396	0.0328
	$\beta_{5,\text{deprivation}_4}$	-0.0515	0.0055	-0.0628	-0.0418		$\beta_{6,\text{age}_3,\text{deprivation}_4}$	-0.0285	0.0286	-0.0750	0.0324
	$\beta_{5,\text{deprivation}_5}$	-0.0634	0.0052	-0.0743	-0.0530		$\beta_{6,\text{age}_4,\text{deprivation}_4}$	0.0626	0.0273	0.0079	0.1090
	$\beta_{5,\text{deprivation}_6}$	-0.2018	0.0125	-0.2232	-0.1819		$\beta_{6,\text{age}_5,\text{deprivation}_4}$	-0.0269	0.0201	-0.0620	0.0109
	$\beta_{5,\text{deprivation}_7}$	-0.0549	0.0058	-0.0667	-0.0438		$\beta_{6,\text{age}_6,\text{deprivation}_4}$	0.0245	0.0154	-0.0073	0.0516
	$\beta_{5,\text{deprivation}_8}$	-0.1590	0.0057	-0.1688	-0.1473		$\beta_{6,\text{age}_7,\text{deprivation}_4}$	0.0112	0.0127	-0.0133	0.0380
	$\beta_{5,\text{deprivation}_9}$	0.0025	0.0078	-0.0120	0.0189		$\beta_{6,\text{age}_8,\text{deprivation}_4}$	0.0194	0.0118	-0.0036	0.0419
	$\beta_{5,\text{deprivation}_{10}}$	0.0030	0.0092	-0.0111	0.0215		$\beta_{6,\text{age}_9,\text{deprivation}_4}$	0.0087	0.0098	-0.0112	0.0271
Age:Deprivation	$\beta_{6,\text{age}_1,\text{deprivation}_1}$	-0.1739	0.0356	-0.2458	-0.1107		$\beta_{6,\text{age}_{10},\text{deprivation}_4}$	0.0190	0.0084	0.0037	0.0369
	$\beta_{6,\text{age}_2,\text{deprivation}_1}$	-0.0838	0.0403	-0.1631	-0.0118		$\beta_{6,\text{age}_{11},\text{deprivation}_4}$	0.0057	0.0087	-0.0127	0.0219
	$\beta_{6,\text{age}_3,\text{deprivation}_1}$	-0.0491	0.0296	-0.1069	0.0096		$\beta_{6,\text{age}_{12},\text{deprivation}_4}$	-0.0086	0.0079	-0.0244	0.0070
	$\beta_{6,\text{age}_4,\text{deprivation}_1}$	0.0265	0.0213	-0.0151	0.0693		$\beta_{6,\text{age}_{13},\text{deprivation}_4}$	-0.0304	0.0076	-0.0453	-0.0154
	$\beta_{6,\text{age}_5,\text{deprivation}_1}$	0.0733	0.0216	0.0271	0.1156		$\beta_{6,\text{age}_1,\text{deprivation}_5}$	-0.0055	0.0434	-0.1005	0.0664
	$\beta_{6,\text{age}_6,\text{deprivation}_1}$	0.0827	0.0155	0.0539	0.1145		$\beta_{6,\text{age}_2,\text{deprivation}_5}$	-0.0208	0.0436	-0.0979	0.0645
	$\beta_{6,\text{age}_7,\text{deprivation}_1}$	0.0969	0.0121	0.0741	0.1206		$\beta_{6,\text{age}_3,\text{deprivation}_5}$	-0.0465	0.0350	-0.1015	0.0195
	$\beta_{6,\text{age}_8,\text{deprivation}_1}$	0.1040	0.0113	0.0823	0.1257		$\beta_{6,\text{age}_4,\text{deprivation}_5}$	-0.0127	0.0284	-0.0645	0.0388
	$\beta_{6,\text{age}_9,\text{deprivation}_1}$	0.0987	0.0103	0.0779	0.1184		$\beta_{6,\text{age}_5,\text{deprivation}_5}$	0.0200	0.0184	-0.0182	0.0546
	$\beta_{6,\text{age}_{10},\text{deprivation}_1}$	0.0760	0.0100	0.0565	0.0962		$\beta_{6,\text{age}_6,\text{deprivation}_5}$	0.0096	0.0173	-0.0224	0.0435
	$\beta_{6,\text{age}_{11},\text{deprivation}_1}$	0.0095	0.0094	-0.0089	0.0277		$\beta_{6,\text{age}_7,\text{deprivation}_5}$	0.0099	0.0113	-0.0128	0.0314
	$\beta_{6,\text{age}_{12},\text{deprivation}_1}$	-0.0693	0.0096	-0.0886	-0.0513		$\beta_{6,\text{age}_8,\text{deprivation}_5}$	0.0078	0.0124	-0.0159	0.0306
	$\beta_{6,\text{age}_{13},\text{deprivation}_1}$	-0.1915	0.0098	-0.2102	-0.1722		$\beta_{6,\text{age}_9,\text{deprivation}_5}$	0.0188	0.0109	-0.0020	0.0400

Covariate	Parameter	Mean	SD	%2.5	%97.5	Covariate	Parameter	Mean	SD	%2.5	%97.5
Age:Deprivation	$\beta_{6,\text{age}_{10},\text{deprivation}_5}$	-0.0000	0.0103	-0.0188	0.0220	Age:Deprivation	$\beta_{6,\text{age}_9,\text{deprivation}_9}$	-0.0606	0.0127	-0.0881	-0.0353
	$\beta_{6,\text{age}_{11},\text{deprivation}_5}$	0.0011	0.0087	-0.0153	0.0188		$\beta_{6,\text{age}_{10},\text{deprivation}_9}$	-0.0499	0.0112	-0.0728	-0.0258
	$\beta_{6,\text{age}_{12},\text{deprivation}_5}$	0.0028	0.0089	-0.0134	0.0225		$\beta_{6,\text{age}_{11},\text{deprivation}_9}$	-0.0117	0.0102	-0.0314	0.0085
	$\beta_{6,\text{age}_{13},\text{deprivation}_5}$	0.0154	0.0083	0.0012	0.0350		$\beta_{6,\text{age}_{12},\text{deprivation}_9}$	0.0291	0.0106	0.0089	0.0478
	$\beta_{6,\text{age}_1,\text{deprivation}_6}$	-0.0208	0.0641	-0.1460	0.0869		$\beta_{6,\text{age}_{13},\text{deprivation}_9}$	0.0951	0.0095	0.0764	0.1130
	$\beta_{6,\text{age}_2,\text{deprivation}_6}$	0.0107	0.0437	-0.0543	0.0981		$\beta_{6,\text{age}_1,\text{deprivation}_{10}}$	0.0777	0.0544	-0.0390	0.1570
	$\beta_{6,\text{age}_3,\text{deprivation}_6}$	-0.0175	0.0377	-0.0804	0.0527		$\beta_{6,\text{age}_2,\text{deprivation}_{10}}$	0.0149	0.0587	-0.0951	0.1189
	$\beta_{6,\text{age}_4,\text{deprivation}_6}$	0.0098	0.0221	-0.0313	0.0592		$\beta_{6,\text{age}_3,\text{deprivation}_{10}}$	0.0457	0.0305	-0.0252	0.0968
	$\beta_{6,\text{age}_5,\text{deprivation}_6}$	0.0137	0.0249	-0.0444	0.0580		$\beta_{6,\text{age}_4,\text{deprivation}_{10}}$	0.0033	0.0247	-0.0394	0.0556
	$\beta_{6,\text{age}_6,\text{deprivation}_6}$	-0.0357	0.0159	-0.0705	-0.0058		$\beta_{6,\text{age}_5,\text{deprivation}_{10}}$	-0.0607	0.0194	-0.1050	-0.0236
	$\beta_{6,\text{age}_7,\text{deprivation}_6}$	-0.0140	0.0154	-0.0430	0.0148		$\beta_{6,\text{age}_6,\text{deprivation}_{10}}$	-0.0522	0.0157	-0.0805	-0.0156
	$\beta_{6,\text{age}_8,\text{deprivation}_6}$	-0.0084	0.0135	-0.0326	0.0180		$\beta_{6,\text{age}_7,\text{deprivation}_{10}}$	-0.0657	0.0133	-0.0929	-0.0423
	$\beta_{6,\text{age}_9,\text{deprivation}_6}$	-0.0264	0.0137	-0.0501	0.0026		$\beta_{6,\text{age}_8,\text{deprivation}_{10}}$	-0.0569	0.0113	-0.0787	-0.0361
	$\beta_{6,\text{age}_{10},\text{deprivation}_6}$	-0.0056	0.0106	-0.0237	0.0161		$\beta_{6,\text{age}_9,\text{deprivation}_{10}}$	-0.0683	0.0105	-0.0877	-0.0484
	$\beta_{6,\text{age}_{11},\text{deprivation}_6}$	0.0088	0.0127	-0.0123	0.0368		$\beta_{6,\text{age}_{10},\text{deprivation}_{10}}$	-0.0373	0.0115	-0.0611	-0.0159
	$\beta_{6,\text{age}_{12},\text{deprivation}_6}$	0.0290	0.0120	0.0076	0.0529		$\beta_{6,\text{age}_{11},\text{deprivation}_{10}}$	0.0058	0.0104	-0.0165	0.0258
	$\beta_{6,\text{age}_{13},\text{deprivation}_6}$	0.0566	0.0114	0.0372	0.0781		$\beta_{6,\text{age}_{12},\text{deprivation}_{10}}$	0.0542	0.0104	0.0346	0.0743
	$\beta_{6,\text{age}_1,\text{deprivation}_7}$	0.1268	0.0490	0.0393	0.2097		$\beta_{6,\text{age}_{13},\text{deprivation}_{10}}$	0.1395	0.0088	0.1218	0.1573
	$\beta_{6,\text{age}_2,\text{deprivation}_7}$	0.0639	0.0414	-0.0167	0.1444	Age:Year	β_{7,age_1}	0.0196	0.0137	-0.0104	0.0425
	$\beta_{6,\text{age}_3,\text{deprivation}_7}$	0.0178	0.0257	-0.0320	0.0690		β_{7,age_2}	0.0001	0.0120	-0.0204	0.0246
	$\beta_{6,\text{age}_4,\text{deprivation}_7}$	-0.0081	0.0246	-0.0436	0.0453		β_{7,age_3}	-0.0150	0.0103	-0.0354	0.0041
	$\beta_{6,\text{age}_5,\text{deprivation}_7}$	0.0080	0.0206	-0.0303	0.0558		β_{7,age_4}	-0.0246	0.0107	-0.0524	-0.0082
	$\beta_{6,\text{age}_6,\text{deprivation}_7}$	-0.0294	0.0158	-0.0592	0.0012		β_{7,age_5}	-0.0430	0.0060	-0.0546	-0.0317
	$\beta_{6,\text{age}_7,\text{deprivation}_7}$	-0.0458	0.0144	-0.0716	-0.0134		β_{7,age_6}	-0.0550	0.0054	-0.0669	-0.0451
	$\beta_{6,\text{age}_8,\text{deprivation}_7}$	-0.0633	0.0134	-0.0859	-0.0337		β_{7,age_7}	-0.0338	0.0045	-0.0428	-0.0246
	$\beta_{6,\text{age}_9,\text{deprivation}_7}$	-0.0402	0.0096	-0.0594	-0.0220		β_{7,age_8}	-0.0153	0.0041	-0.0229	-0.0070
	$\beta_{6,\text{age}_{10},\text{deprivation}_7}$	-0.0452	0.0108	-0.0656	-0.0239		β_{7,age_9}	0.0059	0.0034	-0.0006	0.0128
	$\beta_{6,\text{age}_{11},\text{deprivation}_7}$	-0.0133	0.0092	-0.0306	0.0048		$\beta_{7,\text{age}_{10}}$	0.0111	0.0035	0.0046	0.0178
	$\beta_{6,\text{age}_{12},\text{deprivation}_7}$	-0.0062	0.0096	-0.0237	0.0132		$\beta_{7,\text{age}_{11}}$	0.0157	0.0034	0.0093	0.0224
	$\beta_{6,\text{age}_{13},\text{deprivation}_7}$	0.0349	0.0086	0.0187	0.0521		$\beta_{7,\text{age}_{12}}$	0.0514	0.0035	0.0444	0.0578
	$\beta_{6,\text{age}_1,\text{deprivation}_8}$	0.0422	0.0346	-0.0248	0.1088		$\beta_{7,\text{age}_{13}}$	0.0829	0.0032	0.0767	0.0891
	$\beta_{6,\text{age}_2,\text{deprivation}_8}$	0.0725	0.0372	0.0011	0.1399	Deprivation:Year	$\beta_{8,\text{deprivation}_1}$	0.0292	0.0034	0.0228	0.0362
	$\beta_{6,\text{age}_3,\text{deprivation}_8}$	0.0621	0.0266	0.0103	0.1041		$\beta_{8,\text{deprivation}_2}$	0.0262	0.0033	0.0204	0.0332
	$\beta_{6,\text{age}_4,\text{deprivation}_8}$	-0.0433	0.0326	-0.1023	0.0139		$\beta_{8,\text{deprivation}_3}$	0.0129	0.0032	0.0066	0.0192
	$\beta_{6,\text{age}_5,\text{deprivation}_8}$	-0.0260	0.0171	-0.0537	0.0223		$\beta_{8,\text{deprivation}_4}$	0.0099	0.0031	0.0042	0.0163
	$\beta_{6,\text{age}_6,\text{deprivation}_8}$	-0.0333	0.0161	-0.0614	-0.0005		$\beta_{8,\text{deprivation}_5}$	-0.0003	0.0031	-0.0062	0.0057
	$\beta_{6,\text{age}_7,\text{deprivation}_8}$	-0.0531	0.0143	-0.0808	-0.0256		$\beta_{8,\text{deprivation}_6}$	0.0030	0.0031	-0.0031	0.0092
	$\beta_{6,\text{age}_8,\text{deprivation}_8}$	-0.0524	0.0114	-0.0755	-0.0316		$\beta_{8,\text{deprivation}_7}$	-0.0165	0.0035	-0.0233	-0.0096
	$\beta_{6,\text{age}_9,\text{deprivation}_8}$	-0.0505	0.0104	-0.0707	-0.0305		$\beta_{8,\text{deprivation}_8}$	-0.0175	0.0034	-0.0242	-0.0113
	$\beta_{6,\text{age}_{10},\text{deprivation}_8}$	-0.0241	0.0104	-0.0457	-0.0054		$\beta_{8,\text{deprivation}_9}$	-0.0196	0.0034	-0.0264	-0.0124
	$\beta_{6,\text{age}_{11},\text{deprivation}_8}$	-0.0150	0.0106	-0.0378	0.0035		$\beta_{8,\text{deprivation}_{10}}$	-0.0272	0.0038	-0.0340	-0.0192
	$\beta_{6,\text{age}_{12},\text{deprivation}_8}$	0.0292	0.0099	0.0104	0.0484	Region:Year	$\beta_{9,\text{region}_1}$	-0.0039	0.0036	-0.0115	0.0028
	$\beta_{6,\text{age}_{13},\text{deprivation}_8}$	0.0917	0.0088	0.0729	0.1087		$\beta_{9,\text{region}_2}$	-0.0016	0.0027	-0.0066	0.0039
	$\beta_{6,\text{age}_1,\text{deprivation}_9}$	0.0911	0.0568	-0.0198	0.2050		$\beta_{9,\text{region}_3}$	0.0008	0.0031	-0.0053	0.0064
	$\beta_{6,\text{age}_2,\text{deprivation}_9}$	0.1076	0.0351	0.0532	0.1830		$\beta_{9,\text{region}_4}$	0.0095	0.0032	0.0027	0.0155
	$\beta_{6,\text{age}_3,\text{deprivation}_9}$	0.0705	0.0279	0.0270	0.1283		$\beta_{9,\text{region}_5}$	0.0083	0.0031	0.0026	0.0149
	$\beta_{6,\text{age}_4,\text{deprivation}_9}$	-0.0577	0.0279	-0.1156	0.0000		$\beta_{9,\text{region}_6}$	0.0058	0.0031	-0.0006	0.0116
	$\beta_{6,\text{age}_5,\text{deprivation}_9}$	-0.0685	0.0195	-0.1017	-0.0208		$\beta_{9,\text{region}_7}$	-0.0289	0.0034	-0.0356	-0.0223
	$\beta_{6,\text{age}_6,\text{deprivation}_9}$	-0.0465	0.0177	-0.0785	-0.0099		$\beta_{9,\text{region}_8}$	0.0070	0.0029	0.0009	0.0124
	$\beta_{6,\text{age}_7,\text{deprivation}_9}$	-0.0379	0.0146	-0.0658	-0.0092		$\beta_{9,\text{region}_9}$	0.0030	0.0031	-0.0031	0.0090
	$\beta_{6,\text{age}_8,\text{deprivation}_9}$	-0.0606	0.0115	-0.0839	-0.0369		σ^2	0.0015	0.0001	0.0012	0.0018

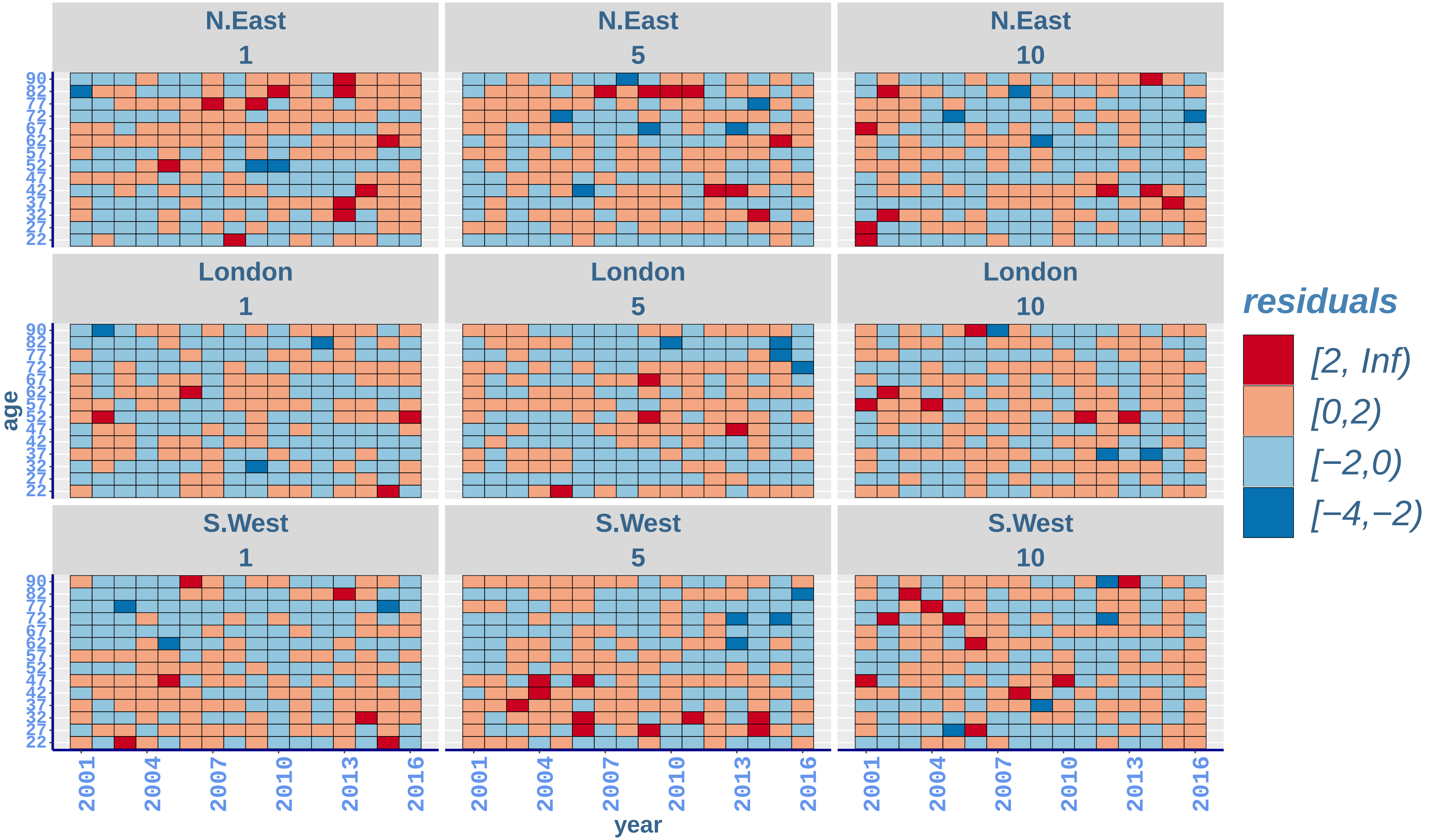

S1 Fig. Heat map of Pearson residuals for all-cancer morbidity for males in North East, London, and South West, deprivation deciles 1, 5, and 10: orange/light blue cells indicate areas with good fit, while red/dark blue cells indicate areas with poor fit. Note that there is a small number of residuals greater than 4, and these are included in the last category.

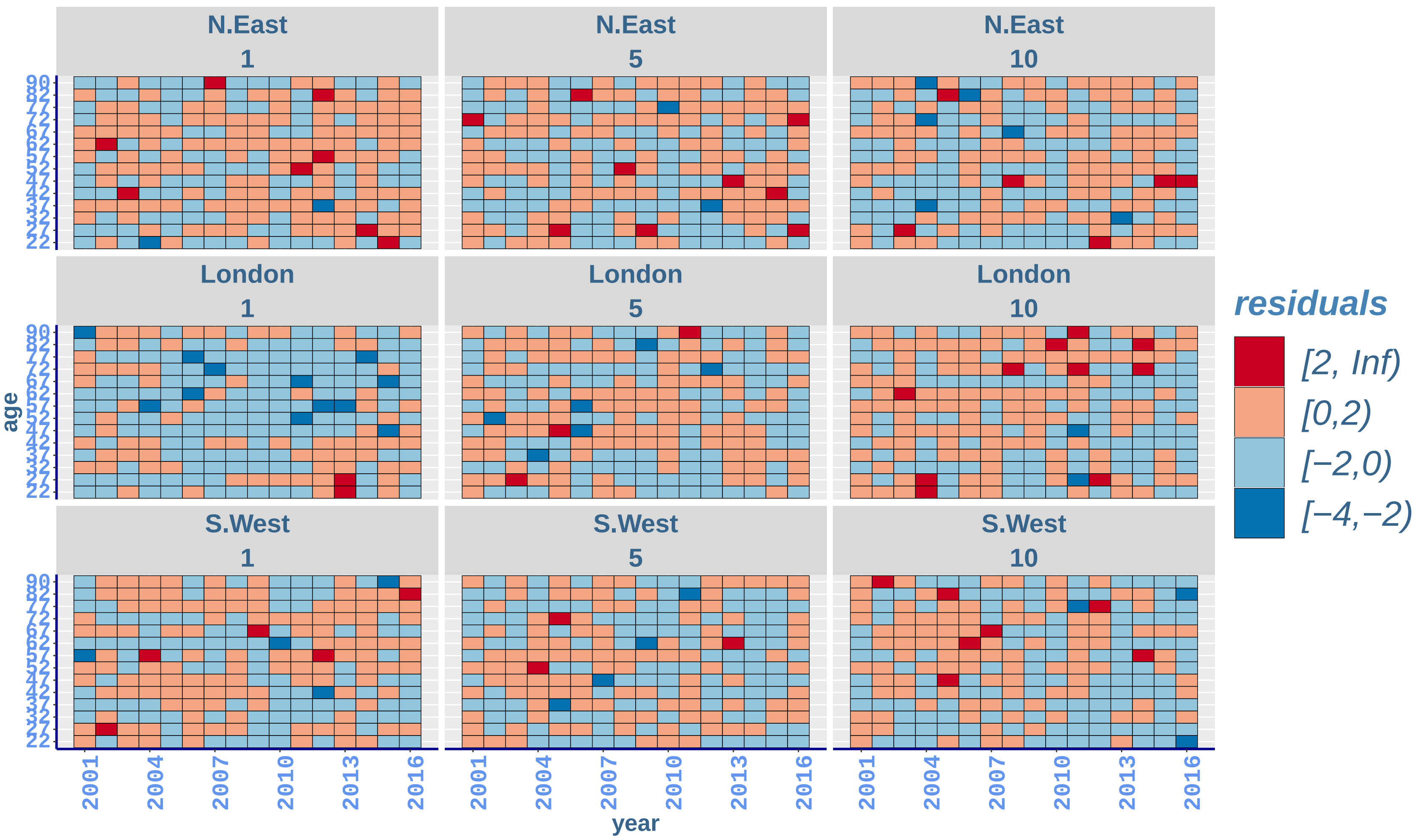

S2 Fig. Heat map of Pearson residuals for all-cancer morbidity for females in North East, London, and South West, deprivation deciles 1, 5, and 10: orange/light blue cells indicate areas with good fit, while red/dark blue cells indicate areas with poor fit. Note that there is a small number of residuals greater than 4, and these are included in the last category.

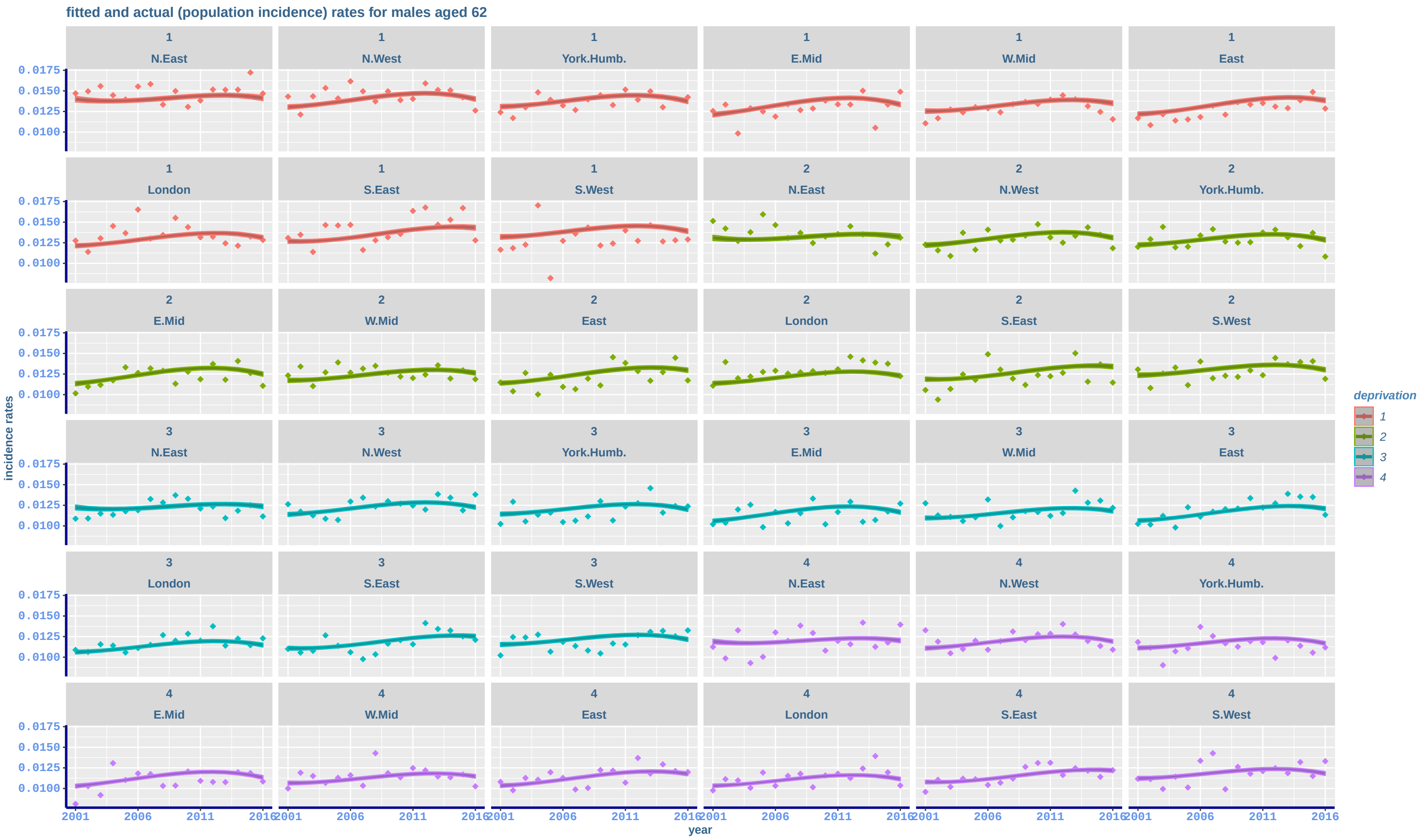

S3 Fig. All-cancer morbidity for males, age 62, deprivation deciles 1-4 for all regions in England between 2001 and 2016: observed rates (dots), fitted rates (lines), with 95% credible intervals for the fitted rates.

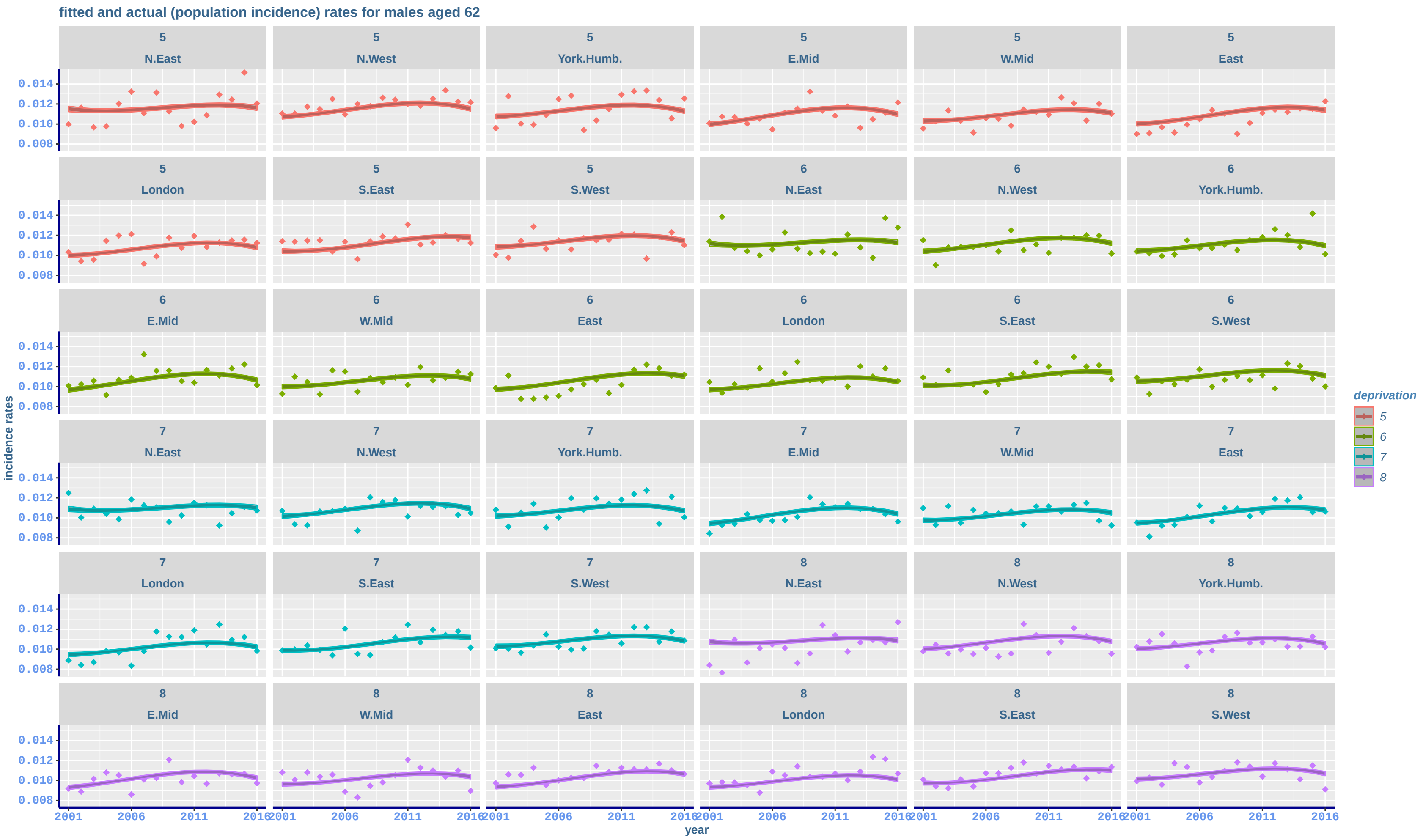

S4 Fig. All-cancer morbidity for males, age 62, deprivation deciles 5-8 for all regions in England between 2001 and 2016: observed rates (dots), fitted rates (lines), with 95% credible intervals for the fitted rates.

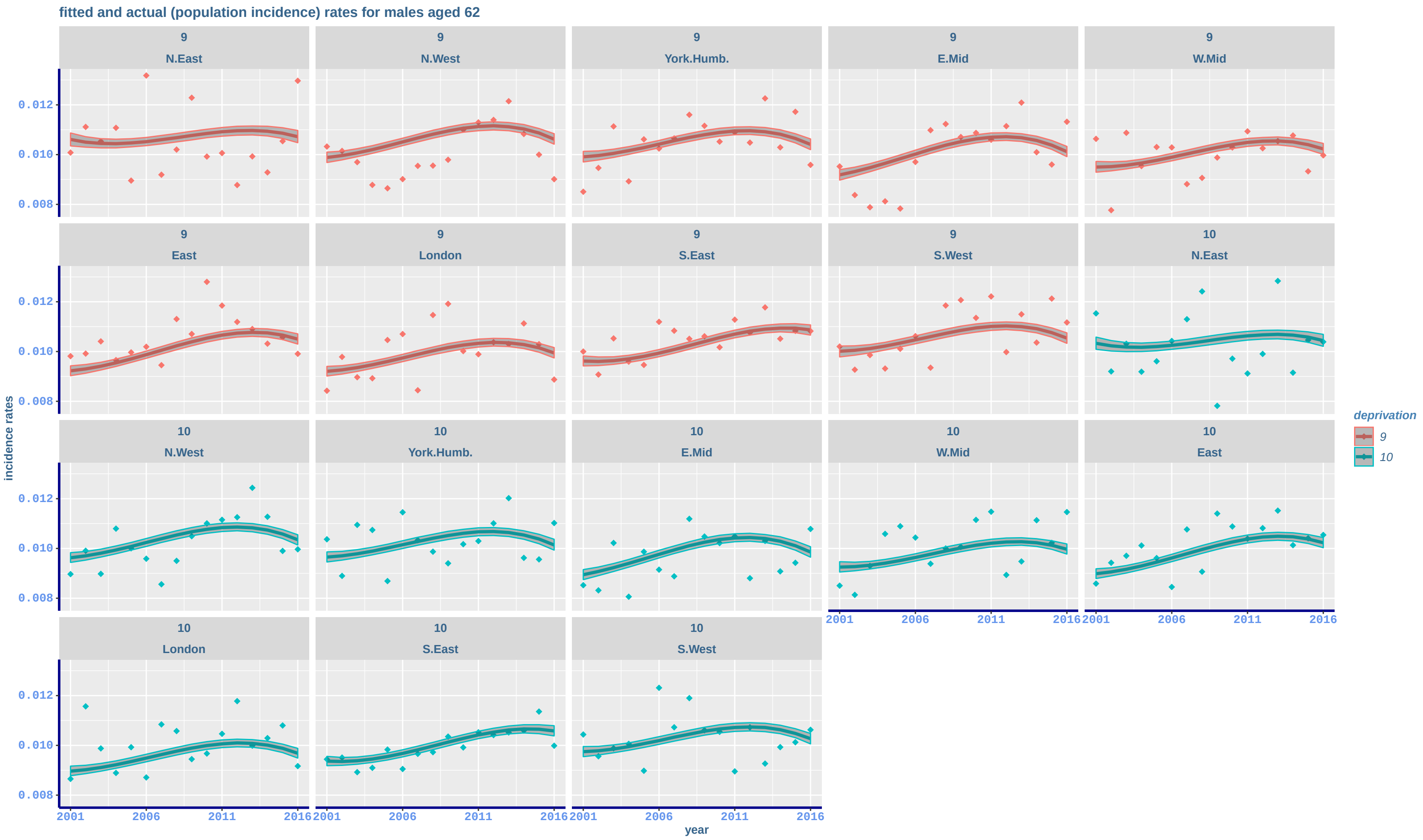

S5 Fig. All-cancer morbidity for males, age 62, deprivation deciles 9-10 for all regions in England between 2001 and 2016: observed rates (dots), fitted rates (lines), with 95% credible intervals for the fitted rates.

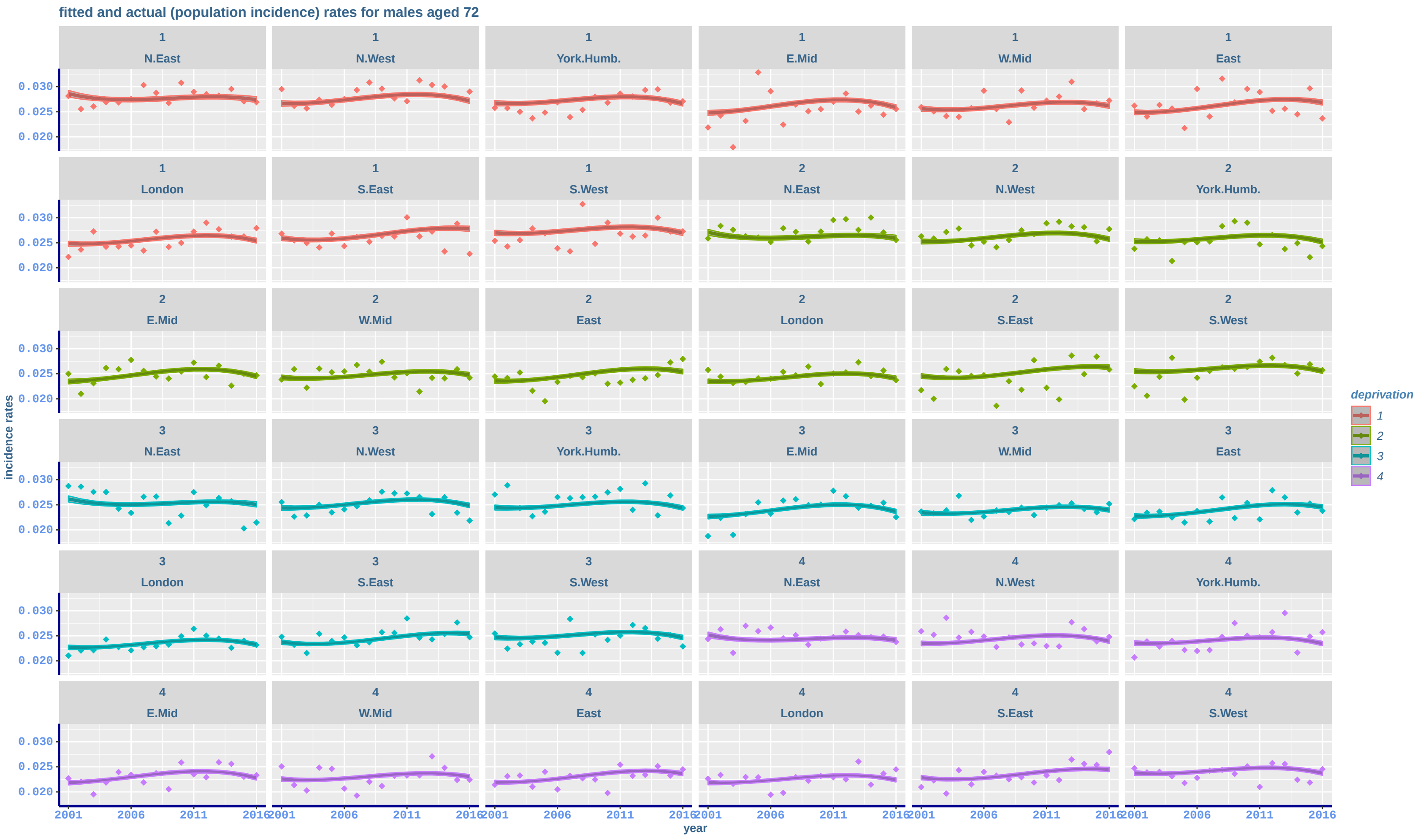

S6 Fig. All-cancer morbidity for males, age 72, deprivation deciles 1-4 for all regions in England between 2001 and 2016: observed rates (dots), fitted rates (lines), with 95% credible intervals for the fitted rates.

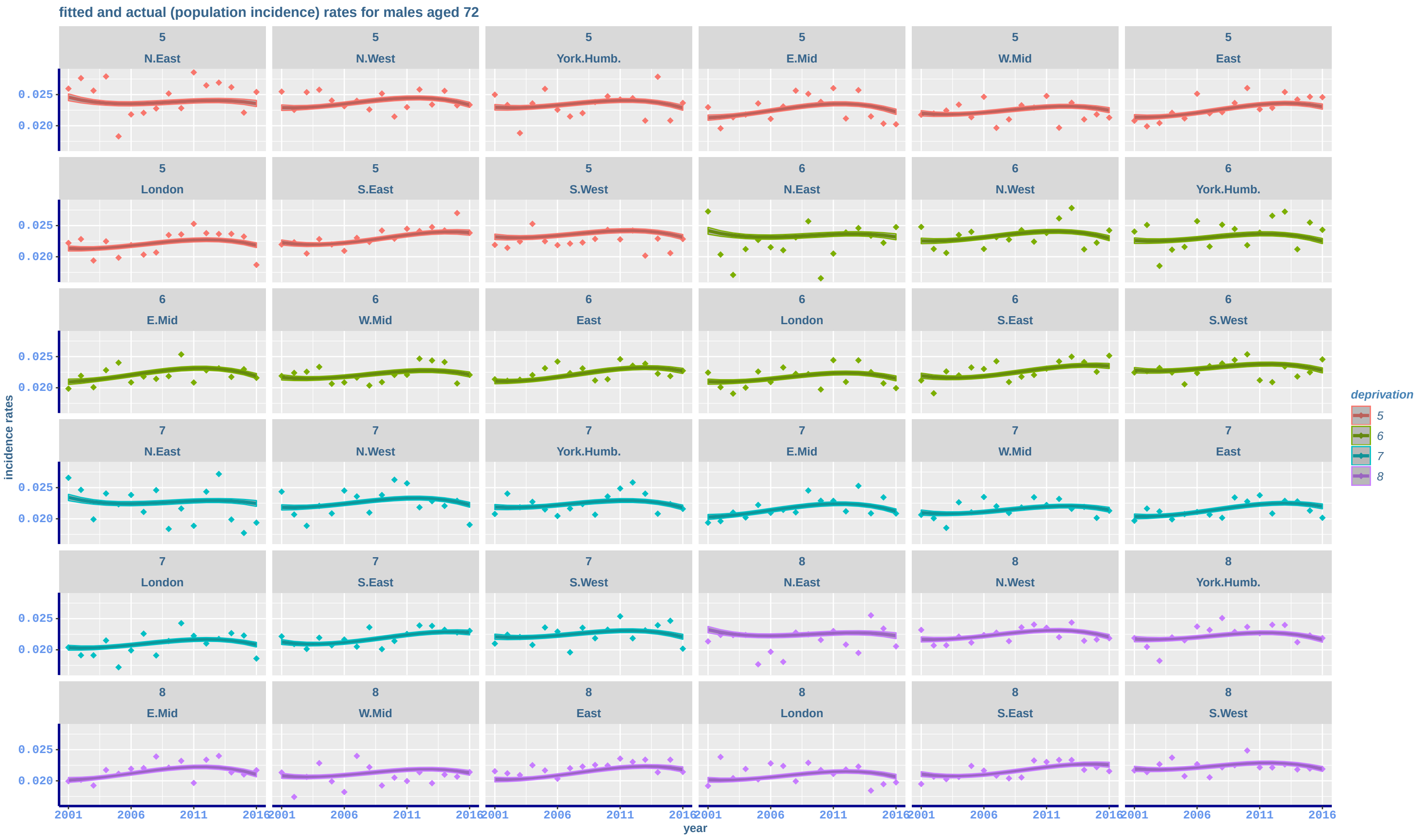

S7 Fig. All-cancer morbidity for males, age 72, deprivation deciles 5-8 for all regions in England between 2001 and 2016: observed rates (dots), fitted rates (lines), with 95% credible intervals for the fitted rates.

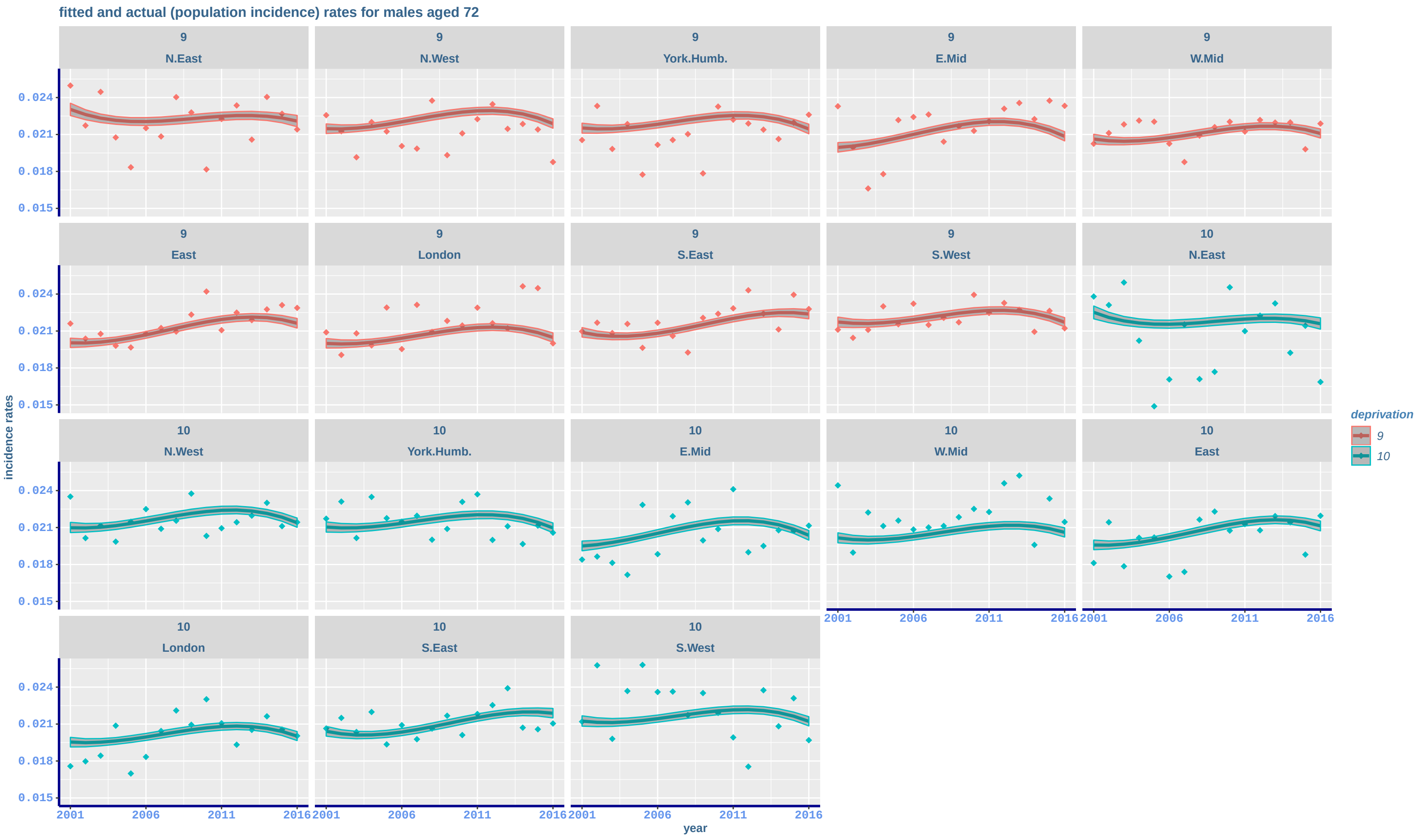

S8 Fig. All-cancer morbidity for males, age 72, deprivation deciles 9-10 for all regions in England between 2001 and 2016: observed rates (dots), fitted rates (lines), with 95% credible intervals for the fitted rates.

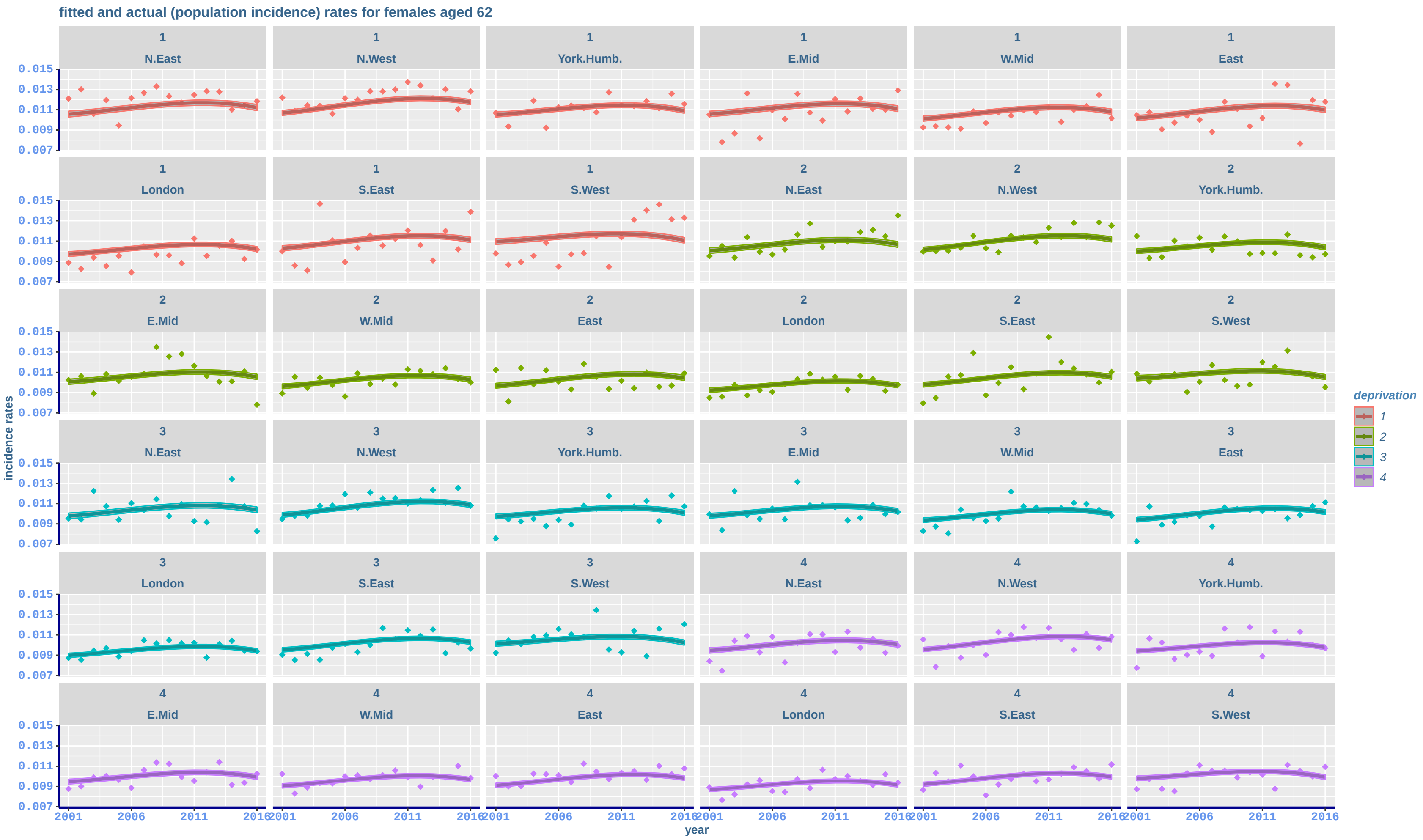

S9 Fig. All-cancer morbidity for females, age 62, deprivation deciles 1-4 for all regions in England between 2001 and 2016: observed rates (dots), fitted rates (lines), with 95% credible intervals for the fitted rates.

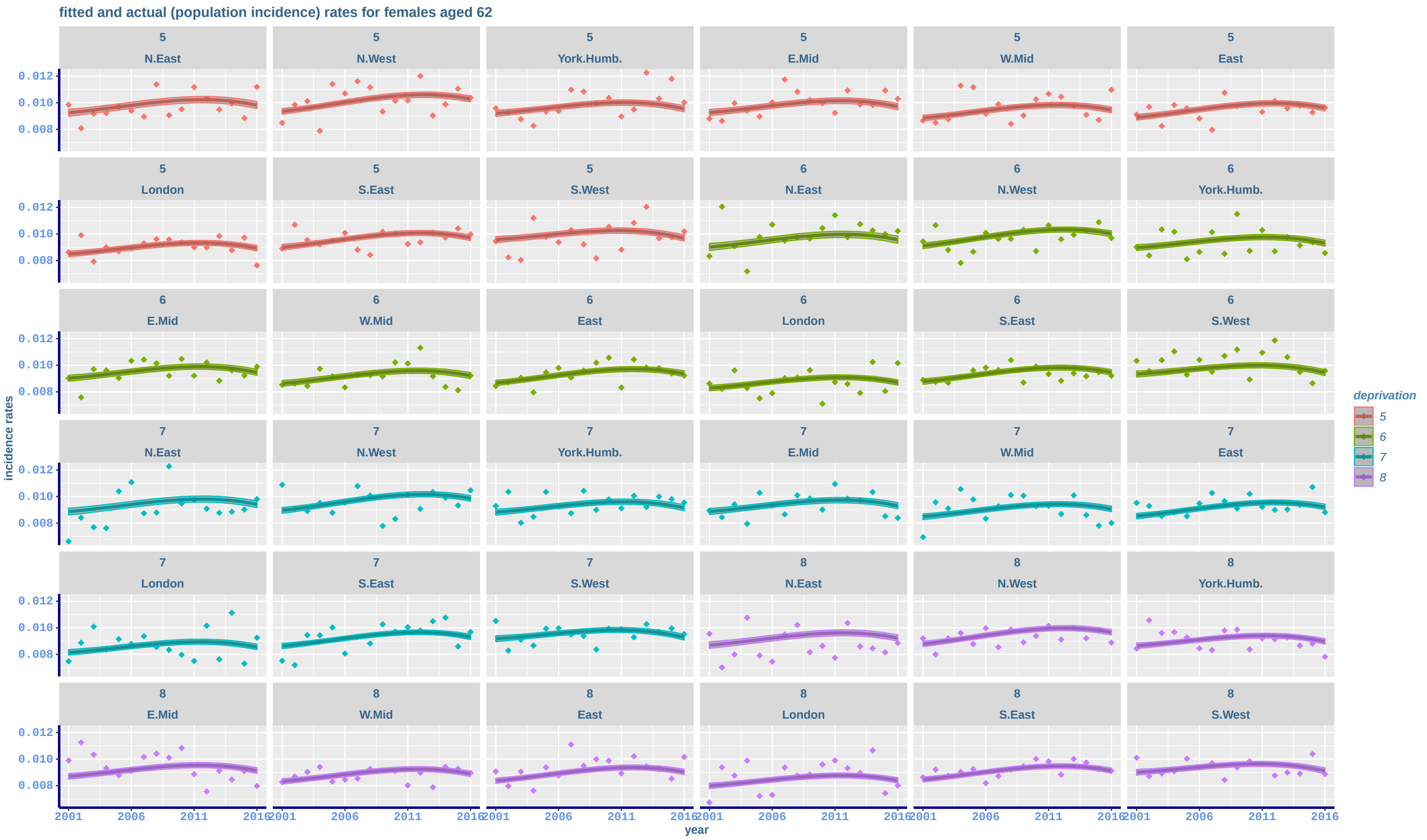

S10 Fig. All-cancer morbidity for females, age 62, deprivation deciles 5-8 for all regions in England between 2001 and 2016: observed rates (dots), fitted rates (lines), with 95% credible intervals for the fitted rates.

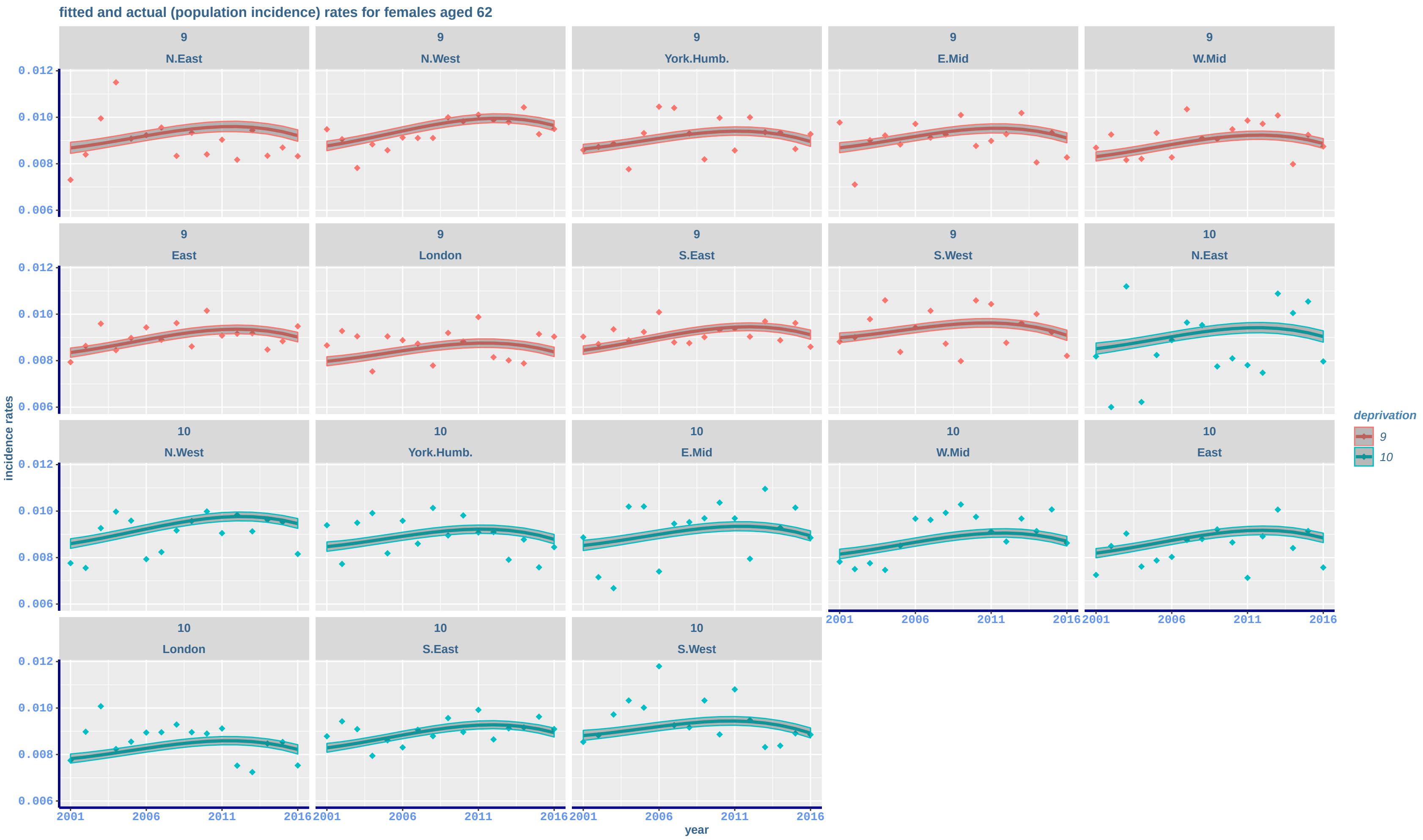

S11 Fig. All-cancer morbidity for females, age 62, deprivation deciles 9-10 for all regions in England between 2001 and 2016: observed rates (dots), fitted rates (lines), with 95% credible intervals for the fitted rates.

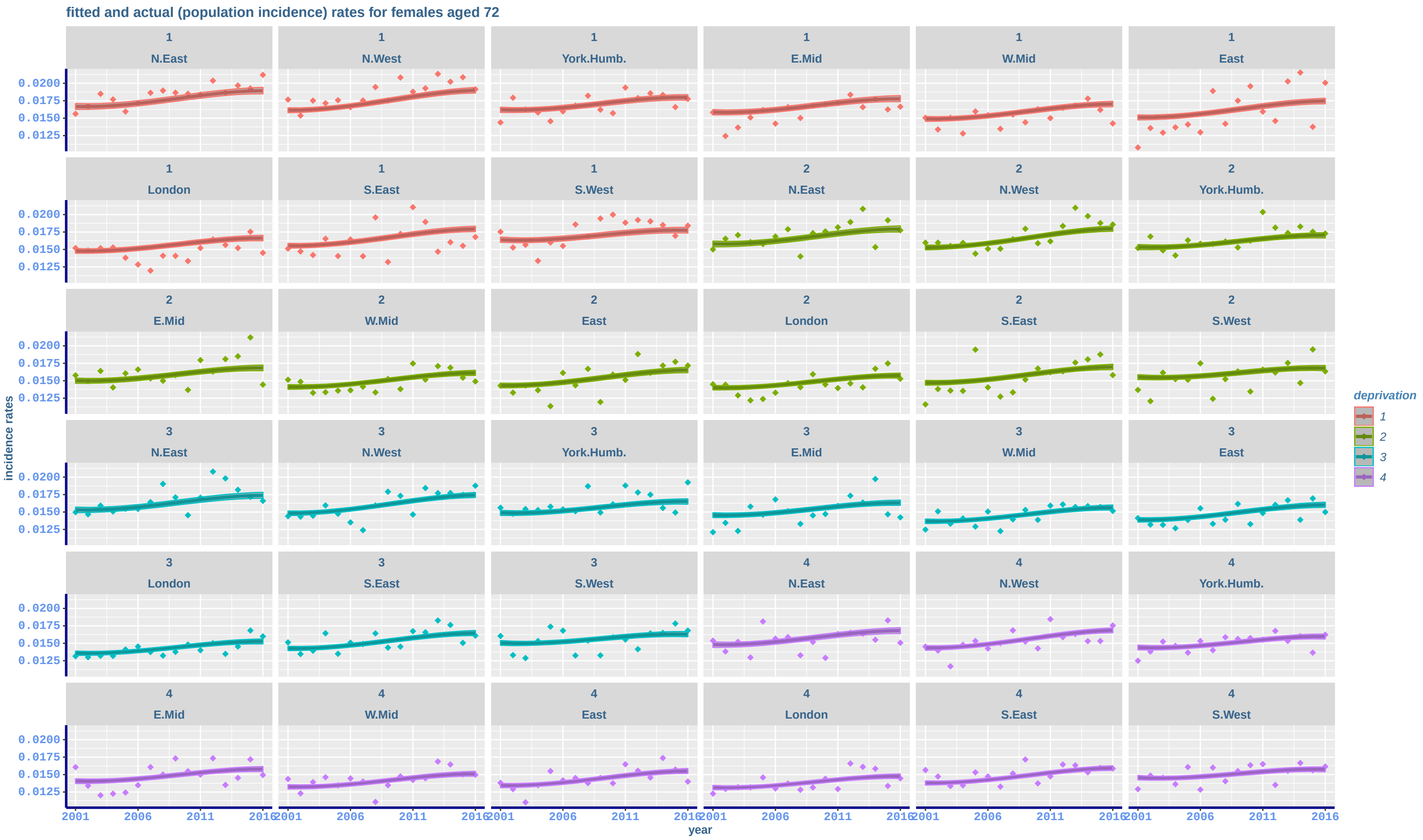

S12 Fig. All-cancer morbidity for females, age 72, deprivation deciles 1-4 for all regions in England between 2001 and 2016: observed rates (dots), fitted rates (lines), with 95% credible intervals for the fitted rates.

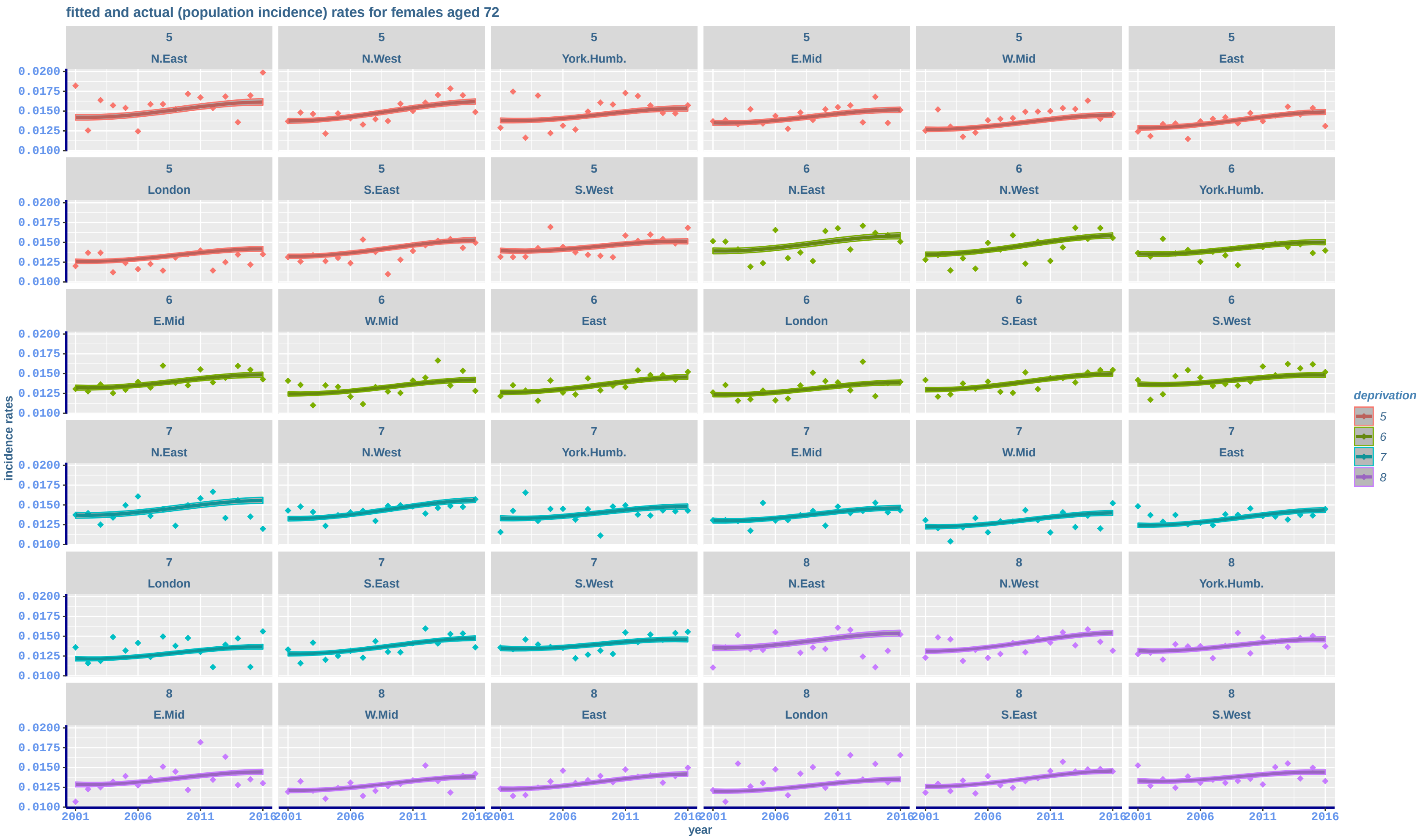

S13 Fig. All-cancer morbidity for females, age 72, deprivation deciles 5-8 for all regions in England between 2001 and 2016: observed rates (dots), fitted rates (lines), with 95% credible intervals for the fitted rates.

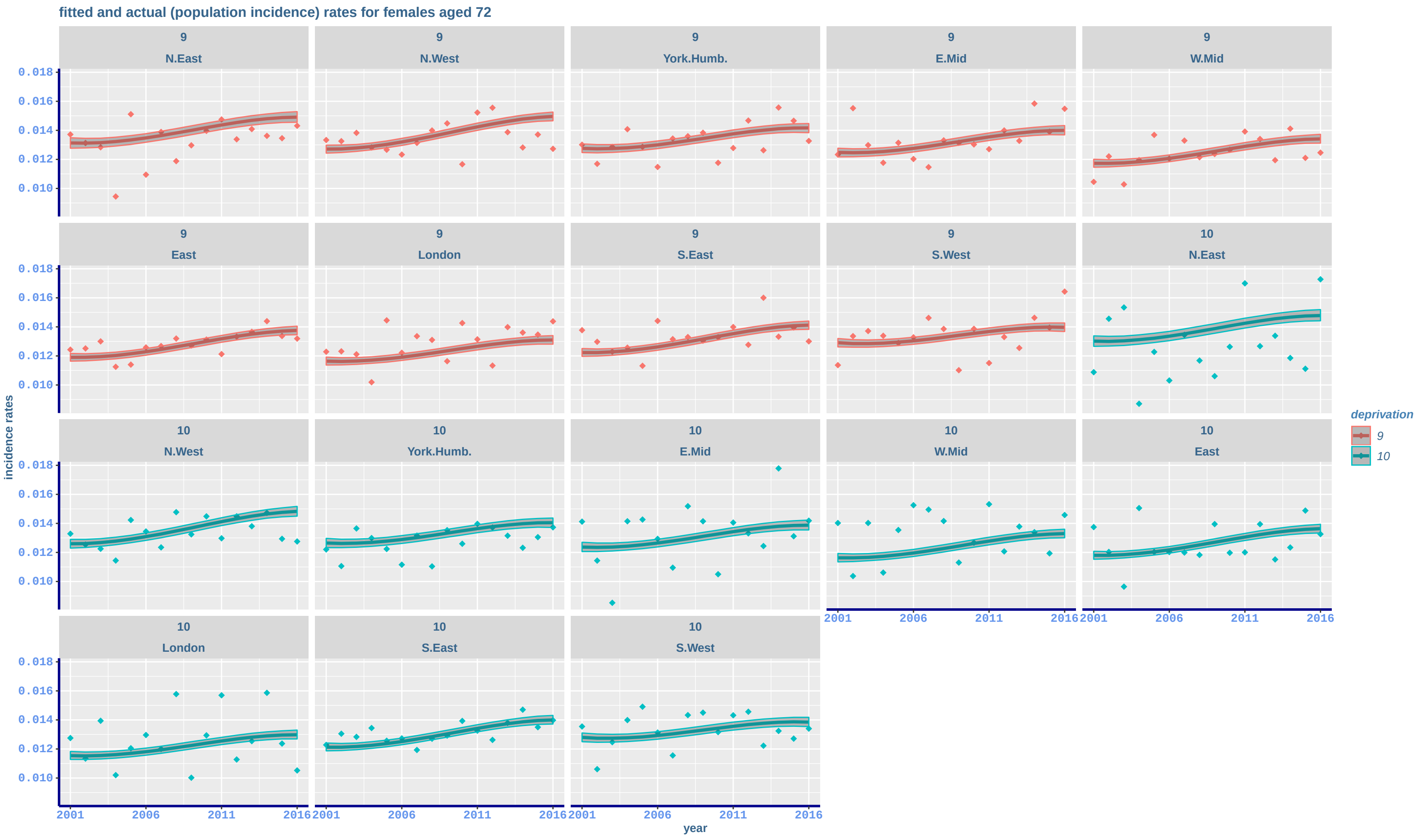

S14 Fig. All-cancer morbidity for females, age 72, deprivation deciles 9-10 for all regions in England between 2001 and 2016: observed rates (dots), fitted rates (lines), with 95% credible intervals for the fitted rates.

S5 Table. Absolute deprivation differences ($AD_{t,r}$), per 100,000 people, in age-standardised fitted incidence rates of all-cancer from 2001 to 2016 for all regions in England for males; 95% credible intervals in brackets

year	N.East	N.West	York.Humb.	E.Mid	W.Mid	East	London	S.East	S.West
2001	218	203	203	189	195	189	189	197	205
	(209, 226)	(195, 210)	(196, 211)	(181, 195)	(188, 202)	(182, 196)	(182, 196)	(190, 204)	(198, 213)
2002	214	203	203	190	194	189	188	195	204
	(206, 221)	(196, 210)	(195, 210)	(183, 196)	(187, 200)	(182, 196)	(182, 195)	(188, 202)	(197, 212)
2003	211	203	203	191	193	190	189	194	204
	(203, 218)	(196, 210)	(196, 210)	(184, 198)	(186, 200)	(183, 196)	(182, 195)	(188, 201)	(197, 211)
2004	209	204	204	193	194	191	190	194	205
	(202, 216)	(197, 211)	(196, 210)	(186, 200)	(187, 200)	(184, 198)	(183, 196)	(188, 201)	(197, 212)
2005	208	206	205	196	194	193	191	195	206
	(201, 216)	(199, 213)	(197, 212)	(189, 202)	(188, 201)	(186, 199)	(184, 197)	(188, 202)	(198, 213)
2006	208	208	206	198	196	195	193	197	207
	(201, 216)	(201, 215)	(199, 213)	(192, 205)	(189, 202)	(188, 202)	(186, 199)	(190, 203)	(200, 214)
2007	209	210	208	201	198	198	195	199	209
	(201, 216)	(203, 217)	(201, 215)	(194, 208)	(191, 204)	(191, 205)	(188, 201)	(192, 205)	(202, 216)
2008	209	212	210	203	199	201	197	201	211
	(202, 217)	(205, 219)	(202, 217)	(197, 210)	(193, 206)	(194, 207)	(190, 203)	(194, 208)	(203, 217)
2009	210	214	211	206	201	203	198	203	212
	(203, 218)	(207, 221)	(204, 218)	(199, 213)	(194, 208)	(196, 210)	(192, 205)	(196, 210)	(205, 219)
2010	212	216	212	207	203	205	200	206	213
	(204, 219)	(208, 223)	(205, 220)	(200, 214)	(196, 210)	(198, 212)	(193, 207)	(199, 213)	(206, 221)
2011	212	217	213	208	204	207	201	208	214
	(205, 220)	(209, 224)	(206, 220)	(201, 215)	(197, 211)	(200, 214)	(194, 208)	(201, 215)	(207, 221)
2012	213	217	213	208	205	209	201	210	214
	(206, 220)	(209, 224)	(206, 220)	(201, 215)	(198, 212)	(202, 216)	(195, 208)	(203, 217)	(207, 222)
2013	213	216	212	207	205	209	201	212	214
	(206, 220)	(209, 223)	(205, 219)	(201, 215)	(198, 212)	(202, 216)	(194, 208)	(204, 219)	(207, 221)
2014	213	214	210	205	204	209	200	213	212
	(205, 220)	(207, 222)	(203, 217)	(198, 212)	(197, 211)	(202, 216)	(193, 206)	(205, 220)	(205, 219)
2015	211	211	207	202	202	207	197	213	209
	(204, 219)	(204, 218)	(200, 214)	(195, 209)	(195, 209)	(200, 214)	(191, 204)	(205, 220)	(202, 217)
2016	209	207	203	197	200	205	194	212	205
	(202, 217)	(200, 214)	(196, 210)	(190, 204)	(192, 206)	(197, 212)	(187, 201)	(204, 219)	(198, 213)

S6 Table. Relative deprivation differences ($RD_{t,r}$) in age-standardised fitted incidence rates of all-cancer from 2001 to 2016 for all regions of England for males; 95% credible intervals in brackets

year	N.East	N.West	York.Humb.	E.Mid	W.Mid	East	London	S.East	S.West
2001	0.243637 (0.234299, 0.252601)	0.243638 (0.234296, 0.252536)	0.243639 (0.234257, 0.252606)	0.243638 (0.234311, 0.252577)	0.243639 (0.234226, 0.252602)	0.243639 (0.234266, 0.252562)	0.243637 (0.23426, 0.252509)	0.243641 (0.234319, 0.252687)	0.243636 (0.234302, 0.252584)
2002	0.24422 (0.234877, 0.253138)	0.244216 (0.234931, 0.253106)	0.244217 (0.234926, 0.25313)	0.244218 (0.234866, 0.253117)	0.244214 (0.234912, 0.253149)	0.244218 (0.23492, 0.253091)	0.244218 (0.234896, 0.253127)	0.244218 (0.234904, 0.253165)	0.244218 (0.234906, 0.25311)
2003	0.24475 (0.235511, 0.253676)	0.244746 (0.235482, 0.253626)	0.244749 (0.235483, 0.253608)	0.244748 (0.235461, 0.253545)	0.244748 (0.235474, 0.253634)	0.244751 (0.235493, 0.253634)	0.24475 (0.235467, 0.253682)	0.24475 (0.235495, 0.253643)	0.244746 (0.235482, 0.253577)
2004	0.245235 (0.236032, 0.254074)	0.245234 (0.235983, 0.254118)	0.245235 (0.236053, 0.254088)	0.245234 (0.236107, 0.254093)	0.245234 (0.23609, 0.254103)	0.245236 (0.236057, 0.254109)	0.245235 (0.235983, 0.254088)	0.245232 (0.236019, 0.254051)	0.245233 (0.236039, 0.254098)
2005	0.245676 (0.236496, 0.254458)	0.245677 (0.236499, 0.254539)	0.245673 (0.236572, 0.254472)	0.24567 (0.236611, 0.254503)	0.245672 (0.236533, 0.254435)	0.24567 (0.2365, 0.254482)	0.245674 (0.236564, 0.254414)	0.245675 (0.236521, 0.254475)	0.245675 (0.236603, 0.254475)
2006	0.246067 (0.236984, 0.254864)	0.246067 (0.236943, 0.254845)	0.246064 (0.236972, 0.25483)	0.246067 (0.236872, 0.254852)	0.246069 (0.236949, 0.25489)	0.246065 (0.236922, 0.254846)	0.246064 (0.236943, 0.254877)	0.246066 (0.237023, 0.254818)	0.246062 (0.236952, 0.254856)
2007	0.246413 (0.237345, 0.255202)	0.246417 (0.237453, 0.255264)	0.246417 (0.237409, 0.255159)	0.246417 (0.237344, 0.255179)	0.246419 (0.237329, 0.255226)	0.246418 (0.237382, 0.255131)	0.246416 (0.237333, 0.255198)	0.246416 (0.237349, 0.255226)	0.246415 (0.237386, 0.255153)
2008	0.24672 (0.237718, 0.255508)	0.246719 (0.237651, 0.255533)	0.246715 (0.237668, 0.255475)	0.246718 (0.237657, 0.255517)	0.246719 (0.237705, 0.255492)	0.246723 (0.237727, 0.255495)	0.24672 (0.237671, 0.255508)	0.24672 (0.237672, 0.255455)	0.246721 (0.237717, 0.255528)
2009	0.24698 (0.238039, 0.255773)	0.246979 (0.238008, 0.255739)	0.24698 (0.23798, 0.255803)	0.246982 (0.23791, 0.25577)	0.246979 (0.237932, 0.255778)	0.246979 (0.238026, 0.255802)	0.246979 (0.238058, 0.255737)	0.24698 (0.237965, 0.255766)	0.246981 (0.237996, 0.255739)
2010	0.247196 (0.238165, 0.255983)	0.247195 (0.238114, 0.255983)	0.247196 (0.238145, 0.255943)	0.247197 (0.238228, 0.25596)	0.247197 (0.238201, 0.256011)	0.247198 (0.238113, 0.255989)	0.247195 (0.238141, 0.255982)	0.247197 (0.238173, 0.256021)	0.2472 (0.238201, 0.255978)
2011	0.247373 (0.238291, 0.256133)	0.247371 (0.238324, 0.25619)	0.247372 (0.238311, 0.256125)	0.247368 (0.238368, 0.25619)	0.247371 (0.238321, 0.256168)	0.247368 (0.238344, 0.256175)	0.247372 (0.23833, 0.256164)	0.247371 (0.238301, 0.256223)	0.247371 (0.238293, 0.25618)
2012	0.2475 (0.238517, 0.256338)	0.2475 (0.238434, 0.256234)	0.247502 (0.238416, 0.256348)	0.247501 (0.238507, 0.256266)	0.247499 (0.238445, 0.256283)	0.2475 (0.238423, 0.256222)	0.247499 (0.238399, 0.256185)	0.2475 (0.238418, 0.256243)	0.2475 (0.238487, 0.256235)
2013	0.247586 (0.238455, 0.256361)	0.247589 (0.238514, 0.25638)	0.247589 (0.238467, 0.256437)	0.247587 (0.238551, 0.25637)	0.247588 (0.238533, 0.256401)	0.247589 (0.238536, 0.256394)	0.247588 (0.238529, 0.256418)	0.24759 (0.238489, 0.256456)	0.247585 (0.238479, 0.256389)
2014	0.247631 (0.238524, 0.256392)	0.247635 (0.238504, 0.2564)	0.247632 (0.238507, 0.256437)	0.247635 (0.238507, 0.256415)	0.247636 (0.238476, 0.256447)	0.24763 (0.238531, 0.256412)	0.247635 (0.238499, 0.256402)	0.24763 (0.238552, 0.256429)	0.247633 (0.238518, 0.2564)
2015	0.24763 (0.238424, 0.256447)	0.247634 (0.238489, 0.256399)	0.247631 (0.23844, 0.256431)	0.247637 (0.238505, 0.256446)	0.247631 (0.238407, 0.256429)	0.247632 (0.238451, 0.256453)	0.247634 (0.238389, 0.256435)	0.247634 (0.238471, 0.256406)	0.24763 (0.238437, 0.256452)
2016	0.247589 (0.238414, 0.256413)	0.247587 (0.238362, 0.256365)	0.247589 (0.238374, 0.256323)	0.247588 (0.238368, 0.256407)	0.24759 (0.238372, 0.256475)	0.247591 (0.238441, 0.256389)	0.247592 (0.238332, 0.256369)	0.24759 (0.238353, 0.256415)	0.24759 (0.238308, 0.256389)

S7 Table. Absolute deprivation differences ($AD_{t,r}$), per 100,000 people, in age-standardised fitted incidence rates of all-cancer from 2001 to 2016 for all regions in England for females; 95% credible intervals in brackets

year	N.East	N.West	York.Humb.	E.Mid	W.Mid	East	London	S.East	S.West
2001	113 (108, 118)	109 (104, 115)	110 (105, 115)	109 (104, 114)	104 (99, 109)	105 (100, 110)	102 (97, 107)	107 (101, 112)	113 (108, 118)
2002	114 (108, 119)	110 (105, 115)	111 (105, 116)	109 (104, 115)	104 (99, 109)	105 (100, 110)	102 (98, 107)	107 (102, 112)	113 (108, 118)
2003	115 (109, 120)	111 (106, 116)	111 (106, 116)	110 (105, 115)	105 (100, 110)	106 (101, 111)	103 (98, 108)	108 (103, 113)	113 (108, 119)
2004	116 (110, 121)	113 (107, 118)	112 (107, 117)	111 (106, 116)	106 (101, 111)	107 (102, 112)	104 (99, 109)	109 (104, 114)	114 (109, 120)
2005	117 (111, 122)	114 (109, 120)	113 (108, 118)	112 (107, 117)	107 (102, 112)	109 (103, 114)	105 (100, 110)	110 (105, 116)	115 (110, 120)
2006	118 (113, 124)	116 (111, 121)	114 (109, 120)	113 (108, 119)	108 (103, 113)	110 (105, 115)	106 (101, 111)	112 (107, 117)	116 (110, 121)
2007	120 (114, 125)	117 (112, 123)	115 (110, 121)	115 (109, 120)	110 (105, 115)	111 (106, 117)	107 (102, 112)	113 (108, 119)	117 (112, 123)
2008	121 (116, 127)	119 (114, 125)	117 (111, 122)	116 (110, 121)	111 (106, 116)	113 (108, 118)	109 (103, 114)	115 (110, 120)	118 (112, 124)
2009	123 (117, 128)	121 (115, 126)	118 (112, 123)	117 (112, 123)	112 (107, 118)	114 (109, 120)	110 (105, 115)	116 (111, 122)	119 (113, 125)
2010	124 (118, 129)	122 (117, 128)	119 (113, 124)	118 (113, 124)	114 (108, 119)	116 (110, 121)	111 (106, 116)	118 (112, 123)	120 (114, 126)
2011	125 (119, 131)	124 (118, 129)	120 (114, 125)	119 (113, 125)	115 (109, 120)	117 (111, 122)	112 (107, 117)	119 (113, 125)	121 (115, 126)
2012	126 (120, 131)	125 (119, 131)	120 (115, 126)	120 (114, 125)	116 (110, 121)	118 (112, 123)	112 (107, 117)	120 (114, 125)	121 (115, 127)
2013	126 (120, 132)	126 (120, 131)	121 (115, 126)	120 (114, 126)	116 (111, 121)	118 (113, 124)	113 (107, 118)	120 (115, 126)	121 (115, 127)
2014	126 (120, 132)	126 (120, 132)	120 (115, 126)	120 (114, 126)	116 (111, 122)	118 (113, 124)	113 (107, 118)	121 (115, 126)	121 (115, 126)
2015	126 (120, 132)	126 (120, 132)	120 (114, 125)	120 (114, 125)	116 (110, 121)	118 (113, 124)	112 (107, 118)	120 (115, 126)	120 (114, 126)
2016	125 (119, 131)	125 (119, 131)	119 (113, 124)	119 (113, 124)	115 (110, 120)	118 (112, 123)	111 (106, 117)	120 (114, 125)	119 (113, 124)

S8 Table. Relative deprivation differences ($RD_{t,r}$) in age-standardised fitted incidence rates of all-cancer from 2001 to 2016 for all regions in England for females; 95% credible intervals in brackets

year	N.East	N.West	York.Humb.	E.Mid	W.Mid	East	London	S.East	S.West
2001	0.1736 (0.1651, 0.1822)	0.1737 (0.1652, 0.1823)	0.1724 (0.164, 0.1809)	0.1731 (0.1645, 0.1817)	0.1728 (0.1644, 0.1814)	0.174 (0.1654, 0.1827)	0.1738 (0.1653, 0.1824)	0.1732 (0.1648, 0.1819)	0.1715 (0.1632, 0.1802)
2002	0.1738 (0.1653, 0.1825)	0.1739 (0.1653, 0.1824)	0.1726 (0.1643, 0.1811)	0.1733 (0.1647, 0.1819)	0.173 (0.1646, 0.1816)	0.1742 (0.1657, 0.1828)	0.174 (0.1655, 0.1827)	0.1734 (0.1649, 0.1821)	0.1717 (0.1633, 0.1803)
2003	0.1739 (0.1655, 0.1826)	0.174 (0.1655, 0.1826)	0.1727 (0.1642, 0.1813)	0.1734 (0.1649, 0.182)	0.1732 (0.1648, 0.1817)	0.1744 (0.1657, 0.183)	0.1742 (0.1657, 0.1827)	0.1736 (0.1652, 0.1822)	0.1719 (0.1634, 0.1804)
2004	0.174 (0.1655, 0.1827)	0.1741 (0.1656, 0.1827)	0.1728 (0.1644, 0.1814)	0.1735 (0.165, 0.1821)	0.1733 (0.1649, 0.1818)	0.1745 (0.166, 0.1831)	0.1743 (0.1657, 0.1829)	0.1737 (0.1652, 0.1822)	0.172 (0.1635, 0.1805)
2005	0.1741 (0.1655, 0.1826)	0.1742 (0.1656, 0.1826)	0.1728 (0.1645, 0.1814)	0.1736 (0.1651, 0.1822)	0.1733 (0.165, 0.1818)	0.1746 (0.166, 0.1832)	0.1744 (0.1658, 0.183)	0.1738 (0.1652, 0.1824)	0.172 (0.1635, 0.1805)
2006	0.1741 (0.1655, 0.1827)	0.1742 (0.1656, 0.1827)	0.1729 (0.1644, 0.1814)	0.1736 (0.165, 0.1822)	0.1733 (0.165, 0.1819)	0.1746 (0.1659, 0.1832)	0.1744 (0.1659, 0.183)	0.1738 (0.1652, 0.1824)	0.172 (0.1635, 0.1805)
2007	0.1741 (0.1655, 0.1826)	0.1741 (0.1656, 0.1826)	0.1728 (0.1644, 0.1814)	0.1736 (0.165, 0.1821)	0.1733 (0.1649, 0.1818)	0.1746 (0.166, 0.1831)	0.1744 (0.1659, 0.183)	0.1737 (0.1652, 0.1824)	0.172 (0.1634, 0.1804)
2008	0.174 (0.1653, 0.1825)	0.174 (0.1655, 0.1826)	0.1727 (0.1643, 0.1812)	0.1735 (0.1649, 0.182)	0.1732 (0.1649, 0.1817)	0.1745 (0.1659, 0.183)	0.1744 (0.1657, 0.1829)	0.1737 (0.1652, 0.1822)	0.1719 (0.1633, 0.1803)
2009	0.1738 (0.1654, 0.1823)	0.1739 (0.1654, 0.1823)	0.1726 (0.1641, 0.1811)	0.1733 (0.1648, 0.1819)	0.1731 (0.1647, 0.1816)	0.1743 (0.1657, 0.183)	0.1742 (0.1657, 0.1828)	0.1735 (0.165, 0.1821)	0.1717 (0.1632, 0.1802)
2010	0.1736 (0.1651, 0.1821)	0.1737 (0.1651, 0.1822)	0.1724 (0.1639, 0.1809)	0.1731 (0.1646, 0.1816)	0.1729 (0.1645, 0.1813)	0.1742 (0.1656, 0.1827)	0.1741 (0.1655, 0.1826)	0.1733 (0.1648, 0.1819)	0.1715 (0.1631, 0.18)
2011	0.1734 (0.1648, 0.182)	0.1735 (0.1649, 0.1819)	0.1721 (0.1636, 0.1806)	0.1729 (0.1643, 0.1814)	0.1726 (0.1642, 0.1811)	0.1739 (0.1654, 0.1825)	0.1738 (0.1653, 0.1824)	0.1731 (0.1646, 0.1817)	0.1713 (0.1628, 0.1798)
2012	0.1731 (0.1645, 0.1816)	0.1732 (0.1647, 0.1816)	0.1718 (0.1634, 0.1803)	0.1726 (0.1641, 0.1811)	0.1723 (0.164, 0.1808)	0.1737 (0.1651, 0.1823)	0.1736 (0.165, 0.1822)	0.1728 (0.1643, 0.1813)	0.171 (0.1624, 0.1794)
2013	0.1728 (0.1641, 0.1812)	0.1728 (0.1643, 0.1812)	0.1715 (0.163, 0.18)	0.1722 (0.1637, 0.1807)	0.172 (0.1637, 0.1804)	0.1733 (0.1648, 0.1819)	0.1733 (0.1647, 0.1818)	0.1725 (0.164, 0.181)	0.1706 (0.1621, 0.1791)
2014	0.1724 (0.1637, 0.1809)	0.1724 (0.1638, 0.1808)	0.1711 (0.1626, 0.1796)	0.1719 (0.1633, 0.1804)	0.1716 (0.1632, 0.1801)	0.173 (0.1645, 0.1816)	0.1729 (0.1644, 0.1814)	0.1721 (0.1637, 0.1807)	0.1702 (0.1617, 0.1787)
2015	0.1719 (0.1633, 0.1804)	0.172 (0.1634, 0.1804)	0.1707 (0.1622, 0.1792)	0.1714 (0.1629, 0.1799)	0.1712 (0.1628, 0.1797)	0.1725 (0.164, 0.1811)	0.1725 (0.1639, 0.181)	0.1717 (0.1633, 0.1803)	0.1698 (0.1613, 0.1783)
2016	0.1715 (0.1628, 0.1799)	0.1715 (0.1629, 0.18)	0.1702 (0.1617, 0.1787)	0.1709 (0.1624, 0.1795)	0.1707 (0.1623, 0.1791)	0.172 (0.1636, 0.1807)	0.172 (0.1634, 0.1806)	0.1712 (0.1628, 0.1798)	0.1693 (0.1608, 0.1778)

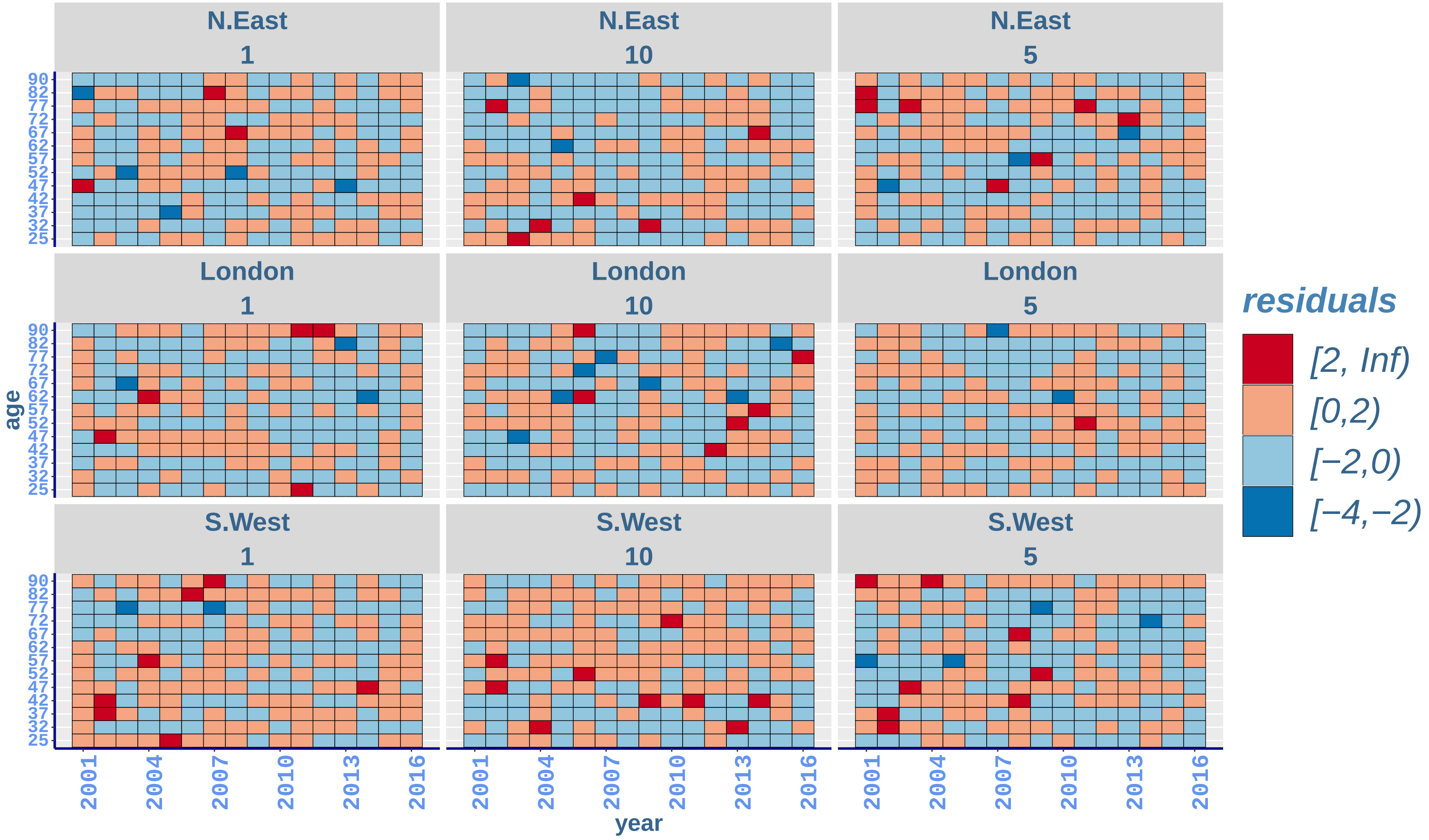

S15 Fig. Heat map of Pearson residuals for all-cancer mortality for males in North East, London, and South West, deprivation deciles 1, 5, and 10: orange/light blue cells indicate areas with good fit, while red/dark blue cells indicate areas with poor fit. Note that there is a small number of residuals greater than 4, and these are included in the last category.

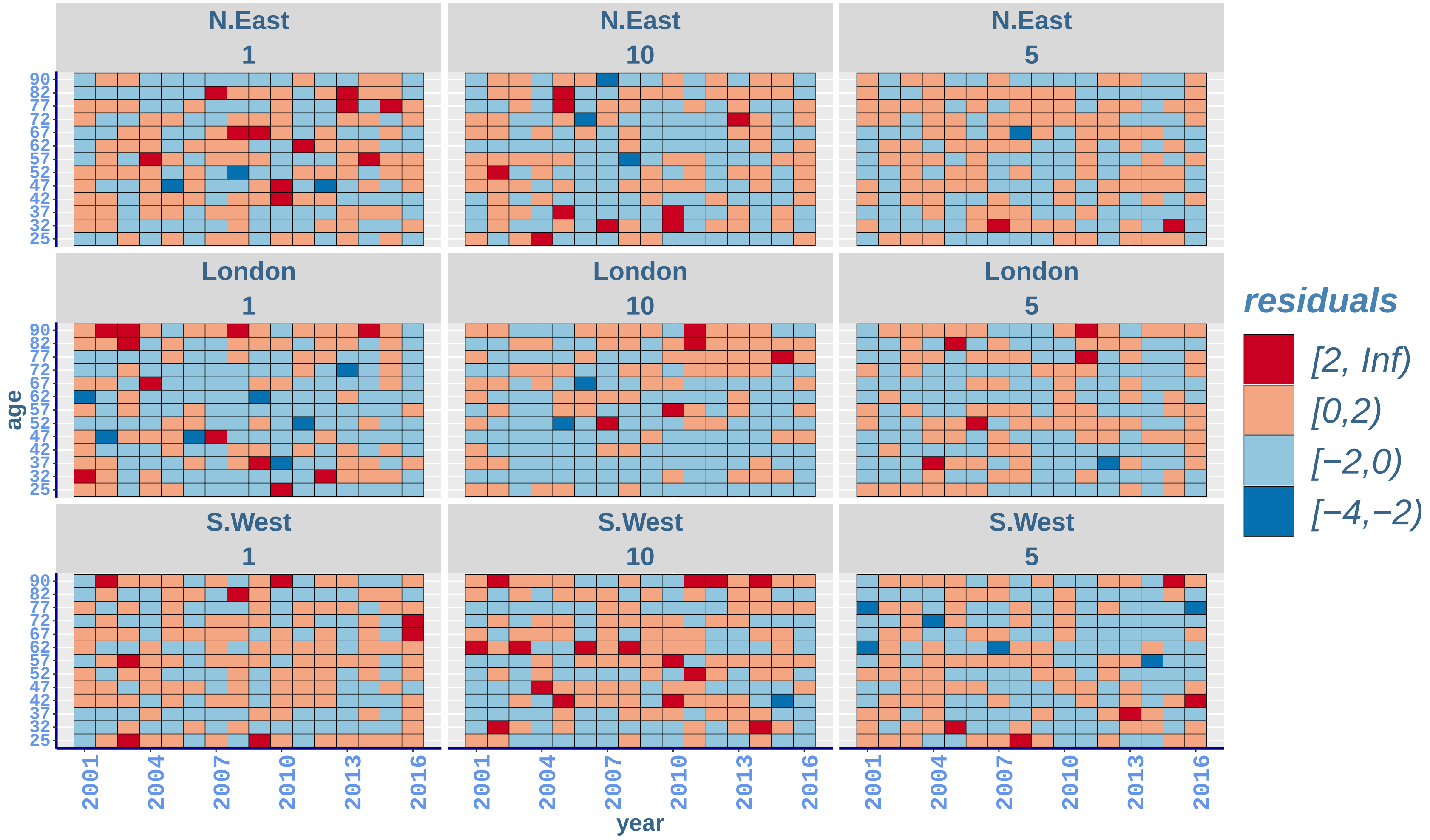

S16 Fig. Heat map of Pearson residuals for all-cancer mortality for females in North East, London, and South West, deprivation deciles 1, 5, and 10: orange/light blue cells indicate areas with good fit, while red/dark blue cells indicate areas with poor fit. Note that there is a small number of residuals greater than 4, and these are included in the last category.

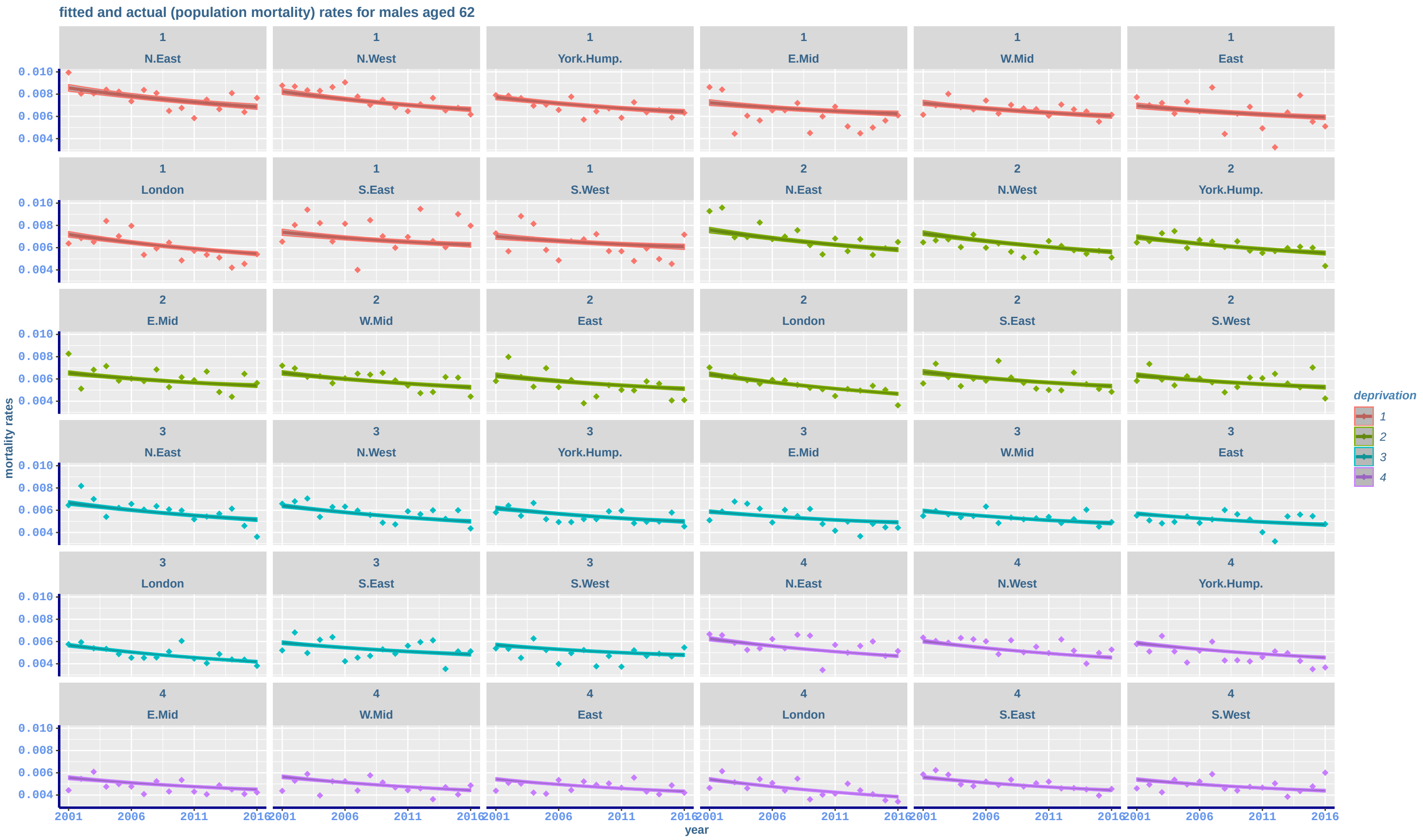

S17 Fig. All-cancer mortality for males, age 62, deprivation deciles 1-4 for all regions in England between 2001 and 2016: observed rates (dots), fitted rates (lines), with 95% credible intervals for the fitted rates.

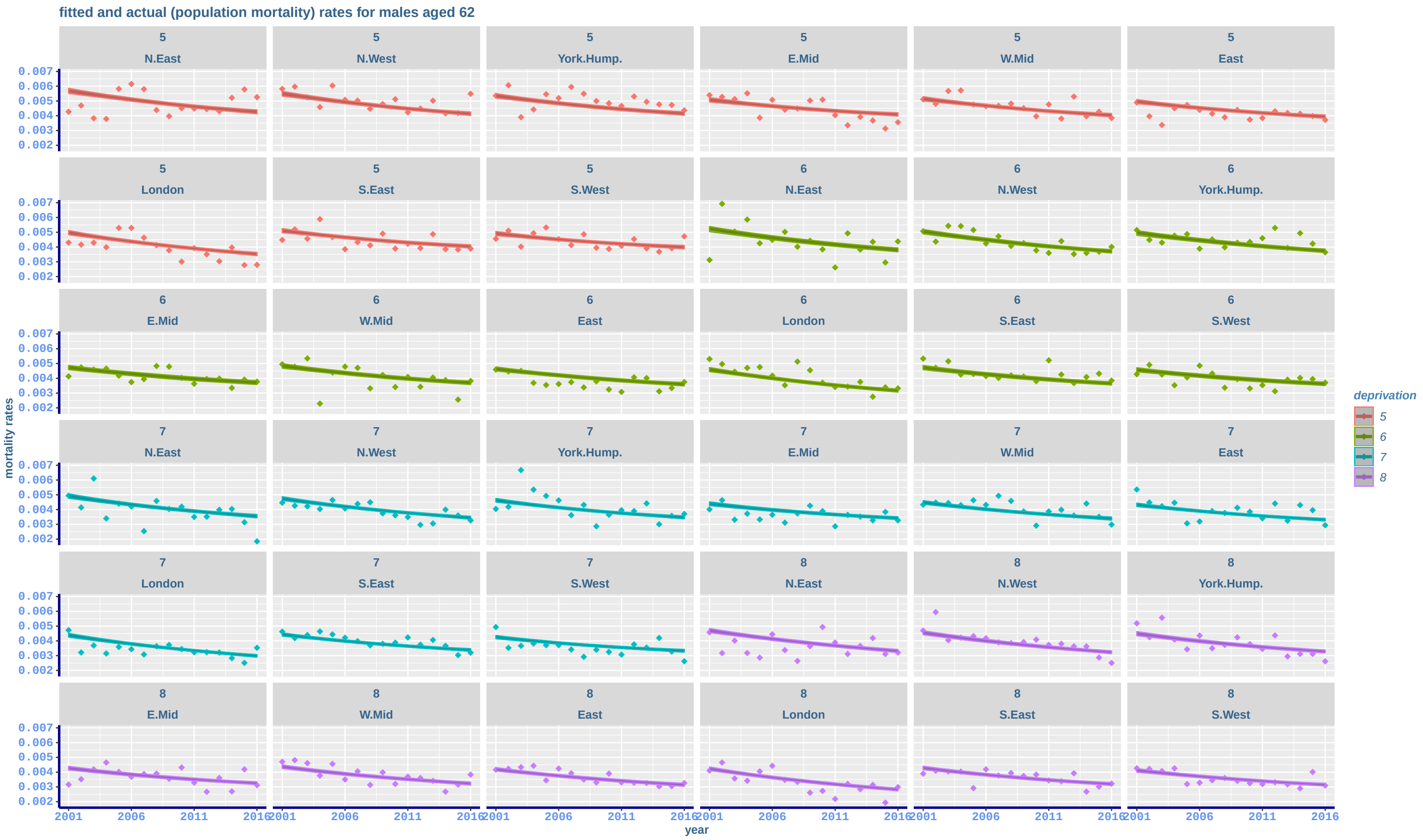

S18 Fig. All-cancer mortality for males, age 62, deprivation deciles 5-8 for all regions in England between 2001 and 2016: observed rates (dots), fitted rates (lines), with 95% credible intervals for the fitted rates.

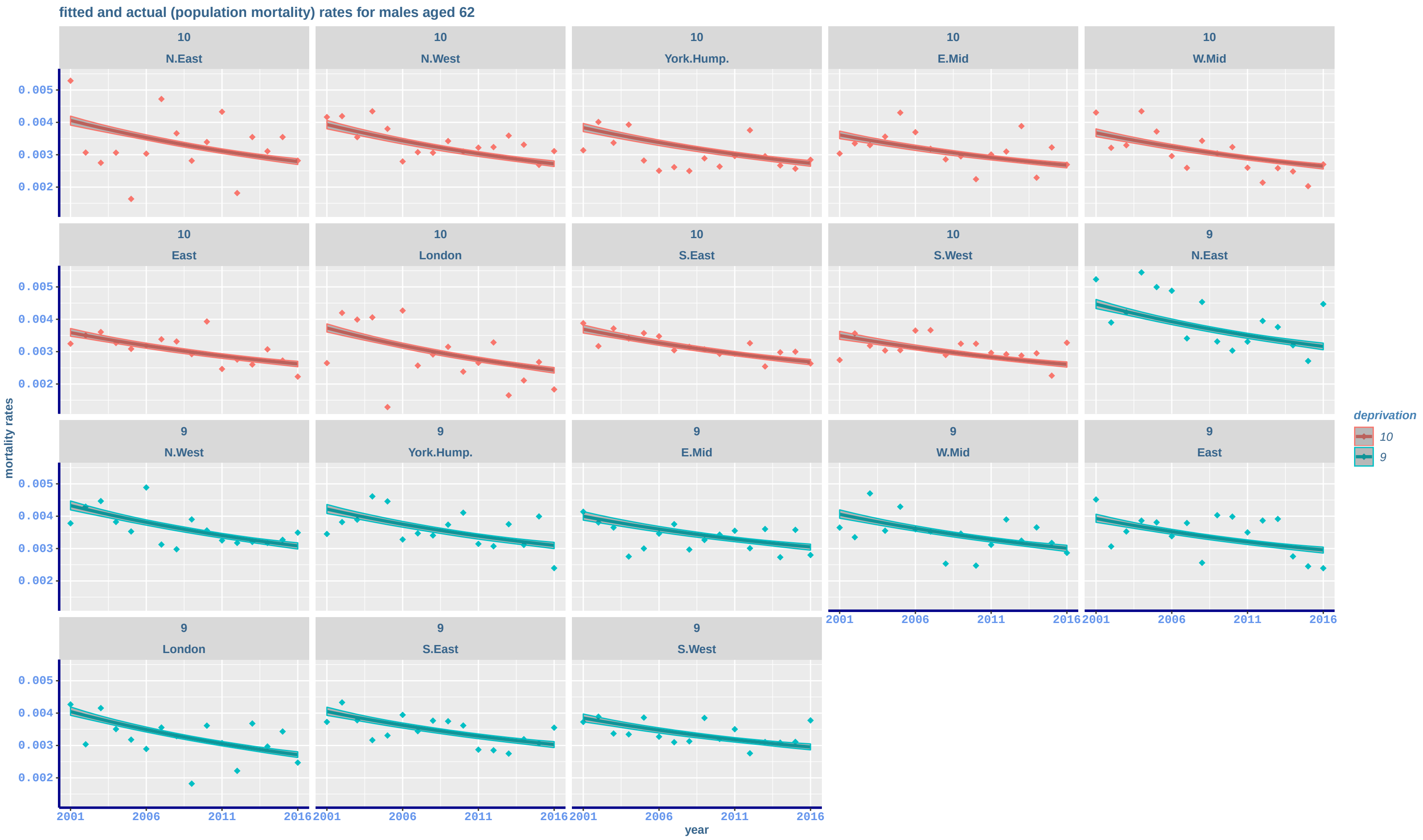

S19 Fig. All-cancer mortality for males, age 62, deprivation deciles 9-10 for all regions in England between 2001 and 2016: observed rates (dots), fitted rates (lines), with 95% credible intervals for the fitted rates.

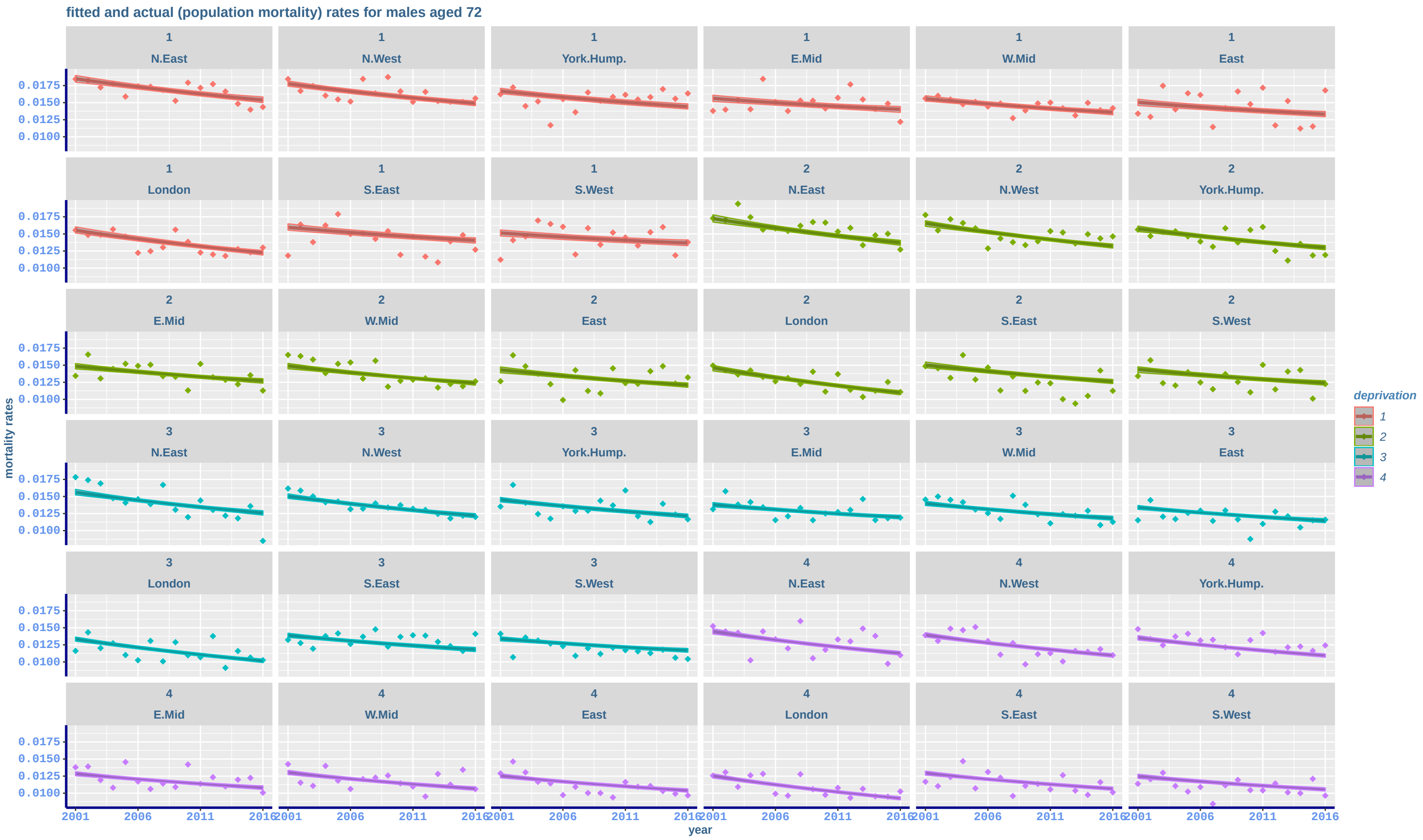

S20 Fig. All-cancer mortality for males, age 72, deprivation deciles 1-4 for all regions in England between 2001 and 2016: observed rates (dots), fitted rates (lines), with 95% credible intervals for the fitted rates.

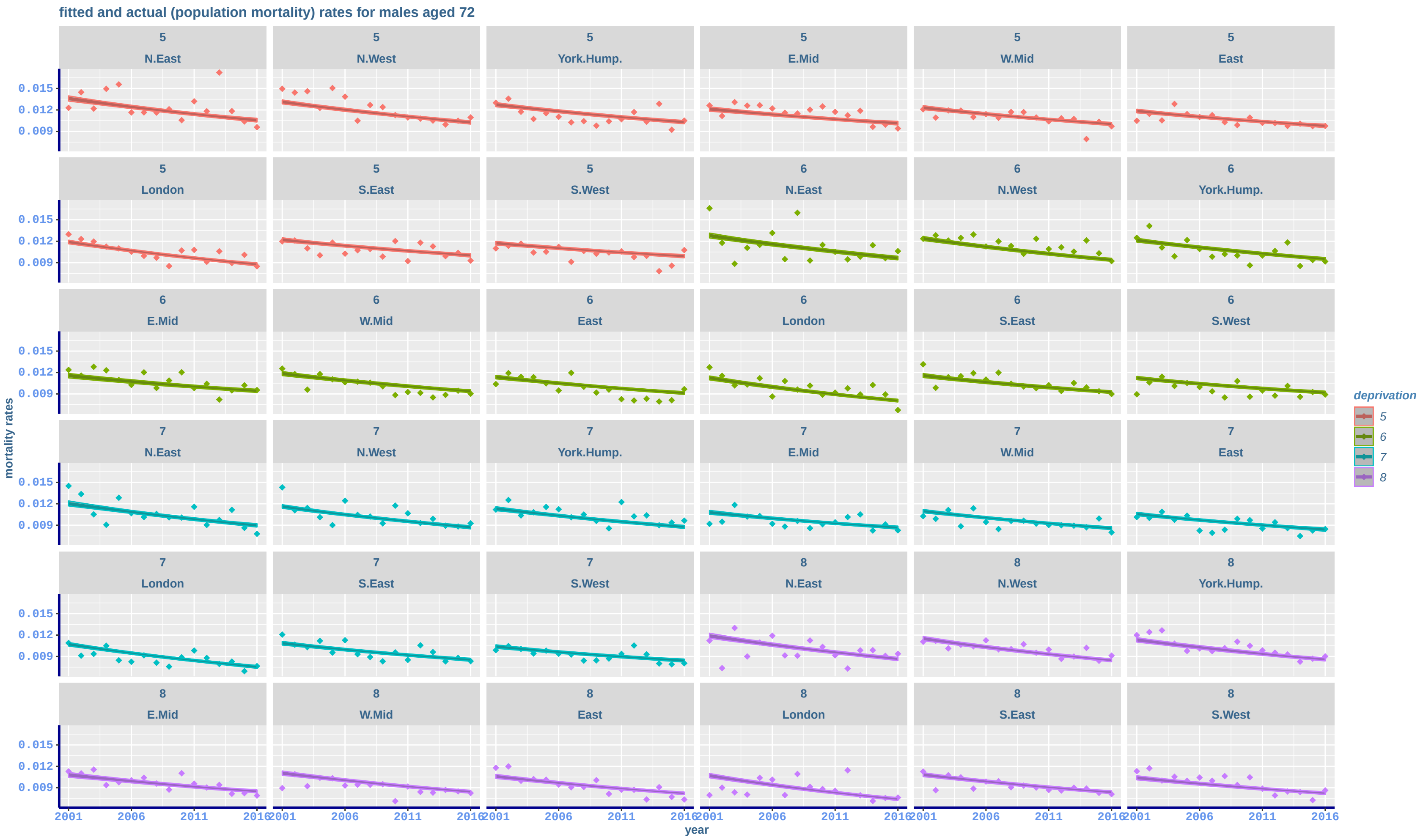

S21 Fig. All-cancer mortality for males, age 72, deprivation deciles 5-8 for all regions in England between 2001 and 2016: observed rates (dots), fitted rates (lines), with 95% credible intervals for the fitted rates.

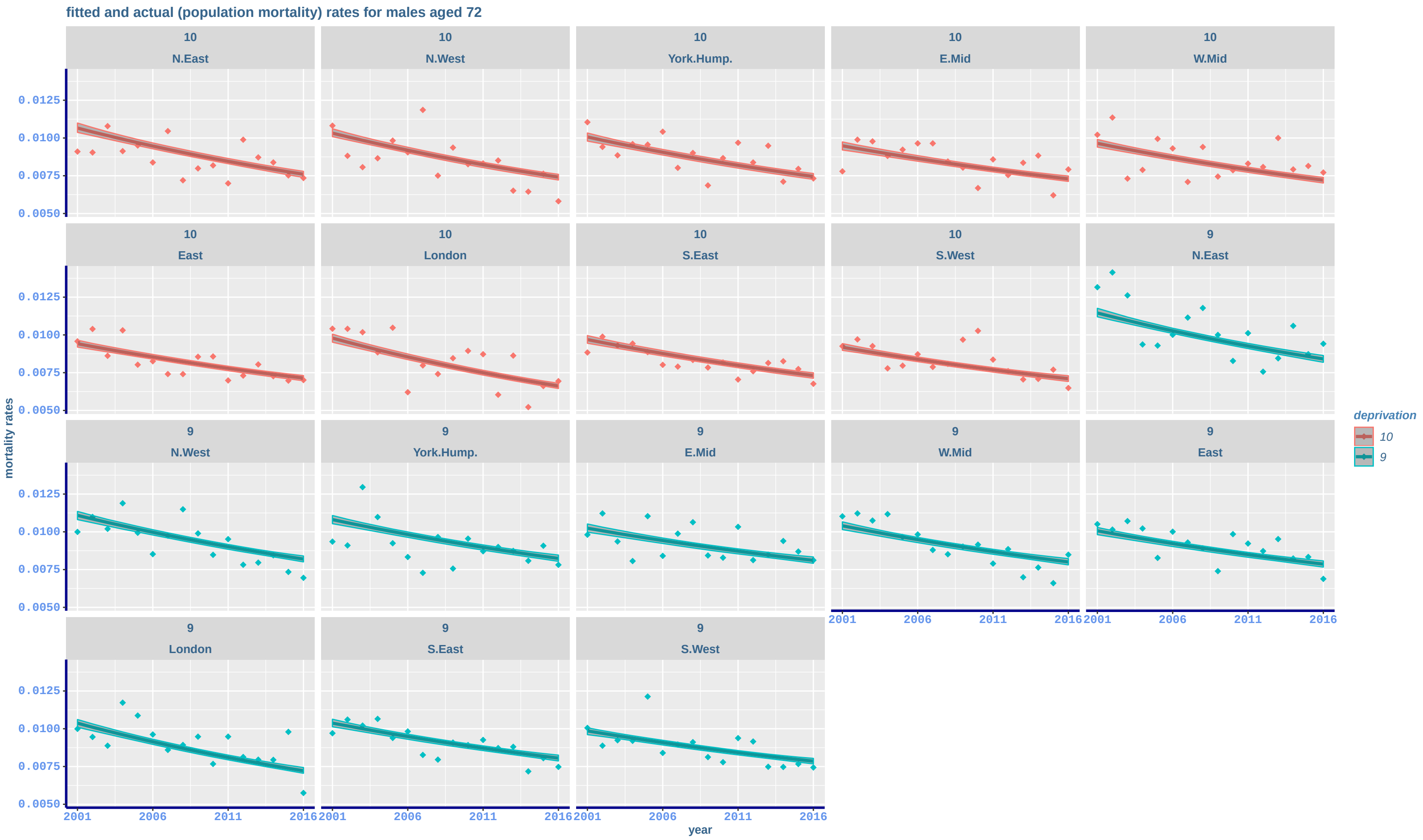

S22 Fig. All-cancer mortality for males, age 72, deprivation deciles 9-10 for all regions in England between 2001 and 2016: observed rates (dots), fitted rates (lines), with 95% credible intervals for the fitted rates.

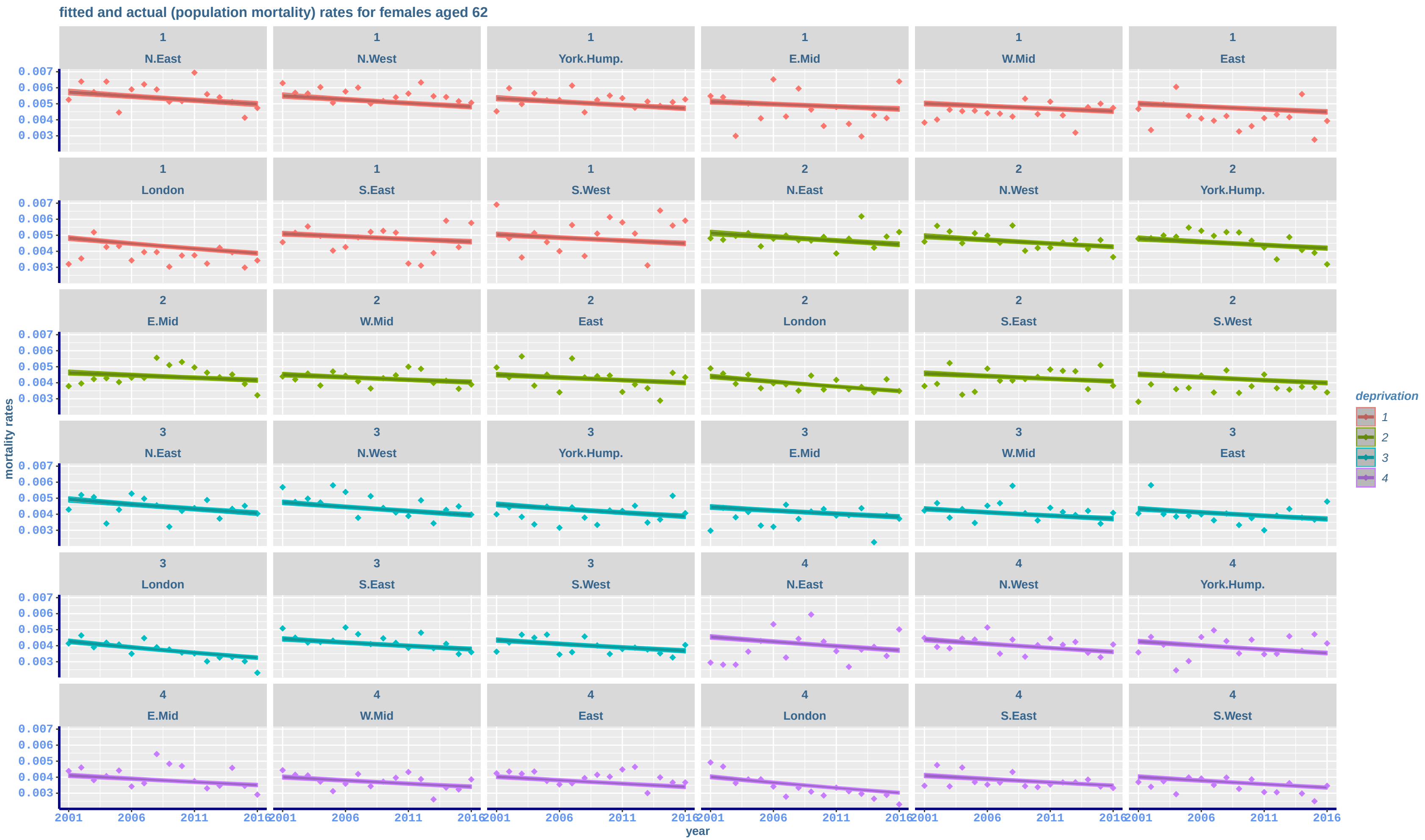

S23 Fig. All-cancer mortality for females, age 62, deprivation deciles 1-4 for all regions in England between 2001 and 2016: observed rates (dots), fitted rates (lines), with 95% credible intervals for the fitted rates.

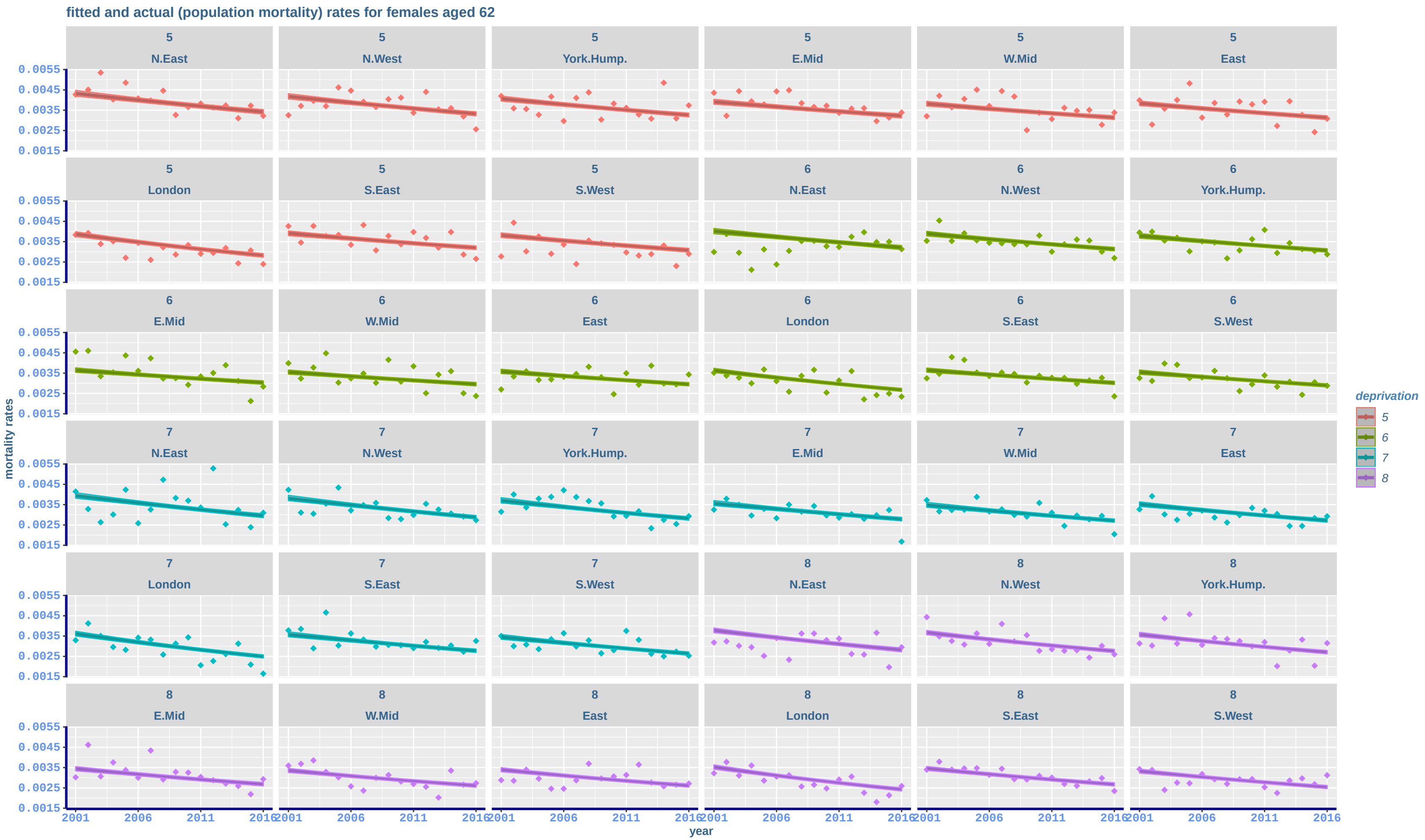

S24 Fig. All-cancer mortality for females, age 62, deprivation deciles 5-8 for all regions in England between 2001 and 2016: observed rates (dots), fitted rates (lines), with 95% credible intervals for the fitted rates.

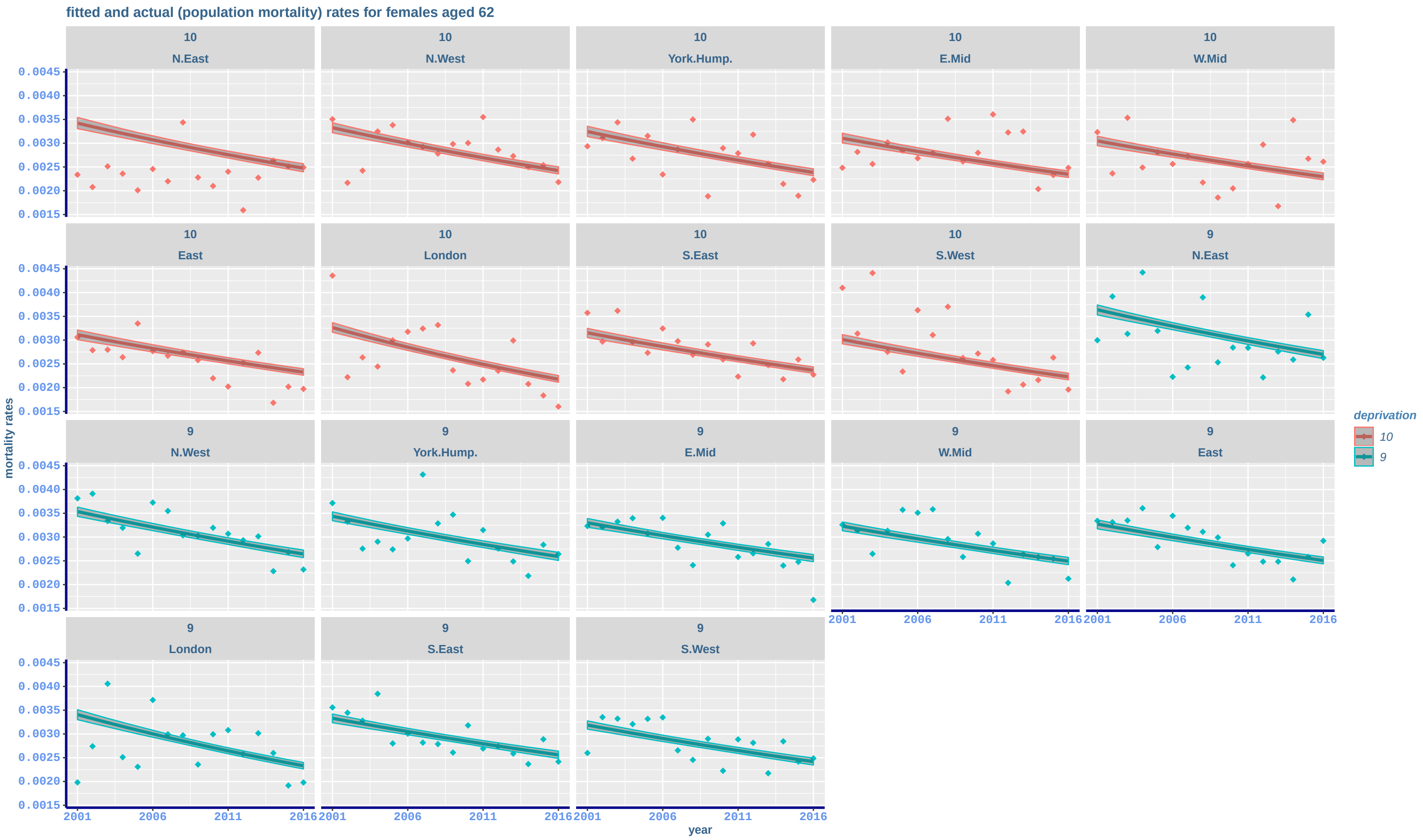

S25 Fig. All-cancer mortality for females, age 62, deprivation deciles 9-10 for all regions in England between 2001 and 2016: observed rates (dots), fitted rates (lines), with 95% credible intervals for the fitted rates.

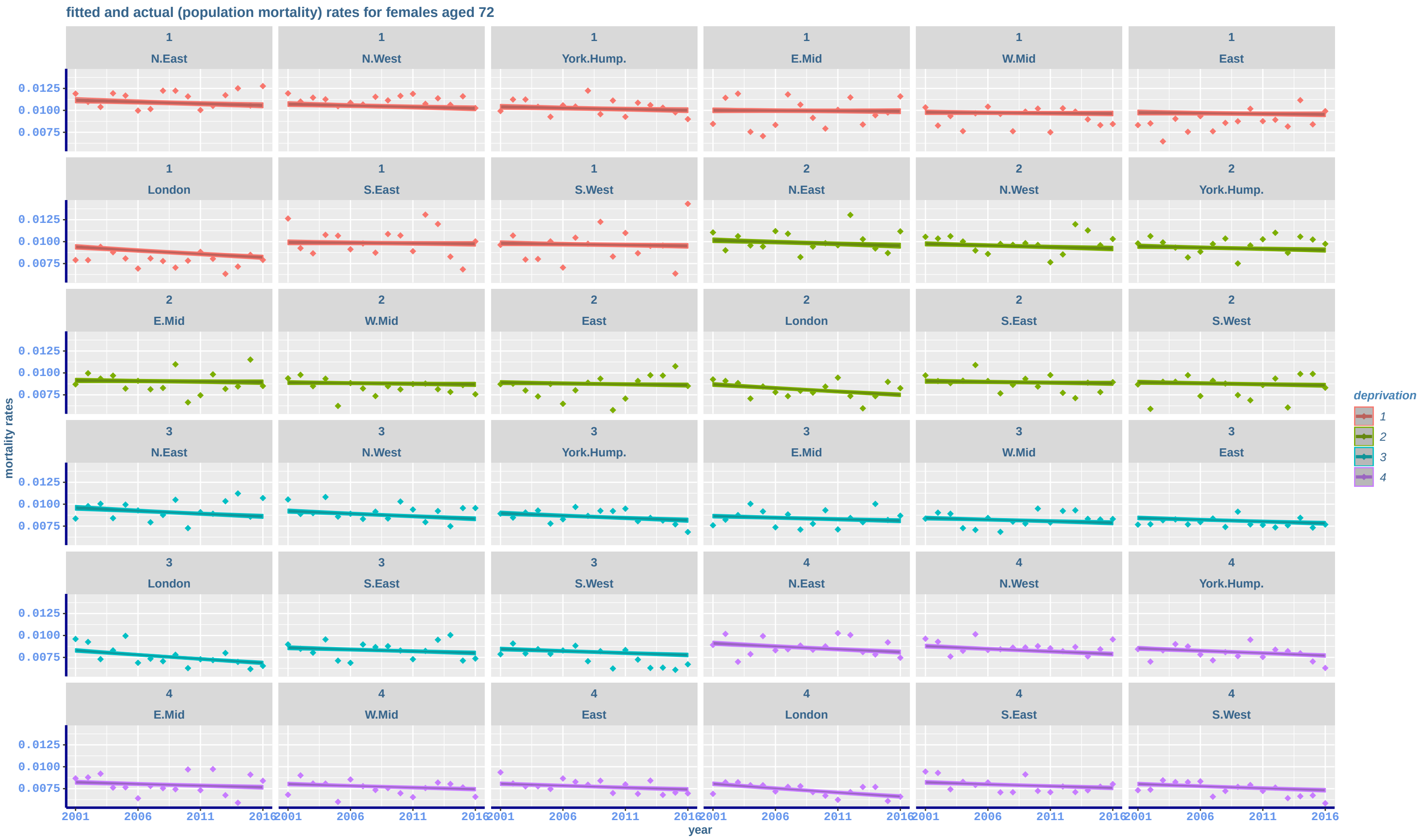

S26 Fig. All-cancer mortality for females, age 72, deprivation deciles 1-4 for all regions in England between 2001 and 2016: observed rates (dots), fitted rates (lines), with 95% credible intervals for the fitted rates.

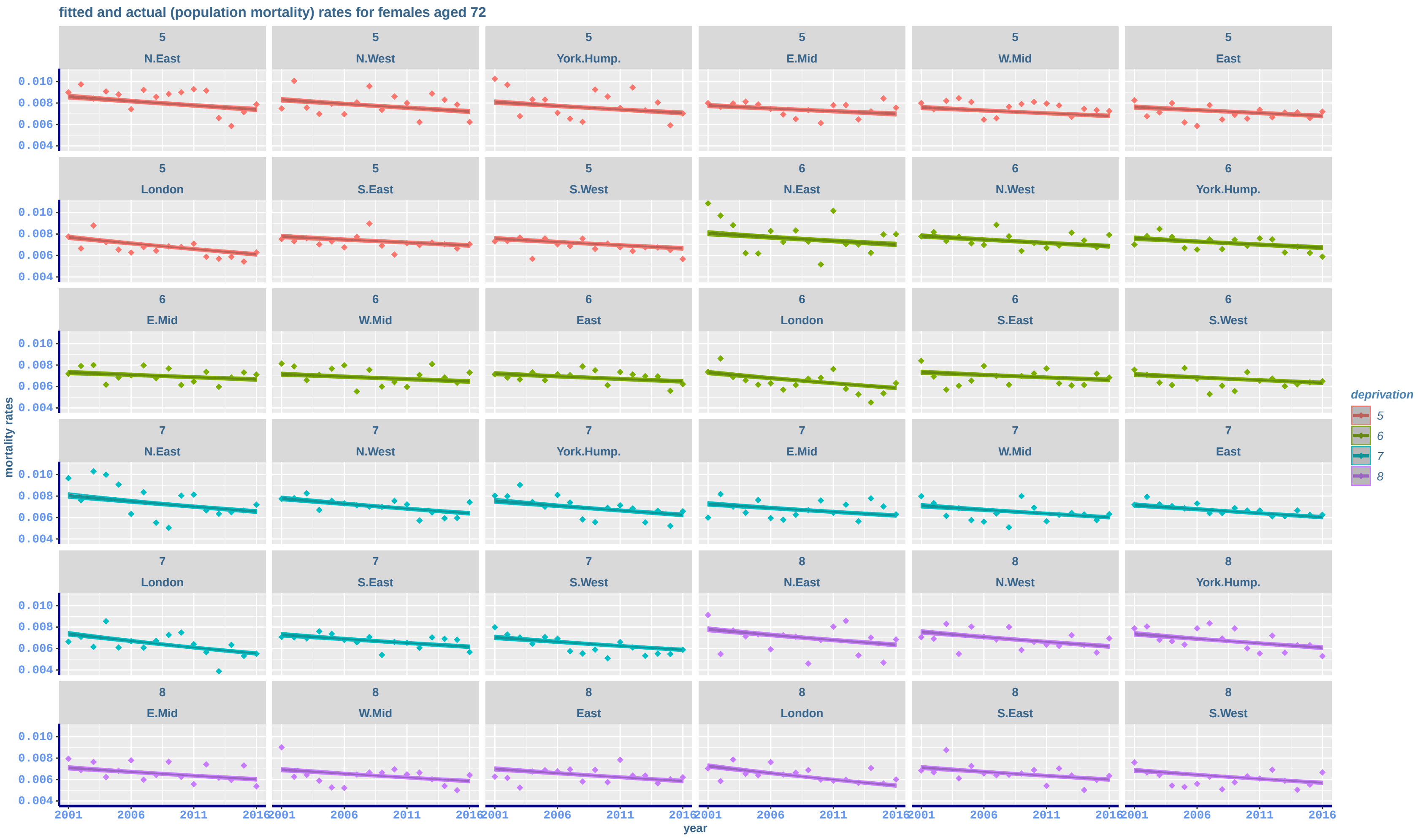

S27 Fig. All-cancer mortality for females, age 72, deprivation deciles 5-8 for all regions in England between 2001 and 2016: observed rates (dots), fitted rates (lines), with 95% credible intervals for the fitted rates.

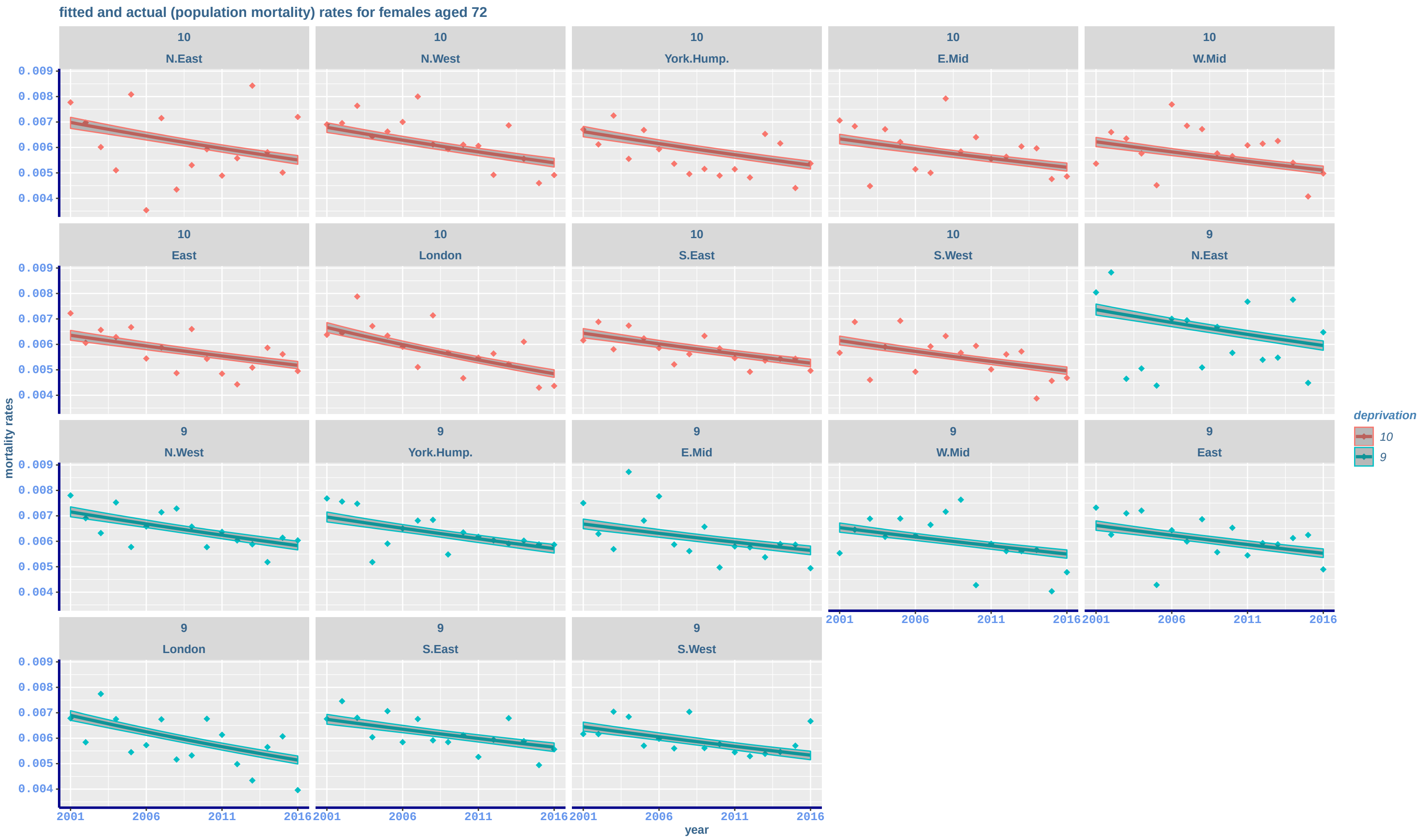

S28 Fig. All-cancer mortality for females, age 72, deprivation deciles 9-10 for all regions in England between 2001 and 2016: observed rates (dots), fitted rates (lines), with 95% credible intervals for the fitted rates.

S9 Table. Absolute deprivation differences ($AD_{t,r}$), per 100,000 people, in age-standardised fitted mortality rates of all-cancer from 2001 to 2016 for all regions in England for males; 95% credible intervals in brackets

year	N.East	N.West	York.Humb.	E.Mid	W.Mid	East	London	S.East	S.West
2001	262 (249, 275)	247 (236, 259)	215 (202, 228)	199 (186, 212)	189 (179, 201)	178 (163, 192)	180 (168, 193)	202 (187, 216)	194 (181, 206)
2002	263 (251, 275)	249 (238, 260)	217 (204, 229)	201 (189, 214)	192 (182, 203)	181 (166, 194)	181 (170, 193)	204 (190, 218)	196 (184, 208)
2003	264 (252, 276)	250 (240, 260)	219 (207, 230)	204 (192, 216)	194 (185, 204)	183 (169, 196)	183 (171, 194)	207 (193, 220)	199 (187, 210)
2004	266 (254, 276)	251 (242, 261)	221 (210, 232)	206 (195, 218)	196 (187, 206)	186 (172, 197)	184 (173, 194)	209 (196, 221)	201 (190, 212)
2005	267 (256, 277)	253 (244, 262)	223 (212, 233)	209 (197, 219)	198 (190, 208)	188 (175, 199)	185 (175, 195)	211 (199, 223)	204 (192, 214)
2006	268 (257, 278)	254 (246, 263)	226 (214, 235)	211 (200, 222)	201 (193, 210)	190 (178, 202)	186 (176, 195)	214 (202, 225)	206 (195, 216)
2007	269 (259, 279)	255 (248, 264)	228 (217, 237)	214 (203, 224)	203 (195, 212)	193 (180, 204)	187 (178, 196)	216 (204, 227)	209 (198, 219)
2008	270 (260, 280)	257 (250, 265)	230 (219, 239)	216 (206, 227)	205 (197, 214)	195 (183, 206)	188 (179, 196)	218 (207, 229)	211 (200, 221)
2009	272 (261, 282)	258 (251, 266)	232 (221, 242)	219 (208, 229)	207 (199, 216)	198 (186, 209)	189 (180, 197)	220 (210, 232)	214 (203, 224)
2010	273 (262, 283)	260 (252, 268)	234 (223, 244)	222 (210, 232)	209 (201, 218)	200 (188, 212)	190 (181, 198)	223 (213, 234)	217 (205, 227)
2011	274 (263, 285)	261 (254, 269)	236 (225, 247)	224 (213, 235)	212 (203, 220)	202 (191, 214)	191 (181, 199)	225 (215, 236)	219 (207, 230)
2012	276 (264, 287)	263 (255, 271)	238 (227, 249)	227 (215, 238)	214 (204, 223)	205 (193, 217)	191 (182, 200)	228 (218, 239)	222 (210, 233)
2013	277 (265, 289)	264 (256, 273)	241 (229, 252)	230 (217, 241)	216 (206, 225)	208 (196, 220)	192 (183, 201)	230 (220, 242)	225 (212, 236)
2014	279 (267, 291)	266 (257, 275)	243 (231, 255)	233 (219, 244)	219 (208, 228)	210 (198, 223)	194 (183, 203)	233 (222, 245)	228 (214, 239)
2015	281 (268, 294)	268 (258, 278)	245 (233, 258)	236 (222, 248)	221 (210, 231)	213 (200, 226)	195 (184, 204)	236 (224, 248)	231 (216, 243)
2016	283 (269, 296)	270 (259, 281)	248 (235, 261)	239 (224, 252)	224 (212, 234)	216 (203, 229)	196 (184, 205)	238 (227, 252)	234 (219, 247)

S10 Table. Relative deprivation differences ($\mathrm{RD}_{t,r}$) in age-standardised fitted mortality rates of all-cancer from 2001 to 2016 in for all regions in England for males; 95% credible intervals in brackets

year	N.East	N.West	York.Humb.	E.Mid	W.Mid	East	London	S.East	S.West
2001	0.5274 (0.4974, 0.5565)	0.5136 (0.4874, 0.5414)	0.4589 (0.4269, 0.4893)	0.4496 (0.4173, 0.4828)	0.4211 (0.3943, 0.4519)	0.4057 (0.3694, 0.44)	0.3951 (0.3663, 0.4253)	0.4477 (0.4142, 0.4809)	0.4528 (0.4205, 0.4838)
2002	0.5415 (0.5128, 0.5695)	0.5275 (0.5026, 0.554)	0.4723 (0.4416, 0.5015)	0.463 (0.4317, 0.4954)	0.4342 (0.4088, 0.4632)	0.4187 (0.3837, 0.4515)	0.408 (0.3805, 0.437)	0.461 (0.4289, 0.493)	0.4661 (0.4346, 0.4961)
2003	0.5555 (0.5279, 0.5827)	0.5414 (0.518, 0.5666)	0.4857 (0.4563, 0.5134)	0.4763 (0.4457, 0.5073)	0.4472 (0.4232, 0.4749)	0.4315 (0.3973, 0.463)	0.4207 (0.394, 0.4484)	0.4743 (0.4436, 0.5055)	0.4795 (0.4486, 0.5081)
2004	0.5694 (0.5427, 0.5953)	0.5552 (0.533, 0.5787)	0.499 (0.4703, 0.525)	0.4895 (0.4596, 0.5198)	0.4602 (0.4377, 0.4866)	0.4444 (0.411, 0.4746)	0.4335 (0.4076, 0.4597)	0.4875 (0.4582, 0.5178)	0.4927 (0.4624, 0.5205)
2005	0.5833 (0.5572, 0.6082)	0.5689 (0.5478, 0.5913)	0.5122 (0.4842, 0.5372)	0.5026 (0.473, 0.5318)	0.4731 (0.4518, 0.4987)	0.4571 (0.4246, 0.4867)	0.4461 (0.4208, 0.471)	0.5006 (0.4721, 0.5306)	0.5059 (0.4763, 0.5329)
2006	0.597 (0.5715, 0.6217)	0.5826 (0.5626, 0.6043)	0.5254 (0.4979, 0.5493)	0.5157 (0.4864, 0.5438)	0.4858 (0.465, 0.5106)	0.4697 (0.4378, 0.499)	0.4587 (0.4338, 0.4824)	0.5136 (0.4863, 0.5433)	0.5189 (0.4892, 0.5455)
2007	0.6107 (0.5846, 0.6357)	0.5961 (0.5766, 0.6174)	0.5384 (0.5112, 0.5619)	0.5286 (0.4997, 0.5563)	0.4985 (0.4781, 0.5224)	0.4823 (0.4507, 0.5115)	0.4711 (0.4462, 0.494)	0.5265 (0.4994, 0.5562)	0.5319 (0.5023, 0.5584)
2008	0.6242 (0.5972, 0.6495)	0.6095 (0.5902, 0.6309)	0.5513 (0.5239, 0.5755)	0.5415 (0.5125, 0.5692)	0.5111 (0.4907, 0.5347)	0.4947 (0.4636, 0.5243)	0.4835 (0.4585, 0.5059)	0.5393 (0.513, 0.5692)	0.5448 (0.515, 0.5716)
2009	0.6376 (0.6098, 0.6638)	0.6228 (0.6031, 0.6447)	0.5641 (0.5364, 0.5895)	0.5542 (0.5246, 0.5823)	0.5236 (0.5024, 0.5473)	0.5071 (0.4761, 0.5375)	0.4957 (0.4704, 0.5184)	0.5521 (0.5263, 0.5817)	0.5575 (0.5275, 0.585)
2010	0.6509 (0.6219, 0.6784)	0.6359 (0.615, 0.6593)	0.5768 (0.5489, 0.6038)	0.5668 (0.5361, 0.5954)	0.5359 (0.5134, 0.5595)	0.5193 (0.488, 0.5507)	0.5078 (0.4819, 0.5308)	0.5646 (0.5392, 0.595)	0.5702 (0.5394, 0.5984)
2011	0.664 (0.6328, 0.6931)	0.6489 (0.6271, 0.6731)	0.5893 (0.5604, 0.6181)	0.5792 (0.5469, 0.6089)	0.5481 (0.5236, 0.5724)	0.5314 (0.4988, 0.5637)	0.5198 (0.4926, 0.5434)	0.5771 (0.5512, 0.609)	0.5826 (0.5512, 0.6122)
2012	0.677 (0.6439, 0.7074)	0.6618 (0.6391, 0.6876)	0.6017 (0.5716, 0.6323)	0.5915 (0.5573, 0.6224)	0.5602 (0.5337, 0.5851)	0.5433 (0.5098, 0.5771)	0.5317 (0.5032, 0.5568)	0.5893 (0.562, 0.6225)	0.595 (0.5619, 0.6258)
2013	0.6898 (0.6547, 0.7223)	0.6744 (0.6497, 0.7015)	0.6139 (0.5825, 0.6467)	0.6037 (0.5676, 0.6361)	0.5721 (0.5433, 0.598)	0.555 (0.5201, 0.5905)	0.5433 (0.5131, 0.5699)	0.6015 (0.5723, 0.6354)	0.6071 (0.572, 0.6399)
2014	0.7024 (0.6646, 0.7369)	0.6869 (0.6591, 0.7154)	0.6259 (0.5926, 0.6607)	0.6156 (0.5777, 0.6498)	0.5838 (0.5527, 0.6113)	0.5666 (0.5308, 0.6041)	0.5548 (0.5225, 0.5832)	0.6134 (0.5822, 0.6489)	0.6191 (0.5817, 0.6538)
2015	0.7147 (0.6743, 0.7515)	0.6992 (0.668, 0.7294)	0.6377 (0.6024, 0.6747)	0.6274 (0.5878, 0.6638)	0.5953 (0.562, 0.6249)	0.578 (0.5409, 0.6175)	0.5661 (0.5315, 0.5964)	0.6251 (0.592, 0.6625)	0.6309 (0.5915, 0.6672)
2016	0.7269 (0.6834, 0.7654)	0.7112 (0.6777, 0.744)	0.6494 (0.6118, 0.6885)	0.6389 (0.5973, 0.6775)	0.6066 (0.5707, 0.6388)	0.5892 (0.551, 0.631)	0.5772 (0.5398, 0.6099)	0.6367 (0.6013, 0.6762)	0.6424 (0.6008, 0.681)

S11 Table. Absolute deprivation differences ($\mathbf{AD}_{t,r}$), per 100,000 people, in age-standardised fitted mortality rates of all-cancer from 2001 to 2016 for all regions in England for females; 95% credible intervals in brackets

year	N.East	N.West	York.Humb.	E.Mid	W.Mid	East	London	S.East	S.West
2001	131<br>(123, 139)	121<br>(114, 129)	116<br>(109, 124)	114<br>(107, 121)	109<br>(103, 116)	102<br>(95, 110)	73<br>(66, 80)	105<br>(98, 112)	115<br>(108, 122)
2002	134<br>(127, 141)	125<br>(118, 132)	120<br>(113, 127)	117<br>(111, 124)	113<br>(107, 119)	105<br>(99, 112)	75<br>(69, 82)	109<br>(102, 115)	118<br>(112, 125)
2003	137<br>(131, 144)	128<br>(122, 134)	123<br>(117, 129)	120<br>(114, 126)	116<br>(110, 122)	109<br>(103, 115)	78<br>(73, 84)	112<br>(106, 118)	121<br>(115, 127)
2004	141<br>(135, 146)	131<br>(126, 137)	126<br>(121, 132)	124<br>(118, 129)	119<br>(114, 124)	112<br>(107, 117)	81<br>(76, 86)	115<br>(110, 121)	124<br>(119, 130)
2005	144<br>(139, 149)	134<br>(130, 140)	129<br>(125, 134)	127<br>(122, 132)	122<br>(118, 127)	115<br>(110, 120)	84<br>(79, 88)	119<br>(114, 123)	127<br>(123, 132)
2006	147<br>(142, 152)	138<br>(133, 142)	133<br>(128, 137)	130<br>(126, 135)	126<br>(122, 130)	118<br>(114, 122)	86<br>(82, 90)	122<br>(118, 126)	130<br>(126, 135)
2007	150<br>(146, 154)	141<br>(137, 145)	136<br>(132, 140)	134<br>(130, 138)	129<br>(125, 132)	121<br>(118, 125)	89<br>(84, 93)	125<br>(121, 129)	133<br>(129, 137)
2008	153<br>(149, 157)	144<br>(140, 148)	139<br>(135, 143)	137<br>(133, 141)	132<br>(128, 135)	125<br>(121, 128)	91<br>(87, 95)	128<br>(125, 132)	136<br>(132, 140)
2009	156<br>(152, 160)	147<br>(143, 151)	142<br>(138, 146)	140<br>(136, 144)	135<br>(131, 139)	128<br>(124, 132)	93<br>(89, 97)	131<br>(128, 135)	139<br>(135, 143)
2010	159<br>(155, 164)	150<br>(146, 154)	145<br>(141, 149)	143<br>(139, 148)	138<br>(134, 142)	131<br>(127, 135)	96<br>(92, 100)	135<br>(131, 139)	142<br>(138, 146)
2011	162<br>(158, 167)	153<br>(148, 157)	148<br>(144, 152)	147<br>(142, 151)	141<br>(137, 145)	134<br>(130, 138)	98<br>(94, 102)	138<br>(134, 142)	145<br>(141, 149)
2012	165<br>(160, 170)	156<br>(151, 161)	151<br>(146, 156)	150<br>(145, 155)	144<br>(140, 149)	137<br>(132, 142)	100<br>(96, 105)	141<br>(136, 146)	148<br>(143, 153)
2013	168<br>(163, 174)	159<br>(154, 165)	154<br>(149, 160)	153<br>(147, 159)	148<br>(143, 153)	140<br>(135, 146)	102<br>(98, 107)	144<br>(139, 150)	151<br>(146, 156)
2014	171<br>(165, 178)	162<br>(156, 168)	157<br>(151, 163)	156<br>(150, 163)	151<br>(145, 156)	143<br>(137, 149)	104<br>(99, 110)	147<br>(141, 153)	154<br>(148, 160)
2015	174<br>(167, 181)	165<br>(158, 172)	160<br>(154, 167)	159<br>(152, 167)	154<br>(148, 160)	146<br>(140, 153)	106<br>(101, 113)	150<br>(144, 157)	156<br>(150, 163)
2016	177<br>(170, 185)	168<br>(161, 176)	163<br>(156, 170)	163<br>(155, 171)	157<br>(150, 164)	149<br>(142, 157)	108<br>(102, 115)	154<br>(146, 161)	159<br>(153, 167)

S12 Table. Relative deprivation differences ($\mathrm{RD}_{t,r}$) in age-standardised fitted mortality rates of all-cancer from 2001 to 2016 for all regions in England for females; 95% credible intervals in brackets

year	N.East	N.West	York.Humb.	E.Mid	W.Mid	East	London	S.East	S.West
2001	0.3897	0.3723	0.3659	0.3732	0.3658	0.3342	0.2266	0.3404	0.3893
	(0.3628, 0.4178)	(0.346, 0.3999)	(0.3398, 0.3933)	(0.3469, 0.4008)	(0.3397, 0.3933)	(0.3086, 0.3611)	(0.2028, 0.2523)	(0.3148, 0.3674)	(0.3625, 0.4175)
2002	0.4045	0.387	0.3805	0.3879	0.3805	0.3485	0.2398	0.3548	0.4042
	(0.3798, 0.4303)	(0.3627, 0.4122)	(0.3565, 0.4056)	(0.3636, 0.4132)	(0.3564, 0.4055)	(0.3251, 0.373)	(0.218, 0.2632)	(0.3313, 0.3793)	(0.3794, 0.4298)
2003	0.4196	0.4018	0.3953	0.4028	0.3952	0.3629	0.253	0.3693	0.4192
	(0.397, 0.443)	(0.3799, 0.4248)	(0.3735, 0.418)	(0.3808, 0.4257)	(0.3735, 0.418)	(0.3417, 0.3852)	(0.2331, 0.2741)	(0.3481, 0.3918)	(0.3966, 0.4426)
2004	0.4348	0.4168	0.4102	0.4178	0.4102	0.3775	0.2665	0.3839	0.4344
	(0.4142, 0.4562)	(0.3968, 0.4379)	(0.3904, 0.4311)	(0.3976, 0.4389)	(0.3903, 0.4311)	(0.3584, 0.3979)	(0.2482, 0.2857)	(0.3646, 0.4044)	(0.4137, 0.4558)
2005	0.4501	0.432	0.4253	0.4329	0.4252	0.3922	0.28	0.3987	0.4497
	(0.4317, 0.4695)	(0.4139, 0.451)	(0.4074, 0.4442)	(0.4149, 0.452)	(0.4073, 0.4442)	(0.3748, 0.4107)	(0.2627, 0.2976)	(0.3813, 0.4174)	(0.4313, 0.469)
2006	0.4656	0.4473	0.4405	0.4482	0.4405	0.4071	0.2937	0.4137	0.4652
	(0.4488, 0.4831)	(0.4309, 0.4647)	(0.4243, 0.4578)	(0.4319, 0.4656)	(0.4243, 0.4577)	(0.3913, 0.4239)	(0.2772, 0.31)	(0.3978, 0.4306)	(0.4484, 0.4828)
2007	0.4813	0.4627	0.4559	0.4637	0.4558	0.4221	0.3075	0.4288	0.4808
	(0.4654, 0.4979)	(0.4473, 0.4787)	(0.4406, 0.4717)	(0.4483, 0.4797)	(0.4405, 0.4715)	(0.4072, 0.4374)	(0.2917, 0.323)	(0.4139, 0.4441)	(0.465, 0.4975)
2008	0.4971	0.4784	0.4715	0.4793	0.4714	0.4373	0.3215	0.444	0.4967
	(0.4815, 0.513)	(0.4634, 0.4938)	(0.4566, 0.4867)	(0.4643, 0.4948)	(0.4565, 0.4866)	(0.4231, 0.452)	(0.3057, 0.3366)	(0.4297, 0.4587)	(0.4812, 0.5125)
2009	0.5131	0.4941	0.4872	0.4951	0.4871	0.4526	0.3356	0.4594	0.5126
	(0.4972, 0.5288)	(0.4788, 0.5096)	(0.4721, 0.5026)	(0.4797, 0.5106)	(0.472, 0.5026)	(0.4379, 0.4676)	(0.3195, 0.3507)	(0.4447, 0.4746)	(0.4968, 0.5284)
2010	0.5292	0.5101	0.5031	0.5111	0.503	0.4681	0.3498	0.475	0.5288
	(0.5125, 0.5456)	(0.4938, 0.5264)	(0.4871, 0.5191)	(0.4948, 0.5273)	(0.4869, 0.5191)	(0.4525, 0.4838)	(0.3334, 0.366)	(0.4593, 0.4907)	(0.5122, 0.5452)
2011	0.5455	0.5262	0.5191	0.5272	0.519	0.4838	0.3642	0.4907	0.5451
	(0.5269, 0.5636)	(0.5081, 0.5439)	(0.5012, 0.5366)	(0.5091, 0.5449)	(0.5011, 0.5366)	(0.4666, 0.5012)	(0.3466, 0.3819)	(0.4735, 0.5081)	(0.5265, 0.5632)
2012	0.562	0.5425	0.5353	0.5435	0.5352	0.4996	0.3788	0.5066	0.5616
	(0.5412, 0.5824)	(0.5223, 0.5622)	(0.5153, 0.5549)	(0.5234, 0.5634)	(0.5152, 0.5548)	(0.4804, 0.5189)	(0.3596, 0.398)	(0.4873, 0.5259)	(0.5408, 0.5819)
2013	0.5787	0.5589	0.5517	0.56	0.5516	0.5156	0.3935	0.5227	0.5782
	(0.5551, 0.6019)	(0.5363, 0.5817)	(0.5293, 0.5742)	(0.5372, 0.5828)	(0.5292, 0.5742)	(0.494, 0.5374)	(0.3725, 0.4152)	(0.5011, 0.5447)	(0.5547, 0.6014)
2014	0.5955	0.5755	0.5682	0.5766	0.5681	0.5318	0.4083	0.5389	0.595
	(0.5694, 0.6216)	(0.5504, 0.6012)	(0.5433, 0.5938)	(0.5515, 0.6022)	(0.5431, 0.5937)	(0.5075, 0.5564)	(0.3849, 0.4326)	(0.5146, 0.5639)	(0.5691, 0.6212)
2015	0.6125	0.5923	0.5849	0.5934	0.5848	0.5481	0.4233	0.5553	0.612
	(0.5839, 0.6418)	(0.5645, 0.6211)	(0.5573, 0.6135)	(0.5654, 0.622)	(0.5572, 0.6135)	(0.521, 0.5763)	(0.3971, 0.4505)	(0.5281, 0.5836)	(0.5835, 0.6414)
2016	0.6297	0.6093	0.6018	0.6104	0.6017	0.5646	0.4385	0.5719	0.6292
	(0.5979, 0.6628)	(0.5783, 0.642)	(0.5709, 0.6343)	(0.5794, 0.643)	(0.5709, 0.6343)	(0.5344, 0.5965)	(0.4095, 0.4691)	(0.5414, 0.604)	(0.5975, 0.6623)

S13 Table. Estimated coefficients for the best fitting model for lung cancer morbidity.

Covariate	Parameter	Mean	SD	%2.5	%97.5	Covariate	Parameter	Mean	SD	%2.5	%97.5
Intercept	β_0	-6.3740	0.0026	-6.3780	-6.3680	Age:Deprivation	$\beta_{7,\mathrm{age}_9,\mathrm{deprivation}_2}$	-0.2019	0.0105	-0.2197	-0.1796
Age	β_{1,age_1}	-2.4090	0.0155	-2.4370	-2.3810		$\beta_{7,\mathrm{age}_1,\mathrm{deprivation}_3}$	0.0912	0.0308	0.0243	0.1372
	β_{1,age_2}	-1.5440	0.0062	-1.5570	-1.5330		$\beta_{7,\mathrm{age}_2,\mathrm{deprivation}_3}$	0.0425	0.0138	0.0123	0.0625
	β_{1,age_3}	-0.7645	0.0074	-0.7778	-0.7508		$\beta_{7,\mathrm{age}_3,\mathrm{deprivation}_3}$	0.0466	0.0129	0.0218	0.0749
	β_{1,age_4}	-0.1314	0.0058	-0.1443	-0.1211		$\beta_{7,\mathrm{age}_4,\mathrm{deprivation}_3}$	0.0183	0.0104	0.0003	0.0398
	β_{1,age_5}	0.3410	0.0051	0.3318	0.3501		$\beta_{7,\mathrm{age}_5,\mathrm{deprivation}_3}$	0.0361	0.0115	0.0135	0.0627
	β_{1,age_6}	0.7337	0.0042	0.7263	0.7418		$\beta_{7,\mathrm{age}_6,\mathrm{deprivation}_3}$	0.0205	0.0093	0.0002	0.0346
	β_{1,age_7}	1.0290	0.0054	1.0190	1.0400		$\beta_{7,\mathrm{age}_7,\mathrm{deprivation}_3}$	-0.0417	0.0084	-0.0563	-0.0240
	β_{1,age_8}	1.1790	0.0057	1.1690	1.1900		$\beta_{7,\mathrm{age}_8,\mathrm{deprivation}_3}$	-0.0861	0.0123	-0.1097	-0.0619
	β_{1,age_9}	1.5650	0.0058	1.5540	1.5750		$\beta_{7,\mathrm{age}_9,\mathrm{deprivation}_3}$	-0.1275	0.0111	-0.1443	-0.1066
Year	β_2	-0.0697	0.0026	-0.0759	-0.0659		$\beta_{7,\mathrm{age}_1,\mathrm{deprivation}_4}$	0.0316	0.0288	-0.0303	0.0773
Gender	β_3	-0.3962	0.0033	-0.4039	-0.3907		$\beta_{7,\mathrm{age}_2,\mathrm{deprivation}_4}$	0.0636	0.0196	0.0259	0.1037
Region	$\beta_{4,\mathrm{region}_1}$	0.1104	0.0086	0.0933	0.1262		$\beta_{7,\mathrm{age}_3,\mathrm{deprivation}_4}$	0.0196	0.0133	-0.0032	0.0429
	$\beta_{4,\mathrm{region}_2}$	0.0831	0.0044	0.0756	0.0924		$\beta_{7,\mathrm{age}_4,\mathrm{deprivation}_4}$	0.0406	0.0091	0.0215	0.0567
	$\beta_{4,\mathrm{region}_3}$	0.0829	0.0057	0.0711	0.0937		$\beta_{7,\mathrm{age}_5,\mathrm{deprivation}_4}$	0.0138	0.0103	-0.0076	0.0344
	$\beta_{4,\mathrm{region}_4}$	-0.0081	0.0064	-0.0170	0.0054		$\beta_{7,\mathrm{age}_6,\mathrm{deprivation}_4}$	0.0050	0.0076	-0.0096	0.0198
	$\beta_{4,\mathrm{region}_5}$	-0.0627	0.0047	-0.0726	-0.0542		$\beta_{7,\mathrm{age}_7,\mathrm{deprivation}_4}$	-0.0161	0.0072	-0.0289	-0.0016
	$\beta_{4,\mathrm{region}_6}$	-0.0711	0.0073	-0.0827	-0.0579		$\beta_{7,\mathrm{age}_8,\mathrm{deprivation}_4}$	-0.0533	0.0108	-0.0756	-0.0344
	$\beta_{4,\mathrm{region}_7}$	-0.0423	0.0071	-0.0576	-0.0308		$\beta_{7,\mathrm{age}_9,\mathrm{deprivation}_4}$	-0.1048	0.0100	-0.1253	-0.0875
	$\beta_{4,\mathrm{region}_8}$	-0.0247	0.0045	-0.0334	-0.0143		$\beta_{7,\mathrm{age}_1,\mathrm{deprivation}_5}$	-0.0346	0.0288	-0.0974	0.0077
	$\beta_{4,\mathrm{region}_9}$	-0.0676	0.0059	-0.0787	-0.0565		$\beta_{7,\mathrm{age}_2,\mathrm{deprivation}_5}$	0.0538	0.0213	0.0221	0.1129
Deprivation	$\beta_{5,\mathrm{deprivation}_1}$	0.5881	0.0063	0.5766	0.6012		$\beta_{7,\mathrm{age}_3,\mathrm{deprivation}_5}$	0.0149	0.0144	-0.0094	0.0467
	$\beta_{5,\mathrm{deprivation}_2}$	0.4276	0.0041	0.4205	0.4364		$\beta_{7,\mathrm{age}_4,\mathrm{deprivation}_5}$	-0.0001	0.0134	-0.0268	0.0246
	$\beta_{5,\mathrm{deprivation}_3}$	0.2798	0.0054	0.2672	0.2881		$\beta_{7,\mathrm{age}_5,\mathrm{deprivation}_5}$	0.0077	0.0110	-0.0136	0.0347
	$\beta_{5,\mathrm{deprivation}_4}$	0.1652	0.0041	0.1567	0.1724		$\beta_{7,\mathrm{age}_6,\mathrm{deprivation}_5}$	-0.0014	0.0086	-0.0182	0.0155
	$\beta_{5,\mathrm{deprivation}_5}$	0.0327	0.0048	0.0218	0.0416		$\beta_{7,\mathrm{age}_7,\mathrm{deprivation}_5}$	-0.0110	0.0113	-0.0322	0.0120
	$\beta_{5,\mathrm{deprivation}_6}$	-0.0847	0.0056	-0.0945	-0.0749		$\beta_{7,\mathrm{age}_8,\mathrm{deprivation}_5}$	0.0013	0.0111	-0.0192	0.0258
	$\beta_{5,\mathrm{deprivation}_7}$	-0.1898	0.0058	-0.1993	-0.1745		$\beta_{7,\mathrm{age}_9,\mathrm{deprivation}_5}$	-0.0306	0.0110	-0.0535	-0.0097
	$\beta_{5,\mathrm{deprivation}_8}$	-0.2851	0.0044	-0.2972	-0.2787		$\beta_{7,\mathrm{age}_1,\mathrm{deprivation}_6}$	-0.0183	0.0311	-0.0625	0.0520
	$\beta_{5,\mathrm{deprivation}_9}$	-0.3861	0.0053	-0.3965	-0.3758		$\beta_{7,\mathrm{age}_2,\mathrm{deprivation}_6}$	-0.0320	0.0226	-0.0789	0.0107
	$\beta_{5,\mathrm{deprivation}_{10}}$	-0.5477	0.0047	-0.5561	-0.5375		$\beta_{7,\mathrm{age}_3,\mathrm{deprivation}_6}$	0.0044	0.0194	-0.0309	0.0354
Age:Gender	$\beta_{6,\mathrm{gender}_F,\mathrm{age}_1}$	0.3495	0.0224	0.3134	0.3926		$\beta_{7,\mathrm{age}_4,\mathrm{deprivation}_6}$	-0.0153	0.0123	-0.0371	0.0107
	$\beta_{6,\mathrm{gender}_F,\mathrm{age}_2}$	0.2743	0.0073	0.2611	0.2878		$\beta_{7,\mathrm{age}_5,\mathrm{deprivation}_6}$	-0.0262	0.0114	-0.0457	-0.0040
	$\beta_{6,\mathrm{gender}_F,\mathrm{age}_3}$	0.1565	0.0088	0.1401	0.1749		$\beta_{7,\mathrm{age}_6,\mathrm{deprivation}_6}$	0.0040	0.0115	-0.0154	0.0272
	$\beta_{6,\mathrm{gender}_F,\mathrm{age}_4}$	0.0571	0.0078	0.0447	0.0748		$\beta_{7,\mathrm{age}_7,\mathrm{deprivation}_6}$	0.0167	0.0101	-0.0060	0.0361
	$\beta_{6,\mathrm{gender}_F,\mathrm{age}_5}$	-0.0104	0.0058	-0.0206	0.0005		$\beta_{7,\mathrm{age}_8,\mathrm{deprivation}_6}$	0.0242	0.0114	0.0034	0.0487
	$\beta_{6,\mathrm{gender}_F,\mathrm{age}_6}$	-0.0798	0.0057	-0.0896	-0.0689		$\beta_{7,\mathrm{age}_9,\mathrm{deprivation}_6}$	0.0425	0.0097	0.0247	0.0625
	$\beta_{6,\mathrm{gender}_F,\mathrm{age}_7}$	-0.1651	0.0067	-0.1782	-0.1529		$\beta_{7,\mathrm{age}_1,\mathrm{deprivation}_7}$	-0.0215	0.0180	-0.0593	0.0096
	$\beta_{6,\mathrm{gender}_F,\mathrm{age}_8}$	-0.2438	0.0077	-0.2585	-0.2283		$\beta_{7,\mathrm{age}_2,\mathrm{deprivation}_7}$	-0.0619	0.0179	-0.0997	-0.0305
	$\beta_{6,\mathrm{gender}_F,\mathrm{age}_9}$	-0.3384	0.0102	-0.3533	-0.3166		$\beta_{7,\mathrm{age}_3,\mathrm{deprivation}_7}$	-0.0614	0.0212	-0.1008	-0.0194
Age:Deprivation	$\beta_{7,\mathrm{age}_1,\mathrm{deprivation}_1}$	0.1506	0.0275	0.0959	0.1993		$\beta_{7,\mathrm{age}_4,\mathrm{deprivation}_7}$	-0.0472	0.0119	-0.0733	-0.0279
	$\beta_{7,\mathrm{age}_2,\mathrm{deprivation}_1}$	0.1276	0.0133	0.1024	0.1522		$\beta_{7,\mathrm{age}_5,\mathrm{deprivation}_7}$	-0.0015	0.0116	-0.0303	0.0160
	$\beta_{7,\mathrm{age}_3,\mathrm{deprivation}_1}$	0.1475	0.0135	0.1218	0.1759		$\beta_{7,\mathrm{age}_6,\mathrm{deprivation}_7}$	0.0077	0.0095	-0.0130	0.0258
	$\beta_{7,\mathrm{age}_4,\mathrm{deprivation}_1}$	0.1184	0.0089	0.1016	0.1353		$\beta_{7,\mathrm{age}_7,\mathrm{deprivation}_7}$	0.0237	0.0087	0.0090	0.0418
	$\beta_{7,\mathrm{age}_5,\mathrm{deprivation}_1}$	0.0503	0.0098	0.0319	0.0704		$\beta_{7,\mathrm{age}_8,\mathrm{deprivation}_7}$	0.0698	0.0099	0.0503	0.0890
	$\beta_{7,\mathrm{age}_6,\mathrm{deprivation}_1}$	0.0013	0.0089	-0.0153	0.0191		$\beta_{7,\mathrm{age}_9,\mathrm{deprivation}_7}$	0.0925	0.0096	0.0747	0.1116
	$\beta_{7,\mathrm{age}_7,\mathrm{deprivation}_1}$	-0.1111	0.0089	-0.1299	-0.0948		$\beta_{7,\mathrm{age}_1,\mathrm{deprivation}_8}$	-0.0268	0.0222	-0.0659	0.0154
	$\beta_{7,\mathrm{age}_8,\mathrm{deprivation}_1}$	-0.2011	0.0122	-0.2228	-0.1749		$\beta_{7,\mathrm{age}_2,\mathrm{deprivation}_8}$	-0.0604	0.0217	-0.1009	-0.0198
	$\beta_{7,\mathrm{age}_9,\mathrm{deprivation}_1}$	-0.2835	0.0107	-0.3030	-0.2614		$\beta_{7,\mathrm{age}_3,\mathrm{deprivation}_8}$	-0.0785	0.0107	-0.1007	-0.0556
	$\beta_{7,\mathrm{age}_1,\mathrm{deprivation}_2}$	0.0222	0.0260	-0.0401	0.0625		$\beta_{7,\mathrm{age}_4,\mathrm{deprivation}_8}$	-0.0612	0.0145	-0.0871	-0.0289
	$\beta_{7,\mathrm{age}_2,\mathrm{deprivation}_2}$	0.1310	0.0157	0.1026	0.1676		$\beta_{7,\mathrm{age}_5,\mathrm{deprivation}_8}$	-0.0408	0.0099	-0.0631	-0.0218
	$\beta_{7,\mathrm{age}_3,\mathrm{deprivation}_2}$	0.1147	0.0142	0.0902	0.1426		$\beta_{7,\mathrm{age}_6,\mathrm{deprivation}_8}$	-0.0162	0.0096	-0.0341	0.0014
	$\beta_{7,\mathrm{age}_4,\mathrm{deprivation}_2}$	0.0819	0.0099	0.0594	0.0972		$\beta_{7,\mathrm{age}_7,\mathrm{deprivation}_8}$	0.0565	0.0084	0.0419	0.0747
	$\beta_{7,\mathrm{age}_5,\mathrm{deprivation}_2}$	0.0536	0.0104	0.0336	0.0723		$\beta_{7,\mathrm{age}_8,\mathrm{deprivation}_8}$	0.0949	0.0110	0.0709	0.1140
	$\beta_{7,\mathrm{age}_6,\mathrm{deprivation}_2}$	-0.0205	0.0103	-0.0406	0.0003		$\beta_{7,\mathrm{age}_9,\mathrm{deprivation}_8}$	0.1326	0.0127	0.1042	0.1557
	$\beta_{7,\mathrm{age}_7,\mathrm{deprivation}_2}$	-0.0549	0.0079	-0.0713	-0.0398		$\beta_{7,\mathrm{age}_1,\mathrm{deprivation}_9}$	-0.0894	0.0243	-0.1451	-0.0521
	$\beta_{7,\mathrm{age}_8,\mathrm{deprivation}_2}$	-0.1261	0.0108	-0.1487	-0.1049		$\beta_{7,\mathrm{age}_2,\mathrm{deprivation}_9}$	-0.1175	0.0266	-0.1628	-0.0789

Covariate	Parameter	Mean	SD	%2.5	%97.5	Covariate	Parameter	Mean	SD	%2.5	%97.5
Age:Deprivation	$\beta_{7,\text{age}_3,\text{deprivation}_9}$	-0.1014	0.0116	-0.1235	-0.0762	Deprivation:Region	$\beta_{11,\text{deprivation}_3,\text{region}_5}$	0.0108	0.0101	-0.0080	0.0317
	$\beta_{7,\text{age}_4,\text{deprivation}_9}$	-0.0492	0.0125	-0.0734	-0.0215		$\beta_{11,\text{deprivation}_3,\text{region}_6}$	0.0088	0.0121	-0.0103	0.0346
	$\beta_{7,\text{age}_5,\text{deprivation}_9}$	-0.0305	0.0165	-0.0583	0.0022		$\beta_{11,\text{deprivation}_3,\text{region}_7}$	-0.0618	0.0092	-0.0806	-0.0418
	$\beta_{7,\text{age}_6,\text{deprivation}_9}$	0.0011	0.0140	-0.0269	0.0236		$\beta_{11,\text{deprivation}_3,\text{region}_8}$	-0.0004	0.0088	-0.0152	0.0190
	$\beta_{7,\text{age}_7,\text{deprivation}_9}$	0.0621	0.0089	0.0465	0.0796		$\beta_{11,\text{deprivation}_3,\text{region}_9}$	-0.0256	0.0112	-0.0429	-0.0000
	$\beta_{7,\text{age}_8,\text{deprivation}_9}$	0.1246	0.0106	0.1031	0.1443		$\beta_{11,\text{deprivation}_4,\text{region}_1}$	0.0112	0.0190	-0.0222	0.0520
	$\beta_{7,\text{age}_9,\text{deprivation}_9}$	0.2003	0.0124	0.1727	0.2197		$\beta_{11,\text{deprivation}_4,\text{region}_2}$	-0.0014	0.0090	-0.0198	0.0157
	$\beta_{7,\text{age}_1,\text{deprivation}_{10}}$	-0.1050	0.0315	-0.1479	-0.0401		$\beta_{11,\text{deprivation}_4,\text{region}_3}$	0.0031	0.0119	-0.0260	0.0226
	$\beta_{7,\text{age}_2,\text{deprivation}_{10}}$	-0.1467	0.0203	-0.1872	-0.1090		$\beta_{11,\text{deprivation}_4,\text{region}_4}$	0.0221	0.0115	-0.0023	0.0431
	$\beta_{7,\text{age}_3,\text{deprivation}_{10}}$	-0.1063	0.0250	-0.1435	-0.0583		$\beta_{11,\text{deprivation}_4,\text{region}_5}$	0.0215	0.0153	-0.0054	0.0530
	$\beta_{7,\text{age}_4,\text{deprivation}_{10}}$	-0.0863	0.0124	-0.1096	-0.0571		$\beta_{11,\text{deprivation}_4,\text{region}_6}$	0.0073	0.0120	-0.0146	0.0328
	$\beta_{7,\text{age}_5,\text{deprivation}_{10}}$	-0.0625	0.0146	-0.0877	-0.0351		$\beta_{11,\text{deprivation}_4,\text{region}_7}$	-0.0469	0.0117	-0.0645	-0.0197
	$\beta_{7,\text{age}_6,\text{deprivation}_{10}}$	-0.0015	0.0107	-0.0217	0.0176		$\beta_{11,\text{deprivation}_4,\text{region}_8}$	0.0116	0.0101	-0.0125	0.0281
	$\beta_{7,\text{age}_7,\text{deprivation}_{10}}$	0.0759	0.0130	0.0551	0.0998		$\beta_{11,\text{deprivation}_4,\text{region}_9}$	-0.0287	0.0131	-0.0506	0.0015
	$\beta_{7,\text{age}_8,\text{deprivation}_{10}}$	0.1520	0.0141	0.1250	0.1772		$\beta_{11,\text{deprivation}_5,\text{region}_1}$	-0.0055	0.0158	-0.0341	0.0333
	$\beta_{7,\text{age}_9,\text{deprivation}_{10}}$	0.2805	0.0104	0.2613	0.3035		$\beta_{11,\text{deprivation}_5,\text{region}_2}$	0.0003	0.0101	-0.0210	0.0192
Gender:Year	$\beta_{8,\text{gender}_F}$	0.1360	0.0024	0.1312	0.1405		$\beta_{11,\text{deprivation}_5,\text{region}_3}$	-0.0123	0.0100	-0.0308	0.0095
Age:Year	β_{9,age_1}	-0.0898	0.0107	-0.1112	-0.0714		$\beta_{11,\text{deprivation}_5,\text{region}_4}$	-0.0008	0.0110	-0.0229	0.0199
	β_{9,age_2}	-0.0900	0.0079	-0.1057	-0.0776		$\beta_{11,\text{deprivation}_5,\text{region}_5}$	0.0178	0.0116	-0.0050	0.0411
	β_{9,age_3}	-0.0374	0.0042	-0.0449	-0.0272		$\beta_{11,\text{deprivation}_5,\text{region}_6}$	-0.0248	0.0113	-0.0458	-0.0024
	β_{9,age_4}	-0.0015	0.0035	-0.0080	0.0062		$\beta_{11,\text{deprivation}_5,\text{region}_7}$	0.0017	0.0125	-0.0198	0.0268
	β_{9,age_5}	0.0310	0.0037	0.0243	0.0378		$\beta_{11,\text{deprivation}_5,\text{region}_8}$	0.0220	0.0092	0.0031	0.0383
	β_{9,age_6}	0.0344	0.0039	0.0281	0.0424		$\beta_{11,\text{deprivation}_5,\text{region}_9}$	0.0017	0.0125	-0.0263	0.0220
	β_{9,age_7}	0.0050	0.0041	-0.0030	0.0125		$\beta_{11,\text{deprivation}_6,\text{region}_1}$	-0.0510	0.0203	-0.0863	-0.0110
	β_{9,age_8}	0.0403	0.0035	0.0329	0.0489		$\beta_{11,\text{deprivation}_6,\text{region}_2}$	-0.0016	0.0112	-0.0215	0.0230
	β_{9,age_9}	0.1080	0.0039	0.1002	0.1162		$\beta_{11,\text{deprivation}_6,\text{region}_3}$	0.0236	0.0098	0.0042	0.0417
Gender:Region	$\beta_{10,\text{gender}_F,\text{region}_1}$	0.1320	0.0098	0.1148	0.1535		$\beta_{11,\text{deprivation}_6,\text{region}_4}$	0.0035	0.0138	-0.0196	0.0307
	$\beta_{10,\text{gender}_F,\text{region}_2}$	0.0784	0.0067	0.0653	0.0904		$\beta_{11,\text{deprivation}_6,\text{region}_5}$	-0.0170	0.0115	-0.0459	0.0007
	$\beta_{10,\text{gender}_F,\text{region}_3}$	0.0583	0.0059	0.0473	0.0697		$\beta_{11,\text{deprivation}_6,\text{region}_6}$	0.0189	0.0105	-0.0034	0.0391
	$\beta_{10,\text{gender}_F,\text{region}_4}$	-0.0318	0.0069	-0.0467	-0.0201		$\beta_{11,\text{deprivation}_6,\text{region}_7}$	0.0368	0.0129	0.0065	0.0577
	$\beta_{10,\text{gender}_F,\text{region}_5}$	-0.1037	0.0066	-0.1164	-0.0915		$\beta_{11,\text{deprivation}_6,\text{region}_8}$	-0.0014	0.0123	-0.0267	0.0223
	$\beta_{10,\text{gender}_F,\text{region}_6}$	-0.0276	0.0067	-0.0407	-0.0157		$\beta_{11,\text{deprivation}_6,\text{region}_9}$	-0.0118	0.0110	-0.0309	0.0061
	$\beta_{10,\text{gender}_F,\text{region}_7}$	-0.0357	0.0090	-0.0545	-0.0191		$\beta_{11,\text{deprivation}_7,\text{region}_1}$	-0.0662	0.0183	-0.0979	-0.0254
	$\beta_{10,\text{gender}_F,\text{region}_8}$	-0.0211	0.0056	-0.0319	-0.0102		$\beta_{11,\text{deprivation}_7,\text{region}_2}$	-0.0385	0.0097	-0.0561	-0.0175
	$\beta_{10,\text{gender}_F,\text{region}_9}$	-0.0488	0.0120	-0.0673	-0.0243		$\beta_{11,\text{deprivation}_7,\text{region}_3}$	0.0048	0.0146	-0.0244	0.0343
Deprivation:Region	$\beta_{11,\text{deprivation}_1,\text{region}_1}$	0.0663	0.0107	0.0415	0.0888		$\beta_{11,\text{deprivation}_7,\text{region}_4}$	0.0100	0.0118	-0.0222	0.0287
	$\beta_{11,\text{deprivation}_1,\text{region}_2}$	0.0935	0.0086	0.0780	0.1117		$\beta_{11,\text{deprivation}_7,\text{region}_5}$	0.0005	0.0139	-0.0218	0.0293
	$\beta_{11,\text{deprivation}_1,\text{region}_3}$	0.0162	0.0087	-0.0015	0.0323		$\beta_{11,\text{deprivation}_7,\text{region}_6}$	0.0492	0.0097	0.0304	0.0695
	$\beta_{11,\text{deprivation}_1,\text{region}_4}$	-0.0001	0.0132	-0.0306	0.0249		$\beta_{11,\text{deprivation}_7,\text{region}_7}$	0.0771	0.0186	0.0446	0.1116
	$\beta_{11,\text{deprivation}_1,\text{region}_5}$	-0.0243	0.0091	-0.0423	-0.0053		$\beta_{11,\text{deprivation}_7,\text{region}_8}$	0.0011	0.0105	-0.0207	0.0211
	$\beta_{11,\text{deprivation}_1,\text{region}_6}$	-0.0751	0.0165	-0.1104	-0.0410		$\beta_{11,\text{deprivation}_7,\text{region}_9}$	-0.0381	0.0124	-0.0632	-0.0167
	$\beta_{11,\text{deprivation}_1,\text{region}_7}$	-0.1069	0.0106	-0.1285	-0.0862		$\beta_{11,\text{deprivation}_8,\text{region}_1}$	-0.0297	0.0241	-0.0828	0.0080
	$\beta_{11,\text{deprivation}_1,\text{region}_8}$	-0.0327	0.0160	-0.0649	-0.0037		$\beta_{11,\text{deprivation}_8,\text{region}_2}$	-0.0503	0.0097	-0.0674	-0.0261
	$\beta_{11,\text{deprivation}_1,\text{region}_9}$	0.0630	0.0181	0.0292	0.0992		$\beta_{11,\text{deprivation}_8,\text{region}_3}$	-0.0250	0.0138	-0.0497	-0.0015
	$\beta_{11,\text{deprivation}_2,\text{region}_1}$	0.0574	0.0131	0.0273	0.0809		$\beta_{11,\text{deprivation}_8,\text{region}_4}$	-0.0289	0.0147	-0.0568	-0.0021
	$\beta_{11,\text{deprivation}_2,\text{region}_2}$	0.0120	0.0076	-0.0031	0.0271		$\beta_{11,\text{deprivation}_8,\text{region}_5}$	-0.0365	0.0160	-0.0651	0.0019
	$\beta_{11,\text{deprivation}_2,\text{region}_3}$	0.0171	0.0089	-0.0038	0.0336		$\beta_{11,\text{deprivation}_8,\text{region}_6}$	0.0513	0.0105	0.0324	0.0734
	$\beta_{11,\text{deprivation}_2,\text{region}_4}$	-0.0099	0.0108	-0.0316	0.0120		$\beta_{11,\text{deprivation}_8,\text{region}_7}$	0.0873	0.0213	0.0457	0.1192
	$\beta_{11,\text{deprivation}_2,\text{region}_5}$	0.0228	0.0136	-0.0007	0.0484		$\beta_{11,\text{deprivation}_8,\text{region}_8}$	0.0038	0.0131	-0.0190	0.0313
	$\beta_{11,\text{deprivation}_2,\text{region}_6}$	-0.0224	0.0092	-0.0421	-0.0053		$\beta_{11,\text{deprivation}_8,\text{region}_9}$	0.0280	0.0142	0.0029	0.0564
	$\beta_{11,\text{deprivation}_2,\text{region}_7}$	-0.0484	0.0095	-0.0680	-0.0288		$\beta_{11,\text{deprivation}_9,\text{region}_1}$	-0.0458	0.0203	-0.0769	-0.0040
	$\beta_{11,\text{deprivation}_2,\text{region}_8}$	0.0034	0.0101	-0.0148	0.0234		$\beta_{11,\text{deprivation}_9,\text{region}_2}$	-0.0437	0.0109	-0.0653	-0.0259
	$\beta_{11,\text{deprivation}_2,\text{region}_9}$	-0.0320	0.0110	-0.0481	-0.0044		$\beta_{11,\text{deprivation}_9,\text{region}_3}$	-0.0473	0.0215	-0.0862	0.0015
	$\beta_{11,\text{deprivation}_3,\text{region}_1}$	0.0282	0.0126	0.0050	0.0522		$\beta_{11,\text{deprivation}_9,\text{region}_4}$	-0.0120	0.0139	-0.0357	0.0163
	$\beta_{11,\text{deprivation}_3,\text{region}_2}$	0.0091	0.0100	-0.0100	0.0306		$\beta_{11,\text{deprivation}_9,\text{region}_5}$	-0.0024	0.0199	-0.0386	0.0332
	$\beta_{11,\text{deprivation}_3,\text{region}_3}$	0.0177	0.0118	-0.0020	0.0415		$\beta_{11,\text{deprivation}_9,\text{region}_6}$	0.0188	0.0153	-0.0069	0.0488
	$\beta_{11,\text{deprivation}_3,\text{region}_4}$	0.0130	0.0150	-0.0217	0.0435		$\beta_{11,\text{deprivation}_9,\text{region}_7}$	0.0811	0.0172	0.0538	0.1122

Covariate	Parameter	Mean	SD	%2.5	%97.5	Covariate	Parameter	Mean	SD	%2.5	%97.5
Deprivation:Region	$\beta_{11,\text{deprivation}_9,\text{region}_8}$	0.0036	0.0115	-0.0219	0.0272		$\beta_{12,\text{age}_6,\text{region}_1}$	0.0473	0.0119	0.0258	0.0736
	$\beta_{11,\text{deprivation}_9,\text{region}_9}$	0.0476	0.0149	0.0227	0.0763		$\beta_{12,\text{age}_6,\text{region}_2}$	0.0305	0.0088	0.0145	0.0487
	$\beta_{11,\text{deprivation}_{10},\text{region}_1}$	0.0349	0.0105	0.0118	0.0539		$\beta_{12,\text{age}_6,\text{region}_3}$	0.0181	0.0104	-0.0015	0.0371
	$\beta_{11,\text{deprivation}_{10},\text{region}_2}$	0.0204	0.0096	0.0040	0.0398		$\beta_{12,\text{age}_6,\text{region}_4}$	0.0167	0.0118	-0.0077	0.0357
	$\beta_{11,\text{deprivation}_{10},\text{region}_3}$	0.0019	0.0125	-0.0200	0.0286		$\beta_{12,\text{age}_6,\text{region}_5}$	-0.0028	0.0071	-0.0157	0.0113
	$\beta_{11,\text{deprivation}_{10},\text{region}_4}$	0.0031	0.0132	-0.0199	0.0363		$\beta_{12,\text{age}_6,\text{region}_6}$	-0.0169	0.0102	-0.0353	0.0014
	$\beta_{11,\text{deprivation}_{10},\text{region}_5}$	0.0066	0.0095	-0.0118	0.0241		$\beta_{12,\text{age}_6,\text{region}_7}$	-0.0091	0.0097	-0.0282	0.0107
	$\beta_{11,\text{deprivation}_{10},\text{region}_6}$	-0.0319	0.0125	-0.0573	-0.0103		$\beta_{12,\text{age}_6,\text{region}_8}$	-0.0256	0.0098	-0.0442	-0.0045
	$\beta_{11,\text{deprivation}_{10},\text{region}_7}$	-0.0200	0.0098	-0.0417	-0.0036		$\beta_{12,\text{age}_6,\text{region}_9}$	-0.0582	0.0076	-0.0741	-0.0435
	$\beta_{11,\text{deprivation}_{10},\text{region}_8}$	-0.0111	0.0137	-0.0373	0.0128		$\beta_{12,\text{age}_7,\text{region}_1}$	0.0653	0.0093	0.0475	0.0829
	$\beta_{11,\text{deprivation}_{10},\text{region}_9}$	-0.0040	0.0120	-0.0260	0.0180		$\beta_{12,\text{age}_7,\text{region}_2}$	0.0150	0.0077	0.0026	0.0336
Age:Region	$\beta_{12,\text{age}_1,\text{region}_1}$	-0.1558	0.0253	-0.2163	-0.1161		$\beta_{12,\text{age}_7,\text{region}_3}$	0.0346	0.0094	0.0145	0.0515
	$\beta_{12,\text{age}_1,\text{region}_2}$	-0.0323	0.0219	-0.0754	0.0059		$\beta_{12,\text{age}_7,\text{region}_4}$	0.0142	0.0098	-0.0050	0.0330
	$\beta_{12,\text{age}_1,\text{region}_3}$	-0.1209	0.0239	-0.1650	-0.0729		$\beta_{12,\text{age}_7,\text{region}_5}$	0.0210	0.0099	-0.0011	0.0403
	$\beta_{12,\text{age}_1,\text{region}_4}$	-0.0221	0.0283	-0.0731	0.0278		$\beta_{12,\text{age}_7,\text{region}_6}$	-0.0100	0.0112	-0.0335	0.0107
	$\beta_{12,\text{age}_1,\text{region}_5}$	-0.0218	0.0268	-0.0840	0.0259		$\beta_{12,\text{age}_7,\text{region}_7}$	-0.0155	0.0093	-0.0359	0.0016
	$\beta_{12,\text{age}_1,\text{region}_6}$	0.0492	0.0346	-0.0208	0.1071		$\beta_{12,\text{age}_7,\text{region}_8}$	-0.0485	0.0090	-0.0655	-0.0314
	$\beta_{12,\text{age}_1,\text{region}_7}$	0.0738	0.0203	0.0383	0.1152		$\beta_{12,\text{age}_7,\text{region}_9}$	-0.0761	0.0095	-0.0919	-0.0574
	$\beta_{12,\text{age}_1,\text{region}_8}$	0.0859	0.0228	0.0442	0.1348		$\beta_{12,\text{age}_8,\text{region}_1}$	0.0877	0.0107	0.0669	0.1067
	$\beta_{12,\text{age}_1,\text{region}_9}$	0.1440	0.0273	0.0938	0.1915		$\beta_{12,\text{age}_8,\text{region}_2}$	0.0208	0.0080	0.0066	0.0368
	$\beta_{12,\text{age}_2,\text{region}_1}$	-0.0652	0.0197	-0.0992	-0.0281		$\beta_{12,\text{age}_8,\text{region}_3}$	0.0364	0.0072	0.0222	0.0498
	$\beta_{12,\text{age}_2,\text{region}_2}$	-0.0341	0.0132	-0.0569	-0.0059		$\beta_{12,\text{age}_8,\text{region}_4}$	-0.0328	0.0085	-0.0469	-0.0141
	$\beta_{12,\text{age}_2,\text{region}_3}$	-0.0344	0.0165	-0.0645	-0.0011		$\beta_{12,\text{age}_8,\text{region}_5}$	-0.0091	0.0092	-0.0233	0.0127
	$\beta_{12,\text{age}_2,\text{region}_4}$	0.0113	0.0178	-0.0239	0.0444		$\beta_{12,\text{age}_8,\text{region}_6}$	-0.0106	0.0098	-0.0300	0.0081
	$\beta_{12,\text{age}_2,\text{region}_5}$	0.0005	0.0152	-0.0244	0.0297		$\beta_{12,\text{age}_8,\text{region}_7}$	0.0375	0.0090	0.0208	0.0554
	$\beta_{12,\text{age}_2,\text{region}_6}$	0.0240	0.0144	-0.0017	0.0580		$\beta_{12,\text{age}_8,\text{region}_8}$	-0.0607	0.0074	-0.0757	-0.0468
	$\beta_{12,\text{age}_2,\text{region}_7}$	-0.0362	0.0192	-0.0755	-0.0056		$\beta_{12,\text{age}_8,\text{region}_9}$	-0.0693	0.0083	-0.0881	-0.0545
	$\beta_{12,\text{age}_2,\text{region}_8}$	0.0529	0.0172	0.0219	0.0943		$\beta_{12,\text{age}_9,\text{region}_1}$	0.0575	0.0110	0.0382	0.0803
	$\beta_{12,\text{age}_2,\text{region}_9}$	0.0812	0.0137	0.0562	0.1031		$\beta_{12,\text{age}_9,\text{region}_2}$	-0.0035	0.0099	-0.0235	0.0149
	$\beta_{12,\text{age}_3,\text{region}_1}$	-0.0590	0.0190	-0.0912	-0.0209		$\beta_{12,\text{age}_9,\text{region}_3}$	0.0183	0.0086	0.0026	0.0373
	$\beta_{12,\text{age}_3,\text{region}_2}$	-0.0340	0.0133	-0.0578	-0.0081		$\beta_{12,\text{age}_9,\text{region}_4}$	-0.0365	0.0170	-0.0708	-0.0074
	$\beta_{12,\text{age}_3,\text{region}_3}$	-0.0098	0.0125	-0.0353	0.0126		$\beta_{12,\text{age}_9,\text{region}_5}$	-0.0398	0.0106	-0.0598	-0.0176
	$\beta_{12,\text{age}_3,\text{region}_4}$	0.0273	0.0157	0.0005	0.0569		$\beta_{12,\text{age}_9,\text{region}_6}$	-0.0333	0.0132	-0.0562	-0.0102
	$\beta_{12,\text{age}_3,\text{region}_5}$	0.0362	0.0124	0.0141	0.0592		$\beta_{12,\text{age}_9,\text{region}_7}$	0.0790	0.0089	0.0628	0.0985
	$\beta_{12,\text{age}_3,\text{region}_6}$	0.0170	0.0113	-0.0029	0.0396		$\beta_{12,\text{age}_9,\text{region}_8}$	-0.0217	0.0093	-0.0391	-0.0016
	$\beta_{12,\text{age}_3,\text{region}_7}$	-0.0491	0.0116	-0.0668	-0.0227		$\beta_{12,\text{age}_9,\text{region}_9}$	-0.0201	0.0098	-0.0360	0.0007
	$\beta_{12,\text{age}_3,\text{region}_8}$	0.0295	0.0114	0.0063	0.0564	Deprivation:Year	$\beta_{13,\text{deprivation}_1}$	0.0309	0.0038	0.0241	0.0384
	$\beta_{12,\text{age}_3,\text{region}_9}$	0.0420	0.0115	0.0240	0.0667		$\beta_{13,\text{deprivation}_2}$	0.0233	0.0036	0.0167	0.0319
	$\beta_{12,\text{age}_4,\text{region}_1}$	-0.0048	0.0110	-0.0293	0.0132		$\beta_{13,\text{deprivation}_3}$	0.0192	0.0043	0.0106	0.0273
	$\beta_{12,\text{age}_4,\text{region}_2}$	0.0220	0.0094	0.0040	0.0407		$\beta_{13,\text{deprivation}_4}$	0.0175	0.0041	0.0091	0.0245
	$\beta_{12,\text{age}_4,\text{region}_3}$	0.0165	0.0114	-0.0056	0.0377		$\beta_{13,\text{deprivation}_5}$	0.0028	0.0050	-0.0107	0.0108
	$\beta_{12,\text{age}_4,\text{region}_4}$	0.0053	0.0113	-0.0146	0.0295		$\beta_{13,\text{deprivation}_6}$	0.0041	0.0043	-0.0046	0.0121
	$\beta_{12,\text{age}_4,\text{region}_5}$	-0.0009	0.0096	-0.0192	0.0156		$\beta_{13,\text{deprivation}_7}$	-0.0118	0.0049	-0.0207	-0.0018
	$\beta_{12,\text{age}_4,\text{region}_6}$	0.0046	0.0104	-0.0167	0.0240		$\beta_{13,\text{deprivation}_8}$	-0.0229	0.0041	-0.0312	-0.0156
	$\beta_{12,\text{age}_4,\text{region}_7}$	-0.0396	0.0121	-0.0604	-0.0185		$\beta_{13,\text{deprivation}_9}$	-0.0296	0.0052	-0.0394	-0.0184
	$\beta_{12,\text{age}_4,\text{region}_8}$	0.0060	0.0114	-0.0129	0.0308		$\beta_{13,\text{deprivation}_{10}}$	-0.0335	0.0052	-0.0437	-0.0238
	$\beta_{12,\text{age}_4,\text{region}_9}$	-0.0092	0.0100	-0.0298	0.0065	Region:Year	$\beta_{14,\text{region}_1}$	-0.0181	0.0052	-0.0287	-0.0089
	$\beta_{12,\text{age}_5,\text{region}_1}$	0.0270	0.0127	0.0018	0.0525		$\beta_{14,\text{region}_2}$	0.0142	0.0031	0.0084	0.0209
	$\beta_{12,\text{age}_5,\text{region}_2}$	0.0156	0.0088	-0.0017	0.0334		$\beta_{14,\text{region}_3}$	-0.0016	0.0035	-0.0080	0.0062
	$\beta_{12,\text{age}_5,\text{region}_3}$	0.0413	0.0088	0.0263	0.0605		$\beta_{14,\text{region}_4}$	-0.0010	0.0042	-0.0084	0.0081
	$\beta_{12,\text{age}_5,\text{region}_4}$	0.0165	0.0113	-0.0070	0.0374		$\beta_{14,\text{region}_5}$	-0.0002	0.0040	-0.0081	0.0071
	$\beta_{12,\text{age}_5,\text{region}_5}$	0.0166	0.0122	-0.0068	0.0426		$\beta_{14,\text{region}_6}$	0.0160	0.0042	0.0099	0.0259
	$\beta_{12,\text{age}_5,\text{region}_6}$	-0.0238	0.0101	-0.0415	-0.0020		$\beta_{14,\text{region}_7}$	-0.0352	0.0037	-0.0427	-0.0284
	$\beta_{12,\text{age}_5,\text{region}_7}$	-0.0408	0.0134	-0.0656	-0.0194		$\beta_{14,\text{region}_8}$	0.0120	0.0034	0.0050	0.0187
	$\beta_{12,\text{age}_5,\text{region}_8}$	-0.0179	0.0101	-0.0410	-0.0004		$\beta_{14,\text{region}_9}$	0.0138	0.0047	0.0057	0.0229
	$\beta_{12,\text{age}_5,\text{region}_9}$	-0.0345	0.0126	-0.0561	-0.0097		σ^2	0.0017	0.0004	0.0011	0.0025

S14 Table. Estimated coefficients for the best fitting model for male lung cancer mortality.

Covariate	Parameter	Mean	SD	%2.5	%97.5	Covariate	Parameter	Mean	SD	%2.5	%97.5
Intercept	β_0	-6.5960	0.0029	-6.6010	-6.5900		β_{6,age_5}	0.0095	0.0051	-0.0007	0.0193
Age	β_{1,age_1}	-2.5210	0.0146	-2.5500	-2.4930		β_{6,age_6}	0.0221	0.0050	0.0119	0.0319
	β_{1,age_2}	-1.6130	0.0103	-1.6330	-1.5940		β_{6,age_7}	0.0036	0.0048	-0.0051	0.0137
	β_{1,age_3}	-0.8333	0.0075	-0.8482	-0.8186		β_{6,age_8}	0.0402	0.0052	0.0305	0.0500
	β_{1,age_4}	-0.1738	0.0058	-0.1853	-0.1626		β_{6,age_9}	0.1071	0.0062	0.0948	0.1193
	β_{1,age_5}	0.3197	0.0060	0.3082	0.3315	Age:Deprivation	$\beta_{7,\text{age}_1,\text{deprivation}_1}$	0.1562	0.0295	0.1036	0.2232
	β_{1,age_6}	0.7311	0.0059	0.7198	0.7431		$\beta_{7,\text{age}_2,\text{deprivation}_1}$	0.1293	0.0254	0.0759	0.1768
	β_{1,age_7}	1.0730	0.0051	1.0640	1.0830		$\beta_{7,\text{age}_3,\text{deprivation}_1}$	0.1662	0.0180	0.1319	0.2007
	β_{1,age_8}	1.2770	0.0057	1.2660	1.2880		$\beta_{7,\text{age}_4,\text{deprivation}_1}$	0.1180	0.0147	0.0891	0.1464
	β_{1,age_9}	1.7400	0.0057	1.7280	1.7510		$\beta_{7,\text{age}_5,\text{deprivation}_1}$	0.0650	0.0134	0.0377	0.0897
Year	β_2	-0.1154	0.0027	-0.1207	-0.1101		$\beta_{7,\text{age}_6,\text{deprivation}_1}$	-0.0082	0.0128	-0.0343	0.0168
AAD	β_3	0.4974	0.0077	0.4836	0.5114		$\beta_{7,\text{age}_7,\text{deprivation}_1}$	-0.0979	0.0131	-0.1240	-0.0729
Region	$\beta_{4,\text{region}_1}$	-0.0255	0.0077	-0.0403	-0.0102		$\beta_{7,\text{age}_8,\text{deprivation}_1}$	-0.2087	0.0141	-0.2361	-0.1802
	$\beta_{4,\text{region}_2}$	0.0988	0.0053	0.0882	0.1089		$\beta_{7,\text{age}_9,\text{deprivation}_1}$	-0.3198	0.0171	-0.3552	-0.2877
	$\beta_{4,\text{region}_3}$	0.0440	0.0057	0.0333	0.0565		$\beta_{7,\text{age}_1,\text{deprivation}_2}$	0.0378	0.0467	-0.0668	0.1343
	$\beta_{4,\text{region}_4}$	0.0812	0.0065	0.0677	0.0939		$\beta_{7,\text{age}_2,\text{deprivation}_2}$	0.1544	0.0309	0.0967	0.2104
	$\beta_{4,\text{region}_5}$	0.0493	0.0055	0.0378	0.0601		$\beta_{7,\text{age}_3,\text{deprivation}_2}$	0.1079	0.0206	0.0666	0.1454
	$\beta_{4,\text{region}_6}$	-0.0047	0.0065	-0.0183	0.0087		$\beta_{7,\text{age}_4,\text{deprivation}_2}$	0.0939	0.0156	0.0661	0.1232
	$\beta_{4,\text{region}_7}$	-0.3036	0.0061	-0.3160	-0.2926		$\beta_{7,\text{age}_5,\text{deprivation}_2}$	0.0414	0.0145	0.0144	0.0703
	$\beta_{4,\text{region}_8}$	0.0695	0.0055	0.0592	0.0808		$\beta_{7,\text{age}_6,\text{deprivation}_2}$	-0.0232	0.0137	-0.0489	0.0035
	$\beta_{4,\text{region}_9}$	-0.0090	0.0064	-0.0216	0.0042		$\beta_{7,\text{age}_7,\text{deprivation}_2}$	-0.0649	0.0140	-0.0916	-0.0349
Deprivation	$\beta_{5,\text{deprivation}_1}$	1.4240	0.0135	1.4010	1.4510		$\beta_{7,\text{age}_8,\text{deprivation}_2}$	-0.1175	0.0137	-0.1444	-0.0915
	$\beta_{5,\text{deprivation}_2}$	1.0230	0.0096	1.0050	1.0410		$\beta_{7,\text{age}_9,\text{deprivation}_2}$	-0.2297	0.0177	-0.2646	-0.1937
	$\beta_{5,\text{deprivation}_3}$	0.6358	0.0095	0.6193	0.6545		$\beta_{7,\text{age}_1,\text{deprivation}_3}$	0.0218	0.0457	-0.0593	0.1044
	$\beta_{5,\text{deprivation}_4}$	0.4453	0.0076	0.4319	0.4620		$\beta_{7,\text{age}_2,\text{deprivation}_3}$	0.0740	0.0253	0.0174	0.1200
	$\beta_{5,\text{deprivation}_5}$	0.1217	0.0082	0.1053	0.1370		$\beta_{7,\text{age}_3,\text{deprivation}_3}$	0.0510	0.0251	0.0022	0.1011
	$\beta_{5,\text{deprivation}_6}$	-0.1861	0.0086	-0.2020	-0.1680		$\beta_{7,\text{age}_4,\text{deprivation}_3}$	0.0124	0.0188	-0.0250	0.0483
	$\beta_{5,\text{deprivation}_7}$	-0.4728	0.0116	-0.4916	-0.4467		$\beta_{7,\text{age}_5,\text{deprivation}_3}$	0.0479	0.0158	0.0175	0.0771
	$\beta_{5,\text{deprivation}_8}$	-0.6928	0.0123	-0.7182	-0.6691		$\beta_{7,\text{age}_6,\text{deprivation}_3}$	0.0281	0.0160	-0.0017	0.0632
	$\beta_{5,\text{deprivation}_9}$	-0.9459	0.0099	-0.9635	-0.9283		$\beta_{7,\text{age}_7,\text{deprivation}_3}$	-0.0301	0.0121	-0.0535	-0.0074
	$\beta_{5,\text{deprivation}_{10}}$	-1.3530	0.0129	-1.3760	-1.3260		$\beta_{7,\text{age}_8,\text{deprivation}_3}$	-0.0885	0.0159	-0.1188	-0.0577
Age:Year	β_{6,age_1}	-0.0629	0.0146	-0.0935	-0.0350		$\beta_{7,\text{age}_9,\text{deprivation}_3}$	-0.1165	0.0175	-0.1503	-0.0792
	β_{6,age_2}	-0.0725	0.0093	-0.0894	-0.0542		$\beta_{7,\text{age}_1,\text{deprivation}_4}$	0.0819	0.0322	0.0162	0.1385
	β_{6,age_3}	-0.0331	0.0080	-0.0496	-0.0155		$\beta_{7,\text{age}_2,\text{deprivation}_4}$	0.0489	0.0269	-0.0042	0.0982
	β_{6,age_4}	-0.0140	0.0075	-0.0280	0.0036		$\beta_{7,\text{age}_3,\text{deprivation}_4}$	0.0355	0.0171	0.0019	0.0686

Covariate	Parameter	Mean	SD	%2.5	%97.5	Covariate	Parameter	Mean	SD	%2.5	%97.5
Age:Deprivation	$\beta_{7,\text{age}_4,\text{deprivation}_4}$	0.0357	0.0181	-0.0022	0.0689		$\beta_{7,\text{age}_4,\text{deprivation}_8}$	-0.0591	0.0186	-0.0955	-0.0206
	$\beta_{7,\text{age}_5,\text{deprivation}_4}$	0.0109	0.0148	-0.0194	0.0394		$\beta_{7,\text{age}_5,\text{deprivation}_8}$	-0.0377	0.0173	-0.0739	-0.0050
	$\beta_{7,\text{age}_6,\text{deprivation}_4}$	-0.0256	0.0126	-0.0520	-0.0030		$\beta_{7,\text{age}_6,\text{deprivation}_8}$	0.0316	0.0166	-0.0010	0.0623
	$\beta_{7,\text{age}_7,\text{deprivation}_4}$	-0.0162	0.0130	-0.0423	0.0078		$\beta_{7,\text{age}_7,\text{deprivation}_8}$	0.0657	0.0155	0.0374	0.0964
	$\beta_{7,\text{age}_8,\text{deprivation}_4}$	-0.0533	0.0132	-0.0778	-0.0266		$\beta_{7,\text{age}_8,\text{deprivation}_8}$	0.0878	0.0157	0.0579	0.1211
	$\beta_{7,\text{age}_9,\text{deprivation}_4}$	-0.1179	0.0161	-0.1536	-0.0863		$\beta_{7,\text{age}_9,\text{deprivation}_8}$	0.1486	0.0179	0.1131	0.1829
	$\beta_{7,\text{age}_1,\text{deprivation}_5}$	-0.0281	0.0414	-0.0998	0.0641		$\beta_{7,\text{age}_1,\text{deprivation}_9}$	-0.0599	0.0430	-0.1464	0.0320
	$\beta_{7,\text{age}_2,\text{deprivation}_5}$	0.0394	0.0281	-0.0205	0.0866		$\beta_{7,\text{age}_2,\text{deprivation}_9}$	-0.1165	0.0333	-0.1785	-0.0512
	$\beta_{7,\text{age}_3,\text{deprivation}_5}$	0.0270	0.0231	-0.0144	0.0734		$\beta_{7,\text{age}_3,\text{deprivation}_9}$	-0.1149	0.0314	-0.1787	-0.0558
	$\beta_{7,\text{age}_4,\text{deprivation}_5}$	0.0065	0.0173	-0.0272	0.0384		$\beta_{7,\text{age}_4,\text{deprivation}_9}$	-0.0399	0.0181	-0.0736	-0.0046
	$\beta_{7,\text{age}_5,\text{deprivation}_5}$	0.0185	0.0169	-0.0128	0.0551		$\beta_{7,\text{age}_5,\text{deprivation}_9}$	-0.0642	0.0170	-0.0950	-0.0283
	$\beta_{7,\text{age}_6,\text{deprivation}_5}$	-0.0099	0.0158	-0.0362	0.0253		$\beta_{7,\text{age}_6,\text{deprivation}_9}$	0.0121	0.0170	-0.0220	0.0436
	$\beta_{7,\text{age}_7,\text{deprivation}_5}$	0.0002	0.0141	-0.0264	0.0297		$\beta_{7,\text{age}_7,\text{deprivation}_9}$	0.0430	0.0176	0.0122	0.0762
	$\beta_{7,\text{age}_8,\text{deprivation}_5}$	-0.0145	0.0166	-0.0448	0.0165		$\beta_{7,\text{age}_8,\text{deprivation}_9}$	0.1315	0.0174	0.1005	0.1699
	$\beta_{7,\text{age}_9,\text{deprivation}_5}$	-0.0389	0.0167	-0.0689	-0.0053		$\beta_{7,\text{age}_9,\text{deprivation}_9}$	0.2089	0.0187	0.1710	0.2474
	$\beta_{7,\text{age}_1,\text{deprivation}_6}$	0.0322	0.0394	-0.0456	0.1077		$\beta_{7,\text{age}_1,\text{deprivation}_{10}}$	-0.2310	0.0550	-0.3173	-0.0980
	$\beta_{7,\text{age}_2,\text{deprivation}_6}$	-0.0195	0.0308	-0.0801	0.0376		$\beta_{7,\text{age}_2,\text{deprivation}_{10}}$	-0.0963	0.0452	-0.1825	-0.0233
	$\beta_{7,\text{age}_3,\text{deprivation}_6}$	-0.0152	0.0262	-0.0647	0.0343		$\beta_{7,\text{age}_3,\text{deprivation}_{10}}$	-0.1190	0.0372	-0.1921	-0.0498
	$\beta_{7,\text{age}_4,\text{deprivation}_6}$	-0.0455	0.0184	-0.0840	-0.0099		$\beta_{7,\text{age}_4,\text{deprivation}_{10}}$	-0.0796	0.0236	-0.1261	-0.0336
	$\beta_{7,\text{age}_5,\text{deprivation}_6}$	-0.0039	0.0170	-0.0332	0.0334		$\beta_{7,\text{age}_5,\text{deprivation}_{10}}$	-0.0471	0.0209	-0.0833	-0.0015
	$\beta_{7,\text{age}_6,\text{deprivation}_6}$	-0.0178	0.0159	-0.0525	0.0106		$\beta_{7,\text{age}_6,\text{deprivation}_{10}}$	0.0280	0.0205	-0.0104	0.0690
	$\beta_{7,\text{age}_7,\text{deprivation}_6}$	0.0208	0.0160	-0.0134	0.0492		$\beta_{7,\text{age}_7,\text{deprivation}_{10}}$	0.0639	0.0190	0.0264	0.0984
	$\beta_{7,\text{age}_8,\text{deprivation}_6}$	0.0165	0.0154	-0.0136	0.0467		$\beta_{7,\text{age}_8,\text{deprivation}_{10}}$	0.1813	0.0162	0.1501	0.2145
	$\beta_{7,\text{age}_9,\text{deprivation}_6}$	0.0324	0.0163	-0.0009	0.0618		$\beta_{7,\text{age}_9,\text{deprivation}_{10}}$	0.2998	0.0212	0.2622	0.3418
	$\beta_{7,\text{age}_1,\text{deprivation}_7}$	-0.0214	0.0667	-0.1422	0.1021	Deprivation:Year	$\beta_{8,\text{deprivation}_1}$	0.0386	0.0052	0.0286	0.0488
	$\beta_{7,\text{age}_2,\text{deprivation}_7}$	-0.0679	0.0253	-0.1216	-0.0227		$\beta_{8,\text{deprivation}_2}$	0.0238	0.0050	0.0135	0.0334
	$\beta_{7,\text{age}_3,\text{deprivation}_7}$	-0.0366	0.0251	-0.0848	0.0155		$\beta_{8,\text{deprivation}_3}$	0.0218	0.0061	0.0106	0.0339
	$\beta_{7,\text{age}_4,\text{deprivation}_7}$	-0.0423	0.0204	-0.0806	-0.0018		$\beta_{8,\text{deprivation}_4}$	0.0218	0.0057	0.0102	0.0327
	$\beta_{7,\text{age}_5,\text{deprivation}_7}$	-0.0308	0.0195	-0.0686	0.0049		$\beta_{8,\text{deprivation}_5}$	0.0017	0.0067	-0.0112	0.0153
	$\beta_{7,\text{age}_6,\text{deprivation}_7}$	-0.0152	0.0168	-0.0512	0.0182		$\beta_{8,\text{deprivation}_6}$	-0.0007	0.0060	-0.0129	0.0107
	$\beta_{7,\text{age}_7,\text{deprivation}_7}$	0.0156	0.0165	-0.0160	0.0483		$\beta_{8,\text{deprivation}_7}$	-0.0120	0.0066	-0.0258	-0.0005
	$\beta_{7,\text{age}_8,\text{deprivation}_7}$	0.0654	0.0180	0.0289	0.1008		$\beta_{8,\text{deprivation}_8}$	-0.0201	0.0058	-0.0311	-0.0088
	$\beta_{7,\text{age}_9,\text{deprivation}_7}$	0.1333	0.0179	0.0971	0.1678		$\beta_{8,\text{deprivation}_9}$	-0.0402	0.0065	-0.0534	-0.0274
	$\beta_{7,\text{age}_1,\text{deprivation}_8}$	0.0105	0.0609	-0.0927	0.1337		$\beta_{8,\text{deprivation}_{10}}$	-0.0346	0.0080	-0.0507	-0.0195
	$\beta_{7,\text{age}_2,\text{deprivation}_8}$	-0.1457	0.0357	-0.2146	-0.0774		σ^2	0.0014	0.0004	0.0007	0.0022
	$\beta_{7,\text{age}_3,\text{deprivation}_8}$	-0.1017	0.0260	-0.1462	-0.0525						

S15 Table. Estimated coefficients for the best fitting model for female lung cancer mortality.

Covariate	Parameter	Mean	SD	%2.5	%97.5	Covariate	Parameter	Mean	SD	%2.5	%97.5
Intercept	β_0	-7.0190	0.0042	-7.0280	-7.0100		$\beta_{8,\text{age}_4,\text{deprivation}_3}$	0.0354	0.0218	-0.0104	0.0748
Age	β_{1,age_1}	-2.2600	0.0272	-2.3110	-2.2100		$\beta_{8,\text{age}_5,\text{deprivation}_3}$	0.0366	0.0158	0.0068	0.0662
	β_{1,age_2}	-1.3820	0.0188	-1.4180	-1.3440		$\beta_{8,\text{age}_6,\text{deprivation}_3}$	0.0236	0.0178	-0.0197	0.0540
	β_{1,age_3}	-0.6425	0.0101	-0.6654	-0.6262		$\beta_{8,\text{age}_7,\text{deprivation}_3}$	-0.0394	0.0172	-0.0733	-0.0025
	β_{1,age_4}	-0.1273	0.0112	-0.1503	-0.1058		$\beta_{8,\text{age}_8,\text{deprivation}_3}$	-0.1018	0.0172	-0.1343	-0.0649
	β_{1,age_5}	0.3165	0.0092	0.2997	0.3340		$\beta_{8,\text{age}_9,\text{deprivation}_3}$	-0.1682	0.0155	-0.2014	-0.1403
	β_{1,age_6}	0.6244	0.0089	0.6076	0.6410		$\beta_{8,\text{age}_1,\text{deprivation}_4}$	0.0381	0.0490	-0.0608	0.1474
	β_{1,age_7}	0.9225	0.0100	0.9054	0.9397		$\beta_{8,\text{age}_2,\text{deprivation}_4}$	0.0523	0.0296	-0.0021	0.1155
	β_{1,age_8}	1.1060	0.0086	1.0890	1.1230		$\beta_{8,\text{age}_3,\text{deprivation}_4}$	-0.0023	0.0226	-0.0446	0.0445
	β_{1,age_9}	1.4420	0.0087	1.4270	1.4610		$\beta_{8,\text{age}_4,\text{deprivation}_4}$	0.0252	0.0209	-0.0133	0.0671
Year	β_2	-0.0007	0.0033	-0.0070	0.0061		$\beta_{8,\text{age}_5,\text{deprivation}_4}$	0.0446	0.0192	0.0086	0.0784
AAD	β_3	0.9768	0.0123	0.9573	0.9968		$\beta_{8,\text{age}_6,\text{deprivation}_4}$	0.0240	0.0167	-0.0111	0.0536
Region	$\beta_{4,\text{region}_1}$	-0.1838	0.0135	-0.2104	-0.1558		$\beta_{8,\text{age}_7,\text{deprivation}_4}$	-0.0102	0.0176	-0.0422	0.0260
	$\beta_{4,\text{region}_2}$	0.1030	0.0078	0.0885	0.1172		$\beta_{8,\text{age}_8,\text{deprivation}_4}$	-0.0642	0.0180	-0.0992	-0.0321
	$\beta_{4,\text{region}_3}$	-0.0075	0.0091	-0.0266	0.0091		$\beta_{8,\text{age}_9,\text{deprivation}_4}$	-0.1076	0.0189	-0.1442	-0.0727
	$\beta_{4,\text{region}_4}$	0.1457	0.0111	0.1255	0.1667		$\beta_{8,\text{age}_1,\text{deprivation}_5}$	0.0227	0.0599	-0.1302	0.1211
	$\beta_{4,\text{region}_5}$	0.0150	0.0084	-0.0013	0.0302		$\beta_{8,\text{age}_2,\text{deprivation}_5}$	0.0082	0.0329	-0.0466	0.0898
	$\beta_{4,\text{region}_6}$	0.0613	0.0104	0.0387	0.0792		$\beta_{8,\text{age}_3,\text{deprivation}_5}$	0.0260	0.0268	-0.0260	0.0728
	$\beta_{4,\text{region}_7}$	-0.5408	0.0111	-0.5620	-0.5169		$\beta_{8,\text{age}_4,\text{deprivation}_5}$	-0.0029	0.0220	-0.0504	0.0353
	$\beta_{4,\text{region}_8}$	0.2240	0.0090	0.2033	0.2380		$\beta_{8,\text{age}_5,\text{deprivation}_5}$	-0.0050	0.0200	-0.0434	0.0394
	$\beta_{4,\text{region}_9}$	0.1831	0.0106	0.1626	0.2039		$\beta_{8,\text{age}_6,\text{deprivation}_5}$	-0.0065	0.0156	-0.0364	0.0220
Deprivation	$\beta_{5,\text{deprivation}_1}$	2.2070	0.0227	2.1660	2.2460		$\beta_{8,\text{age}_7,\text{deprivation}_5}$	-0.0033	0.0159	-0.0331	0.0281
	$\beta_{5,\text{deprivation}_2}$	1.5710	0.0155	1.5390	1.5970		$\beta_{8,\text{age}_8,\text{deprivation}_5}$	0.0041	0.0165	-0.0285	0.0369
	$\beta_{5,\text{deprivation}_3}$	0.9549	0.0119	0.9315	0.9737		$\beta_{8,\text{age}_9,\text{deprivation}_5}$	-0.0433	0.0192	-0.0754	0.0069
	$\beta_{5,\text{deprivation}_4}$	0.6853	0.0106	0.6656	0.7032		$\beta_{8,\text{age}_1,\text{deprivation}_6}$	0.0138	0.0492	-0.0832	0.1181
	$\beta_{5,\text{deprivation}_5}$	0.1763	0.0114	0.1529	0.1979		$\beta_{8,\text{age}_2,\text{deprivation}_6}$	-0.1024	0.0464	-0.1843	-0.0092
	$\beta_{5,\text{deprivation}_6}$	-0.3076	0.0102	-0.3235	-0.2842		$\beta_{8,\text{age}_3,\text{deprivation}_6}$	0.0062	0.0251	-0.0427	0.0583
	$\beta_{5,\text{deprivation}_7}$	-0.7092	0.0127	-0.7333	-0.6808		$\beta_{8,\text{age}_4,\text{deprivation}_6}$	0.0288	0.0261	-0.0223	0.0832
	$\beta_{5,\text{deprivation}_8}$	-1.0790	0.0145	-1.1070	-1.0440		$\beta_{8,\text{age}_5,\text{deprivation}_6}$	-0.0505	0.0215	-0.0907	-0.0120
	$\beta_{5,\text{deprivation}_9}$	-1.4690	0.0169	-1.4940	-1.4340		$\beta_{8,\text{age}_6,\text{deprivation}_6}$	-0.0134	0.0212	-0.0578	0.0293
	$\beta_{5,\text{deprivation}_{10}}$	-2.0300	0.0215	-2.0650	-1.9870		$\beta_{8,\text{age}_7,\text{deprivation}_6}$	0.0224	0.0178	-0.0135	0.0566
Year^2	β_6	-0.0255	0.0035	-0.0336	-0.0194		$\beta_{8,\text{age}_8,\text{deprivation}_6}$	0.0314	0.0173	-0.0022	0.0618
Age:Year	β_{7,age_1}	-0.1492	0.0132	-0.1709	-0.1170		$\beta_{8,\text{age}_9,\text{deprivation}_6}$	0.0636	0.0191	0.0292	0.1089
	β_{7,age_2}	-0.1236	0.0106	-0.1440	-0.1039		$\beta_{8,\text{age}_1,\text{deprivation}_7}$	-0.0100	0.0543	-0.1214	0.0756
	β_{7,age_3}	-0.0415	0.0107	-0.0614	-0.0192		$\beta_{8,\text{age}_2,\text{deprivation}_7}$	-0.0414	0.0414	-0.1308	0.0255
	β_{7,age_4}	0.0091	0.0070	-0.0047	0.0220		$\beta_{8,\text{age}_3,\text{deprivation}_7}$	-0.0589	0.0236	-0.1035	-0.0176
	β_{7,age_5}	0.0529	0.0061	0.0401	0.0641		$\beta_{8,\text{age}_4,\text{deprivation}_7}$	-0.0559	0.0211	-0.0936	-0.0060
	β_{7,age_6}	0.0221	0.0055	0.0116	0.0328		$\beta_{8,\text{age}_5,\text{deprivation}_7}$	0.0037	0.0207	-0.0358	0.0420
	β_{7,age_7}	-0.0125	0.0053	-0.0225	-0.0015		$\beta_{8,\text{age}_6,\text{deprivation}_7}$	0.0261	0.0172	-0.0105	0.0574
	β_{7,age_8}	0.0542	0.0057	0.0426	0.0650		$\beta_{8,\text{age}_7,\text{deprivation}_7}$	0.0303	0.0179	-0.0050	0.0645
	β_{7,age_9}	0.1885	0.0060	0.1773	0.2000		$\beta_{8,\text{age}_8,\text{deprivation}_7}$	0.0318	0.0206	-0.0081	0.0720
Age:Deprivation	$\beta_{8,\text{age}_1,\text{deprivation}_1}$	0.2090	0.0367	0.1381	0.2735		$\beta_{8,\text{age}_9,\text{deprivation}_7}$	0.0743	0.0205	0.0340	0.1196
	$\beta_{8,\text{age}_2,\text{deprivation}_1}$	0.1637	0.0305	0.1052	0.2179		$\beta_{8,\text{age}_1,\text{deprivation}_8}$	-0.1773	0.0506	-0.2795	-0.0777
	$\beta_{8,\text{age}_3,\text{deprivation}_1}$	0.1107	0.0210	0.0678	0.1498		$\beta_{8,\text{age}_2,\text{deprivation}_8}$	0.0038	0.0278	-0.0501	0.0585
	$\beta_{8,\text{age}_4,\text{deprivation}_1}$	0.0796	0.0188	0.0418	0.1144		$\beta_{8,\text{age}_3,\text{deprivation}_8}$	-0.0549	0.0274	-0.1054	-0.0026
	$\beta_{8,\text{age}_5,\text{deprivation}_1}$	0.0540	0.0167	0.0218	0.0862		$\beta_{8,\text{age}_4,\text{deprivation}_8}$	-0.0544	0.0249	-0.1041	-0.0026
	$\beta_{8,\text{age}_6,\text{deprivation}_1}$	0.0078	0.0152	-0.0231	0.0368		$\beta_{8,\text{age}_5,\text{deprivation}_8}$	-0.0202	0.0231	-0.0614	0.0284
	$\beta_{8,\text{age}_7,\text{deprivation}_1}$	-0.1145	0.0152	-0.1434	-0.0828		$\beta_{8,\text{age}_6,\text{deprivation}_8}$	0.0108	0.0192	-0.0250	0.0458
	$\beta_{8,\text{age}_8,\text{deprivation}_1}$	-0.2028	0.0157	-0.2317	-0.1709		$\beta_{8,\text{age}_7,\text{deprivation}_8}$	0.0382	0.0182	0.0006	0.0756
	$\beta_{8,\text{age}_9,\text{deprivation}_1}$	-0.3075	0.0173	-0.3408	-0.2741		$\beta_{8,\text{age}_8,\text{deprivation}_8}$	0.1040	0.0192	0.0619	0.1395
	$\beta_{8,\text{age}_1,\text{deprivation}_2}$	0.0490	0.0338	-0.0278	0.1185		$\beta_{8,\text{age}_9,\text{deprivation}_8}$	0.1500	0.0209	0.1083	0.1904
	$\beta_{8,\text{age}_2,\text{deprivation}_2}$	0.1162	0.0259	0.0731	0.1693		$\beta_{8,\text{age}_1,\text{deprivation}_9}$	-0.1105	0.0445	-0.2095	-0.0262
	$\beta_{8,\text{age}_3,\text{deprivation}_2}$	0.0954	0.0219	0.0558	0.1406		$\beta_{8,\text{age}_2,\text{deprivation}_9}$	-0.0915	0.0377	-0.1679	-0.0080
	$\beta_{8,\text{age}_4,\text{deprivation}_2}$	0.0671	0.0165	0.0336	0.1001		$\beta_{8,\text{age}_3,\text{deprivation}_9}$	-0.0833	0.0261	-0.1333	-0.0261
	$\beta_{8,\text{age}_5,\text{deprivation}_2}$	0.0863	0.0138	0.0601	0.1111		$\beta_{8,\text{age}_4,\text{deprivation}_9}$	-0.0401	0.0238	-0.0858	0.0052
	$\beta_{8,\text{age}_6,\text{deprivation}_2}$	-0.0144	0.0156	-0.0428	0.0187		$\beta_{8,\text{age}_5,\text{deprivation}_9}$	-0.0667	0.0203	-0.1055	-0.0192
	$\beta_{8,\text{age}_7,\text{deprivation}_2}$	-0.0495	0.0136	-0.0771	-0.0230		$\beta_{8,\text{age}_6,\text{deprivation}_9}$	-0.0220	0.0178	-0.0565	0.0143
	$\beta_{8,\text{age}_8,\text{deprivation}_2}$	-0.1317	0.0150	-0.1637	-0.1038		$\beta_{8,\text{age}_7,\text{deprivation}_9}$	0.0430	0.0179	0.0078	0.0780
	$\beta_{8,\text{age}_9,\text{deprivation}_2}$	-0.2184	0.0167	-0.2474	-0.1863		$\beta_{8,\text{age}_8,\text{deprivation}_9}$	0.1364	0.0182	0.1046	0.1711
	$\beta_{8,\text{age}_1,\text{deprivation}_3}$	0.0986	0.0430	0.0327	0.2043		$\beta_{8,\text{age}_9,\text{deprivation}_9}$	0.2346	0.0175	0.1977	0.2646
	$\beta_{8,\text{age}_2,\text{deprivation}_3}$	0.0677	0.0282	0.0119	0.1219		$\beta_{8,\text{age}_1,\text{deprivation}_{10}}$	-0.1333	0.0554	-0.2286	0.0261
	$\beta_{8,\text{age}_3,\text{deprivation}_3}$	0.0475	0.0266	-0.0015	0.1144		$\beta_{8,\text{age}_2,\text{deprivation}_{10}}$	-0.1767	0.0437	-0.2506	-0.0889

Covariate	Parameter	Mean	SD	%2.5	%97.5	Covariate	Parameter	Mean	SD	%2.5	%97.5
Age:Deprivation	$\beta_{8,\text{age}_3,\text{deprivation}_{10}}$	-0.0864	0.0327	-0.1575	-0.0273		$\beta_{10,\text{age}_3,\text{region}_6}$	0.0238	0.0264	-0.0217	0.0867
	$\beta_{8,\text{age}_4,\text{deprivation}_{10}}$	-0.0829	0.0245	-0.1372	-0.0384		$\beta_{10,\text{age}_4,\text{region}_6}$	0.0117	0.0217	-0.0317	0.0476
	$\beta_{8,\text{age}_5,\text{deprivation}_{10}}$	-0.0828	0.0260	-0.1305	-0.0270		$\beta_{10,\text{age}_5,\text{region}_6}$	-0.0052	0.0200	-0.0426	0.0381
	$\beta_{8,\text{age}_6,\text{deprivation}_{10}}$	-0.0361	0.0241	-0.0819	0.0144		$\beta_{10,\text{age}_6,\text{region}_6}$	-0.0068	0.0183	-0.0444	0.0297
	$\beta_{8,\text{age}_7,\text{deprivation}_{10}}$	0.0829	0.0225	0.0397	0.1268		$\beta_{10,\text{age}_7,\text{region}_6}$	-0.0329	0.0159	-0.0644	-0.0041
	$\beta_{8,\text{age}_8,\text{deprivation}_{10}}$	0.1927	0.0226	0.1510	0.2321		$\beta_{10,\text{age}_8,\text{region}_6}$	-0.0101	0.0162	-0.0417	0.0202
	$\beta_{8,\text{age}_9,\text{deprivation}_{10}}$	0.3224	0.0214	0.2795	0.3590		$\beta_{10,\text{age}_9,\text{region}_6}$	-0.0170	0.0189	-0.0610	0.0198
AAD:Region	$\beta_{9,\text{region}_1}$	-0.0165	0.0081	-0.0321	-0.0006		$\beta_{10,\text{age}_1,\text{region}_7}$	-0.0012	0.0433	-0.0743	0.0832
	$\beta_{9,\text{region}_2}$	-0.0422	0.0063	-0.0543	-0.0291		$\beta_{10,\text{age}_2,\text{region}_7}$	-0.1548	0.0312	-0.2123	-0.0877
	$\beta_{9,\text{region}_3}$	0.0003	0.0070	-0.0140	0.0130		$\beta_{10,\text{age}_3,\text{region}_7}$	-0.1108	0.0227	-0.1612	-0.0618
	$\beta_{9,\text{region}_4}$	0.0141	0.0093	-0.0033	0.0338		$\beta_{10,\text{age}_4,\text{region}_7}$	-0.0479	0.0222	-0.0895	-0.0102
	$\beta_{9,\text{region}_5}$	0.0101	0.0072	-0.0031	0.0248		$\beta_{10,\text{age}_5,\text{region}_7}$	-0.0093	0.0173	-0.0420	0.0259
	$\beta_{9,\text{region}_6}$	0.0244	0.0079	0.0094	0.0398		$\beta_{10,\text{age}_6,\text{region}_7}$	-0.0067	0.0176	-0.0401	0.0262
	$\beta_{9,\text{region}_7}$	0.0692	0.0079	0.0534	0.0845		$\beta_{10,\text{age}_7,\text{region}_7}$	0.0310	0.0171	-0.0008	0.0647
	$\beta_{9,\text{region}_8}$	-0.0178	0.0068	-0.0314	-0.0052		$\beta_{10,\text{age}_8,\text{region}_7}$	0.1182	0.0167	0.0829	0.1491
	$\beta_{9,\text{region}_9}$	-0.0416	0.0075	-0.0550	-0.0250		$\beta_{10,\text{age}_9,\text{region}_7}$	0.1815	0.0158	0.1515	0.2128
Age:Region	$\beta_{10,\text{age}_1,\text{region}_1}$	-0.1674	0.0423	-0.2475	-0.0803		$\beta_{10,\text{age}_1,\text{region}_8}$	0.0693	0.0346	0.0054	0.1385
	$\beta_{10,\text{age}_2,\text{region}_1}$	-0.0792	0.0415	-0.1556	0.0019		$\beta_{10,\text{age}_2,\text{region}_8}$	0.0828	0.0271	0.0335	0.1370
	$\beta_{10,\text{age}_3,\text{region}_1}$	-0.0346	0.0265	-0.0894	0.0172		$\beta_{10,\text{age}_3,\text{region}_8}$	0.0336	0.0209	-0.0050	0.0808
	$\beta_{10,\text{age}_4,\text{region}_1}$	0.0005	0.0224	-0.0429	0.0426		$\beta_{10,\text{age}_4,\text{region}_8}$	0.0256	0.0190	-0.0106	0.0634
	$\beta_{10,\text{age}_5,\text{region}_1}$	-0.0095	0.0199	-0.0482	0.0289		$\beta_{10,\text{age}_5,\text{region}_8}$	-0.0210	0.0155	-0.0545	0.0072
	$\beta_{10,\text{age}_6,\text{region}_1}$	0.0529	0.0181	0.0175	0.0871		$\beta_{10,\text{age}_6,\text{region}_8}$	-0.0185	0.0145	-0.0472	0.0075
	$\beta_{10,\text{age}_7,\text{region}_1}$	0.0968	0.0199	0.0558	0.1354		$\beta_{10,\text{age}_7,\text{region}_8}$	-0.0654	0.0164	-0.0932	-0.0302
	$\beta_{10,\text{age}_8,\text{region}_1}$	0.0822	0.0181	0.0449	0.1175		$\beta_{10,\text{age}_8,\text{region}_8}$	-0.0879	0.0150	-0.1156	-0.0568
	$\beta_{10,\text{age}_9,\text{region}_1}$	0.0583	0.0207	0.0179	0.0971		$\beta_{10,\text{age}_9,\text{region}_8}$	-0.0184	0.0139	-0.0445	0.0096
	$\beta_{10,\text{age}_1,\text{region}_2}$	-0.0651	0.0432	-0.1426	0.0224		$\beta_{10,\text{age}_1,\text{region}_9}$	0.2040	0.0434	0.1274	0.3070
	$\beta_{10,\text{age}_2,\text{region}_2}$	-0.0429	0.0321	-0.1158	0.0135		$\beta_{10,\text{age}_2,\text{region}_9}$	0.1568	0.0322	0.0853	0.2129
	$\beta_{10,\text{age}_3,\text{region}_2}$	-0.0498	0.0244	-0.0963	0.0029		$\beta_{10,\text{age}_3,\text{region}_9}$	0.0319	0.0296	-0.0201	0.0953
	$\beta_{10,\text{age}_4,\text{region}_2}$	0.0350	0.0179	0.0010	0.0750		$\beta_{10,\text{age}_4,\text{region}_9}$	-0.0272	0.0221	-0.0704	0.0161
	$\beta_{10,\text{age}_5,\text{region}_2}$	0.0309	0.0162	0.0003	0.0619		$\beta_{10,\text{age}_5,\text{region}_9}$	-0.0451	0.0191	-0.0817	-0.0096
	$\beta_{10,\text{age}_6,\text{region}_2}$	0.0425	0.0134	0.0153	0.0672		$\beta_{10,\text{age}_6,\text{region}_9}$	-0.0778	0.0160	-0.1089	-0.0450
	$\beta_{10,\text{age}_7,\text{region}_2}$	0.0496	0.0151	0.0206	0.0780		$\beta_{10,\text{age}_7,\text{region}_9}$	-0.1146	0.0171	-0.1482	-0.0815
	$\beta_{10,\text{age}_8,\text{region}_2}$	0.0205	0.0152	-0.0084	0.0500		$\beta_{10,\text{age}_8,\text{region}_9}$	-0.1059	0.0156	-0.1375	-0.0781
	$\beta_{10,\text{age}_9,\text{region}_2}$	-0.0208	0.0176	-0.0524	0.0159		$\beta_{10,\text{age}_9,\text{region}_9}$	-0.0220	0.0182	-0.0581	0.0097
	$\beta_{10,\text{age}_1,\text{region}_3}$	-0.1118	0.0542	-0.2069	-0.0132	Region:Year	$\beta_{11,\text{region}_1}$	-0.0079	0.0079	-0.0241	0.0073
	$\beta_{10,\text{age}_2,\text{region}_3}$	-0.0678	0.0263	-0.1189	-0.0229		$\beta_{11,\text{region}_2}$	0.0078	0.0053	-0.0023	0.0176
	$\beta_{10,\text{age}_3,\text{region}_3}$	0.0587	0.0223	0.0121	0.1001		$\beta_{11,\text{region}_3}$	-0.0013	0.0062	-0.0132	0.0106
	$\beta_{10,\text{age}_4,\text{region}_3}$	0.0349	0.0186	-0.0005	0.0730		$\beta_{11,\text{region}_4}$	0.0176	0.0076	0.0036	0.0329
	$\beta_{10,\text{age}_5,\text{region}_3}$	0.0348	0.0174	0.0023	0.0707		$\beta_{11,\text{region}_5}$	0.0197	0.0067	0.0066	0.0321
	$\beta_{10,\text{age}_6,\text{region}_3}$	0.0183	0.0148	-0.0109	0.0463		$\beta_{11,\text{region}_6}$	0.0174	0.0071	0.0030	0.0308
	$\beta_{10,\text{age}_7,\text{region}_3}$	0.0294	0.0156	-0.0006	0.0606		$\beta_{11,\text{region}_7}$	-0.0610	0.0066	-0.0733	-0.0474
	$\beta_{10,\text{age}_8,\text{region}_3}$	0.0128	0.0171	-0.0200	0.0470		$\beta_{11,\text{region}_8}$	0.0038	0.0061	-0.0083	0.0147
	$\beta_{10,\text{age}_9,\text{region}_3}$	-0.0093	0.0163	-0.0403	0.0211		$\beta_{11,\text{region}_9}$	0.0040	0.0068	-0.0100	0.0162
	$\beta_{10,\text{age}_1,\text{region}_4}$	0.0304	0.0565	-0.0721	0.1368	Deprivation:Year	$\beta_{12,\text{deprivation}_1}$	0.0273	0.0059	0.0155	0.0389
	$\beta_{10,\text{age}_2,\text{region}_4}$	0.0727	0.0379	-0.0053	0.1339		$\beta_{12,\text{deprivation}_2}$	0.0317	0.0067	0.0191	0.0448
	$\beta_{10,\text{age}_3,\text{region}_4}$	0.0340	0.0276	-0.0207	0.0859		$\beta_{12,\text{deprivation}_3}$	0.0192	0.0068	0.0059	0.0323
	$\beta_{10,\text{age}_4,\text{region}_4}$	-0.0022	0.0226	-0.0446	0.0414		$\beta_{12,\text{deprivation}_4}$	0.0050	0.0068	-0.0086	0.0184
	$\beta_{10,\text{age}_5,\text{region}_4}$	0.0223	0.0166	-0.0091	0.0565		$\beta_{12,\text{deprivation}_5}$	0.0140	0.0071	-0.0001	0.0268
	$\beta_{10,\text{age}_6,\text{region}_4}$	0.0084	0.0167	-0.0243	0.0418		$\beta_{12,\text{deprivation}_6}$	0.0162	0.0069	0.0029	0.0306
	$\beta_{10,\text{age}_7,\text{region}_4}$	-0.0187	0.0178	-0.0526	0.0154		$\beta_{12,\text{deprivation}_7}$	-0.0138	0.0073	-0.0300	-0.0006
	$\beta_{10,\text{age}_8,\text{region}_4}$	-0.0360	0.0198	-0.0786	-0.0025		$\beta_{12,\text{deprivation}_8}$	-0.0321	0.0086	-0.0499	-0.0158
	$\beta_{10,\text{age}_9,\text{region}_4}$	-0.1108	0.0217	-0.1501	-0.0696		$\beta_{12,\text{deprivation}_9}$	-0.0177	0.0084	-0.0353	-0.0025
	$\beta_{10,\text{age}_1,\text{region}_5}$	0.0541	0.0405	-0.0267	0.1336		$\beta_{12,\text{deprivation}_{10}}$	-0.0499	0.0098	-0.0672	-0.0312
	$\beta_{10,\text{age}_2,\text{region}_5}$	-0.0164	0.0304	-0.0847	0.0350	Age : Year^2	β_{13,age_1}	0.0433	0.0207	-0.0024	0.0823
	$\beta_{10,\text{age}_3,\text{region}_5}$	0.0134	0.0202	-0.0268	0.0495		β_{13,age_2}	0.0021	0.0120	-0.0271	0.0235
	$\beta_{10,\text{age}_4,\text{region}_5}$	-0.0304	0.0187	-0.0629	0.0071		β_{13,age_3}	-0.0297	0.0086	-0.0435	-0.0115
	$\beta_{10,\text{age}_5,\text{region}_5}$	0.0020	0.0195	-0.0318	0.0431		β_{13,age_4}	-0.0172	0.0078	-0.0322	-0.0008
	$\beta_{10,\text{age}_6,\text{region}_5}$	-0.0122	0.0151	-0.0414	0.0172		β_{13,age_5}	-0.0184	0.0079	-0.0330	-0.0047
	$\beta_{10,\text{age}_7,\text{region}_5}$	0.0249	0.0148	-0.0047	0.0527		β_{13,age_6}	0.0459	0.0064	0.0338	0.0583
	$\beta_{10,\text{age}_8,\text{region}_5}$	0.0062	0.0168	-0.0271	0.0373		β_{13,age_7}	0.0143	0.0071	0.0002	0.0279
	$\beta_{10,\text{age}_9,\text{region}_5}$	-0.0415	0.0162	-0.0716	-0.0102		β_{13,age_8}	-0.0296	0.0063	-0.0408	-0.0172
	$\beta_{10,\text{age}_1,\text{region}_6}$	-0.0123	0.0503	-0.1231	0.0703		β_{13,age_9}	-0.0107	0.0058	-0.0233	0.0007
	$\beta_{10,\text{age}_2,\text{region}_6}$	0.0488	0.0349	-0.0106	0.1107		σ^2	0.0017	0.0008	0.0006	0.0031

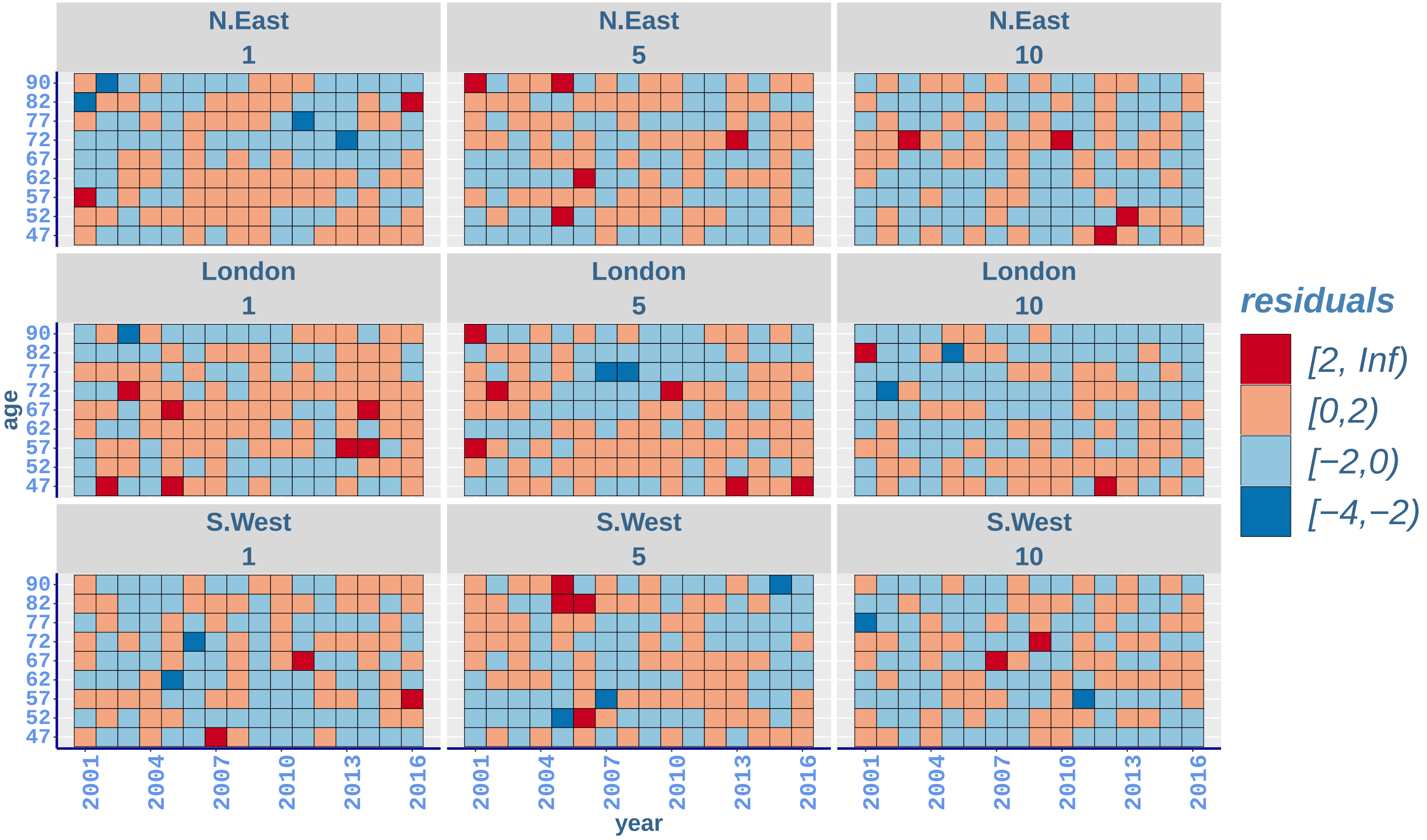

S29 Fig. Heat map of Pearson residuals for trachea, bronchus and lung cancer morbidity for males in North East, London, and South West, deprivation deciles 1, 5, and 10: orange/light blue cells indicate areas with good fit, while red/dark blue cells indicate areas with poor fit. Note that there is a small number of residuals greater than 4, and these are included in the last category.

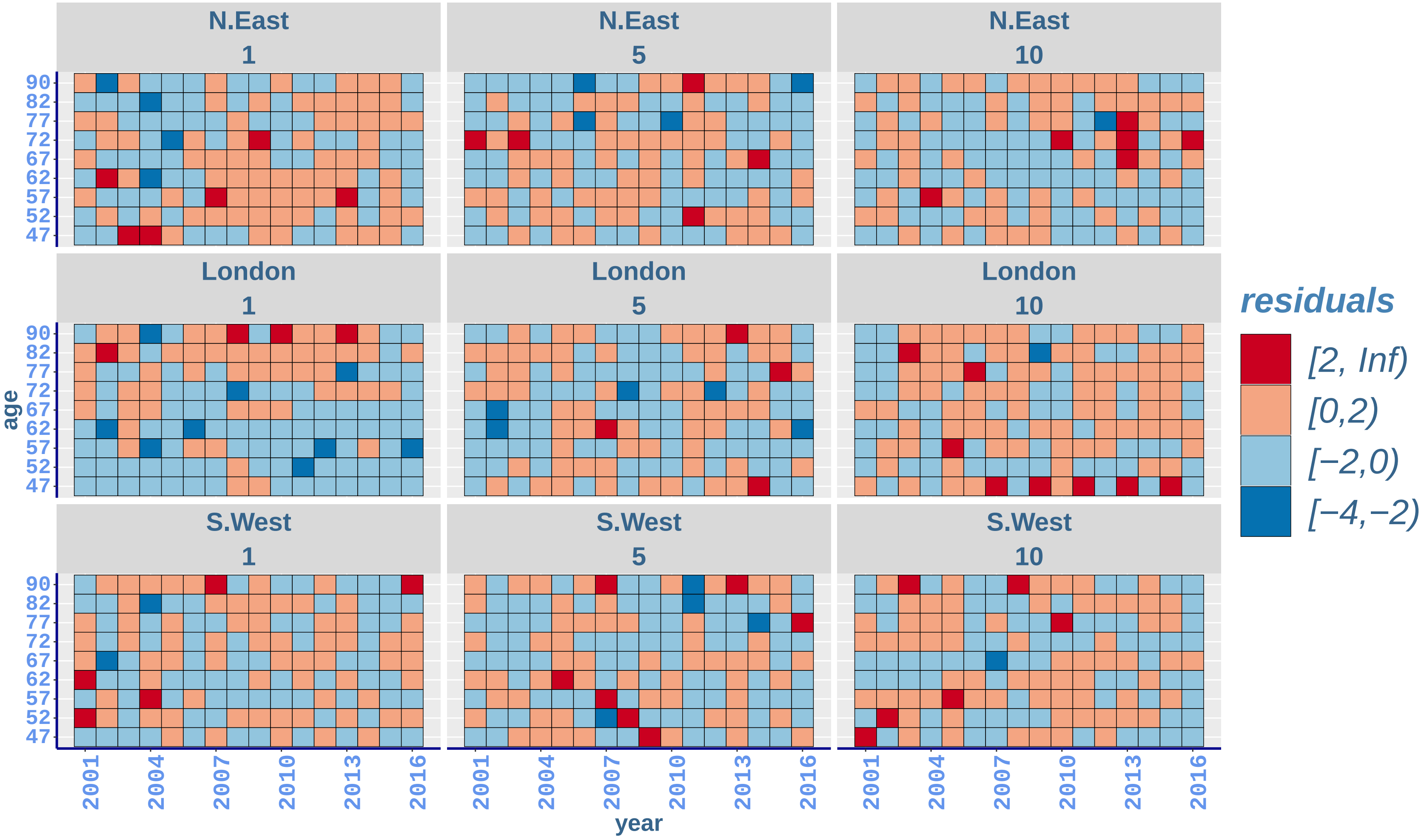

S30 Fig. Heat map of Pearson residuals for trachea, bronchus and lung cancer morbidity for females in North East, London, and South West, deprivation deciles 1, 5, and 10: orange/light blue cells indicate areas with good fit, while red/dark blue cells indicate areas with poor fit. Note that there is a small number of residuals greater than 4, and these are included in the last category.

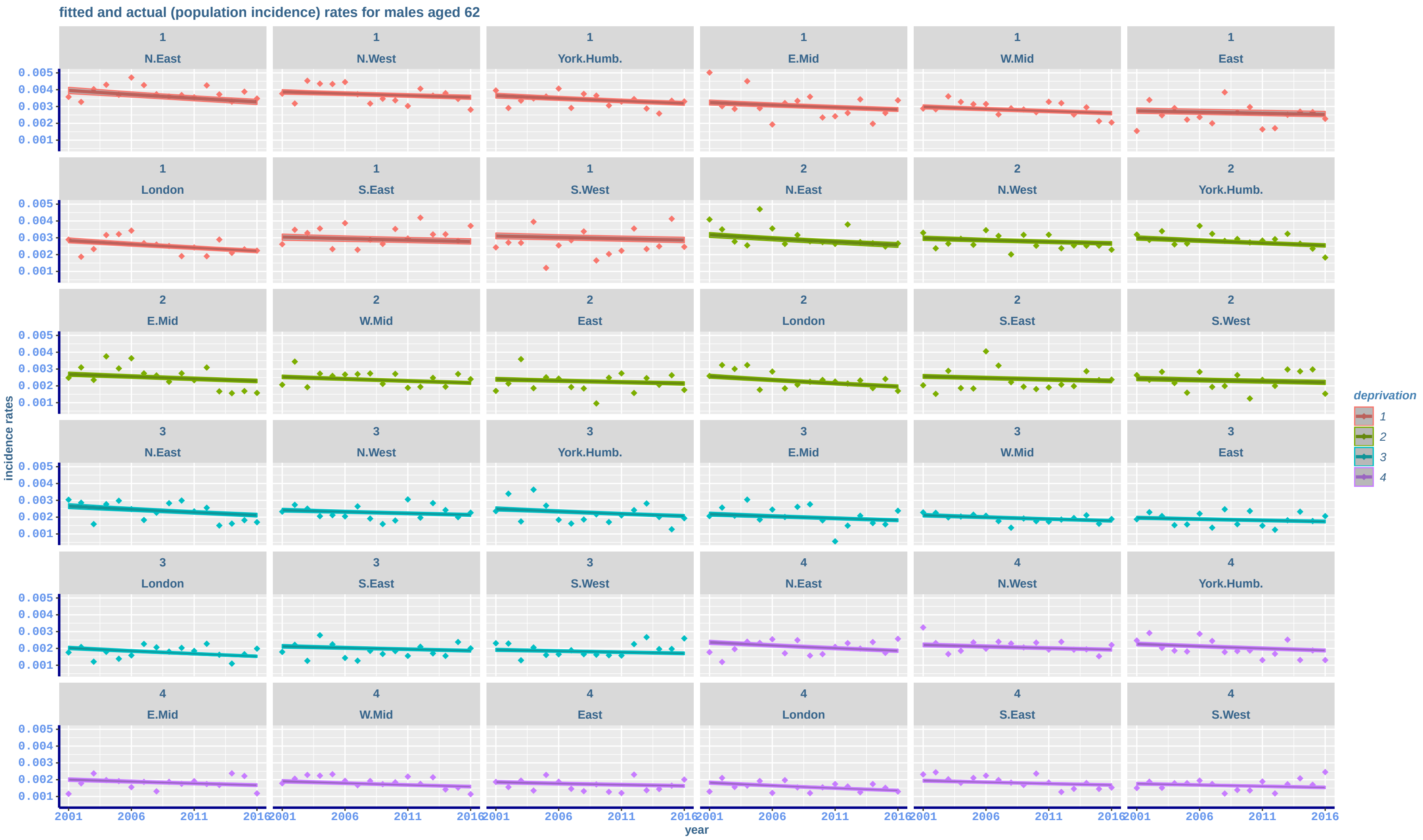

S31 Fig. Trachea, bronchus and lung cancer morbidity for males, age 62, deprivation deciles 1-4 for all regions in England between 2001 and 2016: observed rates (dots), fitted rates (lines), with 95% credible intervals for the fitted rates.

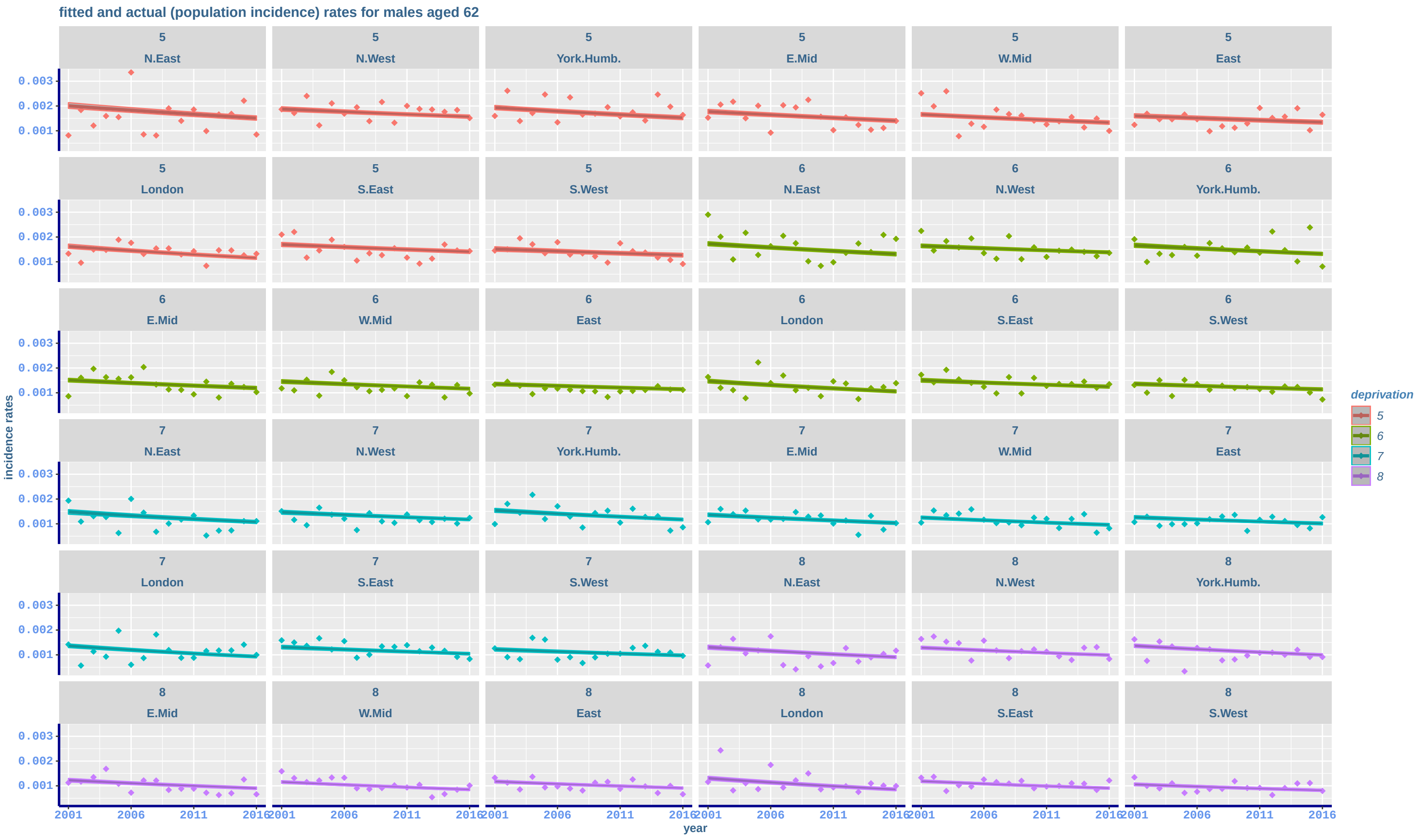

S32 Fig. Trachea, bronchus and lung cancer morbidity for males, age 62, deprivation deciles 5-8 for all regions in England between 2001 and 2016: observed rates (dots), fitted rates (lines), with 95% credible intervals for the fitted rates.

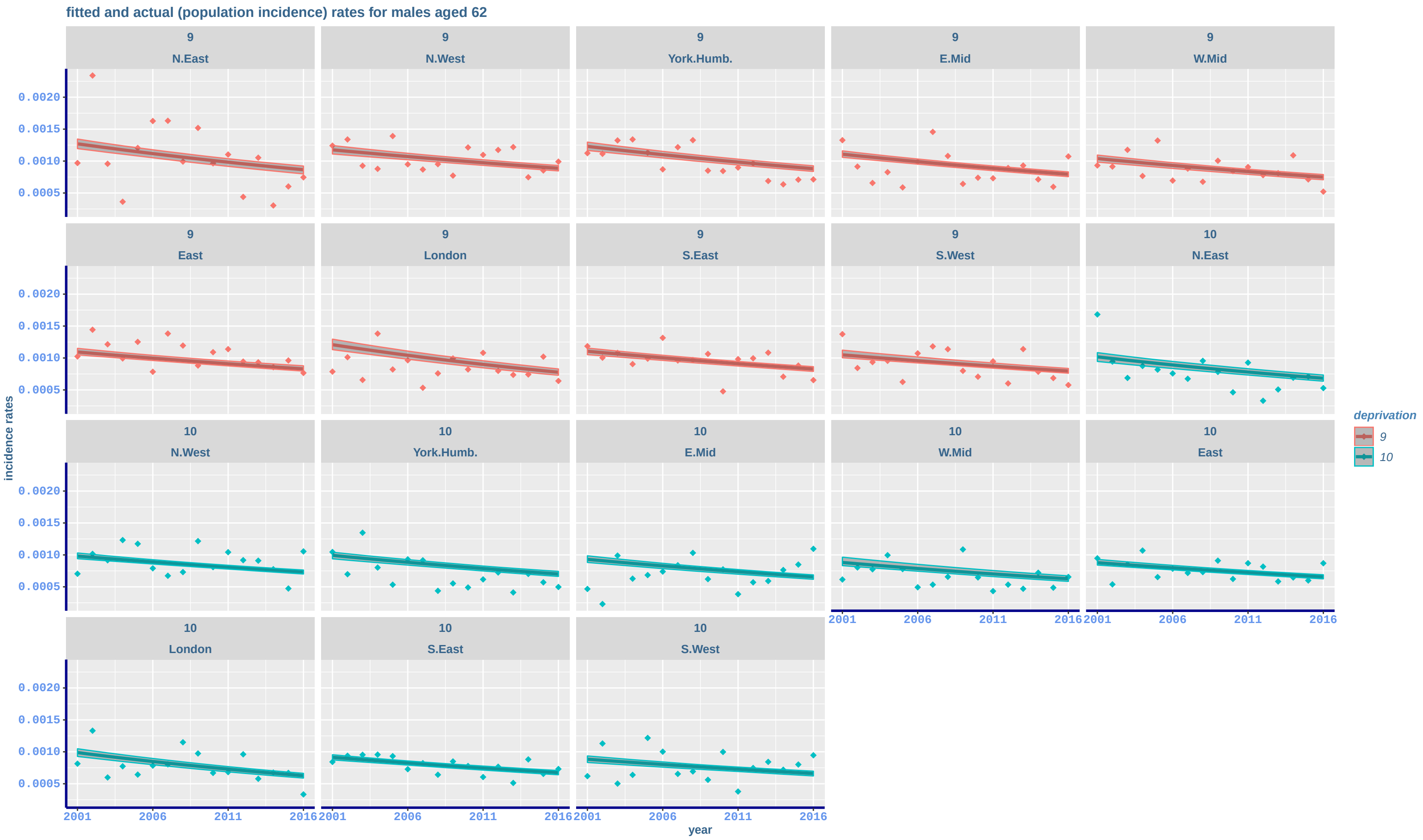

S33 Fig. Trachea, bronchus and lung cancer morbidity for males, age 62, deprivation deciles 9-10 for all regions in England between 2001 and 2016: observed rates (dots), fitted rates (lines), with 95% credible intervals for the fitted rates.

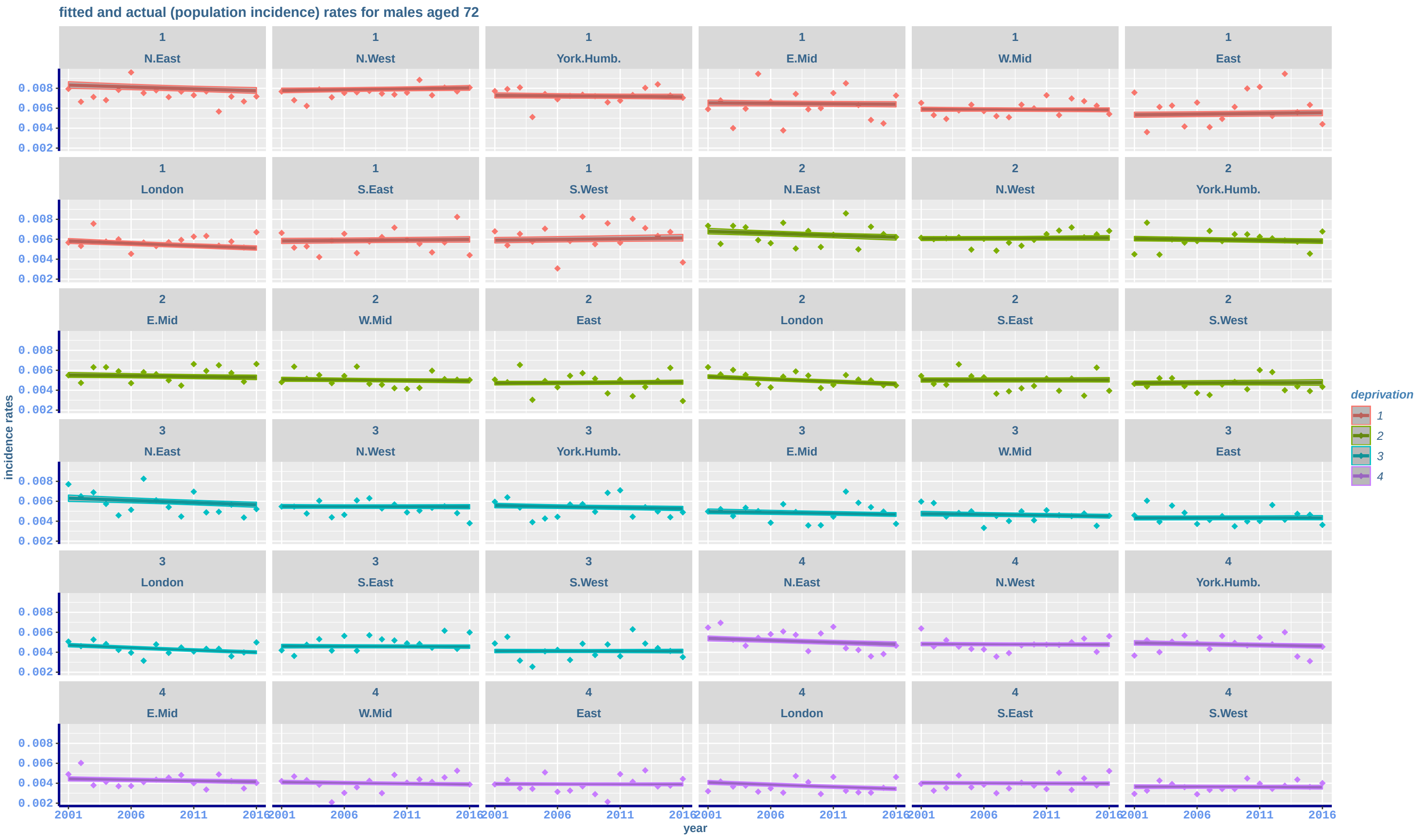

S34 Fig. Trachea, bronchus and lung cancer morbidity for males, age 72, deprivation deciles 1-4 for all regions in England between 2001 and 2016: observed rates (dots), fitted rates (lines), with 95% credible intervals for the fitted rates.

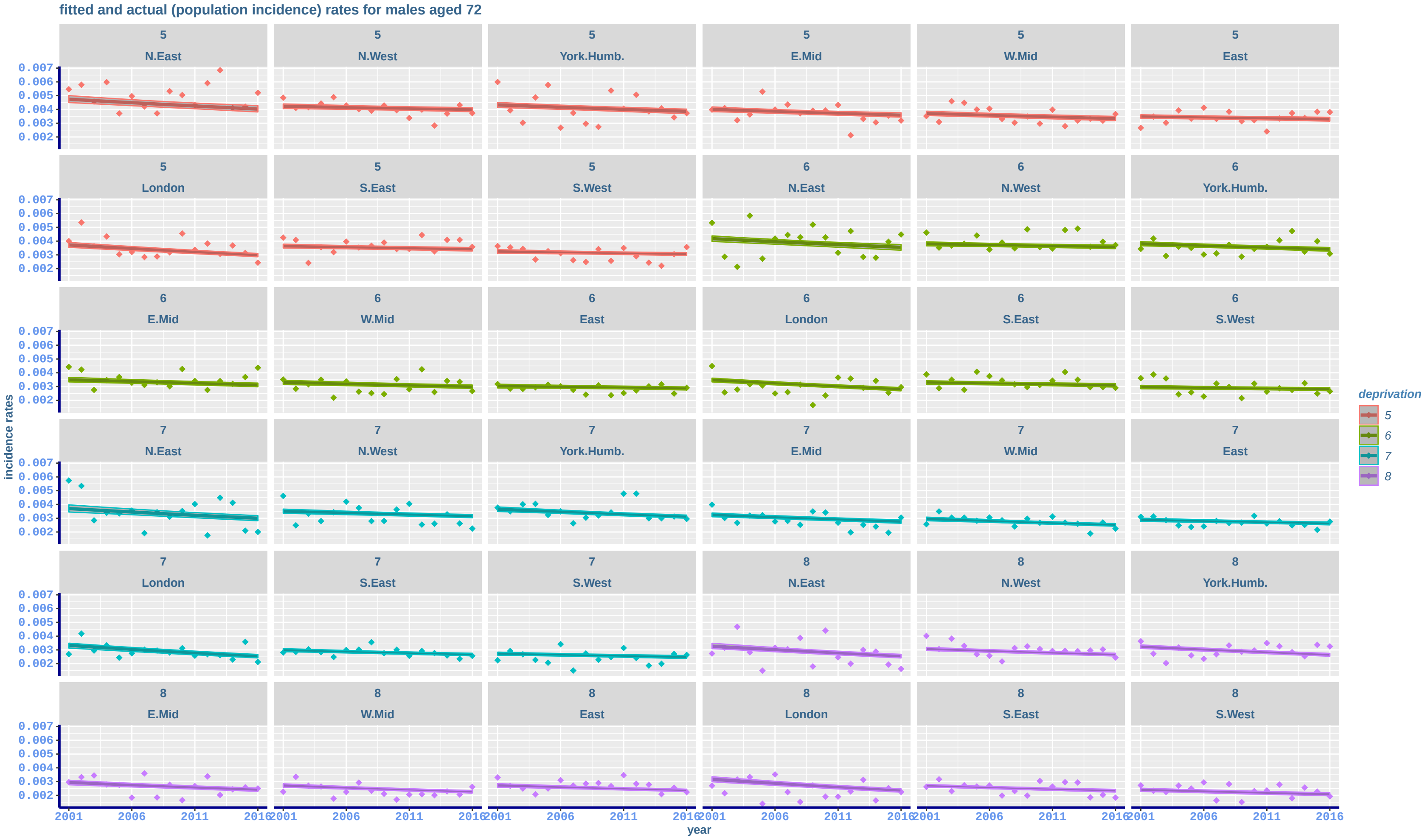

S35 Fig. Trachea, bronchus and lung cancer morbidity for males, age 72, deprivation deciles 5-8 for all regions in England between 2001 and 2016: observed rates (dots), fitted rates (lines), with 95% credible intervals for the fitted rates.

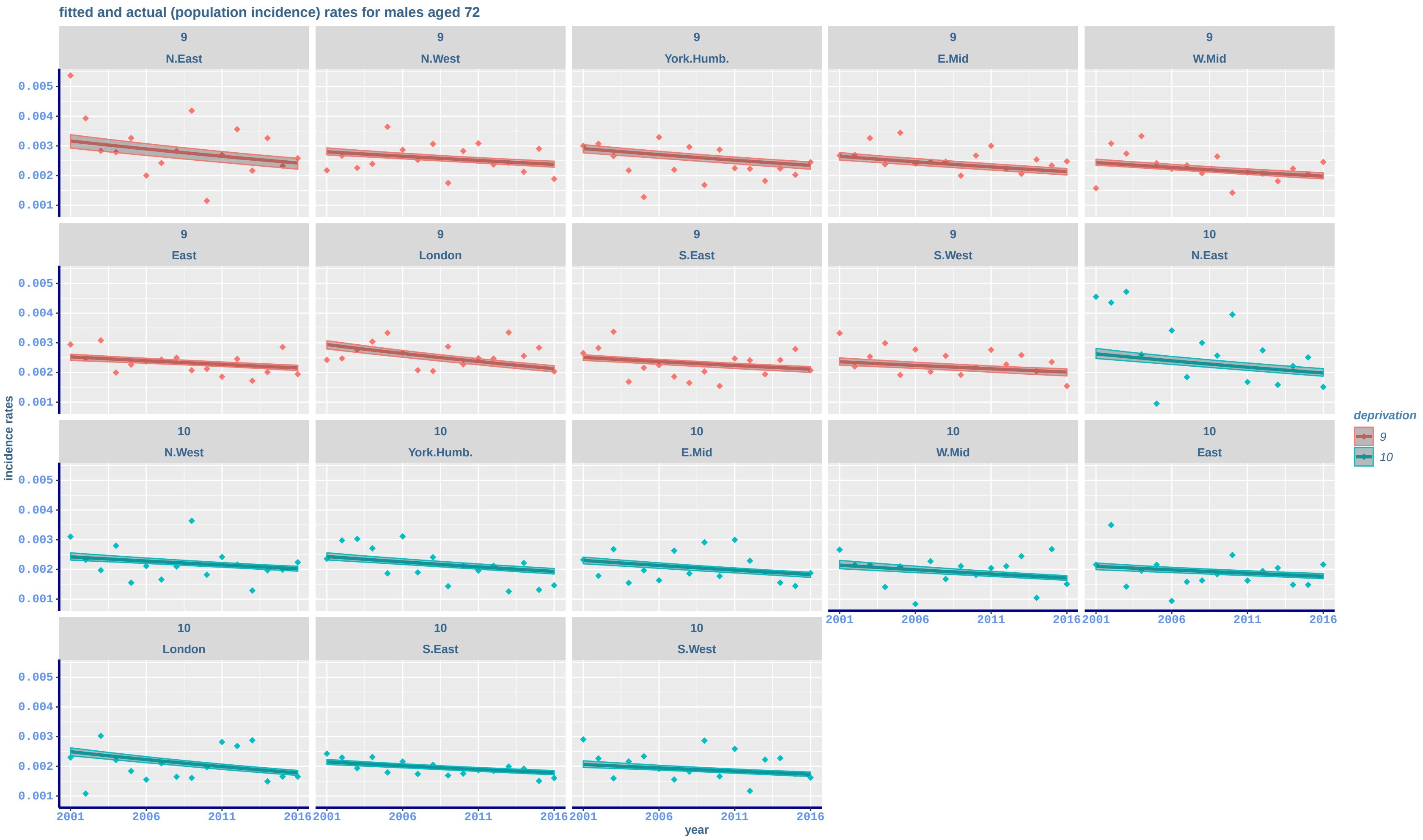

S36 Fig. Trachea, bronchus and lung cancer morbidity for males, age 72, deprivation deciles 9-10 for all regions in England between 2001 and 2016: observed rates (dots), fitted rates (lines), with 95% credible intervals for the fitted rates.

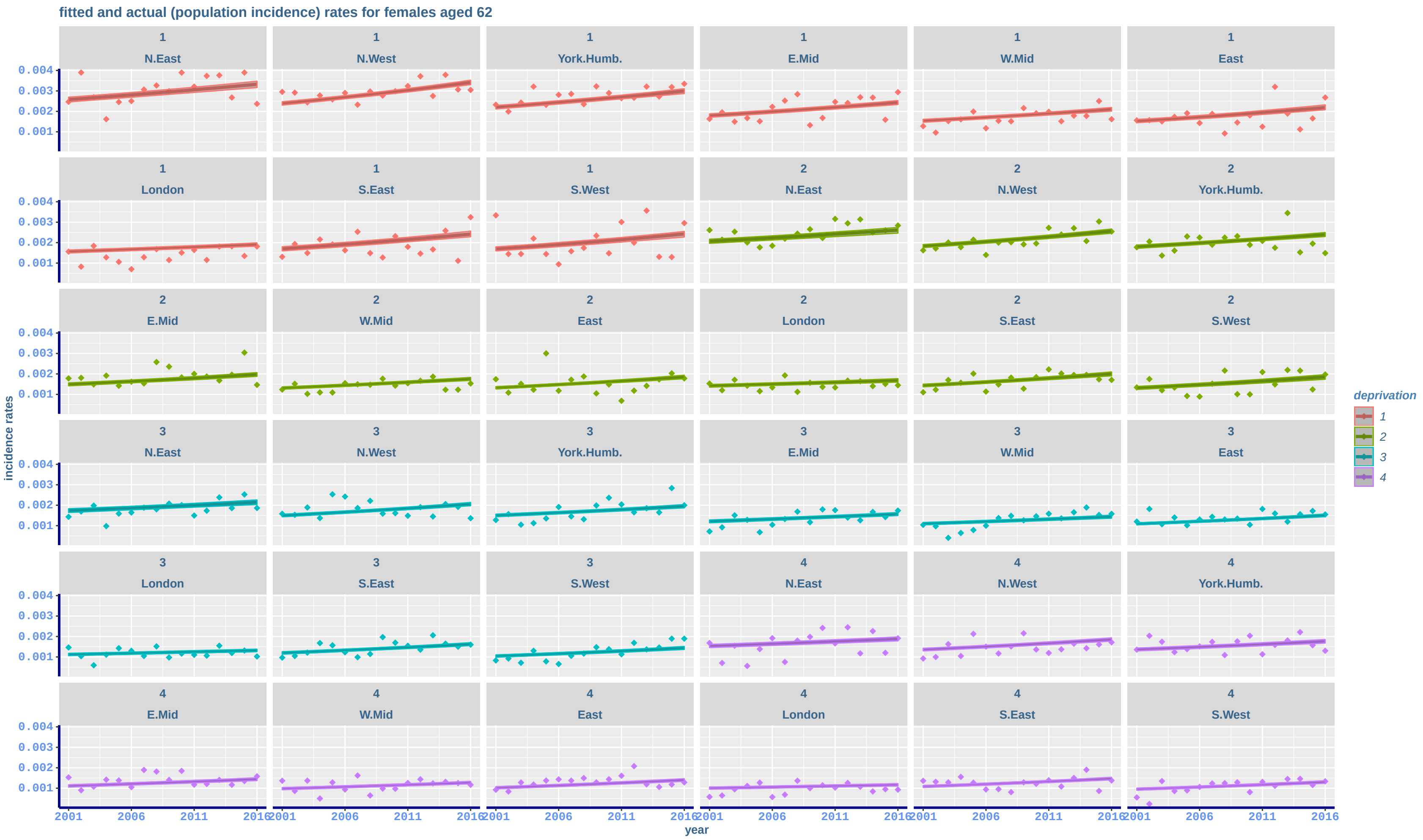

S37 Fig. Trachea, bronchus and lung cancer morbidity for females, age 62, deprivation deciles 1-4 for all regions in England between 2001 and 2016: observed rates (dots), fitted rates (lines), with 95% credible intervals for the fitted rates.

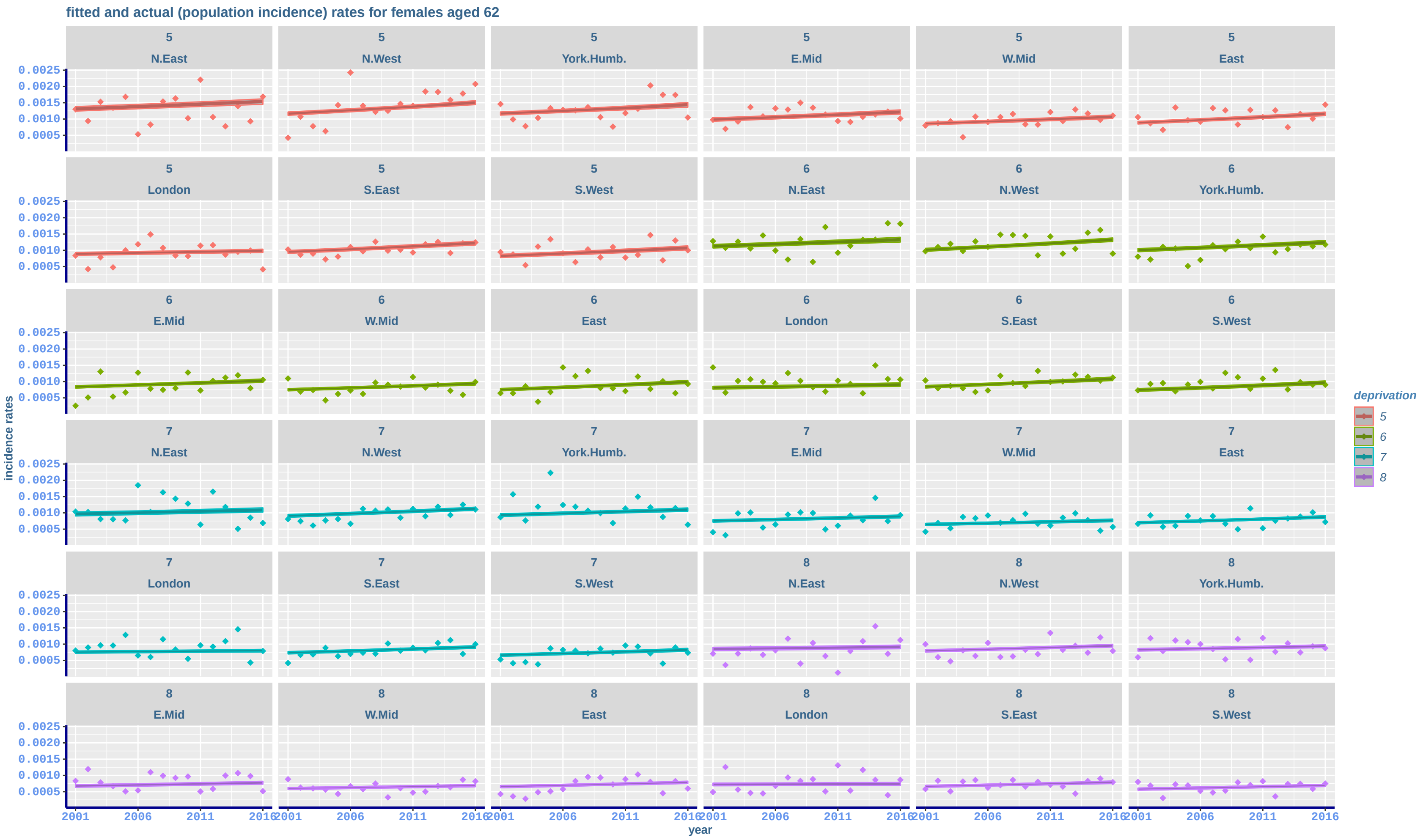

S38 Fig. Trachea, bronchus and lung cancer morbidity for females, age 62, deprivation deciles 5-8 for all regions in England between 2001 and 2016: observed rates (dots), fitted rates (lines), with 95% credible intervals for the fitted rates.

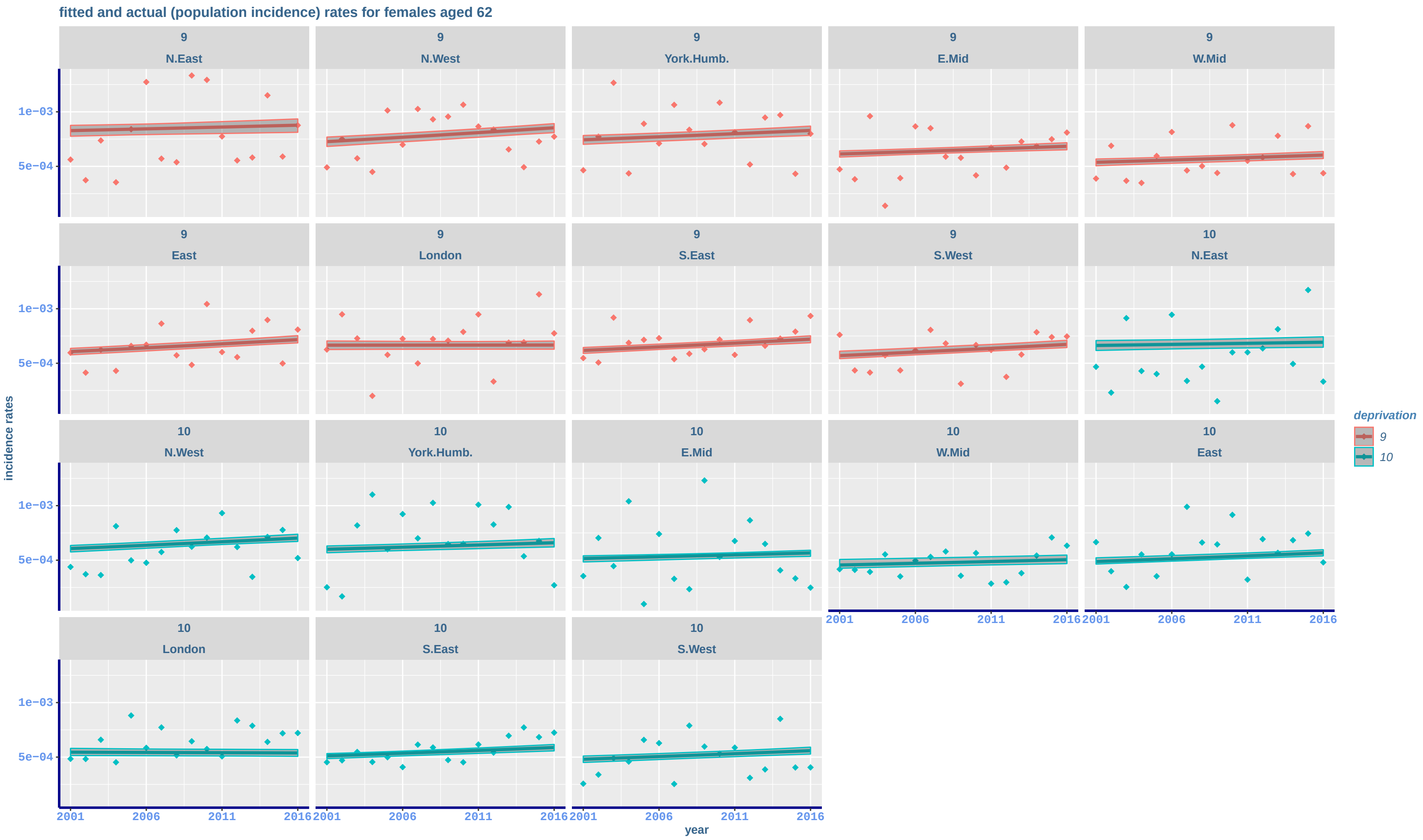

S39 Fig. Trachea, bronchus and lung cancer morbidity for females, age 62, deprivation deciles 9-10 for all regions in England between 2001 and 2016: observed rates (dots), fitted rates (lines), with 95% credible intervals for the fitted rates.

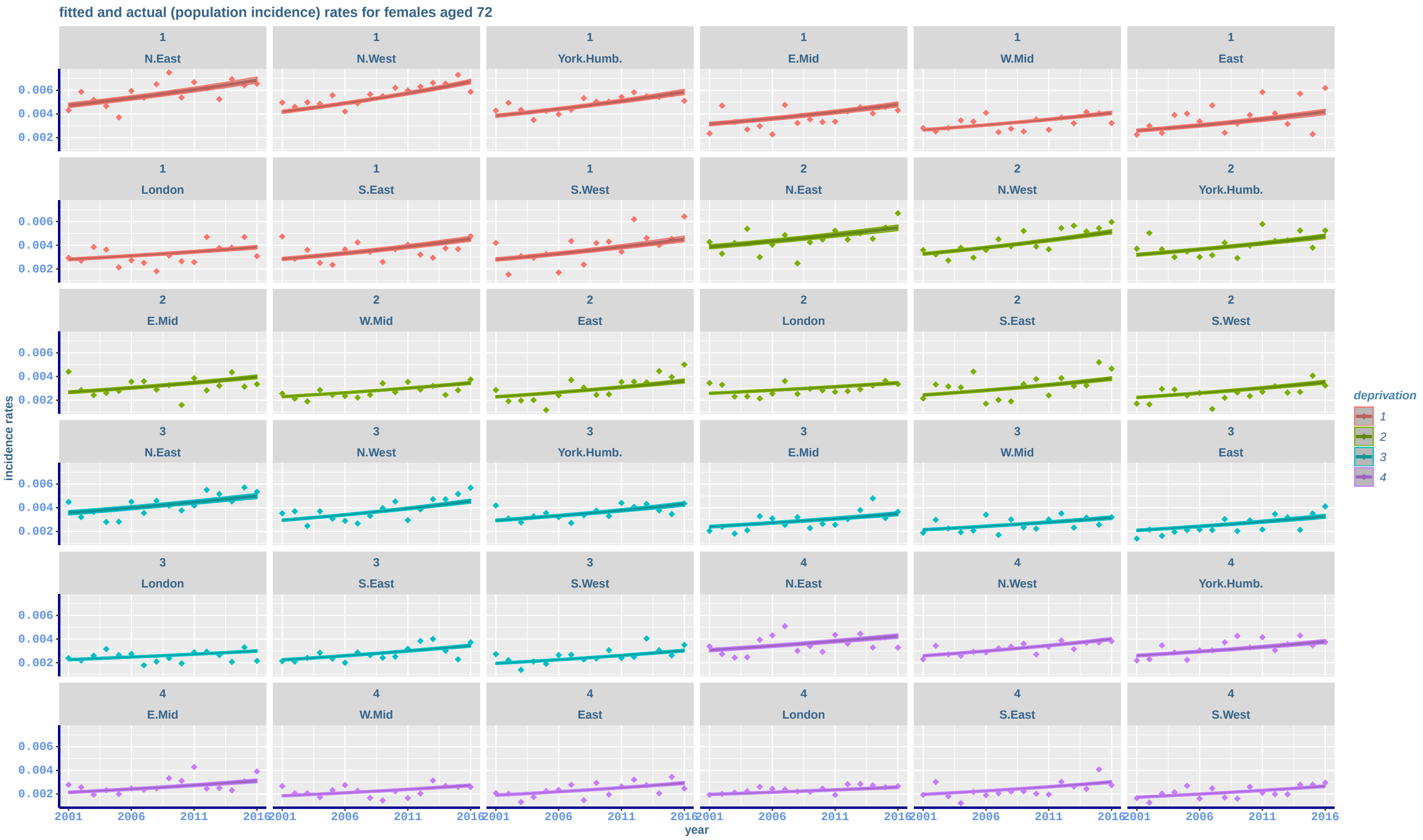

S40 Fig. Trachea, bronchus and lung cancer morbidity for females, age 72, deprivation deciles 1-4 for all regions in England between 2001 and 2016: observed rates (dots), fitted rates (lines), with 95% credible intervals for the fitted rates.

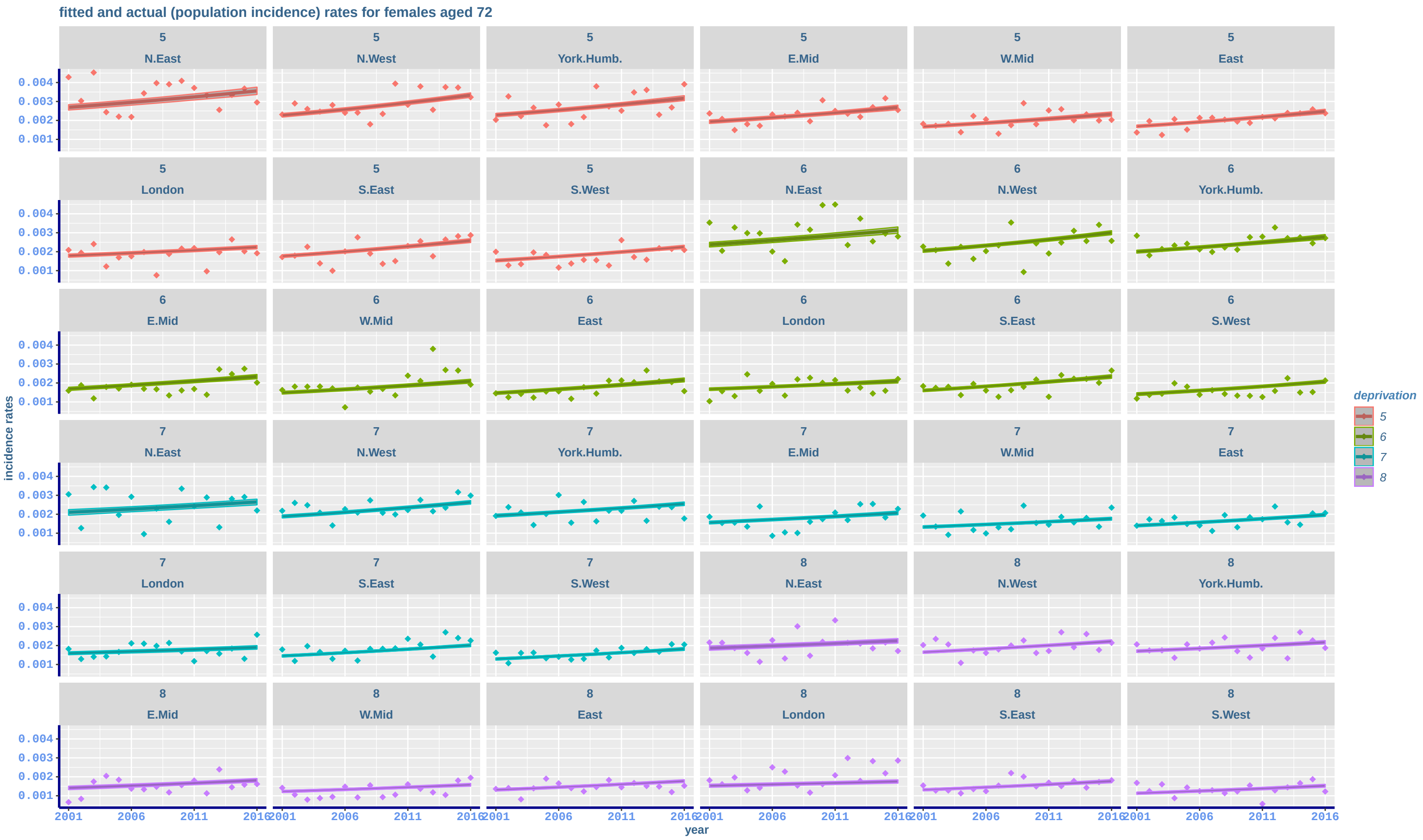

S41 Fig. Trachea, bronchus and lung cancer morbidity for females, age 72, deprivation deciles 5-8 for all regions in England between 2001 and 2016: observed rates (dots), fitted rates (lines), with 95% credible intervals for the fitted rates.

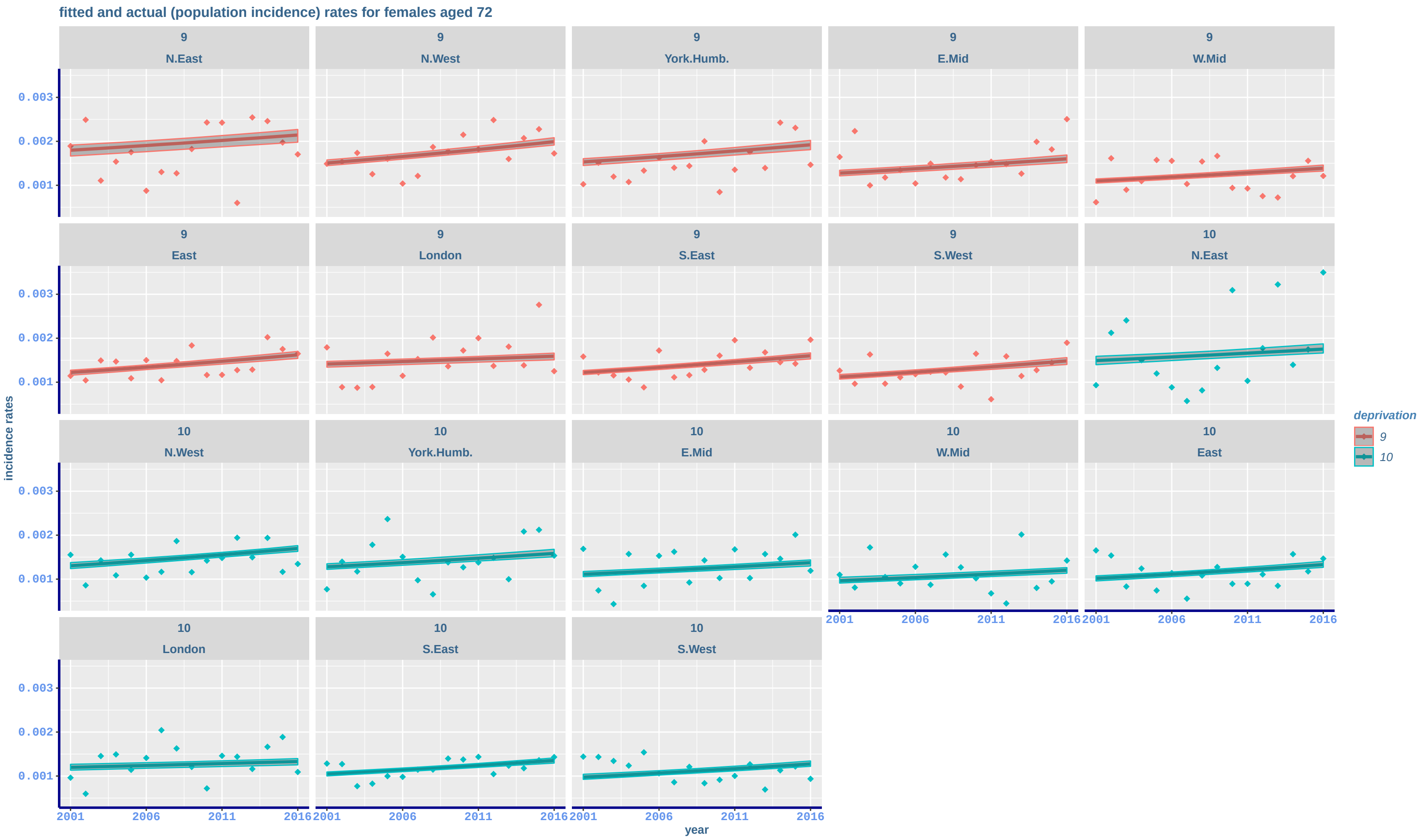

S42 Fig. Trachea, bronchus and lung cancer morbidity for females, age 72, deprivation deciles 9-10 for all regions in England between 2001 and 2016: observed rates (dots), fitted rates (lines), with 95% credible intervals for the fitted rates.

S16 Table. Absolute deprivation differences ($AD_{t,r}$), per 100,000 people, in age-standardised fitted incidence rates of lung, trachea and bronchus cancer from 2001 to 2016 for all regions in England for males; 95% credible intervals in brackets

year	N.East	N.West	York.Humb.	E.Mid	W.Mid	East	London	S.East	S.West
2001	310 (297, 323)	293 (282, 303)	269 (256, 282)	230 (215, 244)	207 (198, 217)	178 (163, 193)	177 (163, 191)	203 (189, 217)	218 (205, 233)
2002	310 (297, 322)	295 (285, 304)	270 (258, 282)	231 (217, 244)	208 (199, 218)	180 (165, 194)	177 (164, 190)	205 (191, 218)	220 (206, 235)
2003	310 (297, 321)	297 (288, 306)	271 (259, 283)	232 (218, 245)	209 (201, 219)	182 (167, 196)	178 (165, 190)	206 (192, 219)	222 (208, 236)
2004	310 (298, 321)	298 (290, 307)	272 (261, 283)	233 (219, 246)	210 (202, 220)	183 (169, 198)	178 (165, 189)	208 (194, 221)	223 (209, 237)
2005	310 (298, 321)	300 (293, 309)	273 (262, 284)	234 (221, 246)	211 (204, 220)	185 (171, 200)	178 (166, 189)	209 (195, 222)	225 (211, 239)
2006	310 (298, 320)	302 (295, 310)	274 (264, 285)	235 (222, 247)	212 (205, 221)	187 (173, 201)	178 (166, 188)	211 (197, 223)	226 (212, 241)
2007	310 (298, 320)	304 (297, 311)	275 (265, 285)	236 (223, 248)	213 (206, 222)	188 (175, 203)	178 (167, 188)	212 (198, 225)	228 (214, 243)
2008	310 (298, 320)	306 (299, 313)	276 (266, 286)	237 (224, 248)	214 (207, 223)	190 (177, 205)	178 (167, 188)	214 (200, 226)	229 (215, 245)
2009	310 (298, 320)	308 (301, 315)	277 (267, 287)	238 (225, 249)	215 (208, 224)	192 (179, 207)	178 (168, 188)	215 (201, 228)	231 (216, 247)
2010	310 (298, 320)	310 (303, 317)	278 (268, 288)	239 (226, 250)	216 (209, 225)	193 (180, 209)	177 (168, 188)	217 (202, 230)	233 (218, 249)
2011	310 (298, 320)	312 (305, 320)	279 (269, 289)	240 (227, 251)	217 (209, 227)	195 (182, 211)	177 (168, 187)	219 (204, 232)	234 (219, 251)
2012	310 (298, 321)	314 (306, 322)	280 (270, 290)	240 (229, 251)	218 (210, 228)	197 (183, 213)	177 (168, 187)	220 (205, 234)	236 (221, 254)
2013	309 (297, 321)	316 (308, 324)	281 (271, 291)	241 (230, 252)	219 (210, 229)	198 (185, 214)	177 (168, 187)	222 (206, 236)	238 (222, 256)
2014	309 (297, 321)	318 (309, 327)	282 (272, 292)	242 (231, 253)	220 (211, 231)	200 (186, 216)	177 (168, 187)	223 (207, 238)	239 (223, 258)
2015	309 (297, 322)	320 (311, 329)	283 (272, 294)	243 (232, 254)	221 (211, 232)	202 (188, 219)	177 (168, 187)	225 (208, 239)	241 (224, 260)
2016	309 (297, 323)	322 (312, 332)	284 (273, 295)	244 (232, 255)	222 (212, 234)	204 (190, 221)	177 (167, 187)	226 (210, 241)	243 (226, 263)

S17 Table. Relative deprivation differences ($RD_{t,r}$) in age-standardised fitted incidence rates of lung, trachea and bronchus cancer from 2001 to 2016 for all regions in England for males; 95% credible intervals in brackets

year	N.East	N.West	York.Humb.	E.Mid	W.Mid	East	London	S.East	S.West
2001	1.7539	1.8482	1.6413	1.527	1.4398	1.2681	1.0356	1.407	1.5347
	(1.6017, 1.8829)	(1.7435, 1.9557)	(1.5069, 1.7943)	(1.3992, 1.6497)	(1.3514, 1.5481)	(1.135, 1.3998)	(0.9274, 1.1335)	(1.3016, 1.5185)	(1.4021, 1.6733)
2002	1.7864	1.8819	1.6725	1.5568	1.4686	1.2948	1.0594	1.4351	1.5642
	(1.6366, 1.9116)	(1.7804, 1.9871)	(1.5381, 1.8219)	(1.4325, 1.6769)	(1.3801, 1.5762)	(1.1619, 1.4241)	(0.9534, 1.1559)	(1.3292, 1.546)	(1.4326, 1.6996)
2003	1.8194	1.9159	1.704	1.5869	1.4977	1.3217	1.0834	1.4636	1.5941
	(1.6699, 1.9411)	(1.8196, 2.0196)	(1.5685, 1.8487)	(1.465, 1.7063)	(1.4096, 1.6065)	(1.1884, 1.4504)	(0.981, 1.1792)	(1.3547, 1.5735)	(1.4613, 1.7261)
2004	1.8527	1.9503	1.7359	1.6174	1.5272	1.349	1.1076	1.4924	1.6243
	(1.7053, 1.9704)	(1.8577, 2.0512)	(1.6001, 1.8767)	(1.4972, 1.7351)	(1.4363, 1.6364)	(1.2153, 1.4763)	(1.0082, 1.2033)	(1.3838, 1.5999)	(1.4899, 1.7562)
2005	1.8863	1.9851	1.7682	1.6482	1.557	1.3766	1.1322	1.5215	1.6549
	(1.7382, 2.0005)	(1.8973, 2.0832)	(1.6326, 1.9064)	(1.5287, 1.7646)	(1.4646, 1.6641)	(1.2407, 1.5069)	(1.0359, 1.2281)	(1.4098, 1.6274)	(1.5188, 1.7884)
2006	1.9204	2.0203	1.8008	1.6793	1.5872	1.4045	1.1571	1.551	1.6858
	(1.7711, 2.0324)	(1.9357, 2.1174)	(1.6639, 1.9401)	(1.559, 1.7945)	(1.4922, 1.6957)	(1.2692, 1.5348)	(1.0613, 1.2532)	(1.4347, 1.6585)	(1.5465, 1.8184)
2007	1.9549	2.0559	1.8338	1.7109	1.6177	1.4327	1.1822	1.5808	1.717
	(1.8063, 2.0654)	(1.9727, 2.1492)	(1.6961, 1.9755)	(1.5917, 1.8256)	(1.5188, 1.7285)	(1.2981, 1.5671)	(1.0859, 1.277)	(1.4615, 1.6859)	(1.5769, 1.8507)
2008	1.9897	2.0919	1.8672	1.7427	1.6485	1.4612	1.2076	1.6109	1.7486
	(1.842, 2.1011)	(2.0066, 2.1825)	(1.7315, 2.0099)	(1.625, 1.8574)	(1.5468, 1.7626)	(1.3272, 1.5995)	(1.1093, 1.3001)	(1.4871, 1.7156)	(1.6059, 1.8847)
2009	2.025	2.1283	1.9009	1.775	1.6797	1.4901	1.2333	1.6414	1.7806
	(1.8732, 2.138)	(2.0409, 2.2199)	(1.7624, 2.0465)	(1.6579, 1.8906)	(1.5743, 1.7996)	(1.3568, 1.6302)	(1.1346, 1.3249)	(1.5121, 1.7483)	(1.633, 1.918)
2010	2.0606	2.1652	1.9351	1.8076	1.7113	1.5193	1.2593	1.6722	1.8129
	(1.9057, 2.1762)	(2.074, 2.2586)	(1.7959, 2.0863)	(1.6907, 1.9224)	(1.6013, 1.8366)	(1.3868, 1.6648)	(1.1592, 1.3505)	(1.5384, 1.7793)	(1.6623, 1.9555)
2011	2.0967	2.2024	1.9696	1.8406	1.7432	1.5488	1.2856	1.7034	1.8456
	(1.939, 2.2144)	(2.1064, 2.2997)	(1.8263, 2.1241)	(1.7236, 1.9549)	(1.6292, 1.8753)	(1.4171, 1.6941)	(1.1848, 1.3756)	(1.5636, 1.8147)	(1.6909, 1.9929)
2012	2.1332	2.2401	2.0046	1.8739	1.7755	1.5786	1.3122	1.7349	1.8787
	(1.9706, 2.2546)	(2.1398, 2.3426)	(1.8564, 2.1657)	(1.7551, 1.9896)	(1.6572, 1.9142)	(1.4454, 1.7264)	(1.2079, 1.4023)	(1.5874, 1.8493)	(1.7197, 2.0323)
2013	2.1701	2.2782	2.0399	1.9077	1.8081	1.6088	1.3391	1.7668	1.9121
	(2.0018, 2.2962)	(2.173, 2.3884)	(1.8864, 2.2088)	(1.788, 2.0238)	(1.6859, 1.9518)	(1.4745, 1.7626)	(1.231, 1.4298)	(1.6114, 1.8854)	(1.7472, 2.0727)
2014	2.2074	2.3168	2.0756	1.9418	1.8412	1.6394	1.3663	1.799	1.946
	(2.0333, 2.3388)	(2.2056, 2.4353)	(1.9171, 2.2523)	(1.82, 2.059)	(1.7141, 1.9898)	(1.5034, 1.7962)	(1.2532, 1.4571)	(1.6363, 1.9223)	(1.7759, 2.1144)
2015	2.2451	2.3557	2.1118	1.9763	1.8746	1.6703	1.3938	1.8316	1.9802
	(2.0639, 2.3845)	(2.2373, 2.4843)	(1.95, 2.2986)	(1.8496, 2.0973)	(1.7419, 2.0317)	(1.5273, 1.8297)	(1.2766, 1.4868)	(1.6617, 1.9622)	(1.8043, 2.1568)
2016	2.2833	2.3952	2.1483	2.0112	1.9084	1.7015	1.4217	1.8646	2.0148
	(2.0971, 2.4293)	(2.2676, 2.5341)	(1.9806, 2.344)	(1.8813, 2.1361)	(1.7698, 2.0759)	(1.5537, 1.8646)	(1.301, 1.5176)	(1.6884, 2.0029)	(1.8319, 2.1975)

S18 Table. Absolute deprivation differences ($AD_{t,r}$), per 100,000 people, in age-standardised fitted incidence rates of lung, trachea and bronchus cancer from 2001 to 2016 for all regions in England for females; 95% credible intervals in brackets

year	N.East	N.West	York.Humb.	E.Mid	W.Mid	East	London	S.East	S.West
2001	180 (172, 188)	162 (156, 167)	146 (139, 153)	115 (108, 121)	96 (92, 101)	90 (82, 97)	89 (83, 95)	103 (96, 110)	108 (100, 115)
2002	185 (177, 192)	167 (162, 172)	150 (144, 157)	119 (111, 125)	100 (95, 104)	93 (86, 100)	91 (85, 98)	107 (100, 114)	112 (104, 119)
2003	190 (182, 198)	173 (168, 178)	155 (149, 162)	122 (115, 128)	103 (99, 107)	97 (89, 104)	94 (88, 100)	111 (104, 118)	115 (108, 123)
2004	195 (188, 203)	179 (174, 184)	160 (154, 167)	126 (119, 132)	106 (102, 111)	100 (93, 108)	96 (91, 102)	114 (107, 122)	120 (111, 127)
2005	201 (193, 208)	185 (180, 190)	165 (159, 172)	130 (123, 137)	110 (106, 114)	104 (97, 112)	99 (94, 105)	119 (111, 126)	124 (115, 132)
2006	207 (199, 214)	192 (187, 197)	171 (164, 177)	135 (127, 141)	113 (109, 118)	108 (100, 116)	102 (96, 107)	123 (115, 131)	128 (119, 137)
2007	212 (204, 220)	198 (194, 203)	176 (170, 182)	139 (131, 145)	117 (113, 122)	112 (104, 121)	105 (99, 110)	127 (119, 135)	133 (123, 141)
2008	218 (210, 226)	205 (200, 210)	182 (175, 188)	143 (135, 150)	121 (117, 126)	116 (109, 125)	108 (102, 113)	132 (124, 140)	137 (128, 146)
2009	224 (216, 232)	212 (207, 218)	187 (181, 194)	148 (140, 154)	125 (120, 130)	120 (112, 130)	110 (105, 116)	136 (128, 145)	142 (132, 151)
2010	231 (222, 238)	220 (214, 225)	193 (187, 200)	153 (145, 159)	129 (124, 134)	125 (117, 135)	113 (108, 119)	141 (132, 150)	147 (137, 157)
2011	237 (228, 245)	227 (222, 233)	199 (193, 207)	158 (149, 165)	133 (128, 138)	129 (121, 140)	117 (111, 123)	146 (137, 155)	152 (141, 162)
2012	244 (235, 252)	235 (229, 242)	206 (199, 213)	163 (154, 170)	137 (132, 143)	134 (125, 145)	120 (114, 126)	151 (141, 160)	157 (146, 168)
2013	250 (241, 259)	243 (237, 250)	212 (205, 220)	168 (159, 175)	142 (136, 148)	139 (130, 150)	123 (117, 130)	156 (146, 166)	163 (151, 174)
2014	257 (248, 266)	252 (245, 259)	219 (212, 228)	173 (164, 181)	146 (140, 153)	144 (135, 156)	126 (121, 133)	162 (151, 172)	169 (156, 180)
2015	265 (254, 274)	261 (253, 269)	226 (218, 235)	179 (170, 187)	151 (145, 158)	150 (139, 162)	130 (124, 137)	168 (156, 178)	175 (162, 187)
2016	272 (261, 282)	270 (261, 279)	233 (225, 243)	185 (175, 193)	156 (149, 163)	155 (144, 168)	133 (127, 141)	173 (161, 185)	181 (167, 194)

S19 Table. Relative deprivation differences ($\mathrm{RD}_{t,r}$) in age-standardised fitted incidence rates of lung, trachea and bronchus cancer from 2001 to 2016 for all regions in England for females; 95% credible intervals in brackets

year	N.East	N.West	York.Humb.	E.Mid	W.Mid	East	London	S.East	S.West
2001	1.8757 (1.7226, 2.0112)	1.9753 (1.8689, 2.0868)	1.7591 (1.6199, 1.9203)	1.6402 (1.5056, 1.7686)	1.5491 (1.458, 1.6651)	1.3714 (1.2293, 1.5088)	1.1299 (1.0172, 1.231)	1.5183 (1.4063, 1.6349)	1.654 (1.5178, 1.7953)
2002	1.9097 (1.7571, 2.0422)	2.0105 (1.9082, 2.1189)	1.7918 (1.6558, 1.948)	1.6713 (1.5408, 1.7967)	1.5793 (1.4897, 1.6943)	1.3994 (1.258, 1.5355)	1.1548 (1.0457, 1.2544)	1.5478 (1.4333, 1.6623)	1.685 (1.5507, 1.823)
2003	1.9442 (1.7935, 2.0726)	2.0461 (1.9488, 2.1516)	1.8248 (1.6881, 1.9777)	1.7029 (1.574, 1.8277)	1.6098 (1.5192, 1.7273)	1.4276 (1.2856, 1.5622)	1.18 (1.0736, 1.2788)	1.5777 (1.4602, 1.6913)	1.7163 (1.5808, 1.8533)
2004	1.979 (1.8293, 2.1042)	2.0822 (1.9894, 2.1862)	1.8582 (1.7201, 2.0069)	1.7348 (1.6068, 1.858)	1.6407 (1.548, 1.7559)	1.4562 (1.3131, 1.5894)	1.2054 (1.102, 1.3037)	1.6079 (1.4904, 1.721)	1.748 (1.611, 1.8871)
2005	2.0142 (1.8624, 2.1346)	2.1186 (2.0303, 2.2184)	1.892 (1.7545, 2.0394)	1.7671 (1.6407, 1.8876)	1.6719 (1.5774, 1.786)	1.4851 (1.3405, 1.62)	1.2311 (1.1311, 1.3305)	1.6385 (1.5184, 1.7505)	1.7801 (1.6401, 1.9189)
2006	2.0499 (1.8984, 2.168)	2.1555 (2.0709, 2.2532)	1.9262 (1.7876, 2.0734)	1.7997 (1.6742, 1.9189)	1.7035 (1.6061, 1.8186)	1.5143 (1.3698, 1.6514)	1.2572 (1.1586, 1.3564)	1.6694 (1.5455, 1.7799)	1.8125 (1.6707, 1.9524)
2007	2.0859 (1.9343, 2.2026)	2.1927 (2.108, 2.2885)	1.9607 (1.8227, 2.1096)	1.8327 (1.7079, 1.9516)	1.7355 (1.6347, 1.8543)	1.5439 (1.4003, 1.6846)	1.2835 (1.1857, 1.3821)	1.7006 (1.5729, 1.8099)	1.8453 (1.7002, 1.9854)
2008	2.1224 (1.9682, 2.2392)	2.2305 (2.1449, 2.3228)	1.9957 (1.8576, 2.1449)	1.8661 (1.7428, 1.9854)	1.7678 (1.6628, 1.8913)	1.5738 (1.4296, 1.7175)	1.3101 (1.21, 1.4056)	1.7322 (1.5993, 1.8413)	1.8785 (1.7306, 2.0199)
2009	2.1593 (2.0019, 2.2765)	2.2686 (2.1817, 2.3619)	2.031 (1.8905, 2.1836)	1.8999 (1.7765, 2.0181)	1.8005 (1.6911, 1.9292)	1.604 (1.4597, 1.753)	1.337 (1.2351, 1.4315)	1.7642 (1.6262, 1.8749)	1.912 (1.7608, 2.0573)
2010	2.1966 (2.0351, 2.316)	2.3072 (2.2155, 2.403)	2.0668 (1.9238, 2.224)	1.9341 (1.8098, 2.0526)	1.8335 (1.721, 1.969)	1.6346 (1.492, 1.7892)	1.3643 (1.2607, 1.4589)	1.7965 (1.6528, 1.9081)	1.946 (1.7905, 2.0987)
2011	2.2344 (2.0696, 2.3558)	2.3462 (2.2509, 2.4469)	2.103 (1.9549, 2.2638)	1.9687 (1.8437, 2.0866)	1.867 (1.75, 2.0094)	1.6655 (1.5242, 1.8214)	1.3918 (1.2873, 1.4865)	1.8292 (1.6797, 1.945)	1.9803 (1.8211, 2.139)
2012	2.2726 (2.103, 2.3983)	2.3857 (2.2851, 2.4912)	2.1396 (1.9881, 2.3083)	2.0036 (1.8778, 2.1228)	1.9008 (1.7796, 2.0492)	1.6968 (1.5529, 1.8538)	1.4197 (1.3135, 1.5145)	1.8623 (1.7055, 1.9834)	2.015 (1.8516, 2.18)
2013	2.3111 (2.1354, 2.4417)	2.4256 (2.3189, 2.5387)	2.1766 (2.0191, 2.3545)	2.039 (1.9128, 2.159)	1.935 (1.8085, 2.0899)	1.7285 (1.5851, 1.8925)	1.4479 (1.3365, 1.5432)	1.8957 (1.7322, 2.0221)	2.05 (1.8808, 2.2216)
2014	2.3502 (2.1684, 2.4859)	2.466 (2.3553, 2.5887)	2.214 (2.0515, 2.4028)	2.0747 (1.9474, 2.1959)	1.9696 (1.8387, 2.1322)	1.7605 (1.6175, 1.9271)	1.4763 (1.3609, 1.5728)	1.9295 (1.7555, 2.0602)	2.0855 (1.909, 2.2641)
2015	2.3897 (2.2016, 2.5324)	2.5068 (2.3866, 2.6416)	2.2519 (2.0866, 2.4498)	2.1109 (1.9793, 2.2359)	2.0046 (1.8668, 2.1761)	1.7928 (1.6423, 1.9621)	1.5052 (1.3846, 1.6033)	1.9637 (1.7834, 2.1008)	2.1214 (1.9387, 2.3104)
2016	2.4297 (2.2344, 2.5796)	2.5481 (2.418, 2.6932)	2.2902 (2.1174, 2.4987)	2.1475 (2.0128, 2.2773)	2.04 (1.8953, 2.2184)	1.8256 (1.672, 1.9977)	1.5343 (1.4082, 1.6389)	1.9983 (1.8115, 2.1441)	2.1577 (1.9681, 2.354)

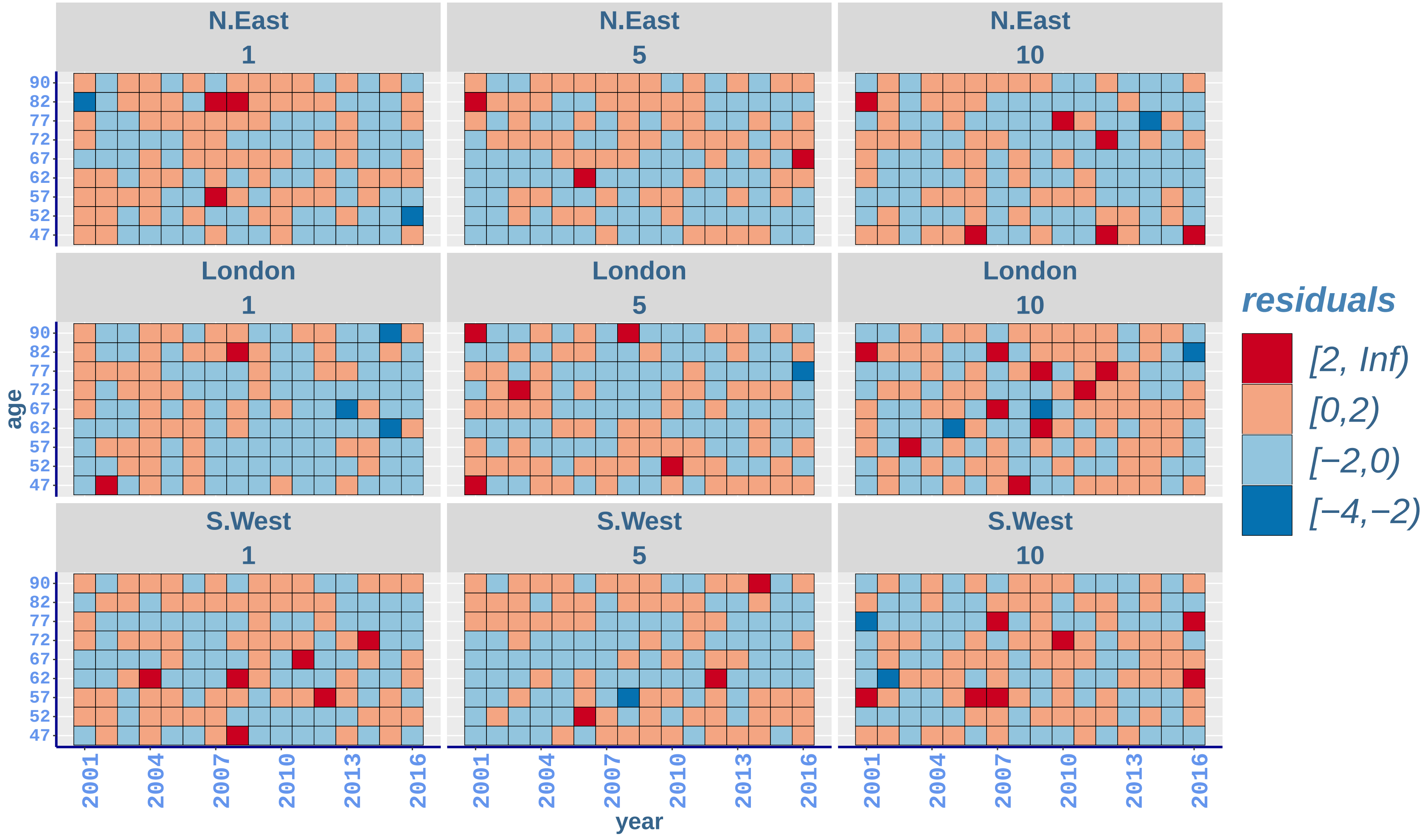

S43 Fig. Heat map of Pearson residuals for trachea, bronchus and lung cancer mortality for males in North East, London, and South West, deprivation deciles 1, 5, and 10: orange/light blue cells indicate areas with good fit, while red/dark blue cells indicate areas with poor fit. Note that there is a small number of residuals greater than 4, and these are included in the last category.

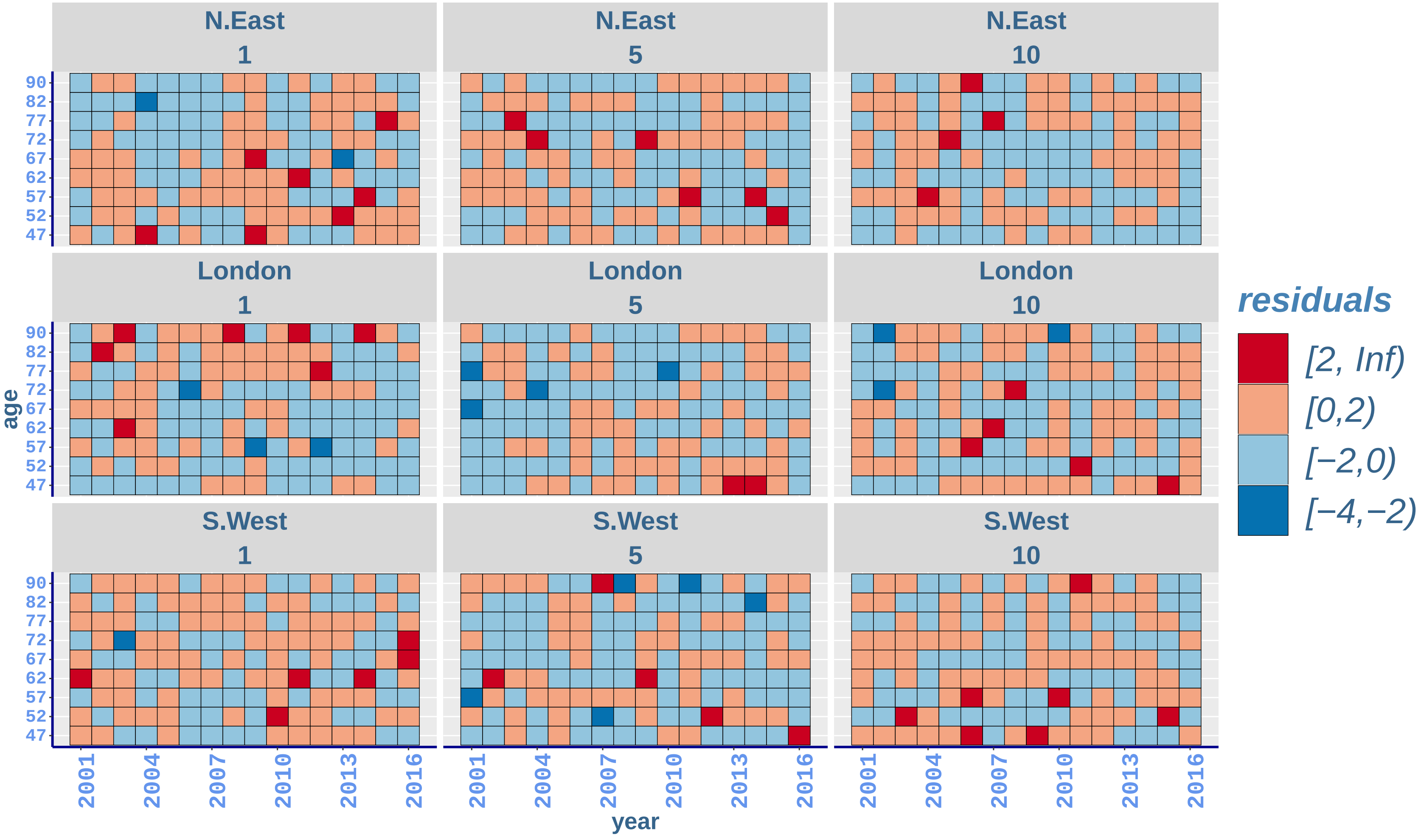

S44 Fig. Heat map of Pearson residuals for trachea, bronchus and lung cancer mortality for females in North East, London, and South West, deprivation deciles 1, 5, and 10: orange/light blue cells indicate areas with good fit, while red/dark blue cells indicate areas with poor fit. Note that there is a small number of residuals greater than 4, and these are included in the last category.

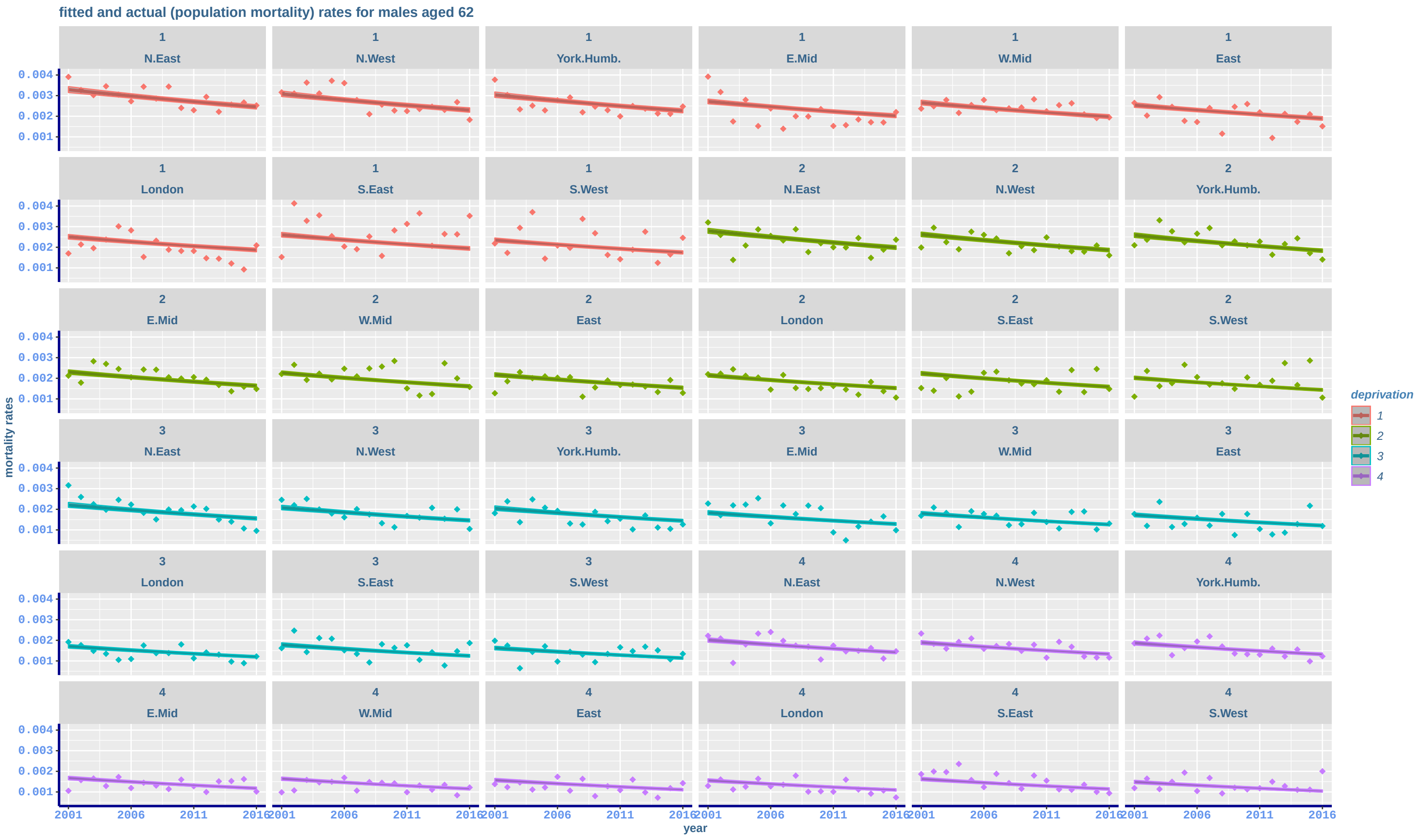

S45 Fig. Trachea, bronchus and lung cancer mortality for males, age 62, deprivation deciles 1-4 for all regions in England between 2001 and 2016: observed rates (dots), fitted rates (lines), with 95% credible intervals for the fitted rates.

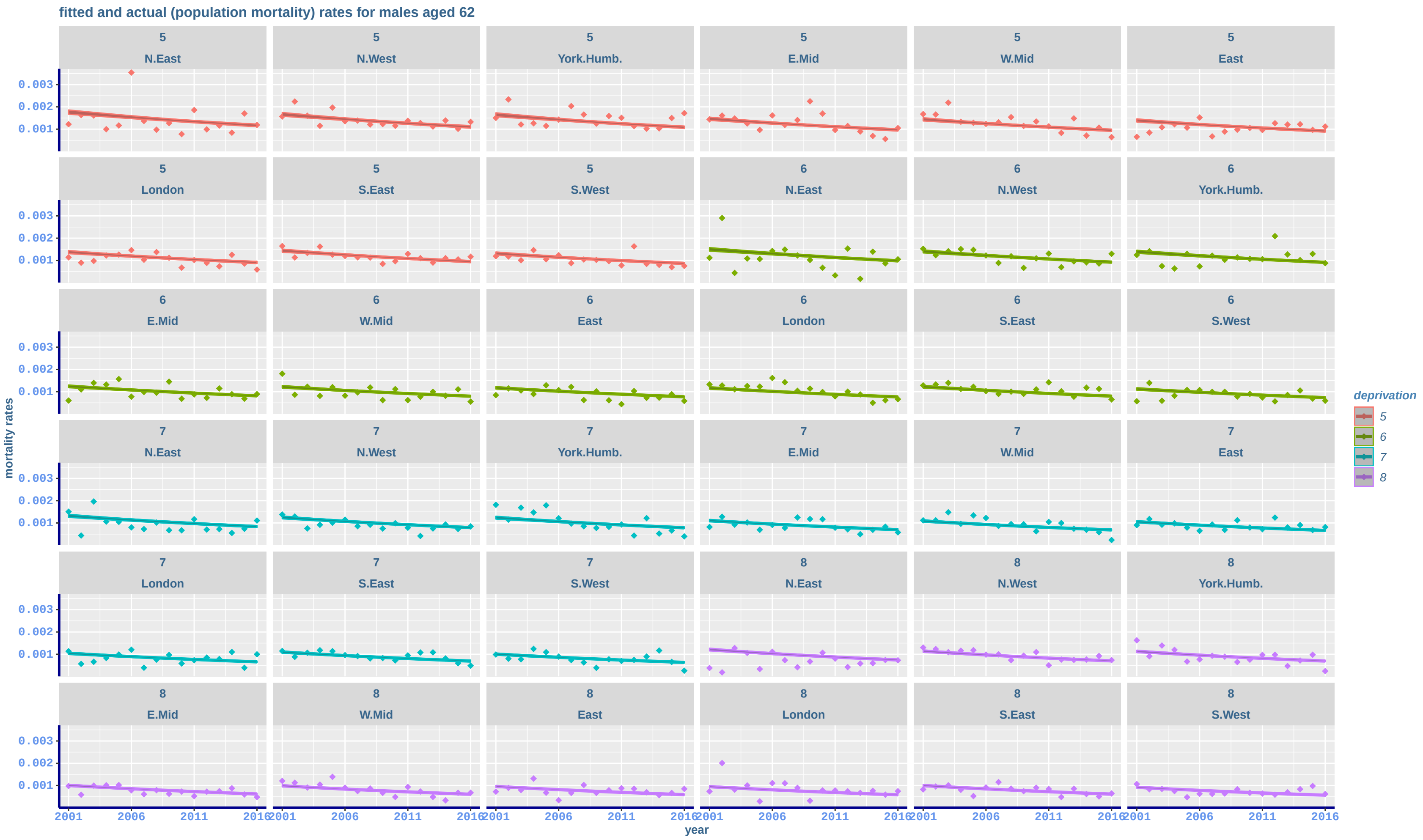

S46 Fig. Trachea, bronchus and lung cancer mortality for males, age 62, deprivation deciles 5-8 for all regions in England between 2001 and 2016: observed rates (dots), fitted rates (lines), with 95% credible intervals for the fitted rates.

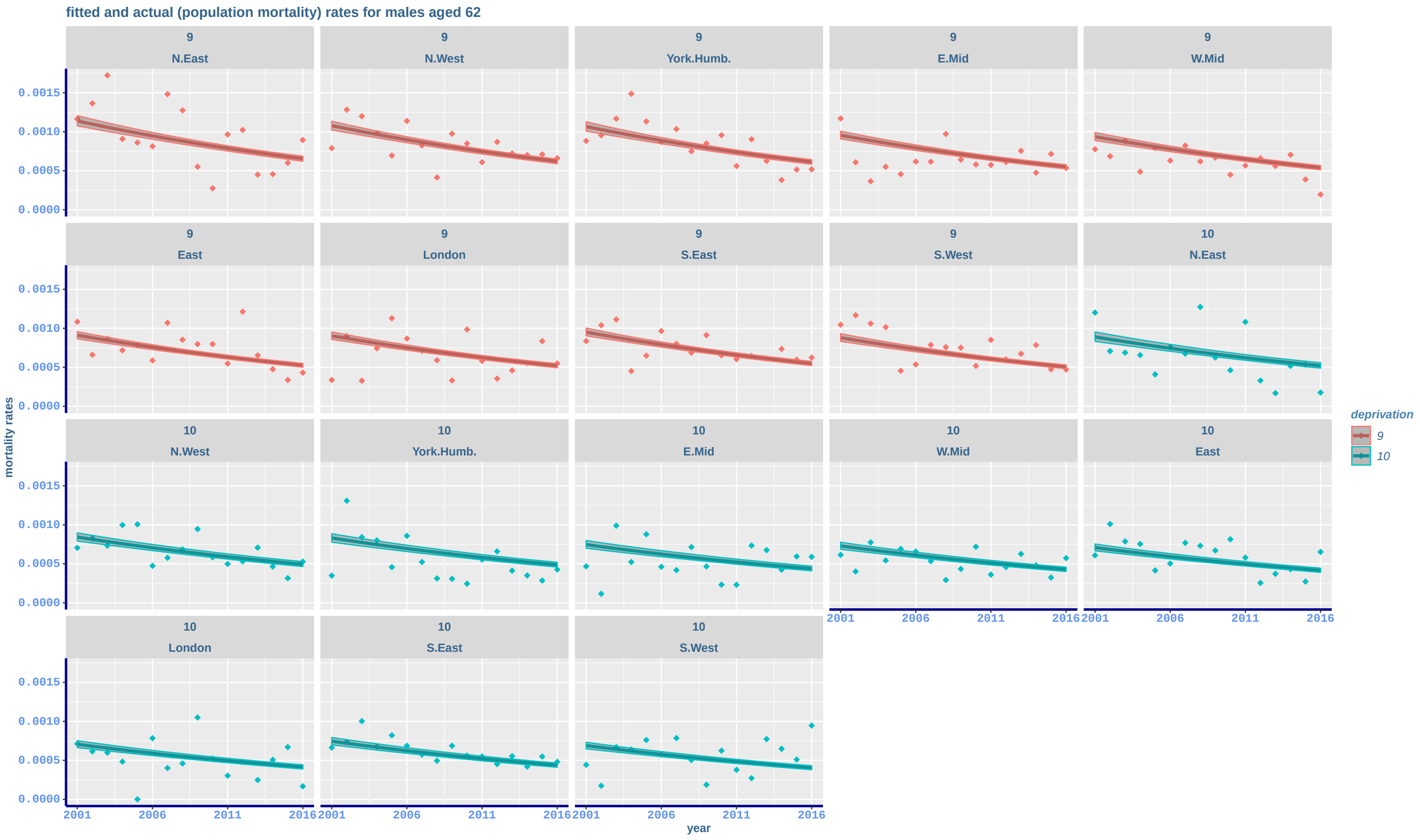

S47 Fig. Trachea, bronchus and lung cancer mortality for males, age 62, deprivation deciles 9-10 for all regions in England between 2001 and 2016: observed rates (dots), fitted rates (lines), with 95% credible intervals for the fitted rates.

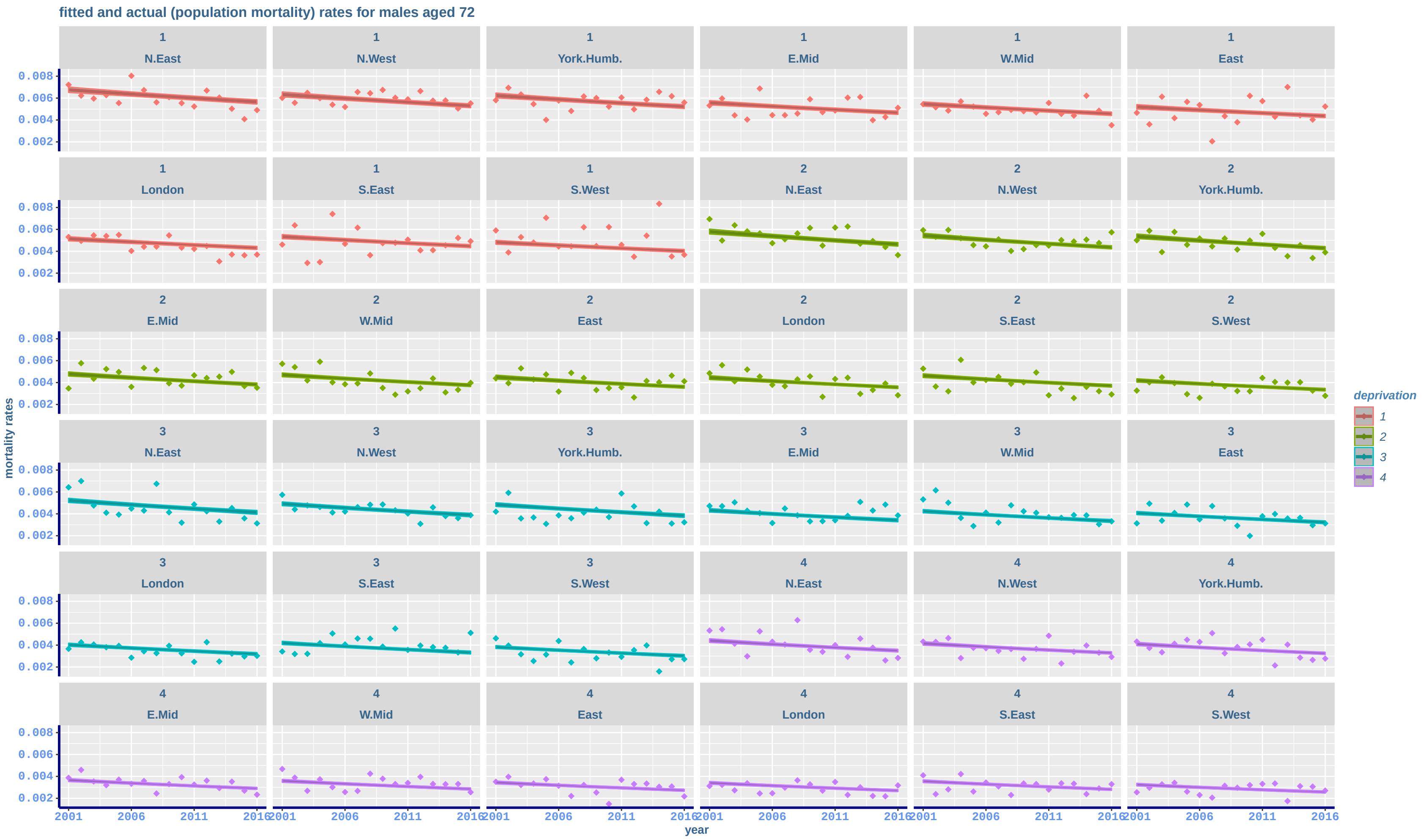

S48 Fig. Trachea, bronchus and lung cancer mortality for males, age 72, deprivation deciles 1-4 for all regions in England between 2001 and 2016: observed rates (dots), fitted rates (lines), with 95% credible intervals for the fitted rates.

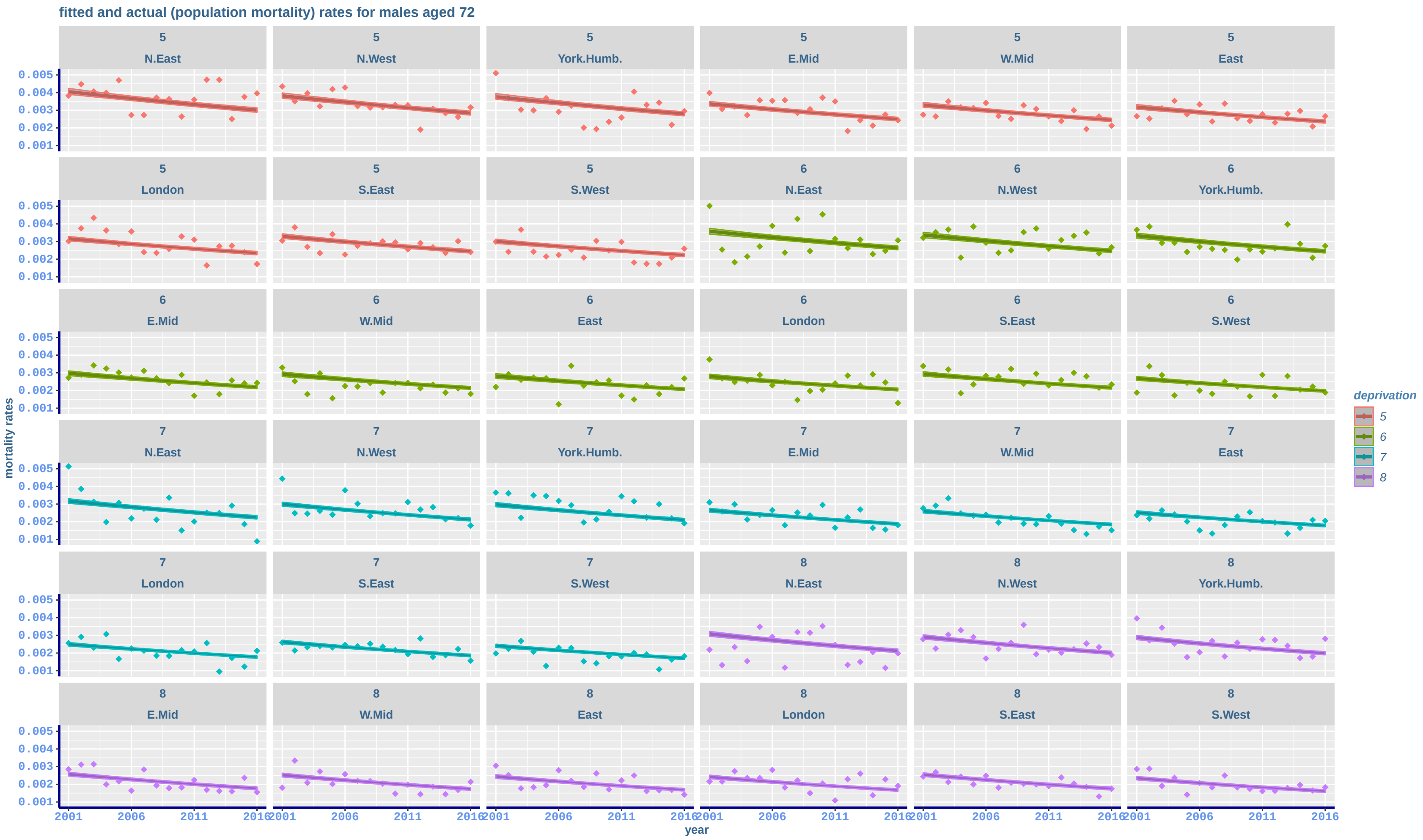

S49 Fig. Trachea, bronchus and lung cancer mortality for males, age 72, deprivation deciles 5-8 for all regions in England between 2001 and 2016: observed rates (dots), fitted rates (lines), with 95% credible intervals for the fitted rates.

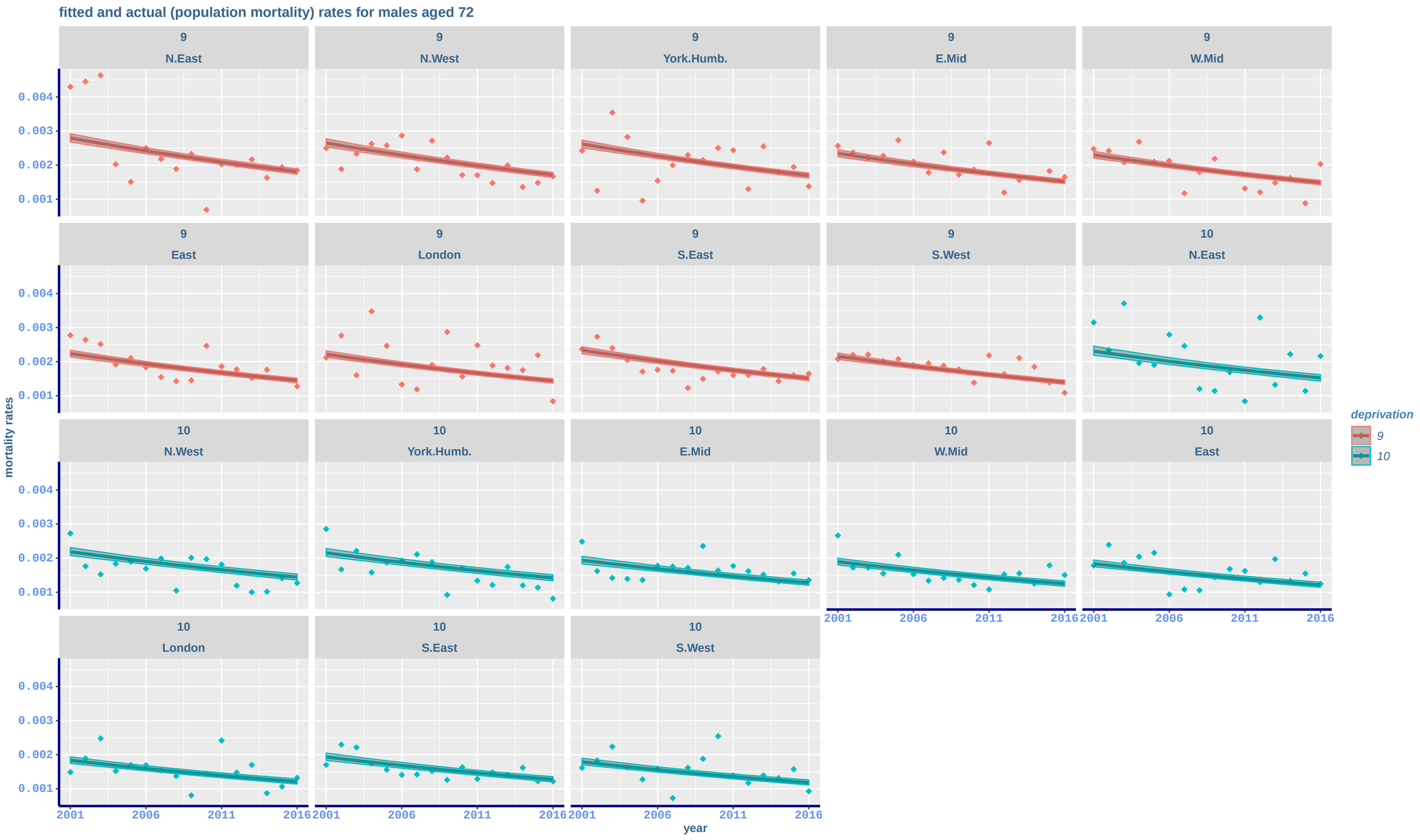

S50 Fig. Trachea, bronchus and lung cancer mortality for males, age 72, deprivation deciles 9-10 for all regions in England between 2001 and 2016: observed rates (dots), fitted rates (lines), with 95% credible intervals for the fitted rates.

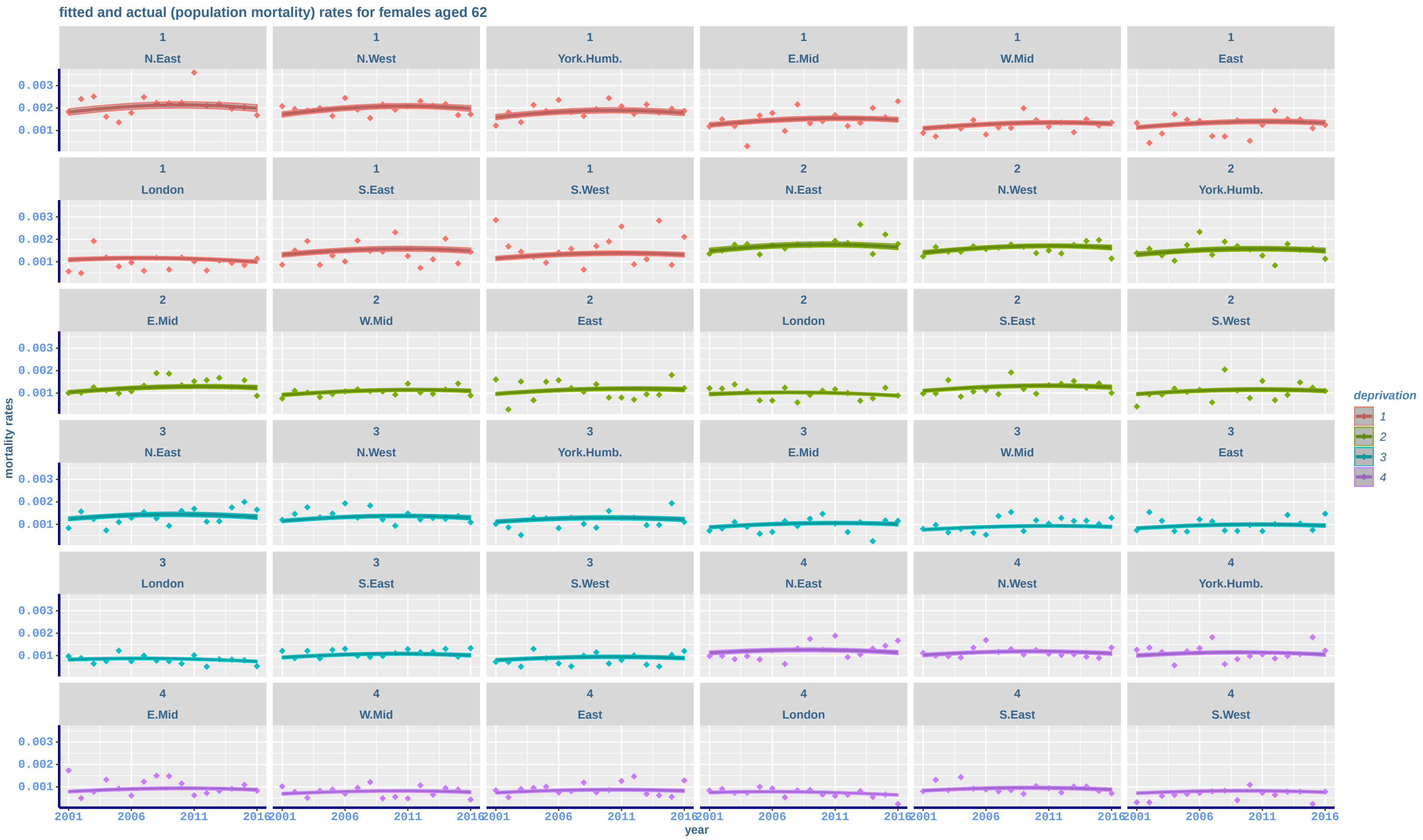

S51 Fig. Trachea, bronchus and lung cancer mortality for females, age 62, deprivation deciles 1-4 for all regions in England between 2001 and 2016: observed rates (dots), fitted rates (lines), with 95% credible intervals for the fitted rates.

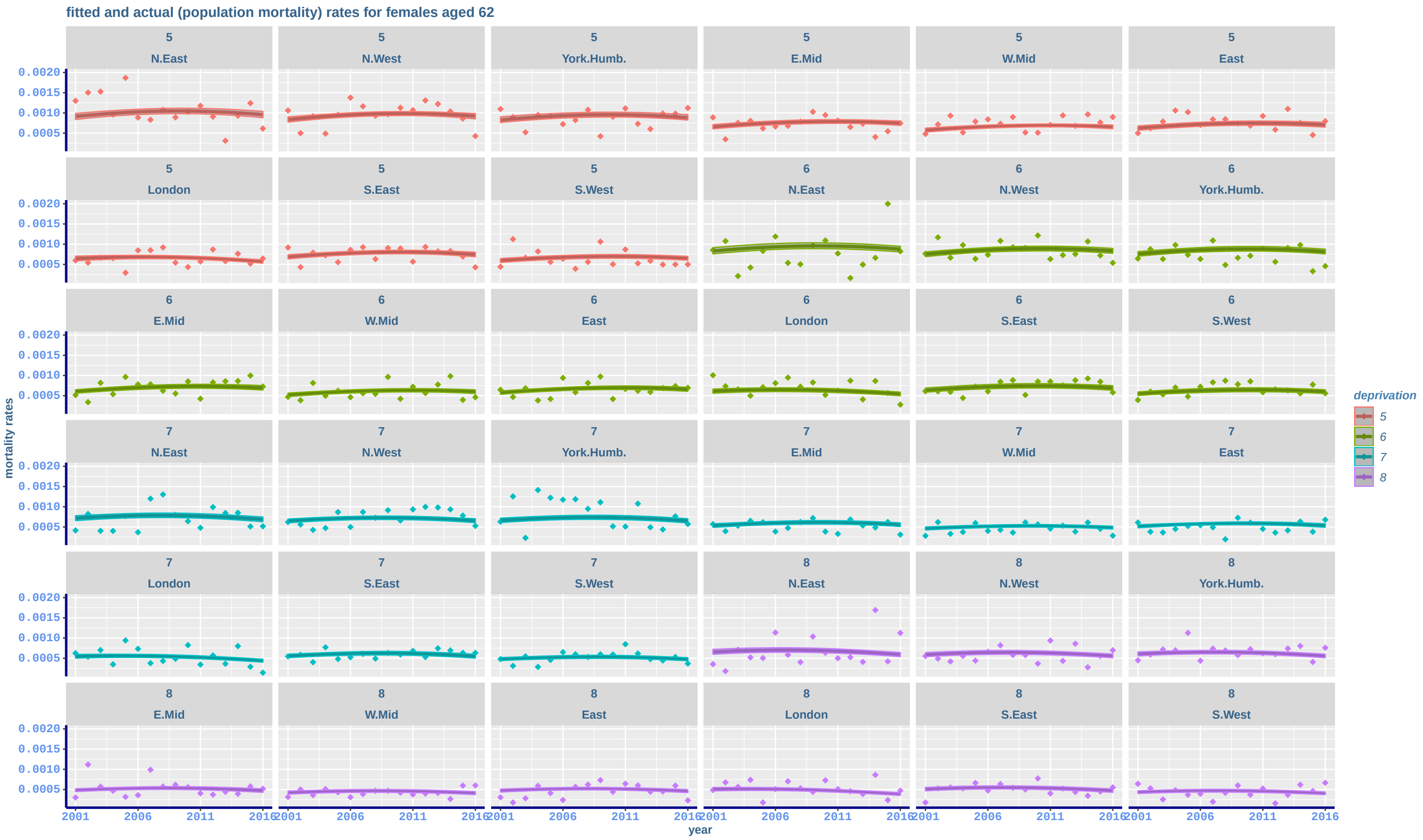

S52 Fig. Trachea, bronchus and lung cancer mortality for females, age 62, deprivation deciles 5-8 for all regions in England between 2001 and 2016: observed rates (dots), fitted rates (lines), with 95% credible intervals for the fitted rates.

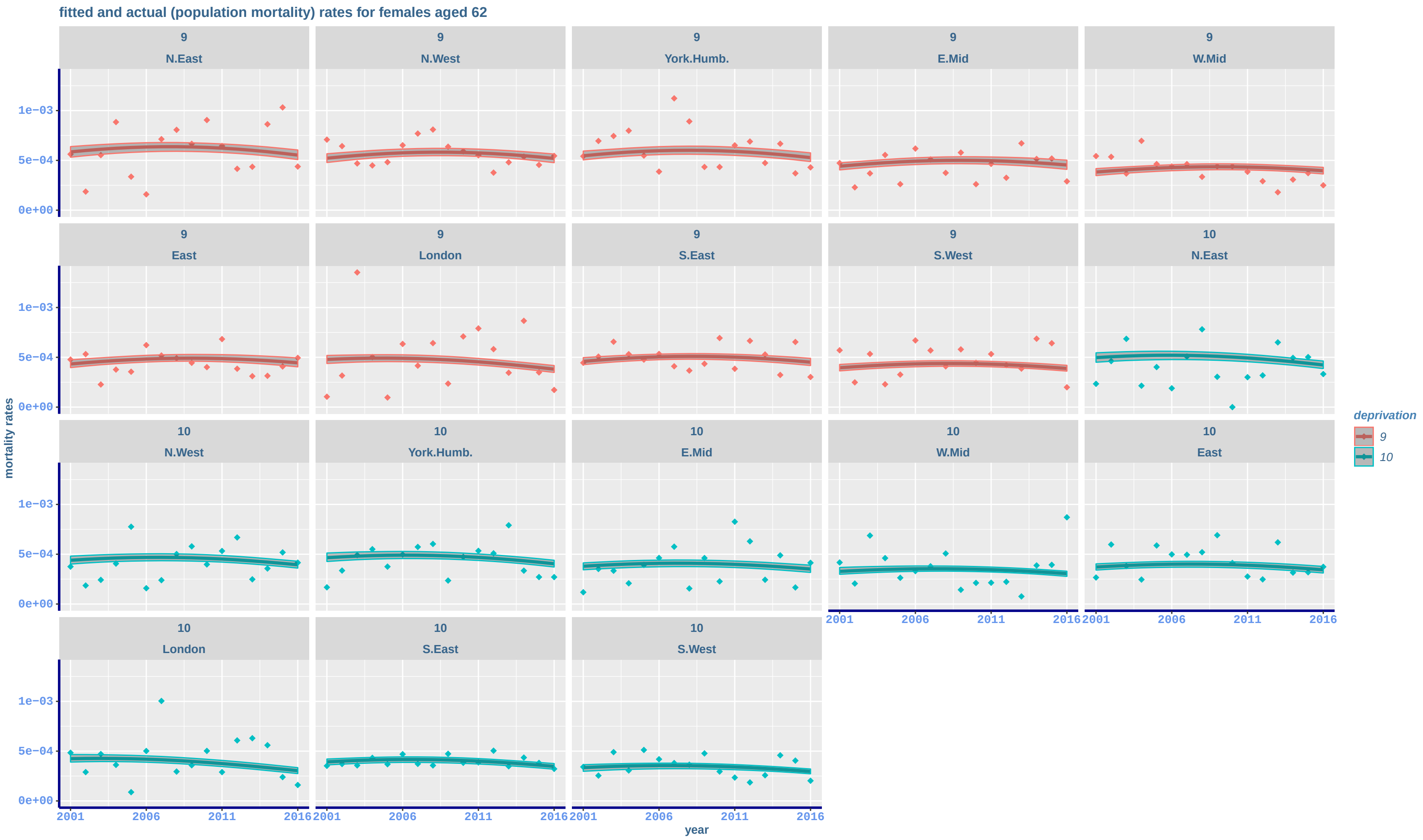

S53 Fig. Trachea, bronchus and lung cancer mortality for females, age 62, deprivation deciles 9-10 for all regions in England between 2001 and 2016: observed rates (dots), fitted rates (lines), with 95% credible intervals for the fitted rates.

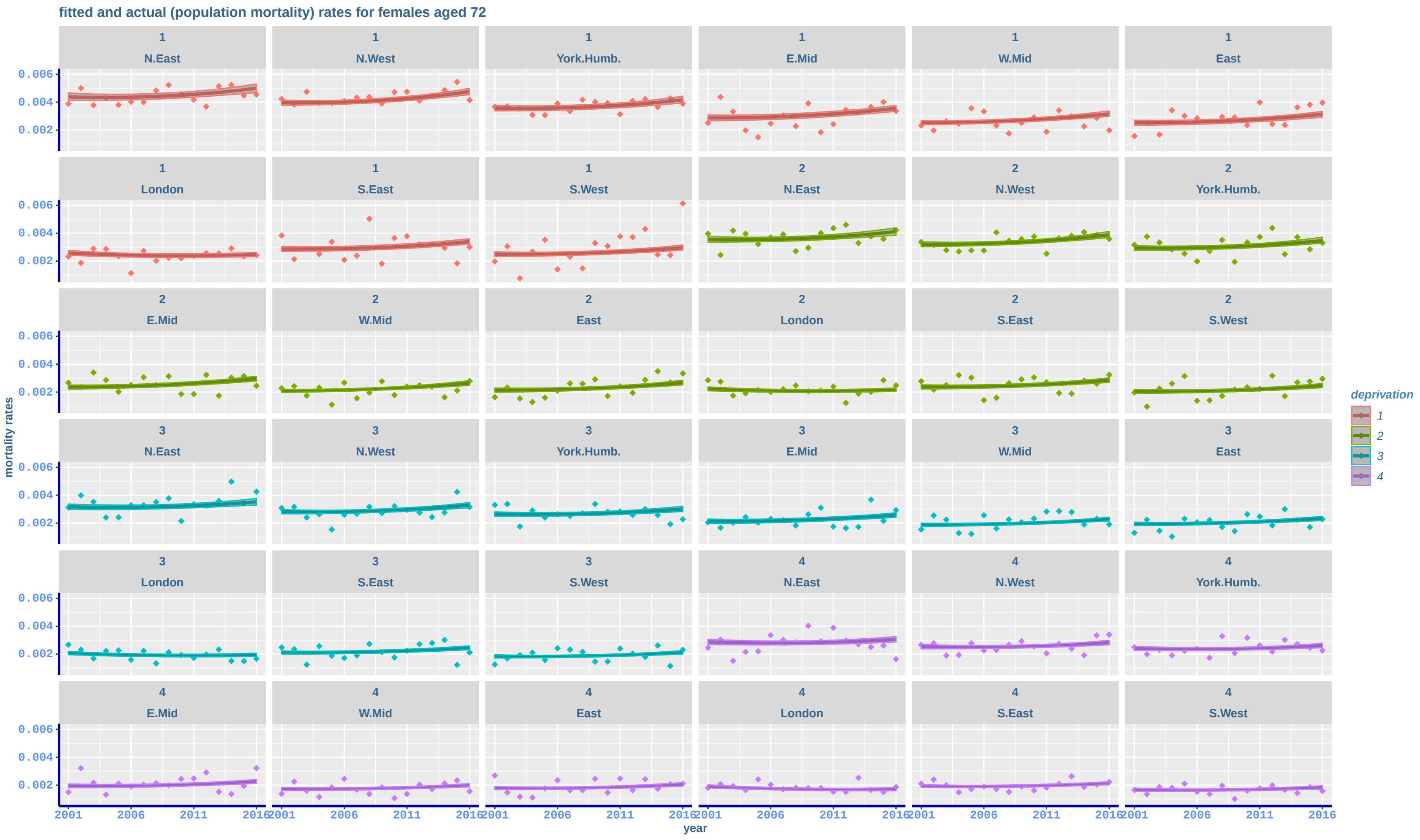

S54 Fig. Trachea, bronchus and lung cancer mortality for females, age 72, deprivation deciles 1-4 for all regions in England between 2001 and 2016: observed rates (dots), fitted rates (lines), with 95% credible intervals for the fitted rates.

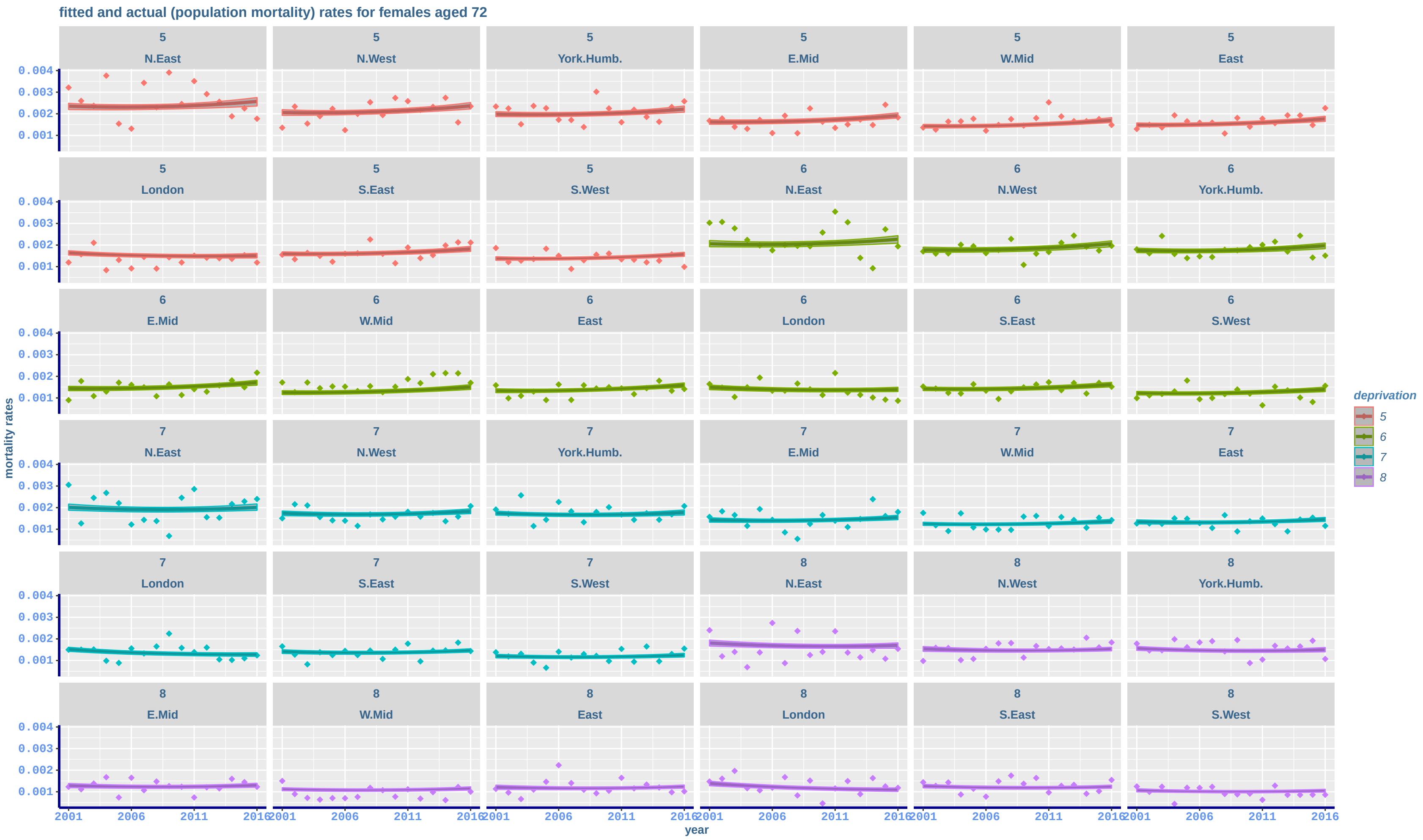

S55 Fig. Trachea, bronchus and lung cancer mortality for females, age 72, deprivation deciles 5-8 for all regions in England between 2001 and 2016: observed rates (dots), fitted rates (lines), with 95% credible intervals for the fitted rates.

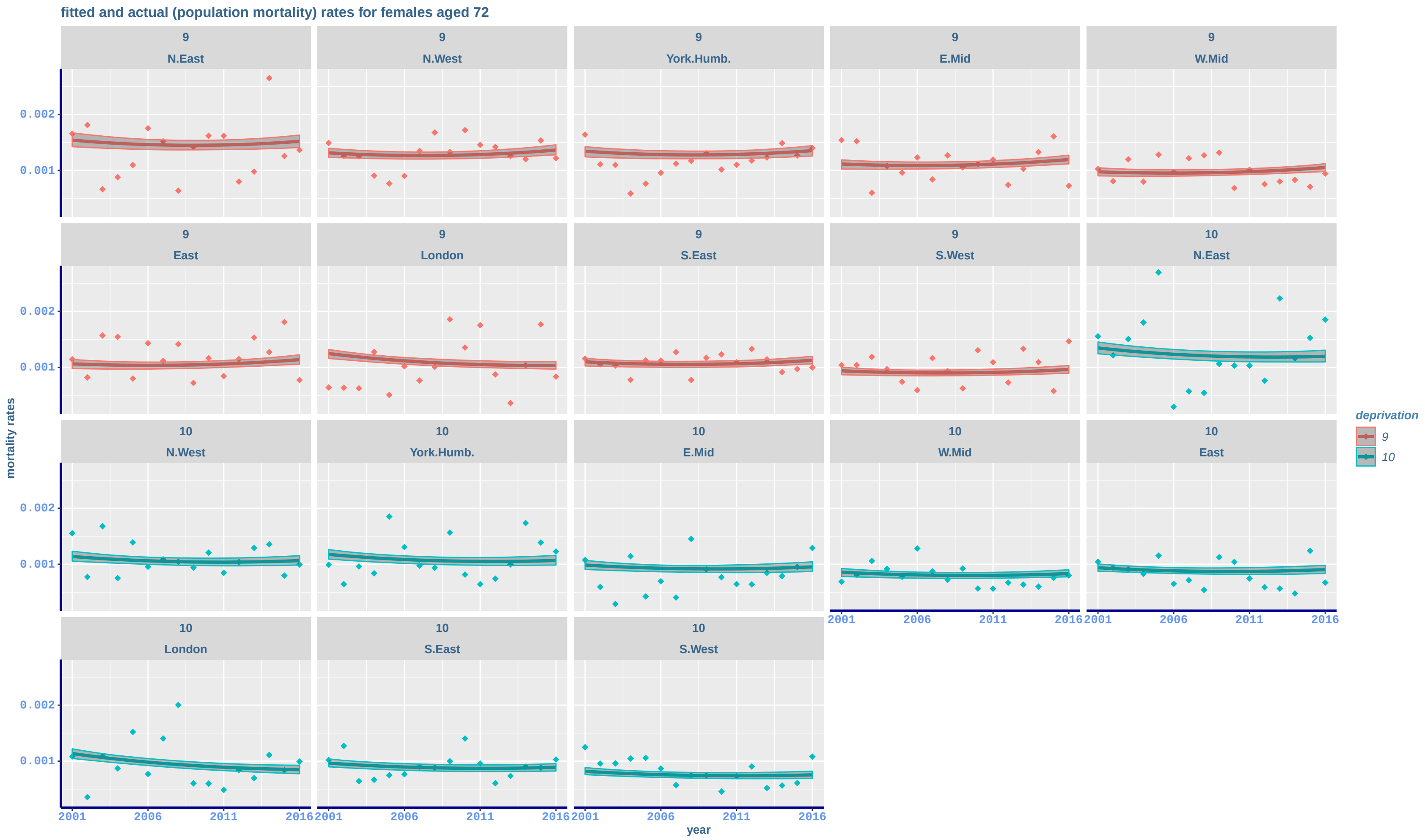

S56 Fig. Trachea, bronchus and lung cancer mortality for females, age 72, deprivation deciles 9-10 for all regions in England between 2001 and 2016: observed rates (dots), fitted rates (lines), with 95% credible intervals for the fitted rates.

S20 Table. Absolute deprivation differences ($AD_{t,r}$), per 100,000 people, in age-standardised fitted mortality rates of lung, trachea and bronchus cancer from 2001 to 2016 for all regions in England for males; 95% credible intervals in brackets

year	N.East	N.West	York.Humb.	E.Mid	W.Mid	East	London	S.East	S.West
2001	253 (243, 263)	235 (226, 244)	232 (223, 240)	206 (198, 214)	202 (194, 209)	191 (183, 199)	187 (179, 195)	192 (184, 200)	170 (162, 177)
2002	252 (243, 261)	234 (226, 242)	231 (223, 238)	205 (198, 213)	201 (194, 208)	190 (183, 198)	187 (179, 193)	192 (185, 198)	170 (163, 176)
2003	251 (243, 259)	234 (226, 241)	230 (223, 237)	205 (198, 212)	200 (194, 206)	189 (183, 196)	186 (179, 192)	191 (185, 197)	169 (163, 175)
2004	250 (243, 257)	233 (226, 239)	229 (223, 235)	204 (198, 210)	200 (194, 205)	189 (183, 195)	185 (179, 191)	191 (185, 196)	169 (163, 174)
2005	249 (242, 256)	232 (225, 237)	228 (223, 234)	203 (197, 209)	199 (194, 204)	188 (183, 194)	185 (179, 190)	190 (184, 195)	168 (163, 173)
2006	248 (242, 254)	231 (225, 236)	227 (222, 232)	203 (197, 208)	198 (193, 203)	188 (183, 193)	184 (179, 189)	189 (184, 194)	168 (163, 172)
2007	247 (241, 253)	230 (224, 235)	227 (222, 231)	202 (196, 207)	197 (193, 202)	187 (182, 192)	184 (179, 188)	189 (184, 193)	167 (163, 171)
2008	246 (240, 252)	229 (224, 234)	226 (221, 230)	201 (195, 206)	197 (192, 201)	186 (182, 192)	183 (178, 187)	188 (183, 192)	167 (162, 171)
2009	245 (239, 251)	228 (223, 233)	225 (220, 230)	200 (195, 205)	196 (191, 200)	185 (181, 191)	182 (178, 186)	187 (183, 192)	166 (162, 170)
2010	244 (238, 250)	227 (222, 232)	224 (219, 229)	199 (193, 205)	195 (190, 199)	185 (180, 190)	182 (177, 186)	187 (182, 191)	166 (161, 169)
2011	243 (237, 249)	226 (221, 231)	223 (218, 229)	199 (192, 204)	194 (189, 199)	184 (179, 190)	181 (176, 186)	186 (181, 191)	165 (160, 169)
2012	242 (236, 248)	225 (220, 231)	222 (216, 229)	198 (191, 204)	193 (188, 199)	183 (178, 189)	180 (175, 185)	185 (180, 191)	165 (160, 169)
2013	241 (234, 247)	224 (218, 231)	221 (215, 228)	197 (190, 203)	193 (187, 198)	183 (177, 189)	179 (174, 185)	185 (179, 190)	164 (159, 169)
2014	240 (233, 247)	223 (217, 230)	220 (214, 228)	196 (189, 203)	192 (186, 198)	182 (176, 189)	179 (173, 185)	184 (178, 190)	164 (158, 169)
2015	239 (231, 247)	222 (216, 230)	219 (212, 227)	195 (188, 203)	191 (185, 198)	181 (175, 188)	178 (172, 184)	183 (177, 190)	163 (157, 169)
2016	238 (230, 246)	222 (214, 230)	218 (211, 227)	194 (187, 202)	190 (183, 197)	180 (174, 188)	177 (171, 184)	183 (176, 190)	162 (156, 169)

S21 Table. Relative deprivation differences ($RD_{t,r}$) in age-standardised fitted mortality rates of lung, trachea and bronchus cancer from 2001 to 2016 for all regions in England for males; 95% credible intervals in brackets

year	N.East	N.West	York.Humb.	E.Mid	W.Mid	East	London	S.East	S.West
2001	1.5859 (1.493, 1.6718)	1.5548 (1.463, 1.6389)	1.5551 (1.463, 1.639)	1.5397 (1.4482, 1.6232)	1.5431 (1.452, 1.6266)	1.5008 (1.4108, 1.5829)	1.4782 (1.3889, 1.5594)	1.4407 (1.3526, 1.5204)	1.3761 (1.2902, 1.4533)
2002	1.6209 (1.5345, 1.6991)	1.5893 (1.5036, 1.6665)	1.5896 (1.5038, 1.6669)	1.574 (1.489, 1.6507)	1.5775 (1.4923, 1.6542)	1.5346 (1.451, 1.6099)	1.5117 (1.429, 1.5861)	1.4737 (1.392, 1.5469)	1.4082 (1.3286, 1.4797)
2003	1.6562 (1.5766, 1.7278)	1.6242 (1.5456, 1.695)	1.6245 (1.5458, 1.6954)	1.6087 (1.5304, 1.6792)	1.6123 (1.5339, 1.6829)	1.5688 (1.4919, 1.638)	1.5456 (1.4694, 1.6139)	1.507 (1.4318, 1.5741)	1.4407 (1.3674, 1.5057)
2004	1.6921 (1.6174, 1.7565)	1.6596 (1.5857, 1.7231)	1.6599 (1.5861, 1.7234)	1.6439 (1.5706, 1.7069)	1.6475 (1.5741, 1.7104)	1.6035 (1.5312, 1.6656)	1.58 (1.5085, 1.6413)	1.5409 (1.4705, 1.6013)	1.4736 (1.4048, 1.5326)
2005	1.7283 (1.6575, 1.7883)	1.6955 (1.6256, 1.7546)	1.6958 (1.626, 1.7549)	1.6796 (1.6102, 1.7383)	1.6832 (1.6136, 1.7421)	1.6386 (1.57, 1.6963)	1.6147 (1.547, 1.6721)	1.5751 (1.5082, 1.6314)	1.5069 (1.4418, 1.5618)
2006	1.7651 (1.6986, 1.8214)	1.7318 (1.6663, 1.7869)	1.7321 (1.6665, 1.7872)	1.7156 (1.6505, 1.7704)	1.7193 (1.654, 1.7741)	1.6741 (1.6101, 1.7278)	1.6499 (1.5864, 1.7033)	1.6098 (1.5469, 1.6623)	1.5407 (1.4794, 1.5921)
2007	1.8023 (1.7378, 1.8578)	1.7685 (1.7053, 1.8234)	1.7688 (1.7056, 1.8235)	1.7522 (1.6893, 1.8064)	1.7559 (1.6928, 1.8105)	1.7101 (1.6484, 1.7636)	1.6856 (1.6243, 1.7386)	1.6449 (1.5851, 1.6967)	1.5749 (1.5168, 1.6251)
2008	1.84 (1.7761, 1.8944)	1.8058 (1.7429, 1.8593)	1.8061 (1.7433, 1.8595)	1.7892 (1.7268, 1.8422)	1.793 (1.7304, 1.8464)	1.7465 (1.6851, 1.7987)	1.7217 (1.6612, 1.7734)	1.6805 (1.6209, 1.7311)	1.6095 (1.5515, 1.6595)
2009	1.8782 (1.8137, 1.9361)	1.8435 (1.7795, 1.9006)	1.8438 (1.7798, 1.9008)	1.8267 (1.7629, 1.8833)	1.8305 (1.7667, 1.8874)	1.7835 (1.7207, 1.8391)	1.7583 (1.6962, 1.8134)	1.7165 (1.6546, 1.7706)	1.6446 (1.5841, 1.6972)
2010	1.9168 (1.8497, 1.9812)	1.8817 (1.815, 1.9449)	1.882 (1.8152, 1.9454)	1.8647 (1.7984, 1.9276)	1.8686 (1.8023, 1.9315)	1.8209 (1.7552, 1.8827)	1.7954 (1.7302, 1.8565)	1.753 (1.6884, 1.8131)	1.6801 (1.6171, 1.7385)
2011	1.956 (1.8832, 2.0287)	1.9204 (1.8489, 1.9923)	1.9207 (1.849, 1.9927)	1.9031 (1.8316, 1.9745)	1.9071 (1.8354, 1.9786)	1.8587 (1.7882, 1.9283)	1.8329 (1.7633, 1.9017)	1.79 (1.7211, 1.8573)	1.7161 (1.6481, 1.7813)
2012	1.9957 (1.9173, 2.0782)	1.9596 (1.8823, 2.041)	1.9599 (1.8824, 2.0413)	1.9421 (1.8653, 2.0231)	1.9461 (1.869, 2.0271)	1.8971 (1.8212, 1.9764)	1.8709 (1.7958, 1.9496)	1.8274 (1.7532, 1.9047)	1.7525 (1.6798, 1.8269)
2013	2.0358 (1.9492, 2.129)	1.9993 (1.9142, 2.0909)	1.9996 (1.9142, 2.0913)	1.9815 (1.8968, 2.0726)	1.9856 (1.9009, 2.0769)	1.9359 (1.8525, 2.0255)	1.9094 (1.8269, 1.9978)	1.8653 (1.7836, 1.9522)	1.7894 (1.7093, 1.8738)
2014	2.0765 (1.9839, 2.1809)	2.0394 (1.948, 2.1424)	2.0398 (1.9481, 2.1428)	2.0215 (1.9303, 2.1236)	2.0256 (1.9342, 2.128)	1.9753 (1.8854, 2.0754)	1.9484 (1.8591, 2.0476)	1.9037 (1.8154, 2.0012)	1.8268 (1.7407, 1.9214)
2015	2.1177 (2.0156, 2.2344)	2.0801 (1.9792, 2.1951)	2.0805 (1.9797, 2.1954)	2.062 (1.9619, 2.176)	2.0661 (1.9659, 2.1805)	2.0151 (1.916, 2.127)	1.9879 (1.8895, 2.0984)	1.9426 (1.8459, 2.0514)	1.8647 (1.7695, 1.9705)
2016	2.1594 (2.0468, 2.2886)	2.1214 (2.0099, 2.2486)	2.1217 (2.0102, 2.2488)	2.1029 (1.9919, 2.2293)	2.1072 (1.9961, 2.2335)	2.0555 (1.9458, 2.1796)	2.0279 (1.9188, 2.1507)	1.982 (1.8748, 2.1028)	1.903 (1.7981, 2.0205)

S22 Table. Absolute deprivation differences ($\mathrm{AD}_{t,r}$), per 100,000 people, in age-standardised fitted mortality rates of lung, trachea and bronchus cancer from 2001 to 2016 for all regions in England for females; 95% credible intervals in brackets

year	N.East	N.West	York.Humb.	E.Mid	W.Mid	East	London	S.East	S.West
2001	146 (137, 156)	138 (130, 145)	119 (111, 127)	92 (83, 100)	83 (77, 88)	77 (72, 85)	66 (60, 73)	94 (88, 100)	86 (80, 93)
2002	151 (142, 161)	142 (135, 149)	123 (115, 131)	95 (86, 103)	86 (80, 91)	80 (75, 88)	68 (62, 75)	97 (91, 103)	89 (84, 96)
2003	156 (147, 165)	147 (140, 154)	127 (120, 134)	99 (90, 106)	89 (84, 94)	83 (78, 90)	70 (64, 76)	100 (94, 106)	92 (87, 98)
2004	160 (152, 169)	152 (145, 158)	131 (124, 138)	102 (94, 109)	92 (87, 98)	87 (81, 93)	71 (66, 78)	103 (98, 109)	95 (90, 101)
2005	165 (156, 174)	156 (149, 162)	134 (128, 141)	105 (98, 112)	95 (90, 100)	90 (84, 96)	73 (67, 79)	106 (101, 112)	98 (93, 104)
2006	169 (160, 177)	161 (154, 166)	138 (131, 145)	109 (101, 115)	98 (93, 103)	92 (87, 99)	74 (69, 81)	109 (104, 115)	101 (95, 106)
2007	173 (164, 181)	165 (158, 170)	141 (135, 148)	112 (105, 118)	101 (96, 106)	95 (89, 102)	76 (70, 82)	112 (106, 118)	103 (98, 109)
2008	176 (168, 185)	168 (162, 174)	144 (138, 151)	115 (108, 121)	104 (99, 109)	98 (92, 104)	77 (72, 84)	115 (109, 121)	106 (100, 111)
2009	179 (171, 188)	172 (166, 178)	147 (141, 154)	117 (111, 124)	107 (102, 112)	100 (94, 107)	78 (73, 85)	117 (111, 124)	108 (102, 113)
2010	182 (174, 191)	175 (169, 181)	150 (143, 157)	120 (113, 126)	109 (104, 115)	103 (96, 109)	79 (74, 85)	119 (113, 126)	110 (104, 116)
2011	185 (176, 194)	178 (172, 184)	152 (145, 159)	122 (116, 129)	111 (106, 117)	105 (98, 112)	80 (74, 86)	121 (115, 128)	111 (105, 117)
2012	187 (178, 197)	181 (174, 187)	154 (147, 161)	124 (118, 131)	113 (108, 119)	107 (100, 114)	80 (75, 87)	123 (116, 130)	113 (106, 119)
2013	189 (179, 199)	183 (176, 190)	156 (148, 163)	126 (120, 134)	115 (110, 122)	109 (102, 116)	80 (75, 87)	124 (117, 132)	114 (108, 121)
2014	190 (180, 201)	185 (177, 192)	157 (149, 165)	128 (121, 136)	117 (111, 124)	110 (103, 118)	81 (75, 88)	126 (118, 134)	115 (108, 122)
2015	191 (180, 203)	186 (178, 194)	158 (149, 166)	129 (122, 137)	118 (112, 126)	111 (104, 119)	80 (74, 88)	127 (119, 135)	116 (109, 123)
2016	192 (180, 205)	187 (178, 196)	159 (149, 167)	130 (123, 139)	119 (113, 127)	112 (105, 121)	80 (74, 88)	127 (119, 137)	117 (109, 124)

S23 Table. Relative deprivation differences ($\mathrm{RD}_{t,r}$) in age-standardised fitted mortality rates of lung, trachea and bronchus cancer from 2001 to 2016 for all regions in England for females; 95% credible intervals in brackets

year	N.East	N.West	York.Humb.	E.Mid	W.Mid	East	London	S.East	S.West
2001	1.7543 (1.5821, 1.938)	1.9896 (1.8248, 2.1551)	1.625 (1.4767, 1.7981)	1.5346 (1.3612, 1.7077)	1.5404 (1.3812, 1.7149)	1.3278 (1.1981, 1.4751)	0.8839 (0.7758, 1.01)	1.5668 (1.4372, 1.7031)	1.6486 (1.4994, 1.8072)
2002	1.7876 (1.619, 1.9657)	2.0261 (1.8665, 2.1832)	1.6571 (1.5138, 1.8243)	1.5657 (1.3969, 1.7316)	1.5712 (1.4174, 1.7348)	1.3558 (1.227, 1.4974)	0.9057 (0.7988, 1.0294)	1.5975 (1.4719, 1.728)	1.6796 (1.5355, 1.8341)
2003	1.8216 (1.6576, 1.9971)	2.0634 (1.9078, 2.215)	1.6899 (1.551, 1.8517)	1.5974 (1.4312, 1.7566)	1.6026 (1.4541, 1.7562)	1.3844 (1.2586, 1.5216)	0.928 (0.8227, 1.0517)	1.6288 (1.5072, 1.7522)	1.7113 (1.5731, 1.8617)
2004	1.8564 (1.6945, 2.0293)	2.1015 (1.9501, 2.2491)	1.7233 (1.5883, 1.8816)	1.6298 (1.4655, 1.7831)	1.6347 (1.4907, 1.7834)	1.4136 (1.2877, 1.5484)	0.9509 (0.847, 1.0759)	1.6607 (1.5427, 1.7797)	1.7437 (1.6091, 1.8929)
2005	1.892 (1.7336, 2.0642)	2.1405 (1.9944, 2.2876)	1.7575 (1.6252, 1.9118)	1.6629 (1.4999, 1.8092)	1.6675 (1.5282, 1.8116)	1.4434 (1.316, 1.5746)	0.9744 (0.872, 1.1014)	1.6934 (1.5776, 1.8095)	1.7769 (1.6447, 1.9262)
2006	1.9284 (1.7715, 2.103)	2.1803 (2.0373, 2.3249)	1.7924 (1.663, 1.9421)	1.6967 (1.5349, 1.8386)	1.7011 (1.5653, 1.8398)	1.4739 (1.3451, 1.6037)	0.9985 (0.897, 1.1266)	1.7267 (1.6114, 1.8449)	1.8107 (1.6797, 1.9602)
2007	1.9657 (1.8082, 2.1433)	2.2211 (2.0819, 2.3627)	1.8281 (1.6988, 1.9751)	1.7312 (1.5706, 1.8716)	1.7355 (1.6025, 1.8737)	1.5051 (1.3746, 1.633)	1.0232 (0.921, 1.1528)	1.7608 (1.6429, 1.8831)	1.8454 (1.7121, 1.9962)
2008	2.0038 (1.8431, 2.185)	2.2628 (2.126, 2.4041)	1.8646 (1.7329, 2.0129)	1.7666 (1.6042, 1.9051)	1.7706 (1.6401, 1.9117)	1.537 (1.4035, 1.6637)	1.0485 (0.9438, 1.1826)	1.7957 (1.6749, 1.9247)	1.8808 (1.7442, 2.0328)
2009	2.0429 (1.8795, 2.2295)	2.3055 (2.1673, 2.45)	1.9019 (1.7651, 2.0517)	1.8027 (1.639, 1.944)	1.8065 (1.6769, 1.9486)	1.5697 (1.4329, 1.6981)	1.0745 (0.9681, 1.2124)	1.8313 (1.7111, 1.9658)	1.9171 (1.7726, 2.0725)
2010	2.083 (1.9136, 2.2778)	2.3492 (2.2081, 2.4978)	1.9401 (1.7981, 2.0918)	1.8397 (1.6745, 1.9867)	1.8434 (1.7121, 1.9861)	1.6031 (1.462, 1.7354)	1.1012 (0.9916, 1.2435)	1.8679 (1.7425, 2.0119)	1.9542 (1.8015, 2.1133)
2011	2.1242 (1.9464, 2.3278)	2.394 (2.2496, 2.5459)	1.9792 (1.8289, 2.1341)	1.8776 (1.712, 2.0312)	1.8811 (1.7487, 2.0271)	1.6374 (1.4915, 1.773)	1.1286 (1.0162, 1.275)	1.9053 (1.7749, 2.0579)	1.9923 (1.8289, 2.1563)
2012	2.1664 (1.9817, 2.3787)	2.4401 (2.2859, 2.6011)	2.0193 (1.8578, 2.1752)	1.9164 (1.7498, 2.0776)	1.9199 (1.784, 2.0709)	1.6725 (1.5221, 1.813)	1.1568 (1.0424, 1.3124)	1.9437 (1.8044, 2.1038)	2.0314 (1.855, 2.204)
2013	2.2098 (2.0171, 2.4313)	2.4873 (2.3221, 2.657)	2.0605 (1.885, 2.2204)	1.9563 (1.7877, 2.1257)	1.9597 (1.8204, 2.1138)	1.7086 (1.5535, 1.855)	1.1858 (1.0677, 1.3492)	1.9831 (1.8341, 2.154)	2.0715 (1.8818, 2.254)
2014	2.2545 (2.0522, 2.4848)	2.5359 (2.3577, 2.7179)	2.1029 (1.9092, 2.2699)	1.9973 (1.8239, 2.1757)	2.0006 (1.8541, 2.1609)	1.7458 (1.5835, 1.8995)	1.2156 (1.0919, 1.3889)	2.0236 (1.8645, 2.2069)	2.1127 (1.9091, 2.3053)
2015	2.3006 (2.0862, 2.5445)	2.586 (2.3935, 2.7855)	2.1465 (1.935, 2.3236)	2.0394 (1.8597, 2.2294)	2.0427 (1.8872, 2.2104)	1.784 (1.6133, 1.9442)	1.2465 (1.1152, 1.4292)	2.0653 (1.8945, 2.2639)	2.1552 (1.9368, 2.36)
2016	2.3482 (2.1216, 2.6041)	2.6377 (2.4294, 2.8557)	2.1914 (1.9622, 2.3786)	2.0829 (1.8974, 2.2849)	2.0862 (1.9205, 2.2651)	1.8234 (1.6435, 1.9919)	1.2783 (1.1389, 1.472)	2.1084 (1.9225, 2.321)	2.1991 (1.9665, 2.4213)

S24 Table. Estimated coefficients for the best fitting model for bowel cancer morbidity.

Covariate	Parameter	Mean	SD	%2.5	%97.5	Covariate	Parameter	Mean	SD	%2.5	%97.5
Intercept	β_0	-6.3250	0.0038	-6.3320	-6.3170		$\beta_{7,\text{gender}_F,\text{age}_9}$	0.0982	0.0079	0.0824	0.1134
Age	β_{1,age_1}	-2.0270	0.0150	-2.0560	-1.9990	Age : Year $\leq$ 2006	$\beta_{8,1,\text{age}_1}$	-0.0104	0.0154	-0.0403	0.0188
	β_{1,age_2}	-1.3420	0.0111	-1.3620	-1.3200		$\beta_{8,1,\text{age}_2}$	-0.0028	0.0124	-0.0260	0.0231
	β_{1,age_3}	-0.7361	0.0099	-0.7560	-0.7177		$\beta_{8,1,\text{age}_3}$	-0.0380	0.0099	-0.0570	-0.0180
	β_{1,age_4}	-0.1000	0.0083	-0.1156	-0.0822		$\beta_{8,1,\text{age}_4}$	0.0555	0.0090	0.0375	0.0734
	β_{1,age_5}	0.3358	0.0073	0.3219	0.3512		$\beta_{8,1,\text{age}_5}$	0.0586	0.0082	0.0430	0.0754
	β_{1,age_6}	0.6021	0.0071	0.5883	0.6169		$\beta_{8,1,\text{age}_6}$	-0.0019	0.0070	-0.0157	0.0116
	β_{1,age_7}	0.8542	0.0066	0.8400	0.8664		$\beta_{8,1,\text{age}_7}$	-0.0183	0.0072	-0.0336	-0.0053
	β_{1,age_8}	1.0290	0.0071	1.0150	1.0430		$\beta_{8,1,\text{age}_8}$	0.0049	0.0075	-0.0102	0.0187
	β_{1,age_9}	1.3840	0.0083	1.3680	1.3990		$\beta_{8,1,\text{age}_9}$	-0.0476	0.0082	-0.0637	-0.0313
Year $\leq$ 2006	$\beta_{2,1}$	0.0435	0.0118	0.0188	0.0639	Age : Year > 2006	$\beta_{8,2,\text{age}_1}$	0.0605	0.0149	0.0299	0.0880
Year > 2006	$\beta_{2,2}$	0.0358	0.0077	0.0207	0.0508		$\beta_{8,2,\text{age}_2}$	0.0503	0.0116	0.0271	0.0717
Gender	β_3	-0.3983	0.0051	-0.4085	-0.3886		$\beta_{8,2,\text{age}_3}$	0.0607	0.0091	0.0423	0.0791
Region	$\beta_{4,\text{region}_1}$	0.1140	0.0073	0.0997	0.1283		$\beta_{8,2,\text{age}_4}$	-0.0079	0.0086	-0.0253	0.0093
	$\beta_{4,\text{region}_2}$	0.0476	0.0051	0.0373	0.0573		$\beta_{8,2,\text{age}_5}$	-0.1317	0.0075	-0.1474	-0.1176
	$\beta_{4,\text{region}_3}$	0.0155	0.0058	0.0042	0.0273		$\beta_{8,2,\text{age}_6}$	-0.0365	0.0070	-0.0504	-0.0229
	$\beta_{4,\text{region}_4}$	-0.0012	0.0059	-0.0131	0.0104		$\beta_{8,2,\text{age}_7}$	-0.0532	0.0070	-0.0664	-0.0384
	$\beta_{4,\text{region}_5}$	0.0200	0.0056	0.0086	0.0309		$\beta_{8,2,\text{age}_8}$	-0.0020	0.0070	-0.0161	0.0114
	$\beta_{4,\text{region}_6}$	-0.0360	0.0053	-0.0467	-0.0255		$\beta_{8,2,\text{age}_9}$	0.0599	0.0075	0.0455	0.0752
	$\beta_{4,\text{region}_7}$	-0.1277	0.0059	-0.1394	-0.1167	Gender:Region	$\beta_{9,\text{gender}_F,\text{region}_1}$	-0.1018	0.0111	-0.1227	-0.0808
	$\beta_{4,\text{region}_8}$	-0.0375	0.0049	-0.0473	-0.0279		$\beta_{9,\text{gender}_F,\text{region}_2}$	-0.0527	0.0078	-0.0672	-0.0370
	$\beta_{4,\text{region}_9}$	0.0053	0.0054	-0.0050	0.0160		$\beta_{9,\text{gender}_F,\text{region}_3}$	-0.0563	0.0090	-0.0734	-0.0391
Deprivation	$\beta_{5,\text{deprivation}_1}$	0.0660	0.0065	0.0536	0.0786		$\beta_{9,\text{gender}_F,\text{region}_4}$	-0.0117	0.0092	-0.0292	0.0078
	$\beta_{5,\text{deprivation}_2}$	0.0494	0.0064	0.0367	0.0617		$\beta_{9,\text{gender}_F,\text{region}_5}$	-0.0354	0.0082	-0.0515	-0.0198
	$\beta_{5,\text{deprivation}_3}$	0.0361	0.0061	0.0241	0.0479		$\beta_{9,\text{gender}_F,\text{region}_6}$	0.0700	0.0077	0.0552	0.0854
	$\beta_{5,\text{deprivation}_4}$	0.0112	0.0061	-0.0003	0.0235		$\beta_{9,\text{gender}_F,\text{region}_7}$	0.0594	0.0089	0.0426	0.0775
	$\beta_{5,\text{deprivation}_5}$	-0.0040	0.0056	-0.0149	0.0069		$\beta_{9,\text{gender}_F,\text{region}_8}$	0.0660	0.0072	0.0516	0.0796
	$\beta_{5,\text{deprivation}_6}$	-0.0034	0.0056	-0.0142	0.0076		$\beta_{9,\text{gender}_F,\text{region}_9}$	0.0625	0.0078	0.0471	0.0774
	$\beta_{5,\text{deprivation}_7}$	-0.0247	0.0058	-0.0364	-0.0132	Gender:Deprivation	$\beta_{10,\text{gender}_F,\text{deprivation}_1}$	-0.0486	0.0097	-0.0668	-0.0295
	$\beta_{5,\text{deprivation}_8}$	-0.0300	0.0057	-0.0408	-0.0185		$\beta_{10,\text{gender}_F,\text{deprivation}_2}$	-0.0403	0.0092	-0.0583	-0.0210
	$\beta_{5,\text{deprivation}_9}$	-0.0509	0.0057	-0.0622	-0.0400		$\beta_{10,\text{gender}_F,\text{deprivation}_3}$	-0.0306	0.0090	-0.0489	-0.0131
	$\beta_{5,\text{deprivation}_{10}}$	-0.0496	0.0059	-0.0614	-0.0382		$\beta_{10,\text{gender}_F,\text{deprivation}_4}$	0.0002	0.0088	-0.0174	0.0161
(Year $\leq$ 2006)^2	$\beta_{6,1}$	0.0041	0.0077	-0.0114	0.0174		$\beta_{10,\text{gender}_F,\text{deprivation}_5}$	0.0037	0.0086	-0.0137	0.0204
(Year < 2006)^2	$\beta_{6,2}$	-0.0522	0.0053	-0.0623	-0.0416		$\beta_{10,\text{gender}_F,\text{deprivation}_6}$	0.0090	0.0081	-0.0074	0.0244
Age:Gender	$\beta_{7,\text{gender}_F,\text{age}_1}$	0.2574	0.0161	0.2258	0.2889		$\beta_{10,\text{gender}_F,\text{deprivation}_7}$	0.0178	0.0083	0.0013	0.0343
	$\beta_{7,\text{gender}_F,\text{age}_2}$	0.1411	0.0127	0.1159	0.1660		$\beta_{10,\text{gender}_F,\text{deprivation}_8}$	0.0248	0.0083	0.0081	0.0412
	$\beta_{7,\text{gender}_F,\text{age}_3}$	0.0138	0.0094	-0.0037	0.0335		$\beta_{10,\text{gender}_F,\text{deprivation}_9}$	0.0404	0.0081	0.0249	0.0566
	$\beta_{7,\text{gender}_F,\text{age}_4}$	-0.1140	0.0089	-0.1315	-0.0970		$\beta_{10,\text{gender}_F,\text{deprivation}_{10}}$	0.0237	0.0088	0.0074	0.0411
	$\beta_{7,\text{gender}_F,\text{age}_5}$	-0.1364	0.0081	-0.1527	-0.1210	Gender : Year $\leq$ 2006	$\beta_{11,1,\text{gender}_F}$	0.0105	0.0057	-0.0001	0.0219
	$\beta_{7,\text{gender}_F,\text{age}_6}$	-0.1271	0.0075	-0.1418	-0.1132	Gender : Year > 2006	$\beta_{11,2,\text{gender}_F}$	0.0220	0.0054	0.0113	0.0333
	$\beta_{7,\text{gender}_F,\text{age}_7}$	-0.0877	0.0071	-0.1021	-0.0741		σ^2	0.0027	0.0004	0.0019	0.0035
	$\beta_{7,\text{gender}_F,\text{age}_8}$	-0.0454	0.0073	-0.0595	-0.0309						

S25 Table. Estimated coefficients for the best fitting model for male bowel cancer mortality.

Covariate	Parameter	Mean	SD	%2.5	%97.5	Covariate	Parameter	Mean	SD	%2.5	%97.5
Intercept	β_0	-7.3450	0.0043	-7.3540	-7.3370	Deprivation	$\beta_{5,\text{deprivation}_1}$	1.3100	0.0385	1.2350	1.3820
Age	β_{1,age_1}	-2.2340	0.0222	-2.2810	-2.1900		$\beta_{5,\text{deprivation}_2}$	0.9063	0.0270	0.8548	0.9575
	β_{1,age_2}	-1.5390	0.0155	-1.5700	-1.5110		$\beta_{5,\text{deprivation}_3}$	0.2916	0.0114	0.2692	0.3142
	β_{1,age_3}	-0.8535	0.0114	-0.8759	-0.8307		$\beta_{5,\text{deprivation}_4}$	0.1116	0.0097	0.0927	0.1312
	β_{1,age_4}	-0.3376	0.0095	-0.3563	-0.3202		$\beta_{5,\text{deprivation}_5}$	0.4596	0.0169	0.4290	0.4902
	β_{1,age_5}	0.1307	0.0085	0.1142	0.1477		$\beta_{5,\text{deprivation}_6}$	-0.5367	0.0191	-0.5725	-0.4993
	β_{1,age_6}	0.5598	0.0079	0.5440	0.5757		$\beta_{5,\text{deprivation}_7}$	-0.2256	0.0105	-0.2461	-0.2054
	β_{1,age_7}	0.9496	0.0077	0.9341	0.9644		$\beta_{5,\text{deprivation}_8}$	-0.7027	0.0231	-0.7446	-0.6575
	β_{1,age_8}	1.3040	0.0076	1.2890	1.3190		$\beta_{5,\text{deprivation}_9}$	-0.6713	0.0201	-0.7099	-0.6324
	β_{1,age_9}	2.0190	0.0079	2.0040	2.0350		$\beta_{5,\text{deprivation}_{10}}$	-0.9429	0.0272	-0.9948	-0.8911
Year	β_2	-0.0826	0.0040	-0.0905	-0.0749	Age:Year	β_{6,age_1}	0.0197	0.0198	-0.0191	0.0578
AAD	β_3	4.0280	0.1304	3.7770	4.2790		β_{6,age_2}	-0.0041	0.0157	-0.0350	0.0277
Region	$\beta_{4,\text{region}_1}$	0.3349	0.0135	0.3085	0.3607		β_{6,age_3}	-0.0215	0.0104	-0.0423	-0.0019
	$\beta_{4,\text{region}_2}$	1.0260	0.0330	0.9620	1.0880		β_{6,age_4}	-0.0174	0.0098	-0.0369	0.0022
	$\beta_{4,\text{region}_3}$	0.3869	0.0153	0.3568	0.4143		β_{6,age_5}	-0.0538	0.0084	-0.0709	-0.0382
	$\beta_{4,\text{region}_4}$	0.7059	0.0242	0.6609	0.7532		β_{6,age_6}	-0.0409	0.0079	-0.0565	-0.0259
	$\beta_{4,\text{region}_5}$	0.8054	0.0261	0.7539	0.8539		β_{6,age_7}	-0.0147	0.0072	-0.0293	-0.0001
	$\beta_{4,\text{region}_6}$	-0.6659	0.0227	-0.7065	-0.6239		β_{6,age_8}	0.0418	0.0075	0.0271	0.0568
	$\beta_{4,\text{region}_7}$	-1.4030	0.0425	-1.4860	-1.3190		β_{6,age_9}	0.0910	0.0081	0.0748	0.1069
	$\beta_{4,\text{region}_8}$	-0.5663	0.0190	-0.6027	-0.5302		σ^2	0.0022	0.0010	0.0007	0.0045
	$\beta_{4,\text{region}_9}$	-0.6240	0.0206	-0.6630	-0.5838						

S26 Table. Estimated coefficients for the best fitting model for female bowel cancer mortality.

Covariate	Parameter	Mean	SD	%2.5	%97.5	Covariate	Parameter	Mean	SD	%2.5	%97.5
Intercept	β_0	-7.7770	0.0046	-7.7860	-7.7680		$\beta_{4,\text{deprivation}_5}$	0.0236	0.0100	0.0049	0.0441
Age	β_{1,age_1}	-2.0380	0.0232	-2.0840	-1.9950		$\beta_{4,\text{deprivation}_6}$	-0.0212	0.0103	-0.0419	-0.0015
	β_{1,age_2}	-1.4290	0.0173	-1.4640	-1.3960		$\beta_{4,\text{deprivation}_7}$	-0.0169	0.0097	-0.0360	0.0019
	β_{1,age_3}	-0.8723	0.0140	-0.9005	-0.8446		$\beta_{4,\text{deprivation}_8}$	-0.0291	0.0102	-0.0495	-0.0092
	β_{1,age_4}	-0.4406	0.0120	-0.4633	-0.4163		$\beta_{4,\text{deprivation}_9}$	-0.0466	0.0101	-0.0667	-0.0268
	β_{1,age_5}	0.0018	0.0105	-0.0185	0.0230		$\beta_{4,\text{deprivation}_{10}}$	-0.1034	0.0111	-0.1250	-0.0823
	β_{1,age_6}	0.4368	0.0100	0.4173	0.4564	Age:Year	β_{5,age_1}	0.0640	0.0238	0.0192	0.1103
	β_{1,age_7}	0.8609	0.0089	0.8433	0.8785		β_{5,age_2}	0.0188	0.0166	-0.0145	0.0502
	β_{1,age_8}	1.2780	0.0083	1.2620	1.2950		β_{5,age_3}	0.0062	0.0137	-0.0209	0.0328
	β_{1,age_9}	2.2010	0.0071	2.1880	2.2160		β_{5,age_4}	-0.0318	0.0128	-0.0577	-0.0061
Year	β_2	-0.0529	0.0047	-0.0623	-0.0439		β_{5,age_5}	-0.0439	0.0104	-0.0644	-0.0240
AAD	β_3	0.0359	0.0156	0.0058	0.0654		β_{5,age_6}	-0.0587	0.0096	-0.0772	-0.0399
Deprivation	$\beta_{4,\text{deprivation}_1}$	0.0793	0.0121	0.0560	0.1035		β_{5,age_7}	-0.0338	0.0091	-0.0513	-0.0158
	$\beta_{4,\text{deprivation}_2}$	0.0557	0.0108	0.0350	0.0771		β_{5,age_8}	0.0177	0.0081	0.0022	0.0338
	$\beta_{4,\text{deprivation}_3}$	0.0321	0.0103	0.0120	0.0520		β_{5,age_9}	0.0613	0.0074	0.0473	0.0758
	$\beta_{4,\text{deprivation}_4}$	0.0266	0.0099	0.0072	0.0460		σ^2	0.0059	0.0014	0.0027	0.0084

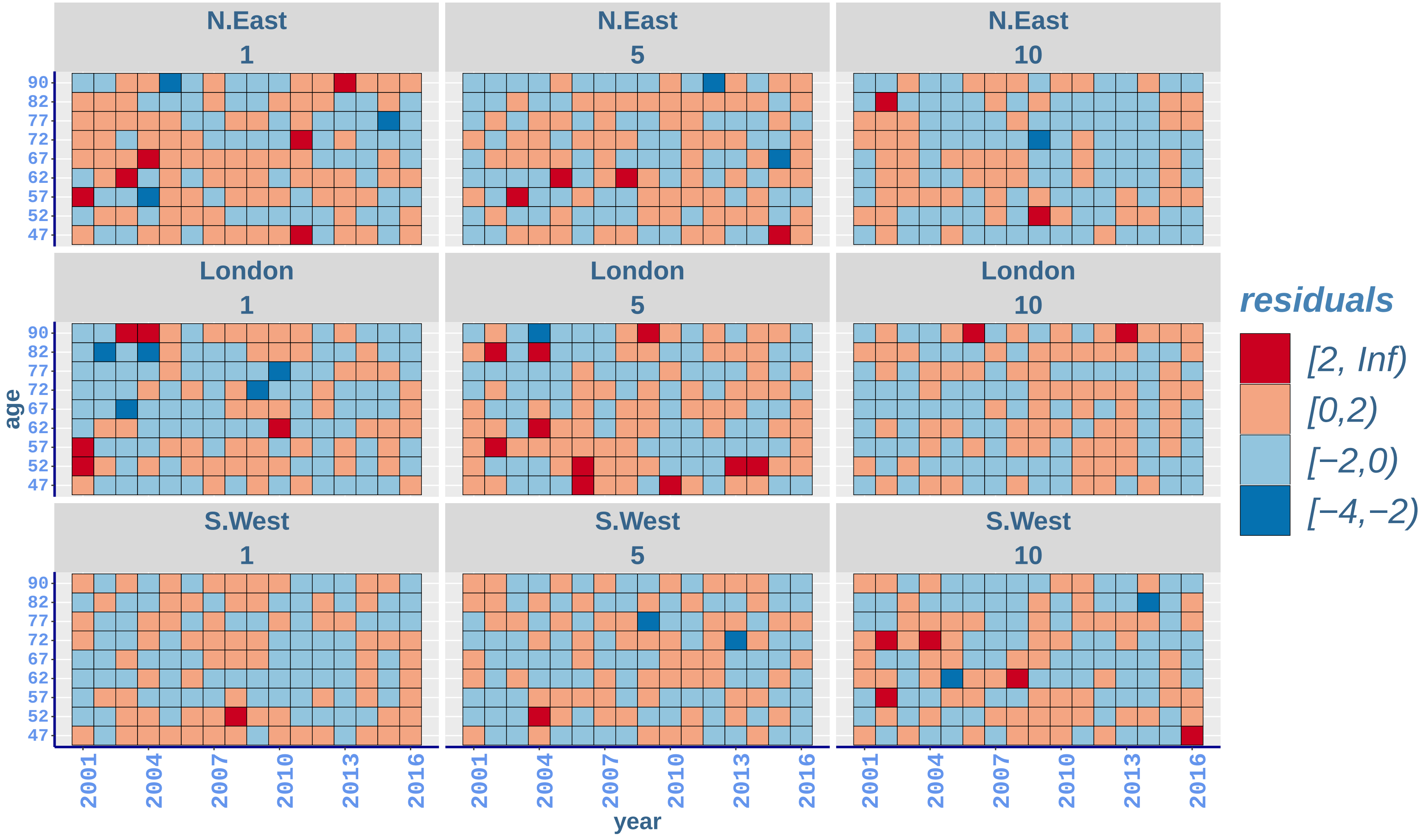

S57 Fig. Heat map of Pearson residuals for bowel cancer morbidity for males in North East, London, and South West, deprivation deciles 1, 5, and 10: orange/light blue cells indicate areas with good fit, while red/dark blue cells indicate areas with poor fit. Note that there is a small number of residuals greater than 4, and these are included in the last category.

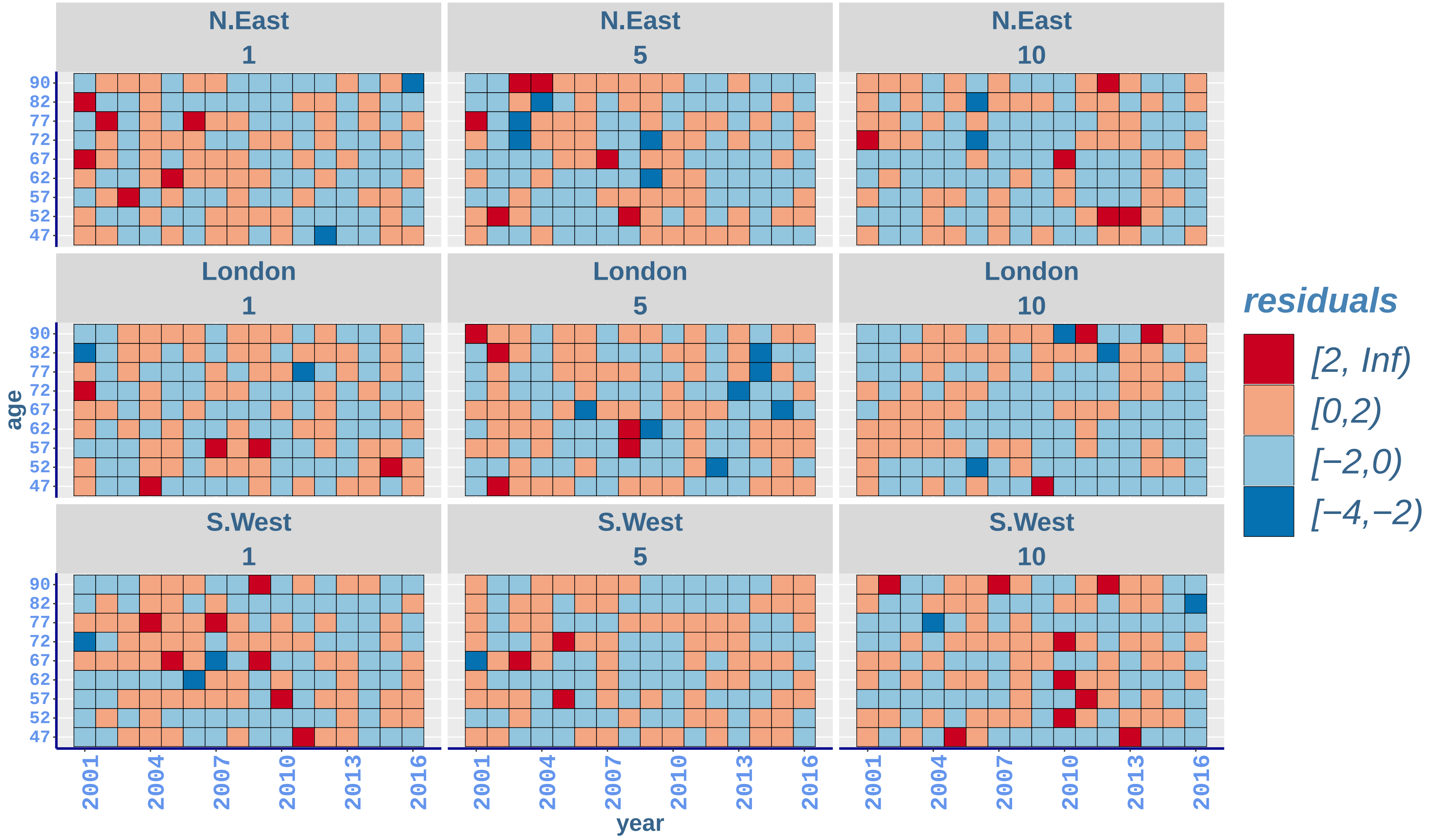

S58 Fig. Heat map of Pearson residuals for bowel cancer morbidity for females in North East, London, and South West, deprivation deciles 1, 5, and 10: orange/light blue cells indicate areas with good fit, while red/dark blue cells indicate areas with poor fit. Note that there is a small number of residuals greater than 4, and these are included in the last category.

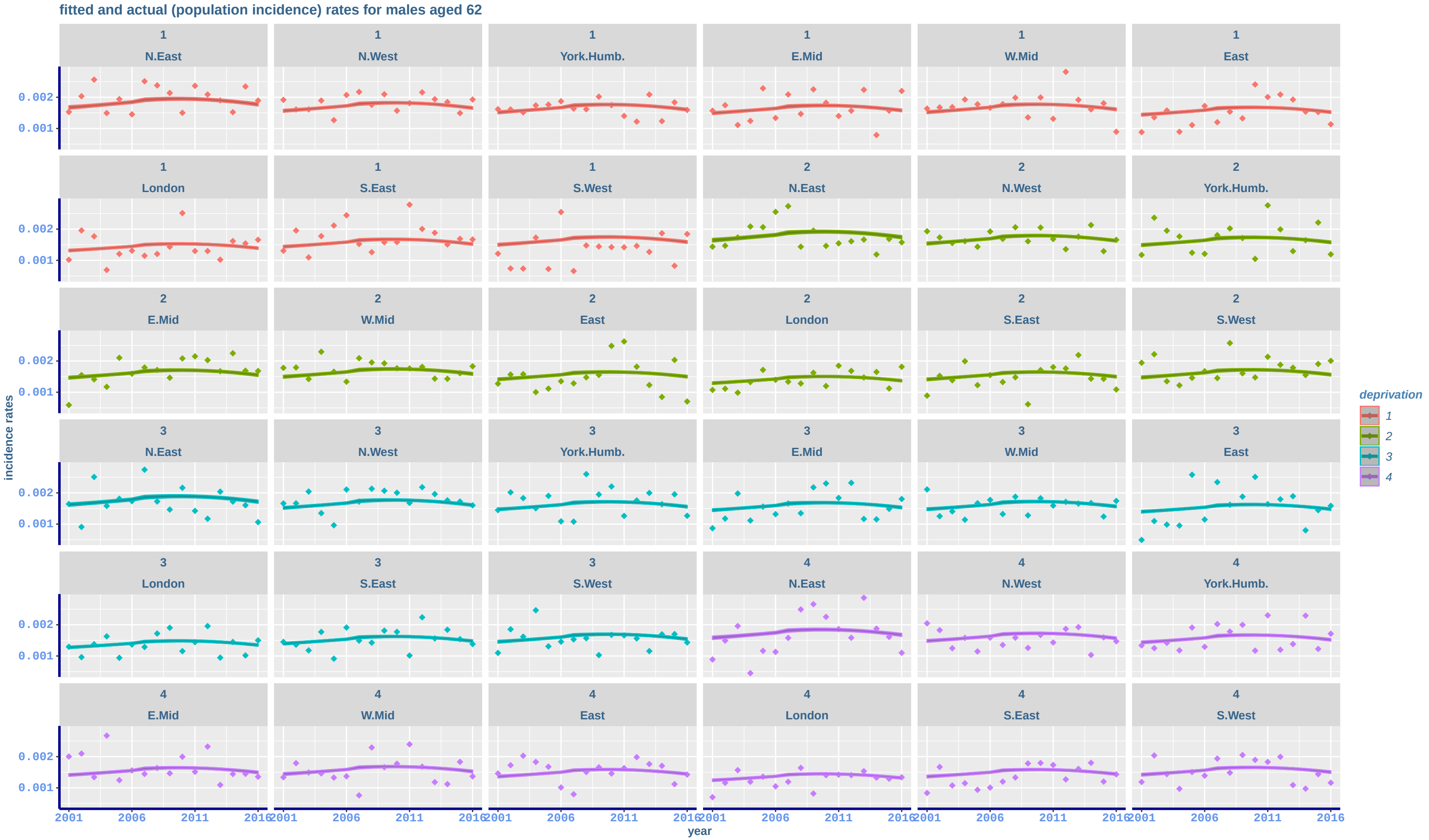

S59 Fig. Bowel cancer morbidity for males, age 62, deprivation deciles 1-4 for all regions in England between 2001 and 2016: observed rates (dots), fitted rates (lines), with 95% credible intervals for the fitted rates.

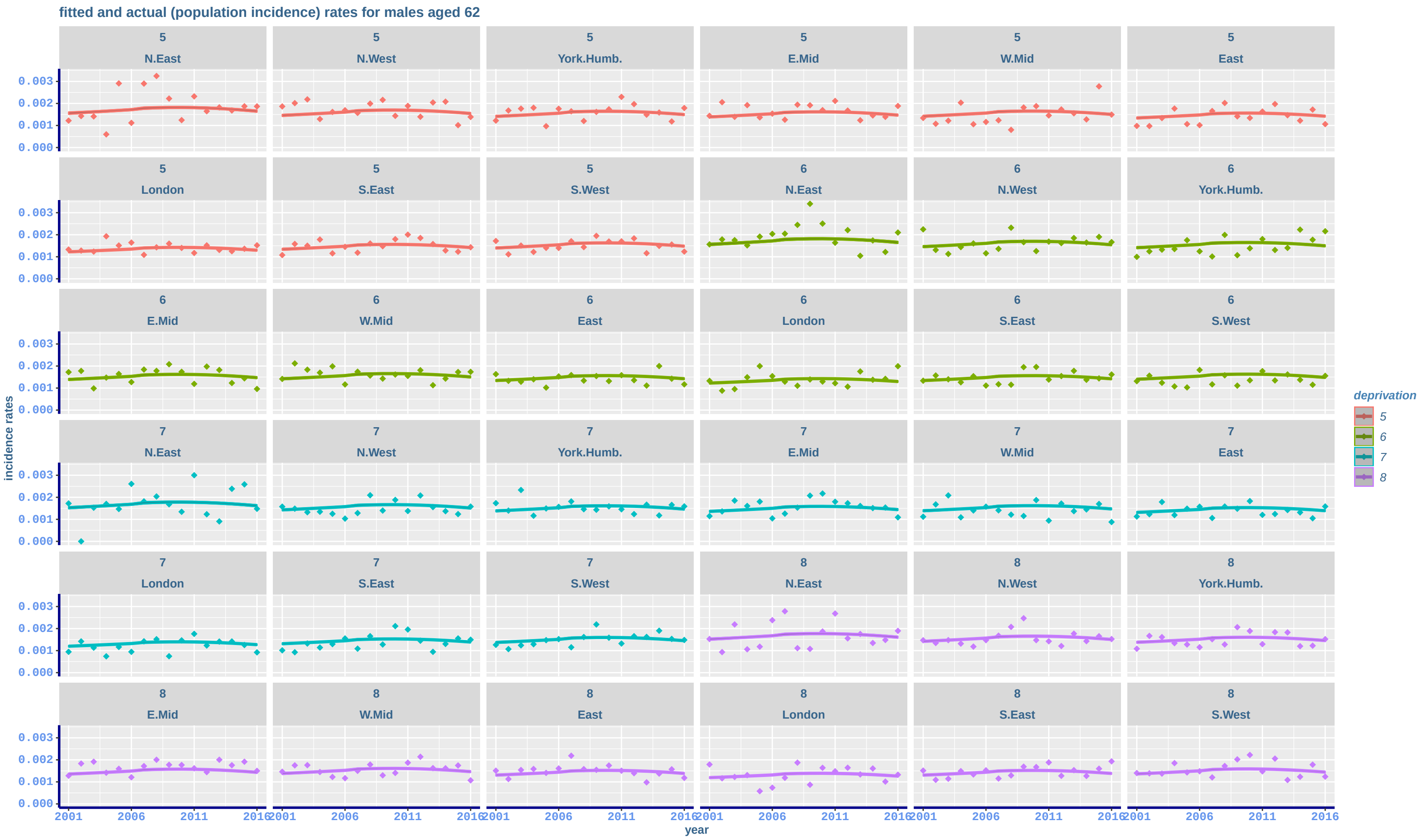

S60 Fig. Bowel cancer morbidity for males, age 62, deprivation deciles 5-8 for all regions in England between 2001 and 2016: observed rates (dots), fitted rates (lines), with 95% credible intervals for the fitted rates.

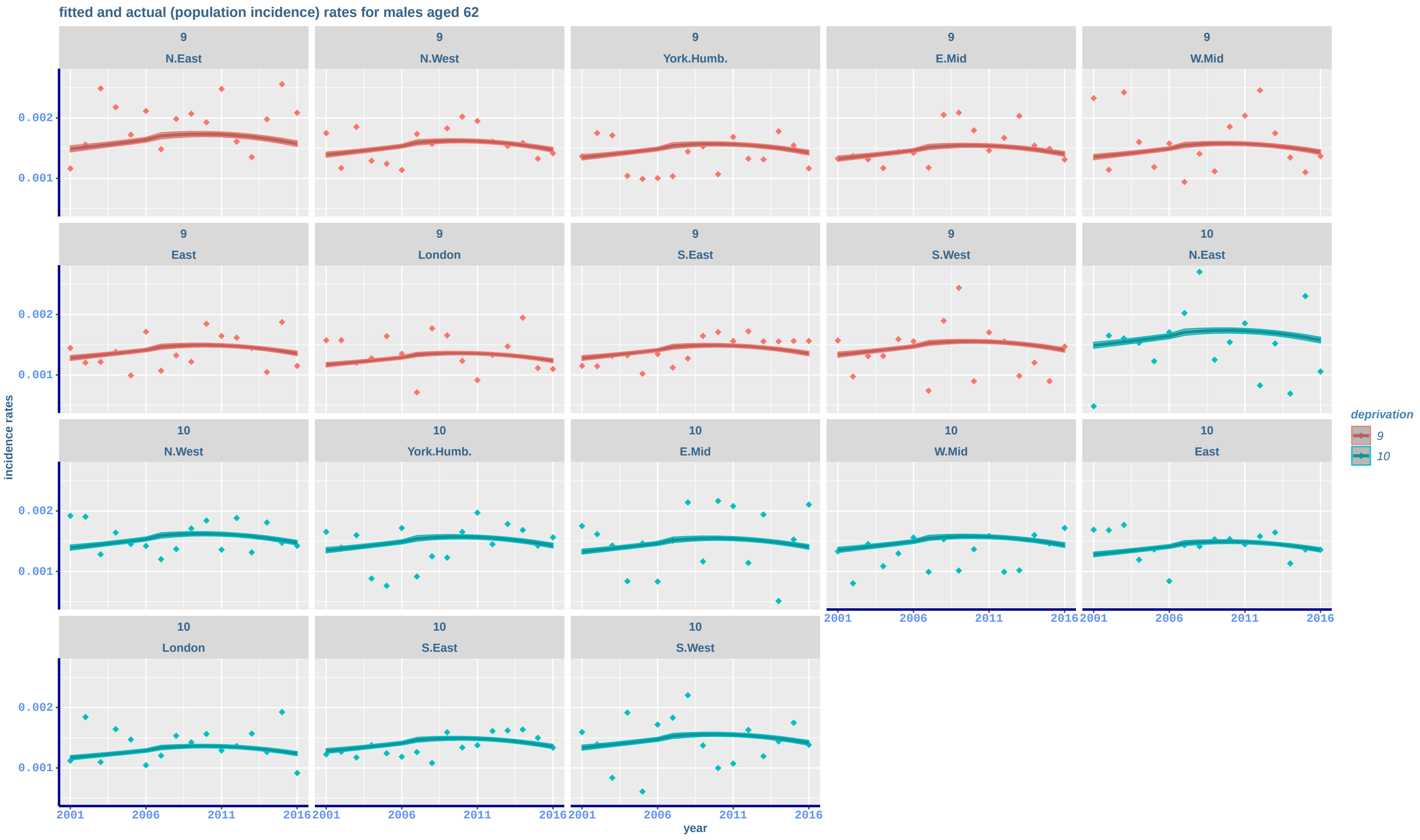

S61 Fig. Bowel cancer morbidity for males, age 62, deprivation deciles 9-10 for all regions in England between 2001 and 2016: observed rates (dots), fitted rates (lines), with 95% credible intervals for the fitted rates.

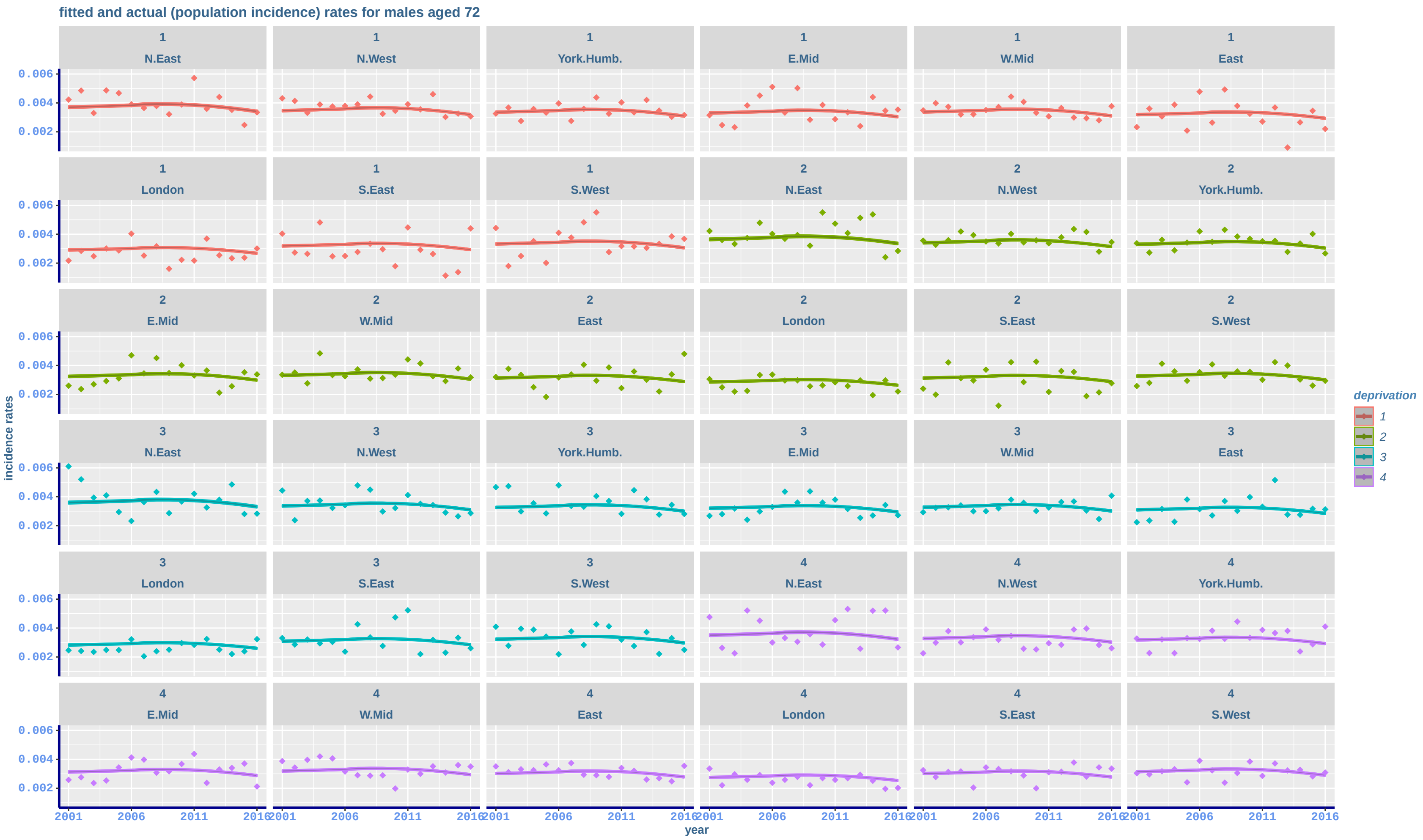

S62 Fig. Bowel cancer morbidity for males, age 72, deprivation deciles 1-4 for all regions in England between 2001 and 2016: observed rates (dots), fitted rates (lines), with 95% credible intervals for the fitted rates.

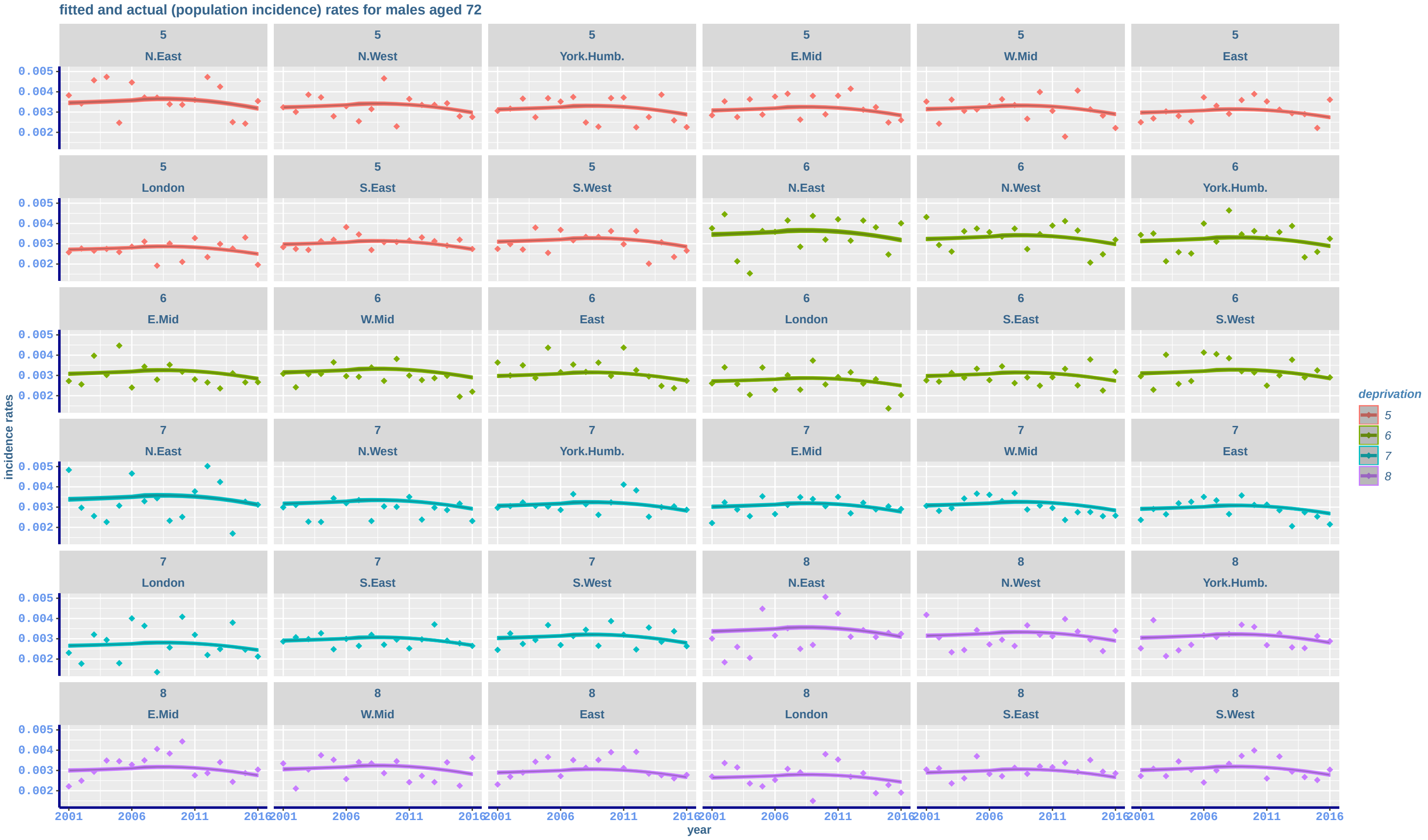

S63 Fig. Bowel cancer morbidity for males, age 72, deprivation deciles 5-8 for all regions in England between 2001 and 2016: observed rates (dots), fitted rates (lines), with 95% credible intervals for the fitted rates.

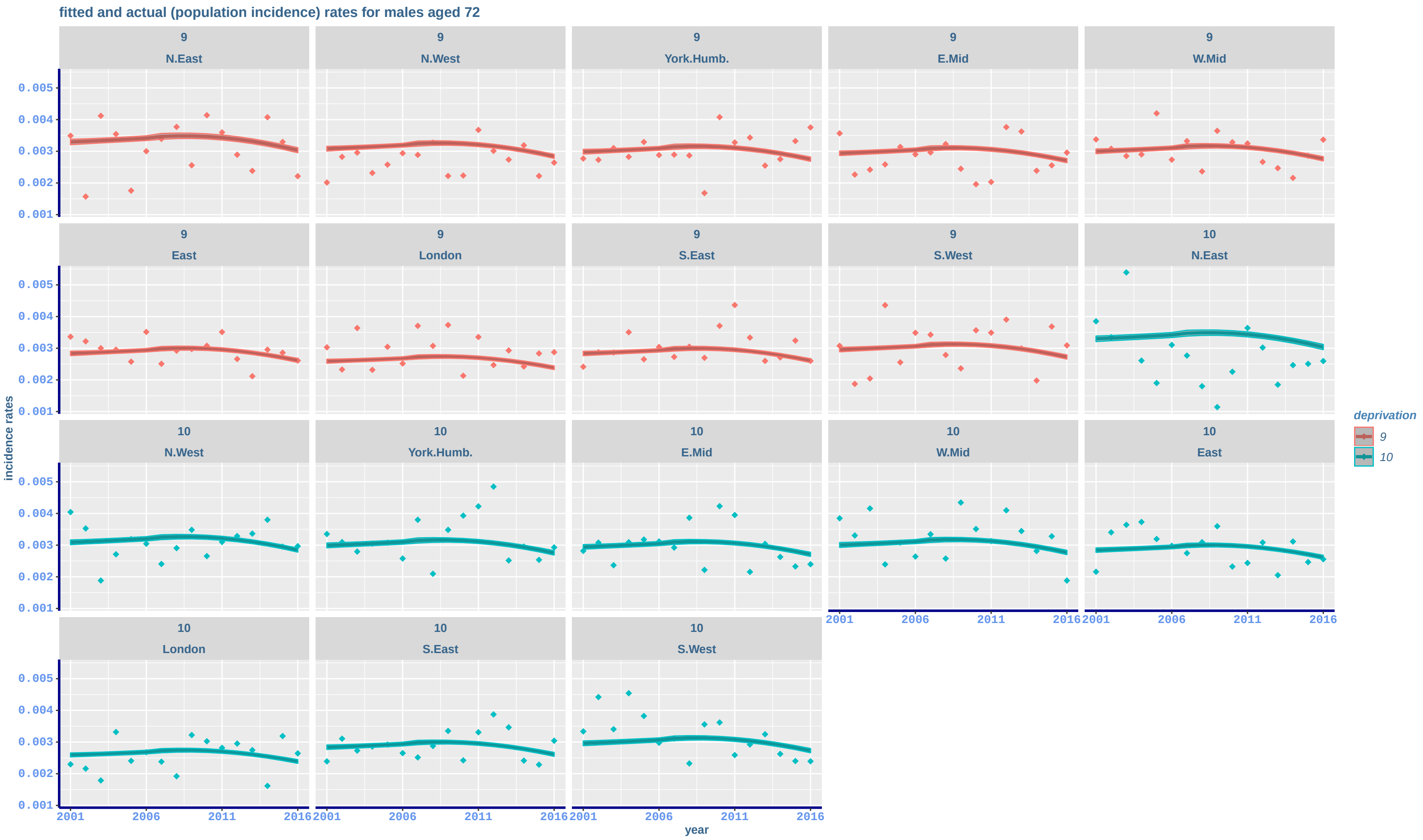

S64 Fig. Bowel cancer morbidity for males, age 72, deprivation deciles 9-10 for all regions in England between 2001 and 2016: observed rates (dots), fitted rates (lines), with 95% credible intervals for the fitted rates.

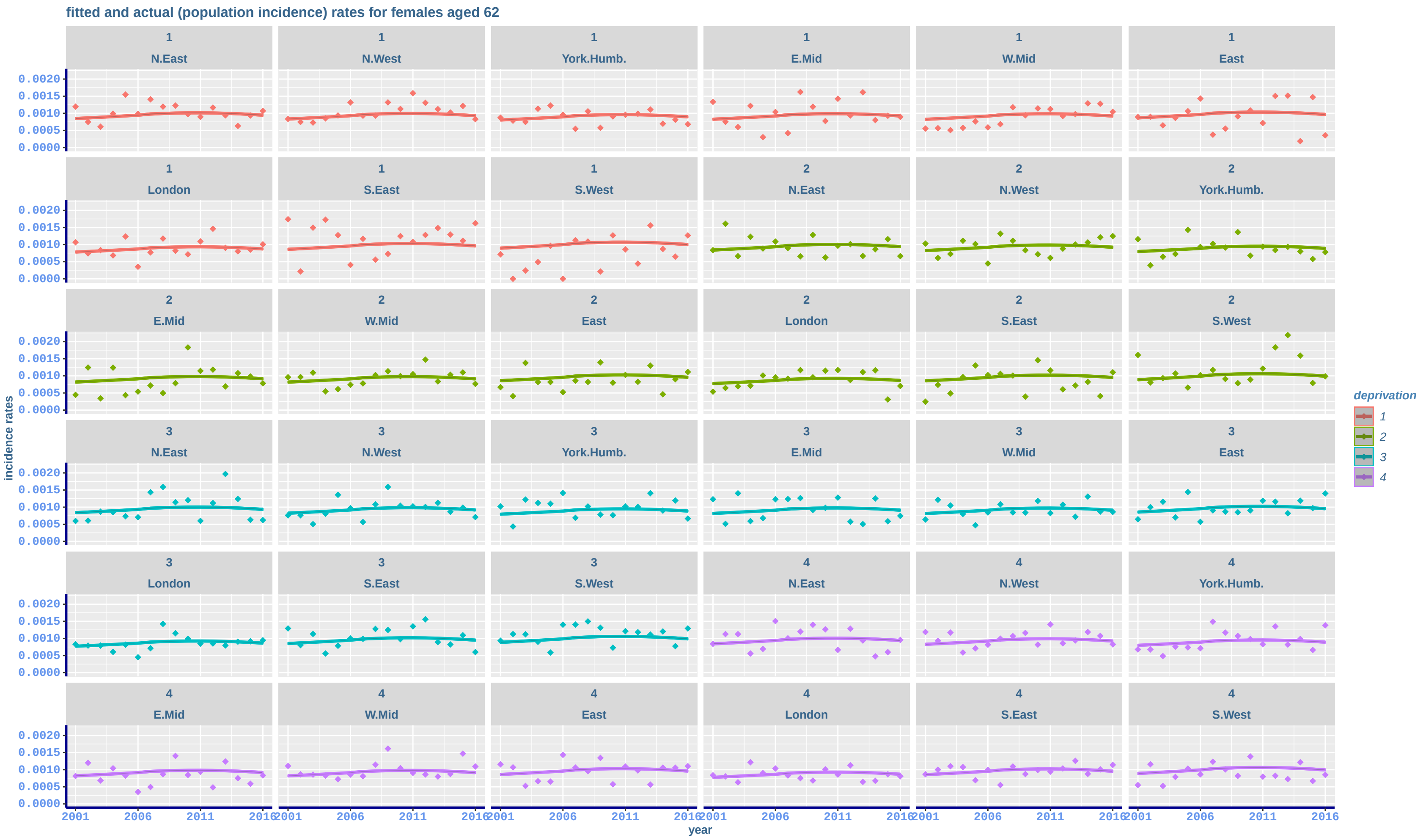

S65 Fig. Bowel cancer morbidity for females, age 62, deprivation deciles 1-4 for all regions in England between 2001 and 2016: observed rates (dots), fitted rates (lines), with 95% credible intervals for the fitted rates.

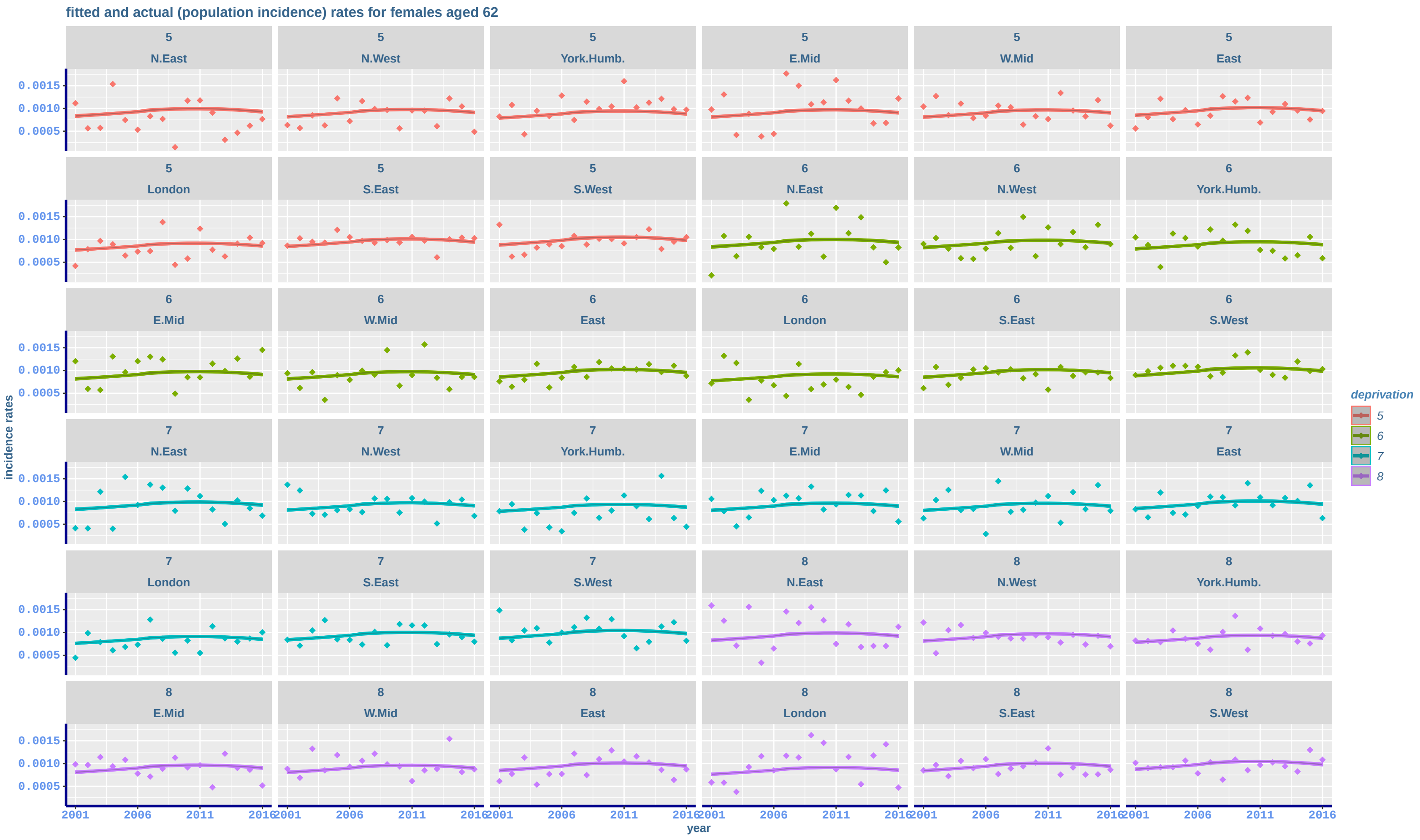

S66 Fig. Bowel cancer morbidity for females, age 62, deprivation deciles 5-8 for all regions in England between 2001 and 2016: observed rates (dots), fitted rates (lines), with 95% credible intervals for the fitted rates.

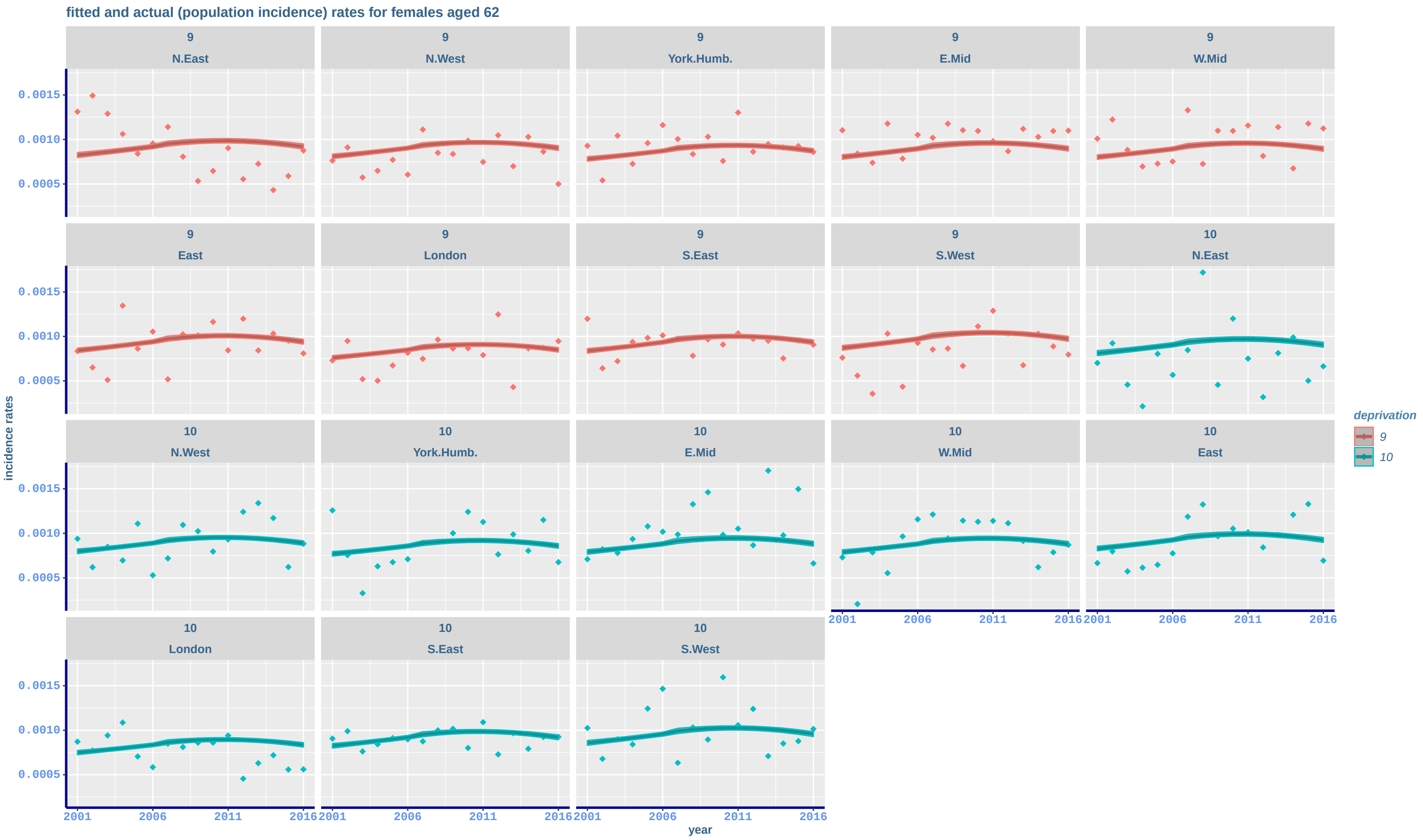

S67 Fig. Bowel cancer morbidity for females, age 62, deprivation deciles 9-10 for all regions in England between 2001 and 2016: observed rates (dots), fitted rates (lines), with 95% credible intervals for the fitted rates.

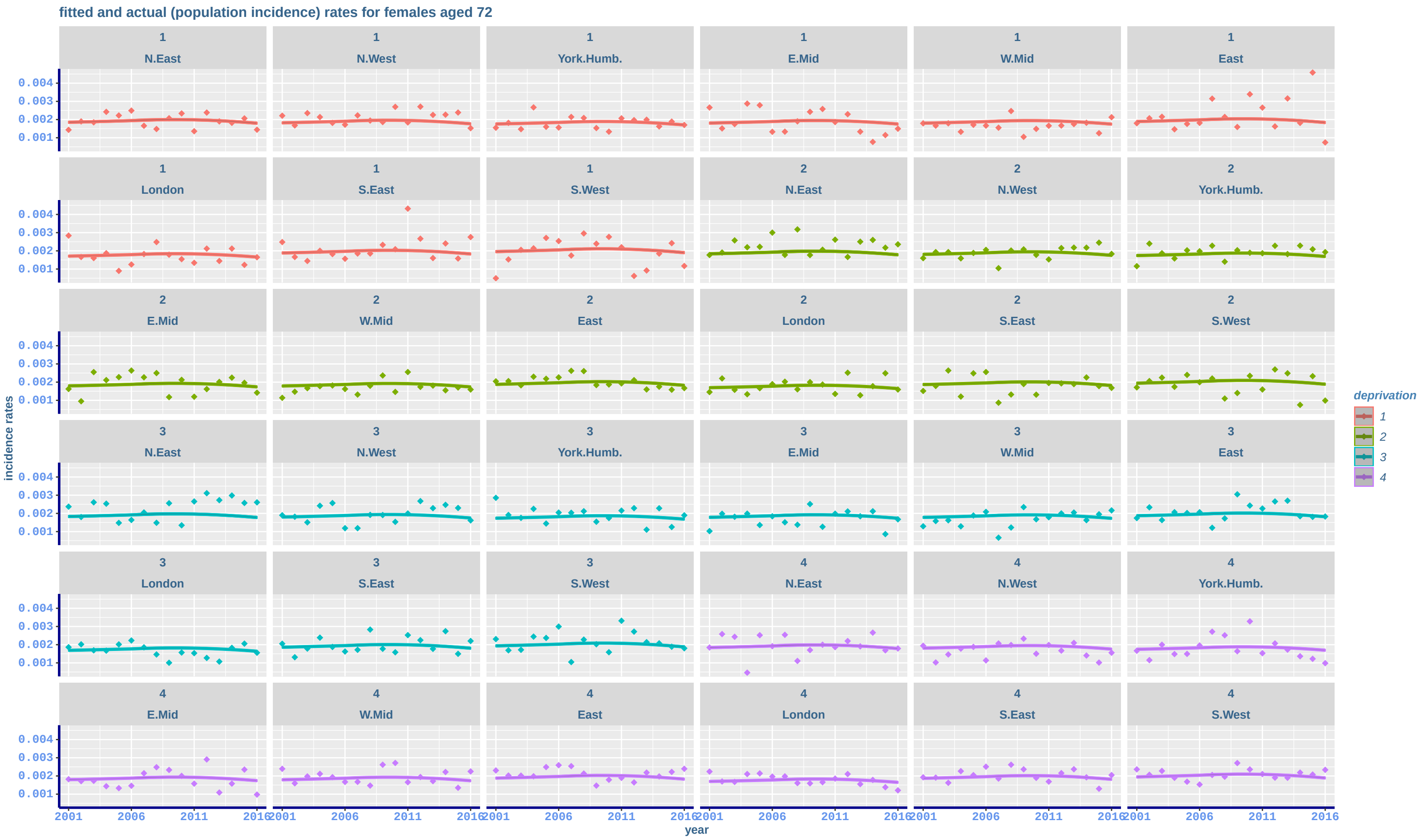

S68 Fig. Bowel cancer morbidity for females, age 72, deprivation deciles 1-4 for all regions in England between 2001 and 2016: observed rates (dots), fitted rates (lines), with 95% credible intervals for the fitted rates.

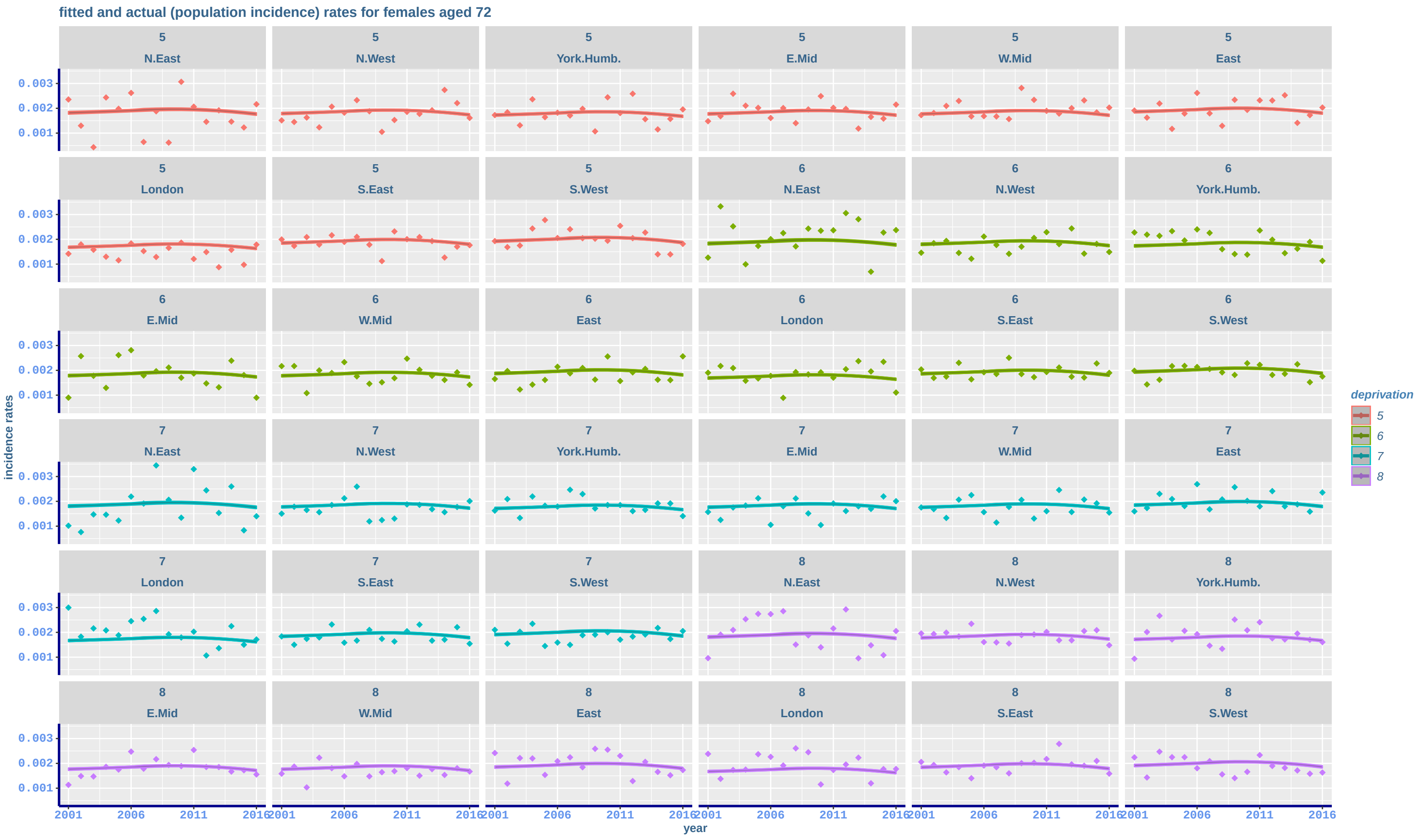

S69 Fig. Bowel cancer morbidity for females, age 72, deprivation deciles 5-8 for all regions in England between 2001 and 2016: observed rates (dots), fitted rates (lines), with 95% credible intervals for the fitted rates.

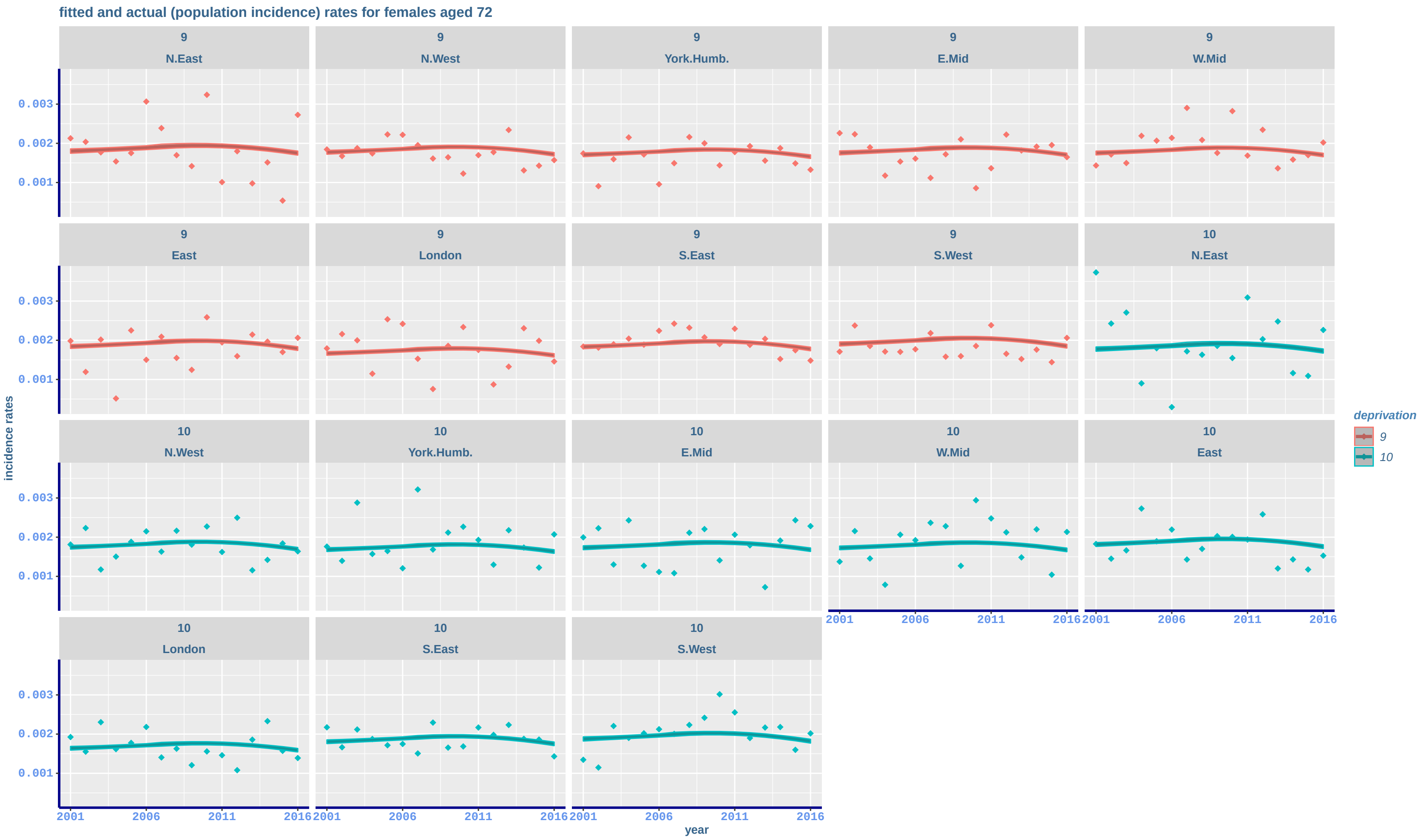

S70 Fig. Bowel cancer morbidity for females, age 72, deprivation deciles 9-10 for all regions in England between 2001 and 2016: observed rates (dots), fitted rates (lines), with 95% credible intervals for the fitted rates.

S27 Table. Absolute deprivation differences ($\mathbf{AD}_{t,r}$), per 100,000 people, in age-standardised fitted incidence rates of bowel cancer from 2001 to 2016 for all regions in England for males; 95% credible intervals in brackets

year	N.East	N.West	York.Humb.	E.Mid	W.Mid	East	London	S.East	S.West
2001	27 (23, 31)	25 (22, 29)	25 (21, 28)	24 (21, 28)	25 (21, 28)	23 (20, 27)	21 (18, 25)	23 (20, 27)	24 (21, 28)
2002	27 (23, 31)	26 (22, 29)	25 (21, 28)	24 (21, 28)	25 (21, 29)	24 (20, 27)	21 (18, 25)	23 (20, 27)	24 (21, 28)
2003	27 (24, 32)	26 (22, 30)	25 (21, 29)	24 (21, 28)	25 (21, 29)	24 (20, 27)	22 (18, 25)	24 (20, 27)	25 (21, 28)
2004	28 (24, 32)	26 (22, 30)	25 (22, 29)	25 (21, 28)	25 (22, 29)	24 (20, 27)	22 (19, 25)	24 (20, 27)	25 (21, 29)
2005	28 (24, 32)	26 (22, 30)	25 (22, 29)	25 (21, 29)	25 (22, 29)	24 (21, 28)	22 (19, 25)	24 (21, 28)	25 (21, 29)
2006	28 (24, 32)	26 (23, 30)	25 (22, 29)	25 (21, 29)	26 (22, 29)	24 (21, 28)	22 (19, 25)	24 (21, 28)	25 (22, 29)
2007	28 (24, 33)	27 (23, 31)	26 (22, 30)	25 (22, 29)	26 (22, 30)	24 (21, 28)	22 (19, 26)	24 (21, 28)	25 (22, 29)
2008	29 (25, 33)	27 (23, 31)	26 (22, 30)	26 (22, 29)	26 (22, 30)	25 (21, 28)	23 (19, 26)	25 (21, 28)	26 (22, 30)
2009	29 (25, 33)	27 (23, 31)	26 (22, 30)	26 (22, 30)	26 (23, 30)	25 (21, 28)	23 (19, 26)	25 (21, 28)	26 (22, 30)
2010	29 (25, 33)	27 (23, 31)	26 (22, 30)	26 (22, 30)	26 (22, 30)	25 (21, 28)	23 (19, 26)	25 (21, 28)	26 (22, 30)
2011	29 (25, 33)	27 (23, 31)	26 (22, 30)	25 (22, 29)	26 (22, 30)	25 (21, 28)	22 (19, 26)	25 (21, 28)	26 (22, 30)
2012	28 (24, 32)	26 (23, 30)	26 (22, 30)	25 (22, 29)	26 (22, 30)	24 (21, 28)	22 (19, 26)	24 (21, 28)	25 (22, 29)
2013	28 (24, 32)	26 (22, 30)	25 (22, 29)	25 (21, 29)	25 (22, 29)	24 (21, 28)	22 (19, 25)	24 (21, 28)	25 (21, 29)
2014	27 (23, 31)	26 (22, 29)	25 (21, 29)	24 (21, 28)	25 (21, 29)	24 (20, 27)	21 (18, 25)	23 (20, 27)	25 (21, 28)
2015	27 (23, 31)	25 (21, 29)	24 (21, 28)	24 (20, 27)	24 (21, 28)	23 (20, 26)	21 (18, 24)	23 (20, 26)	24 (20, 28)
2016	26 (22, 30)	24 (21, 28)	23 (20, 27)	23 (20, 27)	24 (20, 27)	22 (19, 26)	20 (17, 23)	22 (19, 26)	23 (20, 27)

S28 Table. Relative deprivation differences ($\mathrm{RD}_{t,r}$) in age-standardised fitted incidence rates of bowel cancer from 2001 to 2016 for all regions in England for males; 95% credible intervals in brackets

year	N.East	N.West	York.Humb.	E.Mid	W.Mid	East	London	S.East	S.West
2001	0.1275 (0.1085, 0.1475)	0.1275 (0.1085, 0.1474)	0.1275 (0.1085, 0.1475)	0.1275 (0.1085, 0.1475)	0.1275 (0.1085, 0.1475)	0.1275 (0.1085, 0.1475)	0.1275 (0.1085, 0.1474)	0.1275 (0.1085, 0.1475)	0.1275 (0.1085, 0.1474)
2002	0.1275 (0.1086, 0.1474)	0.1275 (0.1085, 0.1475)	0.1275 (0.1085, 0.1475)	0.1275 (0.1085, 0.1474)	0.1275 (0.1085, 0.1475)	0.1275 (0.1085, 0.1474)	0.1275 (0.1085, 0.1475)	0.1275 (0.1085, 0.1475)	0.1275 (0.1085, 0.1475)
2003	0.1275 (0.1085, 0.1474)	0.1275 (0.1085, 0.1475)	0.1275 (0.1085, 0.1474)	0.1275 (0.1085, 0.1475)	0.1275 (0.1085, 0.1475)	0.1275 (0.1086, 0.1475)	0.1275 (0.1085, 0.1474)	0.1275 (0.1085, 0.1475)	0.1275 (0.1085, 0.1474)
2004	0.1275 (0.1086, 0.1475)	0.1275 (0.1084, 0.1475)	0.1275 (0.1085, 0.1475)	0.1275 (0.1085, 0.1475)	0.1275 (0.1085, 0.1475)	0.1275 (0.1085, 0.1475)	0.1275 (0.1085, 0.1475)	0.1275 (0.1086, 0.1475)	0.1275 (0.1085, 0.1475)
2005	0.1275 (0.1085, 0.1475)	0.1275 (0.1085, 0.1475)	0.1275 (0.1086, 0.1475)	0.1275 (0.1085, 0.1475)	0.1275 (0.1085, 0.1475)	0.1275 (0.1085, 0.1474)	0.1275 (0.1085, 0.1474)	0.1275 (0.1085, 0.1475)	0.1275 (0.1085, 0.1475)
2006	0.1275 (0.1086, 0.1475)	0.1275 (0.1086, 0.1475)	0.1275 (0.1084, 0.1474)	0.1275 (0.1085, 0.1475)	0.1275 (0.1085, 0.1475)	0.1275 (0.1085, 0.1474)	0.1275 (0.1086, 0.1474)	0.1275 (0.1085, 0.1475)	0.1275 (0.1085, 0.1474)
2007	0.1275 (0.1085, 0.1475)	0.1275 (0.1085, 0.1474)	0.1275 (0.1085, 0.1474)	0.1275 (0.1085, 0.1475)	0.1275 (0.1085, 0.1475)	0.1275 (0.1086, 0.1475)	0.1275 (0.1085, 0.1475)	0.1275 (0.1085, 0.1474)	0.1275 (0.1085, 0.1474)
2008	0.1275 (0.1086, 0.1474)	0.1275 (0.1086, 0.1474)	0.1275 (0.1085, 0.1475)	0.1275 (0.1085, 0.1475)	0.1275 (0.1085, 0.1474)	0.1275 (0.1085, 0.1474)	0.1275 (0.1085, 0.1475)	0.1275 (0.1085, 0.1474)	0.1275 (0.1085, 0.1475)
2009	0.1275 (0.1085, 0.1475)	0.1275 (0.1085, 0.1475)	0.1275 (0.1085, 0.1475)	0.1275 (0.1085, 0.1475)	0.1275 (0.1085, 0.1475)	0.1275 (0.1085, 0.1474)	0.1275 (0.1085, 0.1474)	0.1275 (0.1085, 0.1475)	0.1275 (0.1085, 0.1474)
2010	0.1275 (0.1085, 0.1474)	0.1275 (0.1085, 0.1475)	0.1275 (0.1085, 0.1475)	0.1275 (0.1085, 0.1475)	0.1275 (0.1085, 0.1475)	0.1275 (0.1085, 0.1475)	0.1275 (0.1085, 0.1475)	0.1275 (0.1085, 0.1474)	0.1275 (0.1086, 0.1475)
2011	0.1275 (0.1085, 0.1474)	0.1275 (0.1086, 0.1475)	0.1275 (0.1085, 0.1475)	0.1275 (0.1085, 0.1475)	0.1275 (0.1085, 0.1475)	0.1275 (0.1085, 0.1475)	0.1275 (0.1085, 0.1474)	0.1275 (0.1085, 0.1475)	0.1275 (0.1085, 0.1475)
2012	0.1275 (0.1085, 0.1475)	0.1275 (0.1085, 0.1474)	0.1275 (0.1084, 0.1475)	0.1275 (0.1085, 0.1474)	0.1275 (0.1085, 0.1475)	0.1275 (0.1085, 0.1475)	0.1275 (0.1085, 0.1475)	0.1275 (0.1085, 0.1475)	0.1275 (0.1085, 0.1474)
2013	0.1275 (0.1086, 0.1474)	0.1275 (0.1085, 0.1475)	0.1275 (0.1085, 0.1475)	0.1275 (0.1085, 0.1475)	0.1275 (0.1086, 0.1475)	0.1275 (0.1085, 0.1475)	0.1275 (0.1085, 0.1474)	0.1275 (0.1085, 0.1475)	0.1275 (0.1085, 0.1475)
2014	0.1275 (0.1085, 0.1475)	0.1275 (0.1085, 0.1475)	0.1275 (0.1085, 0.1475)	0.1275 (0.1085, 0.1475)	0.1275 (0.1086, 0.1475)	0.1275 (0.1085, 0.1475)	0.1275 (0.1086, 0.1475)	0.1275 (0.1085, 0.1474)	0.1275 (0.1085, 0.1475)
2015	0.1275 (0.1086, 0.1474)	0.1275 (0.1085, 0.1475)	0.1275 (0.1085, 0.1474)	0.1275 (0.1086, 0.1474)	0.1275 (0.1085, 0.1474)	0.1275 (0.1085, 0.1474)	0.1275 (0.1085, 0.1475)	0.1275 (0.1085, 0.1475)	0.1275 (0.1086, 0.1474)
2016	0.1275 (0.1085, 0.1475)	0.1275 (0.1085, 0.1475)	0.1275 (0.1086, 0.1475)	0.1275 (0.1086, 0.1475)	0.1275 (0.1085, 0.1474)	0.1275 (0.1086, 0.1475)	0.1275 (0.1085, 0.1475)	0.1275 (0.1086, 0.1475)	0.1275 (0.1086, 0.1475)

S29 Table. Absolute deprivation differences ($AD_{t,r}$), per 100,000 people, in age-standardised fitted incidence rates of bowel cancer from 2001 to 2016 for all regions in England for females; 95% credible intervals in brackets

year	N.East	N.West	York.Humb.	E.Mid	W.Mid	East	London	S.East	S.West
2001	6 (4, 8)	6 (4, 8)	6 (4, 8)	6 (4, 8)	6 (4, 8)	6 (4, 9)	6 (4, 8)	6 (4, 9)	6 (4, 9)
2002	6 (4, 8)	6 (4, 8)	6 (4, 8)	6 (4, 8)	6 (4, 8)	6 (4, 9)	6 (4, 8)	6 (4, 9)	6 (4, 9)
2003	6 (4, 9)	6 (4, 8)	6 (4, 8)	6 (4, 8)	6 (4, 8)	6 (4, 9)	6 (4, 8)	6 (4, 9)	6 (4, 9)
2004	6 (4, 9)	6 (4, 9)	6 (4, 8)	6 (4, 8)	6 (4, 8)	6 (4, 9)	6 (4, 8)	6 (4, 9)	7 (4, 9)
2005	6 (4, 9)	6 (4, 9)	6 (4, 8)	6 (4, 9)	6 (4, 9)	6 (4, 9)	6 (4, 8)	6 (4, 9)	7 (4, 9)
2006	6 (4, 9)	6 (4, 9)	6 (4, 8)	6 (4, 9)	6 (4, 9)	6 (4, 9)	6 (4, 8)	6 (4, 9)	7 (4, 9)
2007	6 (4, 9)	6 (4, 9)	6 (4, 8)	6 (4, 9)	6 (4, 9)	6 (4, 9)	6 (4, 8)	6 (4, 9)	7 (4, 9)
2008	6 (4, 9)	6 (4, 9)	6 (4, 9)	6 (4, 9)	6 (4, 9)	7 (4, 9)	6 (4, 8)	7 (4, 9)	7 (4, 10)
2009	6 (4, 9)	6 (4, 9)	6 (4, 9)	6 (4, 9)	6 (4, 9)	7 (4, 9)	6 (4, 8)	7 (4, 9)	7 (4, 10)
2010	6 (4, 9)	6 (4, 9)	6 (4, 9)	6 (4, 9)	6 (4, 9)	7 (4, 9)	6 (4, 8)	7 (4, 9)	7 (4, 10)
2011	6 (4, 9)	6 (4, 9)	6 (4, 9)	6 (4, 9)	6 (4, 9)	7 (4, 9)	6 (4, 8)	7 (4, 9)	7 (4, 10)
2012	6 (4, 9)	6 (4, 9)	6 (4, 9)	6 (4, 9)	6 (4, 9)	7 (4, 9)	6 (4, 8)	7 (4, 9)	7 (4, 10)
2013	6 (4, 9)	6 (4, 9)	6 (4, 9)	6 (4, 9)	6 (4, 9)	7 (4, 9)	6 (4, 8)	7 (4, 9)	7 (4, 10)
2014	6 (4, 9)	6 (4, 9)	6 (4, 8)	6 (4, 9)	6 (4, 9)	6 (4, 9)	6 (4, 8)	6 (4, 9)	7 (4, 9)
2015	6 (4, 9)	6 (4, 9)	6 (4, 8)	6 (4, 9)	6 (4, 8)	6 (4, 9)	6 (4, 8)	6 (4, 9)	7 (4, 9)
2016	6 (4, 9)	6 (4, 8)	6 (4, 8)	6 (4, 8)	6 (4, 8)	6 (4, 9)	6 (4, 8)	6 (4, 9)	6 (4, 9)

S30 Table. Relative deprivation differences ($AD_{t,r}$) in age-standardised fitted incidence rates of bowel cancer from 2001 to 2016 for all regions in England for females; 95% credible intervals in brackets

year	N.East	N.West	York.Humb.	E.Mid	W.Mid	East	London	S.East	S.West
2001	0.0477<br>(0.0307, 0.0679)	0.0477<br>(0.0308, 0.068)	0.0477<br>(0.0307, 0.068)	0.0477<br>(0.0308, 0.0681)	0.0477<br>(0.0308, 0.0679)	0.0477<br>(0.0307, 0.068)	0.0477<br>(0.0307, 0.068)	0.0477<br>(0.0307, 0.0679)	0.0477<br>(0.0309, 0.068)
2002	0.0477<br>(0.0307, 0.0679)	0.0477<br>(0.0309, 0.068)	0.0477<br>(0.0307, 0.068)	0.0477<br>(0.0308, 0.0679)	0.0477<br>(0.0307, 0.0679)	0.0477<br>(0.0308, 0.0679)	0.0477<br>(0.0309, 0.0679)	0.0477<br>(0.0307, 0.068)	0.0477<br>(0.0309, 0.0679)
2003	0.0477<br>(0.0308, 0.0679)	0.0477<br>(0.0308, 0.068)	0.0477<br>(0.0309, 0.068)	0.0477<br>(0.0308, 0.0679)	0.0477<br>(0.0308, 0.0679)	0.0477<br>(0.0308, 0.068)	0.0477<br>(0.0308, 0.068)	0.0477<br>(0.0308, 0.0679)	0.0477<br>(0.0308, 0.068)
2004	0.0477<br>(0.0307, 0.0679)	0.0477<br>(0.0307, 0.0679)	0.0477<br>(0.0307, 0.068)	0.0477<br>(0.0308, 0.068)	0.0477<br>(0.0307, 0.0679)	0.0477<br>(0.0307, 0.068)	0.0477<br>(0.0308, 0.0679)	0.0477<br>(0.0308, 0.0679)	0.0477<br>(0.0308, 0.068)
2005	0.0477<br>(0.0309, 0.068)	0.0477<br>(0.0309, 0.068)	0.0477<br>(0.0308, 0.0679)	0.0477<br>(0.0307, 0.068)	0.0477<br>(0.0307, 0.068)	0.0477<br>(0.0307, 0.068)	0.0477<br>(0.0308, 0.0681)	0.0477<br>(0.0308, 0.068)	0.0477<br>(0.0307, 0.068)
2006	0.0477<br>(0.0308, 0.0679)	0.0477<br>(0.0308, 0.0679)	0.0477<br>(0.0308, 0.0679)	0.0477<br>(0.0307, 0.0679)	0.0477<br>(0.0309, 0.068)	0.0477<br>(0.0307, 0.068)	0.0477<br>(0.0307, 0.0679)	0.0477<br>(0.0308, 0.068)	0.0477<br>(0.0307, 0.068)
2007	0.0477<br>(0.0308, 0.068)	0.0477<br>(0.0308, 0.068)	0.0477<br>(0.0308, 0.0679)	0.0477<br>(0.0308, 0.0679)	0.0477<br>(0.0307, 0.0679)	0.0477<br>(0.0308, 0.068)	0.0477<br>(0.0307, 0.0681)	0.0477<br>(0.0307, 0.0679)	0.0477<br>(0.0308, 0.068)
2008	0.0477<br>(0.0307, 0.068)	0.0477<br>(0.0308, 0.0679)	0.0477<br>(0.0307, 0.068)	0.0477<br>(0.0308, 0.0679)	0.0477<br>(0.0308, 0.068)	0.0477<br>(0.0308, 0.068)	0.0477<br>(0.0308, 0.0679)	0.0477<br>(0.0308, 0.0679)	0.0477<br>(0.0308, 0.0679)
2009	0.0477<br>(0.0308, 0.068)	0.0477<br>(0.0307, 0.068)	0.0477<br>(0.0308, 0.068)	0.0477<br>(0.0308, 0.0678)	0.0477<br>(0.0307, 0.068)	0.0477<br>(0.0307, 0.0679)	0.0477<br>(0.0308, 0.068)	0.0477<br>(0.0309, 0.0679)	0.0477<br>(0.0307, 0.0679)
2010	0.0477<br>(0.0308, 0.0679)	0.0477<br>(0.0308, 0.0679)	0.0477<br>(0.0308, 0.068)	0.0477<br>(0.0308, 0.068)	0.0477<br>(0.0307, 0.0679)	0.0477<br>(0.0309, 0.0679)	0.0477<br>(0.0308, 0.068)	0.0477<br>(0.0307, 0.068)	0.0477<br>(0.0308, 0.068)
2011	0.0477<br>(0.0308, 0.068)	0.0477<br>(0.0308, 0.068)	0.0477<br>(0.0308, 0.068)	0.0477<br>(0.0308, 0.0679)	0.0477<br>(0.0308, 0.0679)	0.0477<br>(0.0307, 0.0679)	0.0477<br>(0.0308, 0.0679)	0.0477<br>(0.0308, 0.068)	0.0477<br>(0.0307, 0.068)
2012	0.0477<br>(0.0308, 0.0679)	0.0477<br>(0.0308, 0.068)	0.0477<br>(0.0308, 0.0679)	0.0477<br>(0.0307, 0.068)	0.0477<br>(0.0308, 0.068)	0.0477<br>(0.0308, 0.0679)	0.0477<br>(0.0307, 0.068)	0.0477<br>(0.0308, 0.068)	0.0477<br>(0.0308, 0.068)
2013	0.0477<br>(0.0309, 0.0678)	0.0477<br>(0.0307, 0.0679)	0.0477<br>(0.0308, 0.0679)	0.0477<br>(0.0307, 0.0679)	0.0477<br>(0.0307, 0.068)	0.0477<br>(0.0308, 0.068)	0.0477<br>(0.0307, 0.068)	0.0477<br>(0.0307, 0.068)	0.0477<br>(0.0308, 0.0679)
2014	0.0477<br>(0.0308, 0.068)	0.0477<br>(0.0308, 0.0679)	0.0477<br>(0.0308, 0.0679)	0.0477<br>(0.0308, 0.0679)	0.0477<br>(0.0307, 0.0679)	0.0477<br>(0.0308, 0.0679)	0.0477<br>(0.0308, 0.0679)	0.0477<br>(0.0308, 0.0679)	0.0477<br>(0.0307, 0.0679)
2015	0.0477<br>(0.0307, 0.0679)	0.0477<br>(0.0308, 0.0679)	0.0477<br>(0.0307, 0.068)	0.0477<br>(0.0308, 0.0679)	0.0477<br>(0.0307, 0.068)	0.0477<br>(0.0308, 0.0678)	0.0477<br>(0.0307, 0.0679)	0.0477<br>(0.0308, 0.068)	0.0477<br>(0.0308, 0.068)
2016	0.0477<br>(0.0307, 0.068)	0.0477<br>(0.0307, 0.068)	0.0477<br>(0.0308, 0.0679)	0.0477<br>(0.0308, 0.068)	0.0477<br>(0.0309, 0.068)	0.0477<br>(0.0308, 0.0681)	0.0477<br>(0.0308, 0.068)	0.0477<br>(0.0308, 0.0679)	0.0477<br>(0.0308, 0.068)

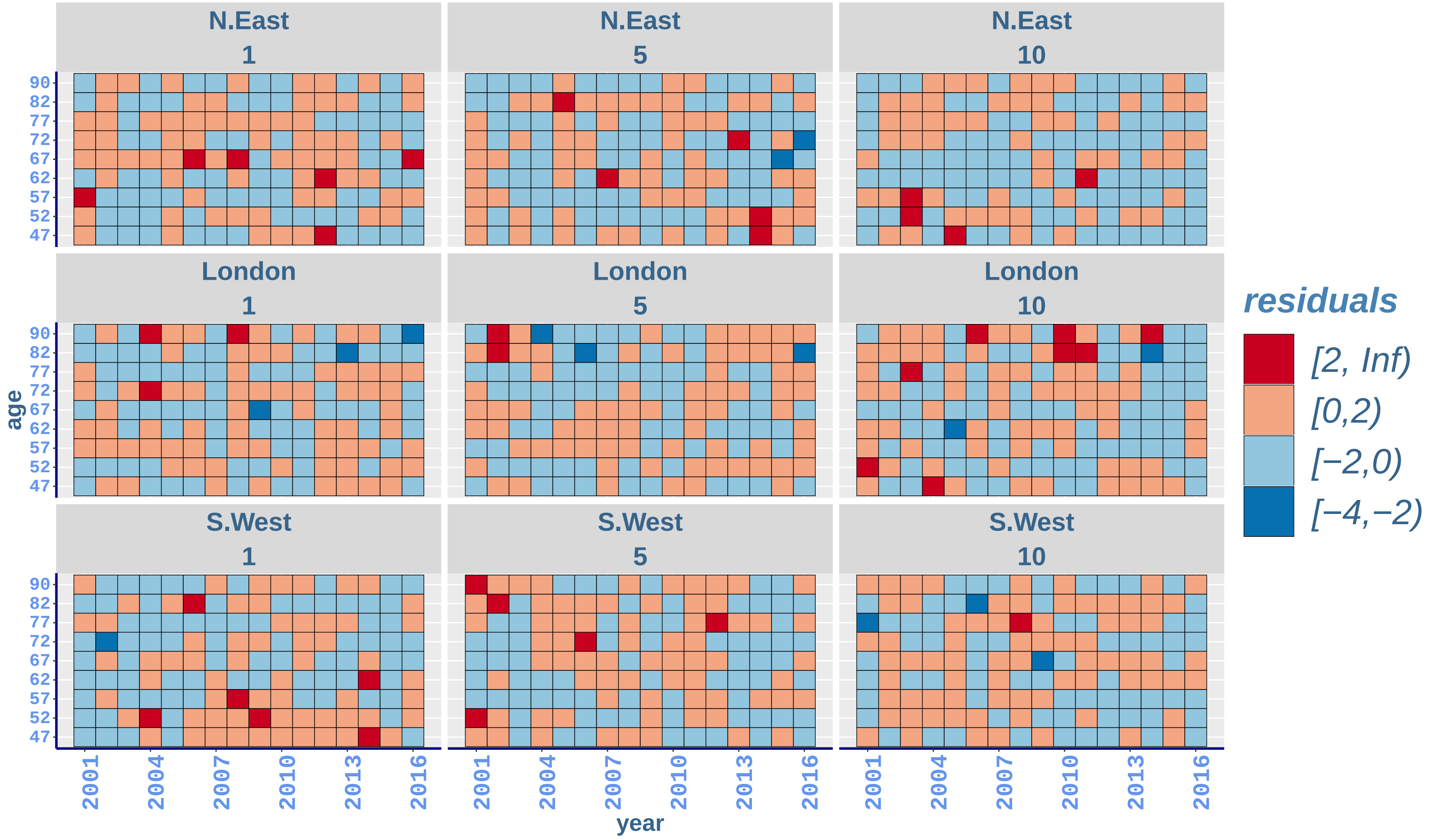

S71 Fig. Heat map of Pearson residuals for bowel cancer mortality for males in North East, London, and South West, deprivation deciles 1, 5, and 10: orange/light blue cells indicate areas with good fit, while red/dark blue cells indicate areas with poor fit. Note that there is a small number of residuals greater than 4, and these are included in the last category.

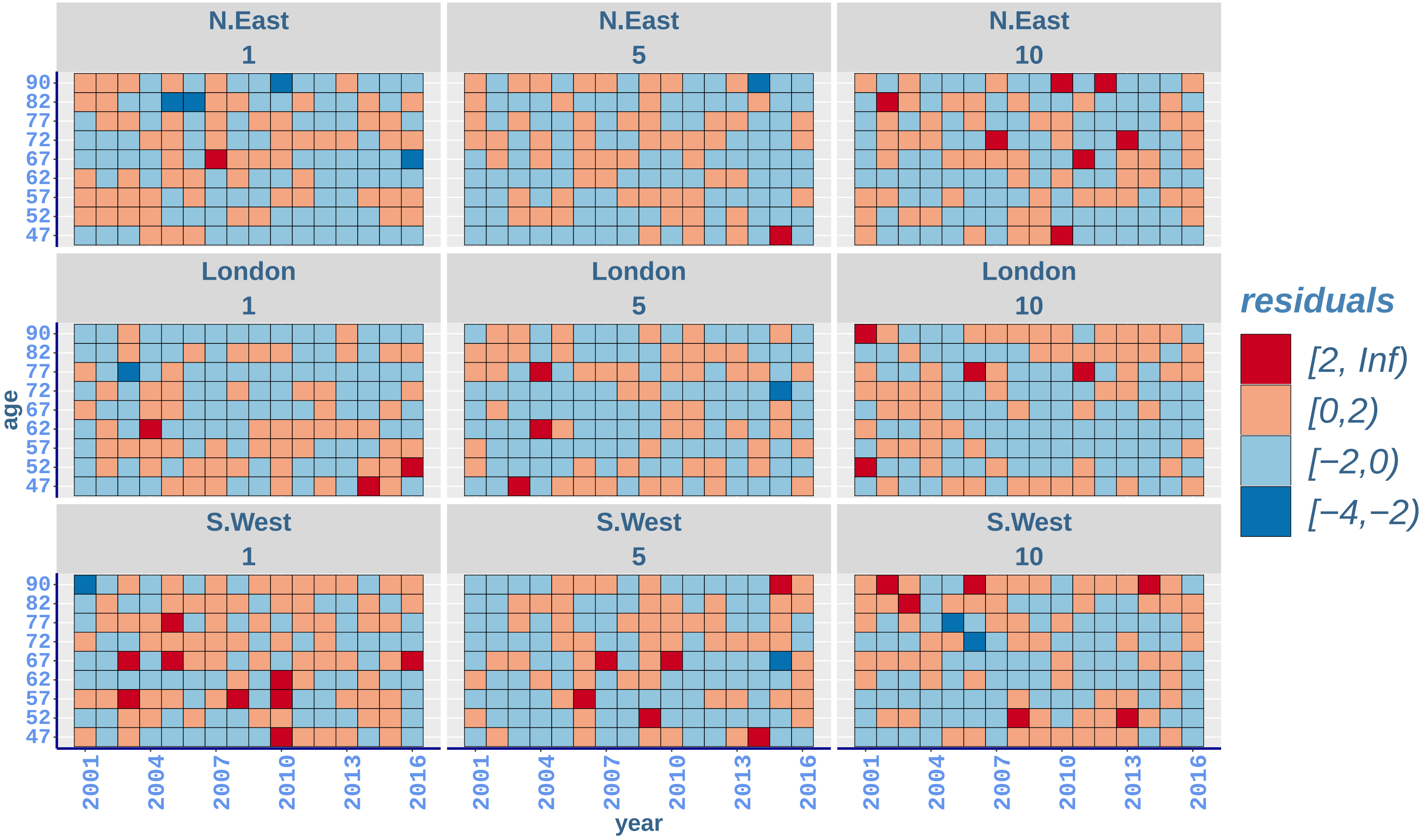

S72 Fig. Heat map of Pearson residuals for bowel cancer mortality for females in North East, London, and South West, deprivation deciles 1, 5, and 10: orange/light blue cells indicate areas with good fit, while red/dark blue cells indicate areas with poor fit. Note that there is a small number of residuals greater than 4, and these are included in the last category.

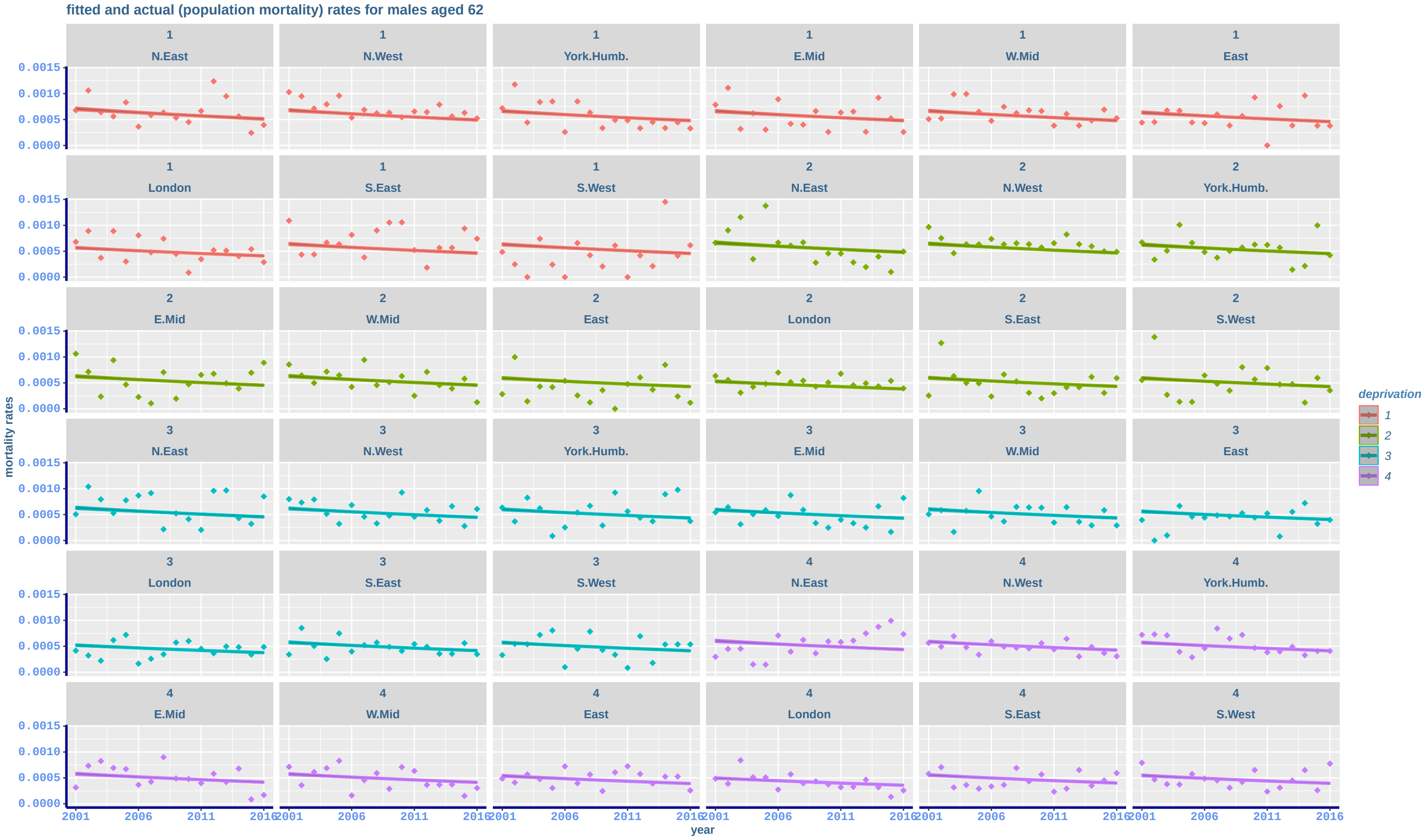

S73 Fig. Bowel cancer mortality for males, age 62, deprivation deciles 1-4 for all regions in England between 2001 and 2016: observed rates (dots), fitted rates (lines), with 95% credible intervals for the fitted rates.

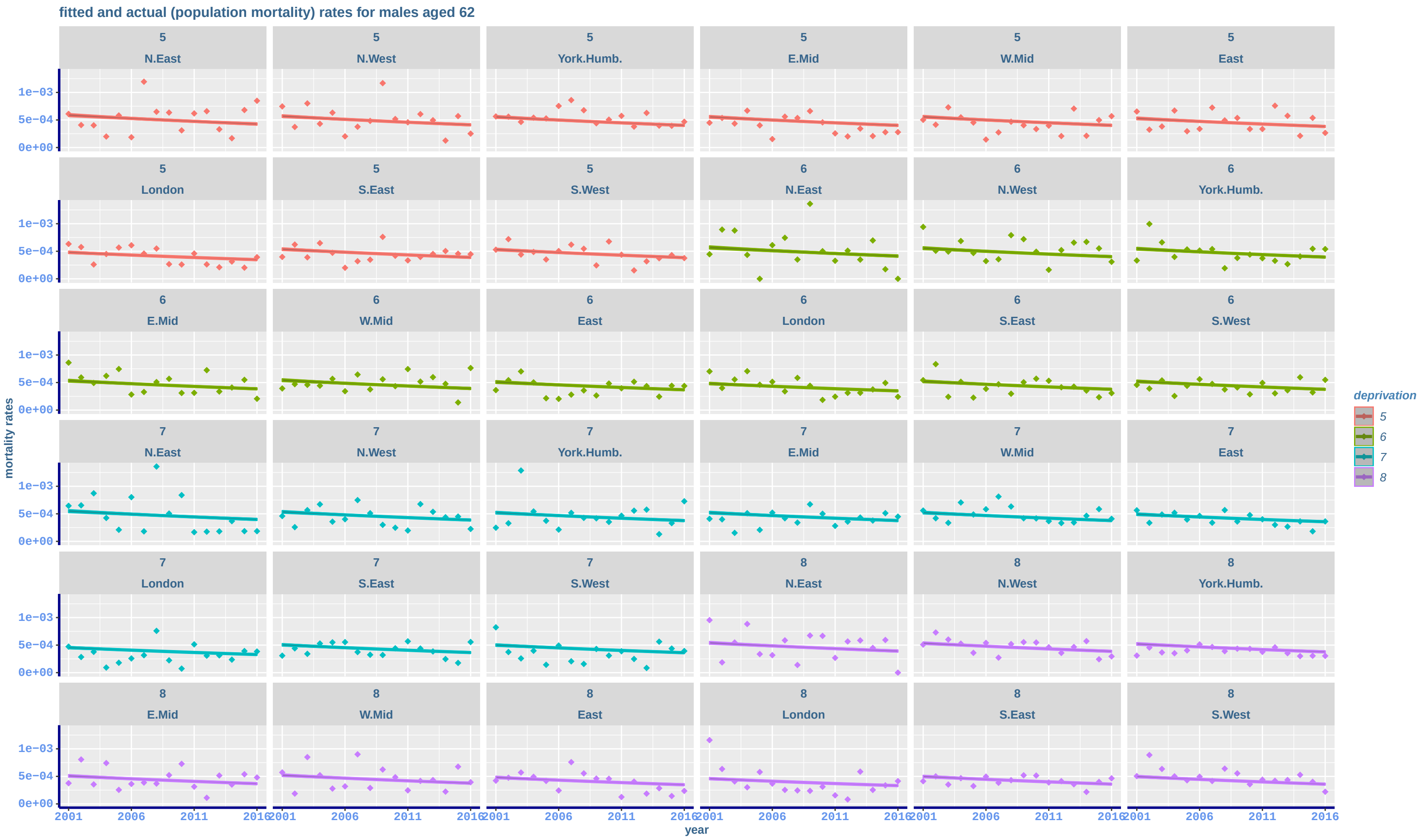

S74 Fig. Bowel cancer mortality for males, age 62, deprivation deciles 5-8 for all regions in England between 2001 and 2016: observed rates (dots), fitted rates (lines), with 95% credible intervals for the fitted rates.

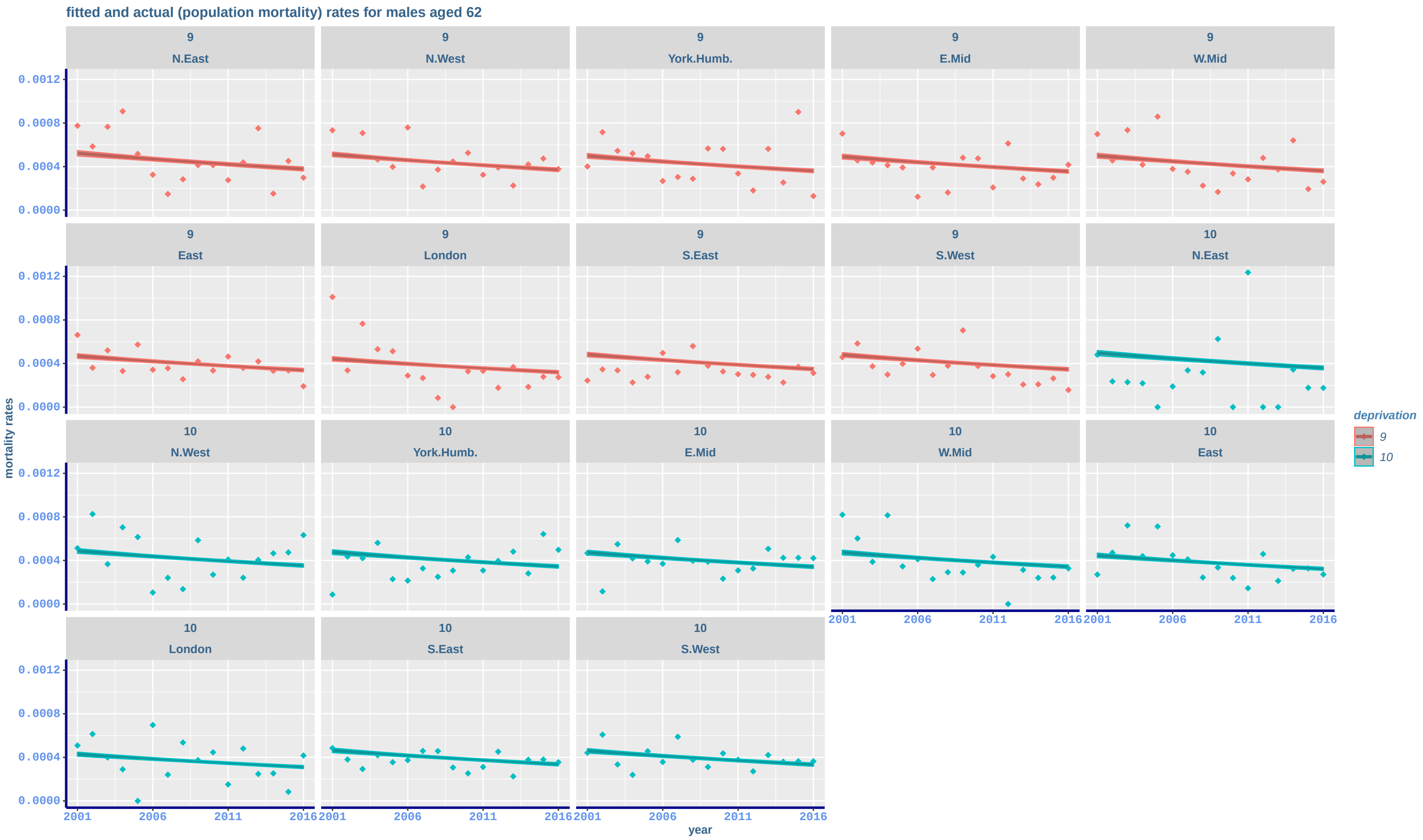

S75 Fig. Bowel cancer mortality for males, age 62, deprivation deciles 9-10 for all regions in England between 2001 and 2016: observed rates (dots), fitted rates (lines), with 95% credible intervals for the fitted rates.

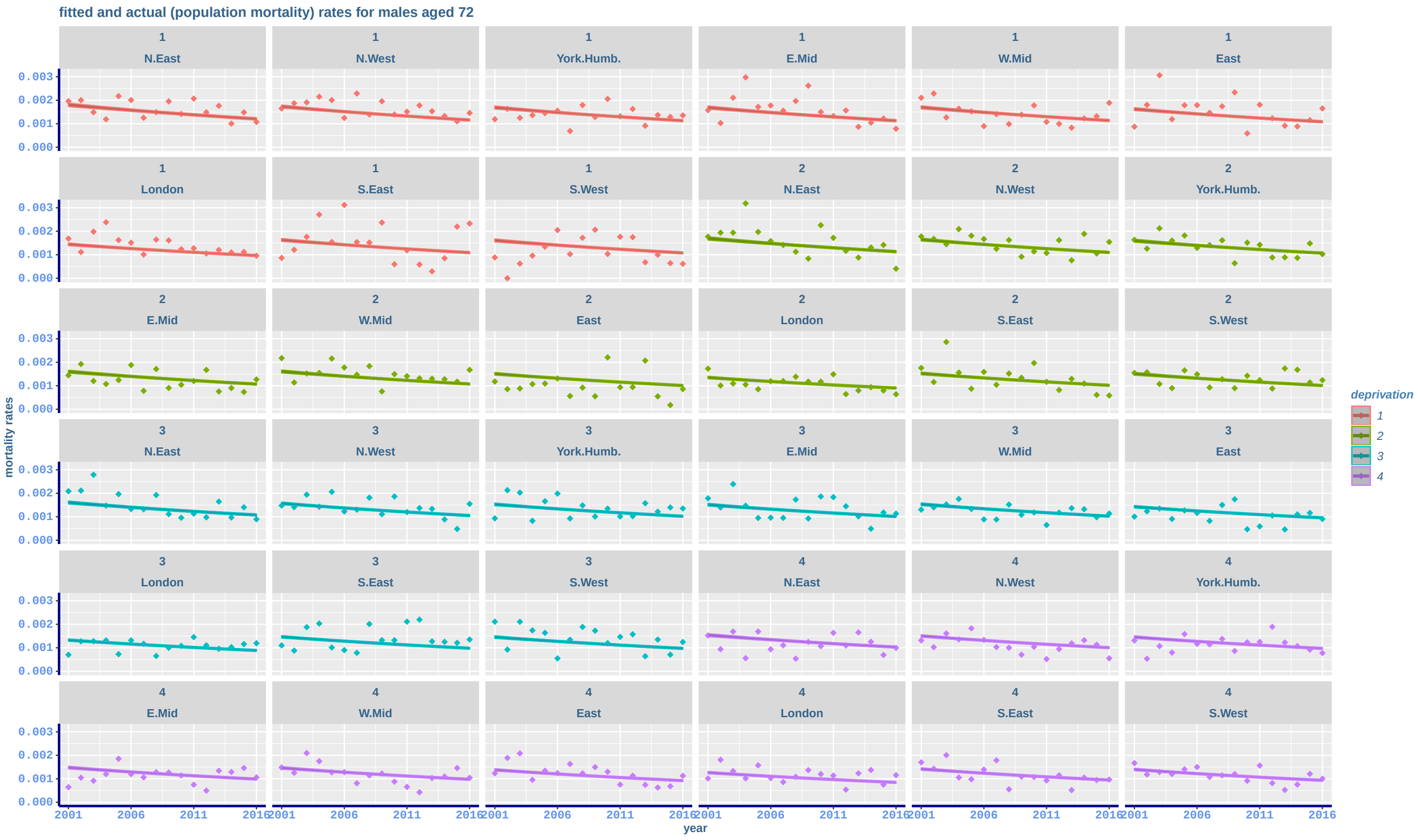

S76 Fig. Bowel cancer mortality for males, age 72, deprivation deciles 1-4 for all regions in England between 2001 and 2016: observed rates (dots), fitted rates (lines), with 95% credible intervals for the fitted rates.

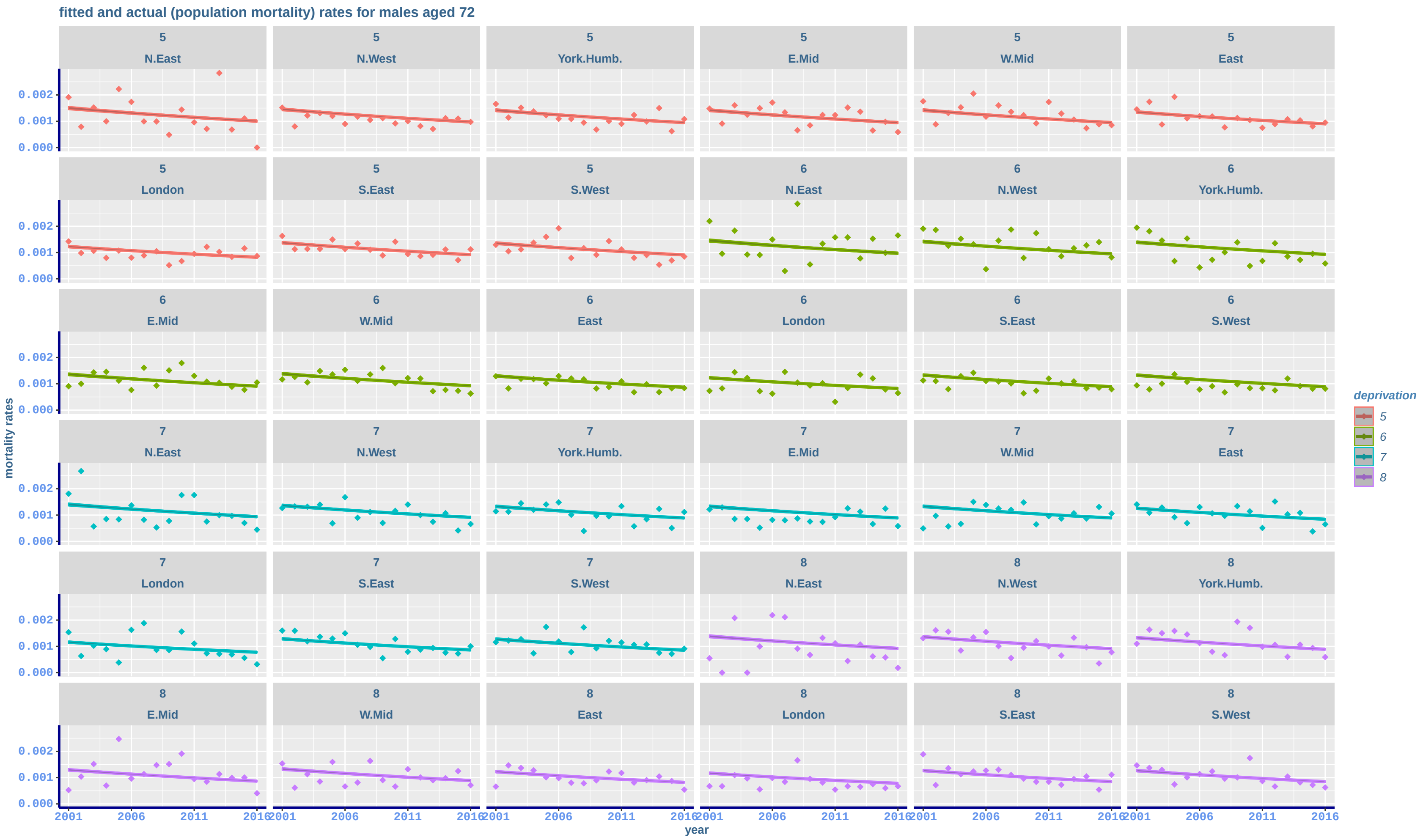

S77 Fig. Bowel cancer mortality for males, age 72, deprivation deciles 5-8 for all regions in England between 2001 and 2016: observed rates (dots), fitted rates (lines), with 95% credible intervals for the fitted rates.

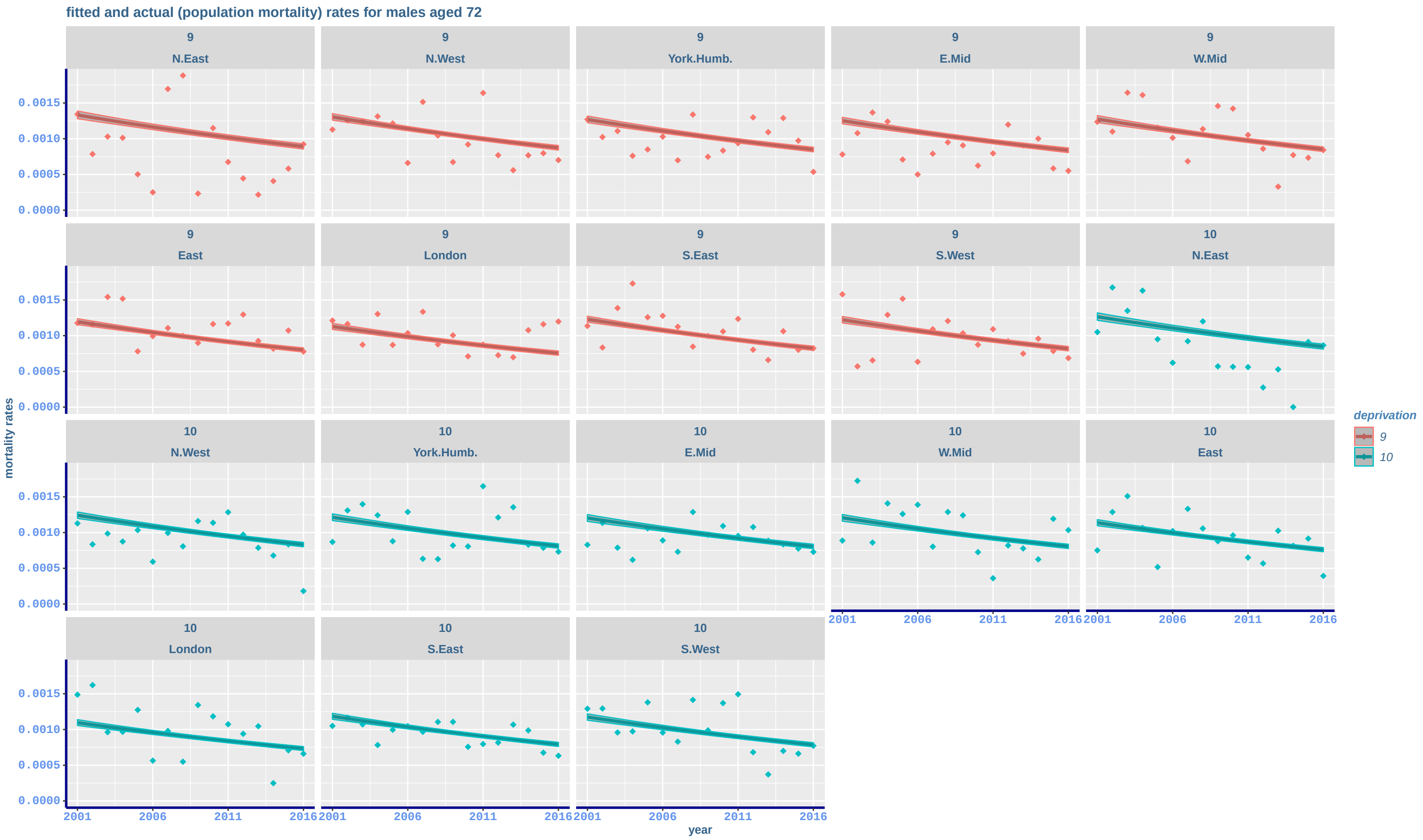

S78 Fig. Bowel cancer mortality for males, age 72, deprivation deciles 9-10 for all regions in England between 2001 and 2016: observed rates (dots), fitted rates (lines), with 95% credible intervals for the fitted rates.

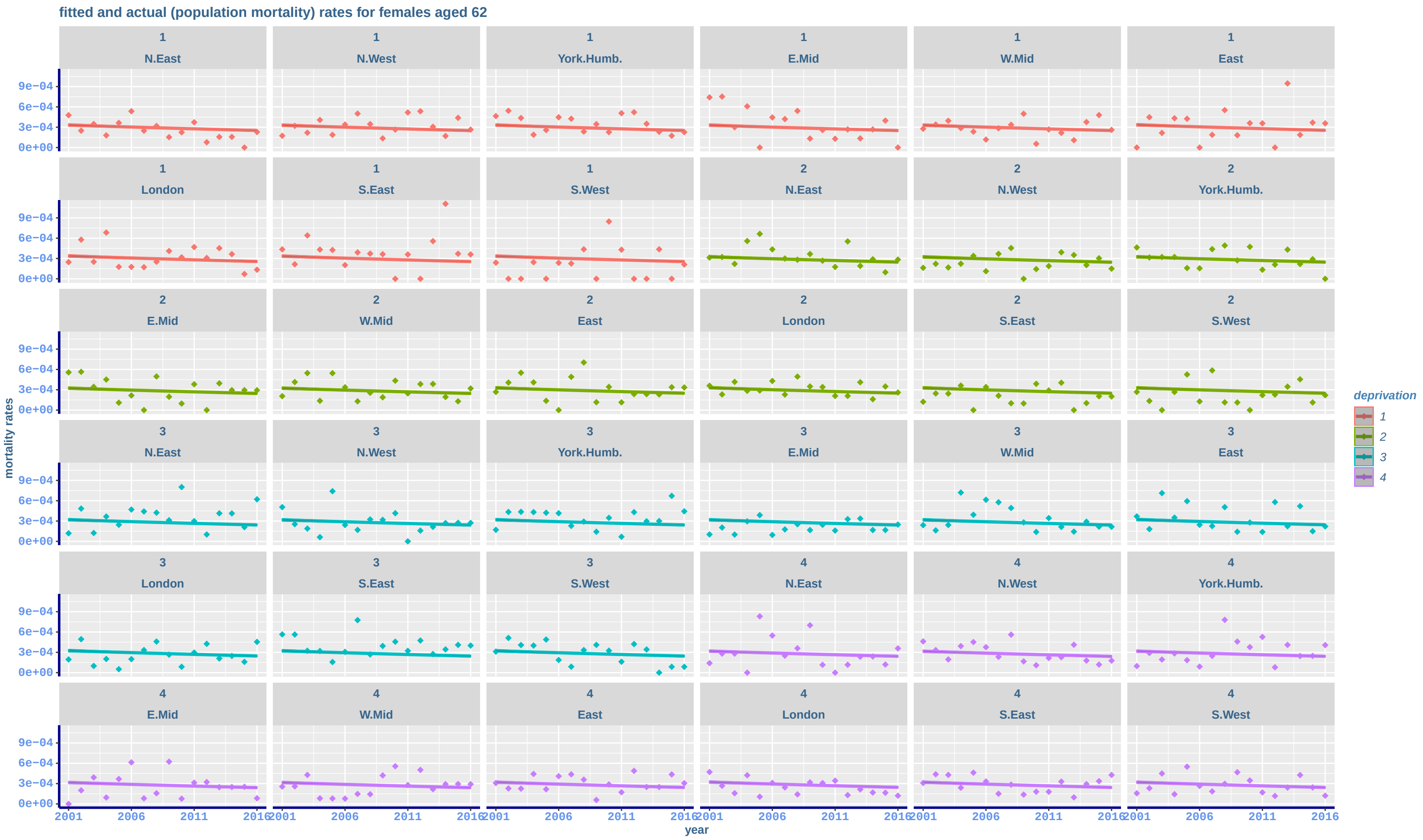

S79 Fig. Bowel cancer mortality for females, age 62, deprivation deciles 1-4 for all regions in England between 2001 and 2016: observed rates (dots), fitted rates (lines), with 95% credible intervals for the fitted rates.

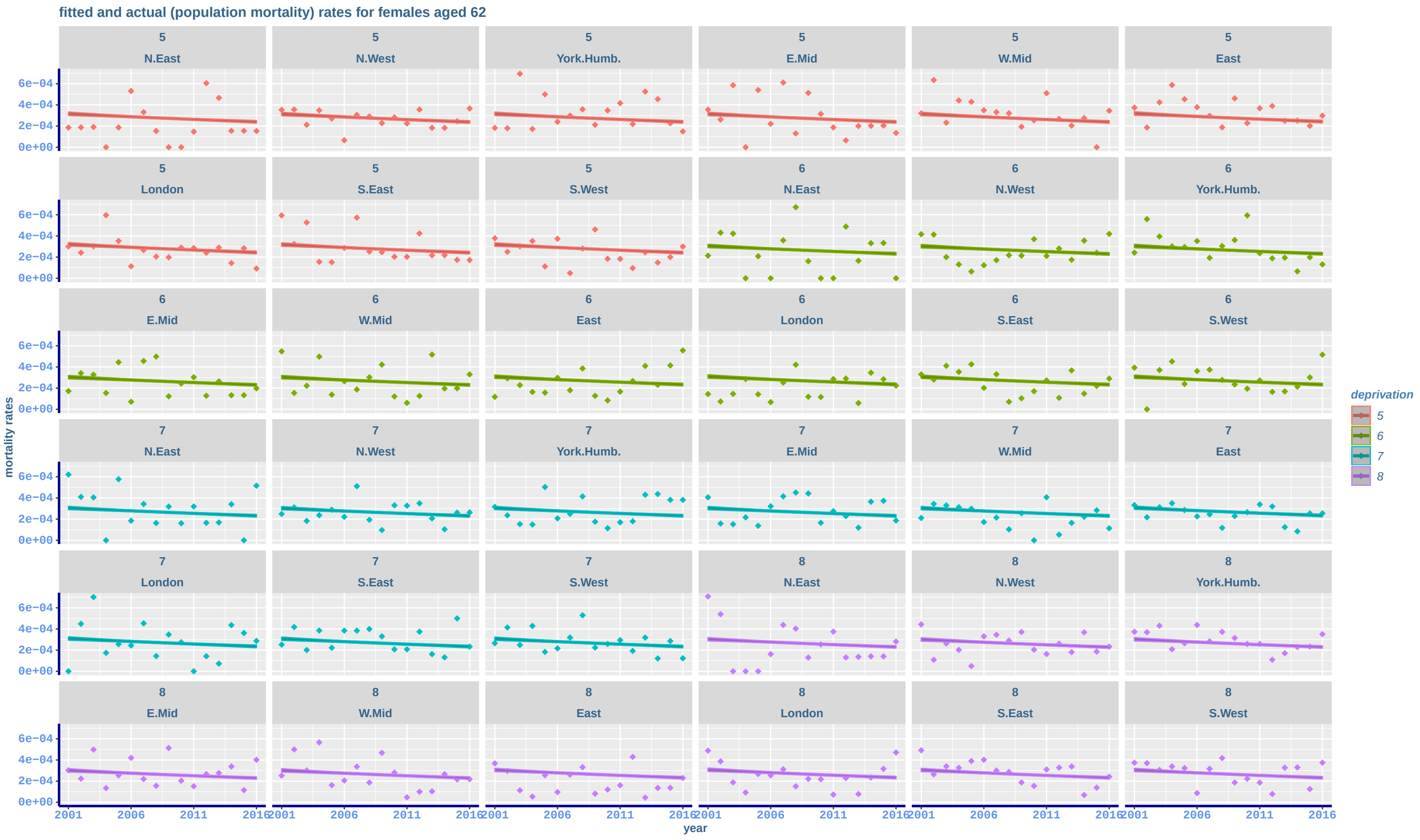

S80 Fig. Bowel cancer mortality for females, age 62, deprivation deciles 5-8 for all regions in England between 2001 and 2016: observed rates (dots), fitted rates (lines), with 95% credible intervals for the fitted rates.

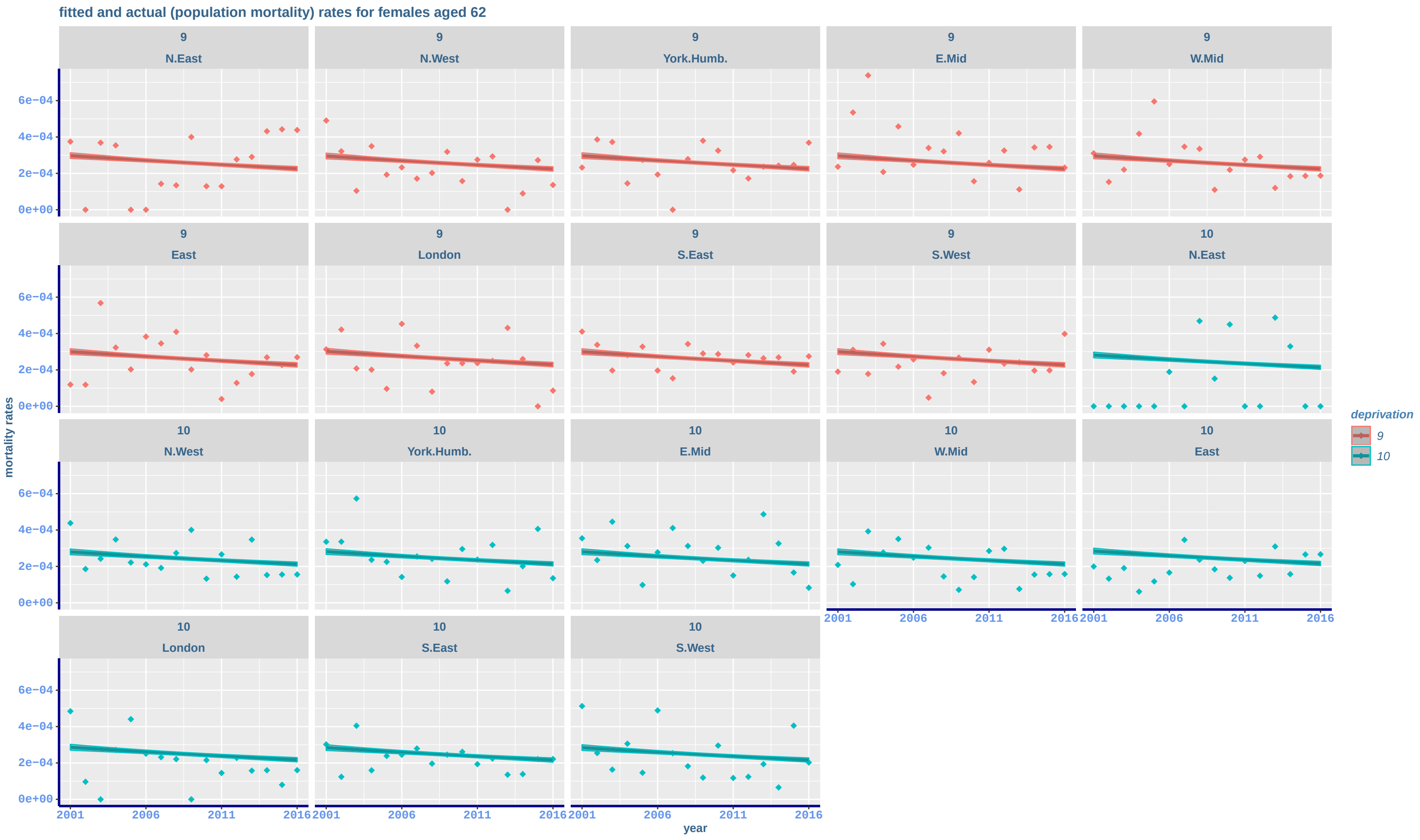

S81 Fig. Bowel cancer mortality for females, age 62, deprivation deciles 9-10 for all regions in England between 2001 and 2016: observed rates (dots), fitted rates (lines), with 95% credible intervals for the fitted rates.

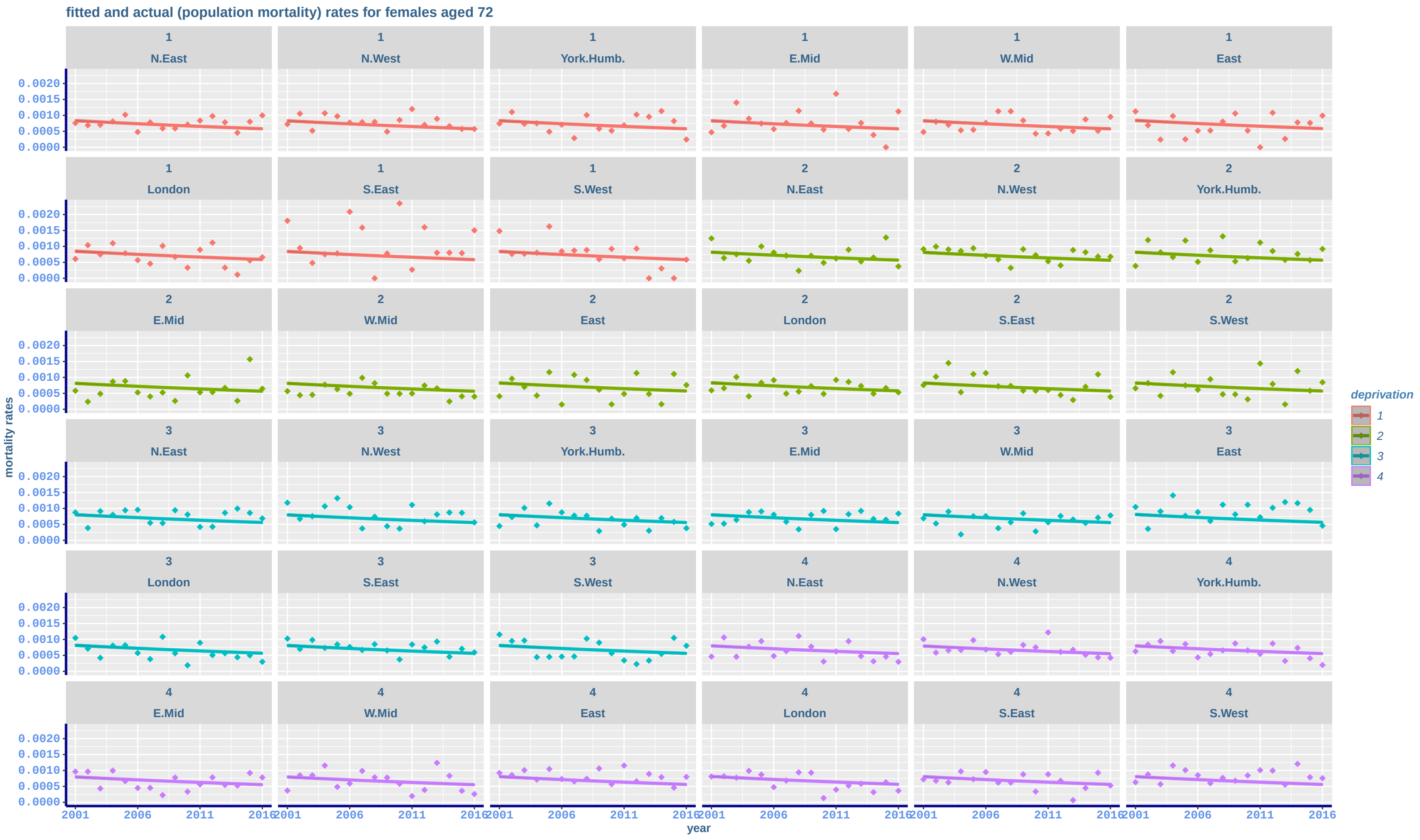

S82 Fig. Bowel cancer mortality for females, age 72, deprivation deciles 1-4 for all regions in England between 2001 and 2016: observed rates (dots), fitted rates (lines), with 95% credible intervals for the fitted rates.

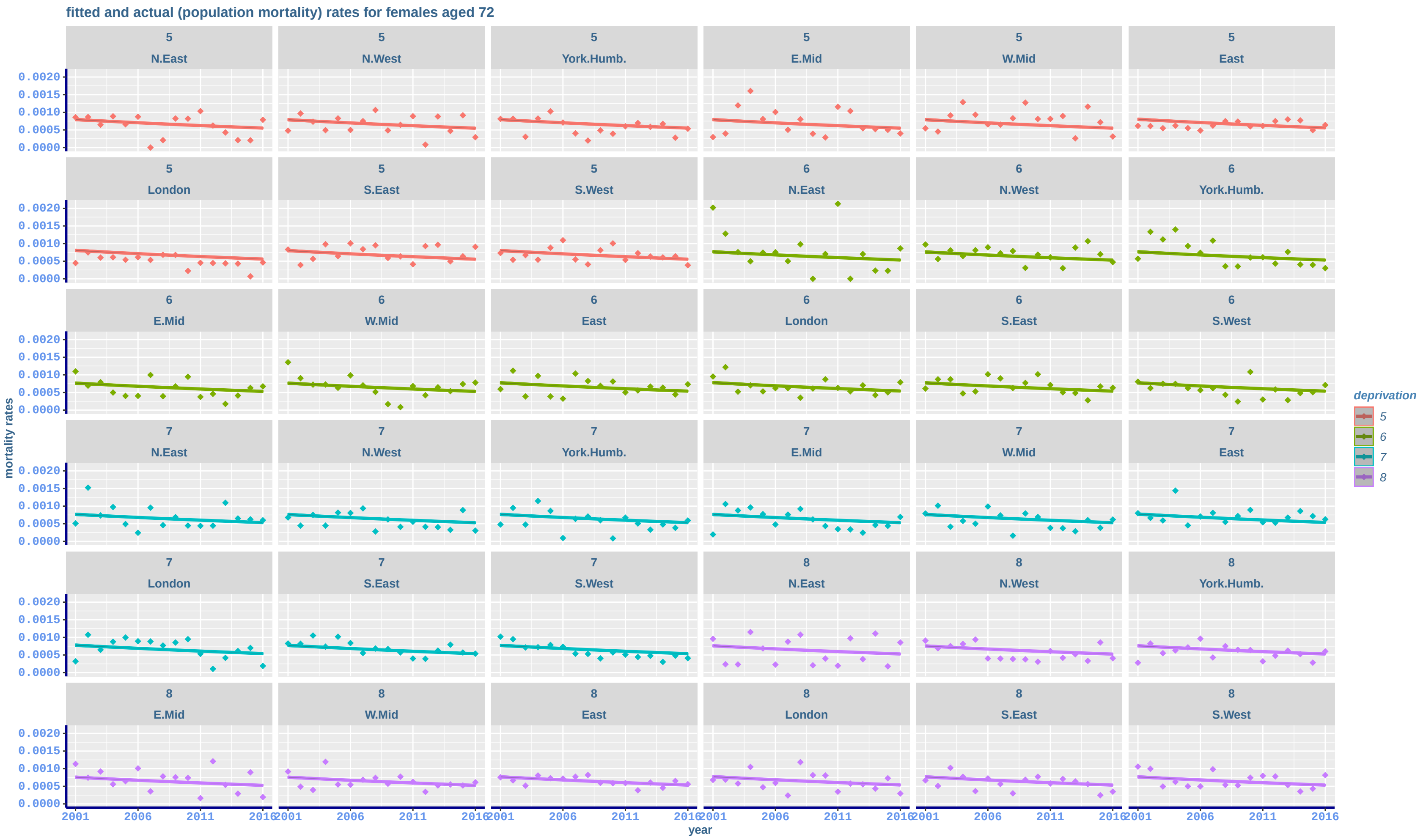

S83 Fig. Bowel cancer mortality for females, age 72, deprivation deciles 5-8 for all regions in England between 2001 and 2016: observed rates (dots), fitted rates (lines), with 95% credible intervals for the fitted rates.

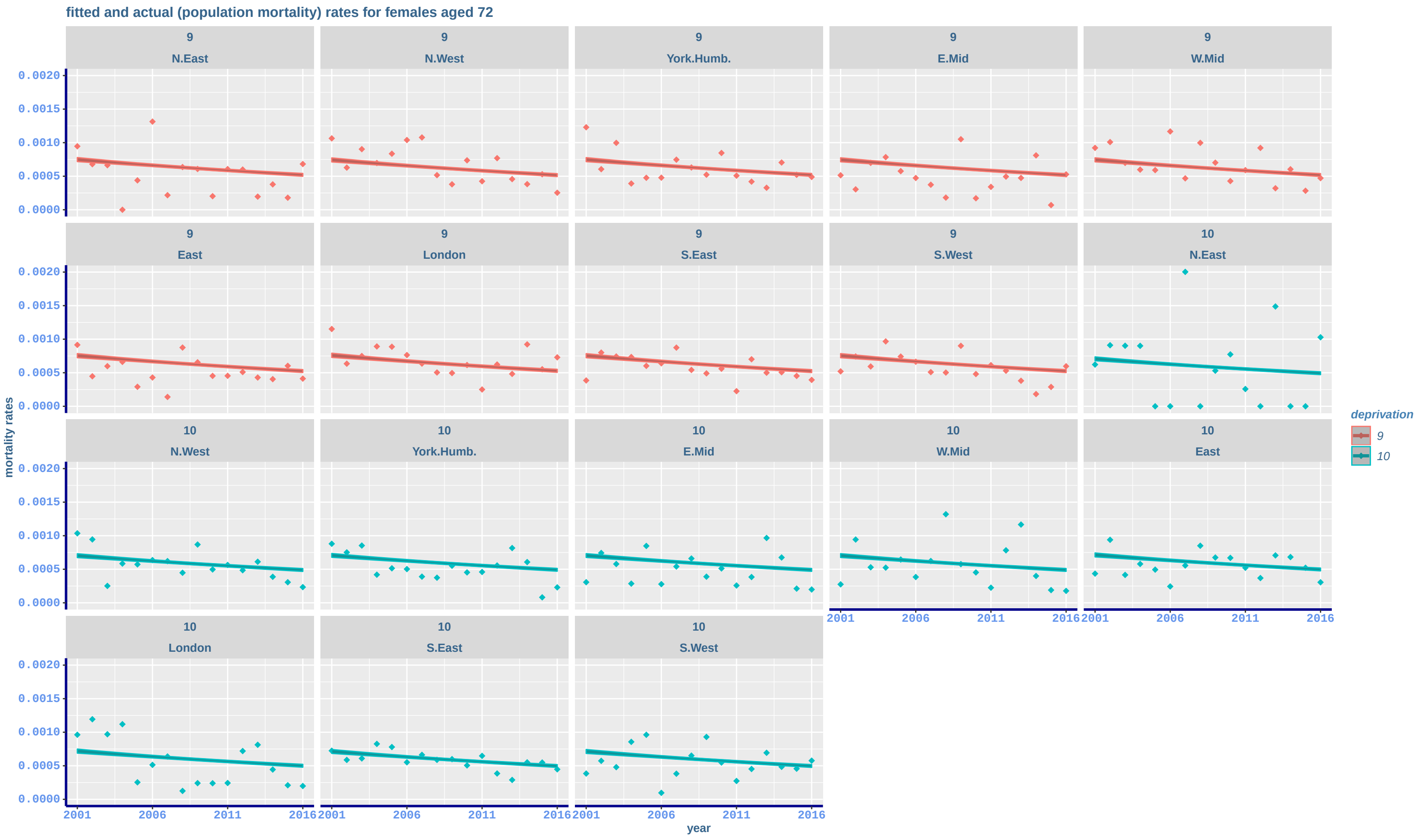

S84 Fig. Bowel cancer mortality for females, age 72, deprivation deciles 9-10 for all regions in England between 2001 and 2016: observed rates (dots), fitted rates (lines), with 95% credible intervals for the fitted rates.

S31 Table. Absolute deprivation differences ($AD_{t,r}$), per 100,000 people, in age-standardised fitted mortality rates of bowel cancer from 2001 to 2016 for all regions in England for males; 95% credible intervals in brackets

year	N.East	N.West	York.Humb.	E.Mid	W.Mid	East	London	S.East	S.West
2001	13<br>(12, 14)	13<br>(11, 14)	12<br>(11, 13)	12<br>(11, 13)	12<br>(11, 14)	12<br>(11, 13)	11<br>(10, 12)	12<br>(11, 13)	12<br>(11, 13)
2002	39<br>(37, 41)	38<br>(36, 40)	37<br>(35, 39)	37<br>(35, 39)	37<br>(35, 39)	35<br>(33, 37)	32<br>(30, 34)	36<br>(34, 38)	35<br>(33, 37)
2003	14<br>(13, 15)	14<br>(12, 15)	13<br>(12, 15)	13<br>(12, 15)	13<br>(12, 15)	13<br>(11, 14)	12<br>(11, 13)	13<br>(12, 14)	13<br>(12, 14)
2004	53<br>(50, 56)	51<br>(49, 54)	50<br>(47, 53)	50<br>(48, 53)	50<br>(47, 53)	48<br>(45, 51)	43<br>(41, 46)	48<br>(46, 51)	48<br>(45, 51)
2005	47<br>(43, 50)	45<br>(42, 49)	44<br>(41, 48)	44<br>(40, 47)	44<br>(41, 48)	42<br>(39, 45)	39<br>(36, 42)	42<br>(39, 46)	42<br>(39, 46)
2006	17<br>(15, 19)	17<br>(15, 19)	16<br>(14, 18)	16<br>(14, 18)	16<br>(14, 18)	15<br>(13, 17)	14<br>(12, 16)	16<br>(14, 18)	15<br>(13, 18)
2007	40<br>(36, 44)	38<br>(35, 43)	37<br>(33, 41)	38<br>(35, 42)	38<br>(34, 42)	36<br>(32, 40)	31<br>(28, 34)	36<br>(33, 40)	35<br>(32, 39)
2008	16<br>(13, 18)	15<br>(13, 18)	15<br>(13, 17)	15<br>(13, 17)	15<br>(13, 17)	14<br>(12, 16)	13<br>(11, 15)	14<br>(12, 17)	14<br>(12, 16)
2009	17<br>(14, 20)	16<br>(14, 19)	16<br>(13, 19)	16<br>(13, 18)	16<br>(13, 19)	15<br>(13, 17)	14<br>(12, 16)	15<br>(13, 18)	15<br>(13, 18)
2010	26<br>(21, 31)	25<br>(21, 30)	25<br>(21, 30)	23<br>(19, 28)	25<br>(20, 30)	23<br>(19, 27)	21<br>(17, 25)	23<br>(19, 28)	23<br>(19, 28)
2011	13<br>(10, 15)	12<br>(10, 15)	12<br>(9, 15)	12<br>(9, 14)	12<br>(9, 15)	11<br>(9, 14)	10<br>(8, 13)	11<br>(9, 14)	11<br>(9, 14)
2012	12<br>(7, 17)	11<br>(6, 16)	11<br>(6, 15)	11<br>(6, 16)	11<br>(6, 15)	11<br>(6, 15)	8<br>(5, 12)	10<br>(6, 15)	10<br>(6, 14)
2013	15<br>(12, 18)	15<br>(12, 18)	14<br>(11, 17)	15<br>(12, 17)	15<br>(12, 17)	13<br>(10, 16)	11<br>(8, 13)	13<br>(10, 16)	13<br>(10, 15)
2014	8<br>(5, 11)	8<br>(5, 11)	8<br>(5, 11)	8<br>(5, 11)	8<br>(5, 11)	7<br>(5, 10)	7<br>(5, 9)	8<br>(5, 10)	8<br>(5, 10)
2015	54<br>(51, 57)	51<br>(48, 54)	49<br>(47, 52)	50<br>(47, 53)	50<br>(48, 53)	48<br>(46, 51)	41<br>(39, 44)	48<br>(45, 50)	47<br>(44, 49)
2016	11<br>(10, 12)	11<br>(10, 12)	10<br>(9, 11)	10<br>(9, 11)	10<br>(9, 11)	10<br>(9, 11)	9<br>(8, 10)	10<br>(9, 11)	10<br>(9, 11)

S32 Table. Relative deprivation differences ($\text{RD}_{t,r}$) in age-standardised fitted mortality rates of bowel cancer from 2001 to 2016 for all regions in England for males; 95% credible intervals in brackets

year	N.East	N.West	York.Humb.	E.Mid	W.Mid	East	London	S.East	S.West
2001	0.1448 (0.1317, 0.1595)	0.1444 (0.1312, 0.159)	0.1446 (0.1315, 0.1593)	0.145 (0.132, 0.1597)	0.1444 (0.1312, 0.159)	0.1448 (0.1317, 0.1595)	0.1436 (0.1306, 0.1583)	0.1444 (0.1313, 0.159)	0.1443 (0.1311, 0.1589)
2002	0.4778 (0.4485, 0.5092)	0.4761 (0.4469, 0.5076)	0.477 (0.4479, 0.5084)	0.4794 (0.45, 0.5109)	0.4757 (0.4464, 0.5071)	0.476 (0.4465, 0.5074)	0.4683 (0.4392, 0.4995)	0.4738 (0.4446, 0.5051)	0.4732 (0.4442, 0.5046)
2003	0.1409 (0.1275, 0.1558)	0.1403 (0.1269, 0.1551)	0.1403 (0.1268, 0.1551)	0.1412 (0.1279, 0.156)	0.1404 (0.1269, 0.1553)	0.1412 (0.1278, 0.1561)	0.1396 (0.1262, 0.1545)	0.1407 (0.1273, 0.1556)	0.1404 (0.127, 0.1552)
2004	0.5488 (0.516, 0.5853)	0.5422 (0.5096, 0.5785)	0.5421 (0.5095, 0.5785)	0.5523 (0.5194, 0.589)	0.5431 (0.5103, 0.5793)	0.552 (0.5191, 0.5887)	0.5356 (0.503, 0.5719)	0.5469 (0.514, 0.5835)	0.5436 (0.5108, 0.5801)
2005	0.3777 (0.3456, 0.4112)	0.3783 (0.346, 0.412)	0.3789 (0.3466, 0.4124)	0.3751 (0.3432, 0.4086)	0.3786 (0.3465, 0.4124)	0.3765 (0.3445, 0.4101)	0.3776 (0.3453, 0.4113)	0.3755 (0.3436, 0.4088)	0.3763 (0.3442, 0.4097)
2006	0.1146 (0.0997, 0.1307)	0.1145 (0.0996, 0.1306)	0.114 (0.0992, 0.1302)	0.1152 (0.1005, 0.1312)	0.1145 (0.0998, 0.1307)	0.1145 (0.0996, 0.1306)	0.1128 (0.098, 0.1291)	0.1143 (0.0996, 0.1304)	0.1139 (0.099, 0.1301)
2007	0.2805 (0.2502, 0.3134)	0.278 (0.2475, 0.311)	0.2742 (0.2438, 0.3071)	0.2856 (0.2552, 0.3185)	0.2786 (0.2481, 0.3115)	0.2809 (0.2505, 0.3141)	0.2623 (0.232, 0.295)	0.2782 (0.2475, 0.3111)	0.2738 (0.2433, 0.3067)
2008	0.103 (0.087, 0.1198)	0.1032 (0.0873, 0.1199)	0.1031 (0.0872, 0.1198)	0.1028 (0.0869, 0.1196)	0.1032 (0.0874, 0.1199)	0.1027 (0.0867, 0.1195)	0.1026 (0.0867, 0.1194)	0.1026 (0.0867, 0.1194)	0.1027 (0.0869, 0.1195)
2009	0.0978 (0.0826, 0.1141)	0.0974 (0.0823, 0.1137)	0.0977 (0.0825, 0.114)	0.0969 (0.0817, 0.1133)	0.0976 (0.0826, 0.1139)	0.0978 (0.0827, 0.114)	0.097 (0.0818, 0.1133)	0.0971 (0.082, 0.1134)	0.0973 (0.0822, 0.1137)
2010	0.1604 (0.1302, 0.1943)	0.1592 (0.1289, 0.1931)	0.1618 (0.1316, 0.1959)	0.1514 (0.1212, 0.185)	0.1607 (0.1305, 0.1947)	0.1582 (0.1279, 0.192)	0.158 (0.1276, 0.192)	0.155 (0.1248, 0.1888)	0.158 (0.1277, 0.192)
2011	0.074 (0.0578, 0.0914)	0.0736 (0.0573, 0.0909)	0.0737 (0.0575, 0.091)	0.0739 (0.0576, 0.0913)	0.0737 (0.0574, 0.0911)	0.0745 (0.0583, 0.0917)	0.0737 (0.0574, 0.091)	0.074 (0.0577, 0.0914)	0.0739 (0.0576, 0.0912)
2012	0.0706 (0.0393, 0.1022)	0.0663 (0.0364, 0.0978)	0.0667 (0.0365, 0.098)	0.0701 (0.0388, 0.1015)	0.067 (0.0367, 0.0984)	0.0737 (0.0425, 0.1051)	0.0608 (0.0356, 0.0914)	0.0667 (0.0369, 0.0981)	0.0658 (0.0366, 0.0971)
2013	0.0977 (0.0762, 0.1188)	0.1002 (0.0788, 0.1212)	0.0974 (0.076, 0.1184)	0.1006 (0.079, 0.1217)	0.0992 (0.0778, 0.1202)	0.0936 (0.0722, 0.1144)	0.0849 (0.0644, 0.1055)	0.0908 (0.0695, 0.1114)	0.0917 (0.0704, 0.1125)
2014	0.0575 (0.0372, 0.0776)	0.0584 (0.0382, 0.0783)	0.0583 (0.0381, 0.0783)	0.0578 (0.0376, 0.0778)	0.0581 (0.0379, 0.0781)	0.0572 (0.037, 0.0772)	0.0597 (0.0398, 0.0796)	0.0583 (0.0381, 0.0782)	0.0586 (0.0385, 0.0785)
2015	0.5899 (0.5591, 0.6238)	0.5701 (0.5402, 0.6035)	0.5713 (0.5414, 0.6047)	0.5805 (0.5502, 0.6142)	0.5768 (0.5468, 0.6104)	0.5938 (0.5627, 0.6279)	0.5361 (0.507, 0.5688)	0.5688 (0.5389, 0.6021)	0.5638 (0.5341, 0.5972)
2016	0.1372 (0.1235, 0.1519)	0.1369 (0.1232, 0.1515)	0.1369 (0.1234, 0.1517)	0.1368 (0.1231, 0.1515)	0.1371 (0.1234, 0.1518)	0.1369 (0.1232, 0.1516)	0.1354 (0.1217, 0.1502)	0.1362 (0.1226, 0.1509)	0.1363 (0.1226, 0.1511)

S33 Table. Absolute deprivation differences ($AD_{t,r}$), per 100,000 people, in age-standardised fitted mortality rates of bowel cancer from 2001 to 2016 for all regions in England for females; 95% credible intervals in brackets

year	N.East	N.West	York.Humb.	E.Mid	W.Mid	East	London	S.East	S.West
2001	5<br>(5, 6)	5<br>(5, 6)	5<br>(5, 6)	5<br>(5, 6)	5<br>(5, 6)	5<br>(5, 6)	5<br>(5, 6)	5<br>(5, 6)	5<br>(5, 6)
2002	18<br>(17, 20)	18<br>(17, 20)	18<br>(17, 20)	18<br>(17, 20)	18<br>(17, 20)	18<br>(17, 20)	19<br>(17, 20)	18<br>(17, 20)	18<br>(17, 20)
2003	6<br>(5, 7)	6<br>(5, 7)	6<br>(5, 7)	6<br>(5, 7)	6<br>(5, 7)	6<br>(5, 7)	6<br>(5, 7)	6<br>(5, 7)	6<br>(5, 7)
2004	28<br>(27, 30)	28<br>(26, 30)	28<br>(26, 30)	28<br>(26, 30)	28<br>(26, 30)	29<br>(27, 30)	29<br>(27, 31)	29<br>(27, 30)	29<br>(27, 30)
2005	23<br>(20, 25)	22<br>(20, 25)	23<br>(20, 25)	23<br>(20, 25)	23<br>(20, 25)	23<br>(21, 25)	23<br>(21, 25)	23<br>(21, 25)	23<br>(21, 25)
2006	7<br>(6, 9)	7<br>(6, 8)	7<br>(6, 8)	7<br>(6, 8)	7<br>(6, 8)	7<br>(6, 9)	7<br>(6, 9)	7<br>(6, 9)	7<br>(6, 9)
2007	19<br>(16, 21)	19<br>(16, 21)	19<br>(16, 21)	19<br>(16, 21)	19<br>(16, 21)	19<br>(16, 22)	19<br>(16, 22)	19<br>(16, 22)	19<br>(16, 22)
2008	7<br>(5, 8)	6<br>(5, 8)	7<br>(5, 8)	7<br>(5, 8)	7<br>(5, 8)	7<br>(5, 8)	7<br>(5, 8)	7<br>(5, 8)	7<br>(5, 8)
2009	7<br>(5, 9)	7<br>(5, 8)	7<br>(5, 9)	7<br>(5, 9)	7<br>(5, 9)	7<br>(5, 9)	7<br>(5, 9)	7<br>(5, 9)	7<br>(5, 9)
2010	11<br>(8, 14)	11<br>(8, 14)	11<br>(8, 14)	11<br>(8, 14)	11<br>(8, 14)	11<br>(8, 14)	11<br>(8, 14)	11<br>(8, 14)	11<br>(8, 14)
2011	5<br>(3, 7)	5<br>(3, 7)	5<br>(3, 7)	5<br>(3, 7)	5<br>(3, 7)	5<br>(3, 7)	5<br>(4, 7)	5<br>(3, 7)	5<br>(3, 7)
2012	5<br>(2, 8)	4<br>(2, 7)	5<br>(2, 7)	5<br>(2, 7)	4<br>(2, 7)	5<br>(2, 8)	5<br>(2, 8)	5<br>(2, 8)	5<br>(2, 8)
2013	9<br>(7, 11)	9<br>(7, 11)	9<br>(7, 11)	9<br>(7, 11)	9<br>(7, 11)	9<br>(7, 11)	9<br>(7, 11)	9<br>(7, 11)	9<br>(7, 11)
2014	3<br>(2, 5)	3<br>(2, 5)	3<br>(2, 5)	3<br>(2, 5)	3<br>(2, 5)	3<br>(2, 5)	3<br>(2, 5)	3<br>(2, 5)	3<br>(2, 5)
2015	34<br>(32, 36)	34<br>(32, 35)	34<br>(32, 36)	34<br>(32, 36)	34<br>(32, 36)	34<br>(32, 36)	34<br>(32, 36)	34<br>(32, 36)	34<br>(32, 36)
2016	5<br>(4, 5)	5<br>(4, 5)	5<br>(4, 5)	5<br>(4, 5)	5<br>(4, 5)	5<br>(4, 5)	5<br>(4, 5)	5<br>(4, 5)	5<br>(4, 5)

S34 Table. Relative deprivation differences ($RD_{t,r}$) in age-standardised fitted mortality rates of bowel cancer from 2001 to 2016 for all regions in England for females; 95% credible intervals in brackets

year	N.East	N.West	York.Humb.	E.Mid	W.Mid	East	London	S.East	S.West
2001	0.0956 (0.0816, 0.11)	0.0956 (0.0817, 0.11)	0.0956 (0.0816, 0.1099)	0.0956 (0.0816, 0.11)	0.0956 (0.0817, 0.1099)	0.0956 (0.0817, 0.1098)	0.0956 (0.0816, 0.11)	0.0956 (0.0817, 0.11)	0.0956 (0.0816, 0.1099)
2002	0.3437 (0.3122, 0.3753)	0.3437 (0.3122, 0.3752)	0.3438 (0.3123, 0.3751)	0.3438 (0.3123, 0.3752)	0.3437 (0.3122, 0.375)	0.3437 (0.3121, 0.375)	0.3437 (0.3123, 0.3751)	0.3437 (0.3122, 0.3752)	0.3437 (0.3123, 0.3751)
2003	0.0966 (0.0828, 0.1111)	0.0966 (0.0829, 0.111)	0.0966 (0.0829, 0.1109)	0.0966 (0.0829, 0.1111)	0.0966 (0.0829, 0.111)	0.0966 (0.0828, 0.111)	0.0965 (0.0828, 0.1112)	0.0966 (0.0828, 0.111)	0.0966 (0.0828, 0.1111)
2004	0.4634 (0.4301, 0.4985)	0.4634 (0.4302, 0.4982)	0.4634 (0.4301, 0.4983)	0.4635 (0.4304, 0.4984)	0.4634 (0.4302, 0.4981)	0.4635 (0.4302, 0.4984)	0.4633 (0.4301, 0.4981)	0.4634 (0.4303, 0.4983)	0.4634 (0.4301, 0.4983)
2005	0.2893 (0.2579, 0.3216)	0.2893 (0.2579, 0.3216)	0.2893 (0.2581, 0.3216)	0.2892 (0.2579, 0.3216)	0.2893 (0.258, 0.3216)	0.2893 (0.258, 0.3216)	0.2893 (0.2581, 0.3216)	0.2892 (0.2579, 0.3216)	0.2893 (0.258, 0.3216)
2006	0.0777 (0.0626, 0.0928)	0.0777 (0.0626, 0.0929)	0.0777 (0.0625, 0.0928)	0.0777 (0.0626, 0.0929)	0.0777 (0.0626, 0.0929)	0.0777 (0.0627, 0.0929)	0.0777 (0.0626, 0.0929)	0.0777 (0.0626, 0.0929)	0.0777 (0.0626, 0.0929)
2007	0.211 (0.1796, 0.2442)	0.2109 (0.1796, 0.2442)	0.2109 (0.1796, 0.2442)	0.211 (0.1797, 0.2443)	0.2109 (0.1796, 0.2442)	0.211 (0.1796, 0.2443)	0.2108 (0.1795, 0.2443)	0.2109 (0.1796, 0.2444)	0.2109 (0.1796, 0.2443)
2008	0.0679 (0.0521, 0.0836)	0.0679 (0.0521, 0.0836)	0.0679 (0.052, 0.0838)	0.0679 (0.0521, 0.0838)	0.0679 (0.0521, 0.0836)	0.0679 (0.0522, 0.0837)	0.0679 (0.0521, 0.0837)	0.0679 (0.0521, 0.0838)	0.0679 (0.0521, 0.0837)
2009	0.0659 (0.0505, 0.0818)	0.0659 (0.0505, 0.0817)	0.0659 (0.0506, 0.0818)	0.0659 (0.0506, 0.0819)	0.0659 (0.0507, 0.0817)	0.0659 (0.0506, 0.0818)	0.0659 (0.0507, 0.0818)	0.0659 (0.0505, 0.0818)	0.0659 (0.0507, 0.0818)
2010	0.1074 (0.0762, 0.14)	0.1074 (0.0763, 0.14)	0.1074 (0.0763, 0.14)	0.1073 (0.0761, 0.1399)	0.1074 (0.0763, 0.1401)	0.1074 (0.0764, 0.1401)	0.1074 (0.0762, 0.1399)	0.1073 (0.0763, 0.1399)	0.1074 (0.0763, 0.14)
2011	0.0493 (0.033, 0.0653)	0.0493 (0.0331, 0.0652)	0.0493 (0.033, 0.0652)	0.0493 (0.033, 0.0653)	0.0493 (0.033, 0.0652)	0.0493 (0.033, 0.0654)	0.0493 (0.0329, 0.0653)	0.0493 (0.033, 0.0653)	0.0493 (0.0329, 0.0652)
2012	0.0441 (0.0181, 0.0743)	0.0441 (0.0181, 0.0741)	0.0441 (0.018, 0.0741)	0.0441 (0.018, 0.0742)	0.0441 (0.018, 0.0741)	0.0442 (0.018, 0.0743)	0.044 (0.0181, 0.0741)	0.0441 (0.018, 0.0742)	0.0441 (0.018, 0.0741)
2013	0.0954 (0.0736, 0.1179)	0.0954 (0.0737, 0.1179)	0.0954 (0.0735, 0.1181)	0.0954 (0.0736, 0.118)	0.0954 (0.0736, 0.1179)	0.0954 (0.0735, 0.1179)	0.0953 (0.0734, 0.1179)	0.0953 (0.0735, 0.118)	0.0953 (0.0735, 0.1179)
2014	0.0365 (0.0185, 0.0539)	0.0365 (0.0184, 0.0539)	0.0365 (0.0185, 0.0538)	0.0365 (0.0185, 0.0539)	0.0365 (0.0184, 0.0539)	0.0365 (0.0185, 0.0538)	0.0365 (0.0185, 0.054)	0.0365 (0.0185, 0.0538)	0.0365 (0.0184, 0.0538)
2015	0.5945 (0.5599, 0.6297)	0.5943 (0.5598, 0.6296)	0.5944 (0.5599, 0.6295)	0.5944 (0.5599, 0.6296)	0.5944 (0.5601, 0.6296)	0.5946 (0.56, 0.6298)	0.594 (0.5597, 0.6291)	0.5944 (0.5597, 0.6296)	0.5943 (0.56, 0.6294)
2016	0.0912 (0.077, 0.1056)	0.0912 (0.077, 0.1057)	0.0912 (0.0769, 0.1057)	0.0912 (0.077, 0.1056)	0.0912 (0.077, 0.1056)	0.0912 (0.077, 0.1057)	0.0912 (0.077, 0.1057)	0.0912 (0.0769, 0.1056)	0.0912 (0.077, 0.1057)

S35 Table. Estimated coefficients for the best fitting model for prostate cancer morbidity.

Covariate	Parameter	Mean	SD	%2.5	%97.5
Intercept	β_0	-5.8730	0.0024	-5.8770	-5.8680
Age	β_{1,age_1}	-3.0200	0.0124	-3.0480	-2.9980
	β_{1,age_2}	-1.6280	0.0139	-1.6520	-1.5940
	β_{1,age_3}	-0.5533	0.0066	-0.5640	-0.5379
	β_{1,age_4}	0.1284	0.0069	0.1162	0.1420
	β_{1,age_5}	0.6558	0.0060	0.6447	0.6680
	β_{1,age_6}	0.8885	0.0049	0.8786	0.8974
	β_{1,age_7}	1.0420	0.0055	1.0310	1.0530
	β_{1,age_8}	1.0320	0.0069	1.0180	1.0450
	β_{1,age_9}	1.4540	0.0075	1.4410	1.4680
Year	β_2	0.1082	0.0040	0.0992	0.1157
Year^2	β_3	-0.0244	0.0027	-0.0300	-0.0199
Year^3	β_4	-0.0161	0.0022	-0.0198	-0.0112
Region	$\beta_{5,\text{region}_1}$	-0.1277	0.0095	-0.1473	-0.1108
	$\beta_{5,\text{region}_2}$	-0.0144	0.0079	-0.0277	0.0018
	$\beta_{5,\text{region}_3}$	-0.0238	0.0081	-0.0396	-0.0087
	$\beta_{5,\text{region}_4}$	-0.0201	0.0077	-0.0337	-0.0038
	$\beta_{5,\text{region}_5}$	0.0447	0.0079	0.0300	0.0601
	$\beta_{5,\text{region}_6}$	0.0257	0.0075	0.0121	0.0401
	$\beta_{5,\text{region}_7}$	0.0946	0.0069	0.0819	0.1073
	$\beta_{5,\text{region}_8}$	-0.0189	0.0072	-0.0315	-0.0040
	$\beta_{5,\text{region}_9}$	0.0398	0.0120	0.0191	0.0600
Deprivation	$\beta_{6,\text{deprivation}_1}$	-0.1276	0.0062	-0.1401	-0.1155
	$\beta_{6,\text{deprivation}_2}$	-0.0890	0.0048	-0.0983	-0.0803
	$\beta_{6,\text{deprivation}_3}$	-0.0613	0.0051	-0.0714	-0.0513
	$\beta_{6,\text{deprivation}_4}$	-0.0338	0.0049	-0.0428	-0.0244
	$\beta_{6,\text{deprivation}_5}$	-0.0083	0.0046	-0.0181	0.0006
	$\beta_{6,\text{deprivation}_6}$	0.0199	0.0044	0.0111	0.0281
	$\beta_{6,\text{deprivation}_7}$	0.0472	0.0046	0.0374	0.0557
	$\beta_{6,\text{deprivation}_8}$	0.0636	0.0044	0.0551	0.0723
	$\beta_{6,\text{deprivation}_9}$	0.0838	0.0049	0.0713	0.0926
	$\beta_{6,\text{deprivation}_{10}}$	0.1056	0.0046	0.0960	0.1135
Age:Year	β_{7,age_1}	0.2215	0.0123	0.1962	0.2431
	β_{7,age_2}	0.1246	0.0079	0.1111	0.1424
	β_{7,age_3}	0.0759	0.0051	0.0646	0.0863
	β_{7,age_4}	0.0100	0.0042	0.0023	0.0185
	β_{7,age_5}	-0.0035	0.0036	-0.0102	0.0034
	β_{7,age_6}	-0.0332	0.0038	-0.0416	-0.0261
	β_{7,age_7}	-0.0643	0.0040	-0.0711	-0.0559
	β_{7,age_8}	-0.1544	0.0042	-0.1622	-0.1463
	β_{7,age_9}	-0.1767	0.0044	-0.1850	-0.1676
Age:Region	$\beta_{8,\text{age}_1,\text{region}_1}$	0.0561	0.0432	-0.0392	0.1453
	$\beta_{8,\text{age}_1,\text{region}_2}$	-0.0631	0.0408	-0.1379	0.0215
	$\beta_{8,\text{age}_1,\text{region}_3}$	-0.1152	0.0433	-0.1989	-0.0350
	$\beta_{8,\text{age}_1,\text{region}_4}$	0.0175	0.0477	-0.0740	0.1135
	$\beta_{8,\text{age}_1,\text{region}_5}$	0.0619	0.0387	-0.0163	0.1388
	$\beta_{8,\text{age}_1,\text{region}_6}$	0.0185	0.0365	-0.0578	0.0833
	$\beta_{8,\text{age}_1,\text{region}_7}$	0.2269	0.0308	0.1687	0.2832
	$\beta_{8,\text{age}_1,\text{region}_8}$	0.0132	0.0303	-0.0396	0.0762
	$\beta_{8,\text{age}_1,\text{region}_9}$	-0.2157	0.0572	-0.3094	-0.1039
	$\beta_{8,\text{age}_2,\text{region}_1}$	-0.0157	0.0376	-0.0860	0.0695
	$\beta_{8,\text{age}_2,\text{region}_2}$	-0.0019	0.0226	-0.0518	0.0306
	$\beta_{8,\text{age}_2,\text{region}_3}$	0.0490	0.0217	-0.0107	0.0817
	$\beta_{8,\text{age}_2,\text{region}_4}$	-0.0953	0.0217	-0.1333	-0.0499
	$\beta_{8,\text{age}_2,\text{region}_5}$	0.0085	0.0166	-0.0264	0.0387
	$\beta_{8,\text{age}_2,\text{region}_6}$	-0.0683	0.0158	-0.0952	-0.0345
	$\beta_{8,\text{age}_2,\text{region}_7}$	0.1456	0.0171	0.1143	0.1813
	$\beta_{8,\text{age}_2,\text{region}_8}$	-0.0097	0.0169	-0.0456	0.0200
	$\beta_{8,\text{age}_2,\text{region}_9}$	-0.0121	0.0160	-0.0451	0.0189
	$\beta_{8,\text{age}_3,\text{region}_1}$	-0.0198	0.0187	-0.0553	0.0154
	$\beta_{8,\text{age}_3,\text{region}_2}$	-0.0128	0.0126	-0.0386	0.0141
	$\beta_{8,\text{age}_3,\text{region}_3}$	0.0374	0.0127	0.0146	0.0650
Age:Region	$\beta_{8,\text{age}_3,\text{region}_4}$	-0.0278	0.0162	-0.0621	-0.0020
	$\beta_{8,\text{age}_3,\text{region}_5}$	-0.0156	0.0148	-0.0461	0.0104
	$\beta_{8,\text{age}_3,\text{region}_6}$	-0.0519	0.0176	-0.0887	-0.0199
	$\beta_{8,\text{age}_3,\text{region}_7}$	0.0499	0.0117	0.0296	0.0754
	$\beta_{8,\text{age}_3,\text{region}_8}$	0.0504	0.0115	0.0290	0.0731
	$\beta_{8,\text{age}_3,\text{region}_9}$	-0.0100	0.0145	-0.0373	0.0181
	$\beta_{8,\text{age}_4,\text{region}_1}$	-0.0200	0.0148	-0.0476	0.0095
	$\beta_{8,\text{age}_4,\text{region}_2}$	-0.0172	0.0110	-0.0397	0.0032
	$\beta_{8,\text{age}_4,\text{region}_3}$	0.0079	0.0117	-0.0159	0.0334
	$\beta_{8,\text{age}_4,\text{region}_4}$	-0.0435	0.0130	-0.0717	-0.0169
	$\beta_{8,\text{age}_4,\text{region}_5}$	-0.0189	0.0130	-0.0428	0.0044
	$\beta_{8,\text{age}_4,\text{region}_6}$	0.0140	0.0100	-0.0031	0.0338
	$\beta_{8,\text{age}_4,\text{region}_7}$	0.0333	0.0120	0.0082	0.0573
	$\beta_{8,\text{age}_4,\text{region}_8}$	0.0240	0.0103	0.0056	0.0468
	$\beta_{8,\text{age}_4,\text{region}_9}$	0.0203	0.0117	-0.0006	0.0431
	$\beta_{8,\text{age}_5,\text{region}_1}$	-0.0410	0.0150	-0.0678	-0.0129
	$\beta_{8,\text{age}_5,\text{region}_2}$	-0.0165	0.0104	-0.0372	0.0021
	$\beta_{8,\text{age}_5,\text{region}_3}$	0.0058	0.0104	-0.0140	0.0282
	$\beta_{8,\text{age}_5,\text{region}_4}$	-0.0009	0.0118	-0.0226	0.0245
	$\beta_{8,\text{age}_5,\text{region}_5}$	0.0039	0.0105	-0.0158	0.0224
	$\beta_{8,\text{age}_5,\text{region}_6}$	0.0118	0.0103	-0.0087	0.0302
	$\beta_{8,\text{age}_5,\text{region}_7}$	-0.0150	0.0088	-0.0324	0.0028
	$\beta_{8,\text{age}_5,\text{region}_8}$	0.0378	0.0077	0.0231	0.0535
	$\beta_{8,\text{age}_5,\text{region}_9}$	0.0142	0.0120	-0.0078	0.0374
	$\beta_{8,\text{age}_6,\text{region}_1}$	-0.0290	0.0135	-0.0550	-0.0041
	$\beta_{8,\text{age}_6,\text{region}_2}$	0.0193	0.0099	-0.0037	0.0367
	$\beta_{8,\text{age}_6,\text{region}_3}$	-0.0100	0.0084	-0.0263	0.0076
	$\beta_{8,\text{age}_6,\text{region}_4}$	-0.0190	0.0116	-0.0411	0.0024
	$\beta_{8,\text{age}_6,\text{region}_5}$	0.0105	0.0090	-0.0080	0.0263
	$\beta_{8,\text{age}_6,\text{region}_6}$	0.0130	0.0103	-0.0082	0.0357
	$\beta_{8,\text{age}_6,\text{region}_7}$	-0.0275	0.0108	-0.0515	-0.0053
	$\beta_{8,\text{age}_6,\text{region}_8}$	0.0326	0.0084	0.0153	0.0488
	$\beta_{8,\text{age}_6,\text{region}_9}$	0.0100	0.0106	-0.0100	0.0296
	$\beta_{8,\text{age}_7,\text{region}_1}$	-0.0060	0.0129	-0.0340	0.0162
	$\beta_{8,\text{age}_7,\text{region}_2}$	0.0237	0.0101	0.0041	0.0427
	$\beta_{8,\text{age}_7,\text{region}_3}$	-0.0088	0.0119	-0.0310	0.0166
	$\beta_{8,\text{age}_7,\text{region}_4}$	0.0384	0.0109	0.0174	0.0605
	$\beta_{8,\text{age}_7,\text{region}_5}$	0.0166	0.0114	-0.0050	0.0403
	$\beta_{8,\text{age}_7,\text{region}_6}$	0.0379	0.0102	0.0158	0.0560
	$\beta_{8,\text{age}_7,\text{region}_7}$	-0.0876	0.0103	-0.1070	-0.0666
	$\beta_{8,\text{age}_7,\text{region}_8}$	-0.0350	0.0100	-0.0544	-0.0156
	$\beta_{8,\text{age}_7,\text{region}_9}$	0.0208	0.0133	-0.0057	0.0474
	$\beta_{8,\text{age}_8,\text{region}_1}$	0.0861	0.0169	0.0516	0.1173
	$\beta_{8,\text{age}_8,\text{region}_2}$	0.0362	0.0113	0.0167	0.0590
	$\beta_{8,\text{age}_8,\text{region}_3}$	0.0255	0.0119	0.0045	0.0494
	$\beta_{8,\text{age}_8,\text{region}_4}$	0.0198	0.0121	-0.0097	0.0397
	$\beta_{8,\text{age}_8,\text{region}_5}$	0.0073	0.0107	-0.0139	0.0303
	$\beta_{8,\text{age}_8,\text{region}_6}$	0.0392	0.0129	0.0145	0.0646
	$\beta_{8,\text{age}_8,\text{region}_7}$	-0.1804	0.0128	-0.2036	-0.1551
	$\beta_{8,\text{age}_8,\text{region}_8}$	-0.0958	0.0105	-0.1148	-0.0741
	$\beta_{8,\text{age}_8,\text{region}_9}$	0.0621	0.0125	0.0381	0.0851
	$\beta_{8,\text{age}_9,\text{region}_1}$	-0.0108	0.0184	-0.0465	0.0278
	$\beta_{8,\text{age}_9,\text{region}_2}$	0.0323	0.0124	0.0040	0.0538
	$\beta_{8,\text{age}_9,\text{region}_3}$	0.0083	0.0142	-0.0155	0.0373
	$\beta_{8,\text{age}_9,\text{region}_4}$	0.1108	0.0141	0.0847	0.1386
	$\beta_{8,\text{age}_9,\text{region}_5}$	-0.0742	0.0128	-0.0981	-0.0475
	$\beta_{8,\text{age}_9,\text{region}_6}$	-0.0142	0.0126	-0.0379	0.0115
	$\beta_{8,\text{age}_9,\text{region}_7}$	-0.1452	0.0126	-0.1687	-0.1217
	$\beta_{8,\text{age}_9,\text{region}_8}$	-0.0175	0.0098	-0.0386	0.0012
	$\beta_{8,\text{age}_9,\text{region}_9}$	0.1105	0.0140	0.0836	0.1370
Region:Year	$\beta_{9,\text{region}_1}$	-0.0419	0.0058	-0.0521	-0.0297
	$\beta_{9,\text{region}_2}$	-0.0161	0.0036	-0.0229	-0.0090

Covariate	Parameter	Mean	SD	%2.5	%97.5	Covariate	Parameter	Mean	SD	%2.5	%97.5
Region:Year	$\beta_{9,\text{region}_3}$	-0.0133	0.0043	-0.0220	-0.0048	Region:Deprivation	$\beta_{10,\text{deprivation}_7,\text{region}_3}$	0.0126	0.0117	-0.0090	0.0366
	$\beta_{9,\text{region}_4}$	0.0271	0.0046	0.0184	0.0370		$\beta_{10,\text{deprivation}_7,\text{region}_4}$	0.0122	0.0119	-0.0127	0.0345
	$\beta_{9,\text{region}_5}$	-0.0176	0.0043	-0.0257	-0.0090		$\beta_{10,\text{deprivation}_7,\text{region}_5}$	-0.0259	0.0114	-0.0503	-0.0036
	$\beta_{9,\text{region}_6}$	0.0279	0.0036	0.0214	0.0357		$\beta_{10,\text{deprivation}_7,\text{region}_6}$	0.0165	0.0100	-0.0026	0.0356
	$\beta_{9,\text{region}_7}$	0.0248	0.0037	0.0178	0.0318		$\beta_{10,\text{deprivation}_7,\text{region}_7}$	-0.0652	0.0141	-0.0963	-0.0382
	$\beta_{9,\text{region}_8}$	0.0326	0.0035	0.0262	0.0395		$\beta_{10,\text{deprivation}_7,\text{region}_8}$	-0.0083	0.0090	-0.0248	0.0099
	$\beta_{9,\text{region}_9}$	-0.0234	0.0037	-0.0309	-0.0158		$\beta_{10,\text{deprivation}_7,\text{region}_9}$	0.0266	0.0106	0.0065	0.0491
Region:Deprivation	$\beta_{10,\text{deprivation}_1,\text{region}_1}$	-0.0042	0.0170	-0.0383	0.0263		$\beta_{10,\text{deprivation}_8,\text{region}_1}$	0.0226	0.0167	-0.0105	0.0541
	$\beta_{10,\text{deprivation}_1,\text{region}_2}$	-0.0186	0.0109	-0.0390	0.0038		$\beta_{10,\text{deprivation}_8,\text{region}_2}$	-0.0034	0.0095	-0.0214	0.0158
	$\beta_{10,\text{deprivation}_1,\text{region}_3}$	-0.0706	0.0128	-0.0956	-0.0457		$\beta_{10,\text{deprivation}_8,\text{region}_3}$	0.0361	0.0112	0.0157	0.0589
	$\beta_{10,\text{deprivation}_1,\text{region}_4}$	-0.0325	0.0179	-0.0695	0.0006		$\beta_{10,\text{deprivation}_8,\text{region}_4}$	0.0005	0.0111	-0.0225	0.0233
	$\beta_{10,\text{deprivation}_1,\text{region}_5}$	0.0371	0.0122	0.0136	0.0628		$\beta_{10,\text{deprivation}_8,\text{region}_5}$	-0.0114	0.0115	-0.0347	0.0123
	$\beta_{10,\text{deprivation}_1,\text{region}_6}$	-0.0020	0.0211	-0.0443	0.0402		$\beta_{10,\text{deprivation}_8,\text{region}_6}$	0.0039	0.0128	-0.0228	0.0295
	$\beta_{10,\text{deprivation}_1,\text{region}_7}$	0.1594	0.0132	0.1348	0.1865		$\beta_{10,\text{deprivation}_8,\text{region}_7}$	-0.0718	0.0130	-0.0977	-0.0457
	$\beta_{10,\text{deprivation}_1,\text{region}_8}$	0.0082	0.0219	-0.0319	0.0490		$\beta_{10,\text{deprivation}_8,\text{region}_8}$	0.0085	0.0092	-0.0088	0.0261
	$\beta_{10,\text{deprivation}_1,\text{region}_9}$	-0.0768	0.0245	-0.1204	-0.0219		$\beta_{10,\text{deprivation}_8,\text{region}_9}$	0.0149	0.0093	-0.0021	0.0330
	$\beta_{10,\text{deprivation}_2,\text{region}_1}$	-0.0056	0.0171	-0.0425	0.0256		$\beta_{10,\text{deprivation}_9,\text{region}_1}$	0.0125	0.0159	-0.0195	0.0430
	$\beta_{10,\text{deprivation}_2,\text{region}_2}$	-0.0182	0.0117	-0.0430	0.0036		$\beta_{10,\text{deprivation}_9,\text{region}_2}$	0.0072	0.0101	-0.0138	0.0280
	$\beta_{10,\text{deprivation}_2,\text{region}_3}$	-0.0497	0.0153	-0.0765	-0.0168		$\beta_{10,\text{deprivation}_9,\text{region}_3}$	0.0537	0.0122	0.0280	0.0754
	$\beta_{10,\text{deprivation}_2,\text{region}_4}$	-0.0183	0.0163	-0.0500	0.0130		$\beta_{10,\text{deprivation}_9,\text{region}_4}$	0.0185	0.0108	-0.0024	0.0413
	$\beta_{10,\text{deprivation}_2,\text{region}_5}$	0.0166	0.0124	-0.0061	0.0408		$\beta_{10,\text{deprivation}_9,\text{region}_5}$	-0.0345	0.0103	-0.0534	-0.0133
	$\beta_{10,\text{deprivation}_2,\text{region}_6}$	-0.0078	0.0160	-0.0397	0.0246		$\beta_{10,\text{deprivation}_9,\text{region}_6}$	0.0041	0.0103	-0.0173	0.0237
	$\beta_{10,\text{deprivation}_2,\text{region}_7}$	0.1116	0.0122	0.0897	0.1366		$\beta_{10,\text{deprivation}_9,\text{region}_7}$	-0.0594	0.0146	-0.0848	-0.0255
	$\beta_{10,\text{deprivation}_2,\text{region}_8}$	-0.0150	0.0127	-0.0388	0.0103		$\beta_{10,\text{deprivation}_9,\text{region}_8}$	-0.0129	0.0108	-0.0349	0.0088
	$\beta_{10,\text{deprivation}_2,\text{region}_9}$	-0.0136	0.0117	-0.0361	0.0093		$\beta_{10,\text{deprivation}_9,\text{region}_9}$	0.0109	0.0106	-0.0111	0.0291
	$\beta_{10,\text{deprivation}_3,\text{region}_1}$	-0.0498	0.0151	-0.0811	-0.0203		$\beta_{10,\text{deprivation}_{10},\text{region}_1}$	0.0232	0.0192	-0.0201	0.0568
	$\beta_{10,\text{deprivation}_3,\text{region}_2}$	0.0100	0.0110	-0.0122	0.0309		$\beta_{10,\text{deprivation}_{10},\text{region}_2}$	0.0141	0.0106	-0.0050	0.0351
	$\beta_{10,\text{deprivation}_3,\text{region}_3}$	-0.0262	0.0114	-0.0521	-0.0054		$\beta_{10,\text{deprivation}_{10},\text{region}_3}$	0.0599	0.0127	0.0370	0.0877
	$\beta_{10,\text{deprivation}_3,\text{region}_4}$	-0.0094	0.0147	-0.0391	0.0198		$\beta_{10,\text{deprivation}_{10},\text{region}_4}$	-0.0211	0.0150	-0.0513	0.0043
	$\beta_{10,\text{deprivation}_3,\text{region}_5}$	-0.0075	0.0138	-0.0316	0.0249		$\beta_{10,\text{deprivation}_{10},\text{region}_5}$	0.0478	0.0132	0.0257	0.0752
	$\beta_{10,\text{deprivation}_3,\text{region}_6}$	0.0206	0.0120	-0.0034	0.0431		$\beta_{10,\text{deprivation}_{10},\text{region}_6}$	-0.0300	0.0101	-0.0501	-0.0106
	$\beta_{10,\text{deprivation}_3,\text{region}_7}$	0.0461	0.0116	0.0266	0.0720		$\beta_{10,\text{deprivation}_{10},\text{region}_7}$	-0.0923	0.0138	-0.1182	-0.0659
	$\beta_{10,\text{deprivation}_3,\text{region}_8}$	0.0056	0.0134	-0.0214	0.0304		$\beta_{10,\text{deprivation}_{10},\text{region}_8}$	-0.0132	0.0094	-0.0293	0.0066
	$\beta_{10,\text{deprivation}_3,\text{region}_9}$	0.0106	0.0136	-0.0167	0.0367		$\beta_{10,\text{deprivation}_{10},\text{region}_9}$	0.0116	0.0118	-0.0107	0.0361
	$\beta_{10,\text{deprivation}_4,\text{region}_1}$	-0.0106	0.0160	-0.0394	0.0211	Deprivation:Year	$\beta_{11,\text{deprivation}_1}$	0.0257	0.0055	0.0147	0.0370
	$\beta_{10,\text{deprivation}_4,\text{region}_2}$	-0.0106	0.0109	-0.0327	0.0116		$\beta_{11,\text{deprivation}_2}$	0.0192	0.0052	0.0084	0.0283
	$\beta_{10,\text{deprivation}_4,\text{region}_3}$	-0.0308	0.0164	-0.0656	-0.0007		$\beta_{11,\text{deprivation}_3}$	0.0156	0.0044	0.0071	0.0246
	$\beta_{10,\text{deprivation}_4,\text{region}_4}$	0.0257	0.0121	0.0060	0.0536		$\beta_{11,\text{deprivation}_4}$	0.0012	0.0049	-0.0083	0.0098
	$\beta_{10,\text{deprivation}_4,\text{region}_5}$	-0.0095	0.0131	-0.0358	0.0150		$\beta_{11,\text{deprivation}_5}$	0.0013	0.0041	-0.0067	0.0091
	$\beta_{10,\text{deprivation}_4,\text{region}_6}$	-0.0228	0.0124	-0.0484	-0.0001		$\beta_{11,\text{deprivation}_6}$	-0.0028	0.0039	-0.0099	0.0046
	$\beta_{10,\text{deprivation}_4,\text{region}_7}$	0.0383	0.0103	0.0181	0.0593		$\beta_{11,\text{deprivation}_7}$	-0.0100	0.0040	-0.0180	-0.0024
	$\beta_{10,\text{deprivation}_4,\text{region}_8}$	0.0045	0.0124	-0.0200	0.0291		$\beta_{11,\text{deprivation}_8}$	-0.0123	0.0035	-0.0191	-0.0054
	$\beta_{10,\text{deprivation}_4,\text{region}_9}$	0.0158	0.0126	-0.0082	0.0408		$\beta_{11,\text{deprivation}_9}$	-0.0145	0.0040	-0.0222	-0.0067
	$\beta_{10,\text{deprivation}_5,\text{region}_1}$	0.0067	0.0174	-0.0257	0.0440		$\beta_{11,\text{deprivation}_{10}}$	-0.0235	0.0039	-0.0314	-0.0160
	$\beta_{10,\text{deprivation}_5,\text{region}_2}$	0.0082	0.0103	-0.0129	0.0283	Age : Year^2	β_{12,age_1}	-0.0256	0.0113	-0.0471	-0.0031
	$\beta_{10,\text{deprivation}_5,\text{region}_3}$	-0.0144	0.0111	-0.0351	0.0068		β_{12,age_2}	-0.0272	0.0109	-0.0512	-0.0068
	$\beta_{10,\text{deprivation}_5,\text{region}_4}$	0.0227	0.0137	-0.0050	0.0535		β_{12,age_3}	-0.0323	0.0051	-0.0428	-0.0239
	$\beta_{10,\text{deprivation}_5,\text{region}_5}$	-0.0044	0.0121	-0.0301	0.0179		β_{12,age_4}	-0.0196	0.0059	-0.0308	-0.0083
	$\beta_{10,\text{deprivation}_5,\text{region}_6}$	-0.0028	0.0114	-0.0255	0.0185		β_{12,age_5}	0.0059	0.0046	-0.0040	0.0145
	$\beta_{10,\text{deprivation}_5,\text{region}_7}$	-0.0186	0.0129	-0.0415	0.0090		β_{12,age_6}	0.0073	0.0041	0.0003	0.0166
	$\beta_{10,\text{deprivation}_5,\text{region}_8}$	0.0150	0.0111	-0.0066	0.0380		β_{12,age_7}	0.0324	0.0047	0.0236	0.0408
	$\beta_{10,\text{deprivation}_5,\text{region}_9}$	-0.0124	0.0110	-0.0328	0.0102		β_{12,age_8}	0.0230	0.0049	0.0137	0.0324
	$\beta_{10,\text{deprivation}_6,\text{region}_1}$	-0.0284	0.0227	-0.0741	0.0185		β_{12,age_9}	0.0361	0.0058	0.0266	0.0479
	$\beta_{10,\text{deprivation}_6,\text{region}_2}$	0.0134	0.0109	-0.0074	0.0348	Region : Year^2	$\beta_{13,\text{region}_1}$	0.0358	0.0060	0.0243	0.0478
	$\beta_{10,\text{deprivation}_6,\text{region}_3}$	0.0295	0.0135	0.0043	0.0559		$\beta_{13,\text{region}_2}$	-0.0200	0.0044	-0.0284	-0.0120
	$\beta_{10,\text{deprivation}_6,\text{region}_4}$	0.0017	0.0127	-0.0273	0.0264		$\beta_{13,\text{region}_3}$	-0.0187	0.0049	-0.0284	-0.0101
	$\beta_{10,\text{deprivation}_6,\text{region}_5}$	-0.0083	0.0112	-0.0309	0.0131		$\beta_{13,\text{region}_4}$	-0.0380	0.0067	-0.0521	-0.0269
	$\beta_{10,\text{deprivation}_6,\text{region}_6}$	0.0202	0.0110	-0.0014	0.0399		$\beta_{13,\text{region}_5}$	-0.0008	0.0050	-0.0119	0.0085
	$\beta_{10,\text{deprivation}_6,\text{region}_7}$	-0.0481	0.0139	-0.0770	-0.0215		$\beta_{13,\text{region}_6}$	0.0098	0.0049	-0.0007	0.0195
	$\beta_{10,\text{deprivation}_6,\text{region}_8}$	0.0077	0.0106	-0.0128	0.0303		$\beta_{13,\text{region}_7}$	-0.0007	0.0041	-0.0091	0.0077
	$\beta_{10,\text{deprivation}_6,\text{region}_9}$	0.0124	0.0099	-0.0078	0.0318		$\beta_{13,\text{region}_8}$	0.0338	0.0040	0.0253	0.0417
	$\beta_{10,\text{deprivation}_7,\text{region}_1}$	0.0336	0.0209	-0.0160	0.0716		$\beta_{13,\text{region}_9}$	-0.0012	0.0044	-0.0094	0.0076
	$\beta_{10,\text{deprivation}_7,\text{region}_2}$	-0.0022	0.0102	-0.0216	0.0173		σ^2	0.0025	0.0003	0.0019	0.0030

S36 Table. Estimated coefficients for the best fitting model for prostate cancer mortality.

Covariate	Parameter	Mean	SD	%2.5	%97.5	Covariate	Parameter	Mean	SD	%2.5	%97.5
Intercept	β_0	-7.3970	0.0070	-7.4110	-7.3840	Age^3	β_5	0.1427	0.0061	0.1308	0.1545
Age	β_1	1.9810	0.0093	1.9640	2.0010	$Year^2$	β_6	0.0096	0.0036	0.0028	0.0171
Year	β_2	-0.1067	0.0047	-0.1159	-0.0977	Age:Year	β_7	-0.0053	0.0082	-0.0215	0.0107
AAD	β_3	0.0186	0.0032	0.0123	0.0248	Age^2 : Year	β_8	0.0391	0.0053	0.0285	0.0492
Age^2	β_4	-0.3940	0.0099	-0.4122	-0.3741		σ^2	0.0022	0.0004	0.0015	0.0030

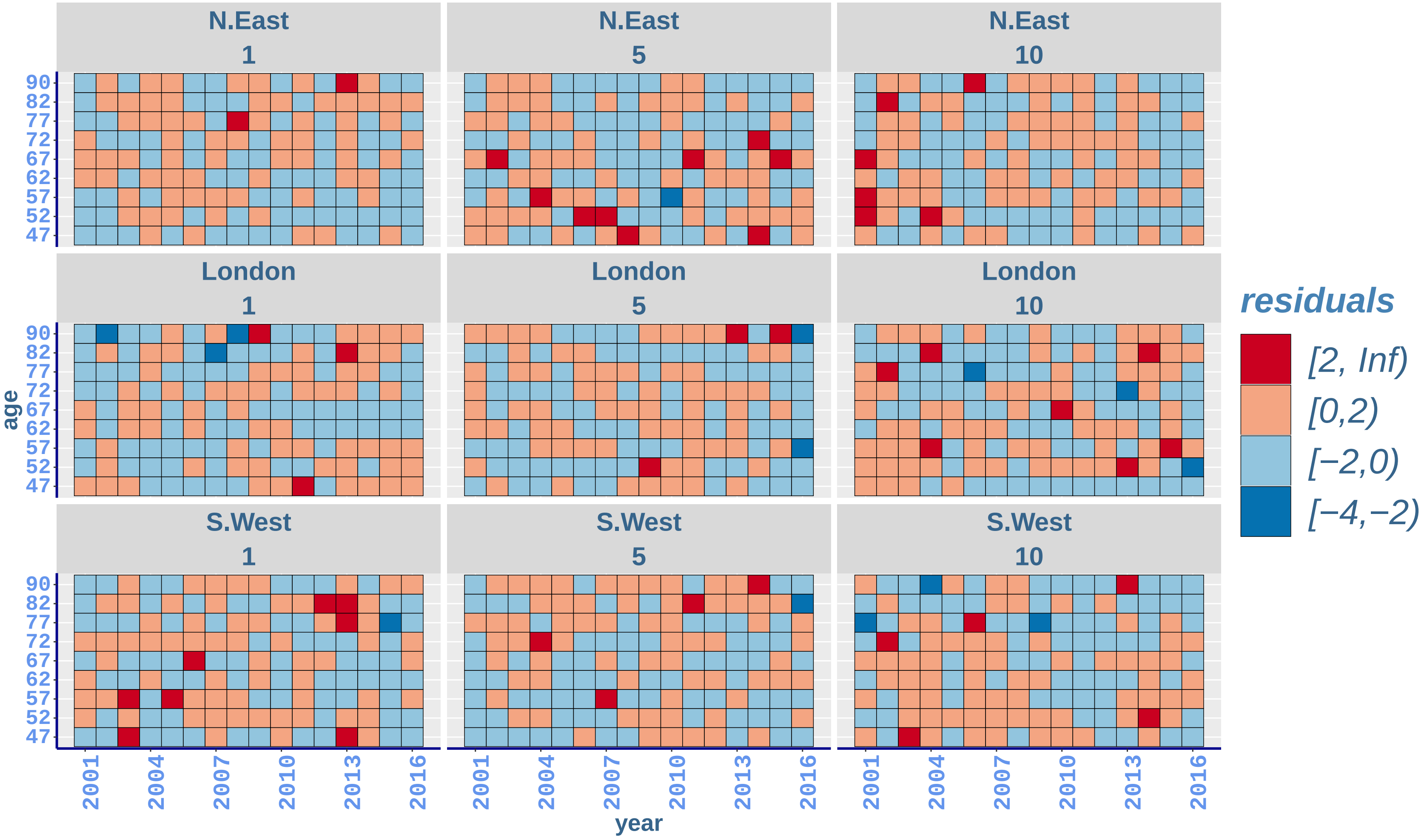

S85 Fig. Heat map of Pearson residuals for prostate cancer morbidity in North East, London, and South West, deprivation deciles 1, 5, and 10: orange/light blue cells indicate areas with good fit, while red/dark blue cells indicate areas with poor fit. Note that there is a small number of residuals greater than 4, and these are included in the last category.

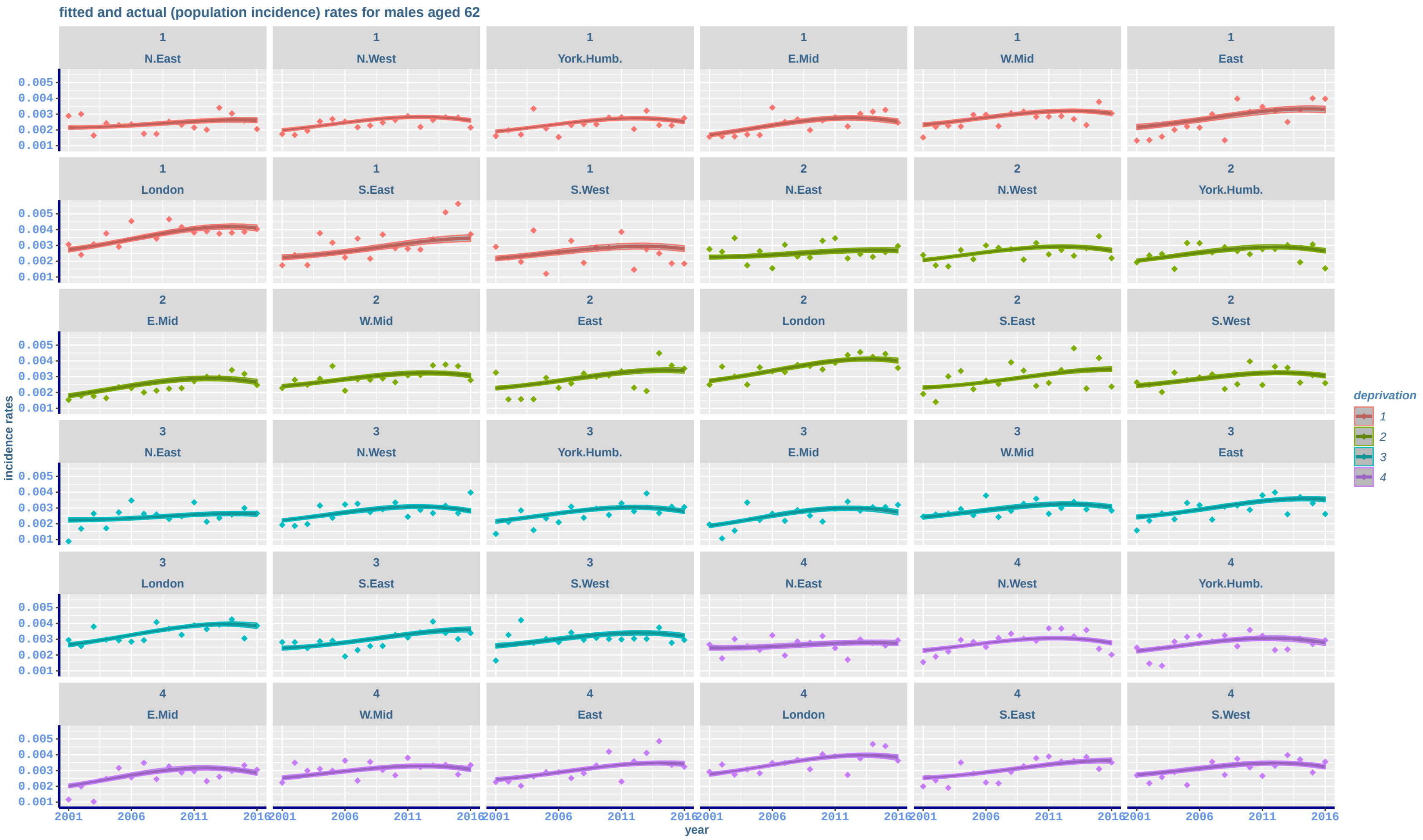

S86 Fig. Prostate cancer morbidity, age 62, deprivation deciles 1-4 for all regions in England between 2001 and 2006: observed rates (dots), fitted rates (lines), with 95% credible intervals for the fitted rates.

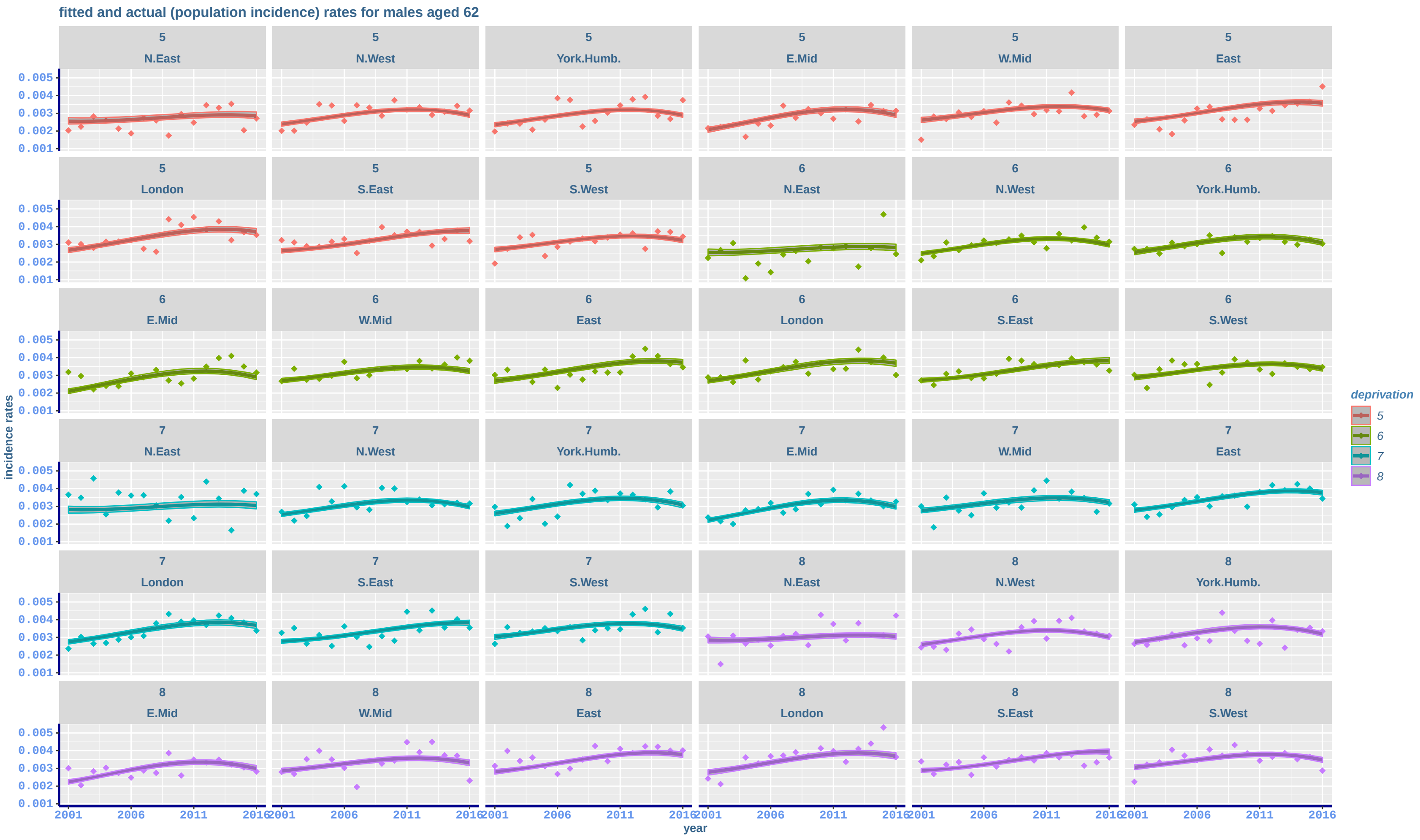

S87 Fig. Prostate cancer morbidity, age 62, deprivation deciles 5-8 for all regions in England between 2001 and 2006: observed rates (dots), fitted rates (lines), with 95% credible intervals for the fitted rates.

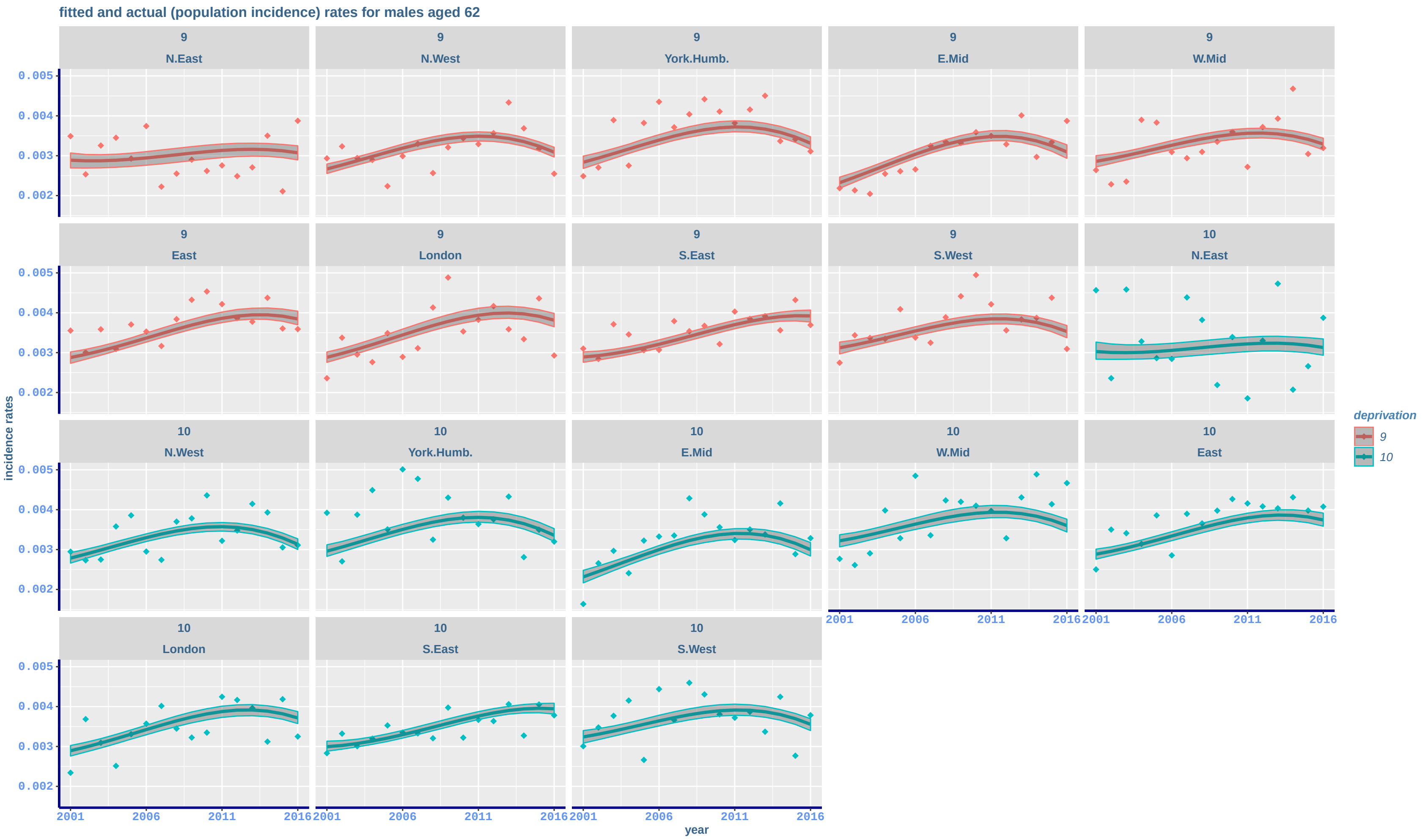

S88 Fig. Prostate cancer morbidity, age 62, deprivation deciles 9-10 for all regions in England between 2001 and 2006: observed rates (dots), fitted rates (lines), with 95% credible intervals for the fitted rates.

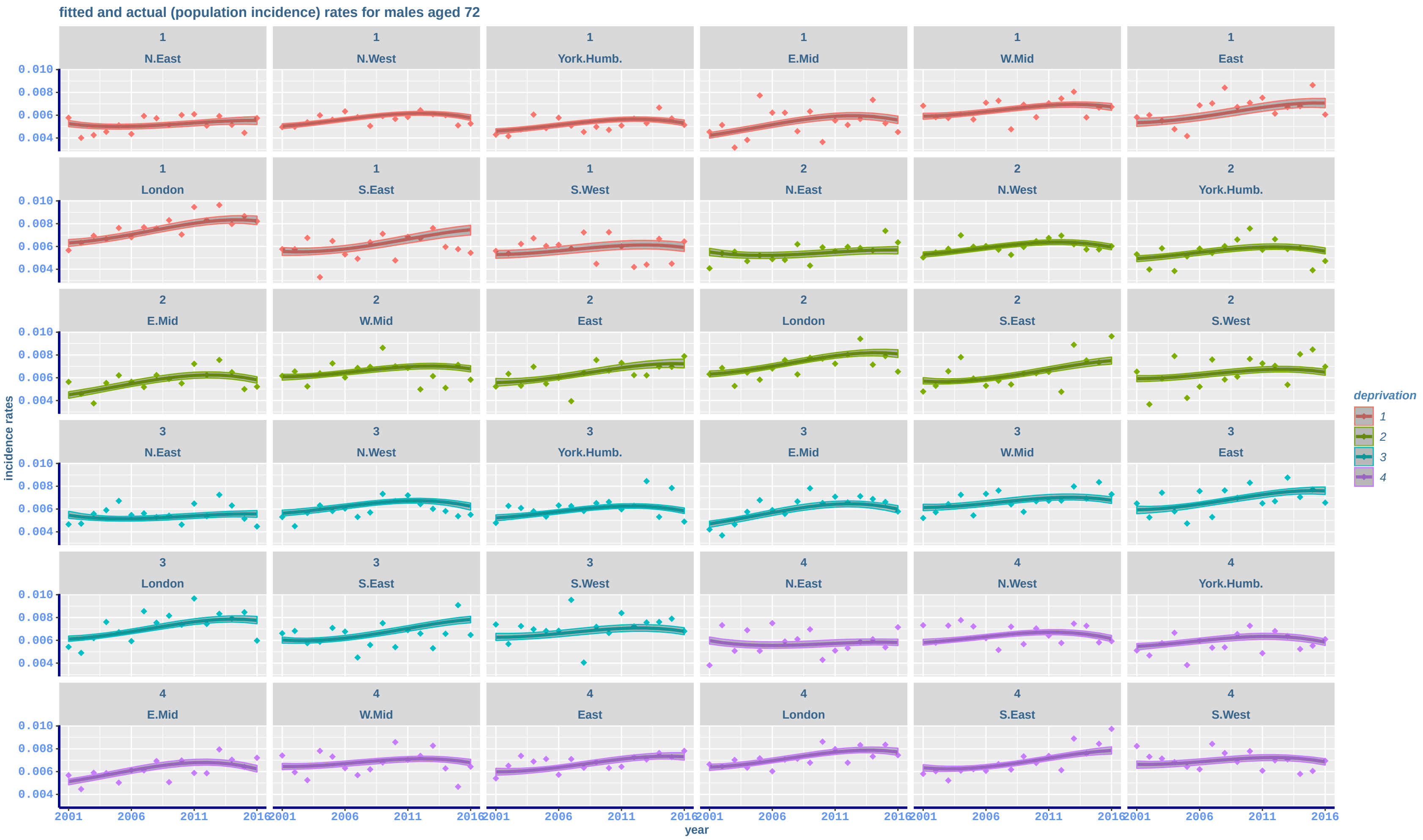

S89 Fig. Prostate cancer morbidity, age 72, deprivation deciles 1-4 for all regions in England between 2001 and 2016: observed rates (dots), fitted rates (lines), with 95% credible intervals for the fitted rates.

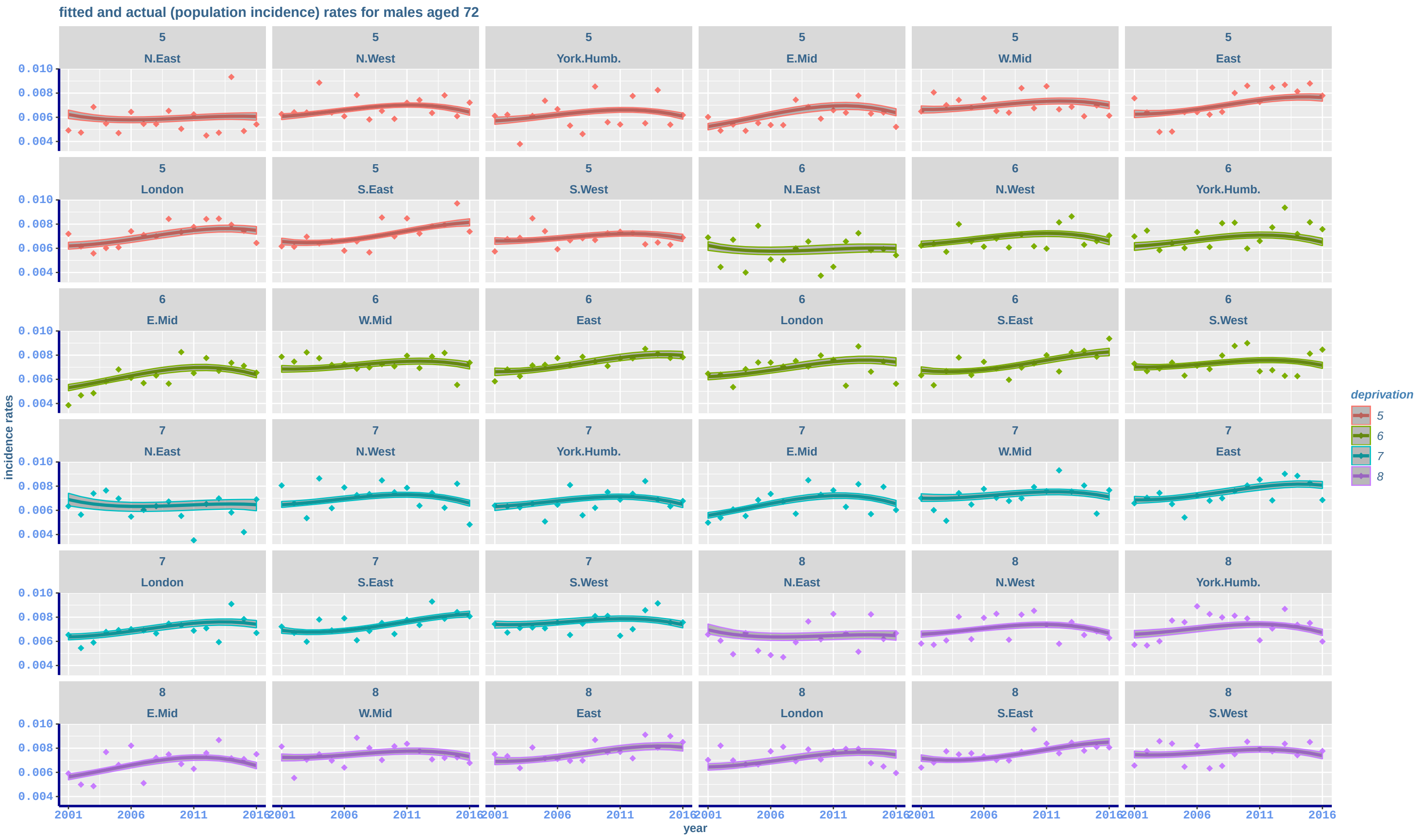

S90 Fig. Prostate cancer morbidity, age 72, deprivation deciles 5-8 for all regions in England between 2001 and 2016: observed rates (dots), fitted rates (lines), with 95% credible intervals for the fitted rates.

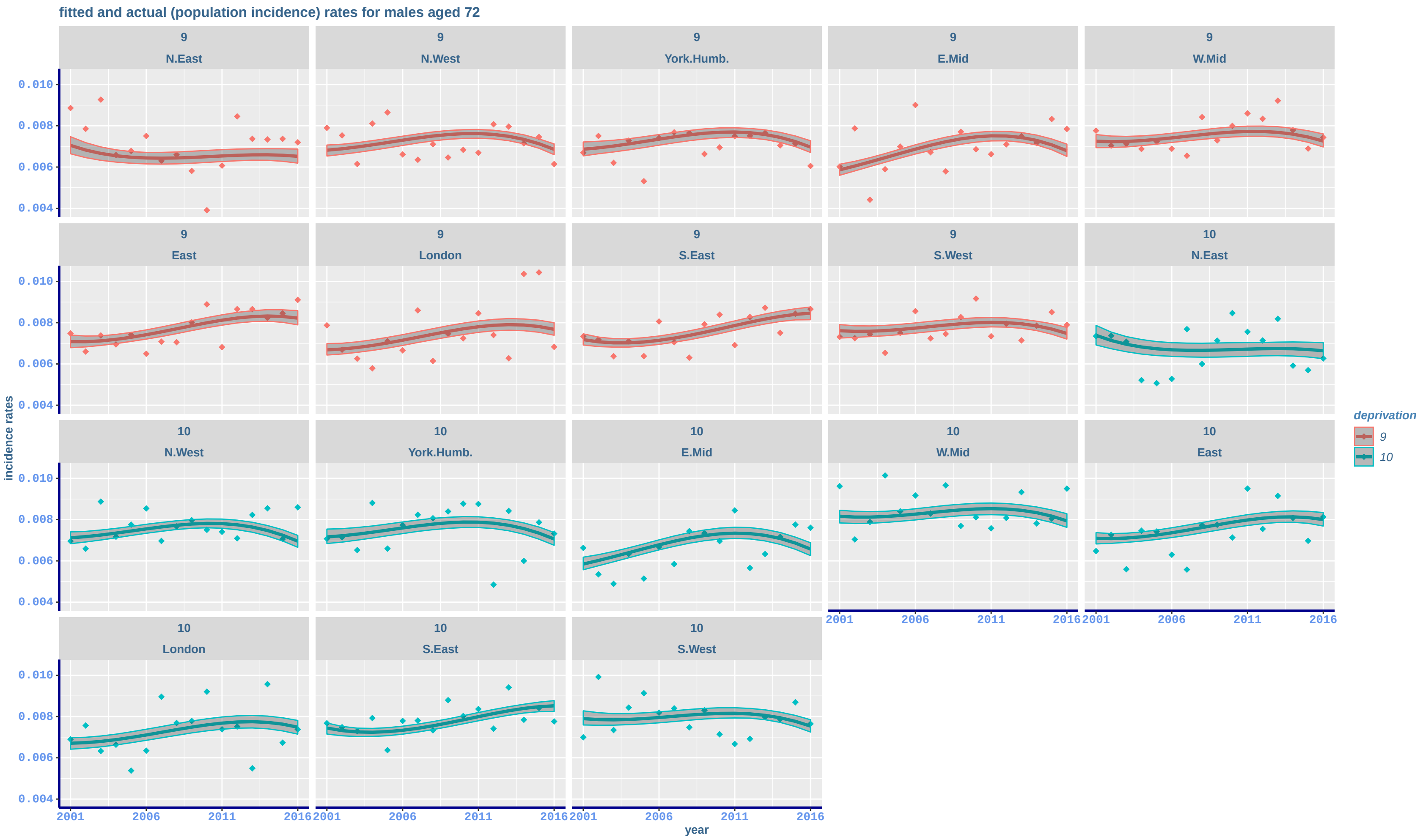

S91 Fig. Prostate cancer morbidity, age 72, deprivation deciles 9-10 for all regions in England between 2001 and 2016: observed rates (dots), fitted rates (lines), with 95% credible intervals for the fitted rates.

S37 Table. Absolute deprivation differences ($AD_{t,r}$), per 100,000 people, in age-standardised fitted incidence rates of malignant neoplasm of prostate from 2001 to 2016 for all regions in England for males; 95% credible intervals in brackets

year	N.East	N.West	York.Humb.	E.Mid	W.Mid	East	London	S.East	S.West
2001	134 (111, 162)	127 (112, 143)	159 (141, 176)	107 (92, 125)	136 (118, 156)	111 (93, 129)	40 (28, 53)	112 (97, 130)	165 (139, 190)
2002	125 (104, 150)	123 (109, 138)	155 (138, 171)	106 (91, 123)	130 (113, 150)	106 (89, 124)	37 (25, 51)	106 (91, 123)	158 (133, 182)
2003	117 (97, 140)	119 (106, 134)	152 (136, 167)	106 (91, 122)	125 (109, 144)	102 (85, 120)	35 (23, 48)	100 (86, 117)	152 (128, 175)
2004	111 (92, 132)	116 (103, 130)	150 (134, 164)	105 (90, 121)	120 (105, 139)	99 (81, 116)	33 (22, 46)	96 (82, 111)	147 (124, 170)
2005	105 (87, 126)	113 (101, 127)	147 (132, 161)	104 (89, 121)	116 (101, 134)	96 (79, 113)	32 (20, 45)	92 (79, 108)	143 (121, 166)
2006	101 (83, 121)	111 (98, 124)	145 (129, 159)	104 (89, 120)	113 (98, 129)	94 (76, 111)	31 (19, 44)	89 (76, 104)	140 (117, 161)
2007	97 (79, 116)	108 (95, 121)	143 (127, 157)	103 (88, 119)	109 (95, 125)	91 (74, 109)	31 (19, 44)	87 (73, 102)	136 (114, 158)
2008	93 (76, 113)	105 (93, 118)	140 (125, 154)	102 (86, 118)	106 (91, 121)	89 (72, 107)	32 (20, 45)	84 (71, 100)	133 (110, 155)
2009	90 (73, 110)	101 (90, 115)	137 (122, 151)	100 (85, 116)	102 (88, 117)	88 (70, 105)	33 (22, 47)	82 (69, 98)	129 (107, 151)
2010	87 (71, 106)	98 (86, 111)	134 (119, 148)	98 (83, 114)	98 (84, 113)	86 (68, 103)	36 (24, 51)	80 (66, 96)	125 (103, 148)
2011	84 (68, 104)	94 (82, 107)	130 (115, 145)	95 (80, 112)	95 (80, 109)	83 (66, 101)	38 (27, 55)	77 (64, 94)	121 (99, 144)
2012	82 (66, 101)	89 (77, 103)	126 (110, 140)	91 (76, 108)	90 (76, 105)	81 (63, 99)	41 (29, 58)	75 (61, 92)	117 (94, 139)
2013	79 (63, 98)	84 (72, 99)	121 (105, 136)	87 (72, 104)	86 (72, 100)	78 (61, 97)	45 (32, 62)	73 (58, 90)	112 (89, 134)
2014	76 (61, 95)	79 (67, 93)	115 (99, 130)	83 (68, 100)	81 (67, 96)	75 (58, 94)	48 (34, 66)	70 (55, 88)	107 (83, 129)
2015	73 (58, 92)	73 (61, 88)	108 (93, 123)	77 (63, 94)	76 (62, 91)	72 (55, 91)	51 (37, 69)	68 (51, 86)	101 (78, 124)
2016	69 (54, 88)	67 (56, 82)	101 (86, 117)	71 (57, 88)	71 (57, 86)	68 (51, 87)	53 (39, 72)	65 (48, 83)	95 (72, 117)

S38 Table. Relative deprivation differences ($\mathbf{RD}_{t,r}$) in age-standardised fitted incidence rates of malignant neoplasm of prostate from 2001 to 2016 for all regions in England for males; 95% credible intervals in brackets

year	N.East	N.West	York.Humb.	E.Mid	W.Mid	East	London	S.East	S.West
2001	0.4098 (0.3338, 0.4958)	0.4135 (0.3594, 0.4738)	0.5591 (0.4878, 0.6288)	0.3986 (0.3281, 0.4761)	0.385 (0.3267, 0.4542)	0.3408 (0.2729, 0.4162)	0.1126 (0.0759, 0.1523)	0.3461 (0.2852, 0.4198)	0.4951 (0.3964, 0.5938)
2002	0.3952 (0.3215, 0.4777)	0.3985 (0.3476, 0.4546)	0.5425 (0.4738, 0.6078)	0.385 (0.318, 0.4597)	0.3703 (0.3157, 0.4359)	0.3275 (0.2617, 0.401)	0.1049 (0.0697, 0.1436)	0.3319 (0.2745, 0.4021)	0.4792 (0.3833, 0.5745)
2003	0.3809 (0.3097, 0.4605)	0.3837 (0.3343, 0.4361)	0.5261 (0.4601, 0.5882)	0.3716 (0.3084, 0.4423)	0.3558 (0.3042, 0.4184)	0.3144 (0.2494, 0.3848)	0.0978 (0.0637, 0.1356)	0.3179 (0.263, 0.3843)	0.4636 (0.3711, 0.5563)
2004	0.3667 (0.2977, 0.4447)	0.369 (0.3221, 0.4191)	0.5099 (0.4462, 0.5686)	0.3584 (0.2978, 0.4273)	0.3416 (0.2933, 0.4012)	0.3017 (0.2383, 0.3704)	0.0914 (0.0587, 0.1291)	0.3041 (0.2512, 0.3675)	0.4481 (0.3577, 0.5378)
2005	0.3528 (0.2852, 0.429)	0.3545 (0.3097, 0.4022)	0.4939 (0.4322, 0.5508)	0.3456 (0.2871, 0.4121)	0.3275 (0.2817, 0.3846)	0.2893 (0.2276, 0.3558)	0.0863 (0.0529, 0.1229)	0.2905 (0.2401, 0.3524)	0.4328 (0.3454, 0.5198)
2006	0.3392 (0.2733, 0.4147)	0.3401 (0.2965, 0.3868)	0.4781 (0.4181, 0.5325)	0.333 (0.2757, 0.3975)	0.3137 (0.2689, 0.3679)	0.2773 (0.2169, 0.3424)	0.0827 (0.0493, 0.1195)	0.2771 (0.2284, 0.3369)	0.4177 (0.3329, 0.504)
2007	0.3258 (0.2617, 0.3991)	0.326 (0.2838, 0.3714)	0.4626 (0.4044, 0.5163)	0.3208 (0.2654, 0.3835)	0.3001 (0.2566, 0.3499)	0.2655 (0.2068, 0.3305)	0.081 (0.0484, 0.1171)	0.264 (0.2166, 0.3228)	0.4028 (0.3191, 0.4874)
2008	0.3128 (0.2499, 0.3846)	0.312 (0.2723, 0.3565)	0.4472 (0.3911, 0.4998)	0.3088 (0.2559, 0.3698)	0.2868 (0.2439, 0.3345)	0.254 (0.1968, 0.3165)	0.0813 (0.0496, 0.1172)	0.2511 (0.2054, 0.3089)	0.3882 (0.3048, 0.4718)
2009	0.3 (0.2393, 0.3716)	0.2983 (0.2594, 0.3417)	0.4321 (0.3773, 0.484)	0.2971 (0.2455, 0.3577)	0.2737 (0.2319, 0.3187)	0.2429 (0.1866, 0.3034)	0.0839 (0.0536, 0.1212)	0.2386 (0.1937, 0.2953)	0.3737 (0.2911, 0.4555)
2010	0.2877 (0.2288, 0.3583)	0.2848 (0.2466, 0.328)	0.4171 (0.3637, 0.4685)	0.2856 (0.2347, 0.3461)	0.2609 (0.2205, 0.3036)	0.2321 (0.1775, 0.2921)	0.0884 (0.0586, 0.128)	0.2263 (0.181, 0.2825)	0.3595 (0.2774, 0.4415)
2011	0.2757 (0.2186, 0.3451)	0.2714 (0.2332, 0.316)	0.4025 (0.3483, 0.4533)	0.2744 (0.2239, 0.3342)	0.2486 (0.2085, 0.2898)	0.2217 (0.1686, 0.2802)	0.094 (0.0642, 0.1351)	0.2144 (0.1698, 0.2701)	0.3456 (0.263, 0.4276)
2012	0.2641 (0.2077, 0.3335)	0.2583 (0.2196, 0.3033)	0.388 (0.3342, 0.4409)	0.2633 (0.2128, 0.3236)	0.2366 (0.1973, 0.2777)	0.2116 (0.1598, 0.2694)	0.1006 (0.0703, 0.144)	0.2028 (0.159, 0.2582)	0.332 (0.2494, 0.4131)
2013	0.2528 (0.1985, 0.3215)	0.2455 (0.2062, 0.2919)	0.3738 (0.3195, 0.428)	0.2524 (0.203, 0.3135)	0.225 (0.1859, 0.2661)	0.2018 (0.1512, 0.2586)	0.108 (0.0758, 0.1533)	0.1917 (0.1474, 0.2471)	0.3188 (0.2366, 0.3993)
2014	0.242 (0.1897, 0.3093)	0.2331 (0.1943, 0.2801)	0.3599 (0.305, 0.4156)	0.2416 (0.1917, 0.3032)	0.214 (0.1758, 0.255)	0.1924 (0.1434, 0.2481)	0.1158 (0.0817, 0.1625)	0.181 (0.1357, 0.2356)	0.306 (0.2238, 0.3876)
2015	0.2316 (0.18, 0.2991)	0.2209 (0.182, 0.2698)	0.3462 (0.2922, 0.403)	0.2311 (0.1813, 0.2929)	0.2034 (0.1654, 0.2456)	0.1833 (0.1354, 0.2382)	0.1241 (0.0884, 0.1722)	0.1707 (0.1253, 0.2245)	0.2935 (0.2121, 0.375)
2016	0.2216 (0.1693, 0.2881)	0.2091 (0.1704, 0.2595)	0.3329 (0.278, 0.3914)	0.2207 (0.1706, 0.2823)	0.1935 (0.1552, 0.2367)	0.1745 (0.1268, 0.2283)	0.1328 (0.0953, 0.1819)	0.161 (0.1146, 0.2142)	0.2813 (0.2005, 0.3616)

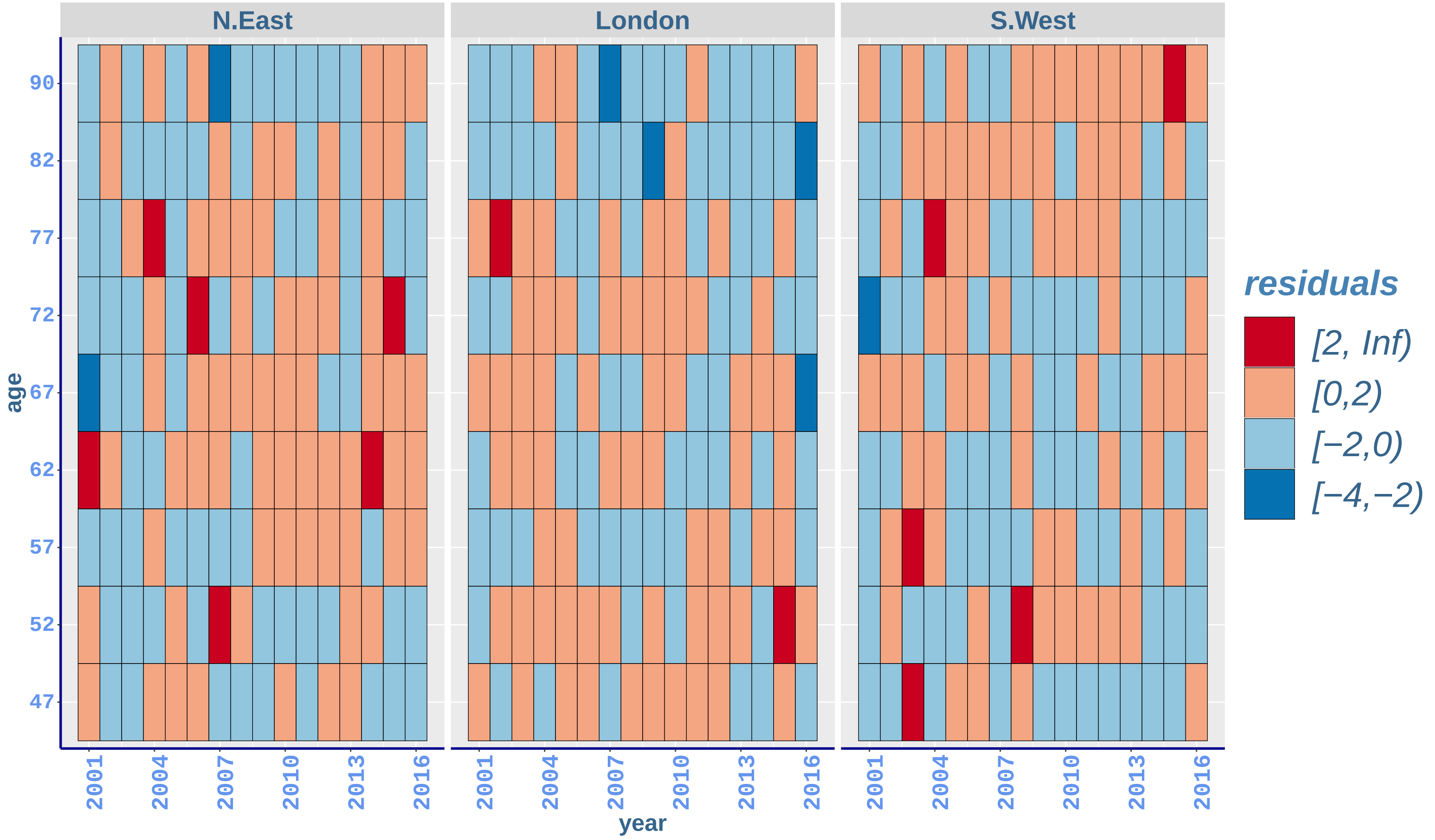

S92 Fig. Heat map of Pearson residuals for prostate cancer mortality in North East, London, and South West, deprivation deciles 1, 5, and 10: orange/light blue cells indicate areas with good fit, while red/dark blue cells indicate areas with poor fit. Note that there is a small number of residuals greater than 4, and these are included in the last category.

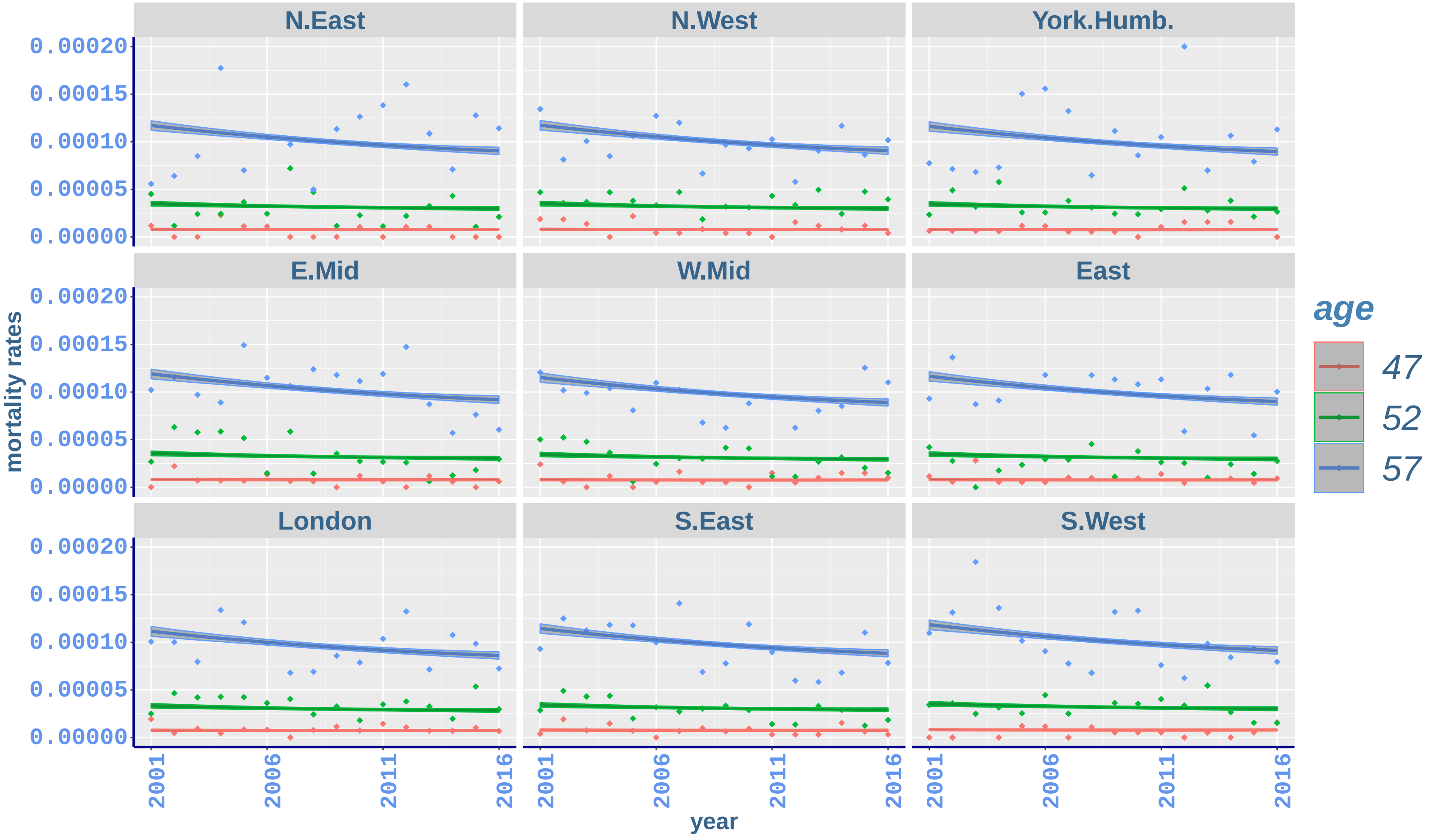

S93 Fig. Prostate cancer mortality, ages 47-57 for all regions in England between 2001 and 2006: observed rates (dots), fitted rates (lines), with 95% credible intervals for the fitted rates.

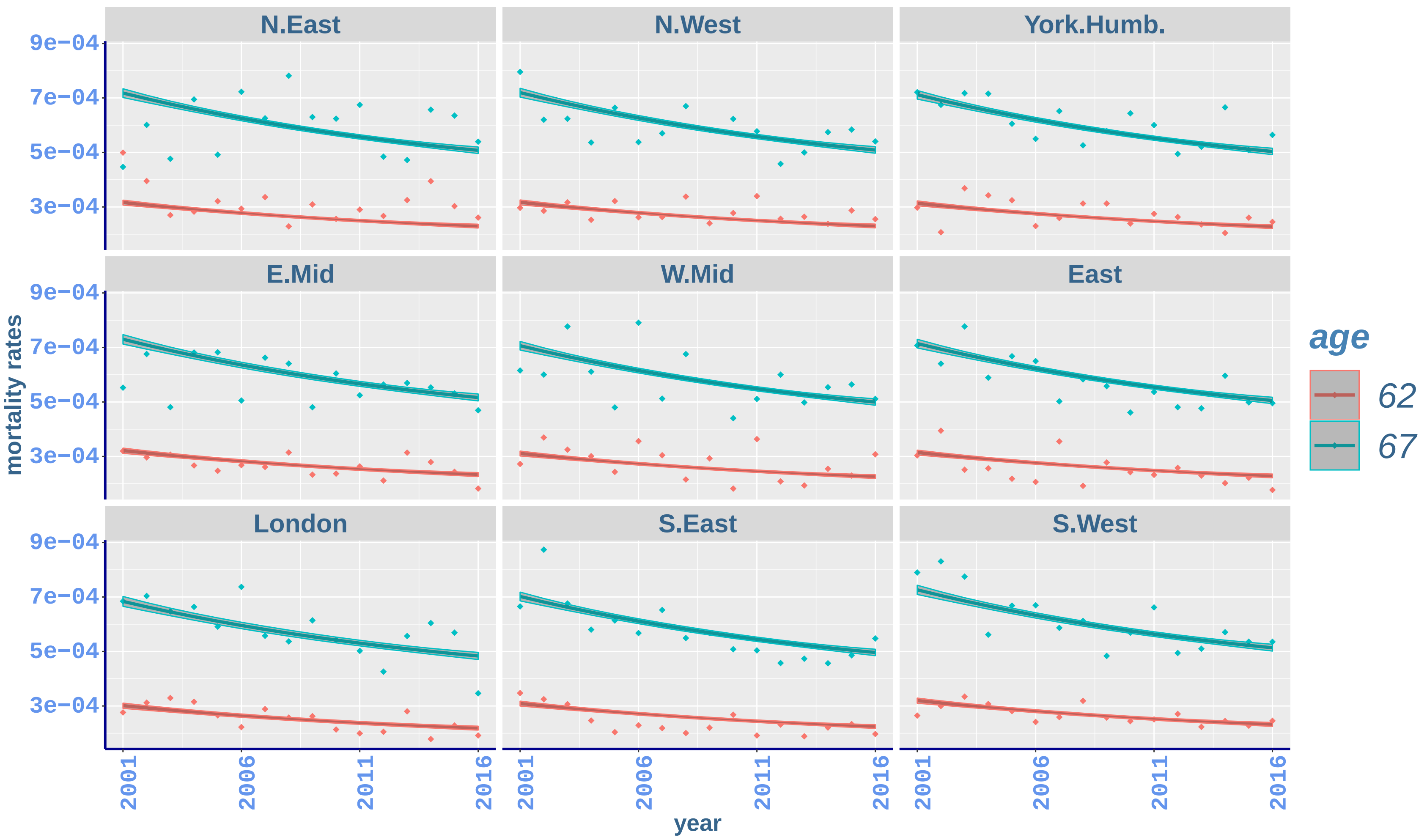

S94 Fig. Prostate cancer mortality, ages 62-67 for all regions in England between 2001 and 2016: observed rates (dots), fitted rates (lines), with 95% credible intervals for the fitted rates.

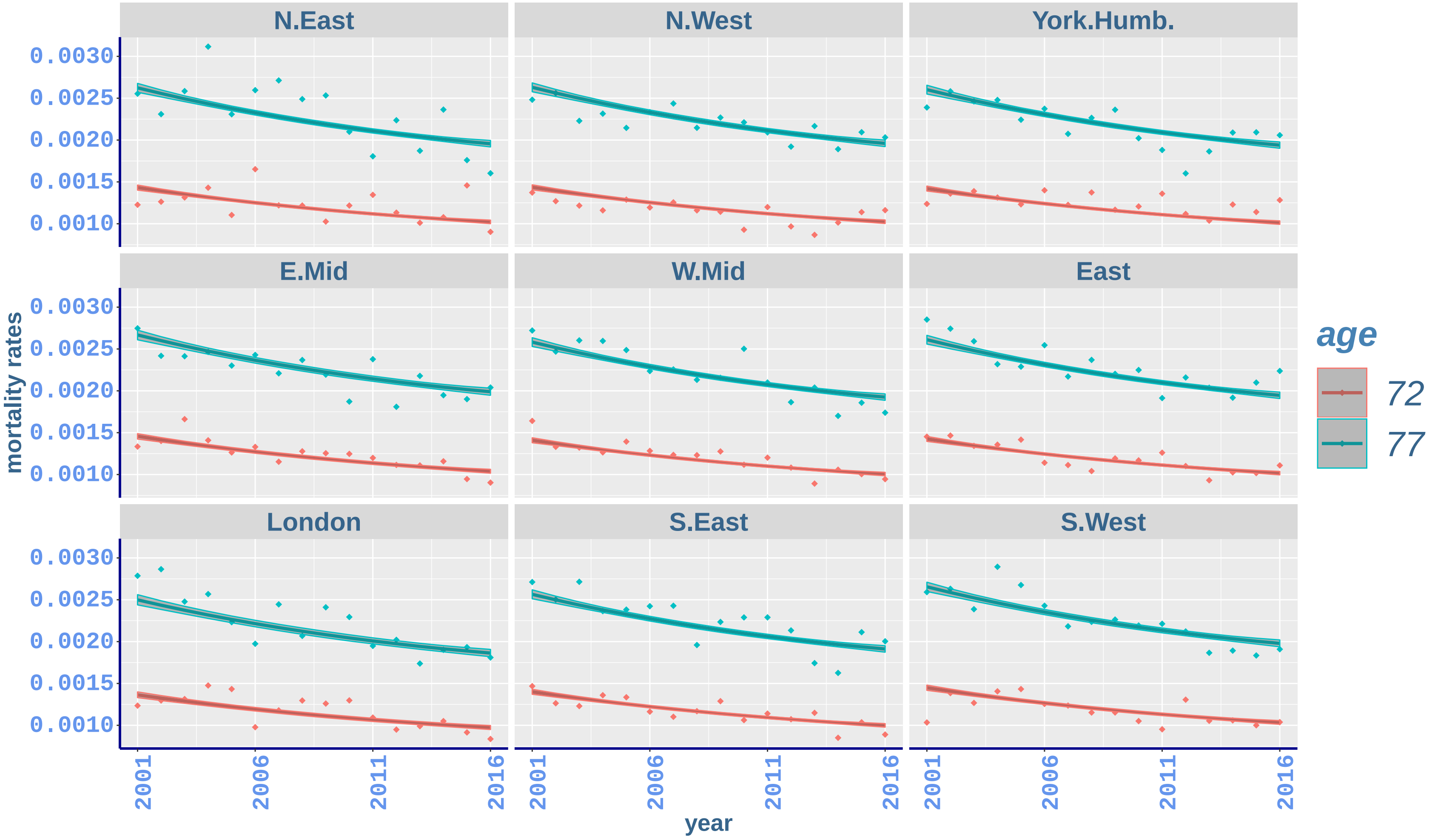

S95 Fig. Prostate cancer mortality, ages 72-77 for all regions in England between 2001 and 2016: observed rates (dots), fitted rates (lines), with 95% credible intervals for the fitted rates.

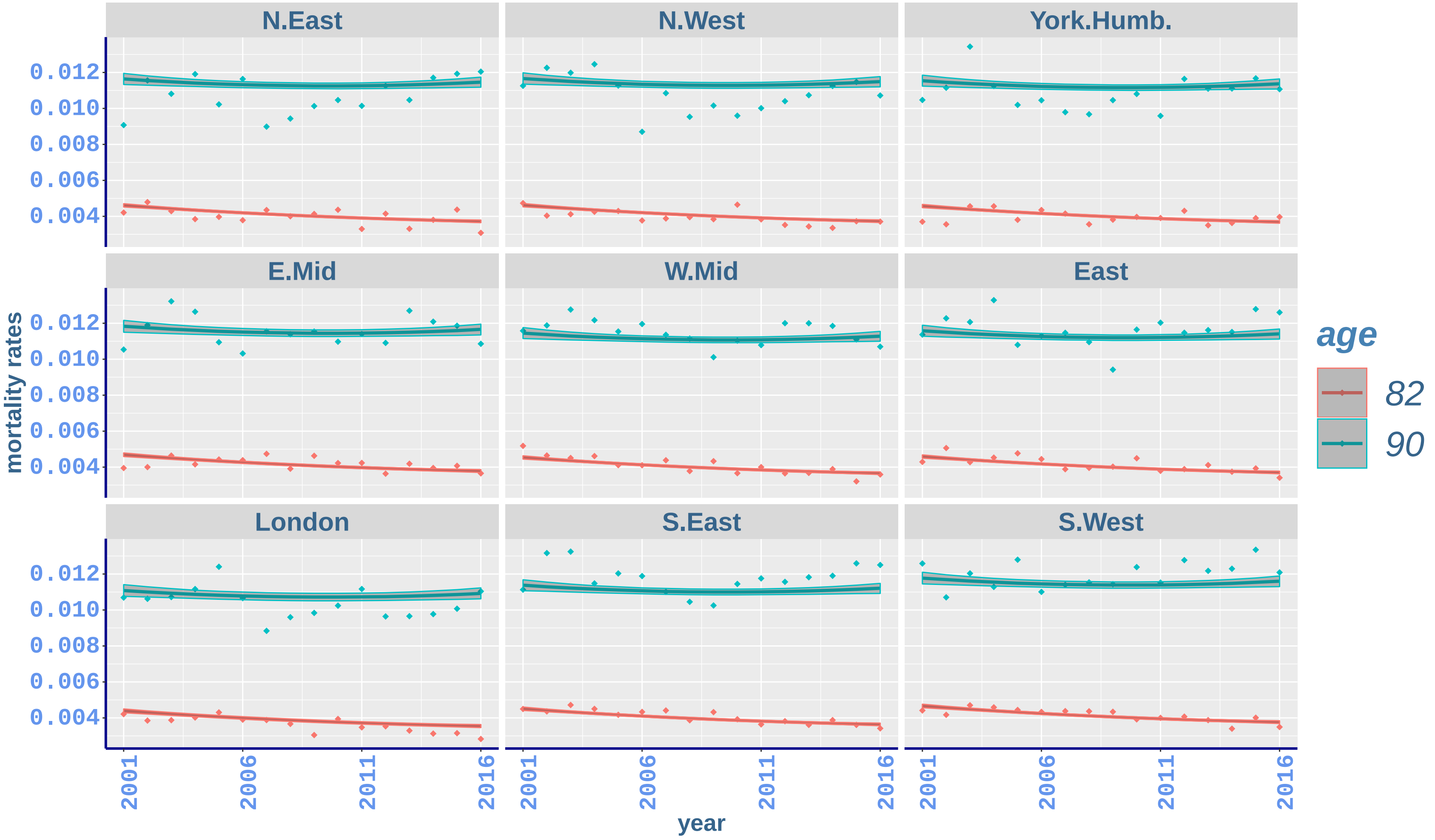

S96 Fig. Prostate cancer mortality, ages 82-90 for all regions in England between 2001 and 2016: observed rates (dots), fitted rates (lines), with 95% credible intervals for the fitted rates.

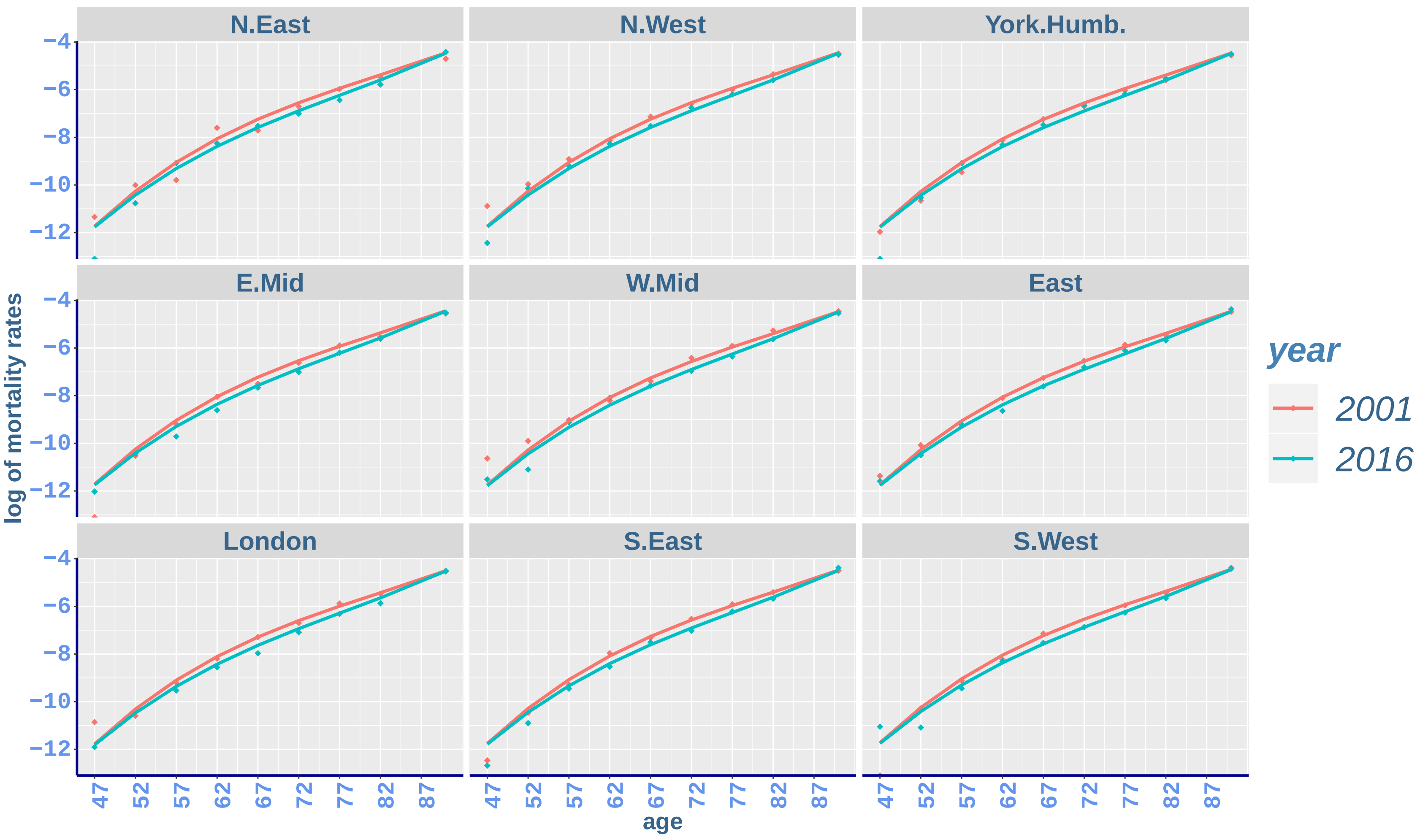

S97 Fig. Prostate cancer mortality for all regions in England in 2001 and 2016: observed rates (dots), fitted rates (lines), with 95% credible intervals for the fitted rates.

S39 Table. Estimated coefficients for the best fitting model for breast cancer morbidity.

Covariate	Parameter	Mean	SD	%2.5	%97.5	Covariate	Parameter	Mean	SD	%2.5	%97.5
Intercept	β_0	-6.6930	0.0038	-6.7000	-6.6850	Deprivation	$\beta_{4,\text{deprivation}_1}$	-0.1011	0.0049	-0.1107	-0.0918
Age	β_{1,age_1}	-4.5020	0.0311	-4.5570	-4.4440		$\beta_{4,\text{deprivation}_2}$	-0.0659	0.0047	-0.0757	-0.0572
	β_{1,age_2}	-2.6270	0.0218	-2.6730	-2.5870		$\beta_{4,\text{deprivation}_3}$	-0.0312	0.0042	-0.0393	-0.0231
	β_{1,age_3}	-1.4850	0.0113	-1.5060	-1.4620		$\beta_{4,\text{deprivation}_4}$	-0.0132	0.0043	-0.0214	-0.0048
	β_{1,age_4}	-0.6698	0.0069	-0.6831	-0.6560		$\beta_{4,\text{deprivation}_5}$	0.0085	0.0041	0.0010	0.0169
	β_{1,age_5}	-0.0166	0.0063	-0.0295	-0.0047		$\beta_{4,\text{deprivation}_6}$	0.0181	0.0040	0.0102	0.0260
	β_{1,age_6}	0.4890	0.0055	0.4787	0.5002		$\beta_{4,\text{deprivation}_7}$	0.0285	0.0040	0.0209	0.0363
	β_{1,age_7}	0.7942	0.0056	0.7829	0.8045		$\beta_{4,\text{deprivation}_8}$	0.0320	0.0038	0.0243	0.0396
	β_{1,age_8}	0.8283	0.0055	0.8170	0.8386		$\beta_{4,\text{deprivation}_9}$	0.0513	0.0037	0.0439	0.0584
	β_{1,age_9}	1.0290	0.0054	1.0170	1.0390		$\beta_{4,\text{deprivation}_{10}}$	0.0731	0.0040	0.0647	0.0809
	$\beta_{1,\text{age}_{10}}$	1.1210	0.0053	1.1110	1.1310	Age:Year	β_{5,age_1}	0.0651	0.0454	-0.0276	0.1420
	$\beta_{1,\text{age}_{11}}$	0.9857	0.0059	0.9735	0.9975		β_{5,age_2}	0.0883	0.0136	0.0623	0.1155
	$\beta_{1,\text{age}_{12}}$	1.1110	0.0053	1.1010	1.1210		β_{5,age_3}	-0.0006	0.0111	-0.0219	0.0212
	$\beta_{1,\text{age}_{13}}$	1.1900	0.0059	1.1780	1.2010		β_{5,age_4}	-0.0417	0.0080	-0.0580	-0.0253
	$\beta_{1,\text{age}_{14}}$	1.7520	0.0060	1.7400	1.7640		β_{5,age_5}	-0.0251	0.0060	-0.0360	-0.0130
Year	β_2	0.0429	0.0041	0.0342	0.0497		β_{5,age_6}	0.0359	0.0060	0.0246	0.0483
Region	$\beta_{3,\text{region}_1}$	-0.0213	0.0053	-0.0318	-0.0114		β_{5,age_7}	-0.0377	0.0055	-0.0483	-0.0268
	$\beta_{3,\text{region}_2}$	-0.0016	0.0038	-0.0087	0.0061		β_{5,age_8}	-0.0818	0.0056	-0.0926	-0.0709
	$\beta_{3,\text{region}_3}$	-0.0258	0.0040	-0.0340	-0.0186		β_{5,age_9}	-0.0460	0.0055	-0.0563	-0.0350
	$\beta_{3,\text{region}_4}$	0.0101	0.0045	0.0018	0.0194		$\beta_{5,\text{age}_{10}}$	0.0397	0.0056	0.0292	0.0511
	$\beta_{3,\text{region}_5}$	0.0081	0.0039	0.0004	0.0157		$\beta_{5,\text{age}_{11}}$	0.0008	0.0058	-0.0096	0.0130
	$\beta_{3,\text{region}_6}$	-0.0014	0.0038	-0.0085	0.0057		$\beta_{5,\text{age}_{12}}$	0.0011	0.0062	-0.0105	0.0136
	$\beta_{3,\text{region}_7}$	-0.0396	0.0039	-0.0469	-0.0321		$\beta_{5,\text{age}_{13}}$	0.0143	0.0058	0.0030	0.0258
	$\beta_{3,\text{region}_8}$	0.0169	0.0034	0.0103	0.0241		$\beta_{5,\text{age}_{14}}$	-0.0123	0.0059	-0.0235	-0.0010
	$\beta_{3,\text{region}_9}$	0.0544	0.0036	0.0473	0.0612		σ^2	0.0042	0.0003	0.0037	0.0049

S40 Table. Estimated coefficients for the best fitting model for breast cancer mortality.

Covariate	Parameter	Mean	SD	%2.5	%97.5	Covariate	Parameter	Mean	SD	%2.5	%97.5
Intercept	β_0	-7.4280	0.0034	-7.4350	-7.4220		$\beta_{4,\text{region}_5}$	0.0007	0.0332	-0.0539	0.0641
Age	β_{1,age_1}	-1.9210	0.0194	-1.9590	-1.8820		$\beta_{4,\text{region}_6}$	0.0442	0.0202	0.0057	0.0781
	β_{1,age_2}	-1.2810	0.0137	-1.3080	-1.2540		$\beta_{4,\text{region}_7}$	-0.0340	0.0481	-0.1113	0.0614
	β_{1,age_3}	-0.7953	0.0112	-0.8174	-0.7735		$\beta_{4,\text{region}_8}$	0.0087	0.0229	-0.0293	0.0546
	β_{1,age_4}	-0.4062	0.0099	-0.4256	-0.3867		$\beta_{4,\text{region}_9}$	-0.0510	0.0662	-0.1578	0.0805
	β_{1,age_5}	-0.1454	0.0091	-0.1629	-0.1275	Age:Year	β_{5,age_1}	-0.0460	0.0187	-0.0821	-0.0086
	β_{1,age_6}	0.0578	0.0087	0.0408	0.0744		β_{5,age_2}	-0.0086	0.0140	-0.0354	0.0193
	β_{1,age_7}	0.2272	0.0087	0.2104	0.2442		β_{5,age_3}	-0.0000	0.0112	-0.0219	0.0220
	β_{1,age_8}	0.4519	0.0084	0.4355	0.4684		β_{5,age_4}	-0.0088	0.0097	-0.0282	0.0112
	β_{1,age_9}	0.7503	0.0080	0.7347	0.7663		β_{5,age_5}	-0.0477	0.0090	-0.0652	-0.0300
	$\beta_{1,\text{age}_{10}}$	1.0360	0.0077	1.0210	1.0510		β_{5,age_6}	-0.0339	0.0091	-0.0518	-0.0161
	$\beta_{1,\text{age}_{11}}$	2.0250	0.0066	2.0120	2.0380		β_{5,age_7}	-0.0096	0.0085	-0.0263	0.0070
Year	β_2	-0.1049	0.0033	-0.1113	-0.0985		β_{5,age_8}	-0.0025	0.0083	-0.0186	0.0139
AAD	β_3	0.1966	0.3226	-0.4475	0.7139		β_{5,age_9}	0.0169	0.0079	0.0011	0.0325
Region	$\beta_{4,\text{region}_1}$	0.0254	0.1028	-0.1790	0.1905		$\beta_{5,\text{age}_{10}}$	0.0659	0.0076	0.0511	0.0808
	$\beta_{4,\text{region}_2}$	0.0119	0.0421	-0.0713	0.0796		$\beta_{5,\text{age}_{11}}$	0.0742	0.0066	0.0613	0.0873
	$\beta_{4,\text{region}_3}$	-0.0061	0.0481	-0.1019	0.0718		σ^2	0.0014	0.0004	0.0007	0.0021
	$\beta_{4,\text{region}_4}$	0.0001	0.0434	-0.0695	0.0863						

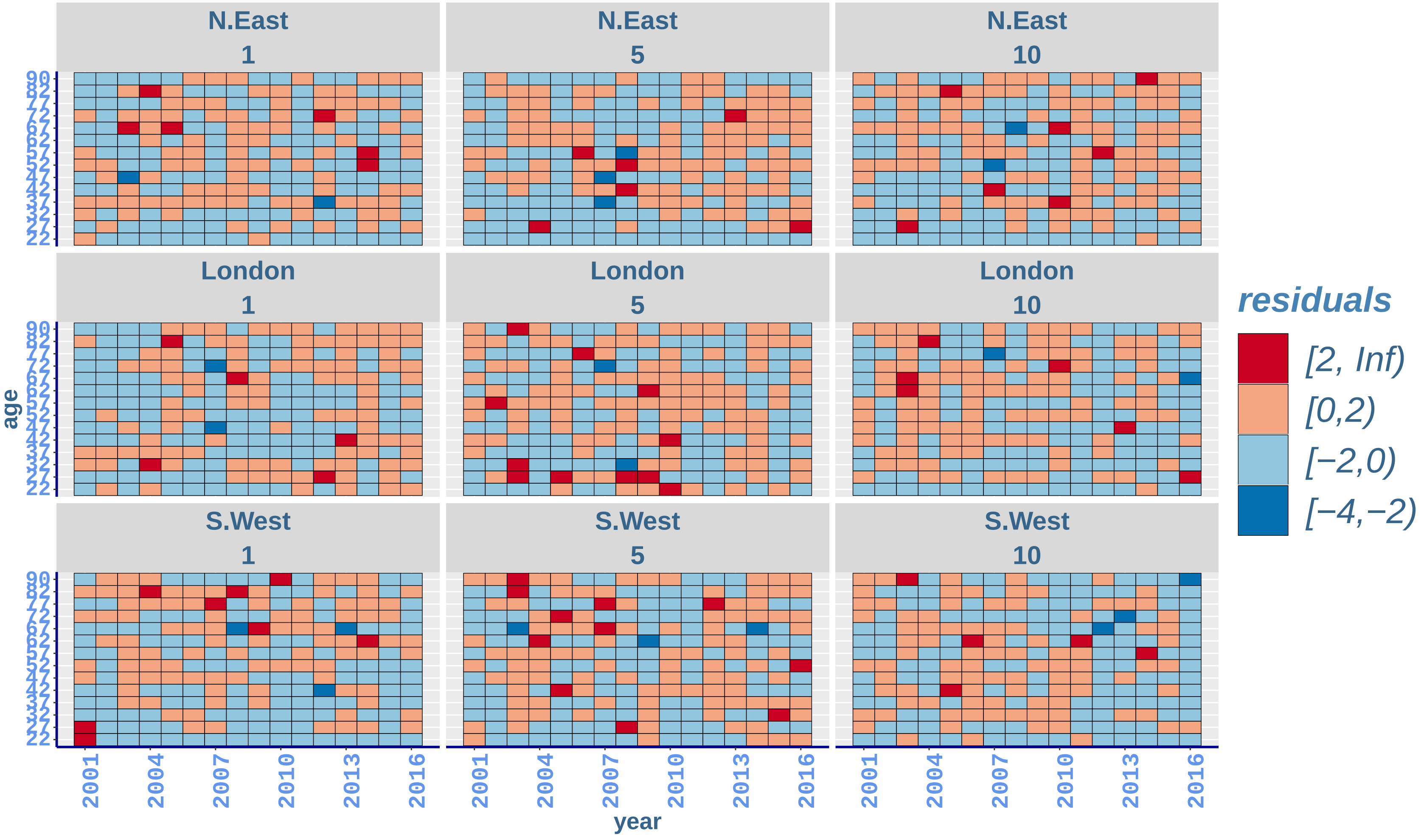

S98 Fig. Heat map of Pearson residuals for breast cancer morbidity in North East, London, and South West, deprivation deciles 1, 5, and 10: orange/light blue cells indicate areas with good fit, while red/dark blue cells indicate areas with poor fit. Note that there is a small number of residuals greater than 4, and these are included in the last category.

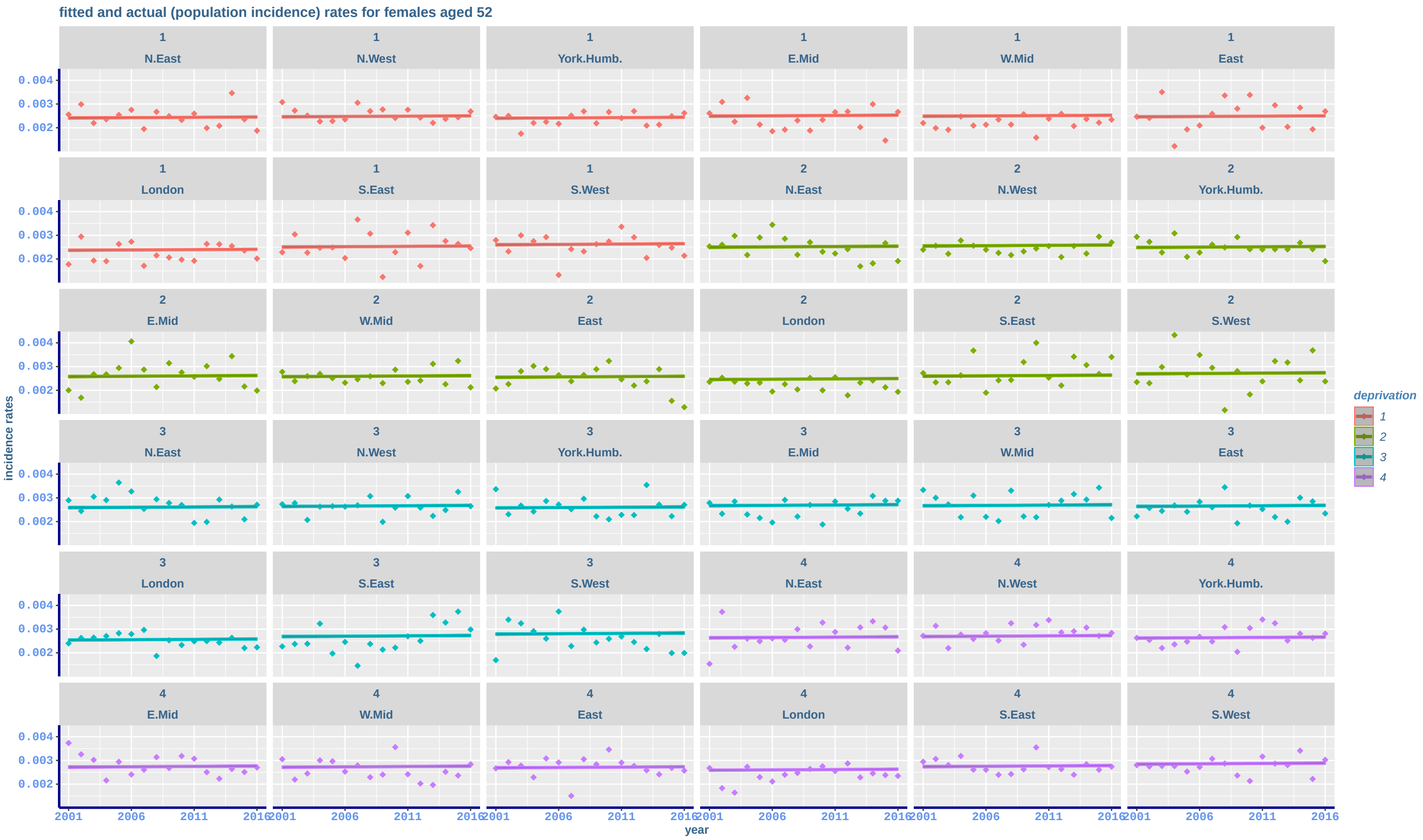

S99 Fig. Breast cancer morbidity, age 52, deprivation deciles 1-4 for all regions in England between 2001 and 2016: observed rates (dots), fitted rates (lines), with 95% credible intervals for the fitted rates.

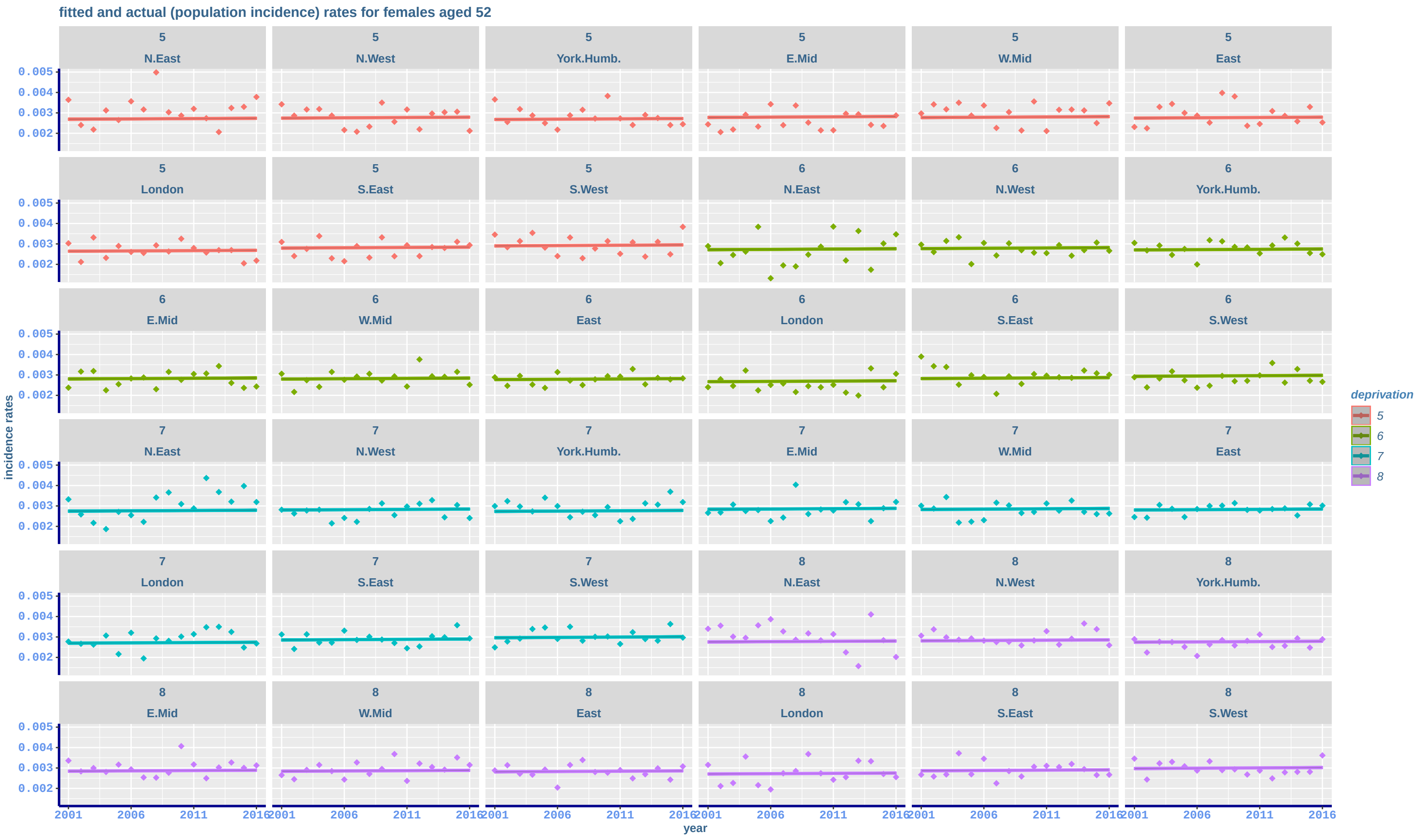

S100 Fig. Breast cancer morbidity, age 52, deprivation deciles 5-8 for all regions in England between 2001 and 2016: observed rates (dots), fitted rates (lines), with 95% credible intervals for the fitted rates.

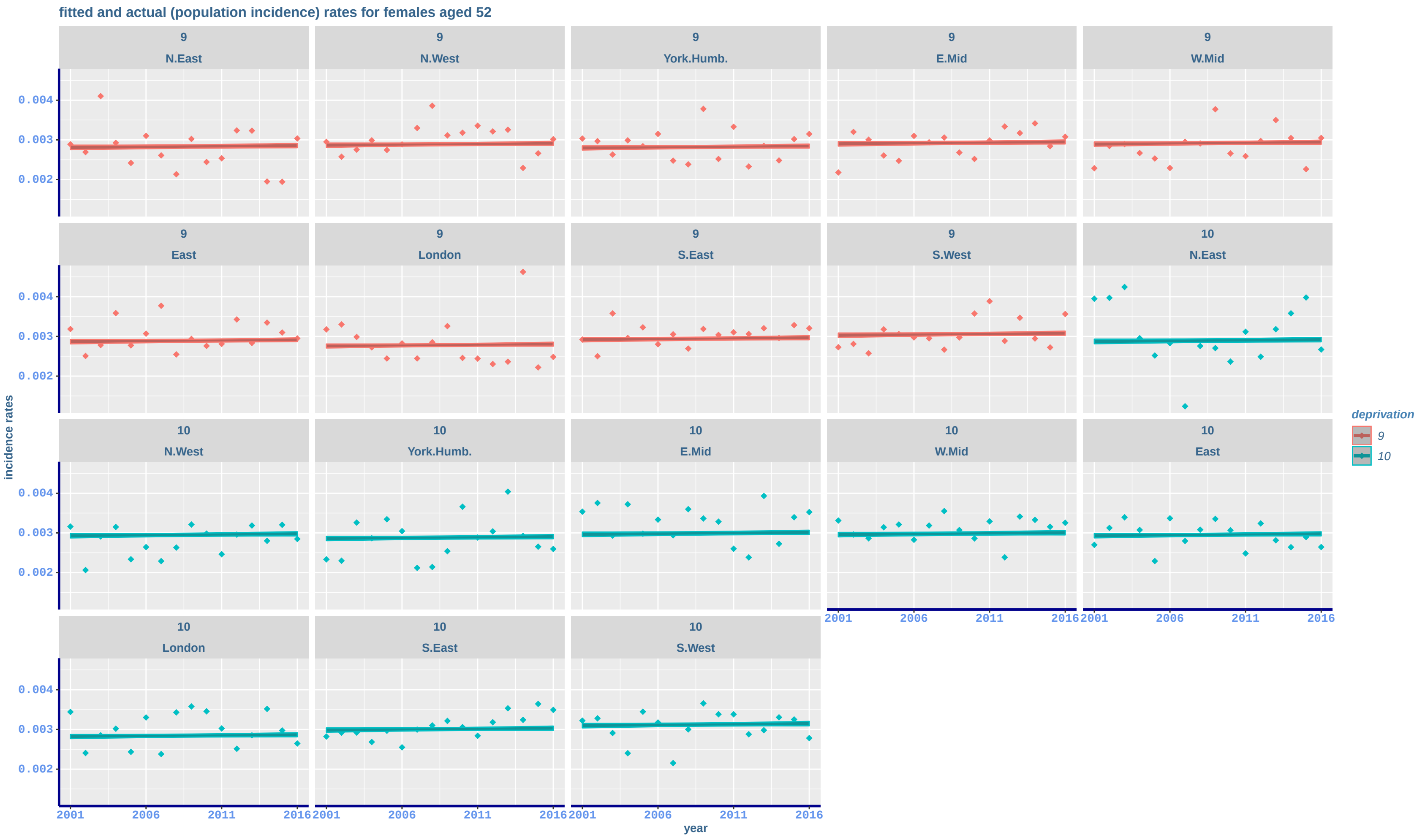

S101 Fig. Breast cancer morbidity, age 52, deprivation deciles 9-10 for all regions in England between 2001 and 2016: observed rates (dots), fitted rates (lines), with 95% credible intervals for the fitted rates.

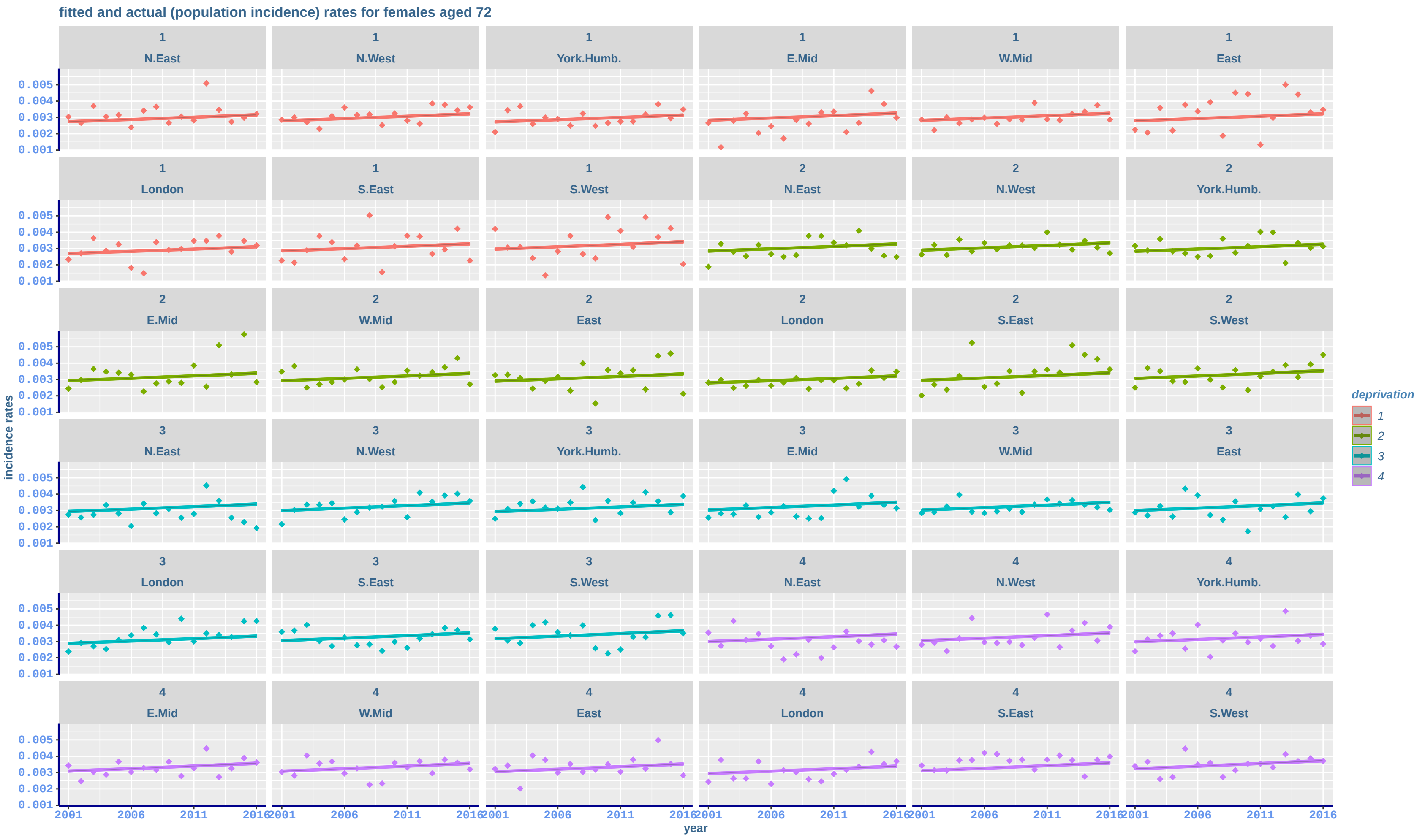

S102 Fig. Breast cancer morbidity, age 72, deprivation deciles 1-4 for all regions in England between 2001 and 2016: observed rates (dots), fitted rates (lines), with 95% credible intervals for the fitted rates.

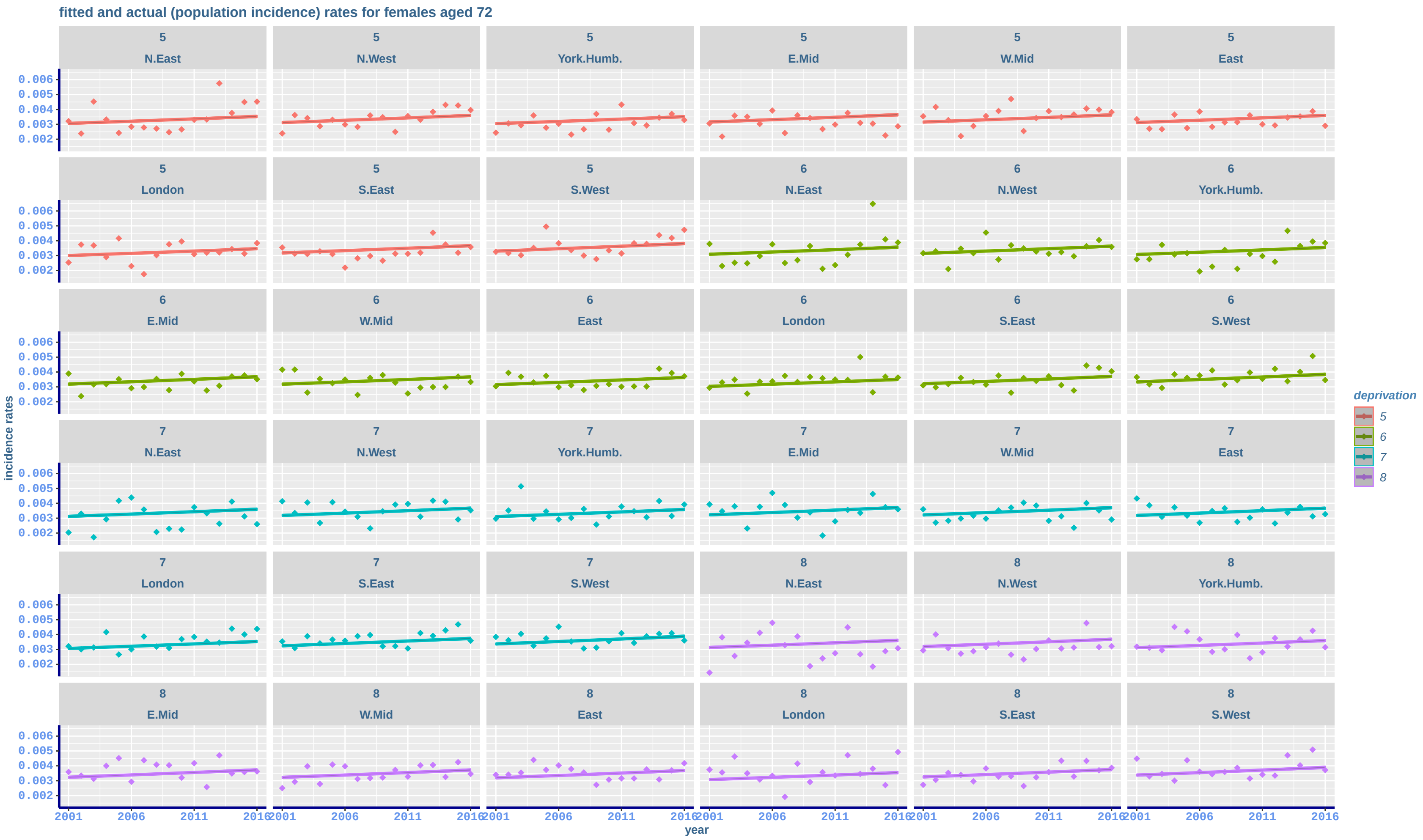

S103 Fig. Breast cancer morbidity, age 72, deprivation deciles 5-8 for all regions in England between 2001 and 2016: observed rates (dots), fitted rates (lines), with 95% credible intervals for the fitted rates.

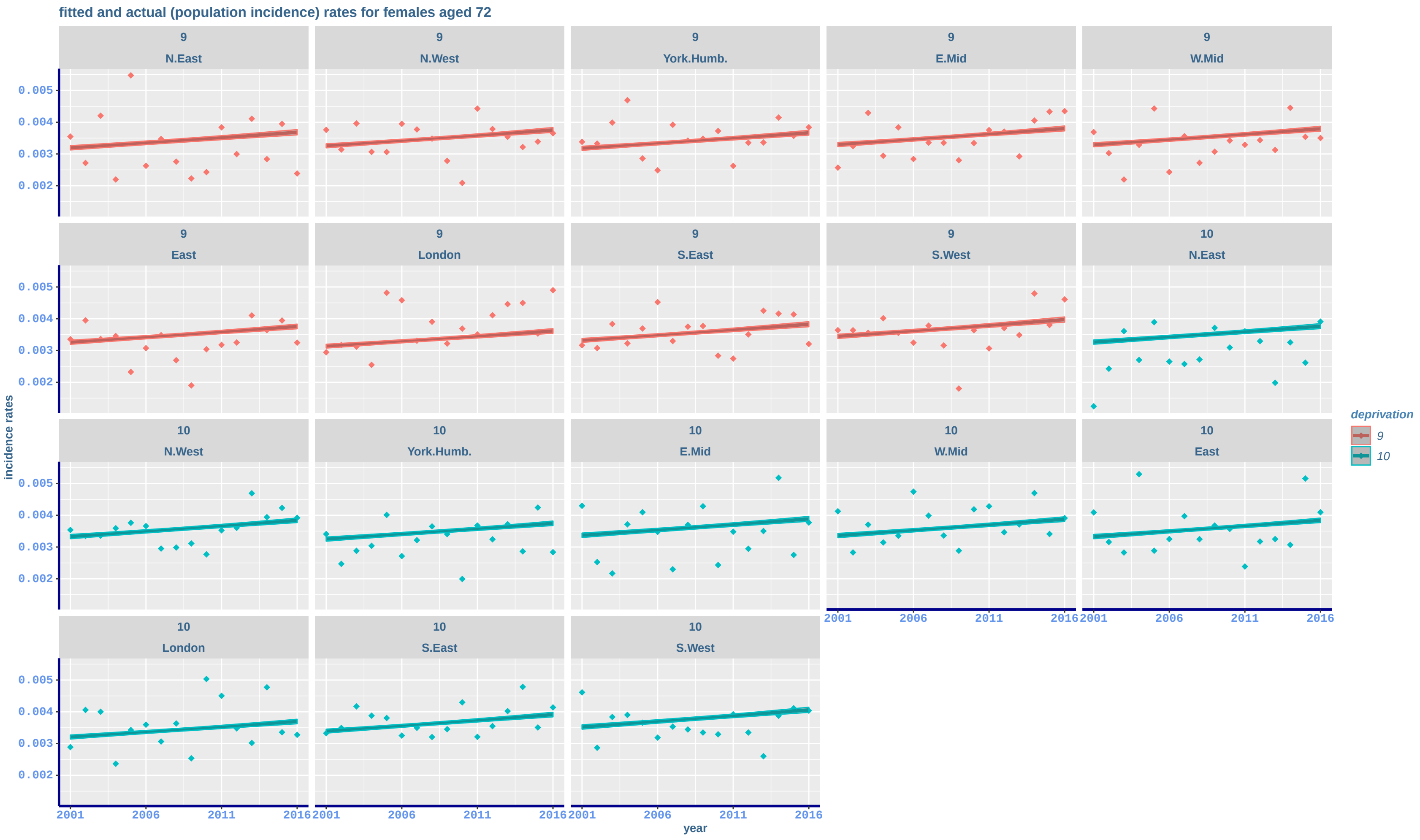

S104 Fig. Breast cancer morbidity, age 72, deprivation deciles 9-10 for all regions in England between 2001 and 2016: observed rates (dots), fitted rates (lines), with 95% credible intervals for the fitted rates.

S41 Table. Absolute deprivation differences ($AD_{t,r}$), per 100,000 people, in age-standardised fitted incidence rates of malignant neoplasm of breast from 2001 to 2016 for all regions in England for males; 95% credible intervals in brackets

year	N.East	N.West	York.Humb.	E.Mid	W.Mid	East	London	S.East	S.West
2001	34 (32, 37)	35 (32, 38)	34 (31, 37)	36 (33, 38)	35 (33, 38)	35 (32, 38)	34 (31, 36)	36 (33, 39)	37 (34, 40)
2002	35 (32, 37)	35 (33, 38)	34 (32, 37)	36 (33, 38)	36 (33, 38)	35 (33, 38)	34 (31, 37)	36 (33, 39)	37 (34, 40)
2003	35 (32, 38)	36 (33, 38)	35 (32, 37)	36 (33, 39)	36 (33, 39)	36 (33, 38)	34 (31, 37)	36 (33, 39)	38 (35, 40)
2004	35 (32, 38)	36 (33, 39)	35 (32, 38)	36 (33, 39)	36 (33, 39)	36 (33, 38)	34 (32, 37)	36 (34, 39)	38 (35, 41)
2005	35 (32, 38)	36 (33, 39)	35 (32, 38)	36 (33, 39)	36 (33, 39)	36 (33, 39)	35 (32, 37)	37 (34, 39)	38 (35, 41)
2006	36 (33, 38)	36 (33, 39)	35 (32, 38)	37 (34, 39)	37 (34, 39)	36 (33, 39)	35 (32, 38)	37 (34, 40)	38 (35, 41)
2007	36 (33, 39)	36 (34, 39)	36 (33, 38)	37 (34, 40)	37 (34, 40)	36 (34, 39)	35 (32, 38)	37 (34, 40)	39 (35, 41)
2008	36 (33, 39)	37 (34, 40)	36 (33, 39)	37 (34, 40)	37 (34, 40)	37 (34, 39)	35 (32, 38)	37 (34, 40)	39 (36, 42)
2009	36 (33, 39)	37 (34, 40)	36 (33, 39)	37 (34, 40)	37 (34, 40)	37 (34, 40)	36 (33, 38)	38 (35, 40)	39 (36, 42)
2010	36 (34, 39)	37 (34, 40)	36 (33, 39)	38 (35, 40)	38 (34, 40)	37 (34, 40)	36 (33, 39)	38 (35, 41)	39 (36, 42)
2011	37 (34, 40)	37 (34, 40)	37 (33, 39)	38 (35, 41)	38 (35, 41)	37 (34, 40)	36 (33, 39)	38 (35, 41)	40 (36, 43)
2012	37 (34, 40)	38 (35, 41)	37 (34, 40)	38 (35, 41)	38 (35, 41)	38 (35, 41)	36 (33, 39)	38 (35, 41)	40 (37, 43)
2013	37 (34, 40)	38 (35, 41)	37 (34, 40)	38 (35, 41)	38 (35, 41)	38 (35, 41)	36 (34, 39)	39 (36, 42)	40 (37, 43)
2014	37 (34, 40)	38 (35, 41)	37 (34, 40)	39 (35, 42)	39 (35, 41)	38 (35, 41)	37 (34, 40)	39 (36, 42)	40 (37, 43)
2015	38 (35, 41)	38 (35, 41)	38 (34, 40)	39 (36, 42)	39 (36, 42)	38 (35, 41)	37 (34, 40)	39 (36, 42)	41 (37, 44)
2016	38 (35, 41)	39 (36, 42)	38 (35, 41)	39 (36, 42)	39 (36, 42)	39 (36, 42)	37 (34, 40)	39 (36, 42)	41 (38, 44)

S42 Table. Relative deprivation differences ($\mathrm{RD}_{t,r}$) in age-standardised fitted incidence rates of malignant neoplasm of breast from 2001 to 2016 for all regions in England for males; 95% credible intervals in brackets

year	N.East	N.West	York.Humb.	E.Mid	W.Mid	East	London	S.East	S.West
2001	0.1894 (0.1726, 0.2053)	0.1894 (0.1726, 0.2053)	0.1894 (0.1726, 0.2053)	0.1894 (0.1726, 0.2053)	0.1894 (0.1726, 0.2052)	0.1894 (0.1727, 0.2052)	0.1894 (0.1727, 0.2053)	0.1894 (0.1726, 0.2053)	0.1894 (0.1727, 0.2053)
2002	0.1894 (0.1726, 0.2052)	0.1894 (0.1727, 0.2052)	0.1894 (0.1726, 0.2053)	0.1894 (0.1727, 0.2052)	0.1894 (0.1727, 0.2053)	0.1894 (0.1726, 0.2052)	0.1894 (0.1726, 0.2052)	0.1894 (0.1726, 0.2053)	0.1894 (0.1726, 0.2052)
2003	0.1894 (0.1726, 0.2052)	0.1894 (0.1726, 0.2053)	0.1894 (0.1726, 0.2053)	0.1894 (0.1726, 0.2053)	0.1894 (0.1727, 0.2053)	0.1894 (0.1726, 0.2052)	0.1894 (0.1726, 0.2053)	0.1894 (0.1726, 0.2052)	0.1894 (0.1726, 0.2052)
2004	0.1894 (0.1726, 0.2052)	0.1894 (0.1726, 0.2053)	0.1894 (0.1726, 0.2053)	0.1894 (0.1727, 0.2053)	0.1894 (0.1726, 0.2053)	0.1894 (0.1727, 0.2053)	0.1894 (0.1727, 0.2052)	0.1894 (0.1726, 0.2053)	0.1894 (0.1726, 0.2053)
2005	0.1894 (0.1726, 0.2053)	0.1894 (0.1727, 0.2053)	0.1894 (0.1726, 0.2053)	0.1894 (0.1726, 0.2053)	0.1894 (0.1726, 0.2052)	0.1894 (0.1726, 0.2052)	0.1894 (0.1727, 0.2053)	0.1894 (0.1726, 0.2052)	0.1894 (0.1726, 0.2052)
2006	0.1894 (0.1726, 0.2052)	0.1894 (0.1726, 0.2053)	0.1894 (0.1726, 0.2053)	0.1894 (0.1726, 0.2052)	0.1894 (0.1726, 0.2053)	0.1894 (0.1726, 0.2053)	0.1894 (0.1726, 0.2052)	0.1894 (0.1726, 0.2053)	0.1894 (0.1727, 0.2053)
2007	0.1894 (0.1726, 0.2053)	0.1894 (0.1727, 0.2052)	0.1894 (0.1726, 0.2053)	0.1894 (0.1727, 0.2053)	0.1894 (0.1726, 0.2053)	0.1894 (0.1726, 0.2052)	0.1894 (0.1727, 0.2052)	0.1894 (0.1726, 0.2053)	0.1894 (0.1727, 0.2053)
2008	0.1894 (0.1726, 0.2053)	0.1894 (0.1726, 0.2052)	0.1894 (0.1727, 0.2052)	0.1894 (0.1727, 0.2053)	0.1894 (0.1727, 0.2053)	0.1894 (0.1726, 0.2052)	0.1894 (0.1726, 0.2052)	0.1894 (0.1726, 0.2052)	0.1894 (0.1726, 0.2052)
2009	0.1894 (0.1726, 0.2052)	0.1894 (0.1726, 0.2052)	0.1894 (0.1726, 0.2053)	0.1894 (0.1726, 0.2052)	0.1894 (0.1726, 0.2052)	0.1894 (0.1727, 0.2053)	0.1894 (0.1726, 0.2052)	0.1894 (0.1726, 0.2053)	0.1894 (0.1726, 0.2052)
2010	0.1894 (0.1726, 0.2052)	0.1894 (0.1726, 0.2053)	0.1894 (0.1726, 0.2053)	0.1894 (0.1726, 0.2053)	0.1894 (0.1726, 0.2053)	0.1894 (0.1726, 0.2053)	0.1894 (0.1726, 0.2053)	0.1894 (0.1727, 0.2053)	0.1894 (0.1726, 0.2053)
2011	0.1894 (0.1726, 0.2053)	0.1894 (0.1726, 0.2053)	0.1894 (0.1726, 0.2052)	0.1894 (0.1726, 0.2053)	0.1894 (0.1726, 0.2053)	0.1894 (0.1727, 0.2053)	0.1894 (0.1727, 0.2053)	0.1894 (0.1725, 0.2052)	0.1894 (0.1726, 0.2052)
2012	0.1894 (0.1727, 0.2052)	0.1894 (0.1727, 0.2053)	0.1894 (0.1726, 0.2053)	0.1894 (0.1726, 0.2053)	0.1894 (0.1727, 0.2053)	0.1894 (0.1726, 0.2053)	0.1894 (0.1726, 0.2053)	0.1894 (0.1727, 0.2052)	0.1894 (0.1726, 0.2052)
2013	0.1894 (0.1726, 0.2053)	0.1894 (0.1727, 0.2053)	0.1894 (0.1726, 0.2053)	0.1894 (0.1726, 0.2052)	0.1894 (0.1726, 0.2053)	0.1894 (0.1727, 0.2052)	0.1894 (0.1726, 0.2053)	0.1894 (0.1726, 0.2053)	0.1894 (0.1727, 0.2052)
2014	0.1894 (0.1727, 0.2052)	0.1894 (0.1727, 0.2053)	0.1894 (0.1726, 0.2052)	0.1894 (0.1727, 0.2053)	0.1894 (0.1726, 0.2052)	0.1894 (0.1726, 0.2053)	0.1894 (0.1726, 0.2053)	0.1894 (0.1725, 0.2052)	0.1894 (0.1726, 0.2053)
2015	0.1894 (1726, 0.2052)	0.1894 (0.1726, 0.2053)	0.1894 (0.1726, 0.2053)	0.1894 (0.1726, 0.2053)	0.1894 (0.1726, 0.2053)	0.1894 (0.1726, 0.2053)	0.1894 (0.1726, 0.2052)	0.1894 (0.1726, 0.2053)	0.1894 (0.1726, 0.2053)
2016	0.1894 (0.1727, 0.2053)	0.1894 (0.1726, 0.2053)	0.1894 (0.1726, 0.2052)	0.1894 (0.1726, 0.2053)	0.1894 (0.1726, 0.2053)	0.1894 (0.1726, 0.2053)	0.1894 (0.1726, 0.2053)	0.1894 (0.1726, 0.2053)	0.1894 (0.1725, 0.2053)

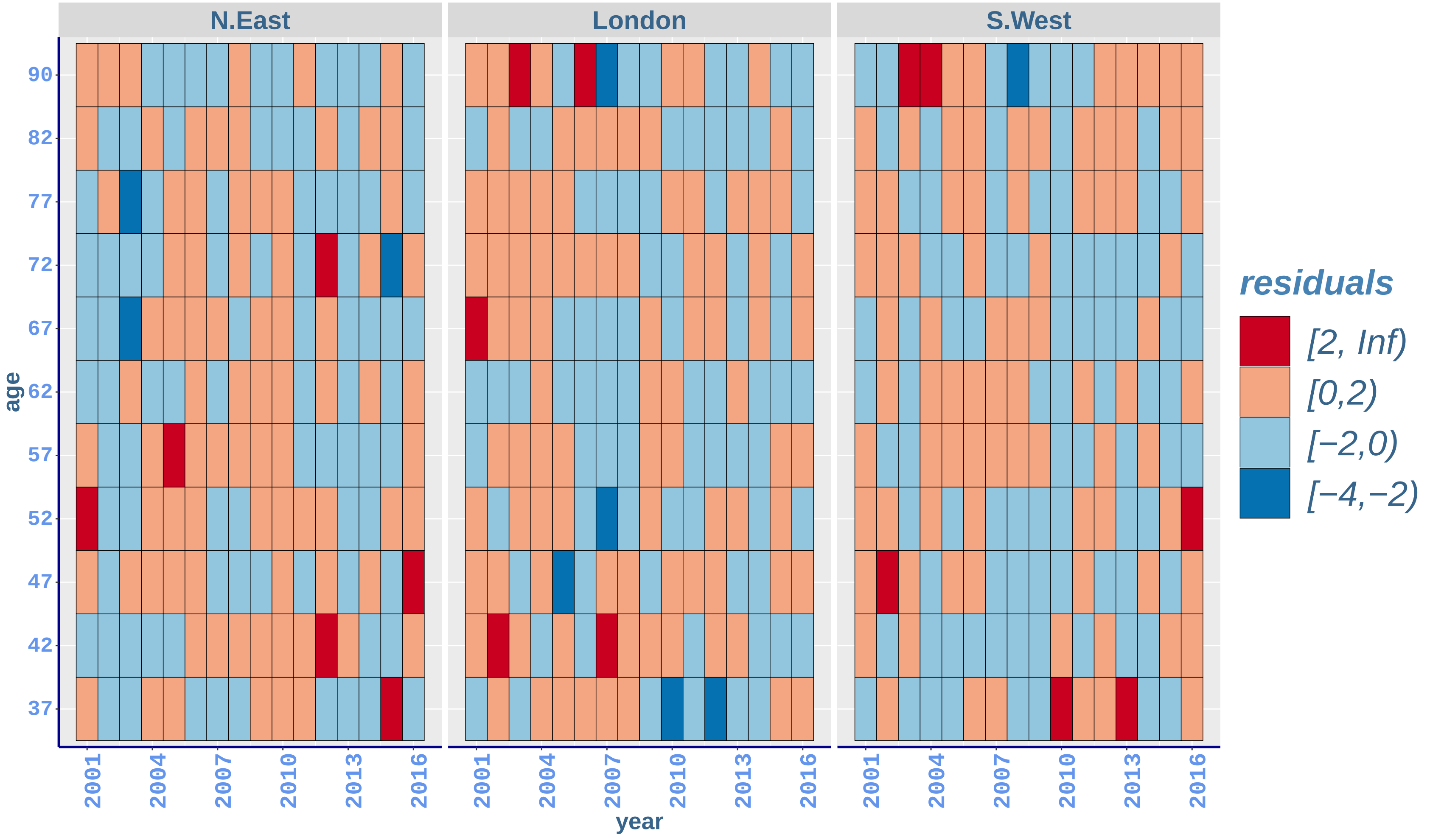

S105 Fig. Heat map of Pearson residuals for breast cancer mortality in North East, London, and South West, deprivation deciles 1, 5, and 10: orange/light blue cells indicate areas with good fit, while red/dark blue cells indicate areas with poor fit. Note that there is a small number of residuals greater than 4, and these are included in the last category.

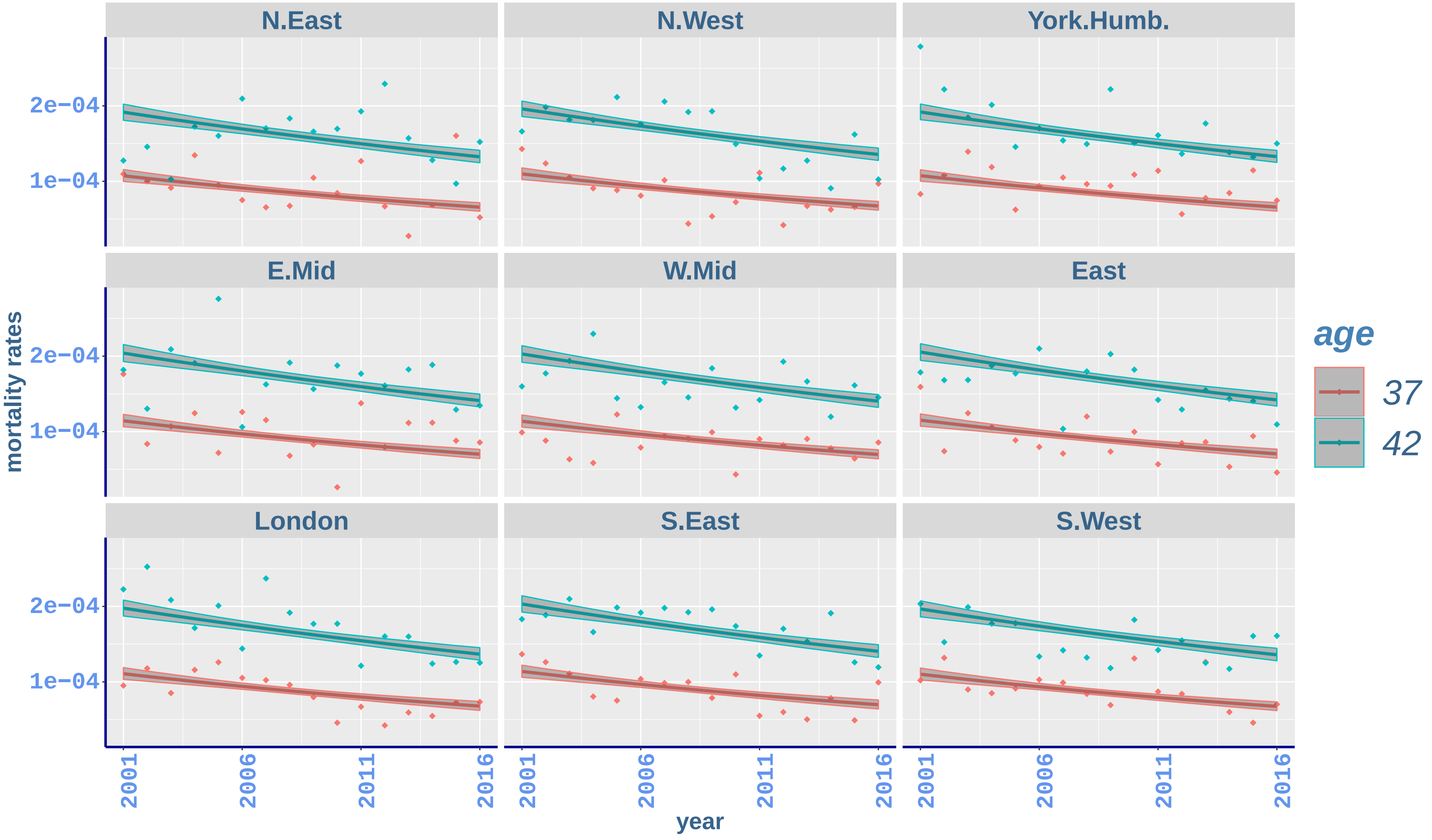

S106 Fig. Breast cancer mortality, ages 37-42 for all regions in England between 2001 and 2006: observed rates (dots), fitted rates (lines), with 95% credible intervals for the fitted rates.

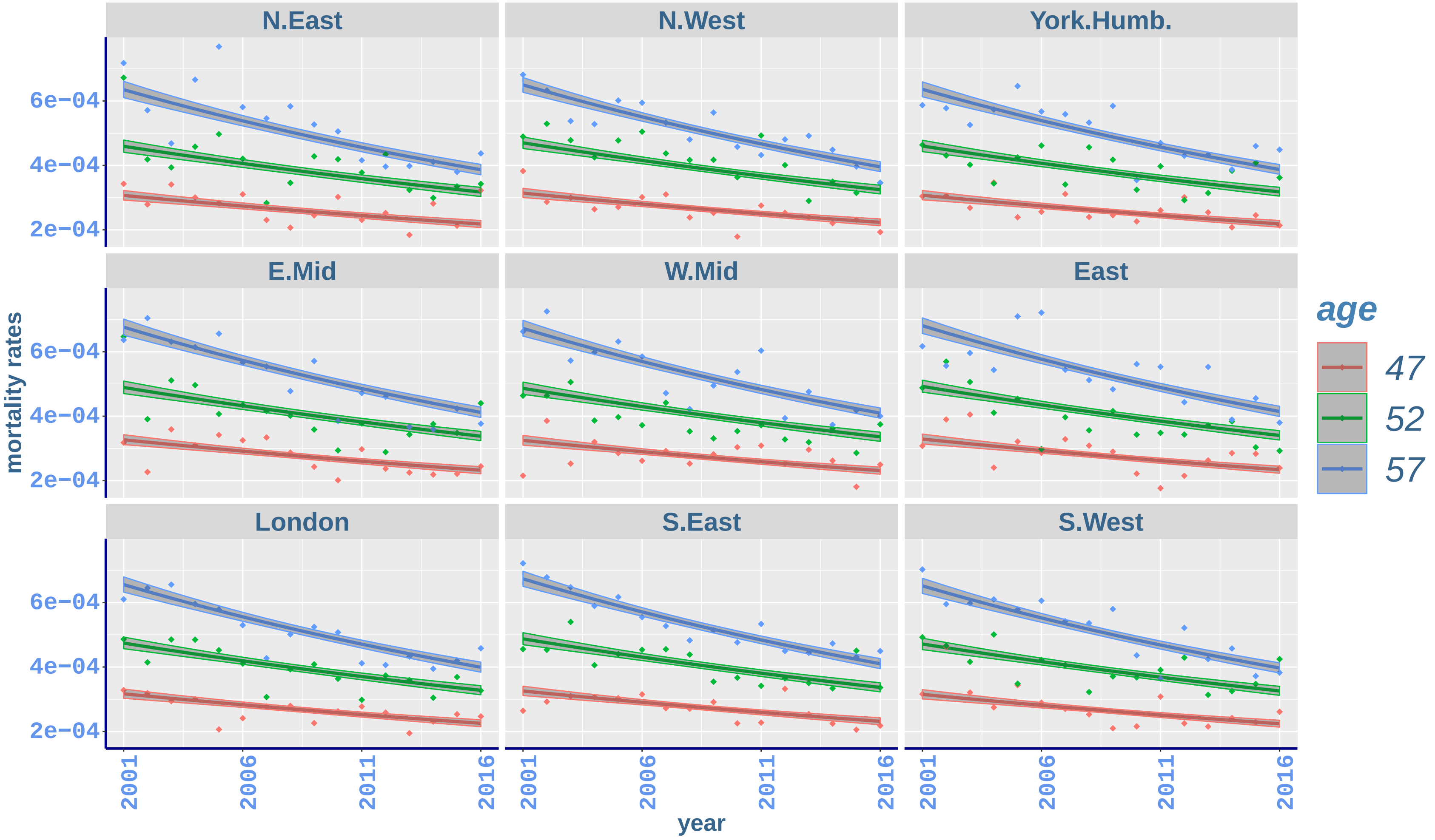

S107 Fig. Breast cancer mortality, ages 47-57 for all regions in England between 2001 and 2016: observed rates (dots), fitted rates (lines), with 95% credible intervals for the fitted rates.

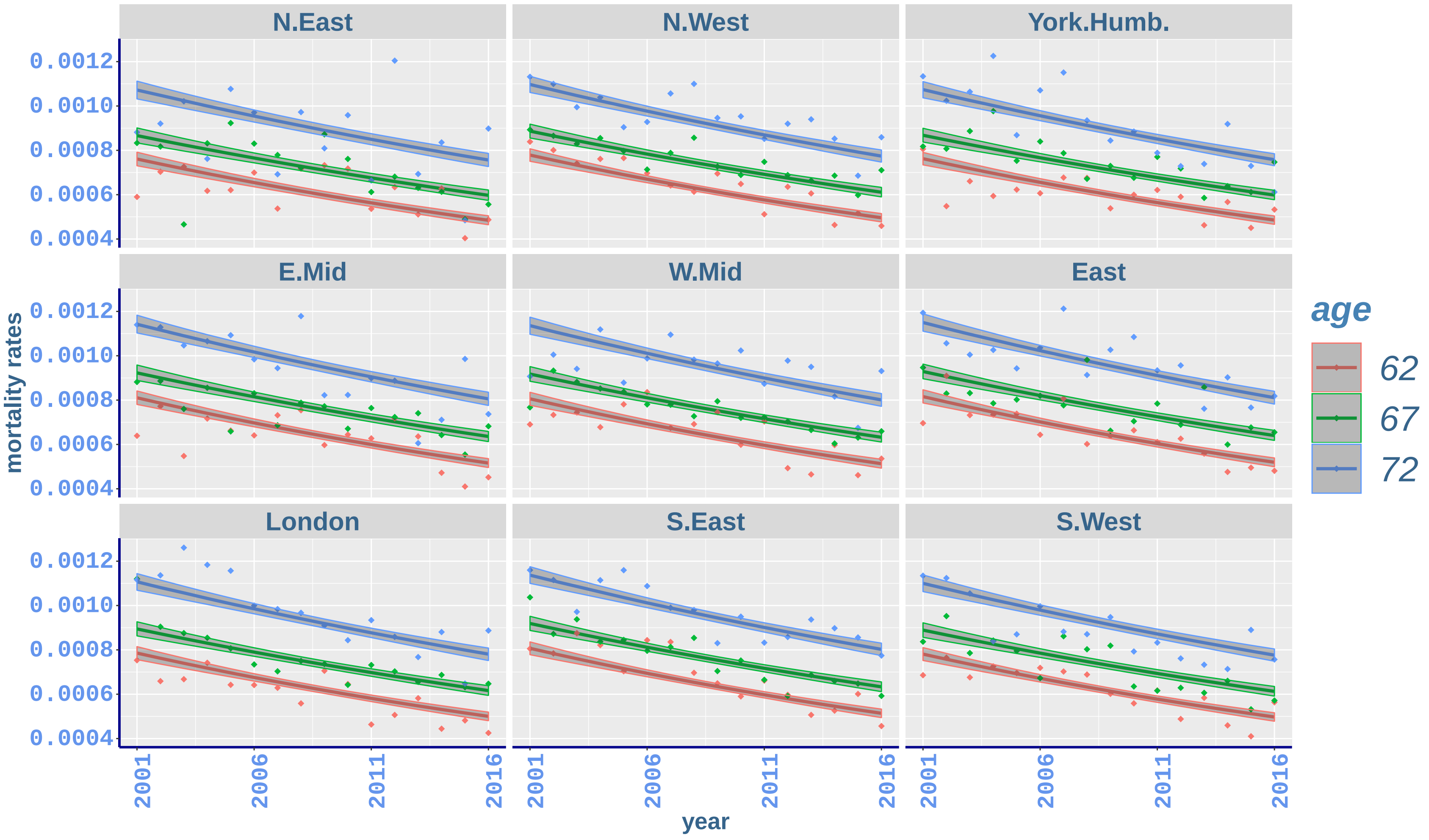

S108 Fig. Breast cancer mortality, ages 62-72 for all regions in England between 2001 and 2016: observed rates (dots), fitted rates (lines), with 95% credible intervals for the fitted rates.

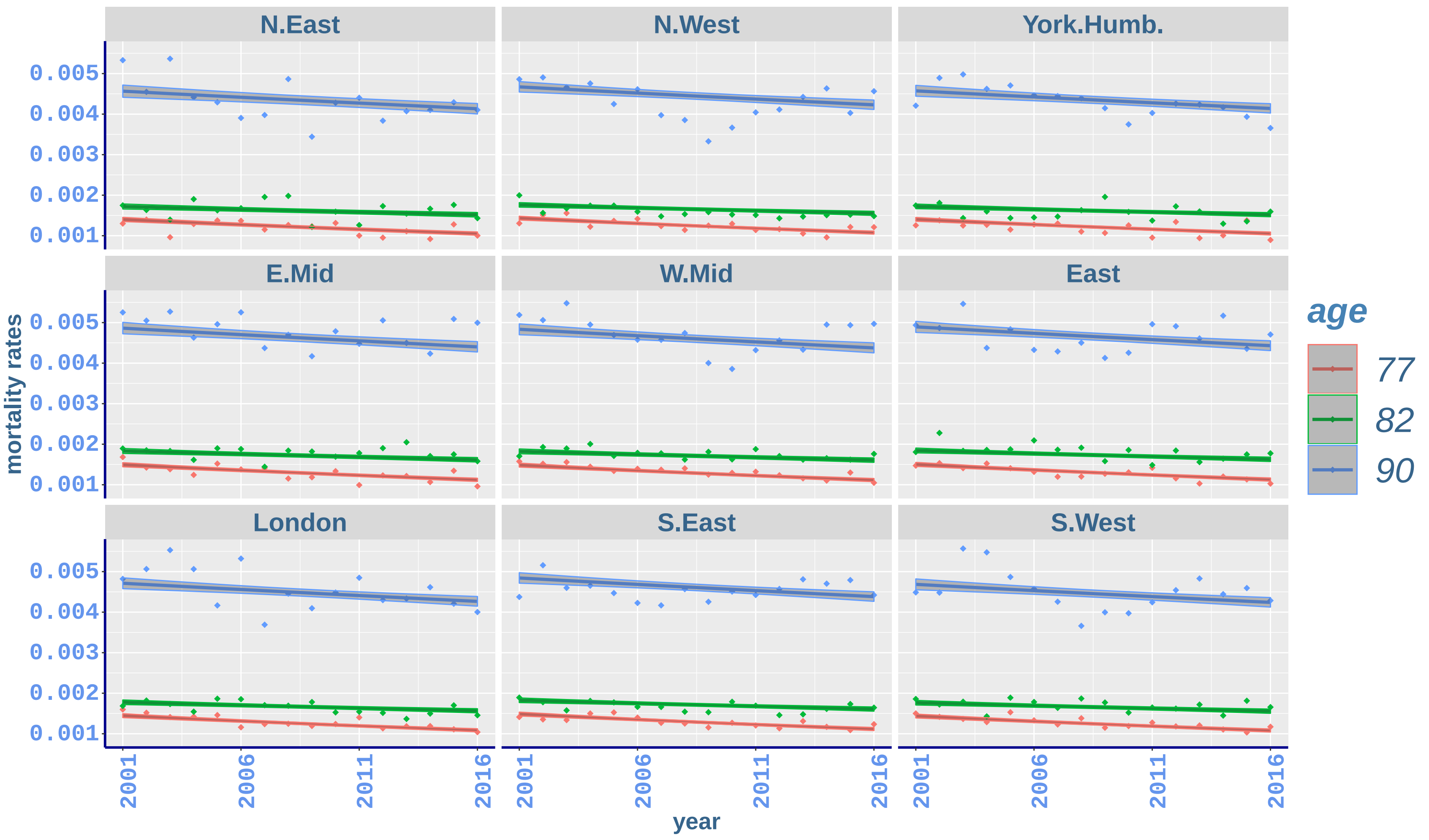

S109 Fig. Breast cancer mortality, ages 77-90 for all regions in England between 2001 and 2016: observed rates (dots), fitted rates (lines), with 95% credible intervals for the fitted rates.

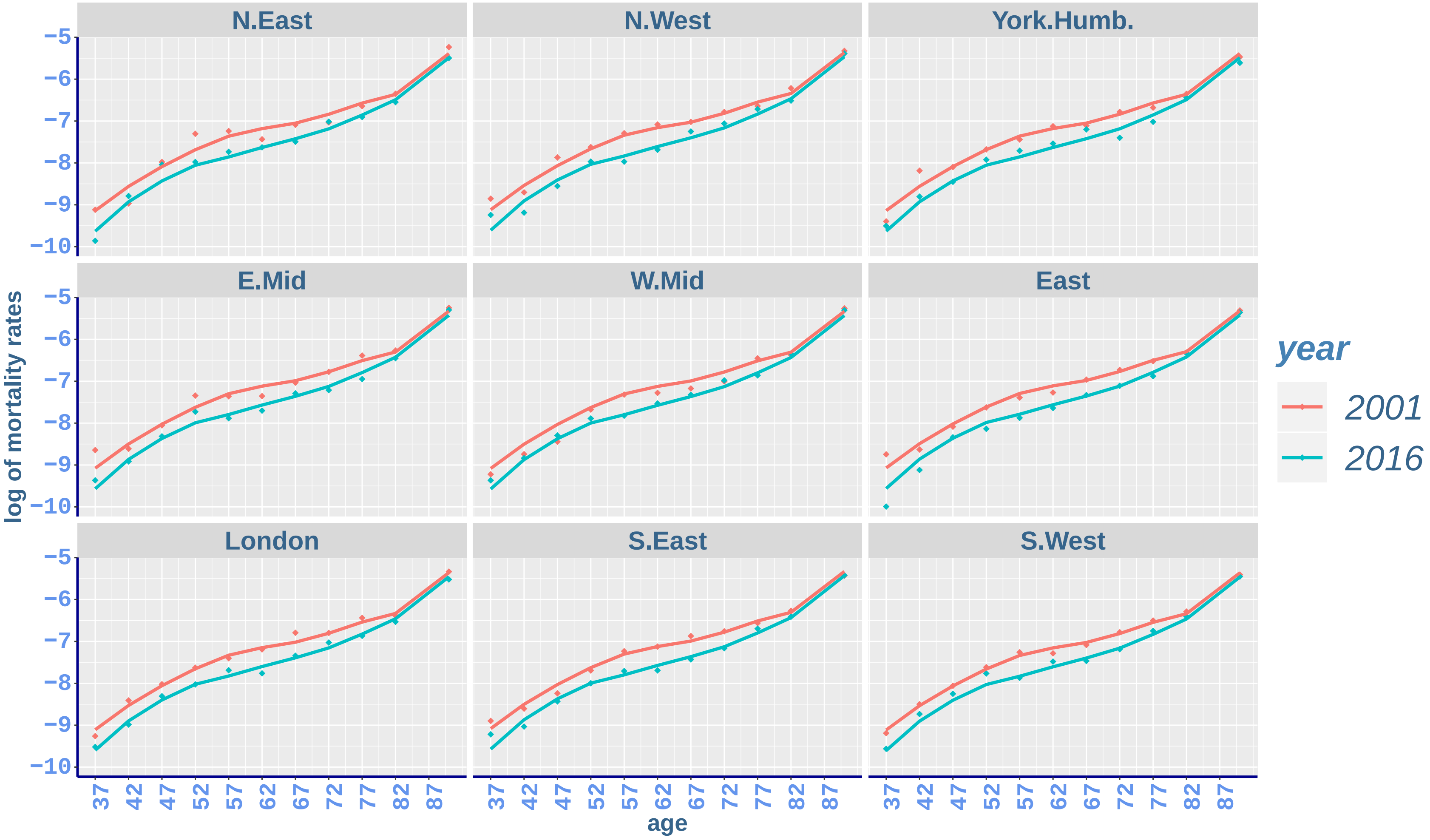

S110 Fig. Breast cancer mortality for all regions in England in 2001 and 2016: observed rates (dots), fitted rates (lines), with 95% credible intervals for the fitted rates.

S43 Table. Absolute regional differences (AD_t), per 100,000 people, and relative regional differences (RD_t) in age-standardised fitted mortality rates of prostate and breast cancers from 2001 to 2016; 95% credible intervals in brackets

	prostate cancer		breast cancer	
year	AD	RD	AD	RD
2001	14<br>(11, 17)	0.1083<br>(0.0841, 0.1344)	6<br>(5, 8)	0.0804<br>(0.06, 0.1044)
2002	14<br>(11, 17)	0.1087<br>(0.0844, 0.1347)	6<br>(5, 8)	0.0804<br>(0.06, 0.1044)
2003	14<br>(11, 17)	0.1091<br>(0.0849, 0.1351)	6<br>(5, 8)	0.0804<br>(0.06, 0.1044)
2004	14<br>(11, 17)	0.1095<br>(0.0851, 0.1355)	6<br>(5, 8)	0.0804<br>(0.06, 0.1044)
2005	14<br>(11, 17)	0.1099<br>(0.0855, 0.1359)	6<br>(4, 8)	0.0804<br>(0.06, 0.1044)
2006	13<br>(11, 16)	0.1103<br>(0.0857, 0.1364)	6<br>(4, 7)	0.0804<br>(0.06, 0.1044)
2007	13<br>(11, 16)	0.1107<br>(0.086, 0.1369)	6<br>(4, 7)	0.0804<br>(0.06, 0.1044)
2008	13<br>(10, 16)	0.1111<br>(0.0862, 0.1373)	6<br>(4, 7)	0.0804<br>(0.06, 0.1044)
2009	13<br>(10, 16)	0.1115<br>(0.0868, 0.1376)	5<br>(4, 7)	0.0804<br>(0.06, 0.1044)
2010	13<br>(10, 16)	0.1119<br>(0.0871, 0.1381)	5<br>(4, 7)	0.0804<br>(0.06, 0.1044)
2011	13<br>(10, 16)	0.1123<br>(0.0875, 0.1385)	5<br>(4, 7)	0.0804<br>(0.06, 0.1044)
2012	13<br>(10, 16)	0.1127<br>(0.0878, 0.1391)	5<br>(4, 7)	0.0804<br>(0.06, 0.1044)
2013	13<br>(10, 16)	0.1131<br>(0.0881, 0.1395)	5<br>(4, 6)	0.0804<br>(0.06, 0.1044)
2014	13<br>(10, 16)	0.1135<br>(0.0885, 0.1399)	5<br>(4, 6)	0.0804<br>(0.06, 0.1044)
2015	13<br>(10, 16)	0.1139<br>(0.0887, 0.1405)	5<br>(4, 6)	0.0804<br>(0.06, 0.1044)
2016	13<br>(10, 16)	0.1143<br>(0.0889, 0.1408)	5<br>(4, 6)	0.0804<br>(0.06, 0.1044)
